# Reductive radical chain initiation through the thermal generation of carbon dioxide radical anion

**DOI:** 10.1038/s44160-025-00919-z

**Published:** 2025-11-06

**Authors:** Ethan R. X. Lim, Bradley D. Cooper, Muralidharan Shanmugam, Jonathan Da Luz, Eric J. L. McInnes, Cristina Trujillo, James J. Douglas, Michael J. James

**Affiliations:** 1 Department of Chemistry, The University of Manchester, Manchester, UK; 2 Early Chemical Development, Pharmaceutical Sciences, AstraZeneca R&D, Macclesfield, UK

**Keywords:** Synthetic chemistry methodology, Reaction mechanisms

## Abstract

Radical chain initiation strategies are fundamental to the synthesis of small molecule drugs and macromolecular materials. Modern methods for initiation through one-electron reduction are largely dominated by photo- and electrochemistry but the large-scale industrial application of these methods is often hampered by scalability challenges. Here we report a general, thermally driven and scalable method for the reductive initiation of radical chains that involves reacting an inexpensive azo initiator with a formate salt to form a carbon dioxide radical anion. Substoichiometric quantities of this initiator system were used to form C(*sp*^2^)–C(*sp*^3^), C(*sp*^2^)–S, C(*sp*^2^)–H, C(*sp*^2^)–B and C(*sp*^2^)–P bonds from complex (hetero)aryl halides, with high chemoselectivity and under transition-metal-free conditions. The developed initiator system was also used to probe the mechanism of other radical reactions.

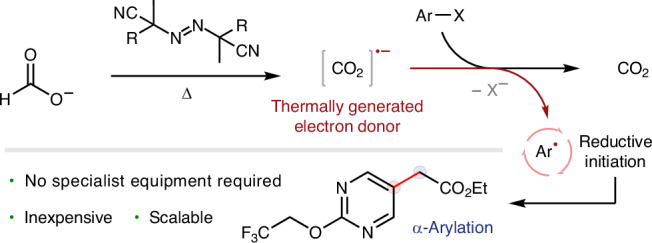

## Abstract (web summary)

Radical chain initiation strategies are fundamental to the synthesis of small molecule drugs and macromolecular materials. Here a general, thermally driven and scalable method for reductive initiation is reported, in which inexpensive azo initiators are reacted with formate salts to form a carbon dioxide radical anion.

## Main

**Fig. 1:**
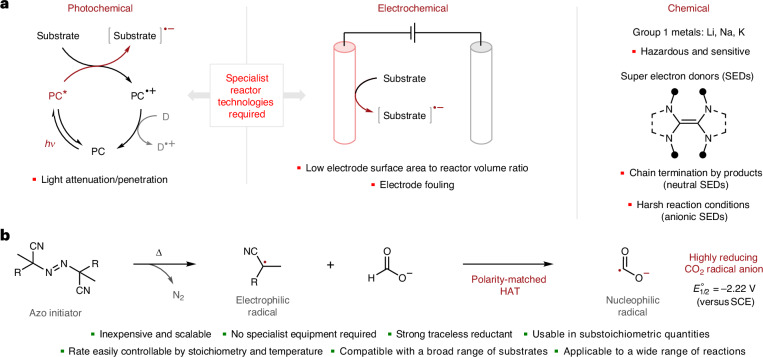
Background and mechanistic hypothesis. **a**, One-electron reduction initiation strategies. **b**, Proposed thermal strategy using azo initiators to generate carbon dioxide radical anion. PC, photocatalyst; D, donor; ${E}_{1/2}^{\circ}$, half-wave potential.

The controlled initiation of radical chains is a subject of fundamental importance to polymer science^1^, organic synthesis^2,3^, atmospheric chemistry^4^ and biochemistry^5^. In the context of synthetic organic chemistry, a substantial proportion of radical reactions are driven by chains^6,7^, but this aspect is often obscured—especially if the length of the chain is short. If the chain length is short, the reaction must be continuously reinitiated for its duration^8^. This is a major reason why photo- and electrochemistry have emerged as leading methods of initiation: they provide flexible and programmable frameworks to promote electron transfer and generate radicals at a controllable rate^9–13^. One drawback of photo-/electrochemical initiation strategies is their non-trivial scalability in a process development and manufacturing setting (beyond the milligram to gram scales used in earlier stages of medicinal chemistry)^14,15^. Indeed, both strategies require specialist reactor technologies, which may increase process development times and costs, limiting applications on the industrial scale when compared to standard manufacturing techniques (Fig. 1a). However, for chains driven by one-electron reduction (electron-catalysed and electron-transfer chain processes)^16,17^ photo- and electrochemical methods still far surpass the general utility of chemical/thermal initiation strategies using ground state electron donors^18,19^. Considering this current state-of-the-art, we sought to develop a general reductive initiation system with the following characteristics: (1) strongly reducing (${{E}_{1/2}^{\circ}} < -2\,{\rm{V}}$ versus saturated calomel electrode (SCE)); (2) usable in substoichiometric quantities; (3) thermally controlled; (4) does not form chain-terminating persistent radicals (a traceless one-electron reductant); (5) compatible with a broad range of substrates; (6) applicable to a wide range of reactions; and (7) inexpensive and scalable using standard manufacturing vessels.

In this study, we describe the realization of this goal by heating inexpensive azo initiators commonly used in the polymer industry^20^ in the presence of formate salts to generate a strong one-electron reductant, carbon dioxide radical anion (CO_2_^**·**−^, ${{E}_{1/2}^{\circ }}=-{2.22}\,{\rm{V}}$ versus SCE)^20,21^. Inspired by advances in the photochemical generation of CO_2_^•−^ (refs. ^22–30^), we hypothesized that electrophilic α-cyano alkyl radicals (derived from the thermal decomposition of an azo initiator) would readily abstract hydrogen atoms from formate salts to from CO_2_^**·**−^ in a polarity-matched process (Fig. 1b). The proposed combination of an azo initiator and formate salt would provide an operationally simple ‘dump and stir’ thermal method of initiation, which would satisfy the aforementioned criteria and be widely applicable across synthetic chemistry.

## Results and discussion

### Initiator and model reaction development

**Fig. 2:**
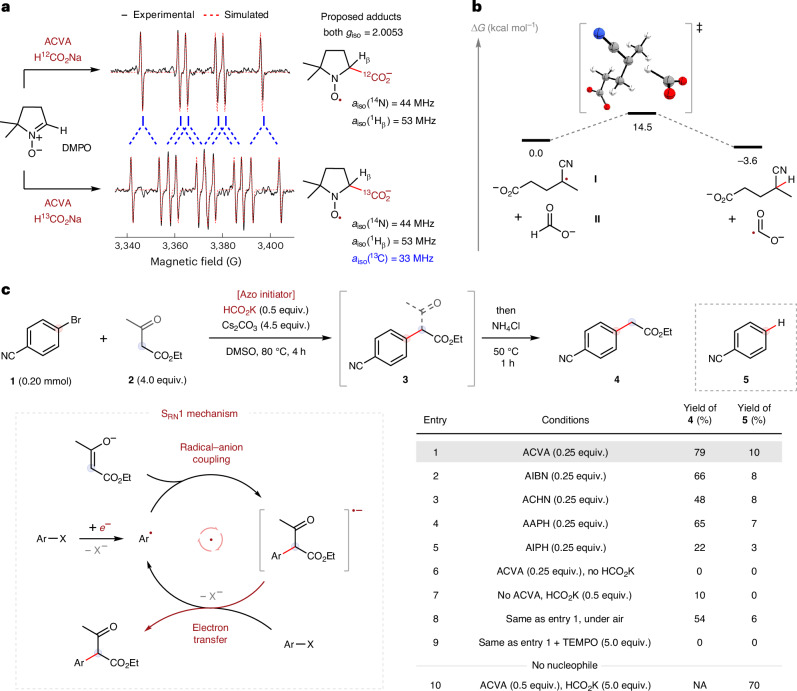
Hypothesis validation and reaction development. **a**, EPR spectroscopy to probe the formation of CO_2_^**·**−^. **b**, Computational study of HAT conducted at the M062X-D3(0) def2-TZVP SMD(DMSO) level of theory. **c**, Optimization of the proposed carbonyl α-arylation reaction. *g*_iso_, isotropic *g* value; *a*_iso_, isotropic *a* value (the hyperfine coupling constant); Ar, aryl; ACHN, 1,1’-azobis(cyclohexane-1-carbonitrile); AAPH, 2,2’-azobis(2-amidinopropane) dihydrochloride; AIPH, 2,2′-azobis[2-(2-imidazolin-2-yl)propane] dihydrochloride; NA, not applicable.

To evaluate the feasibility of our mechanistic proposal, we monitored the reaction of an azo initiator and sodium formate by electron paramagnetic resonance (EPR) spectroscopy in the presence of the spin trap, 5,5-dimethyl-1-pyrroline-*N*-oxide (DMPO). 4,4-Azobis(4-cyanovaleric acid) (ACVA) was selected as the azo initiator, as unlike azobisisobutyronitrile (AIBN), ACVA is not classified as an explosive. When reacting ACVA, HCO_2_Na and DMPO, a species matching previously reported data for a DMPO–CO_2_^**·**−^ adduct was detected (Fig. 2a)^31^. To verify if this species was indeed a CO_2_^**·**−^ adduct, these experiments were repeated using H^13^CO_2_Na and the expected additional ^13^C hyperfine coupling was clearly observed (*a*_iso_^13^C = 33 MHz). These results strongly suggest that CO_2_^**·**−^ is formed from the reaction of ACVA and sodium formate. The feasibility of polarity-matched hydrogen-atom transfer (HAT)^32^ between the ACVA-derived α-cyano alkyl radical **I** and formate **II** was assessed by density functional theory (DFT), which indicated that HAT is both kinetically and thermodynamically viable (Δ*G*^‡^ = 14.5 kcal mol^−1^, Δ*G* = −3.6 kcal mol^−1^; Fig. 2b). It is possible that the distonic nature of **I** and interactions between the α-cyano radical and carboxylate anion may play a role in the efficiency of this electron upconversion process (the conversion of a weak/mild reductant into a stronger reductant product)^17^, but any such interactions are probably weakened by the highly polar dimethylsulfoxide (DMSO) solvent environment^33–36^.

Having validated our key mechanistic hypothesis, model synthetic applications were explored to determine the generality of this initiation strategy. We first targeted C(*sp*^2^)–C(*sp*^3^) bond-forming carbonyl α-arylation reactions due to the synthetic utility and prevalence of the α-aryl carbonyl motif in biologically active compounds^37,38^. Moreover, we posited that a general transition-metal-free radical strategy for carbonyl α-arylation would: (1) complement the scope of existing methods^37,38^ by providing improved compatibility with heteroaromatic systems, which are common poisons for metal catalysts; and (2) avoid issues related to the cost and toxicity of transition metals, as well as the geopolitical, ethical and environmental concerns regarding their supply chains^39,40^. Here, we hypothesized that CO_2_^**·**−^ could initiate the electron-catalysed unimolecular radical-nucleophilic substitution (S_RN_1)^41,42^ of aryl halides with enolate nucleophiles^43,44^. Indeed, CO_2_^**·**−^ is known to readily reduce aryl halides to form aryl radicals^22,23,45^. Thus, aryl halide **1** was reacted with ethyl acetoacetate **2** (4.0 equiv.), Cs_2_CO_3_ (4.5 equiv.), ACVA (0.25 equiv.) and potassium formate (0.5 equiv.) in DMSO at 80 °C for 4 h (Fig. 2c). Pleasingly, following the addition of ammonium chloride (to promote complete deacetylation of the intermediate 1,3-dicarbonyl **3**), this one-pot procedure produced deacetylated α-aryl ester **4** in 79% yield and hydrodehalogenated side product **5** in 10% yield (Fig. 2c, entry 1). Hydrodehalogenated product **5** is proposed to form from a slower competing HAT chain in which the aryl radical abstracts a hydrogen atom from the formate salt (reforming CO_2_^**·**−^). Other commercially available azo initiators were trialled, but none proved superior to ACVA (entries 2–5). No product formation was observed in the absence of ACVA and HCO_2_K (entry 6). However, some product formation was observed with HCO_2_K alone (entry 7); we attribute this background reactivity to inefficient spontaneous initiation events—in which the anionic nucleophile itself serves as a weak one-electron reductant^45^—being amplified by CO_2_^**·**−^ formation. Repeating the optimal reaction with ACVA and HCO_2_K in the presence of air reduced the yield of product **4** to 54% (entry 8). Moreover, all reactivity was completely supressed in the presence of 2,2,6,6-tetramethylpiperidine-1-oxyl (TEMPO) (entry 9). Finally, repeating the reaction in the absence of the nucleophile **2** afforded only the hydrodehalogenated product **5** in 70% yield (entry 10). These findings are all consistent with an S_RN_1 electron-transfer chain process. Other compatible, but less effective formate salts and reaction conditions are described in Supplementary Tables 1–3.

### Electrophile scope and scale-up

**Fig. 3:**
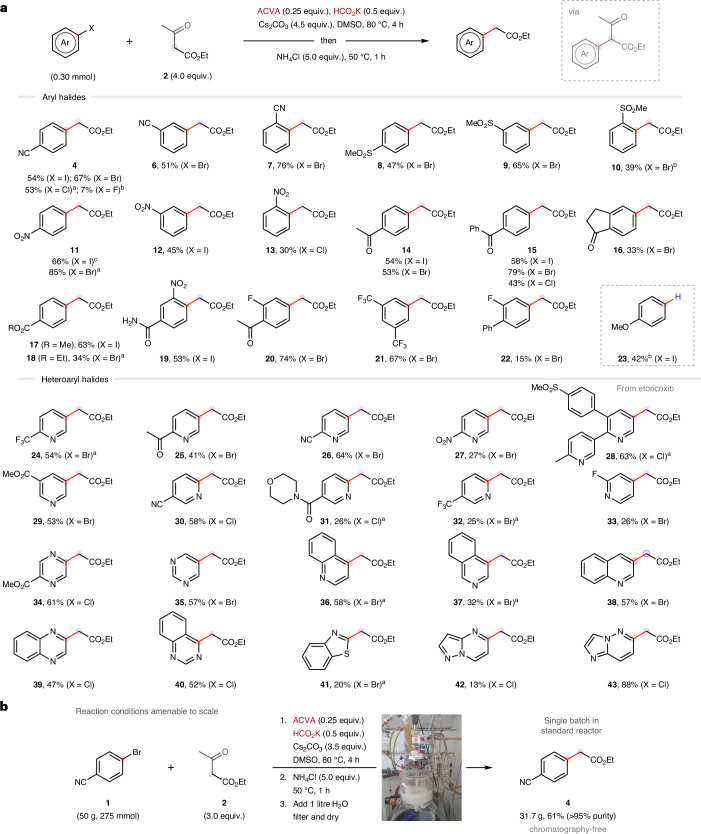
Electrophile scoping and scale-up studies. **a**, Scope of aryl and heteroaryl halides. **b**, Scale-up at AstraZeneca. ^a^See Supplementary Information, ‘Experimental Procedures and Characterisation Data’, for variations of the standard reaction conditions. ^b^Yields determined by ^1^H NMR spectroscopy against an internal standard (1,3,5-trimethoxybenzene). ^c^No ACVA or formate added.

With optimized conditions in hand, the scope and application of this thermally initiated transformation were explored (Fig. 3a). First, the importance of the nucleofuge was examined with *para*-halobenzonitrile derivatives. Pleasingly, the iodo, bromo and chloro derivatives were all converted into α-aryl ester **4** in 54%, 67% and 53% yield, respectively. Conversely, only 7% of **4** was formed from the corresponding fluoride, which indicates that the contribution of a polar S_N_Ar mechanism to these reactions is probably minimal. Moreover, efficient reactivity was observed regardless of the arene substitution pattern because the *meta*- and *ortho*-substituted products **6** and **7** were both formed in good yields. Other electron-deficient nitrobenzene and aryl sulfone derivatives were similarly compatible and converted into products **8**–**13** in 30–85% yield. Interestingly, the reaction of 4-iodonitrobenzene formed product **11** in 66% yield in the absence of ACVA and HCO_2_K. We attribute this reactivity to spontaneous initiation, which is commonly observed when reacting anionic nucleophiles with easily reduced substrates (strongly electron-deficient aryl iodides)^46,47^. Carbonyl and ester derivatives were also tolerated and converted into α-aryl esters **14**–**20** in good to excellent yields. Notably, ester **20** could be formed in 77% yield from a dihalogenated substrate, illustrating the selectivity of this method for heavier halogens. Reactivity was still observed in the absence of strong electron-withdrawing resonance effects: bistrifluoromethyl derivative **21** was prepared in 66% yield. However, less electron-deficient systems displayed lower levels of reactivity as demonstrated by the formation of **22** in 15% yield. Moreover, no α-aryl ester product was formed from electrophiles bearing strong electron-donating groups such as 4-iodoanisole, but hydrodehalogenated product **23** was formed in 42% yield. This important result indicates that while the ACVA–formate initiator system is capable of reducing challenging electron-rich systems, the model S_RN_1 electron-transfer chain was not viable (potentially due to inefficient radical-anion coupling). Attention then turned towards our original target of heteroaryl halides. Pleasingly, pyridine, pyrazine and pyrimidine azaarenes, including the drug etoricoxib, with varying substitution patterns were all tolerated, furnishing α-aryl esters **24**–**35** in 26–63% yield. Finally, a variety of fused bicyclic heteroaryl halides, including quinolines, isoquinolines, quinoxalines, quinazolines, benzothiazoles, pyrazolopyrimidines and imidazopyridazines, were also compatible and substituted to form products **36**–**43** in 13–88% yield.

Next, in collaboration with the process chemistry group at AstraZeneca, we sought to determine if the developed method and initiator system were sufficiently scalable to allow widespread use in the pharmaceutical and related industries. Therefore, the safety of the model reaction system was examined, in particular the use of ACVA, DMSO and base at elevated temperature. Differential scanning calorimetry and fall-hammer testing of ACVA, and high-rate Carius tube testing of the reaction mixture, all confirmed that the reaction can be safely performed at 80 °C. The developed reaction was further optimized via high-throughput experimentation^48^ and time-course analysis, before being performed on a 50-g scale, which provided product **4** in 61% isolated yield and >95% purity following a simple precipitation and filtration process (Fig. 3b). This result was particularly pleasing considering the heterogeneous nature of the reaction mixture (common to many reactions using inorganic bases in organic solvents) because a lack of reaction homogeneity can complicate scale-up^49^. In addition, the clean reaction profile and lack of side products was notable as azo initiators are known to also react as electrophiles with strong nucleophiles^50^. Thus, the low-cost ACVA–formate initiator system appears highly amenable to large-scale applications (current prices: ACVA, £1.57 g^−1^; HCO_2_K, £0.11 g^−1^ (Sigma Aldrich)).

### Microscale parallel screening

**Fig. 4:**
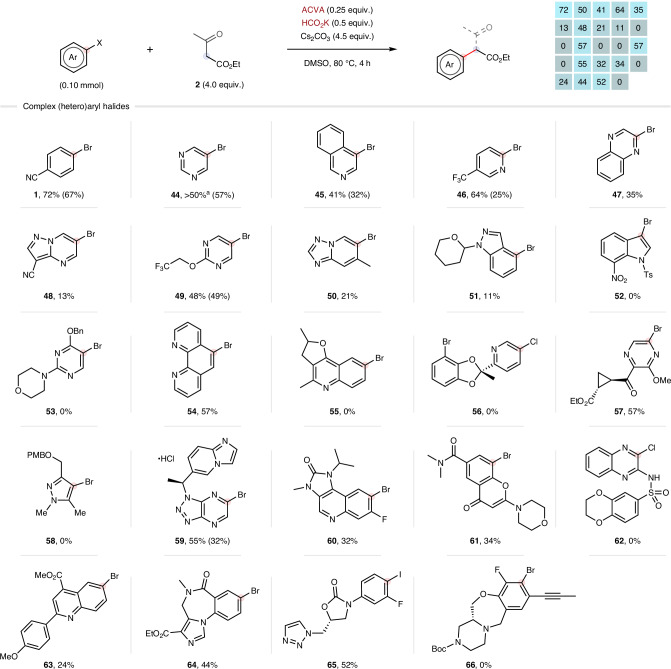
Microscale parallel screening of complex aryl halides. The deacetylation step with ammonium chloride was not performed to facilitate a workflow without additional solid handling. Yields shown are combined uHPLC/MS area/area% of acetylated and deacetylated products (if applicable) at 220 nm (identified by MS analysis). Screening yields are visually summarized in a heatmap-style graphic (higher yields are brighter in colour). Isolated yields at 0.3-mmol scale for validation are reported in parentheses. ^a^Accurate reporting of the reaction outcome was hampered by poor resolution of the reaction components across multiple analytical methods. Ts, *p*-toluenesulfonyl; Bn, benzyl; PMB, *p*-methoxybenzyl; Boc, *t*-butyloxycarbonyl.

Inspired by these promising findings, we sought to test the limits of the developed method and initiator system by exploring their compatibility with complex (hetero)aryl drug-like intermediates through microscale parallel screening at AstraZeneca (Fig. 4) (see Supplementary Information for more details). We had confidence in the ultrahigh-performance liquid chromatography (uHPLC)/mass spectrometry (MS)-based analysis because four substrates previously tested showed close agreement between isolated yields and uHPLC/MS area/area%. Pleasingly, 13 of the remaining 20 complex substrates, dense with Lewis basic-nitrogen functionality, afforded the desired products in synthetically useful yields under unoptimized conditions. To further validate these results, two of these ‘hits’ (**49** and **59**) were performed on a preparative scale and isolated in comparable yield. These results demonstrate the broad synthetic utility of the developed initiator system and α-arylation methodology, which both appear ready for immediate application in industrial drug discovery programmes.

### Nucleophile scope

**Fig. 5:**
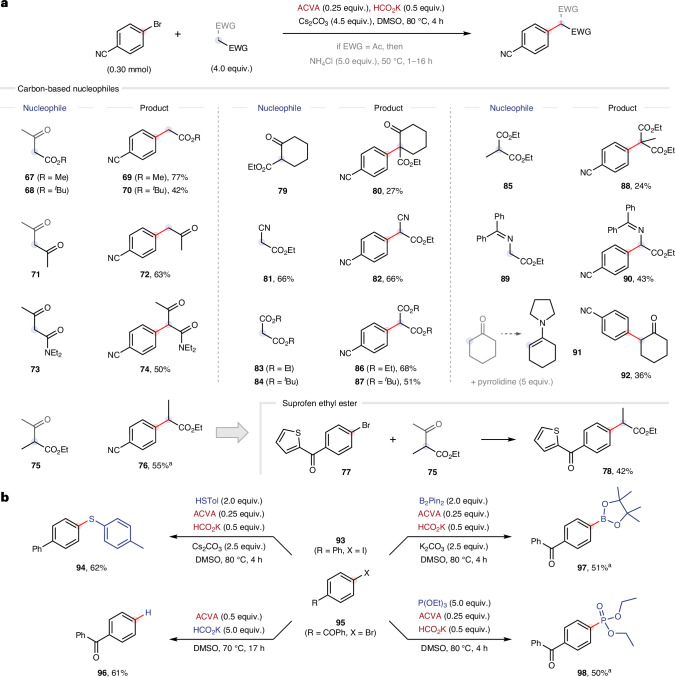
Nucleophile and radical trap scoping studies. **a**, Scope of carbon-based nucleophiles and synthesis of suprofen ethyl ester. **b**, Scope of other nucleophiles and radical traps. ^a^Yields determined by ^1^H NMR spectroscopy against an internal standard (1,3,5-trimethoxybenzene). EWG, electron-withdrawing group; Ac, acetyl; ^*t*^Bu, *tert*-butyl; Tol, *p*-tolyl; Pin, pinacol.

Having sufficiently explored the scope with respect to the electrophile, the nucleophile scope was investigated using **1** as a model substrate (Fig. 5a). First, closely related methyl and *tert*-butyl acetoacetates **67** and **68** were reacted to afford the corresponding α-arylated esters **69** and **70** in 77% and 42% yield, respectively. The ester functionality was not essential for reactivity because acetylacetone **71** was also converted into **72** (following a modified deacetylation protocol). Amide **73** was also tolerated and selectively converted into dicarbonyl **74** in 50% yield. More sterically congested nucleophiles such as **75** could be used to form α-methyl ester **76** in 55% yield. This result was particularly pleasing considering the prevalence of the α-methyl carboxylic acid moiety in non-steroidal anti-inflammatory drugs, as exemplified by the conversion of aryl bromide **77** into suprofen ethyl ester **78** in 42% yield. Cyclic ketone **79** was also successfully arylated to form **80** with a quaternary carbon centre in 27% yield. The ketone functionality was not essential for reactivity because ethyl cyanoacetate **81** was arylated to form **82** in 66% yield. In a similar fashion, malonates **83**–**85** were converted into **86**–**88** in 24–68% yield. Pleasingly, benzophenone glycine derivative **89** could also be used to form the corresponding N-protected unnatural α-aryl amino acid **90** in 43% yield. Finally, we examined non-anionic nucleophiles such as enamines using modified conditions based on the work of Gianetti and co-workers who arylated enamines under photoredox-catalysed conditions^51^. Here, the pyrrolidine-derived enamine of cyclohexanone **91** was formed in situ and arylated to afford **92** in 36% yield. This result supports the mechanistic hypothesis of Gianetti and co-workers who proposed that a chain mechanism was potentially operative alongside a photoredox catalytic cycle. In addition to applications in methodology development, the ACVA–formate initiator system may therefore be used as a general mechanistic tool to probe cases in which a radical chain mechanism is suspected.

To conclude our studies, we chose to demonstrate the utility of the developed ACVA–formate initiator system beyond C(*sp*^2^)–C(*sp*^3^) bond formation. First, simply by exchanging the nucleophile for a thiol, 4-iodobiphenyl **93** was converted into diaryl thioether **94** in 62% yield (Fig. 5b). This reaction presumably proceeds via an S_RN_1 mechanism as thiolates are known to be excellent S_RN_1 nucleophiles^42,52^. Next, by omitting any nucleophile and increasing the equivalents of ACVA and HCO_2_K, aryl bromide **95** was converted into hydrodehalogenated product **96** in 61% yield. The addition of bis(pinacolato)diboron (B_2_Pin_2_) enabled aryl boronic ester **97** to be formed in 51% yield. In a similar fashion, phosphonate ester **98** was formed in 50% yield when using triethyl phosphite as a radical trap. These results illustrate that the developed initiator system can be broadly applied for reaction development.

## Conclusion

We have developed a general, scalable and inexpensive method to initiate radical chains through one-electron reduction by reacting an azo initiator with a formate salt. The power of this initiation approach was demonstrated through the development of a general carbonyl α-(hetero)arylation protocol, which enabled challenging and valuable structural motifs to be formed under remarkably simple reaction conditions. The scalability of this initiation approach and carbonyl α-(hetero)arylation protocol was demonstrated on a 50-g scale in an industrial setting. Moreover, compatibility with a range of complex substrates was illustrated through microscale parallel screening. Finally, in addition to C(*sp*^2^)–C(*sp*^3^) bond formation, the wider synthetic potential of the developed initiation system was illustrated through the development of C(*sp*^2^)–S, C(*sp*^2^)–H, C(*sp*^2^)–B and C(*sp*^2^)–P coupling reactions. Considering the multidisciplinary importance of radical chain chemistry, we anticipate that this inexpensive initiation strategy will find widespread application in both industry and academia. We also speculate that alongside large-scale manufacturing opportunities, the disclosed initiator system may serve as a valuable mechanistic tool to probe the chain character of other radical reactions.

## Methods

### General procedure for carbonyl α-arylation

An 8-ml screw-cap vial was charged with ACVA (25.6 mg, 75 μmol, 0.25 equiv.), HCO_2_K (12.6 mg, 150 μmol, 0.50 equiv.), Cs_2_CO_3_ (440 mg, 1.35 mmol, 4.5 equiv.), and if solid, the aryl halide coupling partner (1.0 equiv.). To the solids was sequentially added a magnetic stirrer bar, anhydrous DMSO (1.5 ml), ethyl acetoacetate (156 mg, 1.20 mmol, 4.0 equiv.), and if liquid, the aryl halide coupling partner through the screw-cap septa. The reaction mixture was sparged with N_2_ for 15 min before being sealed with parafilm. The reaction mixture was then stirred at 80 °C in a metal heating block for 4 h. To promote complete deacetylation of intermediate 1,3-dicarbonyls, NH_4_Cl (80.3 mg, 1.50 mmol, 5.0 equiv.) was added. The reaction was then stirred at 50 °C in a metal heating block for 1 h. The reaction mixture was cooled to room temperature before being diluted with CH_2_Cl_2_ (10 ml) and water (10 ml). The organic phase was collected, and the aqueous phase was extracted with CH_2_Cl_2_ (3 × 10 ml). The organics were combined, washed with brine (25 ml), dried (MgSO_4_) and concentrated under reduced pressure. The crude product was then purified by column chromatography to afford the α-arylated product.

## Supplementary information

Supplementary Information Experimental details, Supplementary Figs. 1–12 and Tables 1–7.

## Data Availability

The authors declare that the data supporting the findings of this study are available within the paper and its Supplementary Information files.

## References

[1] Theriot, J. C. et al. Organocatalyzed atom transfer radical polymerization driven by visible light. *Science* **352**, 1082–1086 (2016). 27033549 10.1126/science.aaf3935

[2] Fazekas, T. J. et al. Diversification of aliphatic C–H bonds in small molecules and polyolefins through radical chain transfer. *Science* **375**, 545–550 (2022). 35113718 10.1126/science.abh4308 PMC8889563

[3] Constantin, T. et al. Halogen-atom and group transfer reactivity enabled by hydrogen tunneling. *Science* **377**, 1323–1328 (2022). 36108027 10.1126/science.abq8663

[4] Johansson, K. O., Head-Gordon, M. P., Schrader, P. E., Wilson, K. R. & Michelsen, H. A. Resonance-stabilized hydrocarbon-radical chain reactions may explain soot inception and growth. *Science* **361**, 997–1000 (2018). 30190399 10.1126/science.aat3417

[5] Freitas, F. P. et al. 7-Dehydrocholesterol is an endogenous suppressor of ferroptosis. *Nature* **626**, 401–410 (2024). 38297129 10.1038/s41586-023-06878-9

[6] Alabugin, I. V., Eckhardt, P., Christopher, K. M. & Opatz, T. The photoredox paradox: electron and hole upconversion as the hidden secrets of photoredox catalysis. *J. Am. Chem. Soc.* **146**, 27233–27254 (2024). 39316772 10.1021/jacs.4c10422

[7] Motherwell, W. B. & Crich, D. *Free Radical Chain Reactions in Organic Synthesis* (Elsevier, 1992).

[8] Studer, A. & Curran, D. P. Catalysis of radical reactions: a radical chemistry perspective. *Angew. Chem. Int. Ed.* **55**, 58–102 (2016). 10.1002/anie.201505090 26459814

[9] Shaw, M. H., Twilton, J. & MacMillan, D. W. C. Photoredox catalysis in organic chemistry. *J. Org. Chem.* **81**, 6898–6926 (2016). 27477076 10.1021/acs.joc.6b01449 PMC4994065

[10] Yan, M., Kawamata, Y. & Baran, P. S. Synthetic organic electrochemical methods since 2000: on the verge of a renaissance. *Chem. Rev.* **117**, 13230–13319 (2017). 28991454 10.1021/acs.chemrev.7b00397 PMC5786875

[11] Magenau, A. J. D., Strandwitz, N. C., Gennaro, A. & Matyjaszewski, K. Electrochemically mediated atom transfer radical polymerization. *Science* **332**, 81–84 (2011). 21454784 10.1126/science.1202357

[12] Fu, M.-C., Shang, R., Zhao, B., Wang, B. & Fu, Y. Photocatalytic decarboxylative alkylations mediated by triphenylphosphine and sodium iodide. *Science* **363**, 1429–1434 (2019). 30923218 10.1126/science.aav3200

[13] Constantin, T. et al. Aminoalkyl radicals as halogen-atom transfer agents for activation of alkyl and aryl halides. *Science* **367**, 1021–1026 (2020). 32108109 10.1126/science.aba2419

[14] Lovato, K., Fier, P. S. & Maloney, K. M. The application of modern reactions in large-scale synthesis. *Nat. Rev. Chem.* **5**, 546–563 (2021). 37117583 10.1038/s41570-021-00288-z

[15] Petrović, N., Malviya, B. K., Kappe, C. O. & Cantillo, D. Scaling-up electroorganic synthesis using a spinning electrode electrochemical reactor in batch and flow mode. *Org. Process Res. Dev.* **27**, 2072–2081 (2023).

[16] Studer, A. & Curran, D. P. The electron is a catalyst. *Nat. Chem.* **6**, 765–773 (2014). 25143210 10.1038/nchem.2031

[17] Syroeshkin, M. A. et al. Upconversion of reductants. *Angew. Chem. Int. Ed.* **58**, 5532–5550 (2019). 10.1002/anie.201807247 30063285

[18] Zhou, S. et al. Identifying the roles of amino acids, alcohols and 1,2-diamines as mediators in coupling of haloarenes to arenes. *J. Am. Chem. Soc.* **136**, 17818–17826 (2014). 25474411 10.1021/ja5101036

[19] Rohrbach, S., Shah, R. S., Tuttle, T. & Murphy, J. A. Neutral organic super electron donors made catalytic. *Angew. Chem. Int. Ed.* **58**, 11454–11458 (2019). 10.1002/anie.201905814 31222953

[20] Odian, G. *Principles of Polymerization* (Wiley, 2004).

[21] Koppenol, W. H. & Rush, J. D. Reduction potential of the carbon dioxide/carbon dioxide radical anion: a comparison with other C1 radicals. *J. Phys. Chem.* **91**, 4429–4430 (1987).

[22] Hendy, C. M., Smith, G. C., Xu, Z., Lian, T. & Jui, N. T. Radical chain reduction via carbon dioxide radical anion (CO_2_^**·**–^). *J. Am. Chem. Soc.* **143**, 8987–8992 (2021). 34102836 10.1021/jacs.1c04427 PMC8925913

[23] Chmiel, A. F., Williams, O. P., Chernowsky, C. P., Yeung, C. S. & Wickens, Z. K. Non-innocent radical ion intermediates in photoredox catalysis: parallel reduction modes enable coupling of diverse aryl chlorides. *J. Am. Chem. Soc.* **143**, 10882–10889 (2021). 34255971 10.1021/jacs.1c05988

[24] Alektiar, S. N. & Wickens, Z. K. Photoinduced hydrocarboxylation via thiol-catalyzed delivery of formate across activated alkenes. *J. Am. Chem. Soc.* **143**, 13022–13028 (2021). 34380308 10.1021/jacs.1c07562

[25] Song, L. et al. Visible-light photocatalytic di- and hydro-carboxylation of unactivated alkenes with CO_2_. *Nat. Catal.* **5**, 832–838 (2022).

[26] Alektiar, S. N., Han, J., Dang, Y., Rubel, C. Z. & Wickens, Z. K. Radical hydrocarboxylation of unactivated alkenes via photocatalytic formate activation. *J. Am. Chem. Soc.* **145**, 10991–10997 (2023). 37186951 10.1021/jacs.3c03671 PMC10636750

[27] Williams, O. P. et al. Practical and general alcohol deoxygenation protocol. *Angew. Chem. Int. Ed.* **62**, e202300178 (2023). 10.1002/anie.202300178 PMC10121858 36840940

[28] Dang, Y. et al. Alkene carboxy-alkylation via CO_2_^**·**–^. *J. Am. Chem. Soc.* **146**, 35035–35042 (2024). 39665217 10.1021/jacs.4c14421 PMC12062844

[29] Yu, B. et al. Switchable divergent di- or tricarboxylation of allylic alcohols with CO_2_. *Chem* **10**, 938–951 (2024).

[30] Ghosh, P. et al. Taming CO_2_^**·**–^ via synergistic triple catalysis in anti-markovnikov hydrocarboxylation of alkenes. *J. Am. Chem. Soc.* **146**, 30615–30625 (2024). 39468468 10.1021/jacs.4c12294

[31] Villamena, F. A., Locigno, E. J., Rockenbauer, A., Hadad, C. M. & Zweier, J. L. Theoretical and experimental studies of the spin trapping of inorganic radicals by 5,5-dimethyl-1-pyrroline *N* -oxide (DMPO). 2. Carbonate radical anion. *J. Phys. Chem. A* **111**, 384–391 (2007). 17214476 10.1021/jp065692d

[32] Ruffoni, A., Mykura, R. C., Bietti, M. & Leonori, D. The interplay of polar effects in controlling the selectivity of radical reactions. *Nat. Synth.* **1**, 682–695 (2022).

[33] Gryn’ova, G., Marshall, D. L., Blanksby, S. J. & Coote, M. L. Switching radical stability by pH-induced orbital conversion. *Nat. Chem.* **5**, 474–481 (2013). 23695628 10.1038/nchem.1625

[34] Zhao, R., Fu, K., Fang, Y., Zhou, J. & Shi, L. Site-specific C(*sp*^3^)–H Aminations of imidates and amidines enabled by covalently tethered distonic radical anions. *Angew. Chem. Int. Ed.* **59**, 20682–20690 (2020). 10.1002/anie.202008806 32706927

[35] Mondal, T., Shaik, S., Kenttämaa, H. & Stuyver, T. Modulating the radical reactivity of phenyl radicals with the help of distonic charges: it is all about electrostatic catalysis. *Chem. Sci.* **12**, 4800–4809 (2021). 34163733 10.1039/d0sc07111k PMC8179573

[36] Gryn’ova, G. & Coote, M. L. Origin and scope of long-range stabilizing interactions and associated SOMO–HOMO conversion in distonic radical anions. *J. Am. Chem. Soc.* **135**, 15392–15403 (2013). 24090128 10.1021/ja404279f

[37] Lloyd-Jones, G. C. Palladium-catalyzed α-arylation of esters: ideal new methodology for discovery chemistry. *Angew. Chem. Int. Ed.* **41**, 953–956 (2002). 10.1002/1521-3773(20020315)41:6<953::aid-anie953>3.0.co;2-9 12491279

[38] Johansson, C. C. C. & Colacot, T. J. Metal-catalyzed α-arylation of carbonyl and related molecules: novel trends in C–C bond formation by C–H bond functionalization. *Angew. Chem. Int. Ed.* **49**, 676–707 (2010). 10.1002/anie.200903424 20058282

[39] Economidou, M., Mistry, N., Wheelhouse, K. M. P. & Lindsay, D. M. Palladium extraction following metal-catalyzed reactions: recent advances and applications in the pharmaceutical industry. *Org. Process Res. Dev.* **27**, 1585–1615 (2023).

[40] Sun, C.-L. & Shi, Z.-J. Transition-metal-free coupling reactions. *Chem. Rev.* **114**, 9219–9280 (2014). 25184859 10.1021/cr400274j

[41] Bunnett, J. F. Aromatic substitution by the S_RN_1 mechanism. *Acc. Chem. Res.* **11**, 413–420 (1978).

[42] Rossi, R. A., Pierini, A. B. & Peñéñory, A. B. Nucleophilic substitution reactions by electron transfer. *Chem. Rev.* **103**, 71–168 (2003). 12517182 10.1021/cr960134o

[43] Rossi, R. A. & Bunnett, J. F. Photostimulated aromatic S_RN_1 reactions. *J. Org. Chem.* **38**, 1407–1410 (1973).

[44] Beugelmans, R., Bois-Choussy, M. & Boudet, B. Studies on S_RN_1 reactions. Part 8: new and direct arylation and hetarylation of β-dicarbonyl compounds by S_RN_1. *Tetrahedron* **38**, 3479–3483 (1982).

[45] Beeler, J. A., Walkingshaw, R. P., Hamud, S. A. S. & White, H. S. Reduction by oxidation: selective hydrodehalogenation of aryl halides by mediated oxalate oxidation. *J. Am. Chem. Soc.* **147**, 12206–12217 (2025). 40162707 10.1021/jacs.5c01366

[46] Costentin, C., Hapiot, P., Médebielle, M. & Savéant, J.-M. “Thermal” S_RN_1 reactions: how do they work? Novel evidence that the driving force controls the transition between stepwise and concerted mechanisms in dissociative electron transfers. *J. Am. Chem. Soc.* **121**, 4451–4460 (1999).

[47] Costentin, C., Hapiot, P., Médebielle, M. & Savéant, J.-M. Investigation of dissociative electron transfer mechanisms and reactivity patterns through kinetic amplification by a chain process. *J. Am. Chem. Soc.* **122**, 5623–5635 (2000).

[48] Douglas, J. J. et al. The implementation and impact of chemical high-throughput experimentation at AstraZeneca. *ACS Catal.* **15**, 5229–5256 (2025).

[49] Meyers, C. et al. Study of a new rate increasing “base effect” in the palladium-catalyzed amination of aryl iodides. *J. Org. Chem.* **69**, 6010–6017 (2004). 15373485 10.1021/jo049774e

[50] Elliott, Q. & Alabugin, I. V. AIBN as an electrophilic reagent for cyano group transfer. *J. Org. Chem.* **88**, 2648–2654 (2023). 36752409 10.1021/acs.joc.2c02859

[51] Hossain, M. M., Shaikh, A. C., Moutet, J. & Gianetti, T. L. Photocatalytic α-arylation of cyclic ketones. *Nat. Synth.* **1**, 147–157 (2022).

[52] Bunnett, J. F. & Creary, X. Arylation of arenethiolate ions by the S_RN_1 mechanism. Convenient synthesis of diaryl sulfides. *J. Org. Chem.* **39**, 3173–3174 (1974).

