# Supplementary material for: Reductive radical chain initiation through the thermal generation of carbon dioxide radical anion
Source: Nat Synth. 2025 Nov 6;5(2):221–9. doi: 10.1038/s44160-025-00919-z (PMC12885953; doi:10.1038/s44160-025-00919-z)

nature synthesis

Supplementary information https://doi.org/10.1038/s44160-025-00919-z

# Reductive radical chain initiation through the thermal generation of carbon dioxide radical anion

In the format provided by the authors and unedited

# Table of Contents


**1. General information** .......... **2**

**2. EPR Spectroscopy** .......... **3**

General Procedure for EPR Studies .......... 3

DMPO Control Experiment .......... 4

Fragmentation of ACVA .......... 5

Evidence for the Formation of the $CO_2^{\bullet-}$ .......... 6

**3. Computational studies** .......... **9**

Radical–Anion Coupling and Electron Transfer .......... 10

Geometries and Thermochemistry Data .......... 11

**4. Carbonyl α-(Hetero)Arylation Optimisation Studies** .......... **25**

Comparison to Other Methods of Initiation .......... 29

**5. Experimental Procedures and Characterisation Data** .......... **31**

**6. Scale Up of the Synthesis of Ethyl 2-(4-cyanophenyl)acetate** .......... **91**

Optimisation of Standard Procedure Prior to 50 g Scale Reaction .......... 91

Process Safety Assessment Prior to 50 g Scale Reaction .......... 96

Safety Assessment of the Standard Procedure .......... 101

50 g Scale Procedure .......... 106

**7. Data for Fig. 4. Microscale Parallel Screening** .......... **109**

List of Substrates .......... 109

General Procedure .......... 110

Reaction Outcomes .......... 111

uHPLC/MS data .......... 113

Ethyl 2-(2-(2,2,2-trifluoroethoxy)pyrimidin-5-yl)acetate (S3) .......... 137

Preparative Scale Synthesis of ethyl 2-[3-[(1S)-1-imidazo[1,2-a]pyridin-6-ylethyl]triazolo[4,5-b]pyrazin-5-yl]acetate (S4) .......... 138

**8. References** .......... **140**

**9. NMR Spectral Data** .......... **141**

# 1. General information

Except where stated, all reagents and anhydrous solvents were purchased from commercial sources and used without further purification.

NMR spectra were recorded on a Bruker AVIII300NB, JEOL ECX400, JEOL ECS400, or Bruker AVIIIHD500 spectrometer. All spectral data was acquired at the stated temperature. Chemical shifts (δ) are quoted in parts per million (ppm). The following residual solvent signals were used as references for $^{1}H$ and $^{13}C$ NMR spectra: $\delta_H$ 7.26 ppm and $\delta_C$ 77.16 ppm for $CDCl_3$, $\delta_H$ 2.50 ppm, $\delta_C$ 39.52 ppm for DMSO-$d_6$ and $\delta_H$ 3.31 ppm, $\delta_C$ 49.00 ppm for $CD_3OD$. Coupling constants (*J*) are reported in Hertz (Hz) to the nearest 0.1 Hz. The multiplicity abbreviations used are: br, broad; s, singlet; d, doublet; t, triplet; q, quartet; m, multiplet. All $^{13}C$ NMR spectra were acquired with proton decoupling. All $^{19}F$ and $^{31}P$ NMR spectra were acquired without proton decoupling.

High-resolution mass-spectra were obtained by the University of Manchester Mass Spectrometry Service, using electrospray ionisation (ESI) or atmospheric-pressure chemical ionisation (APCI) on a Bruker Daltonics, Micro-tof spectrometer.

Thin layer chromatography was carried out on Merck silica gel 60F254 pre-coated aluminium foil sheets and were visualised using UV light (254 nm) and stained with basic aqueous potassium permanganate. Column chromatography was carried out using Fluka silica gel ($SiO_2$), 35–70 μm, 60 Å under a light positive pressure, eluting with the specified solvent system. Melting points were determined using a Cole-Parmer MP-100 Stuart analogue melting point apparatus.

4,4′-Azobis(4-cyanopentanoic acid) (ACVA) containing 13-18% $H_2O$, 2,2'-Azobis(2-amidinopropane) dihydrochloride (AAPH) and 2,2′-azobis[2-(2-imidazolin-2-yl)propane]dihydrochloride (AIPH) were purchased from Fluorochem. 2,2′-Azobis(2-methylpropionitrile) (AIBN) and 1,1′-azobis(cyclohexanecarbonitrile) (ACHN) were purchased from Sigma Aldrich.

Note: the mass of ACVA was calculated on the assumption that ACVA contained 18% $H_2O$. Near identical results were obtained with anhydrous ACVA.

## 2. EPR Spectroscopy

To investigate the formation of $CO_2^{\bullet-}$ from the reaction of ACVA and formate salts, radical trapping experiments using DMPO (5,5-dimethyl-1-pyrroline *N*-oxide) and Electron Paramagnetic Resonance (EPR) spectroscopy were conducted. In such experiments, the spectrum of the DMPO-radical adduct shows hyperfine coupling to the nitroxyl $^{14}N$ (nuclear spin $I = 1$) and β-$^{1}H$ ($I = ½$) nuclei, where the coupling constants depend on the nature of the trapped radical (**Fig. S1**).[53]

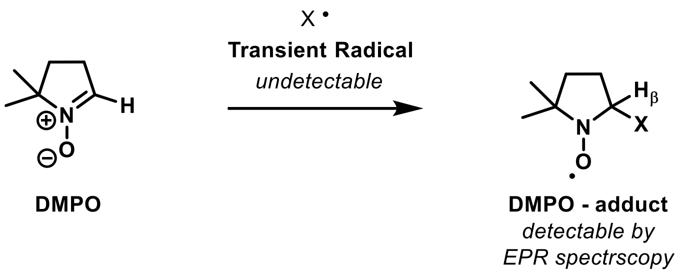


**Fig. S1**. Detection of transient radicals with DMPO.

### General Procedure for EPR Studies

Unless otherwise stated, all EPR samples were prepared under aerobic conditions by combining µL volumes of stock solutions of the respective reagents in $H_2O$ or DMSO to an Eppendorf tube before being transferred into a 3 mm outer diameter/1 mm inner diameter quartz tube for EPR analysis (sample volume ~ 30 mm in height). Variable Temperature EPR spectra were measured on a Bruker Magnettech ESR 5000 Spectrometer equipped with a TCH04 variable temperature unit. The sample position inside the active-resonator was achieved using a special holder that comes with the Magnettech ESR 5000 spectrometer. The optimum spectrometer conditions were: microwave power 1 mW, modulation amplitude 0.2 G, sweep time 30 s. The average microwave frequency was 9.467 GHz (X-band). All EPR spectra were measured as a fluid solution. Magnetic fields were calibrated against a strong pitch standard. Analysis of the spectra and simulations were performed using the EasySpin toolbox (5.2.35) for the Matlab program package[54] using the "garlic" function for isotropic spectra under fast motion. The extracted spin-Hamiltonian parameters are consistent with previously reported values for DMPO-R adducts.[53]

## DMPO Control Experiment

Trace impurities contained within the DMPO sample are shown to illustrate that the measurements and characterisation of the various radical adducts to follow do not correspond to these impurities as the splitting patterns do not match; and the intensity of the signals exceed that of the impurities (**Fig. S2**).

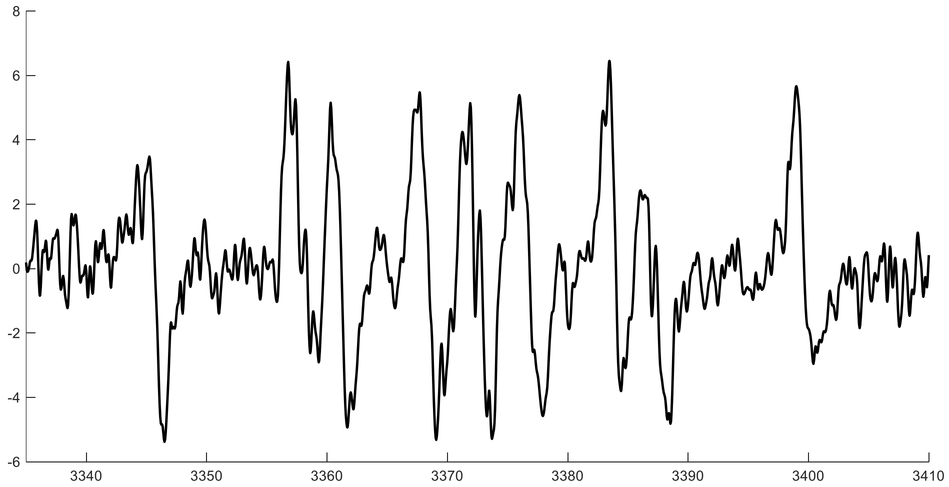


**Fig. S2.** EPR spectrum of DMPO (500 mM) in $H_2O$ at 30 °C to illustrate the presence of trace impurities in the DMPO trapping agent.

## Fragmentation of ACVA

When ACVA was heated in the presence of the DMPO radical trap, a C-centred adduct was observed at 60 °C (**Fig. S3**). Note: $Cs_2CO_3$ was added as a base to increase the solubility of ACVA in $H_2O$. While more evidence would be required to exactly describe this adduct, it is tentatively ascribed to form via the trapping of carbon-centred radical **III**, after the fragmentation of ACVA (**Fig. S4**).

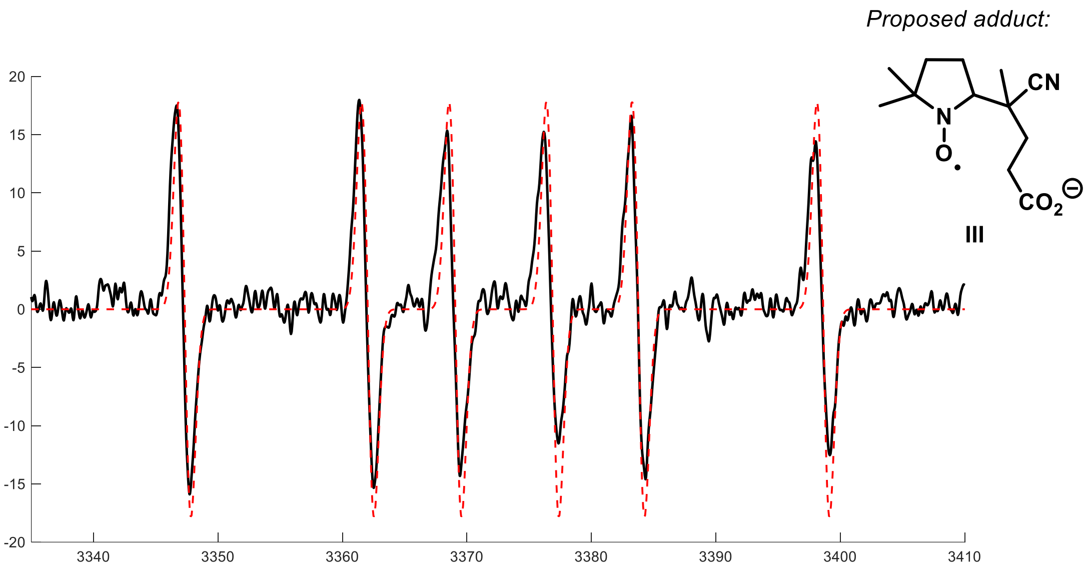


**Fig. S3.** Experimental and simulated EPR spectra of the solution containing, ACVA (250 mM), $Cs_2CO_3$ (250 mM) and DMPO (500 mM) in $H_2O$ at 60 °C (simulation parameters: $g_{iso}$ = 2.0054, $a_{iso}(^{14}N)$ = 41.5 MHz, $a_{iso}(^{1}H)$ = 61 MHz, line-width = 1 G)

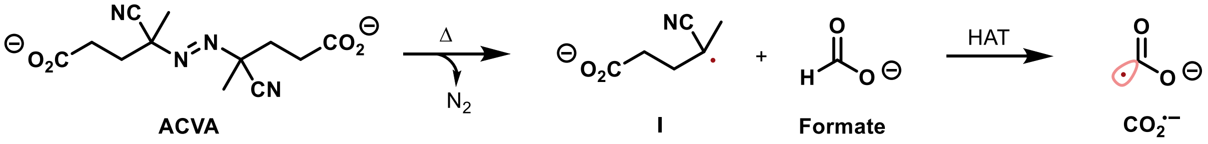


**Fig. S4**. Proposed mechanism for the generation of the $CO_2^{\bullet-}$ from ACVA and Formate.

## Evidence for the Formation of the $CO_2^{\bullet-}$

After confirming the formation of radical intermediates from ACVA under thermal conditions, sodium formate was added to investigate the formation of the $CO_2^{\bullet-}$ as proposed in **Fig. S4**. When a solution containing ACVA (250 mM), $Cs_2CO_3$ (250 mM), sodium formate (1 M) and DMPO (500 mM) in $H_2O$ was heated to 60 °C, a new six-line pattern was observed with the hyperfine coupling different to that of the solution containing ACVA (250 mM), $Cs_2CO_3$ (250 mM) and DMPO (500 mM). **(Fig. S5)**. This DMPO-R adduct was assigned to the formation of $CO_2^{\bullet-}$. To confirm this, $^{13}C$-labelled sodium formate was employed (**Fig. S6**). The hyperfine splitting pattern previously observed was now split by the spin active $^{13}C$ nucleus ($I(^{13}C) = ½$) strongly suggesting that the trapped adduct was indeed the $CO_2^{\bullet-}$ derived from sodium formate (**Fig. S7**).

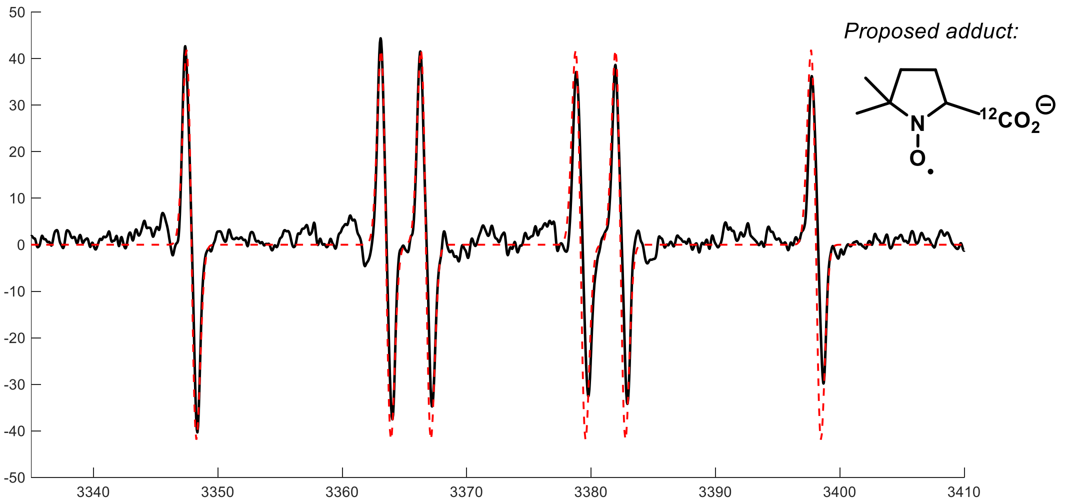


**Fig. S5.** Experimental and simulated EPR spectra of the solution containing, ACVA (250 mM), $Cs_2CO_3$ (250 mM), sodium formate (1 M) and DMPO (500 mM) in $H_2O$ at 60 °C (simulation parameters: $g_{iso} = 2.0054$, $a_{iso}(^{14}N) = 44$ MHz, $a_{iso}(^{1}H) = 53$ MHz, line-width = 0.8 G)

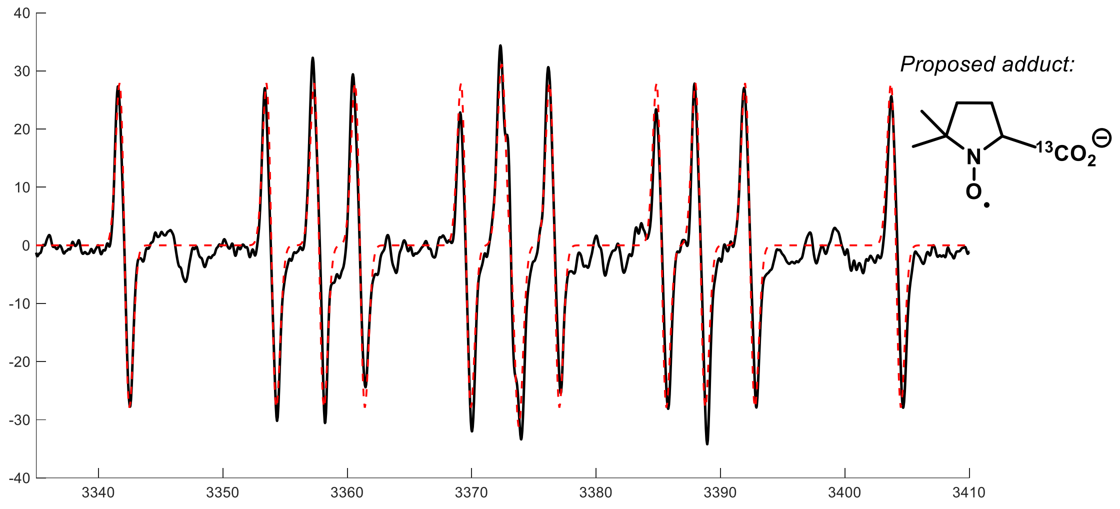


**Fig. S6.** Experimental and simulated EPR spectra of the solution containing, ACVA (250 mM), $Cs_2CO_3$ (250 mM), $^{13}C$ labelled sodium formate (1 M) and DMPO (500 mM) in $H_2O$ at 50 °C(simulation parameters: $g_{iso}$ = 2.0054, $a_{iso}(^{14}N)$ = 44 MHz, $a_{iso}(^{1}H)$ = 53 MHz, $a_{iso}(^{13}C)$ = 33 MHz, linewidth = 0.8 G)

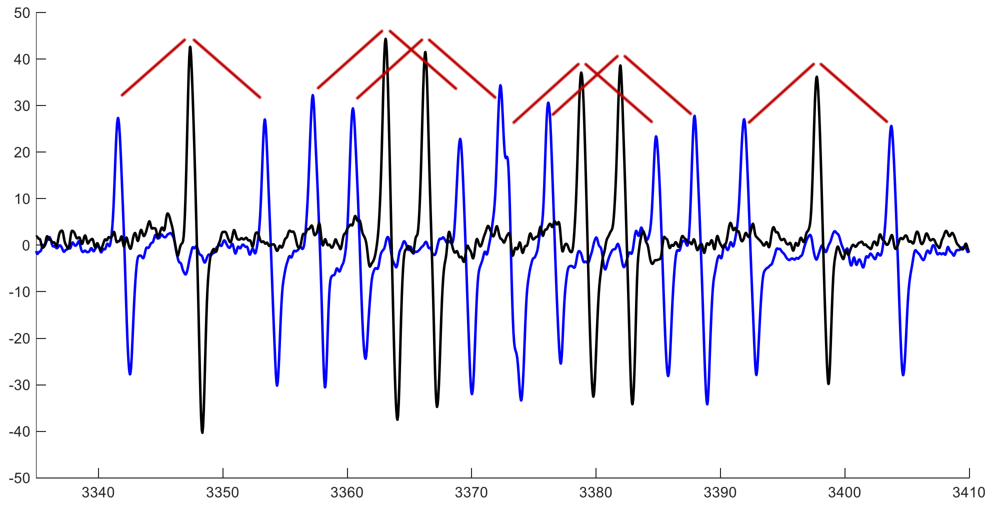

**Fig. S7.** Comparisons of the experimental EPR spectra of the solution containing, ACVA (250 mM), $Cs_2CO_3$ (250 mM), natural abundance (black trace) and $^{13}C$ labelled (blue trace) sodium formate (1 M) and DMPO (500 mM) in $H_2O$ at 60 °C and 50 °C, respectively. The red "^" symbols indicate the additional splitting observed when $^{13}C$-labelled formate was used ($a_{iso}(^{13}C)$ = 33 MHz).

Finally, to investigate the formation of the $CO_2^{\bullet-}$ in DMSO, a final solution containing 70% DMSO and 30% $H_2O$ was analysed ($H_2O$ was required to dissolve the formate to the desired final concentration). The same characteristic hyperfine splitting pattern for the proposed DMPO-$CO_2^{\bullet-}$ adduct was observed (**Fig. S8**).

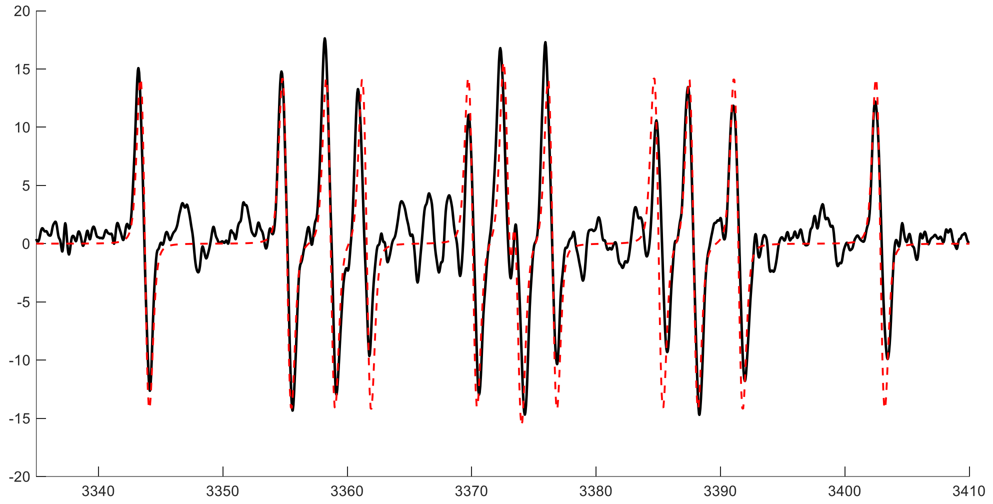


**Fig. S8.** Experimental and simulated EPR spectra of the solution containing, ACVA (250 mM), $^{13}$C-labelled sodium formate (1 M) and DMPO (500 mM) in DMSO:$H_2O$ (7:3) at 55 °C (simulation parameters: $g_{iso}$ = 2.0052, $a_{iso}(^{14}N)$ = 42 MHz, $a_{iso}(^{1}H)$ = 50 MHz, $a_{iso}(^{13}C)$ = 30 MHz, linewidth = 0.8 G (Gaussian) 0.2 G (Lorentzian)

## 3. Computational studies

The authors would like to acknowledge the assistance given by Research IT and the use of the Computational Shared Facility at The University of Manchester. All DFT calculations were carried out using ORCA 6.0.1.[55] All closed-shell species were described with restricted Kohn-Sham theory. Open-shell species were described with the unrestricted formalism. Unless stated otherwise, geometries were optimised using the hybrid meta-GGA functional M062X-D3(0),[56] the def2-TZVP basis set,[57] and the SMD solvation model (DMSO)[58]. The DEFGRID3 keyword was also added to the input. The optimised structures are given below in .xyz format and visualized using ChemCraft.[59] Vibrational frequency calculations were performed at the same level to confirm if an obtained geometry was either a local minimum or a transition state (TS) and to calculate thermochemistry values (at the stated temperature). Free energies were derived from this data and a correction was added to convert gas phase thermochemistry values into the liquid phase [RTln(24.5)].[60]

## Radical–Anion Coupling and Electron Transfer

Calculations for the coupling of anion **III** and aryl radical **IV** suggest that this process would be fast and energetically favourable ($\Delta G^{\ddagger} = 7.3$ kcal mol$^{-1}$, $\Delta G = -24.5$ kcal mol$^{-1}$ for the free anion; and $\Delta G^{\ddagger} = 5.4$ kcal mol$^{-1}$, $\Delta G = -20.7$ kcal mol$^{-1}$ with a caesium countercation). Using a method based on the work of Nicewicz and co-workers,[61] the redox potential of **V** was calculated to be $E_{1/2}^{0} = -2.14$ V vs SCE. The reported redox potential of aryl halide **1** is $E_{1/2}^{0} = -1.95$ V vs SCE.[62] Propagation of the $S_{RN}1$ chain by electron transfer from **V** to aryl halide **1** (to reform aryl radical **IV**) should therefore be facile. It should be noted that spin density of radical anion **V** is primarily located on the aromatic π-system and so the redox potentials of such products will change according to the electronics of the substrate.

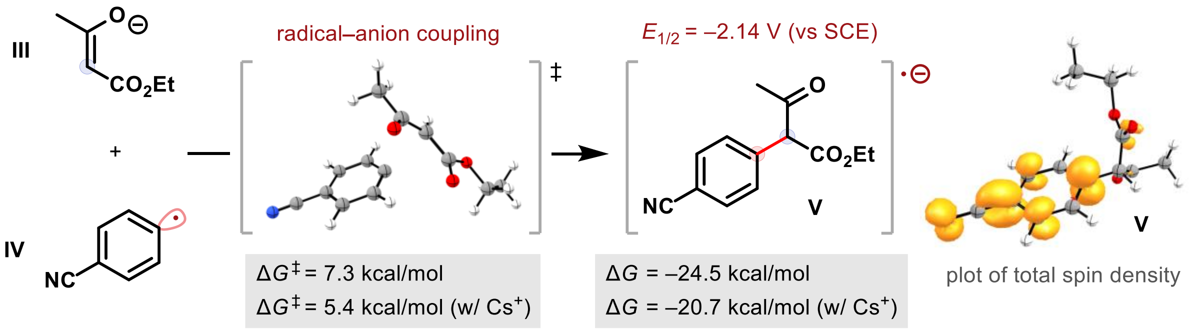


To calculate the redox potential of **V**, the geometries of the **3**/**V** redox couple were optimised at the M062X-D3(0) def2-TZVP level using the CPCM continuum solvation model (Acetonitrile). The obtained geometries were confirmed to be local minima by frequency analysis at the same level. The calculated Gibbs free energies at 298 K were then used to estimate the redox potential of **V** and referenced to saturated calomel electrode (SCE) by subtraction of its absolute potential in acetonitrile ($E_{ref} = 4.422$ V).[63]

$G_{298}$(radical anion) = −783.53296826 Eh

$G_{298}$(neutral) = −783.44925192 Eh

$\Delta G^{\circ}_{1/2}$ = ($G_{298}$[radical-anion] – $G_{298}$[neutral]) = [−783.53296826−(−783.44925192)] Eh × 627.5 kcal mol$^{-1}$ Eh$^{-1}$ = −52.5320034 kcal mol$^{-1}$

$$E^{\circ\text{calc}}_{1/2} = -\frac{\Delta G^{\circ}_{1/2}}{n_e F} - E^{\circ\text{SHE}}_{1/2} + E^{\circ\text{SCE}}_{1/2} = -\frac{-52.53}{23.061} - 4.281 - 0.141 = -2.14 \text{ V vs SCE}$$

## Geometries and Thermochemistry Data

## ACVA Derived α-Cyanoalkyl Radical

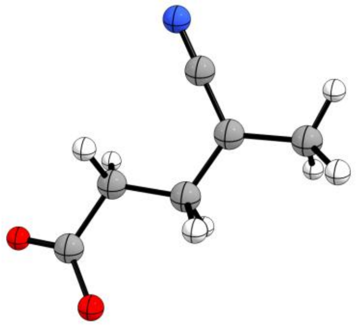

Geometry and vibrational frequencies: M062X-D3(0) def2-TZVP SMD(DMSO)

Charge: −1; Multiplicity: 2; # of imaginary frequencies: 0

G(298 K, 1 atm) = −438.06359197 Eh

G(298 K, 1 M) = G(298 K, 1 atm) + RTln(24.5) = −438.060573850367 Eh

| | | | |
|---|---|---|---|
| O | -3.469906000 | -3.730323000 | 0.164854000 |
| O | -5.655799000 | -3.452056000 | -0.242747000 |
| N | -3.057219000 | 1.824685000 | -0.460677000 |
| C | -4.492873000 | -3.042453000 | -0.040936000 |
| C | -4.266744000 | -1.514041000 | -0.066775000 |
| C | -5.544014000 | -0.694223000 | -0.051822000 |
| C | -4.104631000 | 1.348123000 | -0.307121000 |
| C | -6.554293000 | 1.676217000 | 0.009104000 |
| C | -5.366220000 | 0.786604000 | -0.118375000 |
| H | -3.687567000 | -1.291375000 | -0.968720000 |
| H | -3.628191000 | -1.249787000 | 0.779342000 |
| H | -6.129266000 | -0.918864000 | 0.847171000 |
| H | -6.188050000 | -0.997328000 | -0.884898000 |
| H | -6.303995000 | 2.726814000 | -0.121978000 |
| H | -7.309290000 | 1.395306000 | -0.731177000 |
| H | -7.012961000 | 1.544040000 | 0.994156000 |

**Formate Anion**

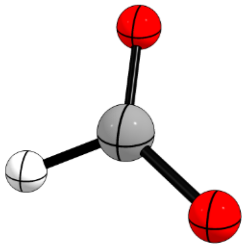

Geometry and vibrational frequencies: M062X-D3(0) def2-TZVP SMD(DMSO)

Charge: −1; Multiplicity: 1; # of imaginary frequencies: 0

G(298 K, 1 atm) = −189.30436041 Eh

G(298 K, 1 M) = G(298 K, 1 atm) + RTln(24.5) = − 189.301342290367 Eh

| | | | |
|---|---|---|---|
| C | -1.373665000 | 3.493577000 | 0.000000000 |
| O | -1.569624000 | 4.723287000 | 0.000000000 |
| O | -0.299157000 | 2.864385000 | 0.000000000 |
| H | -2.303124000 | 2.858341000 | 0.000000000 |

**Hydrogen Atom Transfer Transition State**

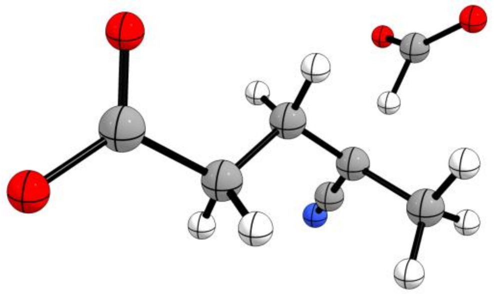

Geometry and vibrational frequencies: M062X-D3(0) def2-TZVP SMD(DMSO)

Charge: −2; Multiplicity: 2; # of imaginary frequencies: 1 (−1027.77 $cm^{-1}$)

G(298 K, 1 atm) = −627.34174919 Eh

G(298 K, 1 M) = G(298 K, 1 atm) + RTln(24.5) = −627.338731070367 Eh

| | | | |
|---|---|---|---|
| C | -2.686014000 | 4.248221000 | -0.427826000 |
| C | -3.211979000 | 3.095244000 | 0.395120000 |
| H | -2.643498000 | 2.182706000 | 0.209097000 |
| H | -4.264933000 | 2.884146000 | 0.186403000 |
| H | -3.122005000 | 3.342771000 | 1.454722000 |
| C | -2.494318000 | 3.982810000 | -1.804626000 |
| N | -2.272150000 | 3.775976000 | -2.923147000 |
| C | -3.205736000 | 5.634574000 | -0.097130000 |
| C | -4.692839000 | 5.818000000 | -0.374826000 |
| H | -2.632963000 | 6.379702000 | -0.654625000 |
| H | -3.007863000 | 5.820363000 | 0.961658000 |
| C | -5.259026000 | 7.210373000 | -0.015202000 |
| H | -5.273891000 | 5.082383000 | 0.190509000 |
| H | -4.909232000 | 5.628796000 | -1.430224000 |
| O | -4.523664000 | 7.978992000 | 0.640248000 |
| O | -6.425528000 | 7.431974000 | -0.409800000 |
| C | -0.187134000 | 4.614655000 | 0.685816000 |
| O | 0.606736000 | 5.196178000 | -0.049105000 |
| O | -0.170070000 | 4.233378000 | 1.853401000 |
| H | -1.312782000 | 4.360062000 | 0.084737000 |

**Hydrogen Atom Transfer Product**

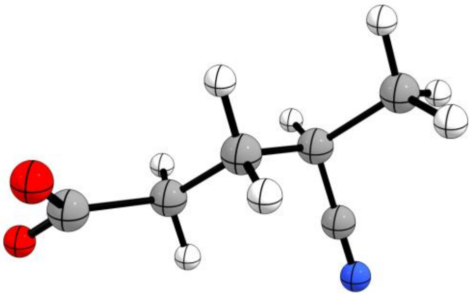

Geometry and vibrational frequencies: M062X-D3(0) def2-TZVP SMD(DMSO)

Charge: −1; Multiplicity: 1; # of imaginary frequencies: 0

G(298 K, 1 atm) = −438.69944061 Eh

G(298 K, 1 M) = G(298 K, 1 atm) + RTln(24.5) = −438.696422490367 Eh

| | | | |
|---|---|---|---|
| O | -3.571199000 | -3.697502000 | -0.362519000 |
| O | -5.618872000 | -3.292891000 | 0.451552000 |
| N | -3.243763000 | 1.868332000 | 0.986501000 |
| C | -4.528224000 | -2.953896000 | -0.054533000 |
| C | -4.314229000 | -1.449875000 | -0.338615000 |
| C | -5.495762000 | -0.561644000 | 0.005064000 |
| C | -4.131611000 | 1.451909000 | 0.389739000 |
| C | -6.519241000 | 1.768532000 | -0.052792000 |
| C | -5.281770000 | 0.917892000 | -0.346167000 |
| H | -5.026542000 | 1.000208000 | -1.406419000 |
| H | -4.059634000 | -1.345919000 | -1.397751000 |
| H | -3.424399000 | -1.138953000 | 0.217756000 |
| H | -5.734913000 | -0.643438000 | 1.067336000 |
| H | -6.379537000 | -0.894016000 | -0.541648000 |
| H | -6.355880000 | 2.814622000 | -0.310499000 |
| H | -7.354697000 | 1.391647000 | -0.642600000 |
| H | -6.784158000 | 1.705321000 | 1.003416000 |

**Carbon Dioxide Radical Anion**

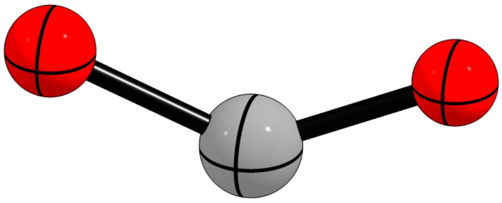

Geometry and vibrational frequencies: M062X-D3(0) def2-TZVP SMD(DMSO)

Charge: −1; Multiplicity: 2; # of imaginary frequencies: 0

G(298 K, 1 atm) = −188.67420080 Eh

G(298 K, 1 M) = G(298 K, 1 atm) + RTln(24.5) = −188.671182680367 Eh

| | | | |
|---|---|---|---|
| C | -4.729805000 | 2.140286000 | 0.121487000 |
| O | -3.933166000 | 1.531714000 | 0.829864000 |
| O | -5.939440000 | 2.093510000 | -0.081821000 |

**Benzonitrile Radical**

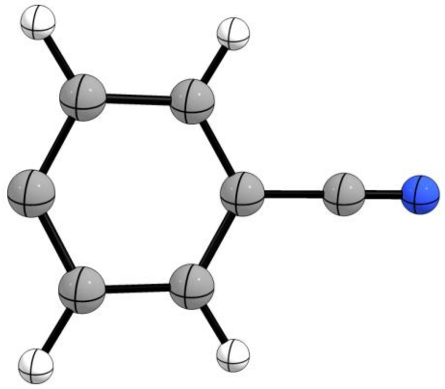

Geometry and vibrational frequencies: M062X-D3(0) def2-TZVP SMD(DMSO)

Charge: 0; Multiplicity: 2; # of imaginary frequencies: 0

G(298 K, 1 atm) = −323.74175189 Eh

G(298 K, 1 M) = *E* + RTln(24.5) = −323.738733770367 Eh

| | | | |
|---|---|---|---|
| C | -2.759420000 | 2.616156000 | 0.000000000 |
| C | -3.932665000 | 1.868987000 | 0.000000000 |
| C | -3.855117000 | 0.474646000 | 0.000000000 |
| C | -2.627821000 | -0.192024000 | 0.000000000 |
| C | -1.450542000 | 0.548280000 | 0.000000000 |
| C | -1.582671000 | 1.912912000 | 0.000000000 |
| C | -5.066412000 | -0.293501000 | 0.000000000 |
| N | -6.035135000 | -0.909641000 | 0.000001000 |
| H | -4.898130000 | 2.358469000 | -0.000001000 |
| H | -2.791604000 | 3.698212000 | 0.000000000 |
| H | -2.599012000 | -1.274019000 | 0.000000000 |
| H | -0.486532000 | 0.055663000 | 0.000000000 |

**Ethyl Acetoacetate Enolate**

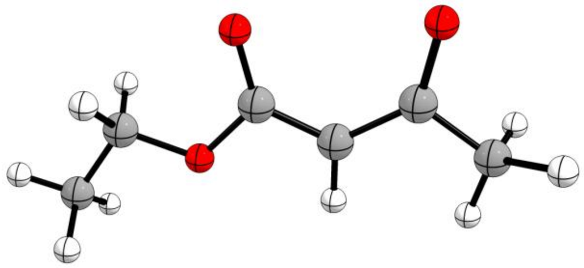

Geometry and vibrational frequencies: M062X-D3(0) def2-TZVP SMD(DMSO)

Charge: −1; Multiplicity: 1; # of imaginary frequencies: 0

G(298 K, 1 atm) = −459.75052184 Eh

G(298 K, 1 M) = $E$ + RTln(24.5) = −459.747503720367 Eh

| | | | |
|---|---|---|---|
| C | -3.195763000 | 0.216698000 | -0.005136000 |
| C | -2.356756000 | 1.487997000 | -0.111217000 |
| H | -2.605339000 | -0.684431000 | 0.159510000 |
| H | -3.779552000 | 0.096385000 | -0.920345000 |
| H | -3.905894000 | 0.329154000 | 0.817338000 |
| O | -2.975535000 | 2.547313000 | -0.311518000 |
| C | -0.962730000 | 1.316534000 | 0.027125000 |
| C | 0.014425000 | 2.353528000 | -0.022784000 |
| O | 1.288007000 | 1.829108000 | 0.071751000 |
| O | -0.128240000 | 3.562209000 | -0.128519000 |
| C | 2.356685000 | 2.765211000 | 0.027305000 |
| C | 3.653087000 | 1.991404000 | 0.072642000 |
| H | 2.281064000 | 3.454007000 | 0.872476000 |
| H | 2.288874000 | 3.360028000 | -0.886212000 |
| H | 3.723463000 | 1.401089000 | 0.987440000 |
| H | 4.498104000 | 2.681042000 | 0.043594000 |
| H | 3.731435000 | 1.316994000 | -0.781488000 |
| H | -0.582713000 | 0.314329000 | 0.164678000 |

### Ethyl Acetoacetate Enolate with $Cs^+$

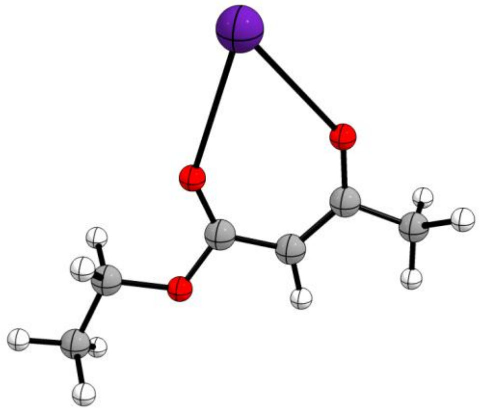

Geometry and vibrational frequencies: M062X-D3(0) def2-TZVP SMD(DMSO)

Charge: 0 ; Multiplicity: 1; # of imaginary frequencies: 0

G(298 K, 1 atm) = −479.80290624 Eh

G(298 K, 1 M) = $E$ + RTln(24.5) = −479.799888120367 Eh

| | | | |
|---|---|---|---|
| C | 4.201098000 | 0.876208000 | 0.508722000 |
| C | 3.215115000 | 2.020089000 | 0.641484000 |
| H | 3.722376000 | -0.087364000 | 0.342320000 |
| H | 4.806370000 | 0.825296000 | 1.416154000 |
| H | 4.878553000 | 1.088618000 | -0.320908000 |
| O | 3.709233000 | 3.157013000 | 0.841582000 |
| C | 1.853575000 | 1.701319000 | 0.529238000 |
| C | 0.794578000 | 2.644190000 | 0.617731000 |
| H | 1.573323000 | 0.672898000 | 0.357838000 |
| O | 0.872773000 | 3.858474000 | 0.809264000 |
| O | -0.429410000 | 2.071550000 | 0.462951000 |
| C | -1.558457000 | 2.942105000 | 0.560778000 |
| C | -2.802965000 | 2.110751000 | 0.364918000 |
| H | -1.482716000 | 3.723557000 | -0.197666000 |
| H | -1.558886000 | 3.427068000 | 1.539168000 |
| H | -2.804144000 | 1.635238000 | -0.616754000 |
| H | -3.684554000 | 2.749519000 | 0.436620000 |
| H | -2.878620000 | 1.335276000 | 1.128207000 |
| Cs | 2.816779000 | 5.653077000 | 1.718053000 |

## Radical–Anion Coupling Transition State

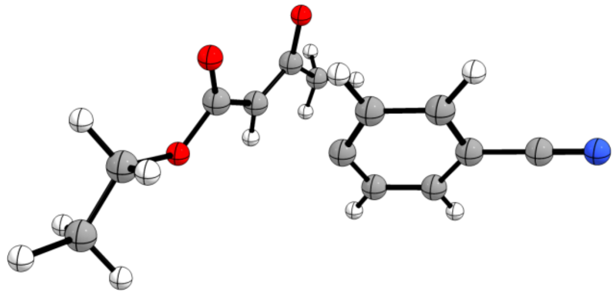

Geometry and vibrational frequencies: M062X-D3(0) def2-TZVP SMD(DMSO)

Charge: −1; Multiplicity: 2; # of imaginary frequencies: 1 (−127.06 $cm^{-1}$)

G(298 K, 1 atm) = −783.47764967 Eh

G(298 K, 1 M) = *E* + RTln(24.5) = −783.474631550367 Eh

| | | | |
|---|---|---|---|
| C | -2.618913000 | 1.472254000 | -0.310899000 |
| C | -3.835159000 | 2.057751000 | -0.604011000 |
| C | -4.970098000 | 1.589670000 | 0.043389000 |
| C | -4.848587000 | 0.556457000 | 0.979061000 |
| C | -3.605357000 | -0.008455000 | 1.273845000 |
| C | -2.472436000 | 0.460285000 | 0.618621000 |
| H | -1.503956000 | 0.028130000 | 0.844158000 |
| H | -3.895753000 | 2.879504000 | -1.309112000 |
| H | -5.944556000 | 2.015937000 | -0.161619000 |
| H | -3.534418000 | -0.803048000 | 2.006502000 |
| C | -6.018174000 | 0.072935000 | 1.648749000 |
| N | -6.958002000 | -0.315929000 | 2.183815000 |
| C | -0.836602000 | 3.314518000 | -0.526843000 |
| C | -1.471356000 | 4.051550000 | 0.505183000 |
| O | -2.359252000 | 4.905405000 | 0.373168000 |
| C | -1.039211000 | 3.704219000 | 1.923725000 |
| H | -0.213576000 | 2.994254000 | 1.965004000 |
| H | -1.896689000 | 3.276114000 | 2.450433000 |
| H | -0.756648000 | 4.616434000 | 2.452801000 |
| C | -1.123981000 | 3.423030000 | -1.925646000 |
| H | -0.027669000 | 2.650846000 | -0.259487000 |
| O | -1.987890000 | 4.075983000 | -2.486724000 |
| O | -0.277161000 | 2.633617000 | -2.660858000 |
| C | -0.511280000 | 2.601231000 | -4.064833000 |
| C | 0.501896000 | 1.667093000 | -4.681271000 |
| H | -0.419241000 | 3.608832000 | -4.476174000 |
| H | -1.530715000 | 2.258509000 | -4.256842000 |
| H | 1.517943000 | 2.016017000 | -4.491330000 |
| H | 0.351997000 | 1.619334000 | -5.760840000 |
| H | 0.398902000 | 0.659727000 | -4.275407000 |

**Radical–Anion Coupling with $Cs^+$ Transition State**

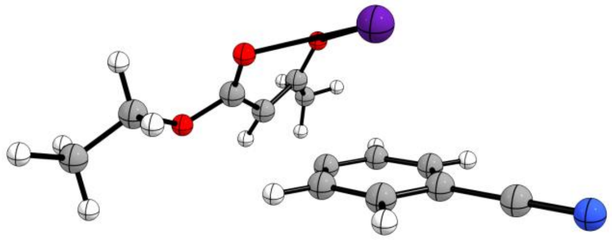

Geometry and vibrational frequencies: M062X-D3(0) def2-TZVP SMD(DMSO)

Charge: 0; Multiplicity: 2; # of imaginary frequencies: 1 (−119.52 $cm^{-1}$)

G(298 K, 1 atm) = −803.53306004 Eh

G(298 K, 1 M) = $E$ + RTln(24.5) = −803.530041920367 Eh

| | | | |
|---|---|---|---|
| C | -2.293770000 | 1.442581000 | 0.125052000 |
| C | -3.095299000 | 1.072032000 | -0.952711000 |
| C | -4.445356000 | 0.808180000 | -0.763625000 |
| C | -4.996996000 | 0.932460000 | 0.517106000 |
| C | -4.199433000 | 1.323849000 | 1.599728000 |
| C | -2.852759000 | 1.589188000 | 1.393240000 |
| H | -2.247177000 | 1.901878000 | 2.238162000 |
| H | -2.678965000 | 0.970982000 | -1.950386000 |
| H | -5.072314000 | 0.499405000 | -1.591630000 |
| H | -4.637897000 | 1.410093000 | 2.586818000 |
| C | -6.386552000 | 0.656581000 | 0.721383000 |
| N | -7.502536000 | 0.436113000 | 0.884736000 |
| C | -0.670947000 | 2.961042000 | -0.383461000 |
| C | -0.849078000 | 3.838456000 | 0.728111000 |
| O | -1.672359000 | 4.767386000 | 0.790857000 |
| C | 0.032789000 | 3.573833000 | 1.928070000 |
| H | 0.354142000 | 2.533629000 | 1.981671000 |
| H | -0.496059000 | 3.849101000 | 2.840113000 |
| H | 0.927819000 | 4.198251000 | 1.855205000 |
| C | -1.137548000 | 3.250153000 | -1.718345000 |
| H | 0.179784000 | 2.297311000 | -0.337345000 |
| O | -2.077278000 | 3.961145000 | -2.054726000 |
| O | -0.421563000 | 2.575690000 | -2.641685000 |
| C | -0.817093000 | 2.739201000 | -4.008475000 |
| C | 0.116439000 | 1.910121000 | -4.854203000 |
| H | -0.761201000 | 3.796558000 | -4.271886000 |
| H | -1.854115000 | 2.417958000 | -4.122347000 |
| H | 1.147930000 | 2.243552000 | -4.733762000 |
| H | -0.156830000 | 2.010306000 | -5.905205000 |
| H | 0.055876000 | 0.855896000 | -4.581352000 |
| Cs | -4.292215000 | 4.364453000 | -0.282606000 |

## Radical–Anion Product

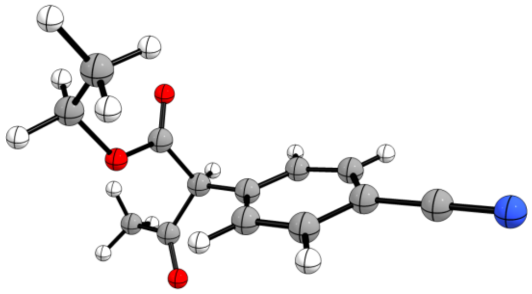

Geometry and vibrational frequencies: M062X-D3(0) def2-TZVP SMD(DMSO)

Charge: −1; Multiplicity: 2; # of imaginary frequencies: 0

G(298 K, 1 atm) = −783.52823361 Eh

G(298 K, 1 M) = $E$ + RTln(24.5) = −783.525215490367 Eh

| | | | |
|---|---|---|---|
| C | -4.349288000 | 3.204111000 | 0.742693000 |
| C | -3.196672000 | 2.265268000 | 0.982792000 |
| H | -5.189604000 | 2.606004000 | 0.378122000 |
| H | -4.125882000 | 3.950190000 | -0.020205000 |
| H | -4.643242000 | 3.689545000 | 1.671034000 |
| O | -2.999467000 | 1.764475000 | 2.061221000 |
| C | -2.346991000 | 1.938012000 | -0.248461000 |
| C | -1.746582000 | 3.206587000 | -0.828557000 |
| O | -1.644592000 | 3.444161000 | -2.006259000 |
| O | -1.278240000 | 4.006011000 | 0.136385000 |
| C | -0.487447000 | 5.136892000 | -0.274830000 |
| C | 0.929421000 | 4.711946000 | -0.583056000 |
| H | -0.521870000 | 5.820986000 | 0.570563000 |
| H | -0.963753000 | 5.608304000 | -1.133288000 |
| H | 1.395698000 | 4.261630000 | 0.294098000 |
| H | 1.516328000 | 5.584336000 | -0.874663000 |
| H | 0.950911000 | 3.993538000 | -1.403185000 |
| C | -1.233296000 | 0.948876000 | -0.052683000 |
| H | -3.039613000 | 1.565488000 | -1.008482000 |
| C | -1.076946000 | -0.139809000 | -0.944882000 |
| C | -0.243071000 | 1.116780000 | 0.952182000 |
| C | -0.036587000 | -1.025136000 | -0.843884000 |
| C | 0.808545000 | 0.251803000 | 1.079953000 |
| C | 0.955696000 | -0.869880000 | 0.187551000 |
| H | -1.811338000 | -0.281046000 | -1.732194000 |
| H | -0.326146000 | 1.947008000 | 1.643256000 |
| H | 1.541182000 | 0.400510000 | 1.864372000 |
| H | 0.042231000 | -1.853085000 | -1.537976000 |
| C | 2.011367000 | -1.764629000 | 0.322823000 |
| N | 2.898460000 | -2.517607000 | 0.443259000 |

## Radical–Anion Product with $Cs^+$

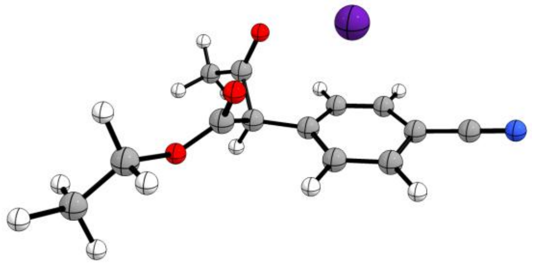

Geometry and vibrational frequencies: M062X-D3(0) def2-TZVP SMD(DMSO)

Charge: 0; Multiplicity: 2; # of imaginary frequencies: 0

G(298 K, 1 atm) = − 803.57464556 Eh

G(298 K, 1 M) = $E$ + RTln(24.5) = −803.571627440367 Eh

| | | | |
|---|---|---|---|
| C | 3.462382000 | 1.371780000 | -0.260928000 |
| C | 2.640901000 | 0.537794000 | 0.666289000 |
| H | 4.353032000 | 1.750322000 | 0.233768000 |
| H | 2.857490000 | 2.194965000 | -0.649613000 |
| H | 3.740794000 | 0.755763000 | -1.121069000 |
| O | 2.989226000 | 0.250320000 | 1.789135000 |
| C | 1.286328000 | 0.042755000 | 0.115717000 |
| C | 0.250436000 | 0.896445000 | 0.842484000 |
| O | -0.039761000 | 0.769277000 | 2.010195000 |
| O | -0.272154000 | 1.813841000 | 0.045919000 |
| C | -1.231674000 | 2.724478000 | 0.625251000 |
| C | -1.708549000 | 3.636571000 | -0.474699000 |
| H | -0.739720000 | 3.275045000 | 1.427551000 |
| H | -2.046727000 | 2.139996000 | 1.051933000 |
| H | -0.878912000 | 4.201925000 | -0.900042000 |
| H | -2.432744000 | 4.342514000 | -0.067243000 |
| H | -2.192228000 | 3.067824000 | -1.269314000 |
| C | 1.079674000 | -1.426706000 | 0.382501000 |
| H | 1.239403000 | 0.303744000 | -0.944414000 |
| C | -0.210060000 | -1.962620000 | 0.639697000 |
| C | 2.181795000 | -2.321547000 | 0.452689000 |
| C | -0.389313000 | -3.255541000 | 1.052683000 |
| C | 0.741809000 | -4.128619000 | 1.229306000 |
| C | 2.036560000 | -3.618977000 | 0.861308000 |
| C | 0.587984000 | -5.429456000 | 1.706357000 |
| N | 0.462464000 | -6.519145000 | 2.102846000 |
| H | -1.086089000 | -1.331152000 | 0.530190000 |
| H | -1.386595000 | -3.630698000 | 1.245882000 |
| H | 2.898016000 | -4.273856000 | 0.904899000 |
| H | 3.175843000 | -1.972556000 | 0.193962000 |
| Cs | 1.271088000 | -1.541389000 | 3.572461000 |

**Radical–Anion Product (Acetonitrile)**

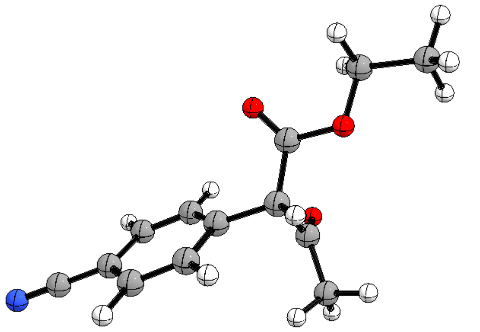

Geometry and vibrational frequencies: M062X-D3(0) def2-TZVP CPCM(MeCN)

Charge: −1; Multiplicity: 2; # of imaginary frequencies: 0

G(298 K) = −783.53296826 Eh

| | | | |
|---|---|---|---|
| O | -2.938874000 | -1.953657000 | 0.309825000 |
| O | -4.736293000 | -1.629974000 | 1.594910000 |
| O | -2.655888000 | 1.067518000 | 0.963882000 |
| N | -10.069105000 | 3.573949000 | 0.560135000 |
| C | -1.270402000 | -3.628696000 | 0.573276000 |
| C | -2.549733000 | -3.064229000 | 1.138832000 |
| C | -2.876421000 | 1.727285000 | -1.320098000 |
| C | -4.051821000 | -1.306897000 | 0.658451000 |
| C | -3.197004000 | 0.893757000 | -0.112477000 |
| C | -4.301855000 | -0.140233000 | -0.279685000 |
| C | -9.093619000 | 2.947016000 | 0.417458000 |
| C | -5.584737000 | 0.612260000 | -0.070916000 |
| C | -6.453946000 | 0.871598000 | -1.160672000 |
| C | -7.586597000 | 1.626934000 | -1.021127000 |
| C | -7.932718000 | 2.194388000 | 0.251753000 |
| C | -5.911948000 | 1.196322000 | 1.182366000 |
| C | -7.037030000 | 1.953569000 | 1.350877000 |
| H | -0.481500000 | -2.876624000 | 0.579365000 |
| H | -0.947471000 | -4.474262000 | 1.180431000 |
| H | -1.420360000 | -3.973998000 | -0.449559000 |
| H | -2.419365000 | -2.703437000 | 2.159676000 |
| H | -3.355739000 | -3.798596000 | 1.134064000 |
| H | -2.362924000 | 2.640415000 | -1.027551000 |
| H | -2.221138000 | 1.139169000 | -1.969385000 |
| H | -3.778718000 | 1.958834000 | -1.886130000 |
| H | -4.264958000 | -0.527495000 | -1.300684000 |
| H | -8.237396000 | 1.793271000 | -1.870808000 |
| H | -6.217206000 | 0.451118000 | -2.133204000 |
| H | -5.253608000 | 1.033277000 | 2.027997000 |
| H | -7.266206000 | 2.376929000 | 2.321270000 |

**Neutral Product (Acetonitrile)**

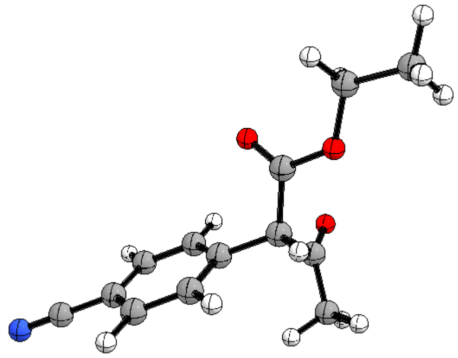

Geometry and vibrational frequencies: M062X-D3(0) def2-TZVP CPCM(MeCN)

Charge: 0; Multiplicity: 1; # of imaginary frequencies: 0

G(298 K) = −783.44925192 Eh

| | | | |
|---|---|---|---|
| O | -3.009146000 | -1.967525000 | 0.250853000 |
| O | -4.515049000 | -1.283716000 | 1.755150000 |
| O | -2.308186000 | 0.980891000 | 0.935862000 |
| N | -10.183346000 | 3.188185000 | 0.514348000 |
| C | -1.584824000 | -3.868070000 | 0.396528000 |
| C | -2.654914000 | -3.076250000 | 1.104379000 |
| C | -2.816328000 | 2.031453000 | -1.144527000 |
| C | -3.938893000 | -1.140710000 | 0.709024000 |
| C | -2.997741000 | 1.021815000 | -0.053326000 |
| C | -4.142392000 | 0.015331000 | -0.255500000 |
| C | -9.173338000 | 2.657195000 | 0.385844000 |
| C | -5.472552000 | 0.708429000 | -0.078557000 |
| C | -6.411724000 | 0.679977000 | -1.105243000 |
| C | -7.632368000 | 1.318304000 | -0.960501000 |
| C | -7.910897000 | 1.993760000 | 0.226037000 |
| C | -5.760413000 | 1.392129000 | 1.102014000 |
| C | -6.975488000 | 2.034045000 | 1.260575000 |
| H | -0.703834000 | -3.251974000 | 0.217866000 |
| H | -1.294910000 | -4.716341000 | 1.016224000 |
| H | -1.952224000 | -4.245470000 | -0.557557000 |
| H | -2.306272000 | -2.675311000 | 2.056448000 |
| H | -3.551387000 | -3.669477000 | 1.284730000 |
| H | -3.774230000 | 2.480762000 | -1.410328000 |
| H | -2.106042000 | 2.794536000 | -0.838303000 |
| H | -2.445077000 | 1.511944000 | -2.031584000 |
| H | -4.066225000 | -0.368364000 | -1.274496000 |
| H | -8.364241000 | 1.295440000 | -1.756619000 |
| H | -6.187227000 | 0.153583000 | -2.024597000 |
| H | -5.031910000 | 1.420234000 | 1.902293000 |
| H | -7.203403000 | 2.564704000 | 2.175232000 |

## 4. Carbonyl α-(Hetero)Arylation Optimisation Studies

The effect of the countercation of the formate salt was investigated for the reaction of 4-bromobenzonitrile **1**, ethyl acetoacetate **2**, ACVA and $Cs_2CO_3$ in DMSO (**Table S1**). With $HCO_2Na$, **4** was formed in 59% and incomplete consumption of **1** was noted (entry 1). Conversely, $HCO_2K$ and $HCO_2Cs$ afforded **4** in 79% and 76% yield respectively (entries 2–3). The higher yields observed with $HCO_2K$ and $HCO_2Cs$ may be due to their apparent superior solubility in DMSO. The role of adventitious water cannot be ruled out due to the highly hygroscopic nature of $HCO_2K$ and $HCO_2Cs$. However, later studies showed that water had a deleterious effect on product formation (Table S5, entries 6–8).

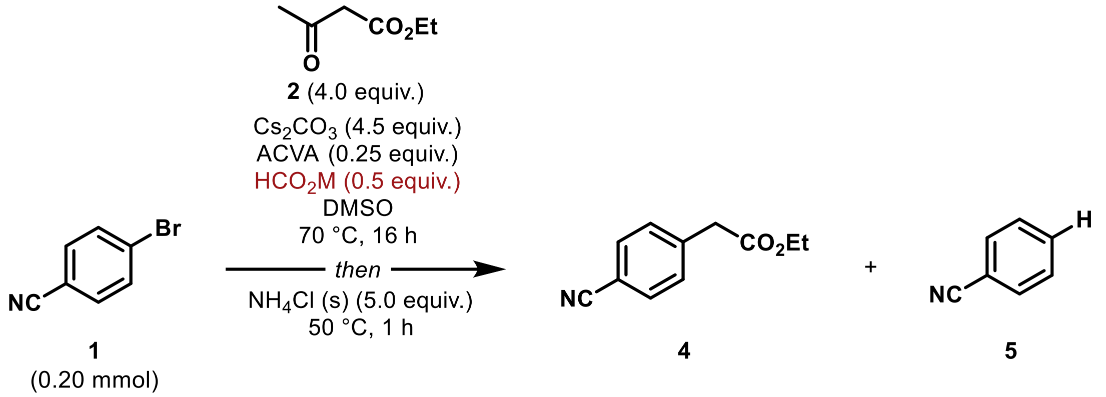


| Entry | Formate salt | Yield of 4 / % | Yield of 5 / % | Yield of 1 / % | Mass balance / % |
|---|---|---|---|---|---|
| 1 | $HCO_2Na$ | 59 | 10 | 13 | 82 |
| 2 | $HCO_2K$ | 79 | 10 | 0 | 89 |
| 3 | $HCO_2Cs$ | 76 | 10 | 2 | 88 |

**Table S1**. Optimisation of the formate salt countercation. Yields determined by $^1H$ NMR spectroscopy against an internal standard (1,3,5-trimethoxybenzene).

The effect of reaction temperature and duration was investigated using the same reaction system (**Table S2**). Performing the reaction at 60 °C for 4 h furnished **4** in 43% yield with significant amounts of unconverted **1** (entry 1). Increasing the reaction temperature to 70 °C improved the yield of **4** to 75% with 7% unreacted **1** remaining (entry 2). Complete conversion of **1** was achieved by increasing the reaction time from 4 h to 16 h at 70 °C with a slight improvement in yield to 79% (entry 3). The same yield and conversion could be achieved at 80 °C for 4 h (79% yield of **4**, entry 4). Thus, 80 °C for 4 h was chosen as the most efficient optimal conditions. Higher temperatures were not attempted as the the autocatalytic decomposition of DMSO in basic media is known to occur at temperatures above 80 °C.[53]

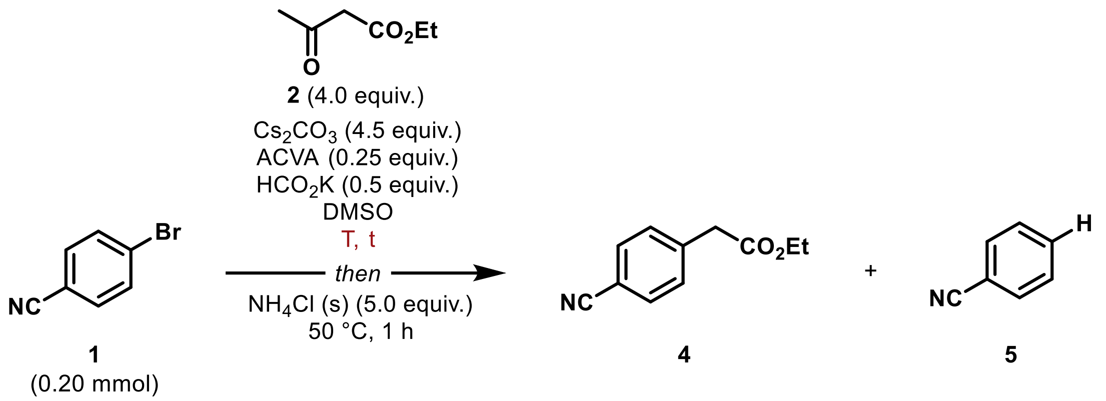


| Entry | Conditions | Yield of 4 / % | Yield of 5 / % | Yield of 1 / % | Mass balance / % |
|---|---|---|---|---|---|
| 1 | 60 °C, 4 h | 43 | 5 | 43 | 91 |
| 2 | 70 °C, 4 h | 75 | 10 | 7 | 92 |
| 3 | 70 °C, 16 h | 79 | 10 | 0 | 89 |
| 4 | 80 °C, 4 h | 79 | 10 | 0 | 89 |

**Table S2**. Optimisation of reaction temperature and time. Yields determined by $^1$H NMR spectroscopy against an internal standard (1,3,5-trimethoxybenzene).

To determine the effect of base on the reaction, several bases were screened for the reaction of 4′-bromoacetophenone **S1**, ethyl acetoacetate **2**, ACVA and $HCO_2K$ in DMSO (**Table S3**). In the absence of base, the product **14** was formed while acetophenone **S2** was observed in 33% yield (entry 1). Inorganic bases $Cs_2CO_3$ and $K_3PO_4$ afforded **S2** in 69% and 59% respectively (entries 2, 3). Low yields were observed with LiHMDS, NaHMDS and KHMDS (entries 4–6). Most hydroxide and alkoxide bases afforded **S2** in low yields (entries 7–15), except for KO*t*Bu, $CsOH{\cdot}H_2O$ and $Me_4NOH{\cdot}5H_2O$ which afforded **S2** in 57%, 63 and 65% yield, respectively. Organic bases were also examined, but the yields of **S2** were generally poor (10, 25% yield, entries 16, 17). Interestingly, no clear trend was observed regarding the homogeneity of the reactions and product yield. The reaction mixture was highly heterogenous when using $Cs_2CO_3$, but appeared far more homogenous when using KO*t*-Bu and $Me_4NOH{\cdot}5H_2O$, which may be due to the presence of adventitious water. However, $Cs_2CO_3$ was retained as the optimal base in an effort to prioritise operational simplicity.

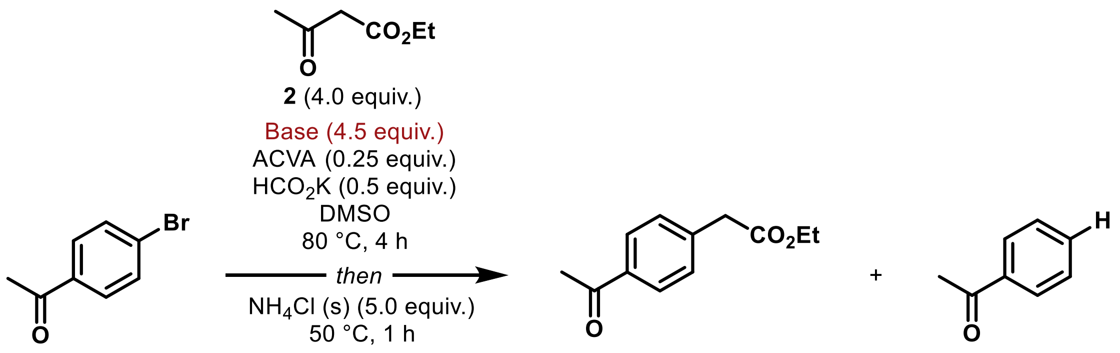


| Entry | Base | Yield of 14 / % | Yield of S2 / % | Yield of S1 / % | Mass balance / % |
|---|---|---|---|---|---|
| 1 | None | 0 | 33 | 56 | 89 |
| 2 | $Cs_2CO_3$ | 69 | 24 | 0 | 93 |
| 3 | $K_3PO_4$ | 59 | 29 | 0 | 88 |
| 4 | LiHMDS (1 M in THF) | 6 | 20 | 45 | 71 |
| 5 | NaHMDS (1 M in THF) | 8 | 13 | 49 | 70 |
| 6 | KHMDS (1 M in THF) | 18 | 26 | 18 | 62 |
| 7 | LiOH | 12 | 27 | 60 | 99 |
| 8 | NaOMe | 25 | 18 | 47 | 90 |
| 9 | KOEt | 18 | 44 | 20 | 82 |
| 10 | NaO*t*Bu | 28 | 22 | 48 | 98 |
| 11 | KO*t*Bu | 57 | 21 | 0 | 78 |
| 12 | NaO*t*-Am | 11 | 20 | 46 | 77 |
| 13 | KO*t*-Am (0.9 M in cyclohexane) | 23 | 15 | 58 | 96 |
| 14 | $CsOH{\cdot}H_2O$ | 63 | 27 | 0 | 90 |
| 15 | $Me_4NOH{\cdot}5H_2O$ | 65 | 29 | 0 | 94 |
| 16 | TMG | 10 | 26 | 47 | 83 |
| 17 | DBU | 10 | 34 | 56 | 100 |

**Table S3**. Base screening. Yields determined by $^1$H NMR spectroscopy against an internal standard (1,3,5-trimethoxybenzene).

## Comparison to Other Methods of Initiation

Other methods of initiation to promote the coupling between 4-bromobenzonitrile **1** and ethyl acetoacetate **2** were compared to the optimised ACVA-formate initiator system (**Table S4**, entry 1). Interestingly, the azo initiator (ACVA) used to form $CO_2^{\bullet-}$ through HAT could be swapped for a persulfate ($K_2S_2O_8$) or peroxide ($(t\mathrm{BuO})_2$), but the yield of **4** was diminished and incomplete consumption of **1** was observed in both cases (entries 2, 3). These results are perhaps unsurprising as persulfate and peroxides are both oxidants and can therefore easily interfere with electron-catalysed processes. Precursors to sulfur dioxide radical anion ($SO_2^{\bullet-}$, a weaker electron donor than $CO_2^{\bullet-}$) were also trialled (entries 4, 5). Sodium dithionite could be used to afford **4** in 41% yield, but no product was observed using rongalite. The mass balance was also poor in both cases, indicating that unwanted side reactions were occurring (e.g. the coupling of aryl radicals with $SO_2^{\bullet-}$).[64] Finally, irradiated the reaction mixture with 450 nm light in the absence of a chemical electron donor, enabled **4** to be formed in 72% yield after 5 h (entry 6). In addition to a lower yield of **4**, the mass balance of the reaction was also lower than the optimal ACVA-formate system. This change in mass balance was attributed to the potential photoexcitation and decomposition of **2** and **4**, as both components form light sensitive carbanions under the reaction conditions.

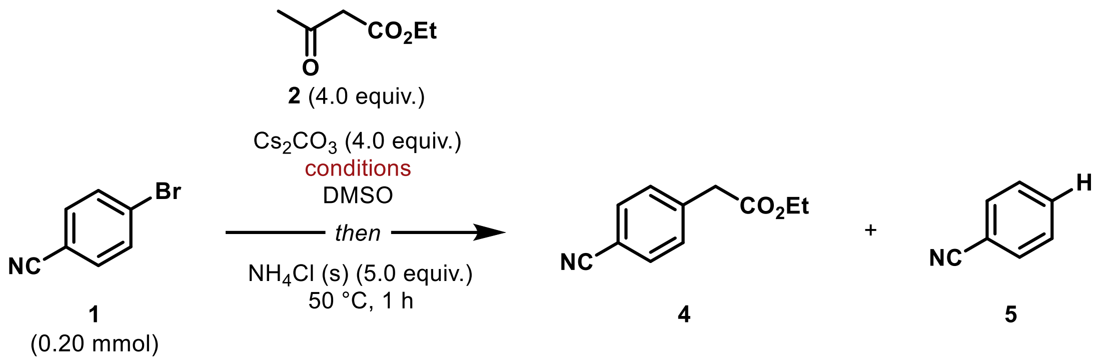


| Entry | Conditions | Yield of 4 / % | Yield of 5 / % | Yield of 1 / % | Mass balance / % |
|---|---|---|---|---|---|
| 1 | ACVA (0.25 equiv.)<br>$HCO_2K$ (0.5 equiv.)<br>80 °C, 4 h | 79 | 10 | 0 | 89 |
| 2 | $K_2S_2O_8$ (1.0 equiv.)<br>$HCO_2K$ (1.0 equiv.)<br>80 °C, 6 h | 52 | 9 | 20 | 81 |
| 3 | ($t$BuO)$_2$ (0.5 equiv.)<br>$HCO_2K$ (0.5 equiv.)<br>80 °C, 4 h | 29 | 6 | 56 | 91 |
| 4 | $Na_2S_2O_4$ (2.0 equiv.)<br>80 °C, 4 h | 41 | n.d. | 31 | 72 |
| 5 | Rongalite (2.0 equiv.)<br>80 °C, 4 h | n.d. | n.d. | 54 | 54 |
| 6 | hν (450 nm), 5 h | 72 | n.d. | n.d. | 72 |

**Table S4**. Variation of the method of initiation. Yields determined by $^1$H NMR spectroscopy against an internal standard (1,3,5-trimethoxybenzene). n.d., not detected.

## 5. Experimental Procedures and Characterisation Data

### General Procedure A

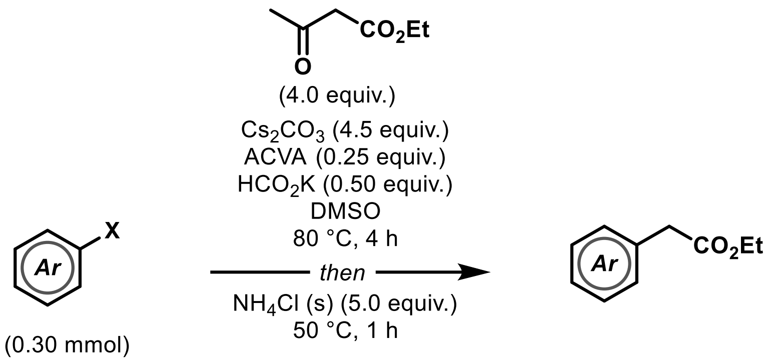


To an 8 mL screw-cap vial was charged ACVA (25.6 mg, 75 µmol, 0.25 equiv.), $HCO_2K$ (12.6 mg, 150 µmol, 0.50 equiv.), $Cs_2CO_3$ (440 mg, 1.35 mmol, 4.5 equiv.), and if solid, the aryl halide coupling partner (1.0 equiv.). To the solids was sequentially added a magnetic stirrer bar, anhydrous DMSO (1.5 mL), ethyl acetoacetate (156 mg, 1.20 mmol, 4.0 equiv.), and if liquid the aryl halide coupling partner through the screw-cap septa. The reaction mixture was sparged with $N_2$ for 15 minutes before being sealed with parafilm. The reaction mixture was then stirred at 80 °C in a metal heating block for 4 hours. To promote complete deacetylation of intermediate 1,3-dicarbonyls, $NH_4Cl$ (80.3 mg, 1.50 mmol, 5.0 equiv.) was added. The reaction was then stirred at 50 °C in a metal heating block for 1 hour. The reaction mixture was cooled to room temperature before being diluted with $CH_2Cl_2$ (10 mL) and water (10 mL). The organic phase was collected, and the aqueous phase was extracted with $CH_2Cl_2$ (3 × 10 mL). The organics were combined, washed with brine (25 mL), dried ($MgSO_4$), and concentrated under reduced pressure. The crude product was then purified by column chromatography to afford the stated product.

**Ethyl 2-(4-cyanophenyl)acetate (4)**

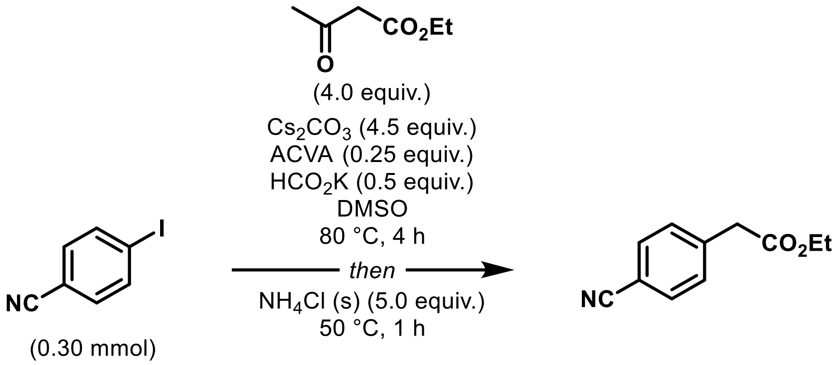


Synthesised according to **General Procedure A** with 4-iodobenzonitrile (68.7 mg, 0.300 mmol, 1.0 equiv.), ethyl acetoacetate (156 mg, 1.20 mmol, 4.0 equiv.), $Cs_2CO_3$ (440 mg, 1.35 mmol, 4.5 equiv.), ACVA (25.6 mg, 75 µmol, 0.25 equiv.) and $HCO_2K$ (12.6 mg, 150 µmol, 0.5 equiv.) in anhydrous DMSO (1.5 mL). The reaction mixture was heated at 80 °C for 4 hours. $NH_4Cl$ (80.3 mg, 1.50 mmol, 5.0 equiv.) was then added and the reaction mixture was stirred at 50 °C for 1 hour. The crude product was purified by column chromatography (5% $Et_3N$ in pentane) to afford the *title compound* **4** as a pale yellow solid (30.8 mg, 0.163 mmol, 54%).

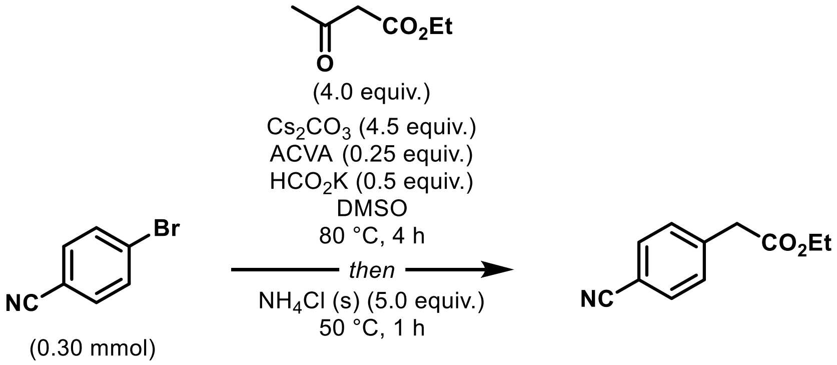


Synthesised according to **General Procedure A** with 4-bromobenzonitrile (54.6 mg, 0.300 mmol, 1.0 equiv.), ethyl acetoacetate (156 mg, 1.20 mmol, 4.0 equiv.), $Cs_2CO_3$ (440 mg, 1.35 mmol, 4.5 equiv.), ACVA (25.6 mg, 75 µmol, 0.25 equiv.) and $HCO_2K$ (12.6 mg, 150 µmol, 0.5 equiv.) in anhydrous DMSO (1.5 mL). The reaction mixture was heated at 80 °C for 4 hours. $NH_4Cl$ (80.3 mg, 1.50 mmol, 5.0 equiv.) was then added and the reaction mixture was stirred at 50 °C for 1 hour. The crude product was purified by column chromatography (5% $Et_3N$ in pentane) to afford the *title compound* **4** as a pale yellow solid (38.2 mg, 0.202 mmol, 67%).

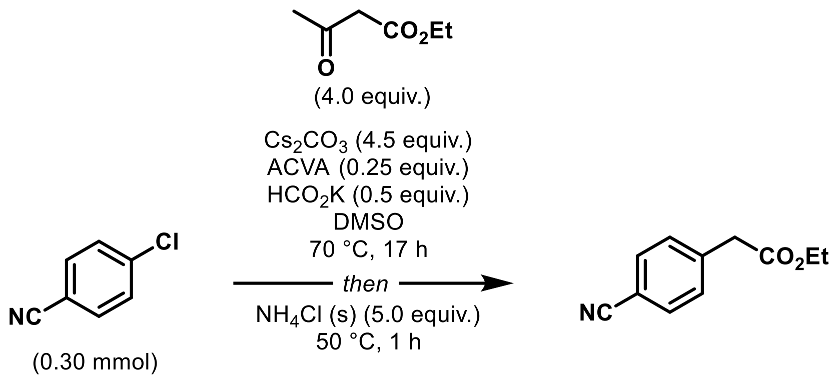

Synthesised according to **General Procedure A** with 4-chlorobenzonitrile (41.3 mg, 0.300 mmol, 1.0 equiv.), ethyl acetoacetate (156 mg, 1.20 mmol, 4.0 equiv.), $Cs_2CO_3$ (440 mg, 1.35 mmol, 4.5 equiv.), ACVA (25.6 mg, 75 µmol, 0.25 equiv.) and $HCO_2K$ (12.6 mg, 150 µmol, 0.5 equiv.) in anhydrous DMSO (1.5 mL). The reaction mixture was heated at 70 °C for 17 hours. $NH_4Cl$ (80.3 mg, 1.50 mmol, 5.0 equiv.) was then added and the reaction mixture was stirred at 50 °C for 1 hour. The crude product was purified by column chromatography (5% → 10% $Et_3N$ in pentane) to afford the *title compound* **4** as a pale yellow solid (30.2 mg, 0.160 mmol, 53%).

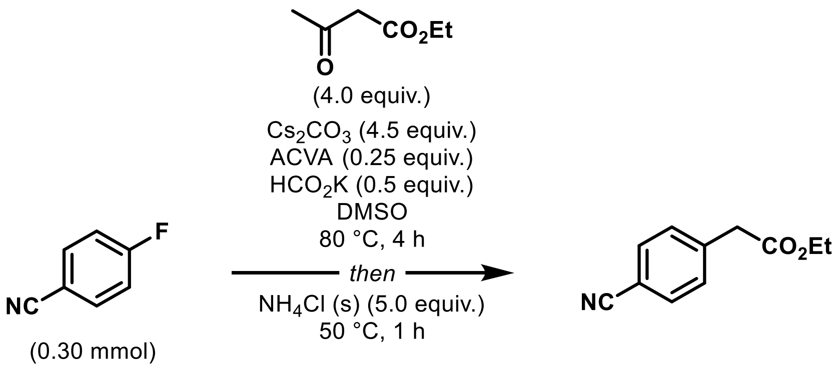

Synthesised according to **General Procedure A** with 4-fluorobenzonitrile (36.3 mg, 0.300 mmol, 1.0 equiv.), ethyl acetoacetate (156 mg, 1.20 mmol, 4.0 equiv.), $Cs_2CO_3$ (440 mg, 1.35 mmol, 4.5 equiv.), ACVA (25.6 mg, 75 µmol, 0.25 equiv.) and $HCO_2K$ (12.6 mg, 150 µmol, 0.5 equiv.) in anhydrous DMSO (1.5 mL). The reaction mixture was heated at 80 °C for 4 hours. $NH_4Cl$ (80.3 mg, 1.50 mmol, 5.0 equiv.) was then added and the reaction mixture was stirred at 50 °C for 1 hour. 1,3,5-Trimethoxybenzene (50.5 mg, 0.300 mmol, 1.0 equiv.) was added to the reaction mixture, which was then analysed by $^1$H NMR spectroscopy to approximate the yield of *title compound* **4** (7% determined by $^1$H NMR).

**mp** 82–84 °C. ***R**$_f$* 0.24 (10% $Et_3N$ in pentane).

**$^{1}$H NMR** (400 MHz, $CDCl_3$) $\delta_H$ 7.62 (d, $J$ = 8.4 Hz, 2H), 7.40 (d, $J$ = 8.4 Hz, 2H), 4.16 (q, $J$ = 7.1 Hz, 2H), 3.67 (s, 2H), 1.26 (t, $J$ = 7.1 Hz, 3H).

**$^{13}$C NMR** (101 MHz, $CDCl_3$) $\delta_C$ 170.5 (C), 139.6 (C), 132.5 (2CH), 130.3 (2CH), 118.9 (C), 111.3 (C), 61.5 ($CH_2$), 41.5 ($CH_2$), 14.3 ($CH_3$).

**HRMS** (APCI$^+$) m/z calcd. for $C_{11}H_{12}O_2N$ $(M + H)^+$ 190.0863, found 190.0871.

**Ethyl 2-(3-cyanophenyl)acetate (6)**

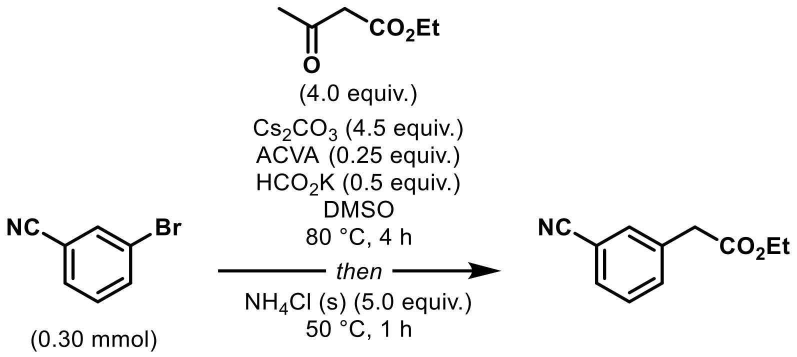


Synthesised according to **General Procedure A** with 3-bromobenzonitrile (54.6 mg, 0.300 mmol, 1.0 equiv.), ethyl acetoacetate (156 mg, 1.20 mmol, 4.0 equiv.), $Cs_2CO_3$ (440 mg, 1.35 mmol, 4.5 equiv.), ACVA (25.6 mg, 75 µmol, 0.25 equiv.) and $HCO_2K$ (12.6 mg, 150 µmol, 0.5 equiv.) in anhydrous DMSO (1.5 mL). The reaction mixture was heated at 80 °C for 4 hours. $NH_4Cl$ (80.3 mg, 1.50 mmol, 5.0 equiv.) was then added and the reaction mixture was stirred at 50 °C for 1 hour. The crude product was purified by column chromatography (5 → 10% $Et_3N$ in pentane) to afford the *title compound* **6** as a white solid (29.2 mg, 0.154 mmol, 51%).

**mp** 51–52 °C. ***$R_f$*** 0.14 (10% $Et_3N$ in pentane).

**$^{1}$H NMR** (400 MHz, $CDCl_3$) $\delta_H$ 7.62 – 7.56 (m, 2H), 7.53 (ddd, $J$ = 7.6, 1.6, 1.5 Hz, 1H), 7.44 (dd, $J$ = 7.6, 7.6 Hz, 1H), 4.17 (q, $J$ = 7.1 Hz, 2H), 3.65 (s, 2H), 1.26 (t, $J$ = 7.1 Hz, 3H).

**$^{13}$C NMR** (101 MHz, $CDCl_3$) $\delta_C$ 170.6 (C), 135.7 (C), 134.0 (CH), 133.0 (CH), 131.0 (CH), 129.5 (CH), 118.8 (C), 112.8 (C), 61.4 ($CH_2$), 40.9 ($CH_2$), 14.3 ($CH_3$).

**HRMS** (ESI$^+$) m/z calcd. for $C_{11}H_{12}NO_2$ $(M + H)^+$ 212.0682, found 212.0676.

**Ethyl 2-(2-cyanophenyl)acetate (7)**

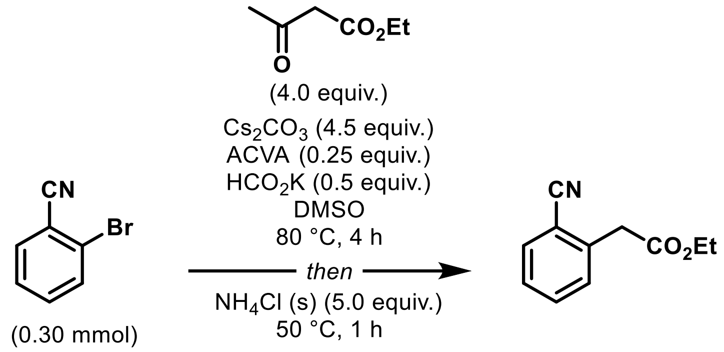


Synthesised according to **General Procedure A** with 2-bromobenzonitrile (54.6 mg, 0.300 mmol, 1.0 equiv.), ethyl acetoacetate (156 mg, 1.20 mmol, 4.0 equiv.), $Cs_2CO_3$ (440 mg, 1.35 mmol, 4.5 equiv.), ACVA (25.6 mg, 75 μmol, 0.25 equiv.) and $HCO_2K$ (12.6 mg, 150 μmol, 0.5 equiv.) in anhydrous DMSO (1.5 mL). The reaction mixture was heated at 80 °C for 4 hours. $NH_4Cl$ (80.3 mg, 1.50 mmol, 5.0 equiv.) was then added and the reaction mixture was stirred at 50 °C for 1 hour. The crude product was purified by column chromatography (5 → 10% $Et_3N$ in pentane) to afford the *title compound* **7** as a white solid (43.2 mg, 0.228 mmol, 76%).

**mp** 48–50 °C. ***R**f* 0.29 (10% $Et_3N$ in pentane).

**$^1$H NMR** (500 MHz, $CDCl_3$) $\delta_H$ 7.66 (d, $J$ = 7.7 Hz, 1H), 7.56 (dd, $J$ = 7.7, 1.1 Hz, 1H), 7.42 (d, $J$ = 7.7 Hz, 1H), 7.38 (dd, $J$ = 7.7, 7.7 Hz, 1H), 4.19 (q, $J$ = 7.1 Hz, 2H), 3.87 (s, 2H), 1.27 (t, $J$ = 7.1 Hz, 3H).

**$^{13}$C NMR** (101 MHz, $CDCl_3$) $\delta_C$ 169.9 (C), 138.0 (C), 133.01 (CH), 132.99 (CH), 130.8 (CH), 127.9 (CH), 117.7 (C), 113.6 (C), 61.6 ($CH_2$), 39.8 ($CH_2$), 14.3 ($CH_3$).

**HRMS** ($ESI^+$) m/z calcd. for $C_{11}H_{11}NNaO_2$ $(M + Na)^+$ 212.0687, found 212.0684.

**Ethyl 2-(4-(methylsulfonyl)phenyl)acetate (8)**

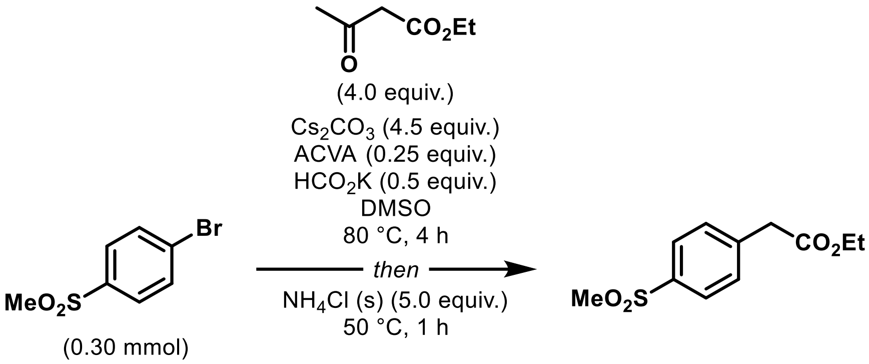


Synthesised according to **General Procedure A** with 1-bromo-4-(methylsulfonyl)benzene (70.5 mg, 0.300 mmol, 1.0 equiv.), ethyl acetoacetate (156 mg, 1.20 mmol, 4.0 equiv.), $Cs_2CO_3$ (440 mg, 1.35 mmol, 4.5 equiv.), ACVA (25.6 mg, 75 μmol, 0.25 equiv.) and $HCO_2K$ (12.6 mg, 150 μmol, 0.5 equiv.) in anhydrous DMSO (1.5 mL). The reaction mixture was heated at 80 °C for 4 hours. $NH_4Cl$ (80.3 mg, 1.50 mmol, 5.0 equiv.) was then added and the reaction mixture was stirred at 50 °C for 1 hour. The crude product was purified by column chromatography (40 → 50% EtOAc in pentane) to afford the *title compound* **8** as a white solid (34.4 mg, 0.142 mmol, 47%).

**mp** 68–69 °C. $\boldsymbol{R_f}$ 0.19 (30% EtOAc in pentane).

**$^1$H NMR** (400 MHz, $CDCl_3$) $\delta_H$ 7.90 (d, $J$ = 8.4 Hz, 2H), 7.49 (d, $J$ = 8.4 Hz, 2H), 4.17 (q, $J$ = 7.1 Hz, 2H), 3.71 (s, 2H), 3.04 (s, 3H), 1.26 (t, $J$ = 7.1 Hz, 3H).

**$^{13}$C NMR** (101 MHz, $CDCl_3$) $\delta_C$ 170.5 (C), 140.5 (C), 139.5 (C), 130.5 (2CH), 127.8 (2CH), 61.5 ($CH_2$), 44.7 ($CH_3$), 41.2 ($CH_2$), 14.3 ($CH_3$).

**HRMS** (ESI$^+$) m/z calcd. for $C_{11}H_{14}NaO_4S$ $(M + Na)^+$ 265.0510, found 265.0517.

**Ethyl 2-(3-(methylsulfonyl)phenyl)acetate (9)**

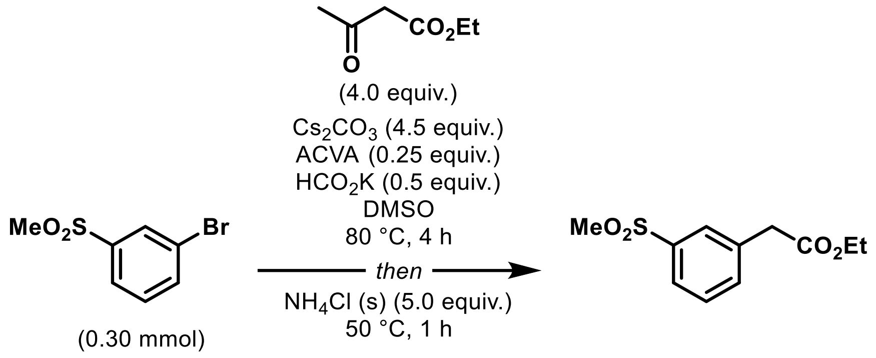

Synthesised according to **General Procedure A** with 1-bromo-3-(methylsulfonyl)benzene (70.5 mg, 0.300 mmol, 1.0 equiv.), ethyl acetoacetate (156 mg, 1.20 mmol, 4.0 equiv.), $Cs_2CO_3$ (440 mg, 1.35 mmol, 4.5 equiv.), ACVA (25.6 mg, 75 µmol, 0.25 equiv.) and $HCO_2K$ (12.6 mg, 150 µmol, 0.5 equiv.) in anhydrous DMSO (1.5 mL). The reaction mixture was heated at 80 °C for 4 hours. $NH_4Cl$ (80.3 mg, 1.50 mmol, 5.0 equiv.) was then added and the reaction mixture was stirred at 50 °C for 1 hour. The crude product was purified by column chromatography (30 → 40% $Et_2O$ in pentane) to afford the *title compound* **9** as a colourless oil (47.6 mg, 0.196 mmol, 65%).

***R*$_f$** 0.57 (30% $Et_2O$ in pentane).

**$^1$H NMR** (400 MHz, $CDCl_3$) $\delta_H$ 7.88 – 7.83 (m, 2H), 7.59 (ddd, *J* = 7.8, 1.6 Hz, 1H), 7.54 (dd, *J* = 7.8 Hz, 1H), 4.17 (q, *J* = 7.1 Hz, 2H), 3.71 (s, 2H), 3.06 (s, 3H), 1.27 (t, *J* = 7.1 Hz, 3H).

**$^{13}$C NMR** (101 MHz, $CDCl_3$) $\delta_C$ 170.7 (C), 140.9 (C), 136.0 (C), 134.9 (CH), 129.7 (CH), 128.4 (CH), 126.2 (CH), 61.4 ($CH_2$), 44.7 ($CH_3$), 41.0 ($CH_2$), 14.3 ($CH_3$).

**HRMS** ($ESI^+$) m/z calcd. for $C_{11}H_{14}NaO_4S$ $(M + Na)^+$ 265.0510, found 265.0514.

**Ethyl 2-(2-(methylsulfonyl)phenyl)acetate (10)**

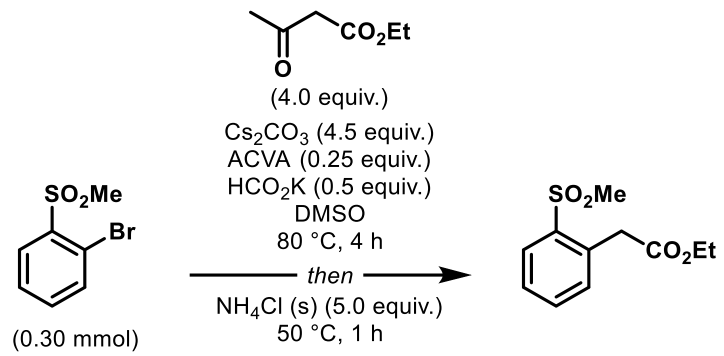


Synthesised according to **General Procedure A** with 2-bromophenyl methyl sulfone (70.5 mg, 0.300 mmol, 1.0 equiv.), ethyl acetoacetate (156 mg, 1.20 mmol, 4.0 equiv.), $Cs_2CO_3$ (440 mg, 1.35 mmol, 4.5 equiv.), ACVA (25.6 mg, 75 μmol, 0.25 equiv.) and $HCO_2K$ (12.6 mg, 150 μmol, 0.5 equiv.) in anhydrous DMSO (1.5 mL). The reaction mixture was heated at 80 °C for 4 hours. $NH_4Cl$ (80.3 mg, 1.50 mmol, 5.0 equiv.) was then added and the reaction mixture was stirred at 50 °C for 1 hour. 1,3,5-Trimethoxybenzene (50.5 mg, 0.300 mmol, 1.0 equiv.) was added to the reaction mixture, which was then analysed by $^1H$ NMR spectroscopy to approximate the yield of **10** (39% determined by $^1H$ NMR).

**Ethyl 2-(4-nitrophenyl)acetate (11)**

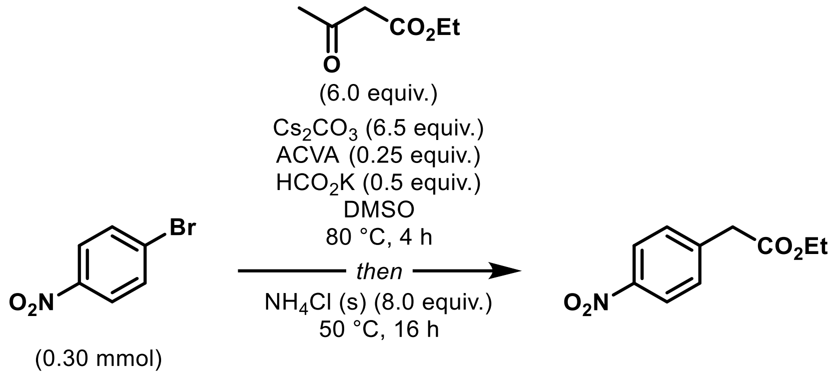


Synthesised according to **General Procedure A** with 1-bromo-4-nitrobenzene (60.6 mg, 0.300 mmol, 1.0 equiv.), ethyl acetoacetate (234 mg, 1.80 mmol, 6.0 equiv.), $Cs_2CO_3$ (635 mg, 1.95 mmol, 6.5 equiv.), ACVA (25.6 mg, 75 µmol, 0.25 equiv.) and $HCO_2K$ (12.6 mg, 150 µmol, 0.5 equiv.) in anhydrous DMSO (1.5 mL). The reaction mixture was heated at 80 °C for 4 hours. $NH_4Cl$ (128 mg, 2.40 mmol, 8.0 equiv.) was then added and the reaction mixture was stirred at 50 °C for 16 hours. The crude product was purified by column chromatography (10 → 20% $Et_2O$ in pentane) to afford the *title compound* **11** as a yellow solid (53.6 mg, 0.256 mmol, 85%).

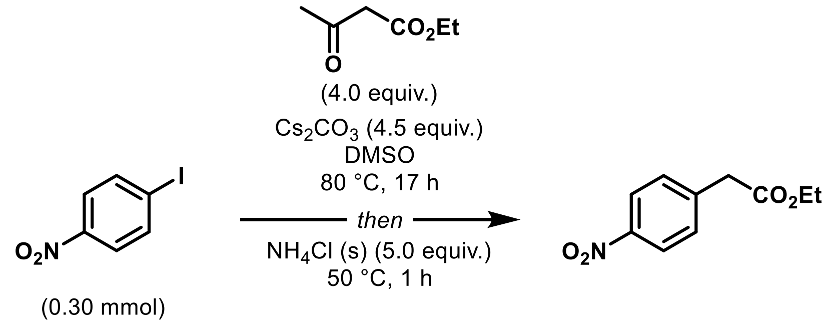


Note: ACVA-$HCO_2K$ mixture was not added.

Synthesised according to **General Procedure A** with 1-iodo-4-nitrobenzene (74.7 mg, 0.300 mmol, 1.0 equiv), ethyl acetoacetate (156 mg, 1.20 mmol, 4.0 equiv.), $Cs_2CO_3$ (440 mg, 1.35 mmol, 4.5 equiv.) in anhydrous DMSO (1.5 mL). The reaction mixture was heated at 80 °C for 17 hours. $NH_4Cl$ (128 mg, 2.40 mmol, 8.0 equiv.) was then added and the reaction mixture was stirred at 50 °C for 1 hour. The crude product was purified by column chromatography (5% $Et_3N$ in pentane) to afford the *title compound* **11** as a yellow solid (41.5 mg, 0.198 mmol, 66%).

**mp** 54–56 °C. ***R**$_f$* 0.45 (20% $Et_2O$ in pentane).

**$^1$H NMR** (400 MHz, $CDCl_3$) $\delta_H$ 8.19 (d, $J$ = 8.6 Hz, 2H), 7.46 (d, $J$ = 8.6 Hz, 2H), 4.18 (q, $J$ = 7.1 Hz, 2H), 3.72 (s, 2H), 1.26 (t, $J$ = 7.1 Hz, 3H).

**$^{13}$C NMR** (101 MHz, $CDCl_3$) $\delta_C$ 170.3 (C), 147.3 (C), 141.6 (C), 130.4 (2CH), 123.9 (2CH), 61.5 ($CH_2$), 41.2 ($CH_2$), 14.3 ($CH_3$).

**HRMS** (ESI$^-$) m/z calcd. for $C_{10}H_{10}NO_4$ $(M - H)^-$ 208.0615, found 208.0625.

**Ethyl 2-(3-nitrophenyl)acetate (12)**

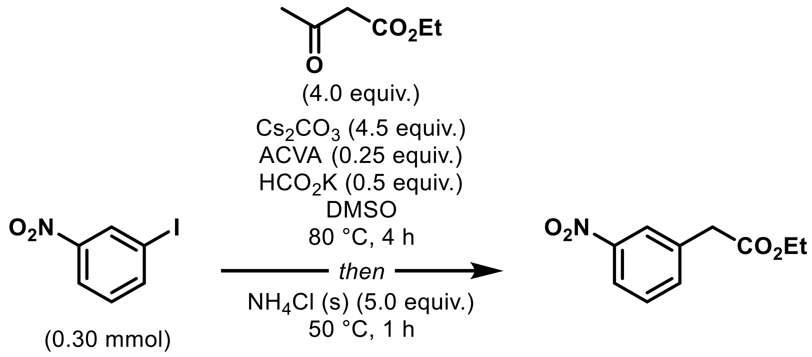


Synthesised according to **General Procedure A** with 1-iodo-3-nitrobenzene (74.7 mg, 0.300 mmol, 1.0 equiv.), ethyl acetoacetate (156 mg, 1.20 mmol, 4.0 equiv.), $Cs_2CO_3$ (440 mg, 1.35 mmol, 4.5 equiv.), ACVA (25.6 mg, 75 µmol, 0.25 equiv.) and $HCO_2K$ (12.6 mg, 150 µmol, 0.5 equiv.) in anhydrous DMSO (1.5 mL). The reaction mixture was heated at 80 °C for 4 hours. $NH_4Cl$ (80.3 mg, 1.50 mmol, 5.0 equiv.) was then added and the reaction mixture was stirred at 50 °C for 1 hour. The crude product was purified by column chromatography (5 → 10% $Et_3N$ in pentane) to afford the *title compound* **12** as a white solid (28.3 mg, 0.135 mmol, 45%).

**mp** 66–68 °C. $\boldsymbol{R_f}$ 0.58 (30% $Et_2O$ in pentane).

**$^1$H NMR** (500 MHz, $CDCl_3$) $\delta_H$ 8.17 (d, $J$ = 2.0 Hz, 1H), 8.14 (ddd, $J$ = 7.9, 2.0, 1.3 Hz, 1H), 7.63 (ddd, $J$ = 7.8, 1.4, 1.3 Hz, 1H), 7.51 (dd, $J$ = 7.9, 7.8 Hz, 1H), 4.18 (q, $J$ = 7.1 Hz, 2H), 3.73 (s, 2H), 1.27 (t, $J$ = 7.1 Hz, 3H).

**$^{13}$C NMR** (126 MHz, $CDCl_3$) $\delta_C$ 170.5 (C), 148.5 (C), 136.1 (C), 135.7 (CH), 129.6 (CH), 124.5 (CH), 122.4 (CH), 61.5 ($CH_2$), 40.9 ($CH_2$), 14.3 ($CH_3$).

**HRMS** (APCI$^+$) m/z calcd. for $C_{10}H_{12}NO_4$ $(M + H)^+$ 210.0761, found 210.0765.

**Ethyl 2-(2-nitrophenyl)acetate (13)**

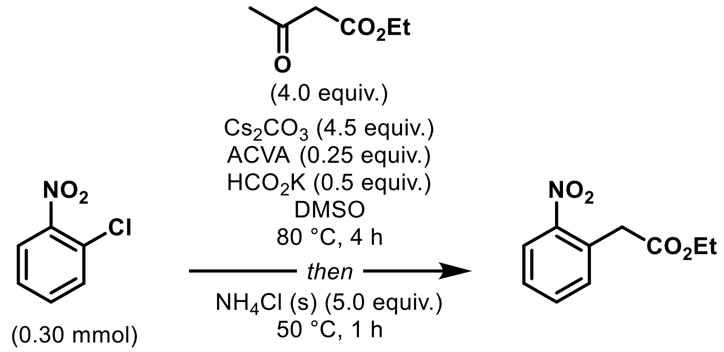


Synthesised according to **General Procedure A** with 1-chloro-2-nitrobenzene (47.3 mg, 0.300 mmol, 1.0 equiv.), ethyl acetoacetate (156 mg, 1.20 mmol, 4.0 equiv.), $Cs_2CO_3$ (440 mg, 1.35 mmol, 4.5 equiv.), ACVA (25.6 mg, 75 µmol, 0.25 equiv.) and $HCO_2K$ (12.6 mg, 150 µmol, 0.5 equiv.) in anhydrous DMSO (1.5 mL). The reaction mixture was heated at 80 °C for 4 hours. $NH_4Cl$ (80.3 mg, 1.50 mmol, 5.0 equiv.) was then added and the reaction mixture was stirred at 50 °C for 1 hour. The crude product was purified by column chromatography (5 → 10% $Et_3N$ in pentane) to afford the *title compound* **13** as a white solid (19.1 mg, 91 µmol, 30%).

**mp** 52–54 °C. ***R**f* 0.39 (30% $Et_2O$ in pentane).

**$^1$H NMR** (400 MHz, $CDCl_3$) $\delta_H$ 8.11 (dd, $J$ = 8.2, 1.1 Hz, 1H), 7.57 (ddd, $J$ = 7.5, 7.5, 1.1 Hz, 1H), 7.46 (dd, $J$ = 8.2, 1.1 Hz, 1H), 7.36 (d, $J$ = 7.5 Hz, 1H), 4.17 (q, $J$ = 7.1 Hz, 2H), 4.02 (s, 2H), 1.25 (t, $J$ = 7.1 Hz, 3H).

**$^{13}$C NMR** (101 MHz, $CDCl_3$) $\delta_C$ 170.0 (C), 149.0 (C), 133.7 (CH), 133.4 (CH), 130.0 (C), 128.7 (CH), 125.4 (CH), 61.4 ($CH_2$), 40.0 ($CH_2$), 14.2 ($CH_3$).

**HRMS** (ESI$^+$) m/z calcd. for $C_{10}H_{11}NNaO_4$ (M + Na)$^+$ 232.0586, found 232.0572.

**Ethyl 2-(4-acetylphenyl)acetate (14)**

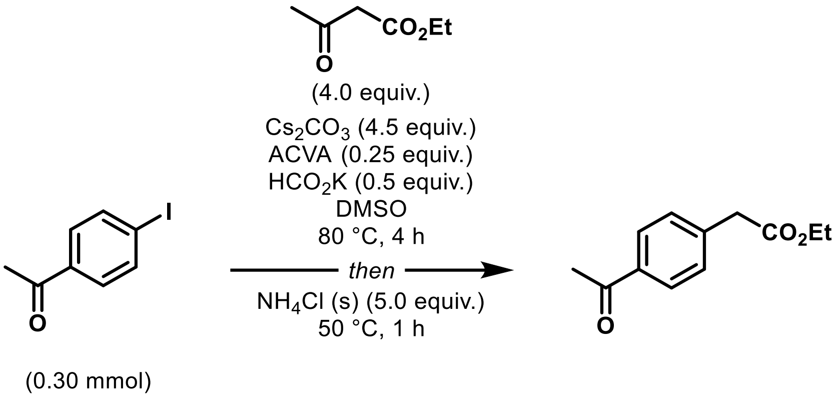


Synthesised according to **General Procedure A** with 4′-iodoacetophenone (73.8 mg, 0.300 mmol, 1.0 equiv.), ethyl acetoacetate (156 mg, 1.20 mmol, 4.0 equiv.), $Cs_2CO_3$ (440 mg, 1.35 mmol, 4.5 equiv.), ACVA (25.6 mg, 75 μmol, 0.25 equiv.) and $HCO_2K$ (12.6 mg, 150 μmol, 0.5 equiv.) in anhydrous DMSO (1.5 mL). The reaction mixture was heated at 80 °C for 4 hours. $NH_4Cl$ (80.3 mg, 1.50 mmol, 5.0 equiv.) was then added and the reaction mixture was stirred at 50 °C for 1 hour. The crude product was purified by column chromatography (10 → 35% EtOAc in pentane) to afford the *title compound* **14** as a white solid (33.6 mg, 0.163 mmol, 54%).

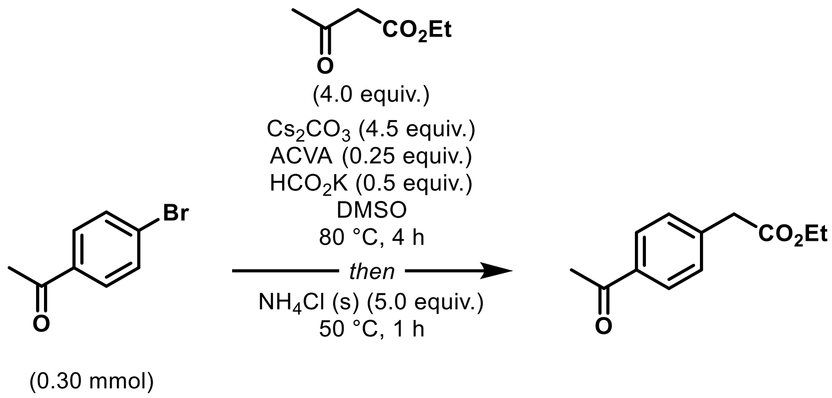


Synthesised according to General Procedure A with 4′-bromoacetophenone (59.7 mg, 0.300 mmol, 1.0 equiv.), ethyl acetoacetate (156 mg, 1.20 mmol, 4.0 equiv.), $Cs_2CO_3$ (440 mg, 1.35 mmol, 4.5 equiv.), ACVA (25.6 mg, 75 μmol, 0.25 equiv.) and $HCO_2K$ (12.6 mg, 150 μmol, 0.5 equiv.) in anhydrous DMSO (1.5 mL). The reaction mixture was heated at 80 °C for 4 hours. $NH_4Cl$ (80.3 mg, 1.50 mmol, 5.0 equiv.) was then added and the reaction mixture was stirred at 50 °C for 1 hour. The crude product was purified by column chromatography (10 → 30% EtOAc in pentane) to afford the *title compound* **14** as a white solid (32.5 mg, 0.158 mmol, 53%).

**mp** 54–55 °C. ***R_f*** 0.24 (10% EtOAc in pentane).

**$^1$H NMR** (400 MHz, $CDCl_3$) $\delta_H$ 7.92 (d, $J$ = 8.5 Hz, 2H), 7.38 (d, $J$ = 8.5 Hz, 2H), 4.16 (q, $J$ = 7.2 Hz, 2H), 3.67 (s, 2H), 2.59 (s, 3H), 1.25 (t, $J$ = 7.2 Hz, 3H).

**$^{13}$C NMR** (101 MHz, $CDCl_3$) $\delta_C$ 197.9 (C), 170.9 (C), 139.6 (C), 136.1 (C), 129.7 (2CH), 128.8 (2CH), 61.3 ($CH_2$), 41.5 ($CH_2$), 26.8 ($CH_3$), 14.3 ($CH_3$).

**HRMS** (APCI$^+$) m/z calcd. For $C_{12}H_{15}O_3$ $(M + H)^+$ 207.1016, found 207.1022.

**Ethyl 2-(4-benzoylphenyl)acetate (15)**

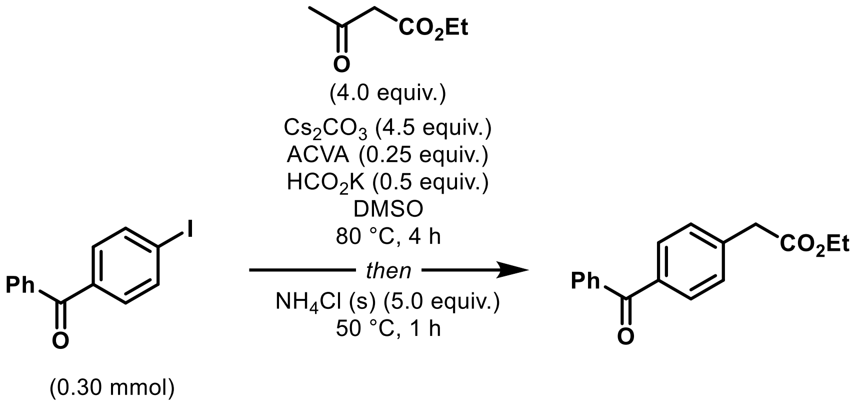


Synthesised according to **General Procedure A** with (4-iodophenyl)(phenyl)methanone (92.4 mg, 0.300 mmol, 1.0 equiv.), ethyl acetoacetate (156 mg, 1.20 mmol, 4.0 equiv.), $Cs_2CO_3$ (440 mg, 1.35 mmol, 4.5 equiv.), ACVA (25.6 mg, 75 μmol, 0.25 equiv.) and $HCO_2K$ (12.6 mg, 150 μmol, 0.5 equiv.) in anhydrous DMSO (1.5 mL). The reaction mixture was heated at 80 °C for 4 hours. $NH_4Cl$ (80.3 mg, 1.50 mmol, 5.0 equiv.) was then added and the reaction mixture was stirred at 50 °C for 1 hour. The crude product was purified by column chromatography (5 → 20% $Et_2O$ in pentane) to afford the *title compound* **15** as a white solid (46.4 mg, 0.173 mmol, 58%).

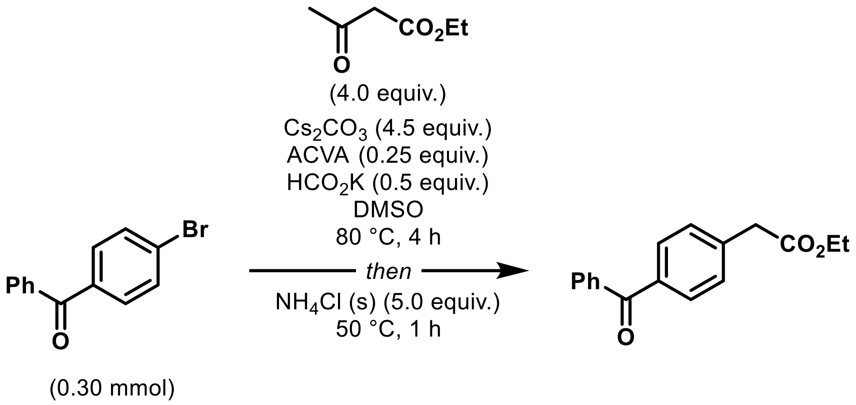


Synthesised according to **General Procedure A** with (4-bromophenyl)(phenyl)methanone (78.3 mg, 0.300 mmol, 1.0 equiv.), ethyl acetoacetate (156 mg, 1.20 mmol, 4.0 equiv.), $Cs_2CO_3$ (440 mg, 1.35 mmol, 4.5 equiv.), ACVA (25.6 mg, 75 μmol, 0.25 equiv.) and $HCO_2K$ (12.6 mg, 150 μmol, 0.5 equiv.) in anhydrous DMSO (1.5 mL). The reaction mixture was heated at 80 °C for 4 hours. $NH_4Cl$ (80.3 mg, 1.50 mmol, 5.0 equiv.) was then added and the reaction mixture was stirred at 50 °C for 1 hour. The crude product was purified by column chromatography (5 → 20% $Et_2O$ in pentane) to afford the *title compound* **15** as a white solid (63.9 mg, 0.238 mmol, 79%).

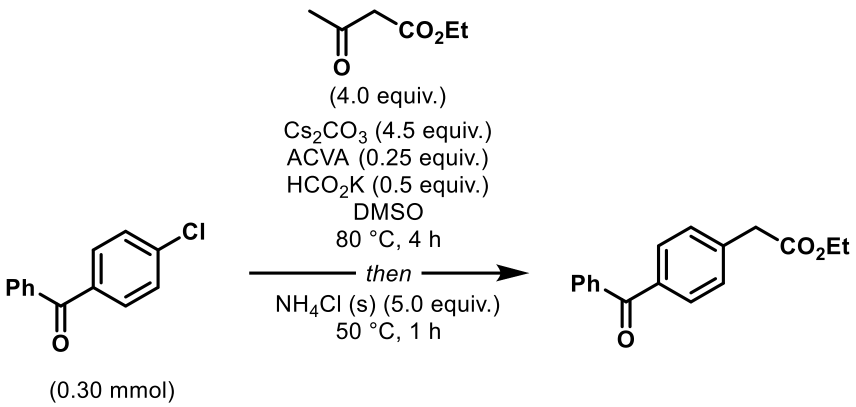


Synthesised according to **General Procedure A** with (4-chlorophenyl)(phenyl)methanone (65.0 mg, 0.300 mmol, 1.0 equiv.), ethyl acetoacetate (156 mg, 1.20 mmol, 4.0 equiv.), $Cs_2CO_3$ (440 mg, 1.35 mmol, 4.5 equiv.), ACVA (25.6 mg, 75 µmol, 0.25 equiv.) and $HCO_2K$ (12.6 mg, 150 µmol, 0.5 equiv.) in anhydrous DMSO (1.5 mL). The reaction mixture was heated at 80 °C for 4 hours. $NH_4Cl$ (80.3 mg, 1.50 mmol, 5.0 equiv.) was then added and the reaction mixture was stirred at 50 °C for 1 hour. The crude product was purified by column chromatography (5 → 10% $Et_3N$ in pentane) to afford the *title compound* **15** as a white solid (34.7 mg, 0.129 mmol, 43%).

**mp** 46–48 °C. $\boldsymbol{R_f}$ 0.27 (30% $Et_2O$ in pentane).

**$^1$H NMR** (500 MHz, $CDCl_3$) $\delta_H$ 7.83 – 7.74 (m, 4H), 7.58 (tt, $J$ = 7.5, 1.5, Hz, 1H), 7.48 (dd, $J$ = 7.5, 1.5 Hz, 2H), 7.41 (d, $J$ = 8.3 Hz, 2H), 4.18 (q, $J$ = 7.1 Hz, 2H), 3.70 (s, 2H), 1.27 (t, $J$ = 7.1 Hz, 3H).

**$^{13}$C NMR** (126 MHz, $CDCl_3$) $\delta_C$ 196.5 (C), 171.0 (C), 139.0 (C), 137.7 (C), 136.5 (C), 132.5 (CH), 130.5 (2CH), 130.1 (2CH), 129.4 (2CH), 128.4 (2CH), 61.3 ($CH_2$), 41.5 ($CH_2$), 14.3 ($CH_3$).

**HRMS** (APCI$^+$) m/z calcd. for $C_{17}H_{17}O_3$ $(M + H)^+$ 269.1172, found 269.1181.

**Ethyl 2-(1-oxo-2,3-dihydro-1*H*-inden-5-yl)acetate (16)**

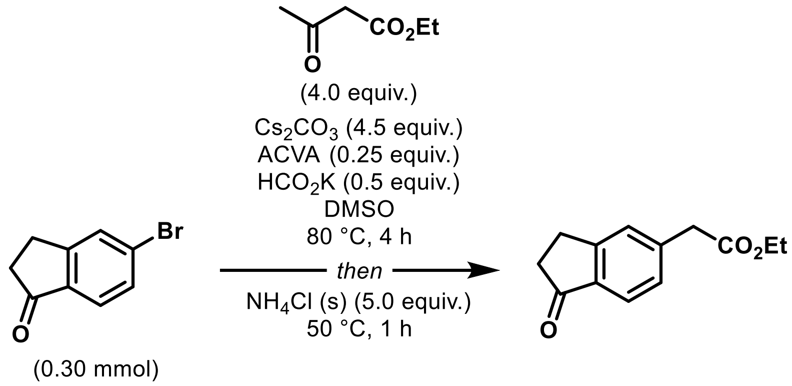


Synthesised according to **General Procedure A** with 5-bromo-2,3-dihydro-1*H*-inden-1-one (63.3 mg, 0.300 mmol, 1.0 equiv.), ethyl acetoacetate (156 mg, 1.20 mmol, 4.0 equiv.), $Cs_2CO_3$ (440 mg, 1.35 mmol, 4.5 equiv.), ACVA (25.6 mg, 75 µmol, 0.25 equiv.) and $HCO_2K$ (12.6 mg, 150 µmol, 0.5 equiv.) in anhydrous DMSO (1.5 mL). The reaction mixture was heated at 80 °C for 4 hours. $NH_4Cl$ (80.3 mg, 1.50 mmol, 5.0 equiv.) was then added and the reaction mixture was stirred at 50 °C for 1 hour. The crude product was purified by column chromatography (20 → 50% $Et_2O$ in pentane) to afford the *title compound* **16** as a yellow solid (21.5 mg, 98 µmol, 33%).

**mp** 36–38 °C. $\boldsymbol{R_f}$ 0.16 (30% $Et_2O$ in pentane).

**$^1$H NMR** (400 MHz, $CDCl_3$) $\delta_H$ 7.71 (d, $J$ = 7.9 Hz, 1H), 7.40 (s, 1H), 7.28 (d, $J$ = 7.9 Hz, 1H), 4.17 (q, $J$ = 7.1 Hz, 2H), 3.69 (s, 2H), 3.17 – 3.09 (m, 2H), 2.73 – 2.65 (m, 2H), 1.26 (t, $J$ = 7.1 Hz, 3H).

**$^{13}$C NMR** (101 MHz, $CDCl_3$) $\delta_C$ 206.7 (C), 171.0 (C), 155.8 (C), 141.3 (C), 136.3 (C), 128.9 (CH), 127.7 (CH), 124.0 (CH), 61.3 ($CH_2$), 41.8 ($CH_2$), 36.5 ($CH_2$), 25.8 ($CH_2$), 14.3 ($CH_3$).

**HRMS** (ESI$^+$) m/z calcd. for $C_{13}H_{14}NaO_3$ (M + Na)$^+$ 241.0841, found 241.0835.

**Methyl 4-(2-ethoxy-2-oxoethyl)benzoate (17)**

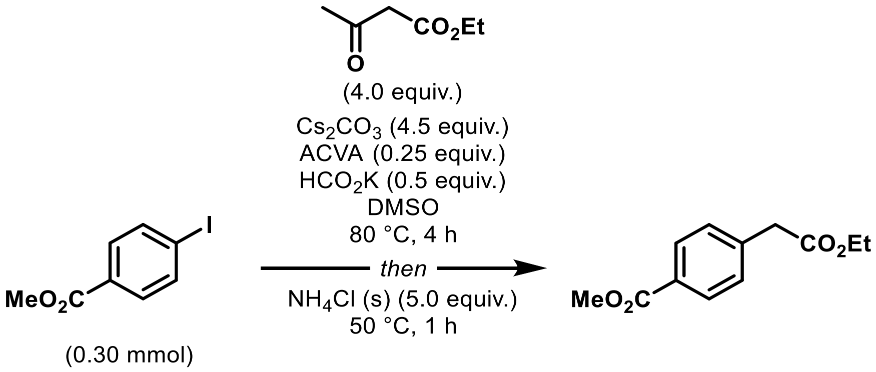


Synthesised according to **General Procedure A** with methyl 4-iodobenzoate (78.6 mg, 0.300 mmol, 1.0 equiv.), ethyl acetoacetate (156 mg, 1.20 mmol, 4.0 equiv.), $Cs_2CO_3$ (440 mg, 1.35 mmol, 4.5 equiv.), ACVA (25.6 mg, 75 µmol, 0.25 equiv.) and $HCO_2K$ (12.6 mg, 150 µmol, 0.5 equiv.) in anhydrous DMSO (1.5 mL). The reaction mixture was heated at 80 °C for 4 hours. $NH_4Cl$ (80.3 mg, 1.50 mmol, 5.0 equiv.) was then added and the reaction mixture was stirred at 50 °C for 1 hour. The crude product was purified by column chromatography (5 → 10% $Et_3N$ in pentane) to afford the *title compound* **17** as a colourless oil (41.8 mg, 0.188 mmol, 63%).

***$R_f$*** 0.50 (30% $Et_2O$ in pentane).

**$^1$H NMR** (400 MHz, $CDCl_3$) $\delta_H$ 7.99 (d, $J$ = 8.3 Hz, 2H), 7.35 (d, $J$ = 8.3 Hz, 2H), 4.15 (q, $J$ = 7.1 Hz, 2H), 3.90 (s, 3H), 3.66 (s, 2H), 1.24 (t, $J$ = 7.1 Hz, 3H).

**$^{13}$C NMR** (101 MHz, $CDCl_3$) $\delta_C$ 171.0 (C), 167.0 (C), 139.4 (C), 130.0 (2CH), 129.5 (2CH), 129.1 (C), 61.2 ($CH_2$), 52.2 ($CH_3$), 41.5 ($CH_2$), 14.3 ($CH_3$).

**HRMS** ($ESI^+$) m/z calcd. for $C_{12}H_{14}NaO_4$ $(M + Na)^+$ 245.0790, found 245.0772.

**Ethyl 4-(2-ethoxy-2-oxoethyl)benzoate (18)**

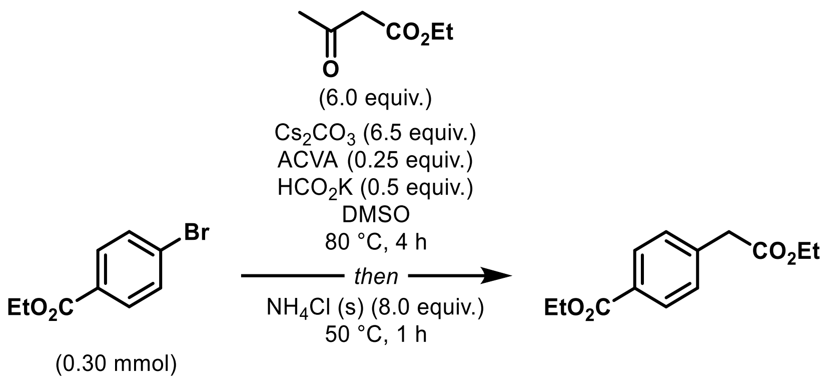


Synthesised according to **General Procedure A** with ethyl 4-bromobenzoate (68.7 mg, 0.300 mmol, 1.0 equiv.), ethyl acetoacetate (234 mg, 1.80 mmol, 6.0 equiv.), $Cs_2CO_3$ (635 mg, 1.95 mmol, 6.5 equiv.), ACVA (25.6 mg, 75 μmol, 0.25 equiv.) and $HCO_2K$ (12.6 mg, 150 μmol, 0.5 equiv.) in anhydrous DMSO (1.5 mL). The reaction mixture was heated at 80 °C for 4 hours. $NH_4Cl$ (128 mg, 2.40 mmol, 8.0 equiv.) was then added and the reaction mixture was stirred at 50 °C for 1 hour. The crude product was purified by column chromatography (5 → 15% EtOAc in pentane) to afford the *title compound* **18** as a colourless oil (24.0 mg, 0.102 mmol, 34%).

***R**$_f$* 0.48 (10% EtOAc in pentane).

**$^1$H NMR** (400 MHz, $CDCl_3$) $\delta_H$ 8.01 (d, *J* = 8.4 Hz, 2H), 7.35 (d, *J* = 8.4 Hz, 2H), 4.37 (q, *J* = 7.1 Hz, 2H), 4.15 (q, *J* = 7.1 Hz, 2H), 3.66 (s, 2H), 1.38 (t, *J* = 7.1 Hz, 3H), 1.24 (t, *J* = 7.1 Hz, 3H).

**$^{13}$C NMR** (101 MHz, $CDCl_3$) $\delta_C$ 171.01 (C), 166.5 (C), 139.3 (C), 129.9 (2CH), 129.5 (C), 129.4 (2CH), 61.2 ($CH_2$), 61.1 ($CH_2$), 41.5 ($CH_2$), 14.5 ($CH_3$), 14.3 ($CH_3$).

**HRMS** (ESI$^+$) m/z calcd. for $C_{13}H_{16}NaO_4$ $(M + Na)^+$ 259.0946, found 259.0951.

**Ethyl 2-(4-carbamoyl-2-nitrophenyl)acetate (19)**

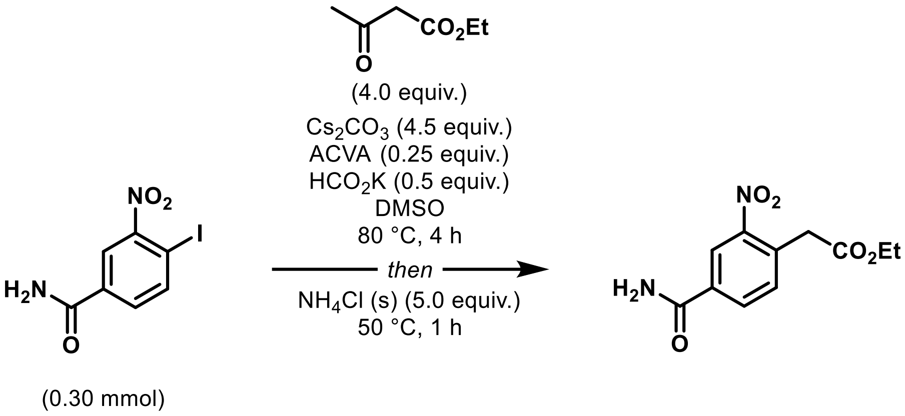


Synthesised according to **General Procedure A** with 4-iodo-3-nitrobenzamide (87.6 mg, 0.300 mmol, 1.0 equiv.), ethyl acetoacetate (156 mg, 1.20 mmol, 4.0 equiv.), $Cs_2CO_3$ (440 mg, 1.35 mmol, 4.5 equiv.), ACVA (25.6 mg, 75 µmol, 0.25 equiv.) and $HCO_2K$ (12.6 mg, 150 µmol, 0.5 equiv.) in anhydrous DMSO (1.5 mL). The reaction mixture was heated at 80 °C for 4 hours. $NH_4Cl$ (80.3 mg, 1.50 mmol, 5.0 equiv.) was then added and the reaction mixture was stirred at 50 °C for 1 hour. The crude product was purified by column chromatography (25 → 40% acetone in pentane) to afford the *title compound* **19** as a white solid (40.3 mg, 0.160 mmol, 53%).

**mp** 120–122 °C. ***R<sub></sub>f*** 0.36 (40% acetone in pentane)

**$^1$H NMR** (400 MHz, MeOD) $\delta_H$ 8.62 (d, *J* = 1.9 Hz, 1H), 8.15 (dd, *J* = 8.0, 1.9 Hz, 1H), 7.60 (d, *J* = 8.0 Hz, 1H), 4.16 (d, *J* = 7.1 Hz, 2H), 4.13 (s, 2H), 1.24 (t, *J* = 7.1 Hz, 3H).

**$^{13}$C NMR** (126 MHz, MeOD) $\delta_C$ 171.5 (C), 169.4 (C), 150.2 (C), 135.8 (C), 135.1 (CH), 134.7, 133.3 (CH), 125.3 (CH), 62.3 ($CH_2$), 40.3 ($CH_2$), 14.4 ($CH_3$).

**HRMS** ($ESI^-$) m/z calcd. for $C_{11}H_{11}N_2O_5$ $(M - H)^-$ 251.0673, found 251.0670.

**Ethyl 2-(4-acetyl-3-fluorophenyl)acetate (20)**

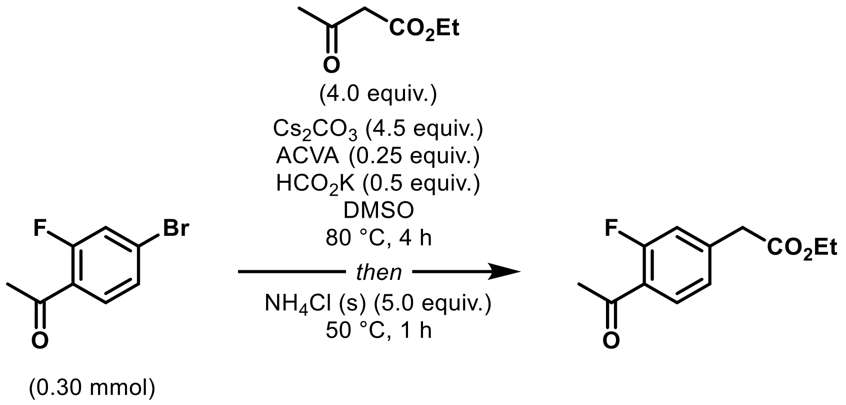


Synthesised according to **General Procedure A** with 1-(4-bromo-2-fluorophenyl)ethan-1-one (65.1 mg, 0.300 mmol, 1.0 equiv.), ethyl acetoacetate (156 mg, 1.20 mmol, 4.0 equiv.), $Cs_2CO_3$ (440 mg, 1.35 mmol, 4.5 equiv.), ACVA (25.6 mg, 75 μmol, 0.25 equiv.) and $HCO_2K$ (12.6 mg, 150 μmol, 0.5 equiv.) in anhydrous DMSO (1.5 mL). The reaction mixture was heated at 80 °C for 4 hours. $NH_4Cl$ (80.3 mg, 1.50 mmol, 5.0 equiv.) was then added and the reaction mixture was stirred at 50 °C for 1 hour. The crude product was purified by column chromatography (5 → 10% $Et_2O$, 5% $Et_3N$ in pentane) to afford the *title compound* **20** as a pale-yellow solid (50.0 mg, 0.223 mmol, 74%).

**mp** 50–51 °C. $\boldsymbol{R_f}$ 0.78 (10% $Et_2O$, 10% $Et_3N$ in pentane).

**$^1$H NMR** (400 MHz, $CDCl_3$) $\delta_H$ 7.83 (dd, $J$ = 7.8, 7.7 Hz, 1H), 7.13 (dd, $J$ = 7.8, 1.6 Hz, 1H), 7.09 (dd, $J$ = 11.7, 1.6 Hz, 1H), 4.16 (q, $J$ = 7.1 Hz, 2H), 3.64 (s, 2H), 2.62 (d, $J$ = 4.9 Hz, 3H), 1.26 (t, $J$ = 7.1 Hz, 3H).

**$^{13}$C NMR** (101 MHz, $CDCl_3$) $\delta_C$ 195.6 (d, $J$ = 3.5 Hz, C), 170.4 (C), 162.27 (d, $J$ = 255.2 Hz, C), 141.8 (C), 130.9 (d, $J$ = 2.9 Hz, CH), 125.6 (d, $J$ = 3.2 Hz, CH), 124.5 (d, $J$ = 12.8 Hz, C), 117.7 (d, $J$ = 24.7 Hz, CH), 61.4 ($CH_2$), 41.1 ($CH_2$), 31.5 (d, $J$ = 7.6 Hz, $CH_3$), 14.3 ($CH_3$).

**$^{19}$F NMR** (376 MHz, $CDCl_3$) $\delta_F$ −109.12 – −109.25 (m, F).

**HRMS** (ESI$^+$) m/z calcd. for $C_{12}H_{13}FNaO_3$ $(M + Na)^+$ 247.0746, found 247.0751.

**Ethyl 2-(3,5-bis(trifluoromethyl)phenyl)acetate (21)**

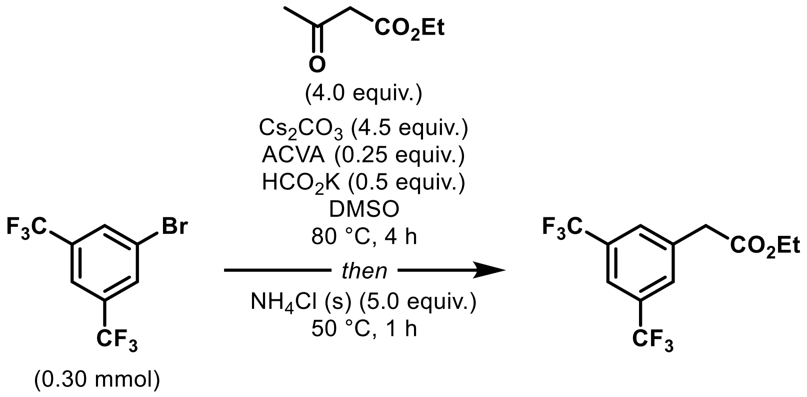


Synthesised according to **General Procedure A** with 1-bromo-3,5-bis(trifluoromethyl)benzene (78.3 mg, 0.300 mmol, 1.0 equiv.), ethyl acetoacetate (156 mg, 1.20 mmol, 4.0 equiv.), $Cs_2CO_3$ (440 mg, 1.35 mmol, 4.5 equiv.), ACVA (25.6 mg, 75 µmol, 0.25 equiv.) and $HCO_2K$ (12.6 mg, 150 µmol, 0.5 equiv.) in anhydrous DMSO (1.5 mL). The reaction mixture was heated at 80 °C for 4 hours. $NH_4Cl$ (80.3 mg, 1.50 mmol, 5.0 equiv.) was then added and the reaction mixture was stirred at 50 °C for 1 hour. The crude product was purified by column chromatography (5% $Et_3N$ in pentane) to afford the *title compound* **21** as an amorphous white solid (60.2 mg, 0.200 mmol, 67%).

***R**$_f$* 0.84 (10% $Et_2O$ in pentane).

**$^1$H NMR** (400 MHz, $CDCl_3$) $\delta_H$ 7.80 (s, 1H), 7.76 (s, 2H), 4.19 (q, *J* = 7.1 Hz, 2H), 3.75 (s, 2H), 1.27 (t, *J* = 7.1 Hz, 3H).

**$^{13}$C NMR** (101 MHz, $CDCl_3$) $\delta_C$ 170.1 (C), 136.5 (C), 131.9 (q, *J* = 33.4 Hz, 2C), 129.8 (q, *J* = 2.7 Hz, 2CH), 123.4 (q, *J* = 272.7 Hz, 2C), 121.4 (h, *J* = 3.7 Hz, CH), 61.6 ($CH_2$), 40.9 ($CH_2$), 14.2 ($CH_3$).

**$^{19}$F NMR** (376 MHz, $CDCl_3$) $\delta_F$ –62.9 (s, 6F).

**HRMS** (ESI$^-$) m/z calcd. for $C_{12}H_9F_6O_2$ $(M - H)^-$ 299.0512, found 299.0507.

**Ethyl 2-(2-fluoro-[1,1'-biphenyl]-4-yl)acetate (22)**

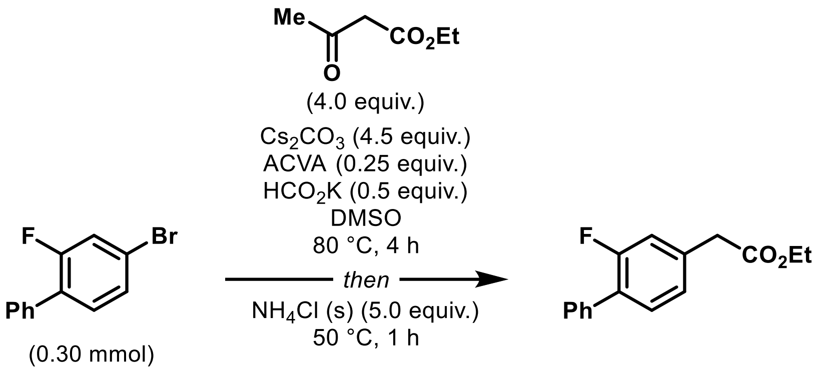


Synthesised according to **General Procedure A** with 4-bromo-2-fluoro-1,1'-biphenyl (75.3 mg, 0.300 mmol, 1.0 equiv.), ethyl acetoacetate (156 mg, 1.20 mmol, 4.0 equiv.), $Cs_2CO_3$ (440 mg, 1.35 mmol, 4.5 equiv.), ACVA (25.6 mg, 75 µmol, 0.25 equiv.) and $HCO_2K$ (12.6 mg, 150 µmol, 0.5 equiv.) in anhydrous DMSO (1.5 mL). The reaction mixture was heated at 80 °C for 4 hours. $NH_4Cl$ (80.3 mg, 1.50 mmol, 5.0 equiv.) was then added and the reaction mixture was stirred at 50 °C for 1 hour. The crude product was purified by column chromatography (5 → 10% $Et_2O$ in pentane) to afford the *title compound* **22** as an amorphous white solid (12.0 mg, 46 µmol, 15%).

$\boldsymbol{R_f}$ 0.45 (10% $Et_2O$ in pentane).

**$^1$H NMR** (400 MHz, $CDCl_3$) $\delta_H$ 7.54 (ddd, $J$ = 8.1, 1.5, 1.5 Hz, 2H), 7.49 – 7.32 (m, 4H), 7.16 – 7.09 (m, 2H), 4.19 (q, $J$ = 7.1 Hz, 2H), 3.65 (s, 2H), 1.29 (t, $J$ = 7.1 Hz, 3H).

**$^{13}$C NMR** (101 MHz, $CDCl_3$) $\delta_C$ 171.2 (C), 159.7 (d, $J$ = 248.3 Hz, C), 135.7 (C), 135.5 (d, $J$ = 8.3 Hz, C), 130.9 (d, $J$ = 4.0 Hz, CH), 129.1 (d, $J$ = 3.2 Hz, 2CH), 128.6 (2CH), 127.9 (d, $J$ = 13.6 Hz, C), 127.8 (CH), 125.4 (d, $J$ = 3.3 Hz, CH), 117.1 (d, $J$ = 23.6 Hz, CH), 61.3 ($CH_2$), 40.9 ($CH_2$), 14.3 ($CH_3$).

**$^{19}$F NMR** (376 MHz, $CDCl_3$) $\delta_F$ −117.90 – −118.13 (m, F).

**HRMS** (ESI$^+$) m/z calcd. for $C_{16}H_{15}FNaO_2$ $(M + Na)^+$ 281.0948, found 281.0942.

**Dehalogenation of 4-iodoanisole to anisole (23)**

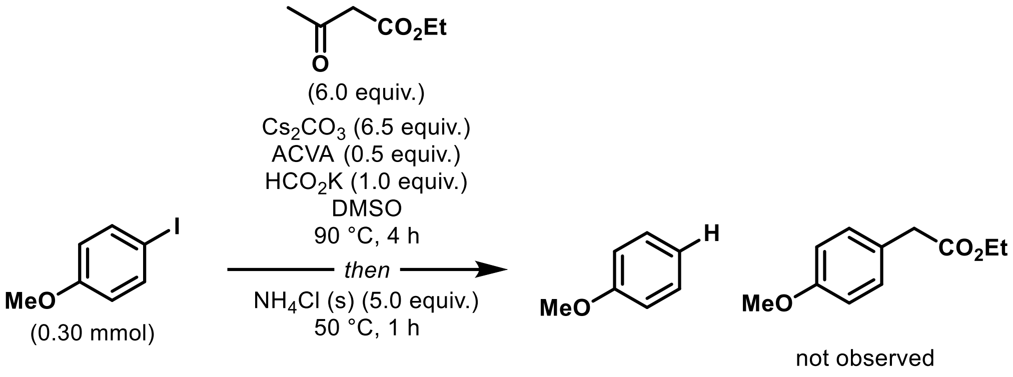


*Note:* More forcing conditions (e.g. additional nucleophile, ACVA and formate at higher temperatures) were employed in an attempt to substitute the electron-rich aryl halide, but no product formation was observed.

Synthesis attempted according to **General Procedure A** with 4-iodoanisole (70.2 mg, 0.300 mmol, 1.0 equiv.), ethyl acetoacetate (234 mg, 1.80 mmol, 6.0 equiv.), $Cs_2CO_3$ (635 mg, 1.95 mmol, 6.5 equiv.), ACVA (51.3 mg, 0.150 mmol, 0.5 equiv.) and $HCO_2K$ (12.6 mg, 0.300 mmol, 1.0 equiv.) in anhydrous DMSO (1.5 mL). The reaction mixture was heated at 90 °C for 4 hours. $NH_4Cl$ (80.3 mg, 1.50 mmol, 5.0 equiv.) was then added and the reaction mixture was stirred at 50 °C for 1 hour. 1,3,5-Trimethoxybenzene (50.5 mg, 0.300 mmol, 1.0 equiv.) was added to the crude reaction mixture, which was then analysed by $^1H$ NMR spectroscopy.

**Ethyl 2-(6-(trifluoromethyl)pyridin-3-yl)acetate (24)**

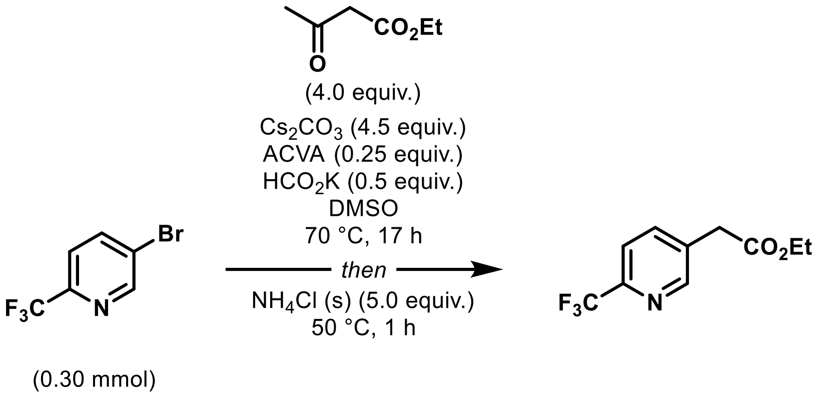


Synthesised according to **General Procedure A** with 5-bromo-2-(trifluoromethyl)pyridine (67.8 mg, 0.300 mmol, 1.0 equiv.), ethyl acetoacetate (156 mg, 1.20 mmol, 4.0 equiv.), $Cs_2CO_3$ (440 mg, 1.35 mmol, 4.5 equiv.), ACVA (25.6 mg, 75 µmol, 0.25 equiv.) and $HCO_2K$ (12.6 mg, 150 µmol, 0.5 equiv.) in anhydrous DMSO (1.5 mL). The reaction mixture was heated at 70 °C for 17 hours. $NH_4Cl$ (80.3 mg, 1.50 mmol, 5.0 equiv.) was then added and the reaction mixture was stirred at 50 °C for 1 hour. The crude product was purified by column chromatography (5% $Et_3N$ in pentane) to afford the *title compound* **24** as a yellow oil (37.5 mg, 0.161 mmol, 54%).

***R$_f$*** 0.29 (5% $Et_3N$ in pentane)

**$^1$H NMR** (400 MHz, $CDCl_3$) $\delta_H$ 8.64 (d, *J* = 2.1 Hz, 1H), 7.84 (dd, *J* = 8.0, 2.1 Hz, 1H), 7.66 (d, *J* = 8.0 Hz, 1H), 4.18 (q, *J* = 7.1 Hz, 2H), 3.71 (s, 2H), 1.27 (t, *J* = 7.1 Hz, 3H).

**$^{13}$C NMR** (126 MHz, $CDCl_3$) $\delta_C$ 170.0 (C), 150.7 (CH), 147.2 (q, *J* = 34.8 Hz, C), 138.3 (CH), 133.2 (C), 121.7 (q, *J* = 274.0 Hz, C), 120.4 (q, *J* = 2.6 Hz, CH), 61.7 ($CH_2$), 38.3 ($CH_2$), 14.2 ($CH_3$).

**$^{19}$F NMR** (376 MHz, $CDCl_3$) $\delta_F$ −67.9 (s, 3F)

**HRMS** ($ESI^+$) m/z calcd. for $C_{10}H_{10}F_3NNaO_2$ $(M + Na)^+$ 256.0556, found 256.0548.

**Ethyl 2-(6-acetylpyridin-3-yl)acetate (25)**

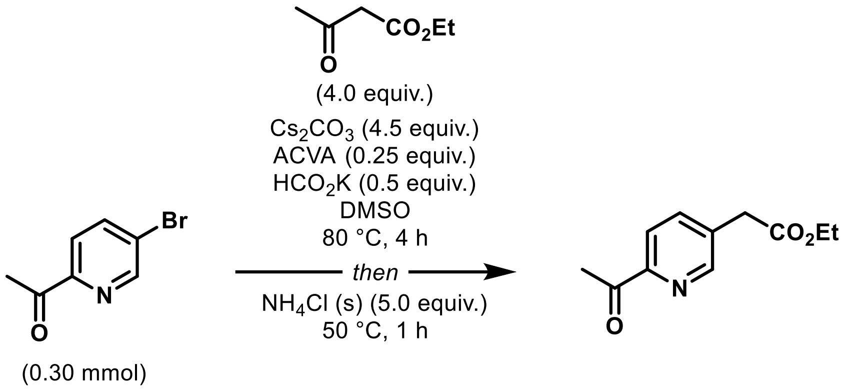


Synthesised according to **General Procedure A** with 1-(5-bromopyridin-2-yl)ethan-1-one (60.0 mg, 0.300 mmol, 1.0 equiv.), ethyl acetoacetate (156 mg, 1.20 mmol, 4.0 equiv.), $Cs_2CO_3$ (440 mg, 1.35 mmol, 4.5 equiv.), ACVA (25.6 mg, 75 µmol, 0.25 equiv.) and $HCO_2K$ (12.6 mg, 150 µmol, 0.5 equiv.) in anhydrous DMSO (1.5 mL). The reaction mixture was heated at 80 °C for 4 hours. $NH_4Cl$ (80.3 mg, 1.50 mmol, 5.0 equiv.) was then added and the reaction mixture was stirred at 50 °C for 1 hour. The crude product was purified by column chromatography (10% $Et_3N$ in pentane) to afford the *title compound* **25** as a white solid (25.5 mg, 0.123 mmol, 41%).

**mp** 42–44 °C. $\boldsymbol{R_f}$ 0.41 (10% $Et_3N$ in pentane)

**$^1$H NMR** (400 MHz, $CDCl_3$) $\delta_H$ 8.58 (dd, $J$ = 2.1, 0.8 Hz, 1H), 8.02 (dd, $J$ = 8.1, 0.8 Hz, 1H), 7.77 (dd, $J$ = 8.1, 2.1 Hz, 1H), 4.18 (q, $J$ = 7.1 Hz, 2H), 3.70 (s, 2H), 2.72 (s, 3H), 1.26 (t, $J$ = 7.1 Hz, 3H).

**$^{13}$C NMR** (101 MHz, $CDCl_3$) $\delta_C$ 199.9 (C), 170.2 (C), 152.6 (C), 149.7 (CH), 137.8 (CH), 133.9 (C), 121.7 (CH), 61.6 ($CH_2$), 38.7 ($CH_2$), 26.0 ($CH_3$), 14.3 ($CH_3$).

**HRMS** ($ESI^+$) m/z calcd. for $C_{11}H_{13}NNaO_3$ $(M + Na)^+$ 230.0788, found 230.0782.

**Ethyl 2-(6-cyanopyridin-3-yl)acetate (26)**

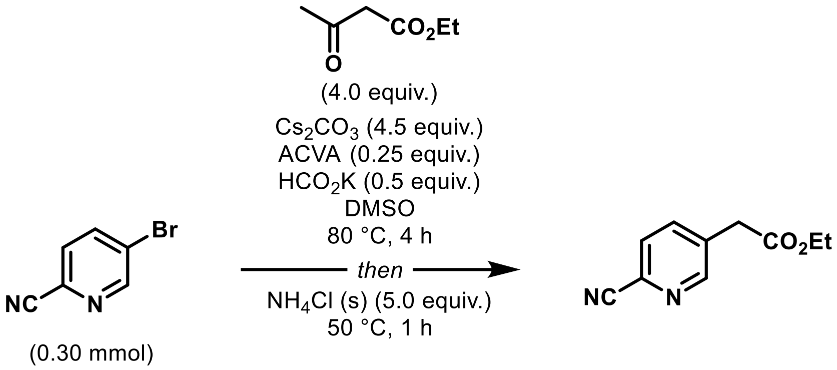

Synthesised according to **General Procedure A** with 5-bromopicolinonitrile (54.9 mg, 0.300 mmol, 1.0 equiv.), ethyl acetoacetate (156 mg, 1.20 mmol, 4.0 equiv.), $Cs_2CO_3$ (440 mg, 1.35 mmol, 4.5 equiv.), ACVA (25.6 mg, 75 µmol, 0.25 equiv.) and $HCO_2K$ (12.6 mg, 150 µmol, 0.5 equiv.) in anhydrous DMSO (1.5 mL). The reaction mixture was heated at 80 °C for 4 hours. $NH_4Cl$ (80.3 mg, 1.50 mmol, 5.0 equiv.) was then added and the reaction mixture was stirred at 50 °C for 1 hour. The crude product was purified by column chromatography (15 → 50% EtOAc in pentane) to afford the *title compound* **26** as a yellow oil (37.0 mg, 0.195 mmol, 64%).

***Rf*** 0.27 (20% EtOAc in pentane).

**$^1$H NMR** (400 MHz, $CDCl_3$) $\delta_H$ 8.62 (d, $J$ = 2.2 Hz, 1H), 7.80 (dd, $J$ = 8.0, 2.2 Hz, 1H), 7.67 (d, $J$ = 8.0 Hz, 1H), 4.18 (q, $J$ = 7.2 Hz, 2H), 3.70 (s, 2H), 1.27 (t, $J$ = 7.2 Hz, 3H).

**$^{13}$C NMR** (101 MHz, $CDCl_3$) $\delta_C$ 169.6 (C), 152.0 (C), 138.0 (CH), 134.0 (CH), 132.7 (CH), 128.3 (C), 117.2 (C), 61.8 ($CH_2$), 38.5 ($CH_2$), 14.2 ($CH_3$).

**HRMS** (ESI$^+$) m/z calcd. for $C_{10}H_{11}N_2O_2$ $(M + H)^+$ 191.0815, found 191.0808.

**Ethyl 2-(6-nitropyridin-3-yl)acetate (27)**

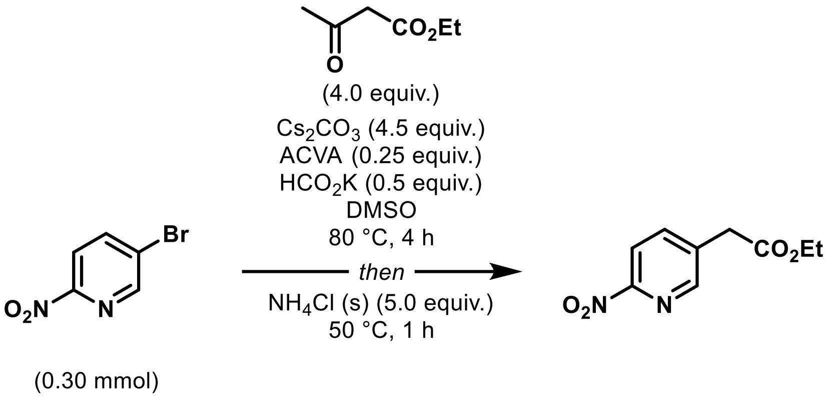


Synthesised according to **General Procedure A** with 5-bromo-2-nitropyridine (60.9 mg, 0.300 mmol, 1.0 equiv.), ethyl acetoacetate (156 mg, 1.20 mmol, 4.0 equiv.), $Cs_2CO_3$ (440 mg, 1.35 mmol, 4.5 equiv.), ACVA (25.6 mg, 75 µmol, 0.25 equiv.) and $HCO_2K$ (12.6 mg, 150 µmol, 0.5 equiv.) in anhydrous DMSO (1.5 mL). The reaction mixture was heated at 80 °C for 4 hours. $NH_4Cl$ (80.3 mg, 1.50 mmol, 5.0 equiv.) was then added and the reaction mixture was stirred at 50 °C for 1 hour. The crude product was purified by column chromatography (30% $Et_2O$ in pentane) to afford the *title compound* **27** as a yellow oil (17.1 mg, 81 µmol, 27%).

$\boldsymbol{R_f}$ 0.20 (30% $Et_2O$ in pentane)

**$^1$H NMR** (400 MHz, $CDCl_3$) $\delta_H$ 8.54 (d, $J$ = 2.2 Hz, 1H), 8.24 (d, $J$ = 8.3 Hz, 1H), 8.00 (dd, $J$ = 8.3, 2.2 Hz, 1H), 4.20 (q, $J$ = 7.1 Hz, 2H), 3.78 (s, 2H), 1.27 (t, $J$ = 7.1 Hz, 3H).

**$^{13}$C NMR** (126 MHz, $CDCl_3$) $\delta_C$ 169.5 (C), 156.0 (C), 149.6 (CH), 140.8 (CH), 136.5 (C), 118.0 (CH), 61.9 ($CH_2$), 38.1 ($CH_2$), 14.3 ($CH_3$).

**HRMS** ($ESI^+$) m/z calcd. for $C_9H_{11}N_2O_4$ $(M + H)^+$ 211.0713, found 211.0715.

**Ethyl 2-(6'-methyl-3-(4-(methylsulfonyl)phenyl)-[2,3'-bipyridin]-5-yl)acetate (28)**

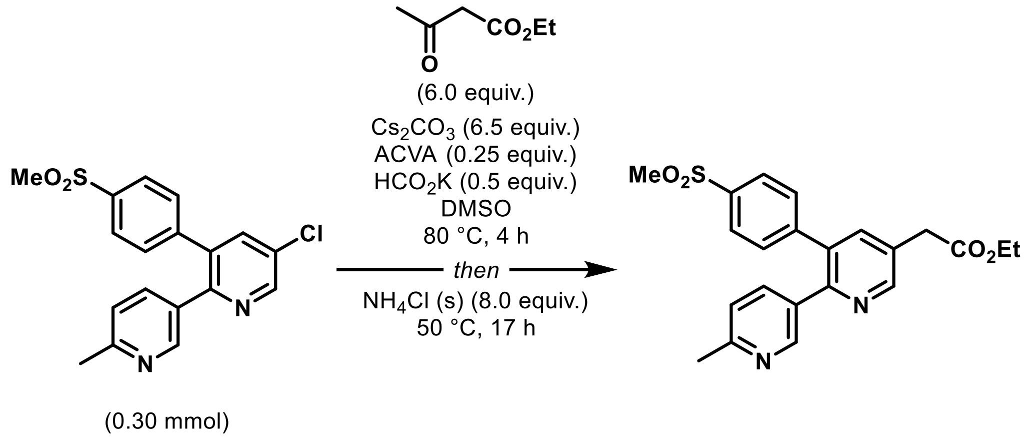


Synthesised according to **General Procedure A** with 5-chloro-6'-methyl-3-(4-(methylsulfonyl)phenyl)-2,3'-bipyridine (108 mg, 0.300 mmol, 1.0 equiv.), ethyl acetoacetate (234 mg, 1.80 mmol, 6.0 equiv.), $Cs_2CO_3$ (635 mg, 1.95 mmol, 6.5 equiv.), ACVA (25.6 mg, 75 µmol, 0.25 equiv.) and $HCO_2K$ (12.6 mg, 150 µmol, 0.5 equiv.) in anhydrous DMSO (1.5 mL). The reaction mixture was heated at 80 °C for 4 hours. $NH_4Cl$ (128 mg, 2.40 mmol, 8.0 equiv.) was then added and the reaction mixture was stirred at 50 °C for 17 hours. The crude product was purified by column chromatography (20 → 25% acetone in $CH_2Cl_2$) to afford the *title compound* **28** as a yellow solid (77.3 mg, 0.188 mmol, 63%).

**mp** 124–126 °C. ***R**f* 0.28 (30% acetone in pentane).

**$^{1}$H NMR** (400 MHz, $CDCl_3$) $\delta_H$ 8.66 (d, *J* = 2.2 Hz, 1H), 8.40 (d, *J* = 2.3 Hz, 1H), 7.88 (d, *J* = 8.4 Hz, 2H), 7.69 (d, *J* = 2.2 Hz, 1H), 7.58 (dd, *J* = 8.0, 2.3 Hz, 1H), 7.40 (d, *J* = 8.4 Hz, 2H), 7.08 (d, *J* = 8.0 Hz, 1H), 4.21 (q, *J* = 7.1 Hz, 2H), 3.73 (s, 2H), 3.08 (s, 3H), 2.53 (s, 3H), 1.29 (t, *J* = 7.1 Hz, 3H).

**$^{13}$C NMR** (101 MHz, $CDCl_3$) $\delta_C$ 170.6 (C), 158.3 (C), 153.2 (C), 150.3 (CH), 150.1 (CH), 145.1 (C), 139.8 (C), 139.6 (CH), 137.5 (CH), 134.3 (C), 132.1 (C), 130.6 (2CH), 129.1 (C), 127.9 (2CH), 122.9 (CH), 61.6 ($CH_2$), 44.7 ($CH_3$), 38.0 ($CH_2$), 24.4 ($CH_3$), 14.3 ($CH_3$).

**HRMS** ($ESI^+$) m/z calcd. for $C_{22}H_{22}N_2NaO_4S$ $(M + Na)^+$ 433.1198, found 433.1214.

**Methyl 5-(2-ethoxy-2-oxoethyl)nicotinate (29)**

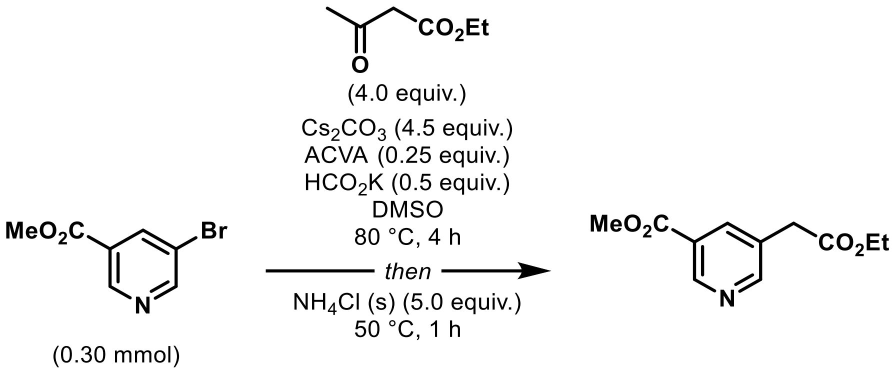


Synthesised according to **General Procedure A** with methyl 5-bromonicotinate (64.8 mg, 0.300 mmol, 1.0 equiv.), ethyl acetoacetate (156 mg, 1.20 mmol, 4.0 equiv.), $Cs_2CO_3$ (440 mg, 1.35 mmol, 4.5 equiv.), ACVA (25.6 mg, 75 µmol, 0.25 equiv.) and $HCO_2K$ (12.6 mg, 150 µmol, 0.5 equiv.) in anhydrous DMSO (1.5 mL). The reaction mixture was heated at 80 °C for 4 hours. $NH_4Cl$ (80.3 mg, 1.50 mmol, 5.0 equiv.) was then added and the reaction mixture was stirred at 50 °C for 1 hour. The crude product was purified by column chromatography (40 → 60% $Et_2O$ in pentane) to afford the *title compound* **29** as a white solid (35.4 mg, 0.159 mmol, 53%).

**mp** 46–47 °C. $\boldsymbol{R_f}$ 0.31 (40% $Et_2O$ in pentane).

**$^1$H NMR** (400 MHz, $CDCl_3$) $\delta_H$ 9.12 (d, $J$ = 2.1 Hz, 1H), 8.68 (d, $J$ = 2.2 Hz, 1H), 8.24 (dd, $J$ = 2.2, 2.1 Hz, 1H), 4.17 (q, $J$ = 7.1 Hz, 2H), 3.94 (s, 3H), 3.68 (s, 2H), 1.26 (t, $J$ = 7.1 Hz, 3H).

**$^{13}$C NMR** (101 MHz, $CDCl_3$) $\delta_C$ 170.3 (C), 165.7 (C), 154.2 (CH), 149.7 (CH), 138.0 (CH), 129.9 (C), 126.0 (C), 61.6 ($CH_2$), 52.6 ($CH_2$), 38.3 ($CH_3$), 14.3 ($CH_3$).

**HRMS** ($ESI^+$) m/z calcd. for $C_{11}H_{14}NO_4$ $(M + H)^+$ 224.0917, found 224.0923.

**Ethyl 2-(5-cyanopyridin-2-yl)acetate (30)**

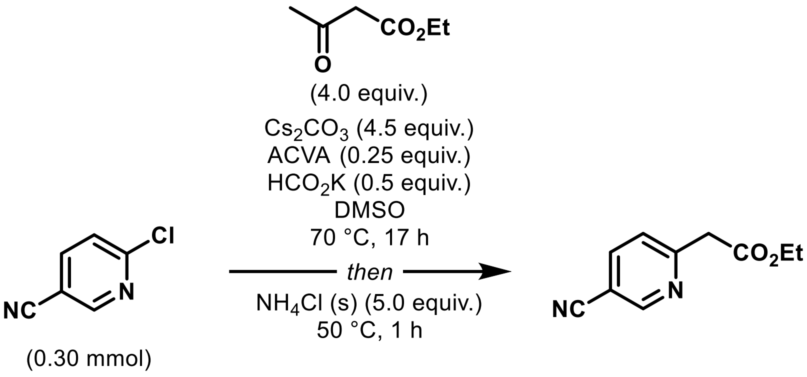


Synthesised according to **General Procedure A** with 6-chloronicotinonitrile (41.6 mg, 0.300 mmol, 1.0 equiv.), ethyl acetoacetate (156 mg, 1.20 mmol, 4.0 equiv.), $Cs_2CO_3$ (440 mg, 1.35 mmol, 4.5 equiv.), ACVA (25.6 mg, 75 µmol, 0.25 equiv.) and $HCO_2K$ (12.6 mg, 150 µmol, 0.5 equiv.) in anhydrous DMSO (1.5 mL). The reaction mixture was heated at 70 °C for 17 hours. $NH_4Cl$ (80.3 mg, 1.50 mmol, 5.0 equiv.) was then added and the reaction mixture was stirred at 50 °C for 1 hours. The crude product was purified by column chromatography (10 → 40% EtOAc in pentane) to afford the *title compound* **30** as a yellow oil (33.1 mg, 0.174 mmol, 58%).

***R$_f$*** 0.22 (20% EtOAc in pentane)

**$^1$H NMR** (400 MHz, $CDCl_3$) $\delta_H$ 8.83 (dd, $J$ = 2.2, 0.9 Hz, 1H), 7.94 (dd, $J$ = 8.1, 2.2 Hz, 1H), 7.46 (dd, $J$ = 8.1, 0.9 Hz, 1H), 4.19 (q, $J$ = 7.2 Hz, 2H), 3.92 (s, 2H), 1.27 (t, $J$ = 7.2 Hz, 3H).

**$^{13}$C NMR** (101 MHz, $CDCl_3$) $\delta_C$ 169.6 (C), 158.9 (C), 152.3 (CH), 139.8 (CH), 124.2 (CH), 116.7 (C), 108.6 (C), 61.6 ($CH_2$), 44.2 ($CH_2$), 14.3 ($CH_3$).

**HRMS** (ESI$^-$) m/z calcd. for $C_{10}H_9NO_2$ $(M - H)^-$ 189.0670, found 189.0664.

**Ethyl 2-(5-(morpholine-4-carbonyl)pyridin-2-yl)acetate (31)**

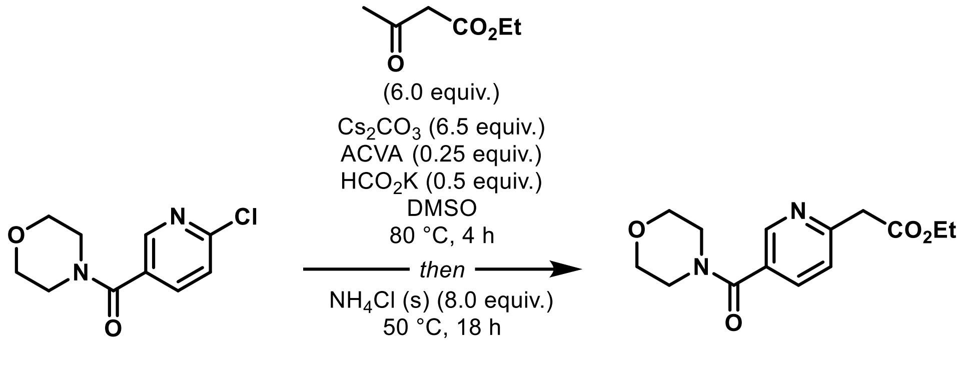


Synthesised according to **General Procedure A** with (6-chloropyridin-3-yl)(morpholino)methanone (68.0 mg, 0.300 mmol, 1.0 equiv.), ethyl acetoacetate (234 mg, 1.80 mmol, 6.0 equiv.), $Cs_2CO_3$ (635 mg, 1.95 mmol, 6.5 equiv.), ACVA (25.6 mg, 75 µmol, 0.25 equiv.) and $HCO_2K$ (12.6 mg, 150 µmol, 0.5 equiv.) in anhydrous DMSO (1.5 mL). The reaction mixture was heated at 80 °C for 4 hours. $NH_4Cl$ (128 mg, 2.40 mmol, 8.0 equiv.) was then added and the reaction mixture was stirred at 50 °C for 18 hours. The crude product was purified by column chromatography (30 → 50% acetone in pentane) to afford the *title compound* **31** as a yellow oil (21.5 mg, 77 µmol, 26%).

***R_f*** 0.19 (40% acetone in pentane)

**$^1$H NMR** (400 MHz, $CDCl_3$) $\delta_H$ 8.61 (d, *J* = 2.2 Hz, 1H), 7.75 (dd, *J* = 8.0, 2.2 Hz, 1H), 7.39 (d, *J* = 8.0 Hz, 1H), 4.19 (q, *J* = 7.1 Hz, 2H), 3.89 (s, 2H), 3.80 – 3.41 (m, 8H), 1.27 (t, *J* = 7.1 Hz, 3H).

**$^{13}$C NMR** (101 MHz, $CDCl_3$) $\delta_C$ 170.3 (C), 167.8 (C), 156.2 (C), 147.8 (CH), 136.1 (CH), 129.7 (C), 124.0 (C), 66.9 (4CH$_2$), 61.4 (CH$_2$), 43.9 (CH$_2$), 14.3 (CH$_3$).

**HRMS** (ESI$^+$) m/z calcd. for $C_{14}H_{18}N_2NaO_4$ $(M + Na)^+$ 301.1164, found 301.1174.

**Ethyl 2-(5-(trifluoromethyl)pyridin-2-yl)acetate (32)**

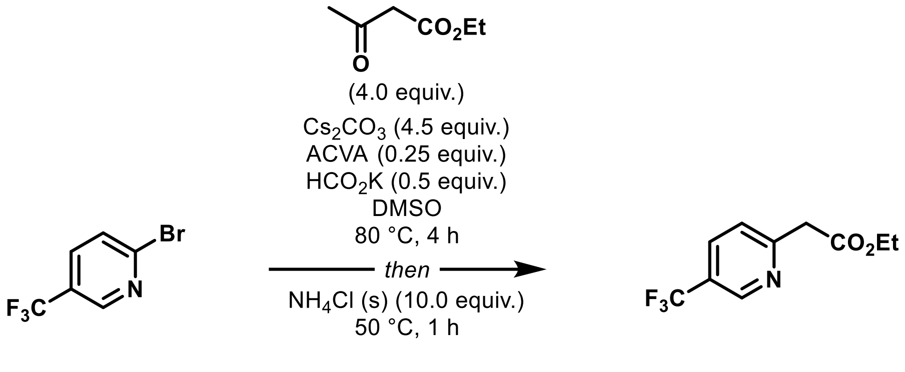


Synthesised according to **General Procedure A** with 2-bromo-5-(trifluoromethyl)pyridine (67.8 mg, 0.300 mmol, 1.0 equiv.), ethyl acetoacetate (156 mg, 1.20 mmol, 4.0 equiv.), $Cs_2CO_3$ (440 mg, 1.35 mmol, 4.5 equiv.), ACVA (25.6 mg, 75 µmol, 0.25 equiv.) and $HCO_2K$ (12.6 mg, 150 µmol, 0.5 equiv.) in anhydrous DMSO (1.5 mL). The reaction mixture was heated at 80 °C for 4 hours. $NH_4Cl$ (160 mg, 3.00 mmol, 10.0 equiv.) was then added and the reaction mixture was stirred at 50 °C for 1 hour. The crude product was purified by column chromatography (5% $Et_3N$ in pentane) to afford the *title compound* **32** as an amorphous white solid (17.5 mg, 75 µmol, 25%).

***R_f*** 0.38 (5% $Et_3N$ in pentane)

**$^1$H NMR** (400 MHz, $CDCl_3$) $\delta_H$ 8.83 (d, *J* = 2.3 Hz, 1H), 7.91 (dd, *J* = 8.2, 2.3 Hz, 1H), 7.45 (d, *J* = 8.2 Hz, 1H), 4.20 (d, *J* = 7.1 Hz, 2H), 4.92 (s, 2H),1.27 (t, *J* = 7.1 Hz, 3H).

**$^{13}$C NMR** (101 MHz, $CDCl_3$) $\delta_C$ 170.0 (C), 158.5 (C), 146.55 (q, *J* = 4.0 Hz, CH), 133.9 (q, *J* = 3.5 Hz, CH). 125.4 (q, *J* = 33.3 Hz, C), 123.7 (q, *J* = 273.1 Hz, C), 123.8 (CH), 61.5 ($CH_2$), 44.0 ($CH_2$), 14.3 ($CH_3$).

**$^{19}$F NMR** (376 MHz, $CDCl_3$) $\delta_F$ −62.4 (s, 3F)

**HRMS** ($ESI^+$) m/z calcd. for $C_{10}H_{10}F_3NNaO_2$ $(M + Na)^+$ 256.0556, found 256.0552.

**Ethyl 2-(2-fluoropyridin-4-yl)acetate (33)**

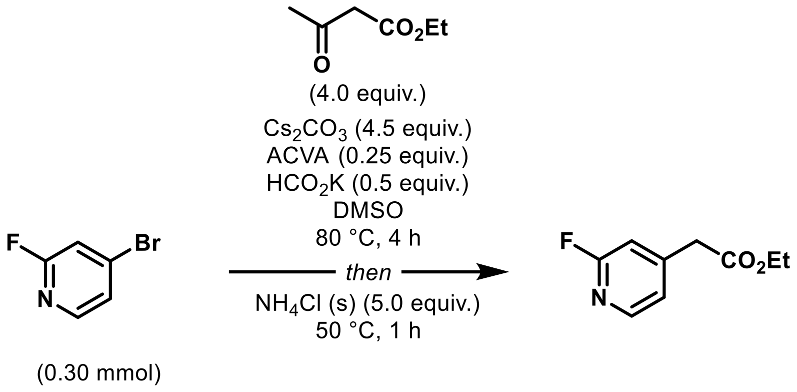


Synthesised according to **General Procedure A** with 4-bromo-2-fluoropyridine (52.8 mg, 0.300 mmol, 1.0 equiv.), ethyl acetoacetate (156 mg, 1.20 mmol, 4.0 equiv.), $Cs_2CO_3$ (440 mg, 1.35 mmol, 4.5 equiv.), ACVA (25.6 mg, 75 µmol, 0.25 equiv.) and $HCO_2K$ (12.6 mg, 150 µmol, 0.5 equiv.) in anhydrous DMSO (1.5 mL). The reaction mixture was heated at 80 °C for 4 hours. $NH_4Cl$ (80.3 mg, 1.50 mmol, 5.0 equiv.) was then added and the reaction mixture was stirred at 50 °C for 1 hour. The crude product was purified by column chromatography (10% $Et_3N$ in pentane) to afford the *title compound* **33** as a yellow oil (14.4 mg, 79 µmol, 26%).

***R<sub></sub>f*** 0.41 (10% $Et_3N$ in pentane)

**$^1$H NMR** (500 MHz, $CDCl_3$) $\delta_H$ 8.17 (d, *J* = 5.1 Hz, 1H), 7.11 (dt, *J* = 5.1, 1.7 Hz, 1H), 6.88 (d, *J* = 1.7 Hz, 1H), 4.18 (q, *J* = 7.2 Hz, 2H), 3.65 (s, 2H), 1.27 (t, *J* = 7.2 Hz, 3H).

**$^{13}$C NMR** (126 MHz, $CDCl_3$) $\delta_C$ 169.6 (C), 164.1 (d, *J* = 238.8 Hz, C), 148.6 (d, *J* = 8.2 Hz, C), 147.8 (d, *J* = 15.3 Hz, CH), 122.4 (d, *J* = 4.0 Hz, CH), 110.4 (d, *J* = 37.9 Hz, CH), 61.6 ($CH_2$), 40.6 (d, *J* = 3.0 Hz, $CH_2$), 14.2 ($CH_3$).

**$^{19}$F NMR** (471 MHz, $CDCl_3$) $\delta_F$ −68.1 (s, F)

**HRMS** (ESI$^+$) m/z calcd. for $C_9H_{11}FNO_2$ $(M + H)^+$ 184.0774, found 184.0776.

**Methyl 5-(2-ethoxy-2-oxoethyl)pyrazine-2-carboxylate (34)**

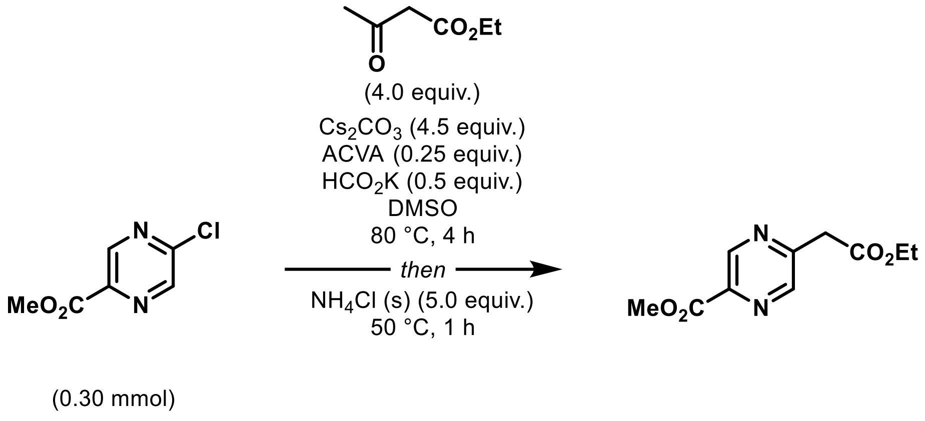


Synthesised according to **General Procedure A** with methyl 5-chloropyrazine-2-carboxylate (51.8 mg, 0.300 mmol, 1.0 equiv.), ethyl acetoacetate (156 mg, 1.20 mmol, 4.0 equiv.), $Cs_2CO_3$ (440 mg, 1.35 mmol, 4.5 equiv.), ACVA (25.6 mg, 75 µmol, 0.25 equiv.) and $HCO_2K$ (12.6 mg, 150 µmol, 0.5 equiv.) in anhydrous DMSO (1.5 mL). The reaction mixture was heated at 80 °C for 4 hours. $NH_4Cl$ (80.3 mg, 1.50 mmol, 5.0 equiv.) was then added and the reaction mixture was stirred at 50 °C for 1 hour. The crude product was purified by column chromatography (30 → 50% EtOAc in pentane) to afford the *title compound* **34** as a brown oil (40.6 mg, 0.182 mmol, 61%).

***R<sub></sub>f*** 0.23 (40% EtOAc in pentane)

**$^1$H NMR** (500 MHz, $CDCl_3$) $\delta_H$ 9.25 (d, $J$ = 1.4 Hz, 1H), 8.71 (d, $J$ = 1.4 Hz, 1H), 4.21 (q, $J$ = 7.1 Hz, 2H), 4.04 (s, 3H), 3.97 (s, 2H), 1.27 (t, $J$ = 7.1 Hz, 3H).

**$^{13}$C NMR** (126 MHz, $CDCl_3$) $\delta_C$ 169.1 (C), 164.5 (C), 153.9 (C), 145.6 (CH), 144.9 (CH), 141.7 (C), 61.8 ($CH_2$), 53.3 ($CH_3$), 41.6 ($CH_2$), 14.2 ($CH_3$).

**HRMS** ($ESI^+$) m/z calcd. for $C_{10}H_{12}N_2NaO_4$ $(M + Na)^+$ 247.0695, found 247.0688.

**Ethyl 2-(pyrimidin-5-yl)acetate (35)**

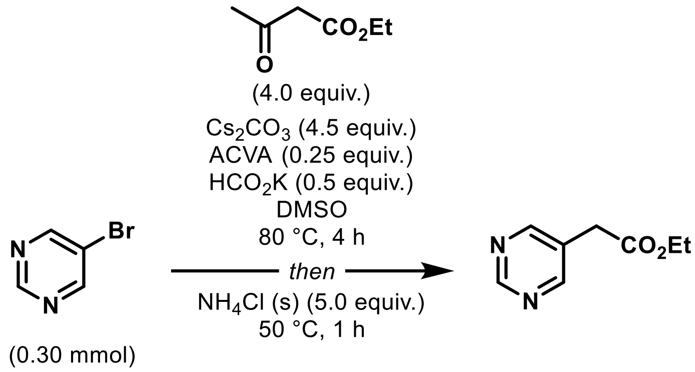


Synthesised according to **General Procedure A** with 5-bromopyrimidine (47.7 mg, 0.300 mmol, 1.0 equiv.), ethyl acetoacetate (156 mg, 1.20 mmol, 4.0 equiv.), $Cs_2CO_3$ (440 mg, 1.35 mmol, 4.5 equiv.), ACVA (25.6 mg, 75 µmol, 0.25 equiv.) and $HCO_2K$ (12.6 mg, 150 µmol, 0.5 equiv.) in anhydrous DMSO (1.5 mL). The reaction mixture was heated at 80 °C for 4 hours. $NH_4Cl$ (80.3 mg, 1.50 mmol, 5.0 equiv.) was then added and the reaction mixture was stirred at 50 °C for 1 hour. The crude product was purified by column chromatography (40 → 60% EtOAc in pentane) to afford the *title compound* **35** as a yellow oil (28.4 mg, 0.171 mmol, 57%).

***R**$_f$* 0.37 (50% EtOAc in pentane).

**$^1$H NMR** (400 MHz, $CDCl_3$) $\delta_H$ 9.13 (s, 1H), 8.68 (s, 2H), 4.18 (q, $J$ = 7.1 Hz, 2H), 3.62 (s, 2H), 1.26 (t, $J$ = 7.1 Hz, 3H).

**$^{13}$C NMR** (126 MHz, $CDCl_3$) $\delta_C$ 169.8 (C), 157.7 (CH), 157.5 (2CH), 128.1 (C), 61.8 ($CH_2$), 36.1 ($CH_2$), 14.2 ($CH_3$).

**HRMS** ($ESI^+$) m/z calcd. for $C_8H_{11}N_2O_2$ $(M + H)^+$ 167.0815, found 167.0813.

**Ethyl 2-(quinolin-4-yl)acetate (36)**

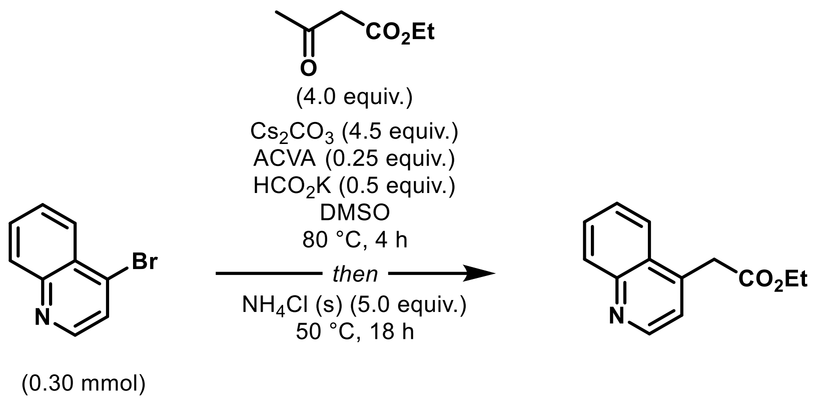


Synthesised according to **General Procedure A** with 4-bromoquinoline (62.4 mg, 0.300 mmol, 1.0 equiv.), ethyl acetoacetate (156 mg, 1.20 mmol, 4.0 equiv.), $Cs_2CO_3$ (440 mg, 1.35 mmol, 4.5 equiv.), ACVA (25.6 mg, 75 µmol, 0.25 equiv.) and $HCO_2K$ (12.6 mg, 150 µmol, 0.5 equiv.) in anhydrous DMSO (1.5 mL). The reaction mixture was heated at 80 °C for 4 hours. $NH_4Cl$ (80.3 mg, 1.50 mmol, 5.0 equiv.) was then added and the reaction mixture was stirred at 50 °C for 18 hours. The crude product was purified by column chromatography (50% $Et_2O$ in pentane) to afford the *title compound* **36** as a yellow oil (37.5 mg, 0.174 mmol, 58%).

$R_f$ 0.23 (50% $Et_2O$ in pentane)

**$^1$H NMR** (400 MHz, $CDCl_3$) $\delta_H$ 8.87 (d, $J$ = 4.4 Hz, 1H), 8.14 (dd, $J$ = 8.2, 0.9 Hz, 1H), 8.01 (dd, $J$ = 8.5, 1.4 Hz, 1H), 7.73 (ddd, $J$ = 8.2, 6.9, 1.4 Hz, 1H), 7.59 (ddd, $J$ = 8.5, 6.9, 0.9 Hz, 1H), 7.34 (d, $J$ = 4.4 Hz, 1H), 4.17 (q, $J$ = 7.1 Hz, 2H), 4.07 (s, 2H), 1.23 (t, $J$ = 7.1 Hz, 3H).

**$^{13}$C NMR** (101 MHz, $CDCl_3$) $\delta_C$ 170.3 (C), 150.3 (CH), 148.6 (C), 140.3 (C), 130.4 (CH), 129.5 (CH), 127.6 (C), 127.0 (CH), 123.7 (CH), 122.7 (CH), 61.5 ($CH_2$), 38.6 ($CH_2$), 14.2 ($CH_3$).

**HRMS** (ESI$^+$) m/z calcd. for $C_{13}H_{13}NNaO_2$ $(M + Na)^+$ 238.0838, found 238.0837.

**Ethyl 2-(isoquinolin-4-yl)acetate (37)**

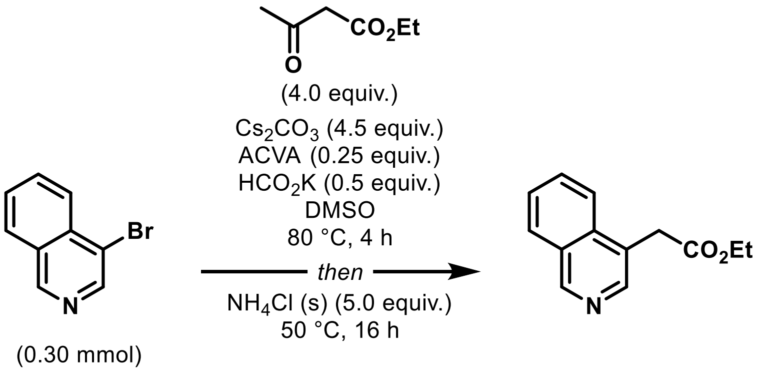


Synthesised according to **General Procedure A** with 4-bromoisoquinoline (62.4 mg, 0.300 mmol, 1.0 equiv.), ethyl acetoacetate (156 mg, 1.20 mmol, 4.0 equiv.), $Cs_2CO_3$ (440 mg, 1.35 mmol, 4.5 equiv.), ACVA (25.6 mg, 75 µmol, 0.25 equiv.) and $HCO_2K$ (12.6 mg, 150 µmol, 0.5 equiv.) in anhydrous DMSO (1.5 mL). The reaction mixture was heated at 80 °C for 4 hours. $NH_4Cl$ (80.3 mg, 1.50 mmol, 5.0 equiv.) was then added and the reaction mixture was stirred at 50 °C for 16 hours. The crude product was purified by column chromatography (20 → 50% EtOAc in pentane) to afford the *title compound* **37** as a pale-yellow solid (20.5 mg, 95 µmol, 32%).

**mp** 38–39 °C. $\boldsymbol{R_f}$ 0.14 (30% $Et_2O$ in pentane).

**$^1$H NMR** (500 MHz, $CDCl_3$) $\delta_H$ 9.21 (s, 1H), 8.45 (s, 1H), 8.02 (d, $J$ = 8.0 Hz, 1H), 7.99 (d, $J$ = 8.6 Hz, 1H), 7.78 (ddd, $J$ = 8.6., 7.0, 1.1 Hz, 1H), 7.65 (ddd, $J$ = 8.0, 1.1 Hz, 1H), 4.16 (q, $J$ = 7.1 Hz, 2H), 4.02 (s, 2H), 1.22 (t, $J$ = 7.1 Hz, 3H).

**$^{13}$C NMR** (101 MHz, $CDCl_3$) $\delta_C$ 171.0 (C), 152.5 (CH), 143.9 (CH), 135.1 (C), 131.0 (CH), 128.5 (CH), 127.4 (CH), 123.3 (CH), 61.4 ($CH_2$), 36.5 ($CH_2$), 14.3 ($CH_3$).

Note: two quaternary carbon signals could not be identified due its low concentration.

**HRMS** (ESI$^+$) m/z calcd. for $C_{13}H_{14}NO_2$ $(M + H)^+$ 216.1019, found 216.1027.

**Ethyl 2-(quinolin-3-yl)acetate (38)**

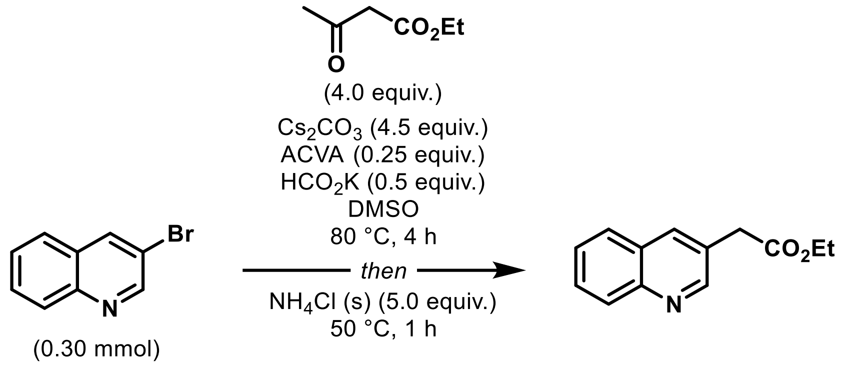


Synthesised according to **General Procedure A** with 3-bromoquinoline (62.4 mg, 0.300 mmol, 1.0 equiv.), ethyl acetoacetate (156 mg, 1.20 mmol, 4.0 equiv.), $Cs_2CO_3$ (440 mg, 1.35 mmol, 4.5 equiv.), ACVA (25.6 mg, 75 µmol, 0.25 equiv.) and $HCO_2K$ (12.6 mg, 150 µmol, 0.5 equiv.) in anhydrous DMSO (1.5 mL). The reaction mixture was heated at 80 °C for 4 hours. $NH_4Cl$ (80.3 mg, 1.50 mmol, 5.0 equiv.) was then added and the reaction mixture was stirred at 50 °C for 1 hour. The crude product was purified by column chromatography (10 → 50% EtOAc in pentane) to afford the *title compound* **38** as a yellow solid (36.9 mg, 0.171 mmol, 57%).

**mp** 39–40 °C. ***R*$_f$** 0.19 (30% EtOAc in pentane).

**$^1$H NMR** (400 MHz, $CDCl_3$) $\delta_H$ 8.84 (d, $J$ = 2.3 Hz, 1H), 8.12 – 8.05 (m, 2H), 7.79 (dd, $J$ = 8.1, 1.5 Hz, 1H), 7.69 (ddd, $J$ = 8.4, 7.0, 1.5 Hz, 1H), 7.53 (ddd, $J$ = 8.1, 7.0, 1.1 Hz, 1H), 4.18 (q, $J$ = 7.1 Hz, 2H), 3.80 (s, 2H), 1.26 (t, $J$ = 7.1 Hz, 3H).

**$^{13}$C NMR** (101 MHz, $CDCl_3$) $\delta_C$ 170.8 (C), 151.7 (CH), 147.3 (C), 136.0 (CH), 129.5 (CH), 129.3 (CH), 128.0 (C), 127.7 (CH), 127.2 (C), 127.0 (CH), 61.4 ($CH_2$), 38.9 ($CH_2$), 14.2 ($CH_3$).

**HRMS** (ESI$^+$) m/z calcd. for $C_{13}H_{14}NO_2$ $(M + H)^+$ 216.1019, found 216.1017.

**Ethyl 2-(quinoxalin-2-yl)acetate (39)**

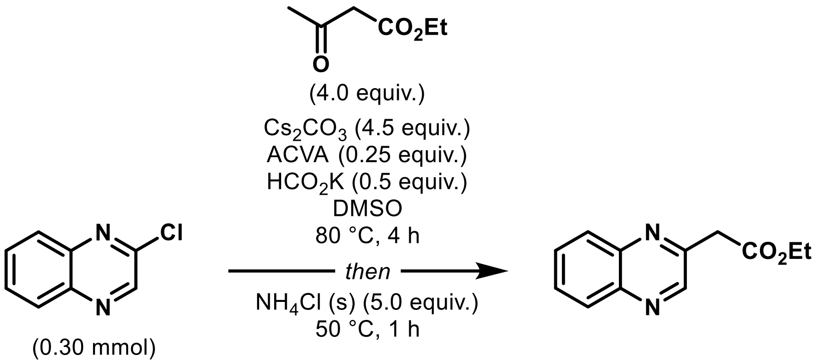


Synthesised according to **General Procedure A** with 2-chloroquinoxaline (49.4 mg, 0.300 mmol, 1.0 equiv.), ethyl acetoacetate (156 mg, 1.20 mmol, 4.0 equiv.), $Cs_2CO_3$ (440 mg, 1.35 mmol, 4.5 equiv.), ACVA (25.6 mg, 75 µmol, 0.25 equiv.) and $HCO_2K$ (12.6 mg, 150 µmol, 0.5 equiv.) in anhydrous DMSO (1.5 mL). The reaction mixture was heated at 80 °C for 4 hours. $NH_4Cl$ (80.3 mg, 1.50 mmol, 5.0 equiv.) was then added and the reaction mixture was stirred at 50 °C for 1 hour. The crude product was purified by column chromatography (30 → 50% EtOAc in pentane) to afford the *title compound* **39** as an orange oil (30.2 mg, 0.140 mmol, 47%).

***R**$_f$* 0.61 (50% EtOAc in pentane).

**$^1$H NMR** (500 MHz, $CDCl_3$) $\delta_H$ 8.87 (s, 1H), 8.17 – 8.07 (m, 1H), 8.08 – 8.00 (m, 1H), 7.81 – 7.71 (m, 2H), 4.22 (q, *J* = 7.1 Hz, 2H), 4.08 (s, 2H), 1.27 (t, *J* = 7.1 Hz, 3H).

**$^{13}$C NMR** (126 MHz, $CDCl_3$) $\delta_C$ 169.7 (C), 150.0 (C), 146.0 (CH), 142.2 (C), 141.6 (C), 130.3 (CH), 129.9 (CH), 129.4 (CH), 129.2 (CH), 61.6 ($CH_2$), 42.4 ($CH_2$), 14.3 ($CH_3$).

**HRMS** ($APCI^+$) m/z calcd. for $C_{12}H_{13}N_2O_2$ $(M + H)^+$ 217.0972, found 217.0967.

**Ethyl 2-(quinazolin-4-yl)acetate (40)**

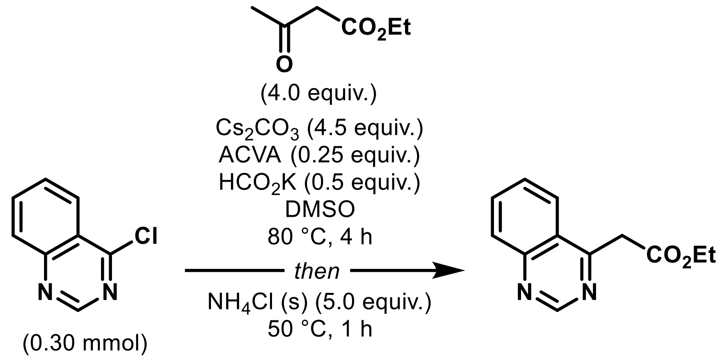


Synthesised according to **General Procedure A** with 4-chloroquinazoline (49.4 mg, 0.300 mmol, 1.0 equiv.), ethyl acetoacetate (156 mg, 1.20 mmol, 4.0 equiv.), $Cs_2CO_3$ (440 mg, 1.35 mmol, 4.5 equiv.), ACVA (25.6 mg, 75 µmol, 0.25 equiv.) and $HCO_2K$ (12.6 mg, 150 µmol, 0.5 equiv.) in anhydrous DMSO (1.5 mL). The reaction mixture was heated at 80 °C for 4 hours. $NH_4Cl$ (80.3 mg, 1.50 mmol, 5.0 equiv.) was then added and the reaction mixture was stirred at 50 °C for 1 hour. The crude product was purified by column chromatography (30 → 50% EtOAc in pentane) to afford the *title compound* **40** as a pale-yellow solid (33.6 mg, 0.155 mmol, 52%).

**mp** 86–88 °C. $\boldsymbol{R_f}$ 0.31 (30% EtOAc in pentane).

Note: **40** exists as a mixture of tautomers (86:14, enamine:imine) in solution.

$^{1}$**H NMR** (500 MHz, $CDCl_3$) $\delta_H$ 12.35 (br s, 1H, *enamine*), 9.26 (s, 1H, *imine*), 8.07 (dd, $J$ = 8.2, 1.4 Hz, 1H, *imine*), 7.92 (ddd, $J$ = 8.2, 6.9, 1.4 Hz, 1H, *imine*), 7.80 (d, $J$ = 3.2 Hz, 1H, *enamine*), 7.74 (dd, $J$ = 8.1, 1.4 Hz, 1H, *enamine*), 7.67 (ddd, $J$ = 8.4, 6.9, 1.2 Hz, 1H, *imine*), 7.59 (ddd, $J$ = 8.3, 7.0, 1.4 Hz, 1H, *enamine*), 7.54 (dd, $J$ = 8.2, 1.4 Hz, 1H, *enamine*), 7.35 (ddd, $J$ = 8.3, 7.0, 1.4 Hz, 1H, *enamine*), 5.50 (s, 1H, *enamine*), 4.32 (s, 2H, *imine*), 4.24 – 4.15 (m, 4H, *enamine* + *imine*), 1.32 (t, $J$ = 7.1 Hz, 3H, *enamine*), 1.24 (t, $J$ = 7.1 Hz, 3H, *imine*).

$^{13}$**C NMR** (101 MHz, $CDCl_3$) $\delta_C$ 170.9 (C, *enamine*), 154.7 (CH, *imine*), 149.0 (C, *enamine*), 144.6 (C, *enamine*), 142.6 (CH, *enamine*), 134.1 (CH, *imine*), 133.1 (CH, *enamine*), 129.4 (CH, *imine*), 128.24 (CH, *enamine*), 128.17 (CH, *imine*), 127.3 (CH, *enamine*), 124.8 (CH, *imine*), 123.3 (CH, *enamine*), 120.6 (C, *enamine*), 79.5 (CH, *enamine*), 61.8 ($CH_2$, *imine*), 59.7 ($CH_2$, *enamine*), 41.7 ($CH_2$, *imine*), 14.6 ($CH_3$, *enamine*), 14.2 ($CH_3$, *imine*).

Note: four quaternary carbon signals for the imine tautomer could not be identified due its low concentration.

**HRMS** (ESI$^+$) m/z calcd. for $C_{12}H_{12}N_2NaO_2$ $(M + Na)^+$ 239.0797, found 239.0792.

**Ethyl 2-(benzo[*d*]thiazol-2-yl)acetate (41)**

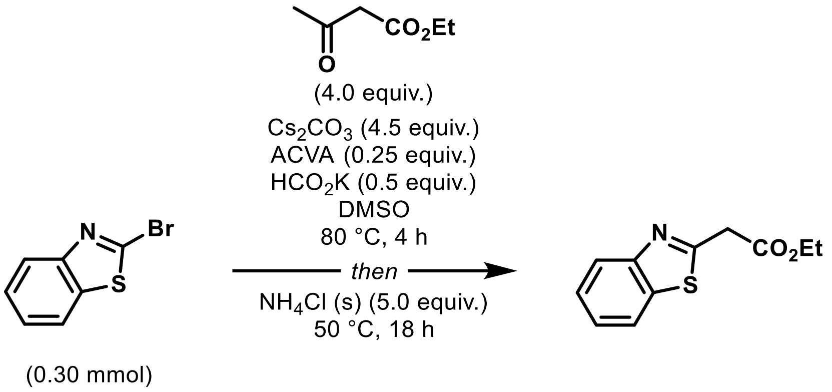


Synthesised according to **General Procedure A** with 2-bromobenzo[*d*]thiazole (64.2 mg, 0.300 mmol, 1.0 equiv.), ethyl acetoacetate (156 mg, 1.20 mmol, 4.0 equiv.), $Cs_2CO_3$ (440 mg, 1.35 mmol, 4.5 equiv.), ACVA (25.6 mg, 75 μmol, 0.25 equiv.) and $HCO_2K$ (12.6 mg, 150 μmol, 0.5 equiv.) in anhydrous DMSO (1.5 mL). The reaction mixture was heated at 80 °C for 4 hours. $NH_4Cl$ (80.3 mg, 1.50 mmol, 5.0 equiv.) was then added and the reaction mixture was stirred at 50 °C for 18 hours. The crude product was purified by column chromatography (5% $Et_3N$ in pentane) to afford the *title compound* **41** as a yellow oil (13.4 mg, 61 μmol, 20%).

***$R_f$*** 0.27 (5% $Et_3N$ in pentane)

**$^1$H NMR** (400 MHz, $CDCl_3$) $\delta_H$ 8.01 (d, *J* = 8.2 Hz, 1H), 7.88 (dd, *J* = 8.3, 1.2 Hz, 1H), 7.48 (ddd, *J* = 8.2, 7.2, 1.2 Hz, 1H), 7.39 (ddd, *J* = 8.3, 7.2, 1.2 Hz, 1H), 4.25 (q, *J* = 7.2 Hz, 2H), 4.18 (s, 2H), 1.30 (t, *J* = 7.2 Hz, 3H).

**$^{13}$C NMR** (101 MHz, $CDCl_3$) $\delta_C$ 168.6 (C), 162.9 (C), 152.8 (C), 135.9 (C), 126.2 (CH), 125.4 (CH), 123.1 (CH), 121.7 (CH), 61.9 ($CH_2$), 40.0 ($CH_2$), 14.3 ($CH_3$).

**HRMS** ($ESI^+$) m/z calcd. for $C_{11}H_{11}NNaO_2S$ $(M + Na)^+$ 244.0403, found 244.0394.

**Ethyl 2-(pyrazolo[1,5-a]pyrimidin-5-yl)acetate (42)**

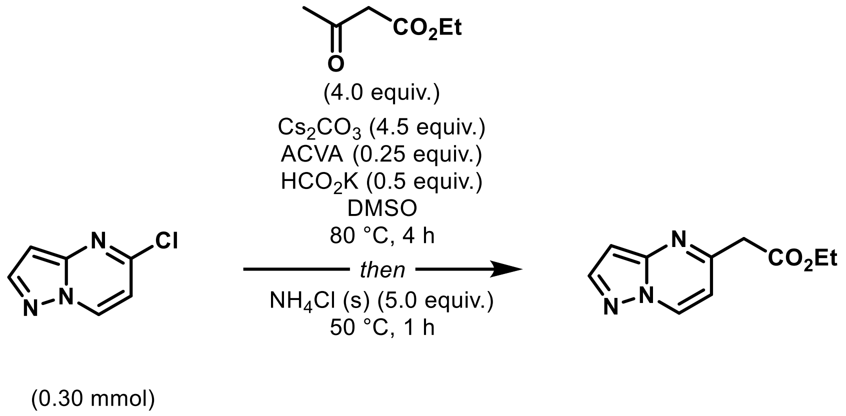

Synthesised according to **General Procedure A** with 5-chloropyrazolo[1,5-a]pyrimidine (46.1 mg, 0.300 mmol, 1.0 equiv.), ethyl acetoacetate (156 mg, 1.20 mmol, 4.0 equiv.), $Cs_2CO_3$ (440 mg, 1.35 mmol, 4.5 equiv.), ACVA (25.6 mg, 75 µmol, 0.25 equiv.) and $HCO_2K$ (12.6 mg, 150 µmol, 0.5 equiv.) in anhydrous DMSO (1.5 mL). The reaction mixture was heated at 80 °C for 4 hours. $NH_4Cl$ (80.3 mg, 1.50 mmol, 5.0 equiv.) was then added and the reaction mixture was stirred at 50 °C for 1 hour. The crude product was purified by column chromatography (50% EtOAc in pentane) to afford the *title compound* **42** as a colourless oil (8.00 mg, 39 µmol, 13%).

$\boldsymbol{R_f}$ 0.42 (50% EtOAc in pentane)

**$^1$H NMR** (400 MHz, $CDCl_3$) $\delta_H$ 8.62 (dd, $J$ = 7.2, 1.0 Hz, 1H), 8.10 (d, $J$ = 2.3 Hz, 1H), 6.85 (d, $J$ = 7.2 Hz, 1H), 6.63 (dd, $J$ = 2.3, 1.0 Hz, 1H), 4.21 (q, $J$ = 7.1 Hz, 2H), 3.87 (s, 2H), 1.27 (t, $J$ = 7.1 Hz, 3H).

**$^{13}$C NMR** (101 MHz, $CDCl_3$) $\delta_C$ 169.5 (C), 154.7 (C), 148.2 (C), 145.5 (CH), 135.0 (CH), 108.7 (C), 96.7 (C), 61.6 ($CH_2$), 44.2 ($CH_2$), 14.3 ($CH_3$).

**HRMS** ($ESI^+$) m/z calcd. for $C_{10}H_{12}N_3O_2$ $(M + H)^+$ 206.0924, found 206.0920.

**Ethyl 2-(imidazo[1,2-*b*]pyridazin-6-yl)acetate (43)**

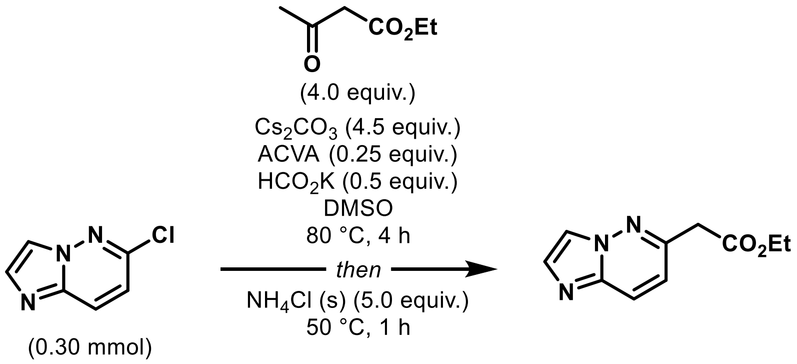


Synthesised according to **General Procedure A** with 6-chloroimidazo[1,2-*b*]pyridazine (46.1 mg, 0.300 mmol, 1.0 equiv.), ethyl acetoacetate (156 mg, 1.20 mmol, 4.0 equiv.), $Cs_2CO_3$ (440 mg, 1.35 mmol, 4.5 equiv.), ACVA (25.6 mg, 75 μmol, 0.25 equiv.) and $HCO_2K$ (12.6 mg, 150 μmol, 0.5 equiv.) in anhydrous DMSO (1.5 mL). The reaction mixture was heated at 80 °C for 4 hours. $NH_4Cl$ (80.3 mg, 1.50 mmol, 5.0 equiv.) was then added and the reaction mixture was stirred at 50 °C for 1 hour. The crude product was purified by column chromatography (70 → 90% EtOAc in pentane) to afford the *title compound* **43** as a white solid (53.9 mg, 0.263 mmol, 88%).

**mp** 40–42 °C. $\boldsymbol{R_f}$ 0.19 (70% EtOAc in pentane).

**$^1$H NMR** (500 MHz, $CDCl_3$) $\delta_H$ 7.91 – 7.87 (m, 2H), 7.72 (br s, 1H), 7.03 (d, $J$ = 9.3 Hz, 1H), 4.19 (q, $J$ = 7.1 Hz, 2H), 3.83 (s, 2H), 1.25 (t, $J$ = 7.1 Hz, 3H).

**$^{13}$C NMR** (126 MHz, $CDCl_3$) $\delta_C$ 169.5 (C), 148.9 (C), 138.3 (C), 133.9 (CH), 125.7 (CH), 118.6 (CH), 116.8 (CH), 61.6 ($CH_2$), 41.2 ($CH_2$), 14.2 ($CH_3$).

**HRMS** ($ESI^+$) m/z calcd. for $C_{10}H_{12}N_3O_2$ $(M + H)^+$ 206.0924, found 206.0927.

**Methyl 2-(4-cyanophenyl)acetate (69)**

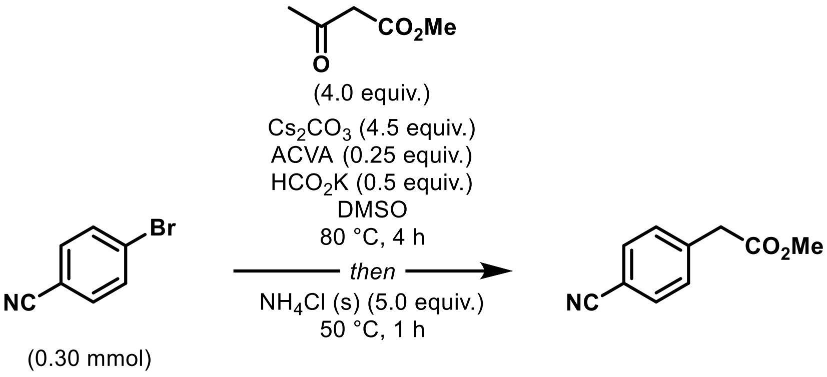


Synthesised according to **General Procedure A** with 4-bromobenzonitrile (54.6 mg, 0.300 mmol, 1.0 equiv.), diethyl malonate (192 mg, 1.20 mmol, 4.0 equiv.), $Cs_2CO_3$ (440 mg, 1.35 mmol, 4.5 equiv.), ACVA (25.6 mg, 75 µmol, 0.25 equiv.) and $HCO_2K$ (12.6 mg, 150 µmol, 0.5 equiv.) in anhydrous DMSO (1.5 mL). The reaction mixture was heated at 80 °C for 4 hours. $NH_4Cl$ (80.3 mg, 1.50 mmol, 5.0 equiv.) was then added and the reaction mixture was stirred at 50 °C for 1 hour. The crude product was purified by column chromatography (10% $Et_3N$ in pentane) to afford the *title compound* **69** as a white solid (40.3 mg, 0.230 mmol, 77%).

**mp** 51–53 °C. $\boldsymbol{R_f}$ 0.21 (20% $Et_2O$ in pentane).

**$^1$H NMR** (400 MHz, $CDCl_3$) $\delta_H$ 7.62 (d, $J$ = 8.4 Hz, 2H), 7.40 (d, $J$ = 8.4 Hz, 2H), 3.71 (s, 3H), 3.69 (s, 2H).

**$^{13}$C NMR** (101 MHz, $CDCl_3$) $\delta_C$ 170.9 (C), 139.4 (C), 132.5 (2CH), 130.3 (2CH), 118.8 (C), 111.3 (C), 52.5 ($CH_3$), 41.19 ($CH_3$).

**HRMS** ($ESI^+$) m/z calcd. for $C_{10}H_9NNaO_2$ $(M + Na)^+$ 198.0525, found 198.0519.

***Tert*-butyl 2-(4-cyanophenyl)acetate (70)**

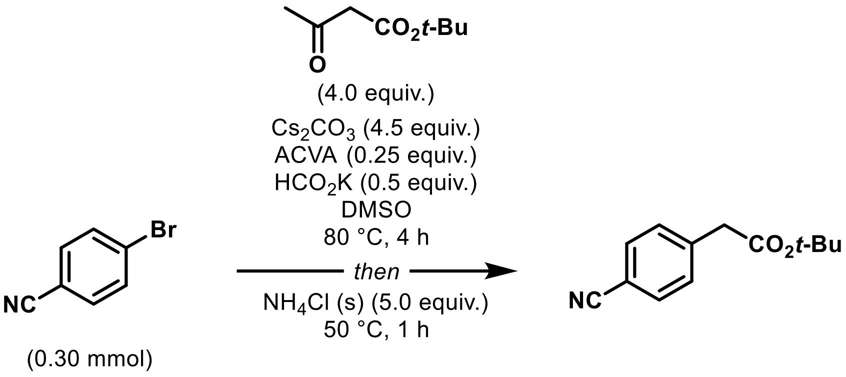


Synthesised according to **General Procedure A** with 4-bromobenzonitrile (54.6 mg, 0.300 mmol, 1.0 equiv.), *tert*-butyl 3-oxobutanoate (190 mg, 1.20 mmol, 4.0 equiv.), $Cs_2CO_3$ (440 mg, 1.35 mmol, 4.5 equiv.), ACVA (25.6 mg, 75 μmol, 0.25 equiv.) and $HCO_2K$ (12.6 mg, 150 μmol, 0.5 equiv.) in anhydrous DMSO (1.5 mL). The reaction mixture was heated at 80 °C for 4 hours. $NH_4Cl$ (80.3 mg, 1.50 mmol, 5.0 equiv.) was then added and the reaction mixture was stirred at 50 °C for 1 hour. The crude product was purified by column chromatography (1 → 20% $Et_2O$ in pentane) to afford the *title compound* **70** as a white solid (27.4 mg, 0.126 mmol, 42%).

**mp** 54–56 °C. $\boldsymbol{R_f}$ 0.52 (30% $Et_2O$ in pentane).

**$^1$H NMR** (400 MHz, $CDCl_3$) $\delta_H$ 7.61 (d, $J$ = 8.4 Hz, 2H), 7.38 (d, $J$ = 8.4 Hz, 2H), 3.59 (s, 2H), 1.43 (s, 9H).

**$^{13}$C NMR** (101 MHz, $CDCl_3$) $\delta_C$ 169.7 (C), 140.2 (C), 132.4 (2CH), 130.3 (2CH), 119.0 (C), 111.1 (C), 81.8 (C), 42.7 ($CH_2$), 28.1 ($3CH_3$).

**HRMS** (ESI$^+$) m/z calcd. for $C_{13}H_{15}NNaO_2$ $(M + Na)^+$ 240.0995, found 240.0986.

**4-(2-Oxopropyl)benzonitrile (72)**

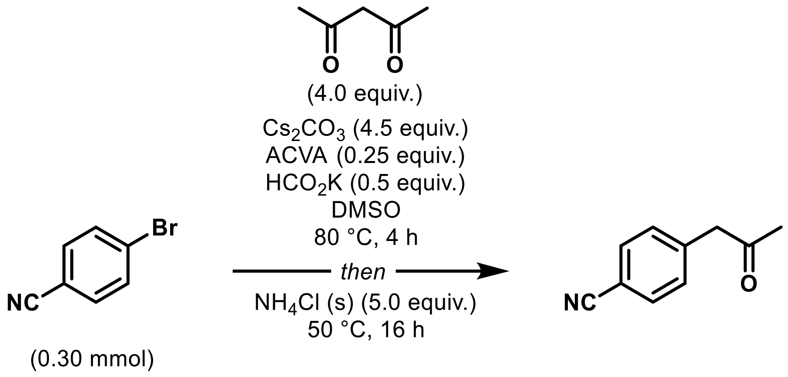


Synthesised according to **General Procedure A** with 4-bromobenzonitrile (54.6 mg, 0.300 mmol, 1.0 equiv.), acetylacetone (120 mg, 1.20 mmol, 4.0 equiv.), $Cs_2CO_3$ (440 mg, 1.35 mmol, 4.5 equiv.), ACVA (25.6 mg, 75 µmol, 0.25 equiv.) and $HCO_2K$ (12.6 mg, 150 µmol, 0.5 equiv.) in anhydrous DMSO (1.5 mL). The reaction mixture was heated at 80 °C for 4 hours. $NH_4Cl$ (80.3 mg, 1.50 mmol, 5.0 equiv.) was then added and the reaction mixture was stirred at 50 °C for 16 hours. The crude product was purified by column chromatography (30 → 50% EtOAc in pentane) to afford the *title compound* **72** as a yellow oil (30.3 mg, 0.190 mmol, 63%).

$\boldsymbol{R_f}$ 0.37 (30% EtOAc in pentane).

**$^1$H NMR** (400 MHz, $CDCl_3$) $\delta_H$ 7.63 (d, $J$ = 8.3 Hz, 2H), 7.34 – 7.25 (d, $J$ = 8.3 Hz 2H), 3.79 (s, 3H), 2.22 (s, 2H).

**$^{13}$C NMR** (101 MHz, $CDCl_3$) $\delta_C$ 204.5 (C), 139.5 (C), 132.5 (2CH), 130.5 (2CH), 118.8 (C), 111.3 (C), 50.6 ($CH_2$), 30.0 ($CH_3$).

**HRMS** ($ESI^+$) m/z calcd. for $C_{10}H_9NNaO$ $(M + Na)^+$ 182.0576, found 182.0573.

**2-(4-cyanophenyl)-*N*,*N*-diethyl-3-oxobutanamide (74)**

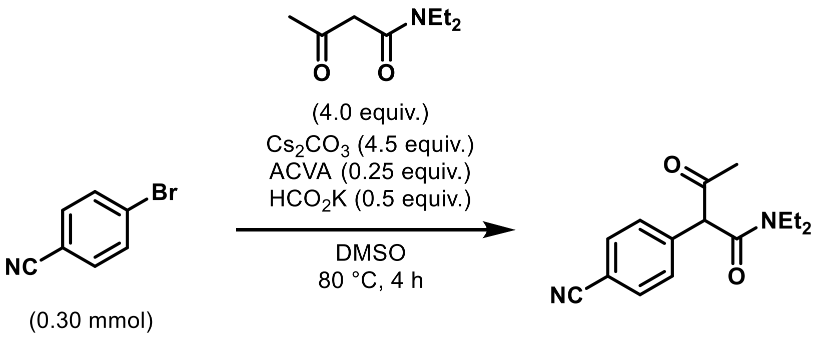


Synthesised according to **General Procedure A** with 4-bromobenzonitrile (54.6 mg, 0.300 mmol, 1.0 equiv.), *N*,*N*-diethyl-3-oxobutanamide (189 mg, 1.20 mmol, 4.0 equiv.), $Cs_2CO_3$ (440 mg, 1.35 mmol, 4.5 equiv.), ACVA (25.6 mg, 75 µmol, 0.25 equiv.) and $HCO_2K$ (12.6 mg, 150 µmol, 0.5 equiv.) in anhydrous DMSO (1.5 mL). The reaction mixture was heated at 80 °C for 4 hours. The reaction was quenched with AcOH (0.1 mL) and the crude product was purified by column chromatography (5% $Et_3N$, 10% → 50% acetone in pentane) to afford the *title compound* **74** as a colourless oil (38.8 mg, 0.150 mmol, 50%).

***R<sub></sub>**$_f$* 0.14 (5% $Et_3N$, 10% acetone in pentane).

**$^1$H NMR** (400 MHz, $CDCl_3$) $\delta_H$ 7.66 (d, $J$ = 8.4 Hz, 2H), 7.44 (d, $J$ = 8.4 Hz, 2H), 4.82 (s, 1H), 3.48 (dq, $J$ = 14.1, 7.1 Hz, 1H), 3.33 (dq, $J$ = 14.1, 7.1 Hz, 1H), 3.22 (dq, $J$ = 12.6, 7.4 Hz, 2H), 2.23 (s, 3H), 1.12 (t, $J$ = 7.1 Hz, 3H), 1.10 (t, $J$ = 7.4 Hz, 3H).

**$^{13}$C NMR** (101 MHz, $CDCl_3$) $\delta_C$ 201.2 (C), 166.8 (C), 139.2 (C), 132.7 (2CH), 130.1 (2CH), 118.6 (C), 112.1 (C), 63.4 (CH), 42.8 ($CH_2$), 40.8 ($CH_2$), 29.1 ($CH_3$), 14.5 ($CH_3$), 12.9 ($CH_3$).

**HRMS** ($APCI^+$) m/z calcd. for $C_{15}H_{18}N_2NaO_2$ $(M + Na)^+$ 281.1260, found 281.1262.

**Ethyl 2-(4-cyanophenyl)propanoate (76)**

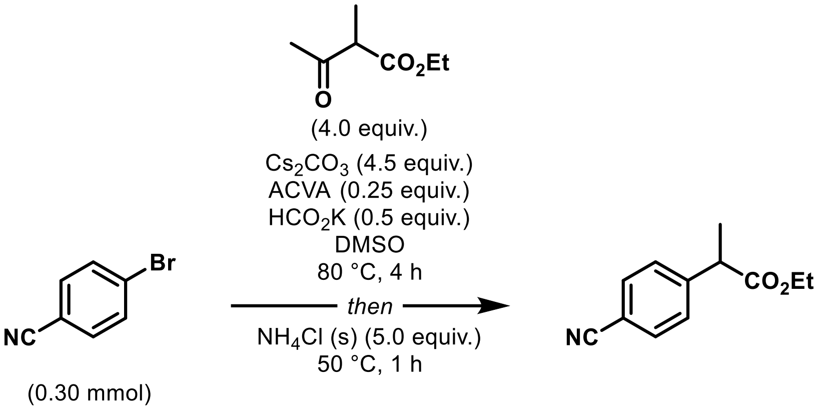

Synthesised according to **General Procedure A** with 4-bromobenzonitrile (54.6 mg, 0.300 mmol, 1.0 equiv.), diethyl malonate (192 mg, 1.20 mmol, 4.0 equiv.), $Cs_2CO_3$ (440 mg, 1.35 mmol, 4.5 equiv.), ACVA (25.6 mg, 75 μmol, 0.25 equiv.) and $HCO_2K$ (12.6 mg, 150 μmol, 0.5 equiv.) in anhydrous DMSO (1.5 mL). The reaction mixture was heated at 80 °C for 4 hours. $NH_4Cl$ (80.3 mg, 1.50 mmol, 5.0 equiv.) was then added and the reaction mixture was stirred at 50 °C for 1 hour. After this time, 1,3,5-trimethoxybenzene (50.5 mg, 0.300 mmol, 1.0 equiv.) was added to the reaction mixture, which was then analysed by $^1H$ NMR spectroscopy to approximate the yield of **76** (55 % determined by $^1H$ NMR).

***Rf*** 0.70 (20% $Et_2O$ in pentane).

**$^1$H NMR** (400 MHz, $CDCl_3$) $\delta_H$ 7.62 (d, $J$ = 8.0 Hz, 2H), 7.42 (d, $J$ = 8.0 Hz, 2H), 4.22 – 4.05 (m, 2H), 3.76 (q, $J$ = 7.1 Hz, 1H), 1.51 (d, J = 7.1 Hz, 3H), 1.21 (t, $J$ = 7.1 Hz, 3H).

**$^{13}$C NMR** (101 MHz, $CDCl_3$) $\delta_C$ 173.5 (C), 146.0 (C), 132.6 (2CH), 128.6 (2CH), 118.9 (C), 111.3 (C), 61.3 ($CH_2$), 45.8 ($CH_2$), 18.5 ($CH_3$), 14.2 ($CH_3$).

**HRMS** ($ESI^-$) m/z calcd. for $C_{12}H_{13}NO_2$ $(M - Na)^-$ 202.0874, found 202.0873.

**Ethyl 2-(4-(thiophene-2-carbonyl)phenyl)propanoate (78)**

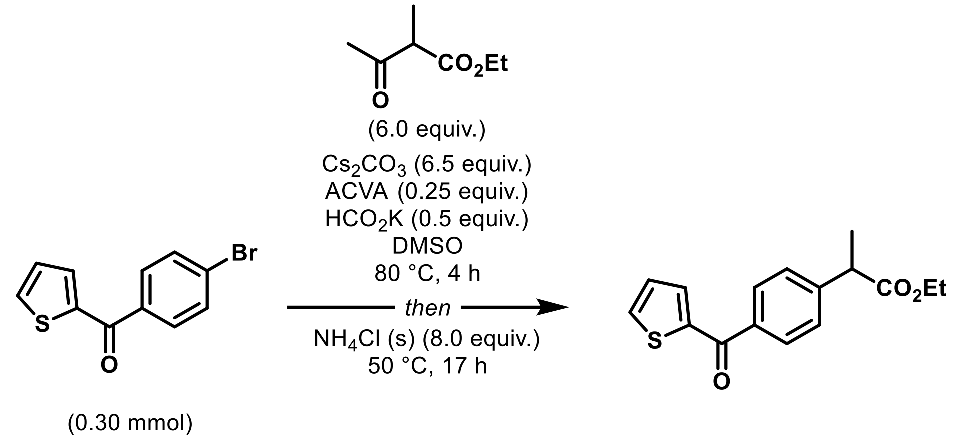


Synthesised according to **General Procedure A** with (4-bromophenyl)(thiophen-2-yl)methanone (80.1 mg, 0.300 mmol, 1.0 equiv.), diethyl 2-methylmalonate (260 mg, 1.80 mmol, 6.0 equiv.), $Cs_2CO_3$ (635 mg, 1.95 mmol, 6.5 equiv.), ACVA (25.6 mg, 75 µmol, 0.25 equiv.) and $HCO_2K$ (12.6 mg, 150 µmol, 0.5 equiv.) in anhydrous DMSO (1.5 mL). The reaction mixture was heated at 80 °C for 4 hours. $NH_4Cl$ (128 mg, 2.40 mmol, 8.0 equiv.) was then added and the reaction mixture was stirred at 50 °C for 17 hours. The crude product was purified by column chromatography (0% → 20% $Et_2O$ in pentane) to afford the *title compound* **78** as a yellow oil (36.1 mg, 0.125 mmol, 42%).

***R_f*** 0.35 (20% $Et_2O$ in pentane).

$^1$H NMR (400 MHz, $CDCl_3$) $\delta_H$ 7.84 (d, $J$ = 8.2 Hz, 2H), 7.72 (dd, $J$ = 5.0, 1.1 Hz, 1H), 7.66 (dd, $J$ = 3.8, 1.1 Hz, 1H), 7.44 (d, $J$ = 8.2 Hz, 2H), 7.17 (dd, $J$ = 5.0, 3.8 Hz, 1H), 4.23 – 4.06 (m, 2H), 3.80 (q, $J$ = 7.2 Hz, 1H), 1.54 (d, $J$ = 7.2 Hz, 3H), 1.23 (t, $J$ = 7.1 Hz, 3H).

**$^{13}$C NMR** (126 MHz, $CDCl_3$) $\delta_C$ 187.9 (C), 174.0 (Cz), 145.3 (C), 143.8 (C), 137.1 (C), 134.9 (CH), 134.3 (CH), 129.7 (2CH), 128.1 (CH), 127.9 (CH), 61.2 ($CH_2$), 45.7 (CH), 18.6 ($CH_3$), 14.3 ($CH_3$).

**HRMS** ($APCI^+$) m/z calcd. for $C_{16}H_{16}O_3NaS$ $(M + Na)^+$ 311.0712, found 311.0705.

**Ethyl 1-(4-cyanophenyl)-2-oxocyclohexane-1-carboxylate (80)**

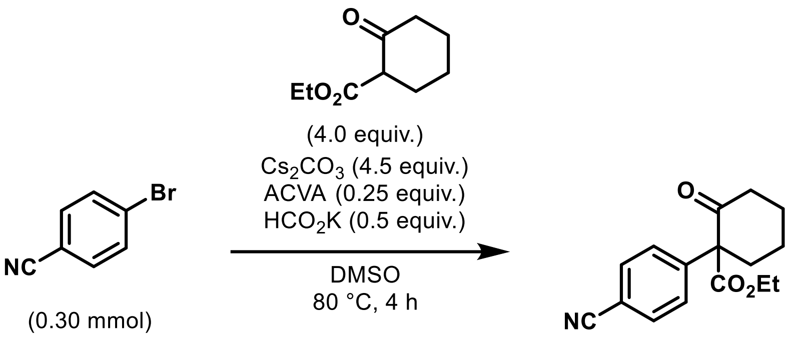


Synthesised according to **General Procedure A** with 4-bromobenzonitrile (54.6 mg, 0.300 mmol, 1.0 equiv.), ethyl 2-oxocyclohexane-1-carboxylate (204 mg, 1.20 mmol, 4.0 equiv.), $Cs_2CO_3$ (440 mg, 1.35 mmol, 4.5 equiv.), ACVA (25.6 mg, 75 μmol, 0.25 equiv.) and $HCO_2K$ (12.6 mg, 150 μmol, 0.5 equiv.) in anhydrous DMSO (1.5 mL). The reaction mixture was heated at 80 °C for 4 hours. The reaction was quenched with AcOH (0.1 mL) and the crude product was purified by column chromatography (10% $Et_3N$, 10% $Et_2O$ in pentane) to afford the *title compound* **80** as a colourless oil (21.8 mg, 80 μmol, 27%).

***R*$_f$** 0.26 (10% $Et_3N$, 10% $Et_2O$ in pentane)

**$^1$H NMR** (400 MHz, $CDCl_3$) $\delta_H$ 7.65 (d, $J$ = 8.8 Hz, 2H), 7.35 (d, $J$ = 8.7 Hz, 2H), 4.24 (q, $J$ = 7.0 Hz, 2H), 2.88 – 2.77 (m, 1H), 2.74 – 2.58 (m, 1H), 2.59 – 2.46 (m, 1H), 2.30 – 2.14 (m, 1H), 2.12 – 1.99 (m, 1H), 1.90 – 1.84 (m, 1H), 1.84 – 1.74 (m, 2H), 1.24 (t, $J$ = 7.0 Hz, 3H).

**$^{13}$C NMR** (101 MHz, $CDCl_3$) $\delta_C$ 205.3 (C), 170.4 (C), 142.2 (C), 132.1 (2CH), 128.9 (2CH), 118.7 (C), 111.7 (C), 66.5 (C), 62.3 ($CH_2$), 41.1 ($CH_2$), 35.6 ($CH_2$), 27.5 ($CH_2$), 22.4 ($CH_2$), 14.1 ($CH_3$).

**HRMS** ($ESI^+$) m/z calcd. for $C_{16}H_{17}NNaO_3$ $(M + Na)^+$ 294.1101, found 294.1101.

**Ethyl 2-cyano-2-(4-cyanophenyl)acetate (82)**

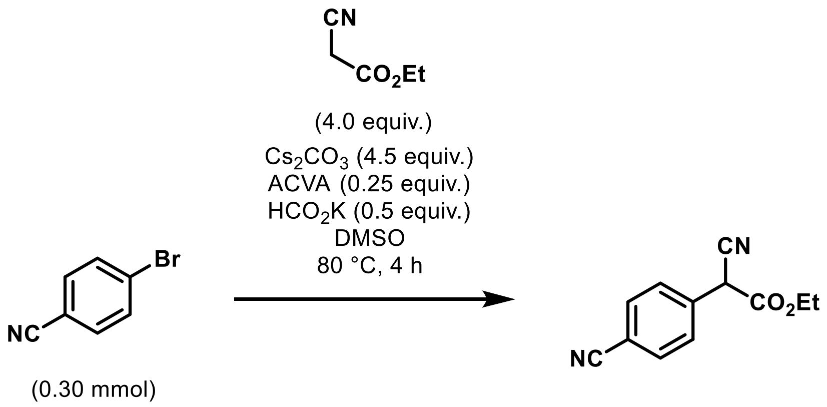


Synthesised according to **General Procedure A** with 4-bromobenzonitrile (54.6 mg, 0.300 mmol, 1.0 equiv.), ethyl 2-cyanoacetate (136 mg, 1.20 mmol, 4.0 equiv.), $Cs_2CO_3$ (440 mg, 1.35 mmol, 4.5 equiv.), ACVA (25.6 mg, 75 μmol, 0.25 equiv.) and $HCO_2K$ (12.6 mg, 150 μmol, 0.5 equiv.) in anhydrous DMSO (1.5 mL). The reaction mixture was heated at 80 °C for 4 hours. The reaction was quenched with AcOH (0.1 mL) and the crude product was purified by column chromatography (25% $Et_2O$ in pentane) to afford the *title compound* **82** as a pale-yellow oil (42.3 mg, 0.197 mmol, 66%).

***R<sub></sub>*** — 

**$^1$H NMR** (500 MHz, $CDCl_3$) $\delta_H$ 7.74 (d, *J* = 8.4 Hz, 2H), 7.61 (d, *J* = 8.4 Hz, 2H), 4.79 (s, 1H), 4.27 (qd, *J* = 7.2, 0.9 Hz, 2H), 1.30 (t, *J* = 7.2 Hz, 3H).

**$^{13}$C NMR** (126 MHz, $CDCl_3$) $\delta_C$ 164.0 (C), 134.9 (C), 133.2 (2CH), 129.0 (2CH), 117.9 (C), 114.7 (C), 113.7 (C), 64.1 ($CH_2$), 43.7 ($CH_2$), 14.0 ($CH_3$).

**HRMS** ($ESI^-$) m/z calcd. for $C_{12}H_9N_2O_2$ $(M - H)^-$ 213.0670, found 213.0659.

**Diethyl 2-(4-cyanophenyl)malonate (86)**

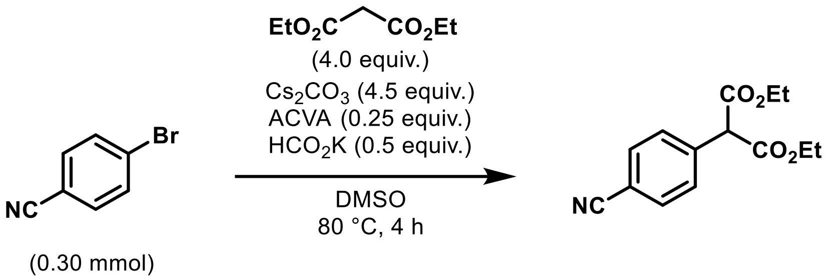


Synthesised according to **General Procedure A** with 4-bromobenzonitrile (54.6 mg, 0.300 mmol, 1.0 equiv.), diethyl malonate (192 mg, 1.20 mmol, 4.0 equiv.), $Cs_2CO_3$ (440 mg, 1.35 mmol, 4.5 equiv.), ACVA (25.6 mg, 75 µmol, 0.25 equiv.) and $HCO_2K$ (12.6 mg, 150 µmol, 0.5 equiv.) in anhydrous DMSO (1.5 mL). The reaction mixture was heated at 80 °C for 4 hours. The reaction was quenched with AcOH (0.1 mL) and the crude product was purified by column chromatography (10% $Et_3N$ in pentane) to afford the *title compound* **86** as a colourless oil (53.6 mg, 0.205 mmol, 68%).

$\boldsymbol{R_f}$ 0.14 (10% $Et_3N$ in pentane).

**$^1$H NMR** (400 MHz, $CDCl_3$) $\delta_H$ 7.66 (d, $J$ = 8.4 Hz, 2H), 7.54 (d, $J$ = 8.4 Hz, 2H), 4.65 (s, 1H), 4.31 – 4.14 (m, 4H), 1.27 (t, $J$ = 7.1 Hz, 6H).

**$^{13}$C NMR** (126 MHz, $CDCl_3$) $\delta_C$ 167.3 (C), 137.9 (C), 132.5 (2CH), 130.4 (2CH), 118.6 (C), 112.5 (C), 62.4 ($2CH_2$), 57.9 (CH), 14.1 ($2CH_3$).

**HRMS** ($ESI^+$) m/z calcd. for $C_{14}H_{15}NNaO_4$ $(M + Na)^+$ 284.0893, found 284.0883.

**Di-*tert*-butyl 2-(4-cyanophenyl)malonate (87)**

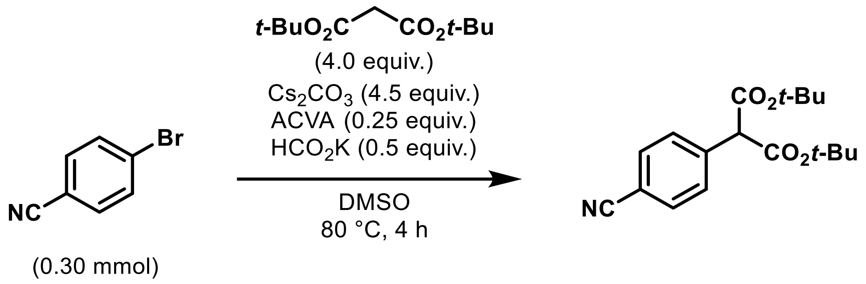


Synthesised according to **General Procedure A** with 4-bromobenzonitrile (54.6 mg, 0.300 mmol, 1.0 equiv.), di-*tert*-butyl malonate (260 mg, 1.20 mmol, 4.0 equiv.), $Cs_2CO_3$ (440 mg, 1.35 mmol, 4.5 equiv.), ACVA (25.6 mg, 75 µmol, 0.25 equiv.) and $HCO_2K$ (12.6 mg, 150 µmol, 0.5 equiv.) in anhydrous DMSO (1.5 mL). The reaction mixture was heated at 80 °C for 4 hours. The reaction was quenched with AcOH (0.1 mL) and the crude product was purified by column chromatography (10% $Et_3N$ in pentane) to afford the *title compound* **87** as a colourless oil (48.1 mg, 0.152 mmol, 51%).

***R_f*** 0.64 (10% $Et_3N$ in pentane).

**$^1$H NMR** (400 MHz, $CDCl_3$) $\delta_H$ 7.65 (d, *J* = 8.4 Hz, 2H), 7.51 (d, *J* = 8.4 Hz, 2H), 4.48 (s, 1H), 1.46 (s, 18H).

**$^{13}$C NMR** (101 MHz, $CDCl_3$) $\delta_C$ 166.5 (2C), 138.8 (C), 132.3 (2CH), 130.4 (2CH), 118.7 (C), 112.1 (C), 82.9 (2C), 60.1 (CH), 28.0 ($6CH_3$).

**HRMS** ($ESI^+$) m/z calcd. for $C_{18}H_{23}NNaO_4$ $(M + Na)^+$ 340.1519, found 340.1505.

**Diethyl 2-(4-cyanophenyl)-2-methylmalonate (88)**

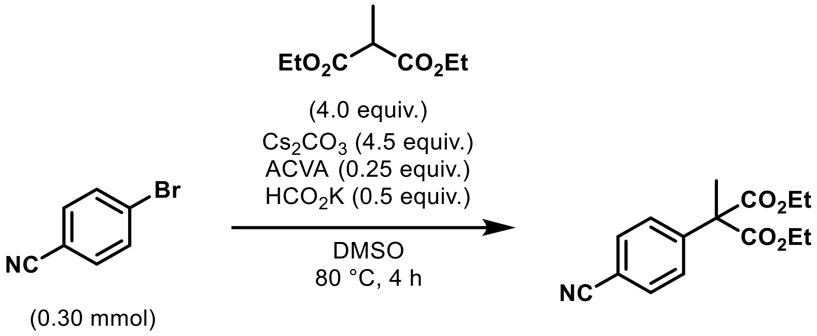


Synthesised according to **General Procedure A** with 4-bromobenzonitrile (54.6 mg, 0.300 mmol, 1.0 equiv.), diethyl 2-methylmalonate (209 mg, 1.20 mmol, 4.0 equiv.), $Cs_2CO_3$ (440 mg, 1.35 mmol, 4.5 equiv.), ACVA (25.6 mg, 75 µmol, 0.25 equiv.) and $HCO_2K$ (12.6 mg, 150 µmol, 0.5 equiv.) in anhydrous DMSO (1.5 mL). The reaction mixture was heated at 80 °C for 4 hours. The reaction was quenched with AcOH (0.1 mL) and the crude product was purified by column chromatography (10% $Et_2O$, 10% $CH_2Cl_2$ in pentane) to afford the *title compound* **88** as a colourless oil (20.2 mg, 73 µmol, 24%).

***$R_f$*** 0.35 (10% $Et_2O$, 10% $CH_2Cl_2$ in pentane).

**$^1$H NMR** (500 MHz, $CDCl_3$) $\delta_H$ 7.64 (d, *J* = 8.4 Hz, 2H), 7.50 (d, *J* = 8.4 Hz, 2H), 4.24 (qd, *J* = 7.1, 2.2 Hz, 4H), 1.86 (s, 3H), 1.26 (t, *J* = 7.1 Hz, 6H).

**$^{13}$C NMR** (126 MHz, $CDCl_3$) $\delta_C$ 170.7 (2C), 143.6 (C), 132.0 (2CH), 128.6 (2CH), 118.7 (C), 111.8 (C), 62.3 ($2CH_2$), 59.0 (C), 22.2 ($CH_3$), 14.1 ($2CH_3$).

**HRMS** ($ESI^+$) m/z calcd. for $C_{15}H_{17}O_4NNa$ $(M + Na)^+$ 298.1050, found 298.1039.

**Ethyl 2-(4-cyanophenyl)-2-((diphenylmethylene)amino)acetate (90)**

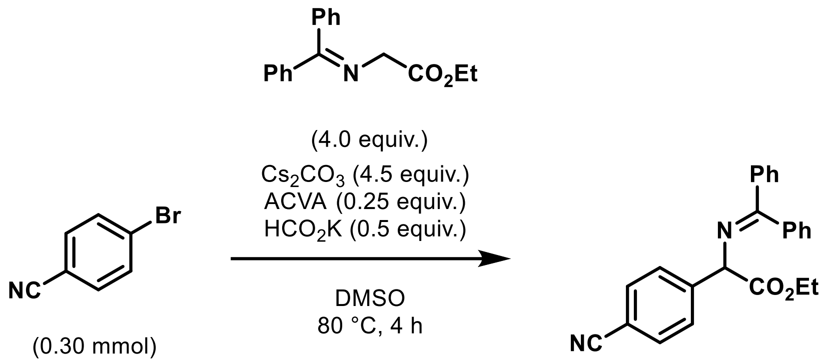

Synthesised according to **General Procedure A** with 4-bromobenzonitrile (54.6 mg, 0.300 mmol, 1.0 equiv.), ethyl 2-((diphenylmethylene)amino)acetate (321 mg, 1.20 mmol, 4.0 equiv.), $Cs_2CO_3$ (440 mg, 1.35 mmol, 4.5 equiv.), ACVA (25.6 mg, 75 µmol, 0.25 equiv.) and $HCO_2K$ (12.6 mg, 150 µmol, 0.5 equiv.) in anhydrous DMSO (1.5 mL). The reaction mixture was heated at 80 °C for 4 hours. The reaction was quenched with AcOH (0.1 mL) and the crude product was purified by column chromatography (5 → 10% $Et_3N$ in pentane) to afford the *title compound* **90** as a colourless oil (47.3 mg, 0.128 mmol, 43%).

***R$_f$*** 0.30 (10% $Et_3N$ in pentane).

**$^1$H NMR** (400 MHz, $CDCl_3$) $\delta_H$ 7.76 – 7.68 (m, 2H), 7.63 (d, *J* = 8.4 Hz, 2H), 7.59 (d, *J* = 8.4 Hz, 2H), 7.51 – 7.40 (m, 4H), 7.38 – 7.31 (m, 2H), 7.13 – 7.03 (m, 2H), 5.17 (s, 1H), 4.20 – 4.08 (m, 2H), 1.19 (t, *J* = 7.1 Hz, 3H).

**$^{13}$C NMR** (125 MHz, $CDCl_3$) $\delta_C$ 171.6 (C), 170.4 (C), 144.4 (C), 139.1 (C), 136.0 (C), 132.4 (2CH), 131.0 (CH), 129.2 (CH), 129.1 (2CH), 128.9 (2CH), 128.8 (2CH), 128.3 (2CH), 127.6 (2CH), 118.9 (C), 111.7 (C), 69.3 (CH), 61.8 ($CH_2$), 14.2 ($CH_3$).

**HRMS** (ESI$^+$) m/z calcd. for $C_{24}H_{20}O_2N_2Na$ $(M + Na)^+$ 391.1417, found 391.1403.

**4-(2-Oxocyclohexyl)benzonitrile (92)**

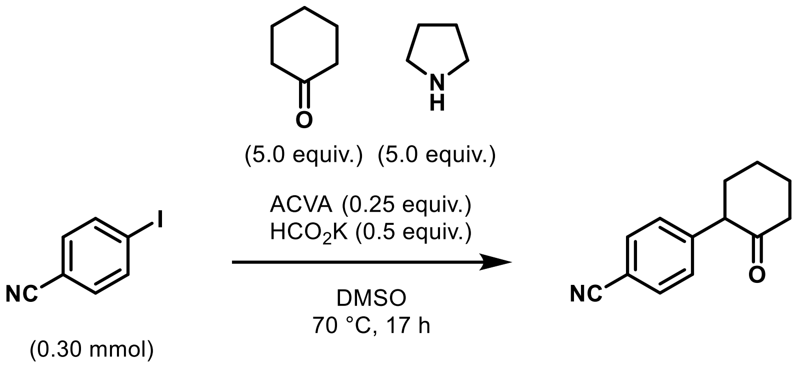


To an 8 mL screw-cap vial was charged 4-4'azobis(4-cyanovaleric acid) (ACVA) (25.6 mg, 75 µmol, 0.25 equiv.), $HCO_2K$ (12.6 mg, 150 µmol, 0.50 equiv.), $Cs_2CO_3$ (440 mg, 1.35 mmol, 4.5 equiv.), and 4-iodobenzonitrile (68.7 mg, 0.300 mmol, 1.0 equiv.). To the solids was sequentially added a magnetic stirrer bar, anhydrous DMSO (1.5 mL), cyclohexanone (147 mg, 1.50 mmol, 5.0 equiv.) and pyrrolidine (107 mg, 1.50 mmol, 5.0 equiv.). The reaction mixture was sparged with $N_2$ for 15 minutes before being sealed with parafilm. The reaction mixture was then stirred at 70 °C in a metal heating block 17 hours. The reaction mixture was cooled to room temperature before being quenched with 1 M HCl (2 mL). Subsequently, $Et_2O$ (10 mL) and water (10 mL) were added. The organic phase was collected, and the aqueous phase was extracted with $Et_2O$ (3 × 10 mL). The organics were combined, washed with brine (25 mL), dried ($MgSO_4$), and concentrated under reduced pressure. The crude product was then purified by column chromatography (30 → 40% $Et_2O$ in pentane) to afford the *title compound* **92** as a white solid (21.3 mg, 0.107 mmol, 36%).

**mp** 74–75 °C. ***R**f* 0.17 (30% $Et_2O$ in pentane).

**$^1$H NMR** (500 MHz, $CDCl_3$) $\delta_H$ 7.62 (d, *J* = 8.2 Hz, 2H), 7.24 (d, *J* = 8.2 Hz, 2H), 3.67 (dd, *J* = 12.5, 5.3 Hz, 1H), 2.67 – 2.40 (m, 2H), 2.34 – 2.24 (m, 1H), 2.24 – 2.15 (m, 1H), 2.10 – 1.92 (m, 2H), 1.91 – 1.72 (m, 2H).

**$^{13}$C NMR** (126 MHz, $CDCl_3$) $\delta_C$ 209.0 (C), 144.3 (C), 132.2 (2CH), 129.7 (2CH), 119.1 (C), 110.9 (C), 57.6 (CH), 42.4 ($CH_2$), 35.2 ($CH_2$), 27.8 ($CH_2$), 25.5 ($CH_2$).

**HRMS** ($ESI^+$) m/z calcd. for $C_{13}H_{13}ONNa$ $(M + Na)^+$ 222.0889, found 222.0997.

**Aryl Halide Thiolation**

The thiolation of aryl halides was investigated using 4-iodo-1,1'-biphenyl as a model substrate. The aryl halide was reacted with *p*-toluenethiol (2.0 equiv.), $Cs_2CO_3$ (2.5 equiv.), ACVA (0.25 equiv.) and $HCO_2K$ (0.50 equiv.) in DMSO at 80 °C for 4 hours to afford **94** in 62% yield. No product formation was observed in the absence of ACVA and $HCO_2K$.

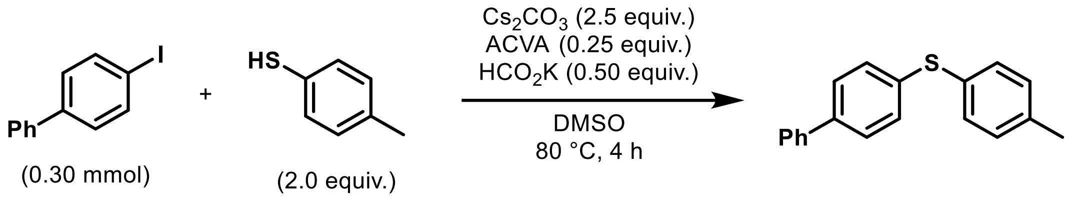


**Procedure:** To an 8 mL screw-cap vial was charged 4-4'azobis(4-cyanovaleric acid) (ACVA) (25.6 mg, 75 µmol, 0.25 equiv.), *p*-toluenethiol (74.5, 0.600 mmol, 2.0 equiv.), $HCO_2K$ (12.6 mg, 150 µmol, 0.50 equiv.), $Cs_2CO_3$ (244 mg, 0.750 mmol, 2.5 equiv.), (153 mg, 0.600 mmol, 2.0 equiv.), and 4-iodo-1,1'-biphenyl (84.0, 0.300 mmol, 1.0 equiv.). To the solids was sequentially added a magnetic stirrer bar and anhydrous DMSO (1.5 mL). The reaction mixture was sparged with $N_2$ for 15 minutes before being sealed with parafilm. The reaction mixture was then stirred at 80 °C in a metal heating block 4 hours. The reaction mixture was cooled to room temperature before being quenched with AcOH (0.1 mL) The reaction mixture was cooled to room temperature before being diluted with $CH_2Cl_2$ (10 mL) and water (10 mL). The organic phase was collected, and the aqueous phase was extracted with $CH_2Cl_2$ (3 × 10 mL). The organics were combined, washed with brine (25 mL), dried ($MgSO_4$), and concentrated under reduced pressure. The crude product was then purified by column chromatography (1% $Et_2O$ in pentane) to afford **94** as a white solid (51.2 mg, 0.185 mmol, 62%).

**mp** 90–91 °C. ***R**_f_* 0.43 (1% EtOAc in pentane).

**$^1$H NMR** (500 MHz, $CDCl_3$) $\delta_H$ 7.59 (d, *J* = 7.4 Hz, 2H), 7.53 (d, *J* = 8.3 Hz, 2H), 7.46 (dd, *J* = 7.4, 7.2 Hz, 2H), 7.41 – 7.34 (m, 5H), 7.19 (d, *J* = 7.9 Hz, 2H), 2.39 (s, 3H).

**$^{13}$C NMR** (126 MHz, $CDCl_3$) $\delta_C$ 140.5 (C), 139.5 (C), 137.9 (C), 136.4 (C), 132.5 (2CH), 131.3 (C), 130.3 (2CH), 130.2 (2CH), 128.9 (2CH), 127.8 (2CH), 127.5 (CH), 127.0 (2CH), 21.3 ($CH_3$).

**HRMS** (APCI$^+$) m/z calcd. for $C_{19}H_{17}S$ $(M + H)^+$ 277.1045, found 277.1059.

**Aryl Halide Hydrodehalogenation**

The hydrodehalogenation of 4-bromobenzophenone was investigated using the ACVA-$HCO_2K$ initiator system. Pleasingly, benzophenone **96** was formed in 61% yield when 4-bromobenzophenone was reacted with ACVA (0.5 equiv.) and $HCO_2K$ (5.0 equiv.) in DMSO at 70 °C for 17 hours.

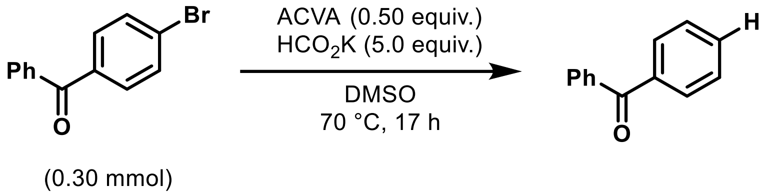


**Procedure:** To an 8 mL screw-cap vial was charged 4-4'azobis(4-cyanovaleric acid) (ACVA) (51.3 mg, 150 µmol, 0.50 equiv.), $HCO_2K$ (126 mg, 1.50 mmol, 0.50 equiv.), and (4-bromophenyl)(phenyl)methanone (78.3 mg, 0.300 mmol, 1.0 equiv.). To the solids was sequentially added a magnetic stirrer bar and anhydrous DMSO (1.5 mL). The reaction mixture was sparged with $N_2$ for 15 minutes before being sealed with parafilm. The reaction mixture was then stirred at 80 °C in a metal heating block 4 hours. The reaction mixture was cooled to room temperature before being diluted with $CH_2Cl_2$ (10 mL) and water (10 mL). The organic phase was collected, and the aqueous phase was extracted with $CH_2Cl_2$ (3 × 10 mL). The organics were combined, washed with brine (25 mL), dried ($MgSO_4$), and concentrated under reduced pressure. The crude product was then purified by column chromatography (5 → 10% $Et_2O$ in pentane) to afford **96** as a white solid (33.4 mg, 0.183 mmol, 61%).

**mp** 61–62 °C. $\boldsymbol{R_f}$ 0.56 (10% $Et_2O$ in pentane)

**$^1$H NMR** (500 MHz, $CDCl_3$) $\delta_H$ 7.81 (dd, $J$ = 7.8, 1.4 Hz, 4H), 7.59 (tt, $J$ = 7.4, 1.4, 2H), 7.49 (dd, $J$ = 7.8, 7.4 Hz, 4H).

**$^{13}$C NMR** (126 MHz, $CDCl_3$) $\delta_C$ 196.9 (C), 137.7 (2C), 132.5 (2CH), 130.2 ($4CH_2$), 128.4 ($4CH_2$).

**HRMS** (APCI$^+$) m/z calcd. for $C_{13}H_{11}O$ $(M + H)^+$ 183.0804, found 183.0806.

**Aryl Halide Borylation**

Boronic ester **97** was formed in 51% yield when 4-bromobenzophenone was reacted with bis(pinacolato)diboron ($B_2Pin_2$, 2.0 equiv.), $K_2CO_3$ (2.5 equiv.), ACVA (0.25 equiv.) and $HCO_2K$ (0.5 equiv.) in DMSO at 80 °C for 4 hours.

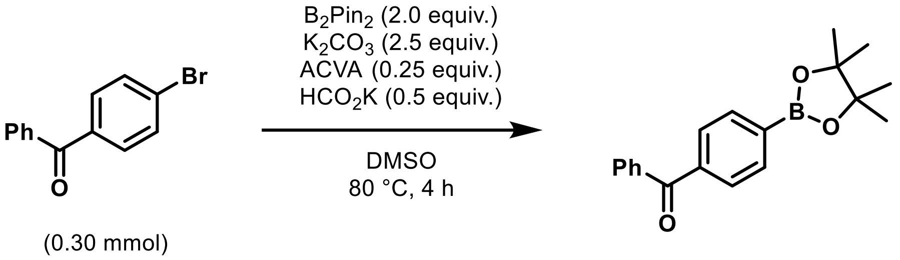


**Procedure:** To an 8 mL screw-cap vial was charged 4-4'azobis(4-cyanovaleric acid) (ACVA) (25.6 mg, 75 μmol, 0.25 equiv.), $HCO_2K$ (12.6 mg, 150 μmol, 0.50 equiv.), $K_2CO_3$ (104 mg, 0.750 mmol, 2.5 equiv.), bis(pinacolato)diboron (153 mg, 0.600 mmol, 2.0 equiv.), and (4-bromophenyl)(phenyl)methanone (78.3 mg, 0.300 mmol, 1.0 equiv.). To the solids was sequentially added a magnetic stirrer bar and anhydrous DMSO (1.5 mL). The reaction mixture was sparged with $N_2$ for 15 minutes before being sealed with parafilm. The reaction mixture was then stirred at 80 °C in a metal heating block 4 hours. The reaction mixture was cooled to room temperature before being quenched with AcOH (0.1 mL). 1,3,5-Trimethoxybenzene (50.5 mg, 0.300 mmol, 1.0 equiv.) was added to the reaction mixture, which was then analysed by $^{1}$H NMR spectroscopy to approximate the yield of **97** (51% determined by $^{1}$H NMR).

**mp** 91–93 °C. ***Rf*** 0.22 (10% $Et_2O$ in pentane).

**$^{1}$H NMR** (400 MHz, $CDCl_3$) $\delta_H$ 7.92 (d, $J$ = 8.1 Hz, 2H), 7.84 – 7.74 (m, 4H), 7.59 (t, $J$ = 7.4 Hz, 1H), 7.48 (t, $J$ = 7.6 Hz, 2H), 1.37 (s, 12H).

**$^{13}$C NMR** (101 MHz, $CDCl_3$) $\delta_C$ 197.1 (C), 139.9 (C), 137.7 (C), 134.7 (2CH), 132.7 (CH), 130.3 (2CH), 129.2 (2CH), 128.4 (2CH), 84.4 (2C), 25.0 ($4CH_3$).

Note: The quaternary $^{13}$C–B peak could not be detected due to quadropolar relaxation.

**HRMS** (APCI$^{+}$) m/z calcd. for $C_{19}H_{22}BO_3$ $(M + H)^{+}$ 309.1657, found 309.1652.

### Aryl Halide Phosphonylation

Phosphonate ester **98** was formed in 50% yield by reacting 4-bromobenzophenone with $P(OEt)_3$ (5.0 equiv.), ACVA (0.25 equiv.) and $HCO_2K$ (0.5 equiv.) in DMSO at 80 °C for 4 h.

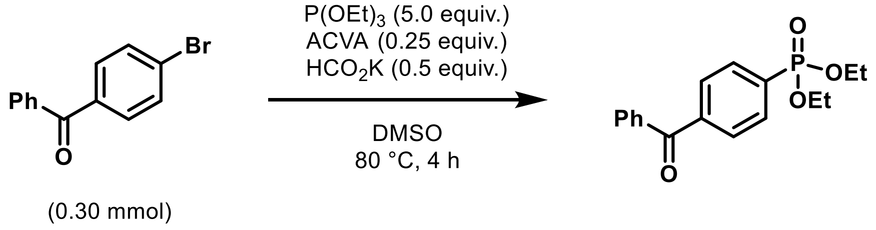


**Procedure:** To an 8 mL screw-cap vial was charged 4-4'azobis(4-cyanovaleric acid) (ACVA) (25.6 mg, 75 μmol, 0.25 equiv.), $HCO_2K$ (12.6 mg, 150 μmol, 0.50 equiv.), triethyl phosphite (249 mg, 1.50 mmol, 5.0 equiv.), and (4-bromophenyl)(phenyl)methanone (78.3 mg, 0.300 mmol, 1.0 equiv.). To the solids was sequentially added a magnetic stirrer bar and anhydrous DMSO (1.5 mL). The reaction mixture was sparged with $N_2$ for 15 minutes before being sealed with parafilm. The reaction mixture was then stirred at 80 °C in a metal heating block 4 hours. The reaction mixture was cooled to room temperature before being quenched with AcOH (0.1 mL). 1,3,5-Trimethoxybenzene (50.5 mg, 0.300 mmol, 1.0 equiv.) was added to the reaction mixture, which was then analysed by $^1$H NMR spectroscopy to approximate the yield of **98** (50% determined by $^1$H NMR).

***R<sub>f</sub>*** 0.28 (50% EtOAc in pentane).

**$^1$H NMR** (400 MHz, $CDCl_3$) $\delta_H$ 7.93 (dd, $J$ = 12.9, 8.3 Hz, 2H), 7.85 (dd, $J$ = 8.3, 4.0 Hz, 2H), 7.80 (d, $J$ = 7.8 Hz, 2H), 7.62 (t, $J$ = 7.5 Hz, 1H), 7.50 (dd, $J$ = 7.8, 7.5 Hz, 2H), 4.27 – 4.06 (m, 4H), 1.35 (t, $J$ = 7.1 Hz, 6H).

**$^{13}$C NMR** (101 MHz, $CDCl_3$) $\delta_C$ 196.1 (C), 141.1 (d, $J$ = 3.3 Hz, C), 137.0 (C), 133.2 (CH), 132.7 (d, $J$ = 187.0 Hz, C), 131.9 (d, $J$ = 10.1 Hz, 2CH), 130.3 (2CH), 129.8 (d, $J$ = 15.1 Hz, 2CH), 128.6 (2CH), 62.6 (d, $J$ = 5.5 Hz, $2CH_2$), 16.5 (d, $J$ = 6.4 Hz, $2CH_3$).

**$^{31}$P NMR** (162 MHz, $CDCl_3$) 17.15 – 16.93 (m, P).

**HRMS** (ESI$^+$) m/z calcd. for $C_{17}H_{19}NaO_4P$ $(M + Na)^+$ 341.0913, found 341.0929.

## 6. Scale Up of the Synthesis of Ethyl 2-(4-cyanophenyl)acetate

### Optimisation of Standard Procedure Prior to 50 g Scale Reaction

Based on previous optimisation results a selected set of conditions were sampled prior to scale up to ensure optimal conditions were used.

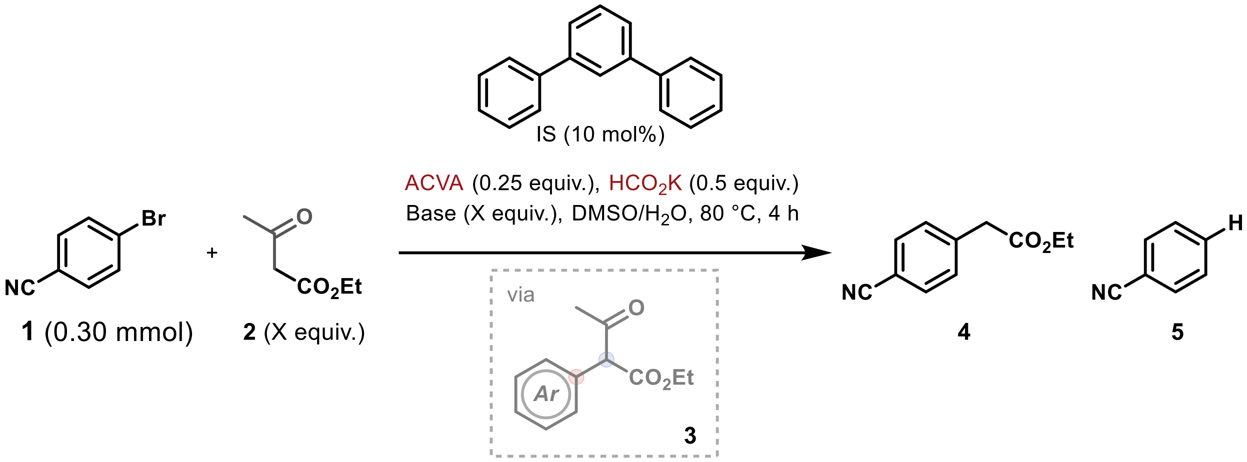


**Procedure:** To 4 mL screw cap vials was added the required solid base. To a 50 mL duran was added the other solid reagents (ACVA, $HCO_2K$, 4-cyanobenzonitrile and *meta*-terphenyl internal standard) at scale sufficient to perform 20 × 0.3 mmol reactions (6.0 mmol). These were dissolved up to 20 mL total volume with degassed DMSO to give a stock solution of 0.3 mmol/ml of 4-cyanobenzonitrile. The reagents were taken into a glovebox along with DMSO, water and ethyl acetoacetate. Teflon coated stir discs were added to the 4 mL reaction vials followed by 1 mL of stock solution. The reactions were then made to the require volume and solvent composition with DMSO and/or water. Ethyl acetoacetate was added and the vials capped then removed from the glovebox. The vials were heated to 80 °C in a tumble stirrer situated behind a blast shield for 4 hours. The reactions were cooled to room temperature then sampled for UHPLC/MS analysis (15 µL into 1.5 mL of 1:1 MeCN/water) and analysed at 220 nm.

Note: No mass corresponding to the acetylated product **3** was detected. It was assumed that deacetylation was occurring either during the reaction or UHPLC analysis.

**Table S5**. UHPLC/MS analysis at 220 nm and photos of reactions after 4 h.  Nu: nucleophile

| Reaction number | Description | Base | Base (equiv.) | ACVA (equiv.) | HCO2K (equiv.) | ethyl acetoacetate (equiv.) | IS (mol%) | Solvent | Solvent (mL) | water (mL) | Concentration (M) | Temp. | Conv. at 4h | **1** | **2** | **5** | **4** | Prod/IS at 4h |
|---|---|---|---|---|---|---|---|---|---|---|---|---|---|---|---|---|---|---|
| 1 | *Standard conditions* | $Cs_2CO_3$ | 4.5 | 0.25 | 0.5 | 4 | 10 | DMSO | 1.5 | 0 | 0.2 | 80 | 98 | 0.9 | 8.2 | 6.9 | 43.3 | 4.5 |
| 2 | *4 equiv. of base* | $Cs_2CO_3$ | 4 | 0.25 | 0.5 | 4 | 10 | DMSO | 1.5 | 0 | 0.2 | 80 | 96 | 1.9 | 2.4 | 8.6 | 46.6 | 4.8 |
| 3 | *3 equiv. Nu, 3.5 equiv. base* | $Cs_2CO_3$ | 3.5 | 0.25 | 0.5 | 3 | 10 | DMSO | 1.5 | 0 | 0.2 | 80 | 97 | 1.3 | 5.5 | 9.1 | 44.1 | 4.5 |
| 4 | *3 equiv. Nu, 3.0 equiv. base* | $Cs_2CO_3$ | 3 | 0.25 | 0.5 | 3 | 10 | DMSO | 1.5 | 0 | 0.2 | 80 | 88 | 5.9 | 0.0 | 14.1 | 42.1 | 3.9 |
| 5 | *2.5equiv. Nu, 3.0 equiv. base* | $Cs_2CO_3$ | 3 | 0.25 | 0.5 | 2.5 | 10 | DMSO | 1.5 | 0 | 0.2 | 80 | 92 | 3.6 | 1.3 | 12.6 | 44.0 | 4.3 |
| 6 | *14:1 DMSO/Water* | $Cs_2CO_3$ | 4.5 | 0.25 | 0.5 | 4 | 10 | DMSO | 1.4 | 0.1 | 0.2 | 80 | 71 | 12.9 | 11.5 | 6.5 | 30.9 | 2.6 |
| 7 | *6.5:1 DMSO/Water* | $Cs_2CO_3$ | 4.5 | 0.25 | 0.5 | 4 | 10 | DMSO | 1.3 | 0.2 | 0.2 | 80 | 47 | 23.7 | 17.3 | 5.8 | 21.1 | 1.5 |
| 8 | *2:1 DMSO/Water* | $Cs_2CO_3$ | 4.5 | 0.25 | 0.5 | 4 | 10 | DMSO | 1 | 0.5 | 0.2 | 80 | 4 | 59.7 | 3.3 | 2.7 | 2.3 | 0.1 |
| 9 | *Standard conditions* | $K_3PO_4$ | 4.5 | 0.25 | 0.5 | 4 | 10 | DMSO | 1.5 | 0 | 0.2 | 80 | 64 | 19.2 | 3.0 | 5.4 | 33.8 | 2.7 |
| 10 | *4 equiv. of base* | $K_3PO_4$ | 4 | 0.25 | 0.5 | 4 | 10 | DMSO | 1.5 | 0 | 0.2 | 80 | 58 | 22.6 | 2.4 | 5.7 | 31.3 | 2.4 |
| 11 | *3 equiv. Nu, 3.5 equiv. base* | $K_3PO_4$ | 3.5 | 0.25 | 0.5 | 3 | 10 | DMSO | 1.5 | 0 | 0.2 | 80 | 61 | 20.9 | 1.9 | 7.2 | 32.3 | 2.5 |
| 12 | *3 equiv. Nu, 3.0 equiv. base* | $K_3PO_4$ | 3 | 0.25 | 0.5 | 3 | 10 | DMSO | 1.5 | 0 | 0.2 | 80 | 63 | 19.4 | 1.1 | 9.3 | 32.5 | 2.5 |
| 13 | *2.5equiv. Nu, 3.0 equiv. base* | $K_3PO_4$ | 3 | 0.25 | 0.5 | 2.5 | 10 | DMSO | 1.5 | 0 | 0.2 | 80 | 64 | 18.9 | 0.9 | 10.7 | 32.9 | 2.5 |
| 14 | *14:1 DMSO/Water* | $K_3PO_4$ | 4.5 | 0.25 | 0.5 | 4 | 10 | DMSO | 1.4 | 0.1 | 0.2 | 80 | 57 | 20.7 | 7.8 | 6.2 | 27.8 | 2.1 |
| 15 | *6.5:1 DMSO/Water* | $K_3PO_4$ | 4.5 | 0.25 | 0.5 | 4 | 10 | DMSO | 1.3 | 0.2 | 0.2 | 80 | 40 | 31.3 | 6.9 | 5.8 | 20.7 | 1.4 |
| 16 | *2:1 DMSO/Water* | $K_3PO_4$ | 4.5 | 0.25 | 0.5 | 4 | 10 | DMSO | 1 | 0.5 | 0.2 | 80 | 6 | 58.9 | 2.3 | 2.9 | 3.9 | 0.2 |

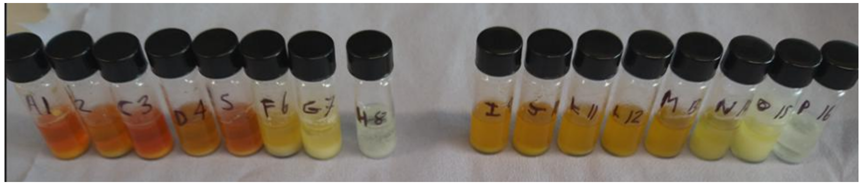

**Time Course Analysis of Entries 1 and 3 from Table S5.**

To ensure equal performance of entry 3 (**Table S5**) compared to the standard conditions, and better understand the end of reaction point, the reaction progress was monitored via uHPLC/MS.

**Entry 1:** To a 4 mL screw-cap vial was charged ACVA (42.5 mg, 0.15 mmol, 0.25 equiv.), $HCO_2K$ (25.2 mg, 0.30 mmol, 0.50 equiv.), $Cs_2CO_3$ (880 mg, 2.7 mmol, 4.5 equiv.), *meta*-terphenyl internal standard (13.8 mg, 0.06 mmol, 0.1 equiv.) and 4-bromobenzonitrile (109 mg, 0.60 mmol, 1.0 equiv.). The vial was loosely caped and taken into a glovebox. A Teflon coated stir disc was added followed by DMSO (3.0 mL) and ethyl acetoacetate (305 µL, 2.4 mmol, 4.0 equiv.). A sample was taken at T = 0 then the reaction heated to 80 °C and sampled over the course of 250 min (~4 h).

**Entry 3:** To a 4 mL screw-cap vial was charged ACVA (42.5 mg, 0.15 mmol, 0.25 equiv.), $HCO_2K$ (25.2 mg, 0.30 mmol, 0.50 equiv.), $Cs_2CO_3$ (684 mg, 2.1 mmol, 3.5 equiv.), *meta*-terphenyl internal standard (13.8 mg, 0.06 mmol, 0.1 equiv.) and 4-bromobenzonitrile (109 mg, 0.60 mmol, 1.0 equiv.). The vial was loosely caped and taken into a glovebox. A Teflon coated stir disc was added followed by DMSO (3.0 mL) and ethyl acetoacetate (229 µL, 1.8 mmol, 3.0 equiv.). A sample was taken at T = 0 then the reaction heated to 80 °C and sampled over the course of 250 min (~4 h).

The area% of the starting material **1** and product **4** divided by area of internal standard was normalised to the quantity of internal standard at T = 0.

Conditions from entry 3 (3 equiv. Nu, 3.0 equiv. base) were chosen for scale up as there appeared minimal difference in either the endpoint reaction analysis or rate of the reaction.

**Table S6**. Reaction time course data

| Time (min) | Entry 1 product area/IS area | Entry 1 starting material area / IS area | Entry 3 product area/IS area | Entry 3 starting material area/IS area |
|---|---|---|---|---|
| 0 | 9.13 | 0.00 | 8.48 | 0.00 |
| 5 | 8.47 | 0.64 | 7.97 | 1.00 |
| 10 | 8.05 | 1.27 | 7.83 | 2.57 |
| 15 | 6.77 | 3.01 | 7.05 | 3.79 |
| 20 | 6.60 | 4.22 | 6.33 | 4.92 |
| 25 | 5.94 | 5.27 | 5.71 | 5.88 |
| 30 | 5.35 | 6.23 | 5.19 | 6.80 |
| 45 | 3.58 | 8.28 | 3.78 | 8.83 |
| 60 | 2.76 | 9.87 | 2.80 | 9.95 |
| 81 | 1.76 | 11.37 | 1.71 | 10.48 |
| 95 | 1.35 | 11.90 | 1.41 | 11.51 |
| 120 | 0.89 | 12.72 | 0.99 | 12.59 |
| 187 | 0.36 | 13.88 | 0.38 | 12.86 |
| 257 | 0.16 | 13.50 | 0.20 | 12.34 |

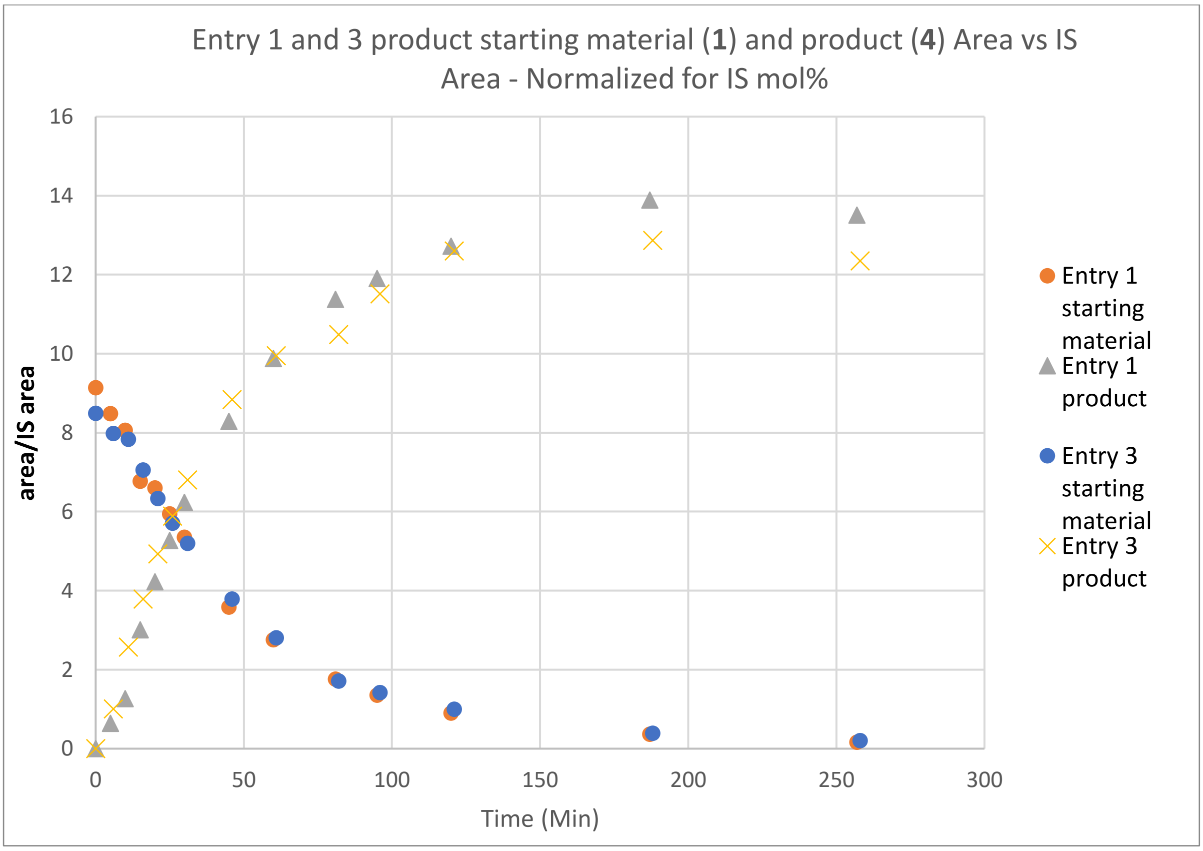


**Fig. S9.** Reaction time course graphical representation

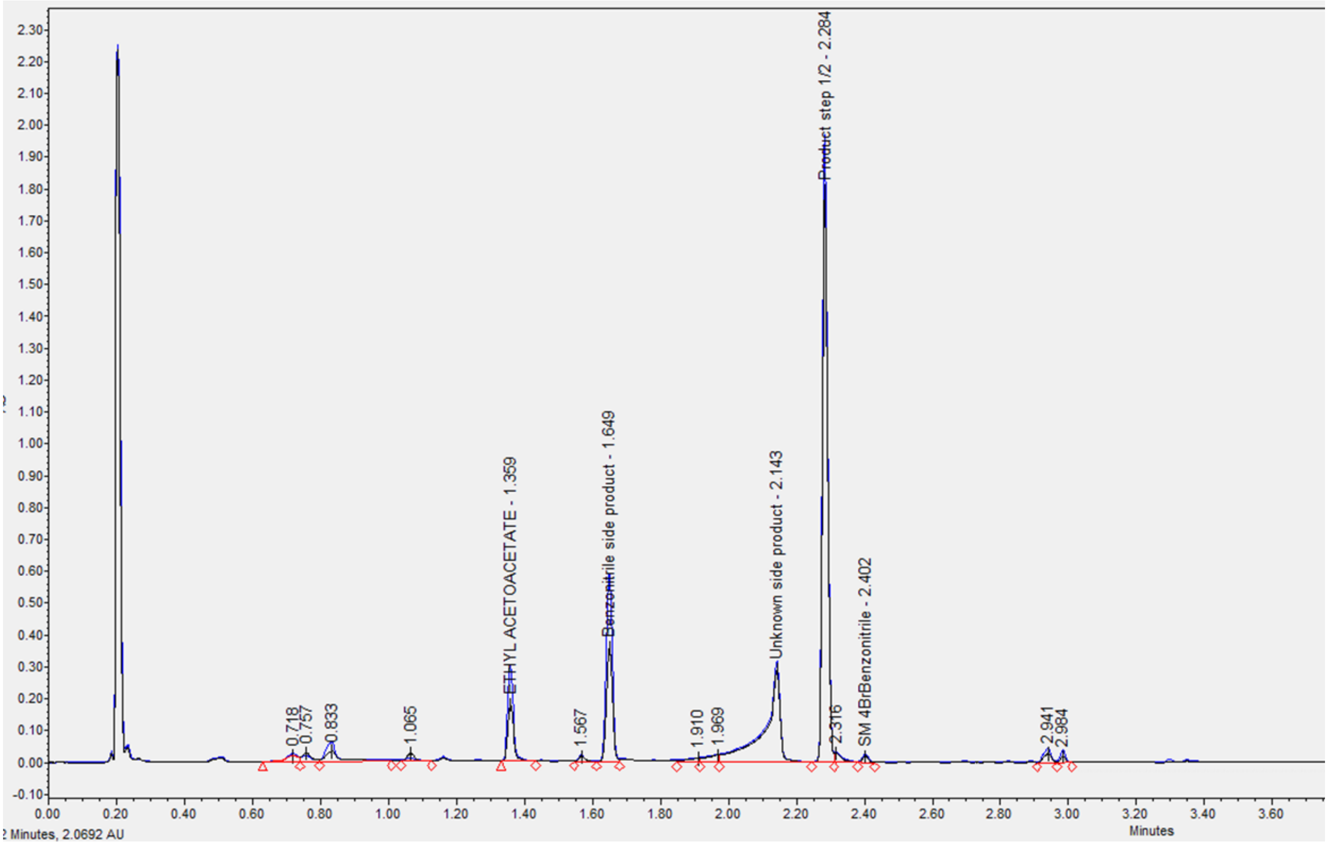


**Fig. S10**. Overlay of the T = 257 min time point for entry 1 and 3.

The unknown broad signal at t = 2.14 min could not be assigned by mass analysis, but it is thought to originate from the potential dimerization of ethyl acetoacetate under the reaction conditions.

**Process Safety Assessment Prior to 50 g Scale Reaction**

This information is supplied to facilitate the development of your programmes and processes for ensuring the safety of your workers or physical assets. AstraZeneca's provision of this information should, in no way, be interpreted as an express or implied warranty of its accuracy or completeness or as otherwise affecting your independent duty to develop and implement a reliable and effective safety programme.

**Explosive Screening of AZ11973086 (ACVA, 4,4'-Azobis(4-cyanovaleric acid))**

High Rate Carius Tube (HRCT) testing indicated that (ACVA, 4,4'-Azobis(4-cyanovaleric acid)) (97.4% w/w) as provided is not a potential explosive, however the compound still decomposes energetically and should be treated/handled appropriately.

A positive result in the fallhammer at 50 J indicates that AZ11973086 (ACVA, 4,4'-Azobis(4-cyanovaleric acid)) (97.4% w/w) should not be subjected to high energy operations, e.g. milling, micronisation, etc.

The negative result at 10 J however, indicates that an explosion should not be triggered during normal operations involving the handling of this material, e.g. weighing and charging.

Any change in purity and/or the impurity profile could have the potential to change the enthalpy and/or rate of decomposition and would require a reassessment of the potential hazard.

| Experiment | **Results** | **Cnclusions** |
|---|---|---|
| **Substance: (ACVA, 4,4'-Azobis(4-cyanovaleric acid)) (97.4% w/w)** | | |
| Differential Scanning Calorimetry Test | DSC test showed a large exotherm (615 J/g) from 98°C to 173°C followed by an exotherm (289 J/g) from 199°C to 342°C. | The heat of decomposition for this sample is above the 800 J/g threshold value,[65] thus indicating that the material may have explosive properties.<br><br>A Yoshida plot of the DSC test data indicates that the material is a potential explosive and has a high risk of being shock sensitive.<br><br>Hence, further Fallhammer and High Rate Carius Tube tests were carried out |
| Fallhammer Test | Fallhammer test @ 50 J<br><br>Positive<br><br>8 positive (bangs) out of 8 trials.<br><br>Fallhammer test @ 10 J<br><br>Negative<br><br>No bangs, flame or sparking was observed out of 8 trials, however some decomposition may have occurred. | A positive result in the Fallhammer at 50 J indicates that (ACVA, 4,4'-Azobis(4-cyanovaleric acid)) (97.4% w/w) should not be subjected to high energy operations, e.g. milling, micronisation, etc. However, the negative result at 10 J indicates that an explosion should not be triggered during normal operations involving the handling of this material, e.g. weighing and charging.<br><br>Any change in purity and/ or the impurity profile could have the potential to change the enthalpy and/or rate of decomposition and would require a reassessment of the potential hazard |
| High Rate Carius Tube Test | The results of experiment - Run 1<br><br>Maximum pressure achieved = 194 psig at 239°C. | The test results on this sample indicate that the material does not have explosive properties.<br><br>Any change in purity and/or the impurity profile could have the potential to change the rate of decomposition and would require a reassessment of the potential hazard. |

## Differential Scanning Calorimetry Test

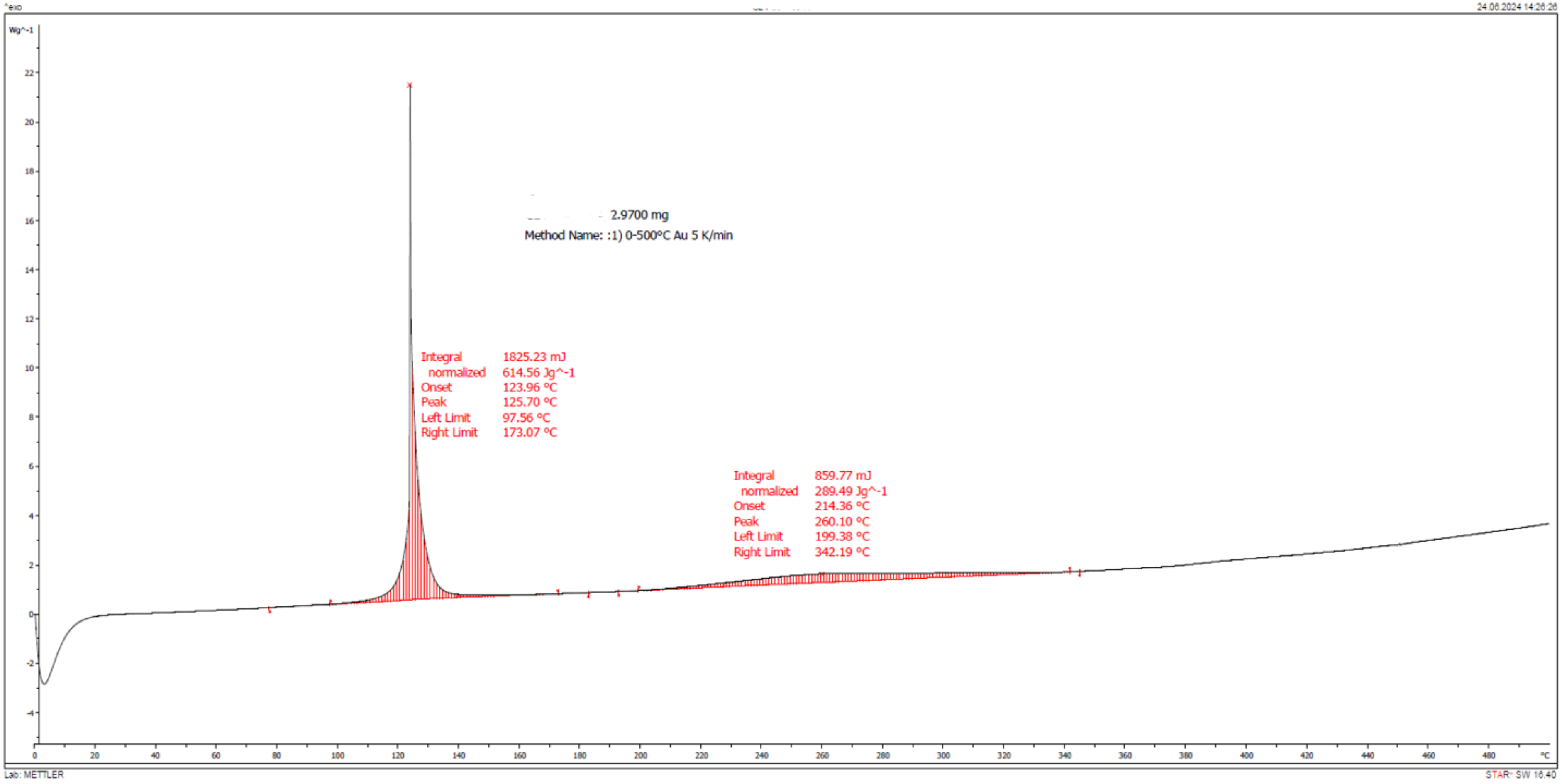

**High Rate Carius Tube Test**

(ACVA, 4,4'-Azobis(4-cyanovaleric acid)) 97.4% w/w by NMR. Sigma Aldrich 11590-25G, Lot BCCJ6327

| | |
|---|---|
| **Ramp rate** | 2K/min |
| **Temperature cutout** | 350°C |
| **Pressure cutout** | 500psig |
| **Scale** | 3 g |

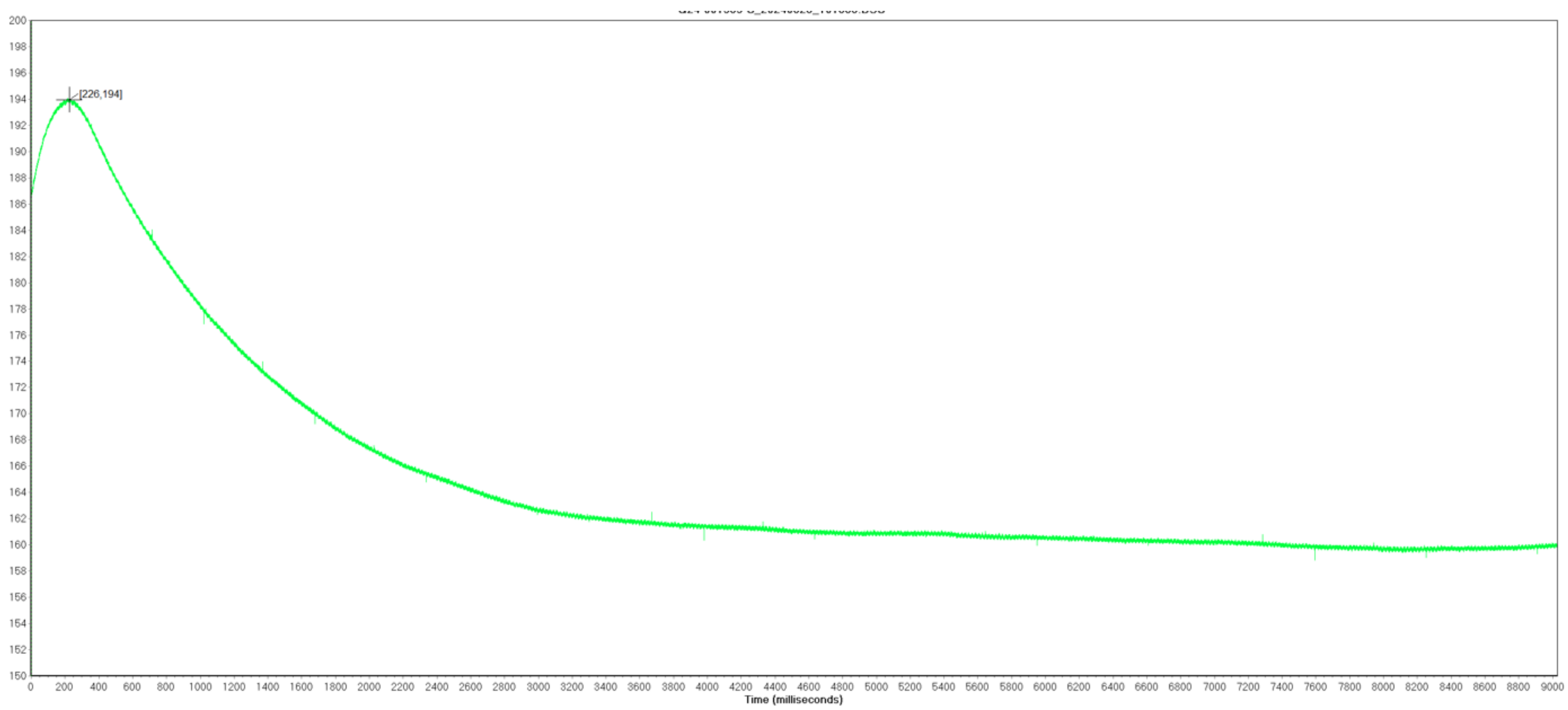

[226,194]
Time (milliseconds)

**Safety Assessment of the Standard Procedure**

**Use of DMSO**

DMSO, like other aprotic solvents is often not inert to reagents. DMSO is well documented in the literature as an energetic solvent; it decomposes with a significant release of heat and gas thus presenting a significant thermal hazard, especially when mixed with a range of other reagents.[66] The decomposition reaction is evident at the boiling point; DMSO cannot be distilled at ambient pressure without decomposition. This decomposition is self-accelerating and is catalysed by acid and particularly with organic or inorganic halogens (even trace amounts). In some cases, the reaction is virtually instantaneous whilst others are characterized by the steady accumulation of heat and pressure with eventual runaway. Caution is therefore necessary when using any untested combinations of DMSO and reagents. The most commonly stated incompatibilities are acidic materials and acid chlorides although some safety data sheets also document instability with strong base (see Brethericks Handbook of Reactive Chemical Hazards 8th Edition. Entry 0917 for more details).

**Proposed Process**

The onset of the autocatalytic decomposition of DMSO was detected at a lower temperature of 145°C in the all-in reaction mixture than seen in neat DMSO at 161°C.

Tsafe for the all-in reaction on the proposed laboratory scale is 128°C (TNR-10 K – Exotherm contribution).

Using an assumed 400 kJ/mol 4-bromobenzonitrile for the heat of the desired reaction gives a calculated adiabatic rise of ~31 K which would not access the 128°C Tsafe from the 80°C process temperature.

Nitrogen gas evolution is expected from the thermal decomposition of the ACVA as part of the desired reaction. Carbon dioxide evolution is also expected due to the presence of caesium carbonate and potassium formate in the reaction mixture. The reactor should remain vented throughout.

**General Considerations for Scale Up**

All in reactions are inherently unsafe and thus if changes to the reaction are made further safety assessment should be made prior to scale up.

Due to the known autocatalytic decomposition reaction of DMSO it would be prudent to avoid prolonged holding beyond the proposed 4 hours at 80 °C and avoid temperature cycling of the reaction mixture (cooling and reheating).

The limited initial investigation carried out and detailed in this report indicates that it may be possible to further scale up the proposed reaction, however, the reaction should be further assessed before scale up beyond the general laboratory scale (<2 litres).

Given the autocatalytic nature of the DMSO decomposition and the presence of an energetic azo material it would be prudent to employ suitable precautions on the laboratory scale, for example, the use of blast screens.

**Test Results**

**Reaction Heat**

Calculation indicated that the initial exotherm observed from 53 °C in the C80 test would equate to -247 kJ/mol 4-bromobenzonitrile. If it is assumed that this is due to the desired reaction then using a more conservative figure indicates that an assumed exotherm of 400 kJ/mol 4-bromobenzonitrile would give a ~31 K adiabatic rise which from the 80 °C process temperature would not access the decomposition exotherm, Tsafe 128 °C, on the laboratory scale (<2 litres). However, DMSO is known to be sensitive to trace impurities and to aging when held for a prolonged time at higher temperatures or temperature cycled. It would therefore be prudent to avoid prolonged holding beyond the proposed 4 hours at 80 °C or cooling and reheating the reaction.

**Gas Evolution**

Nitrogen gas evolution is expected from the thermal decomposition of the ACVA. Also, carbon dioxide will be produced from reaction and/or thermal decomposition of the caesium carbonate and potassium formate present in the reaction mixture. Although gas evolution was not measured calorimetrically for the process, C80 test indicates gas onset from 58 °C which will be accessed during the heat up to 80°C and likely to be associated with the desired reaction.

**Thermal Stability**

The C80 test showed an initial low exotherm from 53 °C to 132 °C (35 J/g) and gas evolution from 58 °C, which are likely to be associated with the desired reaction. That was followed by a large exotherm from 145 °C which was incomplete at the test end at 249 °C (>555 J/g) with further gas evolution from 151 °C, and likely due to decomposition of the reaction mixture.

Overlay of a previously run trace for the decomposition of neat DMSO showed that the onset of the exotherm in this all-in reaction sample was at a lower temperature of 145 °C vs 161 °C but that the Tsafe on the scale, calculated as 128 °C will not be accessed at the proposed reaction temperature.

| Material/ Operation | Reaction Heat/Gas Generation | Thermal Stability | Comments |
|---|---|---|---|
| All-in reaction heated to 80 °C and held for 4 hours | The heat of reaction for the all-in reaction was not measured, however the ramped thermal stability C80 test indicated that the desired reaction releases at least -247 kJ/mol 4-bromobenzonitrile and is accompanied by gassing (gas onset from 58 °C).<br><br>Calculation using an assumed conservative heat of reaction of 400 kJ/mol 4-bromobenzonitrile gave an adiabatic rise of ~31 K.<br><br>Adiabatic temperature rise will not access boiling point of batch (DMSO bpt = 189 °C) from the 80 °C operating temperature.<br><br>Nitrogen gas evolution is expected from the thermal decomposition of the ACVA to give the active free radical initiator species for the reaction. Also, carbon dioxide may also be produced from reaction and/or thermal decomposition of the caesium carbonate present in the reaction mixture. Although gas evolution was not measured calorimetrically for the process, C80 test indicates gas onset from 58 °C which will be accessed during the heat up to 80 °C and likely to be associated with the desired reaction. | C80 test showed an exotherm from 53 °C to 132 °C (34.9 J/g) followed by an exotherm from 145 °C to the test end at 249°C (>555 J/g) with gas evolution from 58 °C (desired reaction) and further gas evolution from 151 °C.<br><br>Mixture will be thermally stable up to 128 °C (Tsafe) which is below the boiling point of the reaction solvent (DMSO Bpt 189 °C). | The reaction should be heated to 80 °C in a controlled manner, with good agitation, at a rate commensurate with vent disengagement capacity. In the event of agitator failure heating of the batch should be stopped and not restarted until normal operating conditions have been restored.<br><br>The reactor should remain vented throughout.<br><br>Due to the known autocatalytic decomposition reaction of DMSO it would be prudent to avoid prolonged hold times in excess of the proposed 4 hours at the 80 °C process temperature and avoid temperature cycling of the reaction mixture.<br><br>All-in reactions are inherently unsafe and thus the concentration should not be changed without input from the Chemical Reaction Hazards group. |

## C80 Experimental results

| | | | |
|---|---|---|---|
| SETARAM Instrumentation KEP Technologies | **Figure :** 1<br>25/06/2024 | **Experiment :**<br>**Procedure :** 30 - 260 0.5 K/min 25/06/24 - Modified | **Atmosphere :** 1:Air<br>**Mass :** 985.96 (mg) |
| C80 CS Evol. (No option) | | **Zone name :** 2 Standard zone | **Molar mass :** - admin |

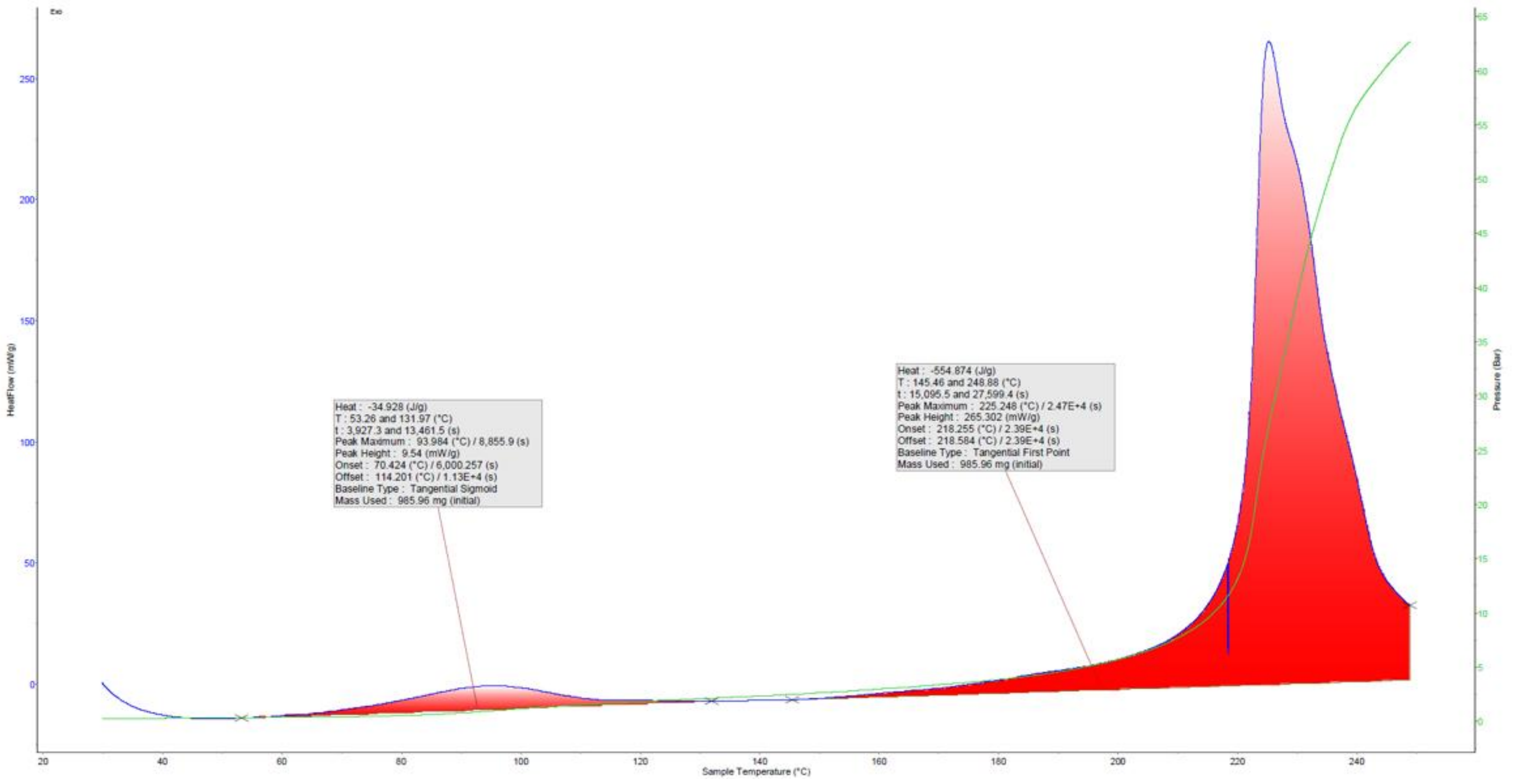

## 50 g Scale Procedure

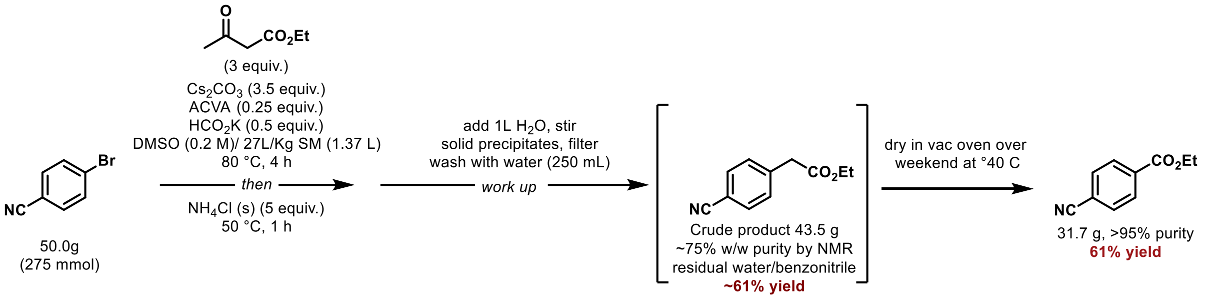


Reactor 1 is a Radleys reactor ready 5 L fitted with a nitrogen inlet, condenser, $O_2$ sensor, overhead stirrer and bottom run off valve (BRO) situated behind a blast shield in a walk-in fume hood (**Fig. S11a**).

DMSO was degassed separately via $N_2$ bubbling for 6 hours prior to the reaction. $Cs_2CO_3$ is known to cause reaction variability due to particle size. $Cs_2CO_3$ used in this reaction was purchased from Fluorochem (F050215) and used as supplied.

To reactor 1 was added the solid reagents via an addition port; 4-bromobenzonitrile (50.0 g, 275 mmol, 1.0 equiv.), ACVA (19.3 g, 68.7 mmol, 0.25 equiv.), $HCO_2K$ (11.6 g, 137 mmol, 0.50 equiv.) and $Cs_2CO_3$ (313 g, 962 mmol, 3.5 equiv.). DMSO (1375 mL, 27 L/Kg limiting reagent) was added via the solid addition port to wash in residual solids. The overhead stirrer was set to 250 rpm to suspend the solids in the reaction solvent. Ethyl acetoacetate (107 g, 823 mmol, 3.0 equiv.) was weighed out as a liquid and added via the solid addition port, then washed in with 10 mL of DMSO. The colourless heterogenous reaction (**Fig. S11b**) was sampled (ensuring liquid and solids were sampled) for T = 0 then heated to an internal temperature set point of 80 °C. After 4 hours, uHPLC/MS analysis indicated <3% of the starting material remained and the deep orange heterogenous reaction was cooled to 50 °C (**Fig. S11c**). Ammonium chloride (73.5 g, 137 mmol, 5.0 equiv.) was added and the reaction stirred for 1 h upon which time uHPLC/MS analysis showed no change in the reaction profile. The reaction was cooled to 25 °C and stirred overnight. To the reaction was slowly added water (500 mL) causing an exotherm to 35 °C. Further water (500 mL) was added causing the reaction to become a homogenous orange solution that subsequently formed a white precipitate upon cooling to ambient temperature (**Fig. S11d**). This heterogenous reaction mixture was stirred for 3 h upon which uHPLC/MS analysis indicated the solution phase had minimal product remaining. The reaction mixture was drained through the BRO and filtered through a split Buchner funnel into a receiving flask (**Fig. S11e**). Water (250 mL) was added to the

reactor to wash out the remaining solids onto the filter cake which was pulled dry then the solids (43.4 g, 75% NMR assay, reaming mass balance DMSO/Water) (**Fig. S11f**) were transferred to a vacuum oven at 40 °C for 48 hours. This provided 31.7 g, >98% NMR assay, >95% area/area% uHPLC at 220 nm, 61% yield. Data in accordance with that previously reported.

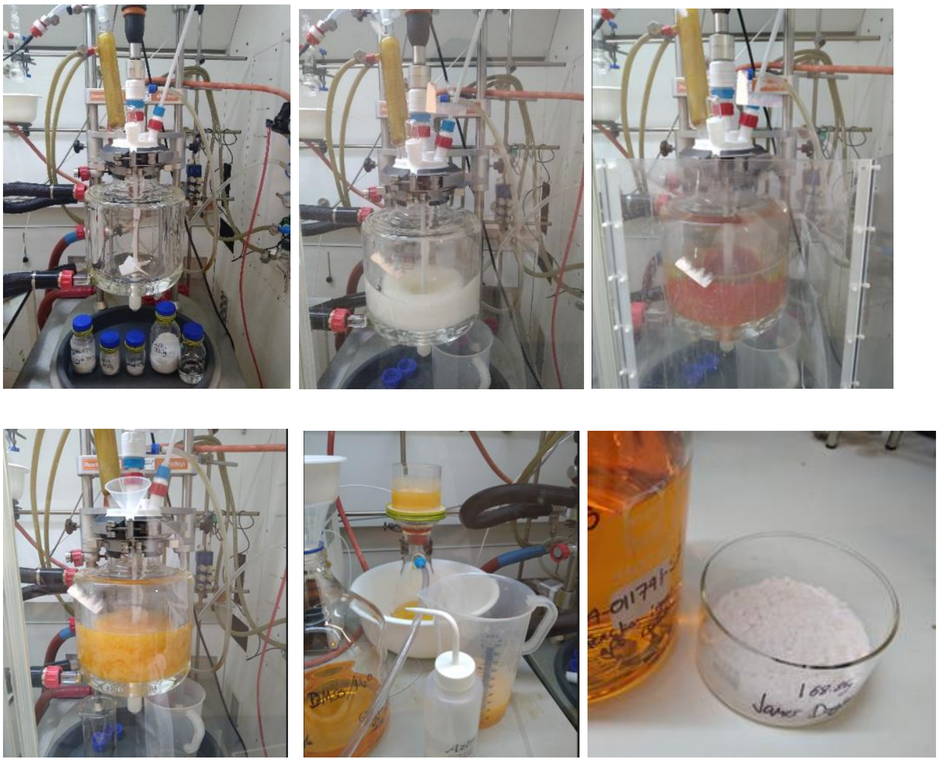

**Fig. S11.** Pictures from the 50 g scale reaction First row, L to R: a) prior to reaction; b) prior to heating; c) end of reaction. Second row, L to R: d) following water quench; e) filtration; f) filtration liquors and crude solids before drying.

**$^{1}$H NMR of ethyl 2-(4-cyanophenyl)acetate (4) prepared from 50 g scale procedure**

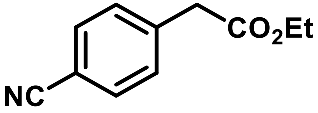


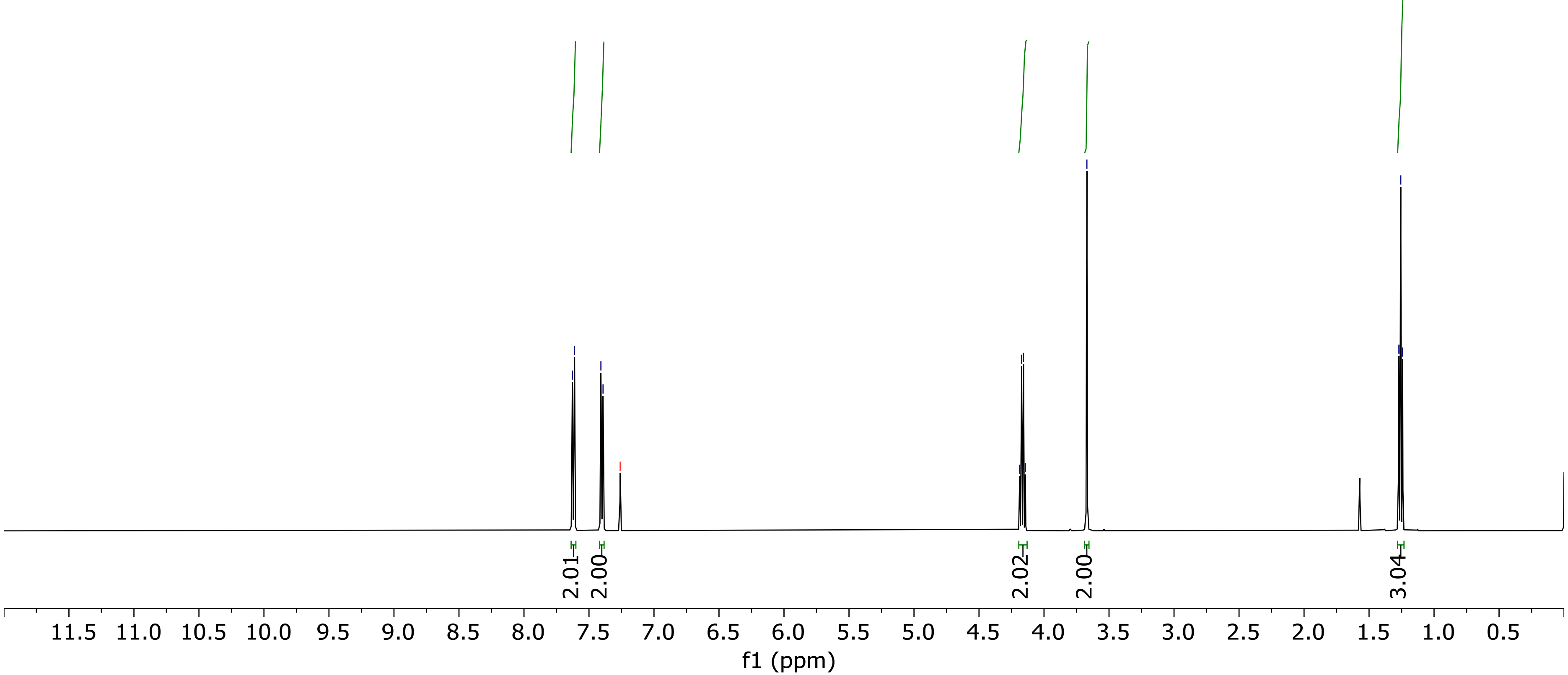

# 7. Data for Fig. 4. Microscale Parallel Screening

## List of Substrates

The reactions to assess the substrate scope were conducted on 0.1 mmol scale at 0.1 M, omitting the deacetylation step, for ease of experimental set up. Substrates were manually selected with increasing complexity, as measured by SC Score and CM (**Fig. S12**).[67,68]

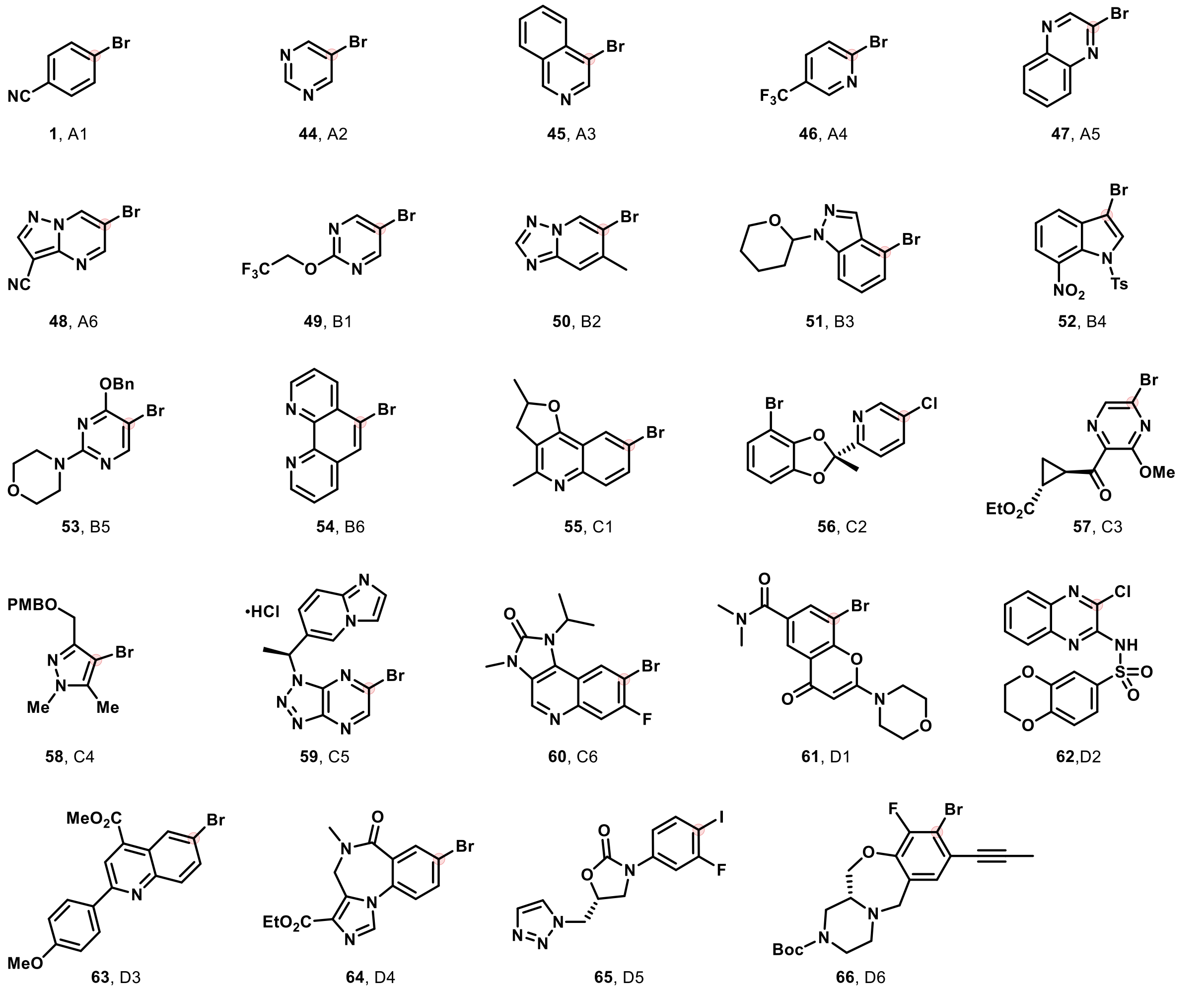


**Fig. S12**. Aryl halides chosen for microscale parallel screening.

## General Procedure

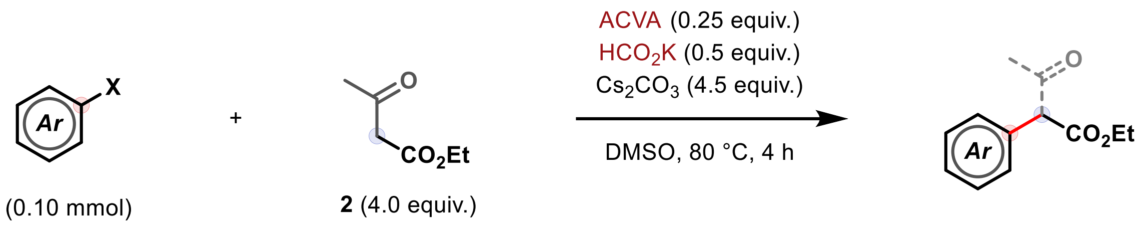


The following solids were weighed out using a Chronect XPR weighing robot to 4 mL screw top vials in the following order, $Cs_2CO_3$ (147 mg, 0.450 mmol, 4.5 equiv.), $HCO_2K$ (4.20 mg, 50 µmol, 0.50 equiv.) and ACVA (7.10 mg, 25 µmol, 0.25 equiv.). The required aryl halide (0.100 mmol) was manually weighed into the vials along with an additional 1.0 equiv. of $Cs_2CO_3$ into entry 17 (**59**) to account for the HCl salt. The vial was loosely capped and taken into a glovebox. A Teflon coated stir disc was added followed by DMSO (1.0 mL) and ethyl acetoacetate (51 µL, 0.400 mmol, 4.0 equiv.). The reactions were heated to 80 °C for 4 hours then cooled and sampled for uHPLC/MS analysis at 220 nm.

Data is reported as area% at 220 nm following MS identification of peaks, without UV response calibration, providing indicative results. The starting material standards were run independently to allow assessment of reaction conversion. As the deacylation step was omitted, some substrates returned mixture of acyl and deacylated products. In these cases, the sum of the two products was reported. Dehalogenation of the starting material was observed in most cases as a side product. In the case of **44 A2**, the dehalogenated product (pyrimidine) could not be resolved from the solvent front across multiple methods including GCMS analysis. Because of this the area% of the product is likely an overestimate. Other significant byproducts that could be assigned via MS are also listed in **Table S7**. The unknown broad peak at 2.1 min was not integrated, if observed.

**Reaction Outcomes**

Substrates B2 **(50)**, B3 **(51)**, B5 **(53)**, C1 **(55)**, C2 **(56)**, C4 **(58)**, D2 **(62)**, D6 **(66)** **–** resulted in <35% consumption of starting material.

Substrates A6 **(48)** and B4 **(52)** – led to high conversion but complex mixtures

Substrates C3 **(57)**, D3 **(63)**, D4, **(64)** – ester hydrolysis was observed of the starting material and/or the products

**Conversion**

| | | | | | |
|---|---|---|---|---|---|
| 100 | 100 | 63 | 100 | 100 | 100 |
| 100 | 34 | 30 | 99 | 29 | 97 |
| 28 | 8 | 100 | 3 | 100 | 98 |
| 81 | 19 | 91 | 100 | 94 | 10 |

**Sum of products**

| | | | | | |
|---|---|---|---|---|---|
| 72 | 82 | 41 | 64 | 35 | 13 |
| 48 | 21 | 11 | 0 | 0 | 57 |
| 0 | 0 | 57 | 0 | 55 | 32 |
| 34 | 0 | 24 | 44 | 52 | 0 |

**Sum of unknowns**

| | | | | | |
|---|---|---|---|---|---|
| 8 | 18 | 8 | 19 | 65 | 87 |
| 36 | 6 | 9 | 99 | 12 | 4 |
| 9 | 4 | 18 | 3 | 45 | 34 |
| 16 | 19 | 14 | 8 | 8 | 8 |

**Table S7**. Tabulated reaction data uHPLC/MS at 220 nm

| | | | **End of reaction area% at 220nm** | | | | | | | | | |
|---|---|---|---|---|---|---|---|---|---|---|---|---|
| **Entry** | **Complexity SCScore** | **Complexity CM*** | **Starting material** | **Dehalogenated Starting material** | **Product** | **Deacetylated Product** | **Sum of products** | **Other unknown** | **Hydrolysed product** | **Deacetylated product hydrolysis** | **Hydrolysed SM** | **Consumption of SM** |
| **A1** | 1.37 | 7.1 | 0 | 20 | | 72 | 72 | 8 | | | | 100 |
| **A2** | 1.34 | 6.26 | 0 | Not resolved | 82 | | | 18 | | | | |
| **A3** | 1.72 | 7.65 | 37 | 14 | 13 | 28 | 41 | 8 | | | | 63 |
| **A4** | 1.97 | 7.65 | 0 | 17 | 17 | 47 | 64 | 19 | | | | 100 |
| **A5** | 1.54 | 7.27 | 0 | | | 35 | 35 | 65 | | | | 100 |
| **A6** | 2.78 | 7.61 | 0 | | | 13 | 13 | 87 | | | | 100 |
| **B1** | 1.92 | 7.23 | 0 | 16 | | 48 | 48 | 36 | | | | 100 |
| **B2** | 2.84 | 7.74 | 66 | 7 | 3 | 17 | 21 | 6 | | | | 34 |
| **B3** | 3.15 | 8.69 | 70 | 10 | 11 | | 11 | 9 | | | | 30 |
| **B4** | 2.45 | 8.43 | 1 | | | | 0 | 99 | | | | 99 |
| **B5** | 2.63 | 8.69 | 71 | 17 | | | 0 | 12 | | | | 29 |
| **B6** | 2.41 | 8.16 | 3 | 37 | 11 | 46 | 57 | 4 | | | | 97 |
| **C1** | 3.08 | 8.51 | 72 | 19 | | | 0 | 9 | | | | 28 |
| **C2** | 2.66 | 8.47 | 92 | 4 | | | 0 | 4 | | | | 8 |
| **C3** | 2.56 | 8.7 | 0 | | 18 | 38 | 57 | 18 | | 26 | | 100 |
| **C4** | 2.65 | 8.46 | 97 | | | | 0 | 3 | | | | 3 |
| **C5** | 3.41 | 8.76 | 0 | | | 55 | 55 | 45 | | | | 100 |
| **C6** | 4.13 | 8.66 | 2 | 31 | | 32 | 32 | 34 | | | | 98 |
| **D1** | 3.71 | 8.76 | 19 | 32 | | 34 | 34 | 16 | | | | 81 |
| **D2** | 3.11 | 9.01 | 81 | | | | 0 | 19 | | | | 19 |
| **D3** | 2.76 | 8.69 | 9 | 18 | | 24 | 24 | 14 | 10 | | 24 | 91 |
| **D4** | 3.52 | 8.81 | 0 | 29 | | 44 | 44 | 8 | | 19 | | 100 |
| **D5** | 4.16 | 8.72 | 6 | 33 | | 52 | 52 | 8 | | | | 94 |
| **D6** | 4.04 | 9.12 | 90 | 2 | | | 0 | 8 | | | | 10 |

## uHPLC/MS data

### Reaction A1 (1)

Product observed at 2.33 min as loss of acetyl group

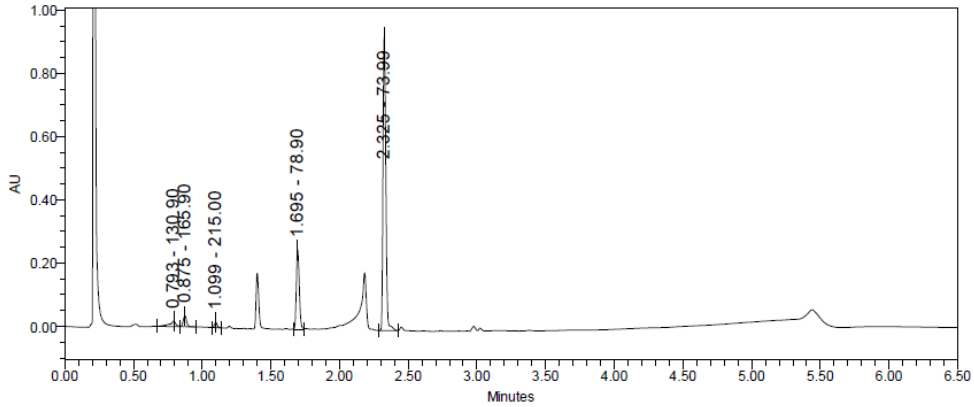


Injection Id: 8314; Result Id: 8677; Date Processed: Friday, December 6, 2024 3:51:14 PM GMT;
Processed Channel Descr. PDA Spectrum PDA 220.0 nm (PDA Spectrum (210-400)nm)

Peak Results

| | RT | % Area | Area (µV*sec) | Height (µV) | Channel |
|---|---|---|---|---|---|
| 1 | 0.793 | 2.975 | 55050 | 17630 | PDA Spectrum |
| 2 | 0.875 | 3.340 | 61806 | 34593 | PDA Spectrum |
| 3 | 1.099 | 1.198 | 22177 | 16623 | PDA Spectrum |
| 4 | 1.695 | 20.305 | 375759 | 251383 | PDA Spectrum |
| 5 | 2.325 | 72.183 | 1335811 | 928425 | PDA Spectrum |

**Reaction A2 (44)**

Product observed at 0.92 min as loss of acetyl group

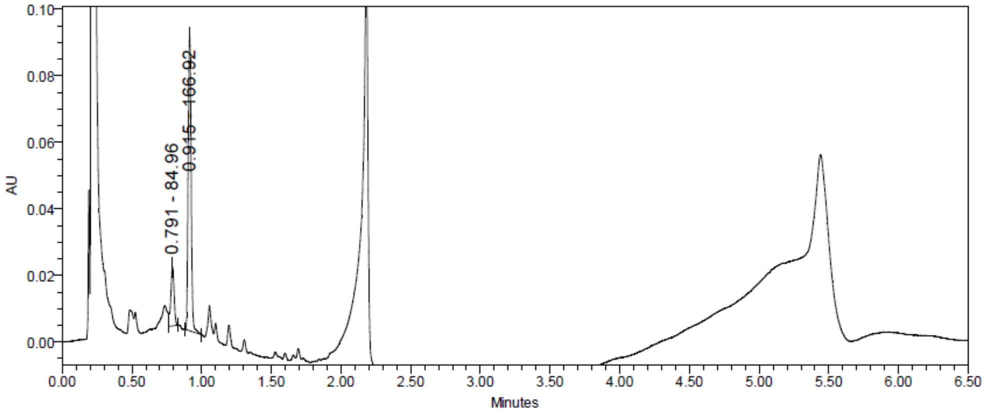


Peak Results

| | RT | % Area | Area (μV*sec) | Height (μV) | Channel |
|---|---|---|---|---|---|
| 1 | 0.791 | 18.008 | 29041 | 17670 | PDA Spectrum |
| 2 | 0.915 | 81.992 | 132231 | 88666 | PDA Spectrum |

**Reaction A3 (45)**

Product observed at 1.56 and at 1.03 min as loss of acetyl group

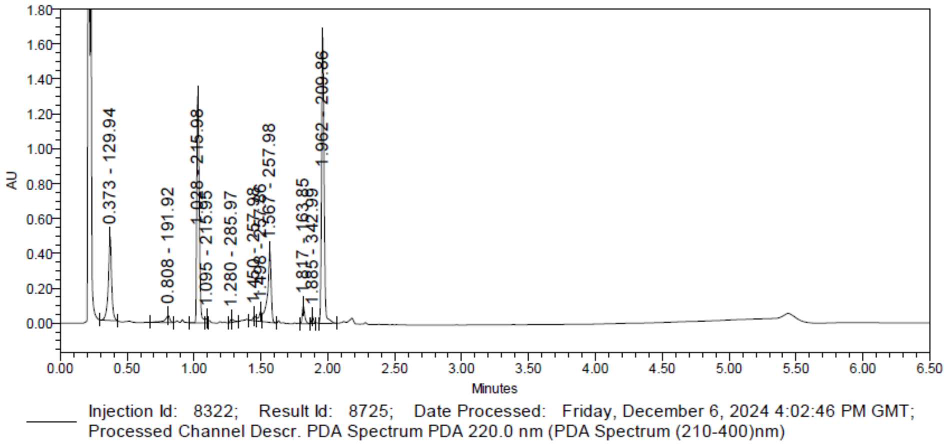


Peak Results

| | RT | % Area | Area (µV*sec) | Height (µV) | Channel |
|---|---|---|---|---|---|
| 1 | 0.373 | 13.701 | 804467 | 480848 | PDA Spectrum |
| 2 | 0.808 | 1.744 | 102409 | 39447 | PDA Spectrum |
| 3 | 1.028 | 28.233 | 1657803 | 1301838 | PDA Spectrum |
| 4 | 1.095 | 0.621 | 36440 | 25044 | PDA Spectrum |
| 5 | 1.280 | 0.571 | 33502 | 17644 | PDA Spectrum |
| 6 | 1.450 | 0.702 | 41201 | 27734 | PDA Spectrum |
| 7 | 1.498 | 1.542 | 90551 | 54551 | PDA Spectrum |
| 8 | 1.567 | 12.812 | 752294 | 407882 | PDA Spectrum |
| 9 | 1.817 | 2.266 | 133062 | 99973 | PDA Spectrum |
| 10 | 1.885 | 0.692 | 40614 | 39787 | PDA Spectrum |
| 11 | 1.962 | 37.117 | 2179429 | 1640093 | PDA Spectrum |

**Reaction A4 (46)**

Product observed at 2.69 and at 2.02 min as loss of acetyl group

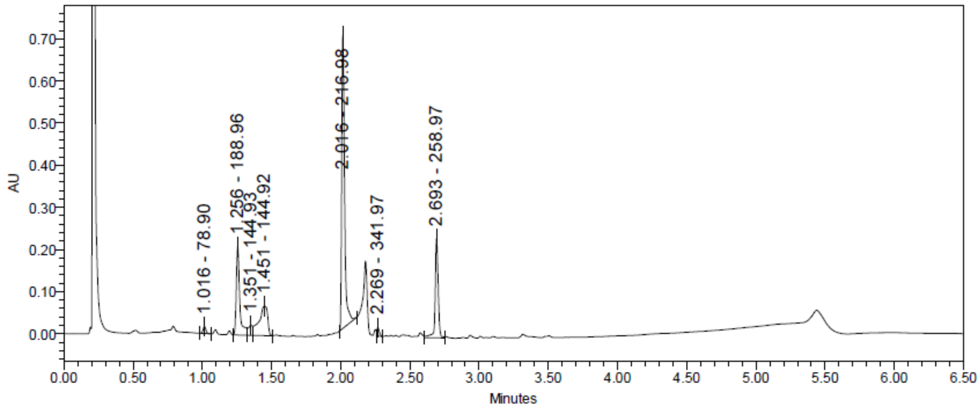


Peak Results

| | RT | % Area | Area (µV*sec) | Height (µV) | Channel |
|---|---|---|---|---|---|
| 1 | 1.016 | 1.094 | 23308 | 16198 | PDA Spectrum |
| 2 | 1.256 | 17.156 | 365488 | 209006 | PDA Spectrum |
| 3 | 1.351 | 2.506 | 53395 | 24084 | PDA Spectrum |
| 4 | 1.451 | 14.339 | 305482 | 69818 | PDA Spectrum |
| 5 | 2.016 | 46.883 | 998795 | 696827 | PDA Spectrum |
| 6 | 2.269 | 1.259 | 26812 | 20064 | PDA Spectrum |
| 7 | 2.693 | 16.763 | 357122 | 235710 | PDA Spectrum |

**Reaction A5 (47)**

Product observed at 2.35 min as loss of acetyl group

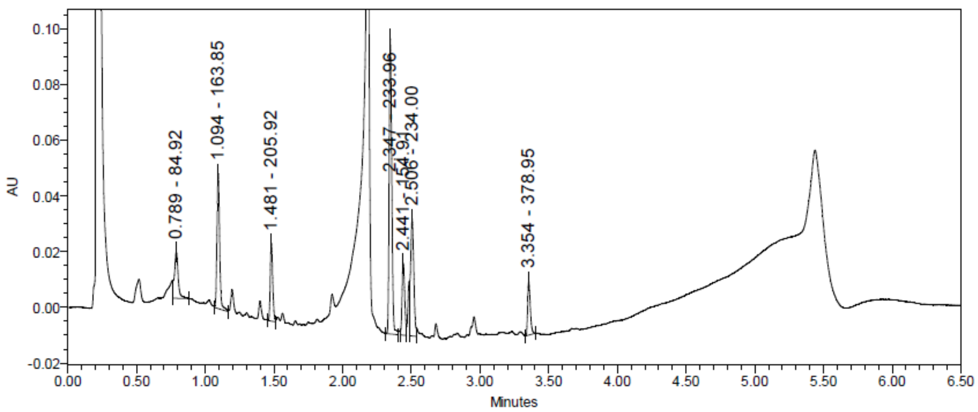


Injection Id: 8330; Result Id: 8685; Date Processed: Friday, December 6, 2024 3:51:23 PM GMT;
Processed Channel Descr. PDA Spectrum PDA 220.0 nm (PDA Spectrum (210-400)nm)

Peak Results

| | RT | % Area | Area (µV*sec) | Height (µV) | Channel |
|---|---|---|---|---|---|
| 1 | 0.789 | 7.845 | 31599 | 16625 | PDA Spectrum |
| 2 | 1.094 | 16.446 | 66247 | 48474 | PDA Spectrum |
| 3 | 1.481 | 8.647 | 34830 | 27850 | PDA Spectrum |
| 4 | 2.347 | 35.173 | 141679 | 105906 | PDA Spectrum |
| 5 | 2.441 | 8.658 | 34877 | 25795 | PDA Spectrum |
| 6 | 2.506 | 16.904 | 68089 | 42041 | PDA Spectrum |
| 7 | 3.354 | 6.327 | 25486 | 19310 | PDA Spectrum |

# Reaction A6 (48)

Product observed at 1.67 min as loss of acetyl group

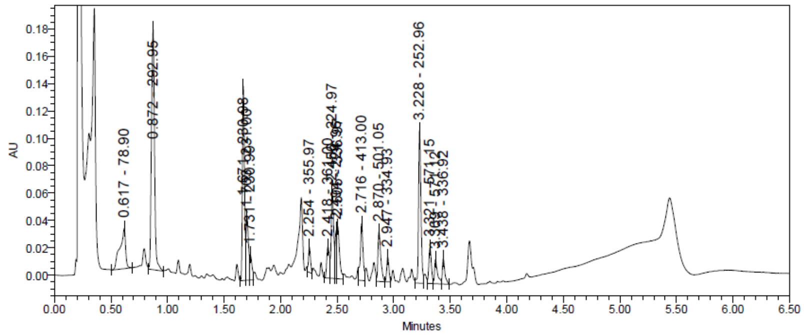


Injection Id: 8334; Result Id: 8639; Date Processed: Friday, December 6, 2024 3:50:28 PM GMT;
Processed Channel Descr. PDA Spectrum PDA 220.0 nm (PDA Spectrum (210-400)nm)

Peak Results

| | RT | % Area | Area (µV*sec) | Height (µV) | Channel |
|---|---|---|---|---|---|
| 1 | 0.617 | 7.424 | 103136 | 29474 | PDA Spectrum |
| 2 | 0.872 | 23.760 | 330090 | 175792 | PDA Spectrum |
| 3 | 1.671 | 13.031 | 181042 | 142199 | PDA Spectrum |
| 4 | 1.703 | 4.990 | 69329 | 46118 | PDA Spectrum |
| 5 | 1.731 | 1.999 | 27770 | 18730 | PDA Spectrum |
| 6 | 2.254 | 1.685 | 23403 | 18447 | PDA Spectrum |
| 7 | 2.418 | 2.795 | 38825 | 22842 | PDA Spectrum |
| 8 | 2.456 | 6.830 | 94888 | 63544 | PDA Spectrum |
| 9 | 2.494 | 2.592 | 36017 | 35644 | PDA Spectrum |
| 10 | 2.505 | 4.411 | 61275 | 38094 | PDA Spectrum |
| 11 | 2.716 | 4.888 | 67910 | 41571 | PDA Spectrum |
| 12 | 2.870 | 4.671 | 64897 | 36424 | PDA Spectrum |
| 13 | 2.947 | 1.801 | 25026 | 18118 | PDA Spectrum |
| 14 | 3.228 | 11.764 | 163435 | 111766 | PDA Spectrum |
| 15 | 3.321 | 2.976 | 41338 | 26364 | PDA Spectrum |
| 16 | 3.369 | 2.288 | 31781 | 17711 | PDA Spectrum |
| 17 | 3.438 | 2.096 | 29112 | 18591 | PDA Spectrum |

**Reaction B1 (49)**

Product observed at 2.36 min as loss of acetyl group

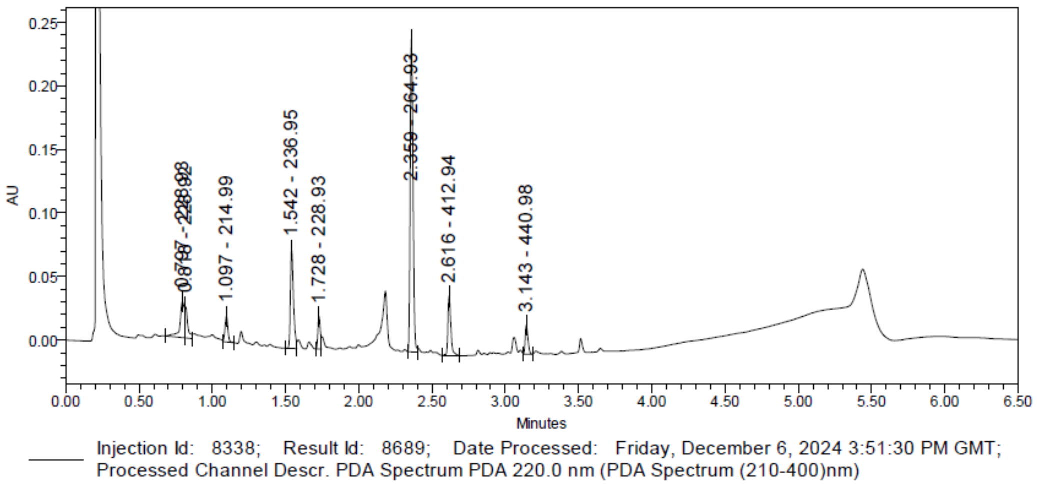


Peak Results

| | RT | % Area | Area (μV*sec) | Height (μV) | Channel |
|---|---|---|---|---|---|
| 1 | 0.797 | 8.175 | 56391 | 28272 | PDA Spectrum |
| 2 | 0.818 | 5.114 | 35278 | 24566 | PDA Spectrum |
| 3 | 1.097 | 4.007 | 27639 | 20119 | PDA Spectrum |
| 4 | 1.542 | 16.466 | 113576 | 77197 | PDA Spectrum |
| 5 | 1.728 | 4.396 | 30324 | 25525 | PDA Spectrum |
| 6 | 2.359 | 48.032 | 331310 | 246324 | PDA Spectrum |
| 7 | 2.616 | 9.503 | 65549 | 46594 | PDA Spectrum |
| 8 | 3.143 | 4.307 | 29708 | 22673 | PDA Spectrum |

## Reaction B2 (50)

Product observed at 2.03 and at 1.37 min as loss of acetyl group

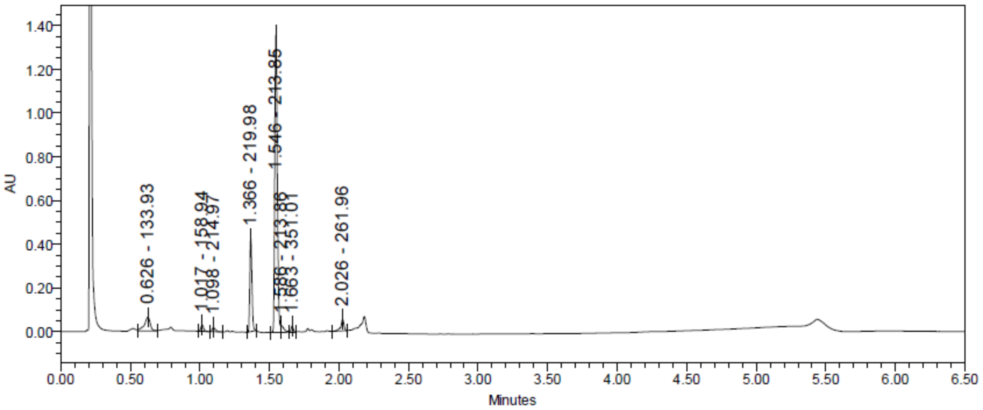


Injection Id: 8342; Result Id: 8825; Date Processed: Friday, December 6, 2024 4:23:48 PM GMT;
Processed Channel Descr. PDA Spectrum PDA 220.0 nm (PDA Spectrum (210-400)nm)

Peak Results

| | RT | % Area | Area (µV*sec) | Height (µV) | Channel |
|---|---|---|---|---|---|
| 1 | 0.626 | 7.274 | 191184 | 62790 | PDA Spectrum |
| 2 | 1.017 | 1.519 | 39932 | 29331 | PDA Spectrum |
| 3 | 1.098 | 1.242 | 32655 | 19004 | PDA Spectrum |
| 4 | 1.366 | 17.359 | 456262 | 425007 | PDA Spectrum |
| 5 | 1.546 | 66.252 | 1741356 | 1361261 | PDA Spectrum |
| 6 | 1.586 | 1.930 | 50722 | 30765 | PDA Spectrum |
| 7 | 1.663 | 1.245 | 32729 | 28654 | PDA Spectrum |
| 8 | 2.026 | 3.178 | 83523 | 53046 | PDA Spectrum |

**Reaction B3 (51)**

Product observed at 2.76 min

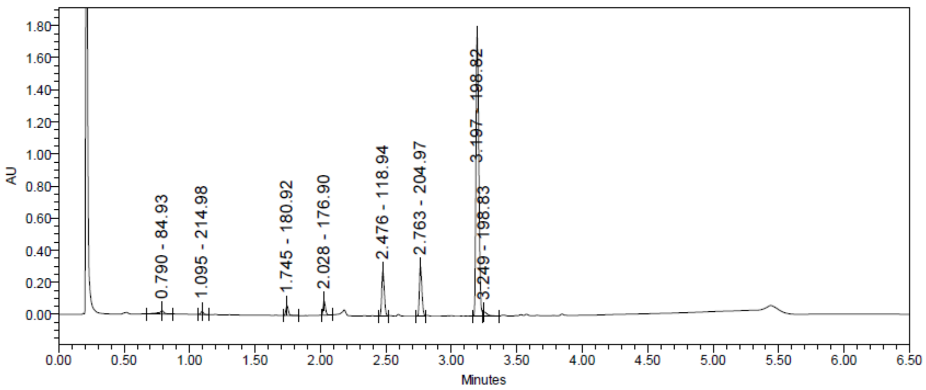


Injection Id: 8346; Result Id: 8693; Date Processed: Friday, December 6, 2024 3:51:35 PM GMT;
Processed Channel Descr. PDA Spectrum PDA 220.0 nm (PDA Spectrum (210-400)nm)

Peak Results

| | RT | % Area | Area (µV*sec) | Height (µV) | Channel |
|---|---|---|---|---|---|
| 1 | 0.790 | 1.532 | 58398 | 19158 | PDA Spectrum |
| 2 | 1.095 | 0.693 | 26402 | 19275 | PDA Spectrum |
| 3 | 1.745 | 2.151 | 81988 | 60776 | PDA Spectrum |
| 4 | 2.028 | 2.832 | 107979 | 88037 | PDA Spectrum |
| 5 | 2.476 | 10.006 | 381461 | 278932 | PDA Spectrum |
| 6 | 2.763 | 10.979 | 418559 | 309964 | PDA Spectrum |
| 7 | 3.197 | 70.290 | 2679752 | 1747886 | PDA Spectrum |
| 8 | 3.249 | 1.518 | 57858 | 25175 | PDA Spectrum |

**Reaction B4 (52)**

No product could be detected

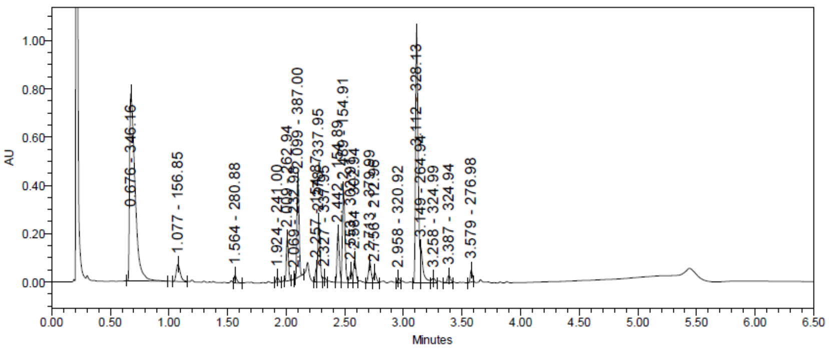

Injection Id: 8350; Result Id: 8827; Date Processed: Friday, December 6, 2024 4:27:54 PM GMT;
Processed Channel Descr. PDA Spectrum PDA 220.0 nm (PDA Spectrum (210-400)nm)

Peak Results

| | RT | % Area | Area (µV*sec) | Height (µV) | Channel |
|---|---|---|---|---|---|
| 1 | 0.676 | 34.819 | 2514027 | 777519 | PDA Spectrum |
| 2 | 1.077 | 2.401 | 173346 | 71293 | PDA Spectrum |
| 3 | 1.564 | 0.666 | 48052 | 32602 | PDA Spectrum |
| 4 | 1.924 | 0.424 | 30581 | 20240 | PDA Spectrum |
| 5 | 2.009 | 2.961 | 213755 | 178402 | PDA Spectrum |
| 6 | 2.069 | 0.004 | 283 | 739 | PDA Spectrum |
| 7 | 2.099 | 7.246 | 523144 | 405332 | PDA Spectrum |
| 8 | 2.257 | 0.603 | 43531 | 56499 | PDA Spectrum |
| 9 | 2.278 | 4.804 | 346881 | 251367 | PDA Spectrum |
| 10 | 2.327 | 0.494 | 35667 | 18887 | PDA Spectrum |
| 11 | 2.442 | 3.764 | 271762 | 204810 | PDA Spectrum |
| 12 | 2.489 | 8.054 | 581505 | 384208 | PDA Spectrum |
| 13 | 2.553 | 0.947 | 68366 | 50561 | PDA Spectrum |
| 14 | 2.584 | 1.959 | 141414 | 94612 | PDA Spectrum |
| 15 | 2.713 | 1.881 | 135818 | 86619 | PDA Spectrum |
| 16 | 2.756 | 0.985 | 71118 | 44244 | PDA Spectrum |
| 17 | 2.958 | 0.364 | 26274 | 19272 | PDA Spectrum |
| 18 | 3.112 | 21.975 | 1586644 | 1038645 | PDA Spectrum |
| 19 | 3.149 | 3.423 | 247131 | 145975 | PDA Spectrum |
| 20 | 3.258 | 0.590 | 42627 | 18225 | PDA Spectrum |
| 21 | 3.387 | 0.787 | 56858 | 28727 | PDA Spectrum |
| 22 | 3.579 | 0.851 | 61453 | 49777 | PDA Spectrum |

## Reaction B5 (53)

No product could be detected

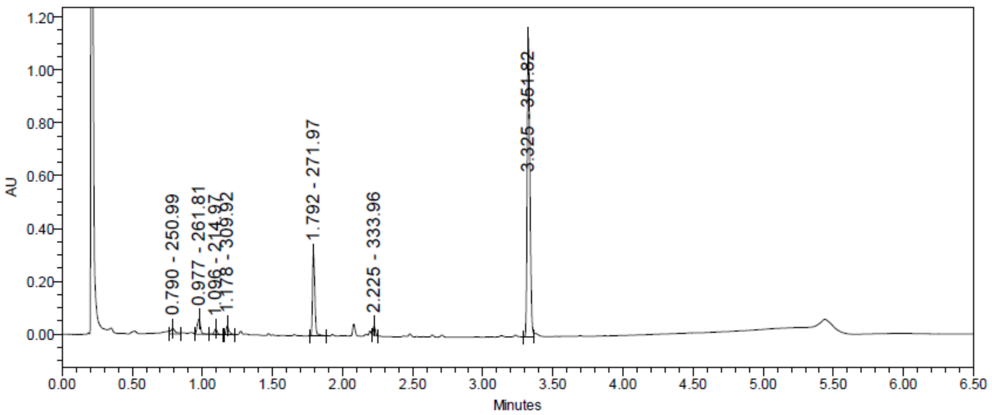


Injection Id: 8354; Result Id: 8697; Date Processed: Friday, December 6, 2024 3:51:43 PM GMT;
Processed Channel Descr. PDA Spectrum PDA 220.0 nm (PDA Spectrum (210-400)nm)

Peak Results

| | RT | % Area | Area (µV*sec) | Height (µV) | Channel |
|---|---|---|---|---|---|
| 1 | 0.790 | 2.271 | 49067 | 20410 | PDA Spectrum |
| 2 | 0.977 | 4.458 | 96323 | 58067 | PDA Spectrum |
| 3 | 1.096 | 1.441 | 31137 | 20867 | PDA Spectrum |
| 4 | 1.178 | 2.217 | 47898 | 33616 | PDA Spectrum |
| 5 | 1.792 | 16.892 | 364984 | 309988 | PDA Spectrum |
| 6 | 2.225 | 1.666 | 36003 | 34533 | PDA Spectrum |
| 7 | 3.325 | 71.055 | 1535247 | 1131764 | PDA Spectrum |

**Reaction B6 (54)**

Product observed at 1.66 and at 1.22 min as loss of acyl

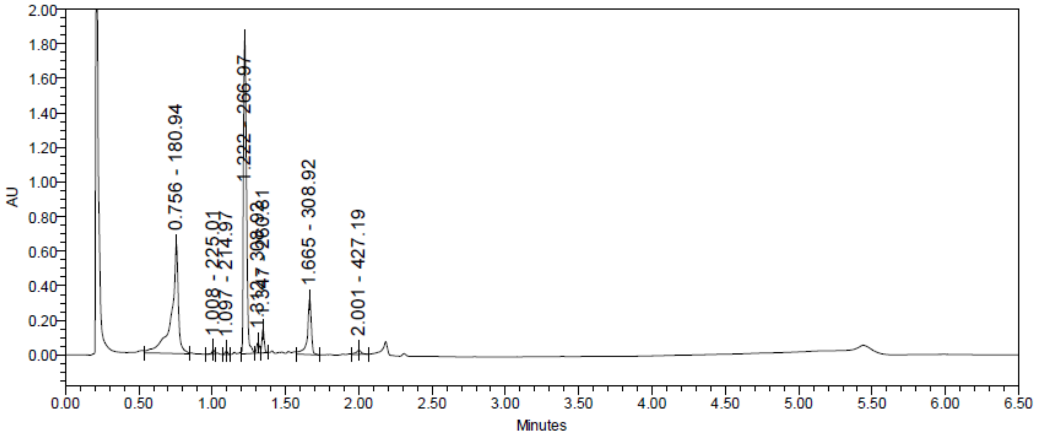


Injection Id: 8358; Result Id: 8651; Date Processed: Friday, December 6, 2024 3:50:45 PM GMT;
Processed Channel Descr. PDA Spectrum PDA 220.0 nm (PDA Spectrum (210-400)nm)

Peak Results

| | RT | % Area | Area (µV*sec) | Height (µV) | Channel |
|---|---|---|---|---|---|
| 1 | 0.756 | 37.138 | 2096303 | 631971 | PDA Spectrum |
| 2 | 1.008 | 0.592 | 33421 | 26815 | PDA Spectrum |
| 3 | 1.097 | 0.438 | 24748 | 19677 | PDA Spectrum |
| 4 | 1.222 | 45.847 | 2587866 | 1813834 | PDA Spectrum |
| 5 | 1.312 | 1.531 | 86406 | 59364 | PDA Spectrum |
| 6 | 1.347 | 2.594 | 146424 | 135193 | PDA Spectrum |
| 7 | 1.665 | 10.740 | 606249 | 317310 | PDA Spectrum |
| 8 | 2.001 | 1.120 | 63207 | 23668 | PDA Spectrum |

## Reaction C1 (55)

No product could be detected

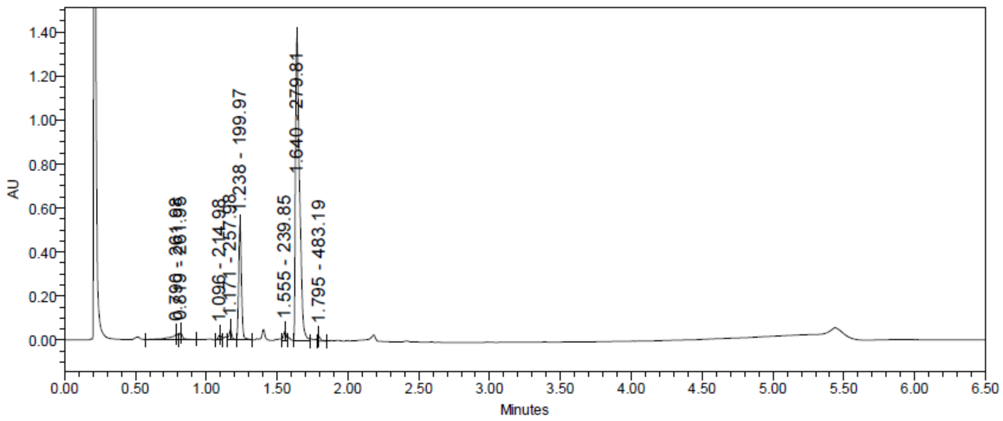


Injection Id: 8362; Result Id: 8701; Date Processed: Friday, December 6, 2024 3:51:47 PM GMT;
Processed Channel Descr. PDA Spectrum PDA 220.0 nm (PDA Spectrum (210-400)nm)

Peak Results

| | RT | % Area | Area (µV*sec) | Height (µV) | Channel |
|---|---|---|---|---|---|
| 1 | 0.790 | 2.517 | 95869 | 25346 | PDA Spectrum |
| 2 | 0.819 | 1.371 | 52206 | 29372 | PDA Spectrum |
| 3 | 1.096 | 0.834 | 31757 | 22212 | PDA Spectrum |
| 4 | 1.171 | 1.692 | 64449 | 46704 | PDA Spectrum |
| 5 | 1.238 | 18.917 | 720555 | 525068 | PDA Spectrum |
| 6 | 1.555 | 1.539 | 58607 | 42875 | PDA Spectrum |
| 7 | 1.640 | 72.486 | 2761051 | 1377087 | PDA Spectrum |
| 8 | 1.795 | 0.645 | 24584 | 19525 | PDA Spectrum |

**Reaction C2 (56)**

No product could be detected

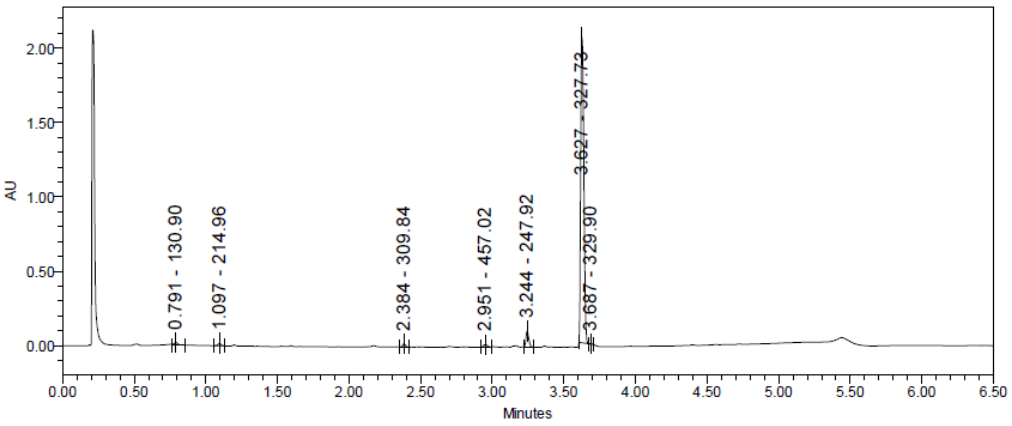


Injection Id: 8366; Result Id: 8894; Date Processed: Monday, December 9, 2024 11:49:33 AM GMT;
Processed Channel Descr. PDA Spectrum PDA 220.0 nm (PDA Spectrum (210-400)nm)

Peak Results

| | RT | % Area | Area (μV*sec) | Height (μV) | Channel |
|---|---|---|---|---|---|
| 1 | 0.791 | 0.991 | 34010 | 17725 | PDA Spectrum |
| 2 | 1.097 | 0.726 | 24910 | 16824 | PDA Spectrum |
| 3 | 2.384 | 0.958 | 32888 | 22200 | PDA Spectrum |
| 4 | 2.951 | 0.983 | 33725 | 16748 | PDA Spectrum |
| 5 | 3.244 | 4.312 | 148004 | 106183 | PDA Spectrum |
| 6 | 3.627 | 91.964 | 3156231 | 2051228 | PDA Spectrum |
| 7 | 3.687 | 0.065 | 2243 | 2153 | PDA Spectrum |

**Reaction C3 (57)**

Product observed at 3.22 and at 2.70 min as loss of acetyl group

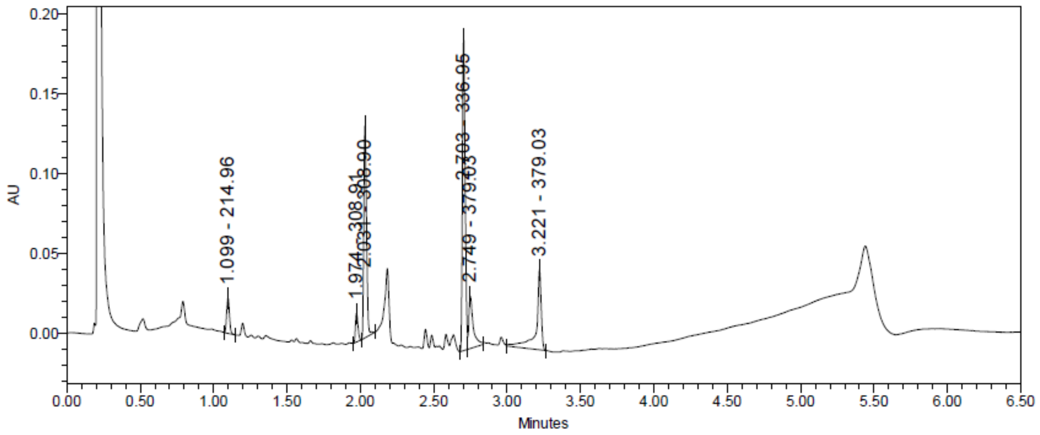


Injection Id: 8370; Result Id: 8705; Date Processed: Friday, December 6, 2024 3:51:51 PM GMT;
Processed Channel Descr. PDA Spectrum PDA 220.0 nm (PDA Spectrum (210-400)nm)

Peak Results

| | RT | % Area | Area (µV*sec) | Height (µV) | Channel |
|---|---|---|---|---|---|
| 1 | 1.099 | 4.373 | 29192 | 22055 | PDA Spectrum |
| 2 | 1.974 | 3.260 | 21764 | 17864 | PDA Spectrum |
| 3 | 2.031 | 25.639 | 171145 | 133085 | PDA Spectrum |
| 4 | 2.703 | 38.338 | 255912 | 195928 | PDA Spectrum |
| 5 | 2.749 | 10.181 | 67959 | 32779 | PDA Spectrum |
| 6 | 3.221 | 18.209 | 121552 | 50182 | PDA Spectrum |

**Reaction C4 (58)**

No product could be detected

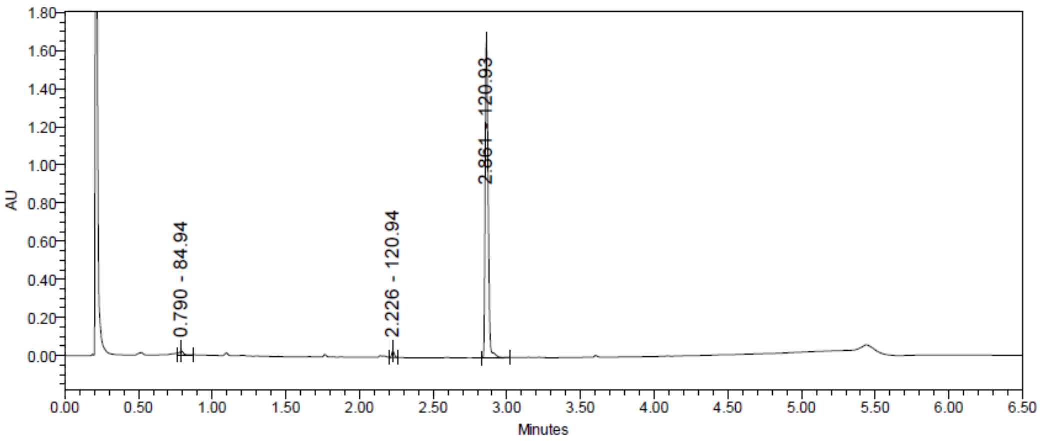


Injection Id: 8374; Result Id: 8659; Date Processed: Friday, December 6, 2024 3:50:53 PM GMT; Processed Channel Descr. PDA Spectrum PDA 220.0 nm (PDA Spectrum (210-400)nm)

Peak Results

| | RT | % Area | Area (µV*sec) | Height (µV) | Channel |
|---|---|---|---|---|---|
| 1 | 0.790 | 1.645 | 39781 | 20230 | PDA Spectrum |
| 2 | 2.226 | 1.614 | 39023 | 32560 | PDA Spectrum |
| 3 | 2.861 | 96.741 | 2339272 | 1652577 | PDA Spectrum |

**Reaction C5 (59)**

Product observed at 1.21 min as loss of acetyl group

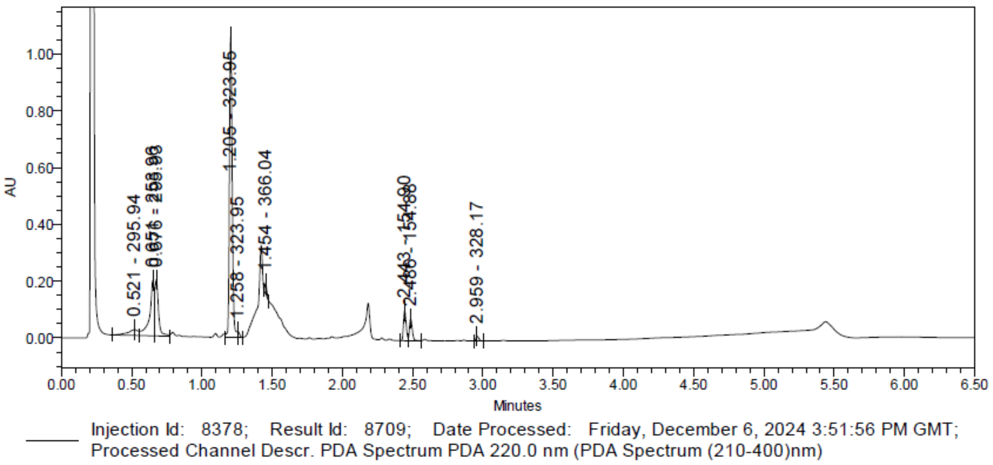

Peak Results

| | RT | % Area | Area (µV*sec) | Height (µV) | Channel |
|---|---|---|---|---|---|
| 1 | 0.521 | 3.923 | 105551 | 20717 | PDA Spectrum |
| 2 | 0.651 | 16.275 | 437891 | 195245 | PDA Spectrum |
| 3 | 0.676 | 13.099 | 352441 | 197885 | PDA Spectrum |
| 4 | 1.205 | 54.589 | 1468793 | 1058466 | PDA Spectrum |
| 5 | 1.258 | 0.744 | 20029 | 19733 | PDA Spectrum |
| 6 | 1.454 | 1.316 | 35408 | 36673 | PDA Spectrum |
| 7 | 2.443 | 5.171 | 139143 | 104851 | PDA Spectrum |
| 8 | 2.486 | 4.031 | 108453 | 77398 | PDA Spectrum |
| 9 | 2.959 | 0.852 | 22935 | 17003 | PDA Spectrum |

## Reaction C6 (60)

Product observed at 1.71 min as loss of acetyl group

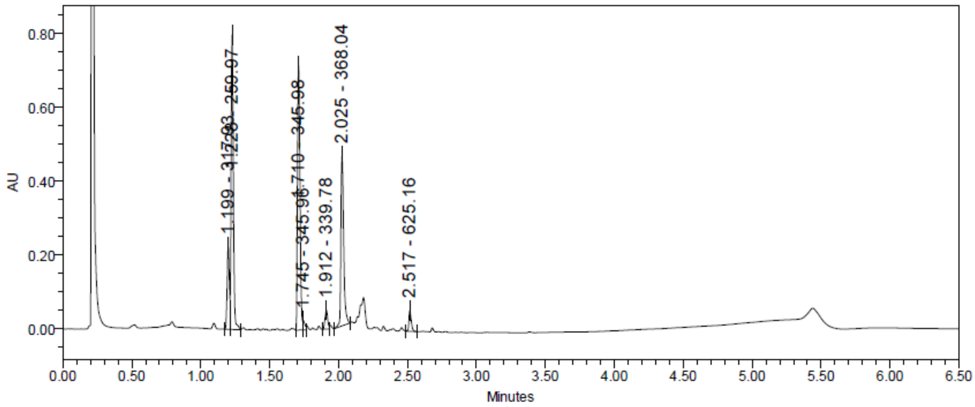


Injection Id: 8382; Result Id: 8663; Date Processed: Friday, December 6, 2024 3:50:58 PM GMT;
Processed Channel Descr. PDA Spectrum PDA 220.0 nm (PDA Spectrum (210-400)nm)

Peak Results

| | RT | % Area | Area (µV*sec) | Height (µV) | Channel |
|---|---|---|---|---|---|
| 1 | 1.199 | 8.485 | 243237 | 222438 | PDA Spectrum |
| 2 | 1.228 | 31.370 | 899215 | 797804 | PDA Spectrum |
| 3 | 1.710 | 31.809 | 911807 | 718604 | PDA Spectrum |
| 4 | 1.745 | 0.906 | 25963 | 24895 | PDA Spectrum |
| 5 | 1.912 | 2.385 | 68377 | 51157 | PDA Spectrum |
| 6 | 2.025 | 22.698 | 650643 | 459516 | PDA Spectrum |
| 7 | 2.517 | 2.347 | 67281 | 56554 | PDA Spectrum |

## Reaction D1 (61)

Product observed at 1.66 min as loss of acetyl group

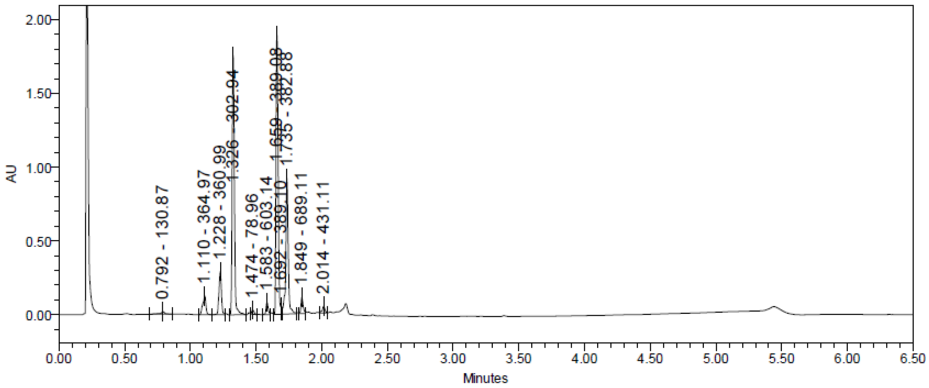


Injection Id: 8386; Result Id: 8713; Date Processed: Friday, December 6, 2024 3:52:01 PM GMT;
Processed Channel Descr. PDA Spectrum PDA 220.0 nm (PDA Spectrum (210-400)nm)

Peak Results

| | RT | % Area | Area (µV*sec) | Height (µV) | Channel |
|---|---|---|---|---|---|
| 1 | 0.792 | 0.618 | 39433 | 16149 | PDA Spectrum |
| 2 | 1.110 | 3.320 | 211675 | 126171 | PDA Spectrum |
| 3 | 1.228 | 6.004 | 382819 | 292693 | PDA Spectrum |
| 4 | 1.326 | 32.160 | 2050546 | 1757198 | PDA Spectrum |
| 5 | 1.474 | 0.545 | 34731 | 27846 | PDA Spectrum |
| 6 | 1.583 | 1.508 | 96147 | 84150 | PDA Spectrum |
| 7 | 1.659 | 33.585 | 2141440 | 1906058 | PDA Spectrum |
| 8 | 1.692 | 0.682 | 43492 | 52788 | PDA Spectrum |
| 9 | 1.735 | 18.638 | 1188363 | 920735 | PDA Spectrum |
| 10 | 1.849 | 1.959 | 124938 | 109349 | PDA Spectrum |
| 11 | 2.014 | 0.981 | 62547 | 44344 | PDA Spectrum |

**Reaction D2 (62)**

No product could be detected

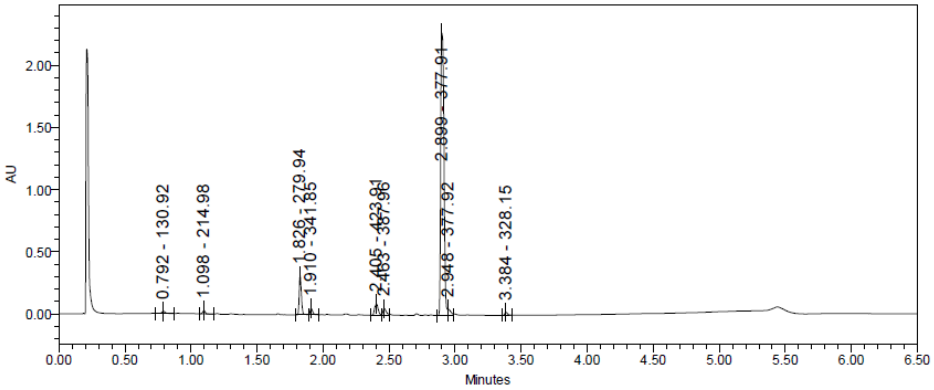


Injection Id: 8390; Result Id: 8667; Date Processed: Friday, December 6, 2024 3:51:03 PM GMT;
Processed Channel Descr. PDA Spectrum PDA 220.0 nm (PDA Spectrum (210-400)nm)

Peak Results

| | RT | % Area | Area (µV*sec) | Height (µV) | Channel |
|---|---|---|---|---|---|
| 1 | 0.792 | 0.946 | 45491 | 18609 | PDA Spectrum |
| 2 | 1.098 | 0.741 | 35629 | 25871 | PDA Spectrum |
| 3 | 1.826 | 7.584 | 364857 | 314571 | PDA Spectrum |
| 4 | 1.910 | 1.479 | 71156 | 52008 | PDA Spectrum |
| 5 | 2.405 | 3.582 | 172315 | 91032 | PDA Spectrum |
| 6 | 2.463 | 2.061 | 99135 | 55013 | PDA Spectrum |
| 7 | 2.899 | 81.245 | 3908872 | 2270790 | PDA Spectrum |
| 8 | 2.948 | 1.639 | 78872 | 48998 | PDA Spectrum |
| 9 | 3.384 | 0.725 | 34868 | 24737 | PDA Spectrum |

**Reaction D3 (63)**

Product observed at 3.42 min as loss of acetyl group

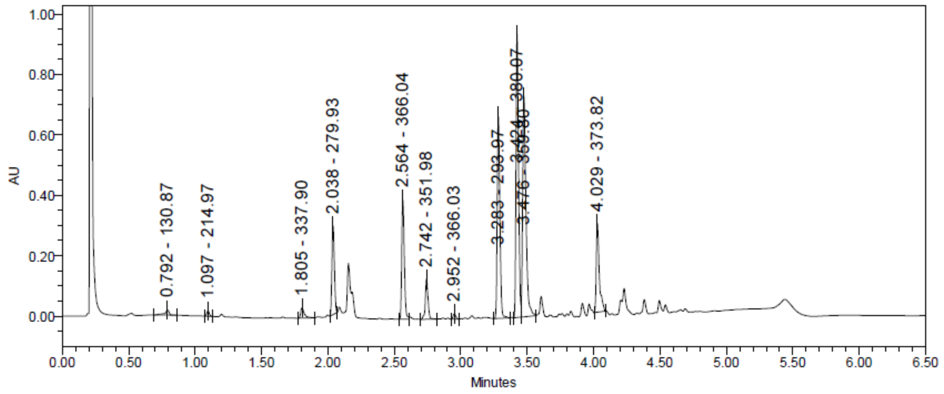


Injection Id: 8394; Result Id: 8900; Date Processed: Monday, December 9, 2024 12:52:17 PM GMT;
Processed Channel Descr. PDA Spectrum PDA 220.0 nm (PDA Spectrum (210-400)nm)

Peak Results

| | RT | % Area | Area (μV*sec) | Height (μV) | Channel |
|---|---|---|---|---|---|
| 1 | 0.792 | 0.829 | 42628 | 17039 | PDA Spectrum |
| 2 | 1.097 | 0.456 | 23466 | 17748 | PDA Spectrum |
| 3 | 1.805 | 1.254 | 64469 | 33958 | PDA Spectrum |
| 4 | 2.038 | 7.307 | 375803 | 293404 | PDA Spectrum |
| 5 | 2.564 | 10.345 | 532015 | 396186 | PDA Spectrum |
| 6 | 2.742 | 3.725 | 191562 | 133149 | PDA Spectrum |
| 7 | 2.952 | 0.499 | 25665 | 17858 | PDA Spectrum |
| 8 | 3.283 | 18.162 | 934023 | 669721 | PDA Spectrum |
| 9 | 3.424 | 24.022 | 1235401 | 939748 | PDA Spectrum |
| 10 | 3.476 | 24.440 | 1256904 | 730224 | PDA Spectrum |
| 11 | 4.029 | 8.962 | 460929 | 293860 | PDA Spectrum |

**Reaction D4 (64)**

Product observed at 2.00 min as loss of acetyl group

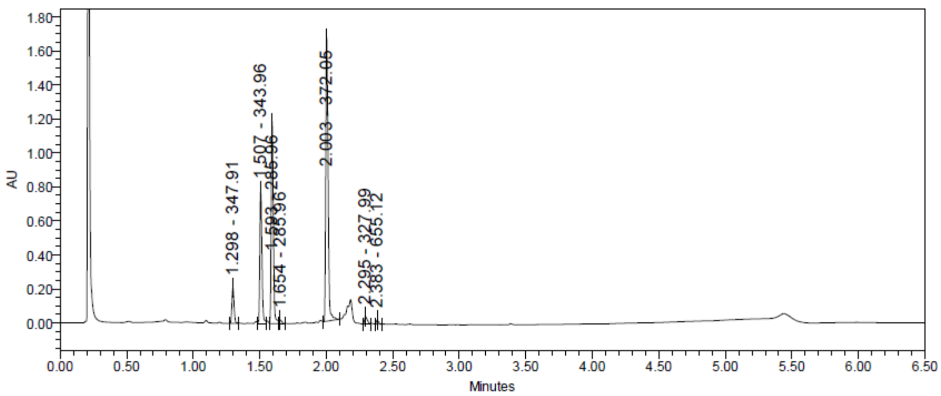

Injection Id: 8398; Result Id: 8671; Date Processed: Friday, December 6, 2024 3:51:08 PM GMT;
Processed Channel Descr. PDA Spectrum PDA 220.0 nm (PDA Spectrum (210-400)nm)

Peak Results

| | RT | % Area | Area (µV*sec) | Height (µV) | Channel |
|---|---|---|---|---|---|
| 1 | 1.298 | 5.169 | 237968 | 208785 | PDA Spectrum |
| 2 | 1.507 | 19.030 | 876144 | 779773 | PDA Spectrum |
| 3 | 1.593 | 28.707 | 1321666 | 1189029 | PDA Spectrum |
| 4 | 1.654 | 0.819 | 37730 | 21266 | PDA Spectrum |
| 5 | 2.003 | 44.266 | 2038022 | 1670493 | PDA Spectrum |
| 6 | 2.295 | 1.406 | 64742 | 44164 | PDA Spectrum |
| 7 | 2.383 | 0.603 | 27761 | 23703 | PDA Spectrum |

## Reaction D5 (65)

Product observed at 2.10 min as loss of acetyl group

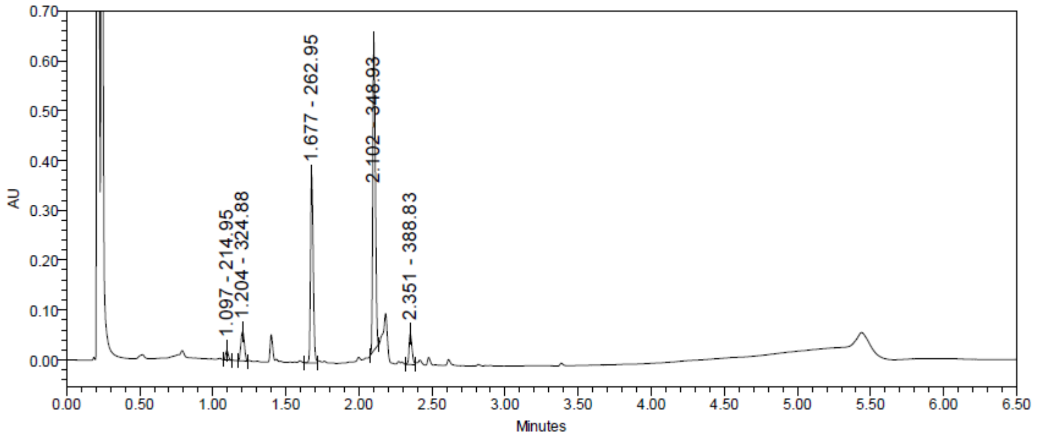


Injection Id: 8402; Result Id: 8994; Date Processed: Monday, December 9, 2024 6:53:49 PM GMT; Processed Channel Descr. PDA Spectrum PDA 220.0 nm (PDA Spectrum (210-400)nm)

Peak Results

| | RT | % Area | Area (µV*sec) | Height (µV) | Channel |
|---|---|---|---|---|---|
| 1 | 1.097 | 1.658 | 24245 | 18887 | PDA Spectrum |
| 2 | 1.204 | 6.827 | 99810 | 58200 | PDA Spectrum |
| 3 | 1.677 | 33.307 | 486910 | 377084 | PDA Spectrum |
| 4 | 2.102 | 52.256 | 763920 | 618999 | PDA Spectrum |
| 5 | 2.351 | 5.952 | 87006 | 62268 | PDA Spectrum |

## Reaction D6 (66)

No product could be detected

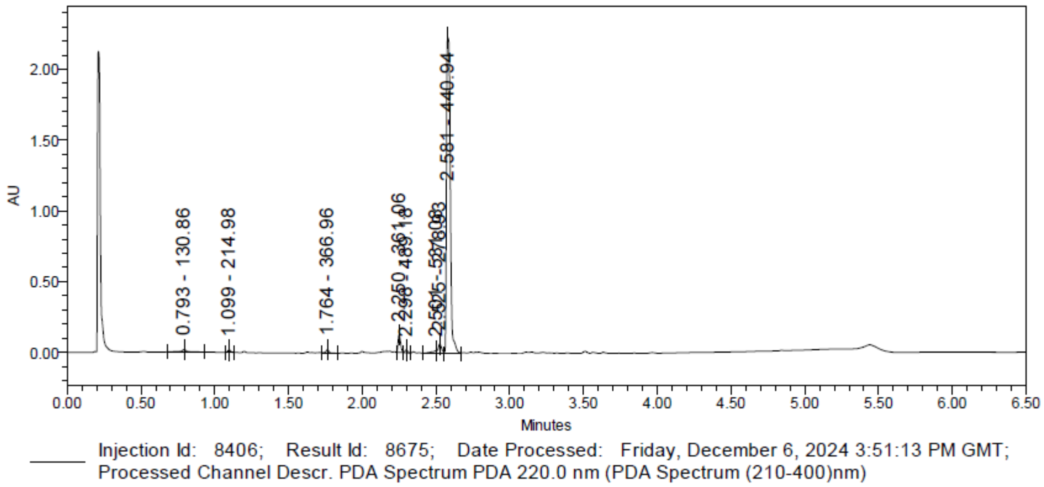


Peak Results

| | RT | % Area | Area (µV*sec) | Height (µV) | Channel |
|---|---|---|---|---|---|
| 1 | 0.793 | 1.106 | 49564 | 17270 | PDA Spectrum |
| 2 | 1.099 | 0.520 | 23280 | 17426 | PDA Spectrum |
| 3 | 1.764 | 0.953 | 42684 | 25403 | PDA Spectrum |
| 4 | 2.250 | 3.368 | 150903 | 124185 | PDA Spectrum |
| 5 | 2.298 | 0.629 | 28174 | 20639 | PDA Spectrum |
| 6 | 2.501 | 0.929 | 41627 | 18153 | PDA Spectrum |
| 7 | 2.525 | 2.336 | 104671 | 66207 | PDA Spectrum |
| 8 | 2.581 | 90.159 | 4039235 | 2228537 | PDA Spectrum |

**Ethyl 2-(2-(2,2,2-trifluoroethoxy)pyrimidin-5-yl)acetate (S3)**

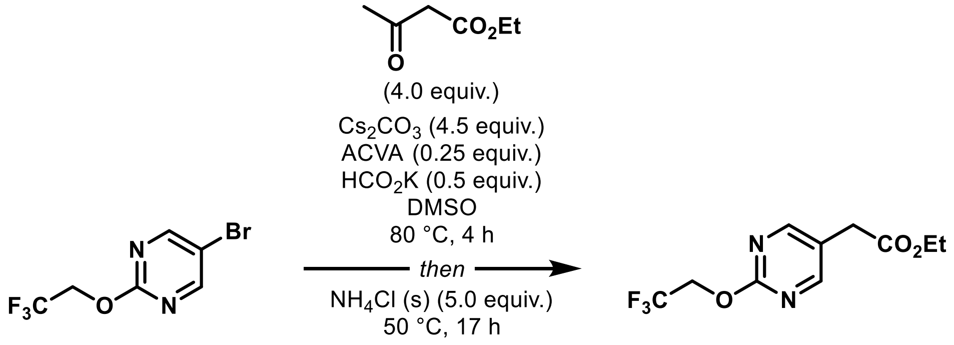


**49** (0.30 mmol)

Synthesised according to **General Procedure A** with **49** (77.1 mg, 0.300 mmol, 1.0 equiv.), ethyl acetoacetate (156 mg, 1.20 mmol, 4.0 equiv.), $Cs_2CO_3$ (440 mg, 1.35 mmol, 4.5 equiv.), ACVA (25.6 mg, 75 μmol, 0.25 equiv.) and $HCO_2K$ (12.6 mg, 150 μmol, 0.5 equiv.) in anhydrous DMSO (1.5 mL). The reaction mixture was heated at 80 °C for 4 hours. $NH_4Cl$ (80.3 mg, 1.50 mmol, 5.0 equiv.) was then added and the reaction mixture was stirred at 50 °C for 17 hours. The crude product was purified by column chromatography (5 → 15% EtOAc in pentane) to afford the *title compound* **S3** as a white solid (38.7 mg, 0.146 mmol, 49%).

***$R_f$*** 0.37 (20% EtOAc in pentane).

**$^1$H NMR** (500 MHz, $CDCl_3$) $\delta_H$ 8.48 (s, 2H), 4.80 (q, *J* = 8.3 Hz, 2H), 4.19 (q, *J* = 7.2 Hz, 2H), 3.58 (s, 2H), 1.28 (t, *J* = 7.2 Hz, 3H).

**$^{13}$C NMR** (126 MHz, $CDCl_3$) $\delta_C$ 170.1 (C), 163.1 (C), 160.0 (2CH), 123.3(q, *J* = 278.1 Hz, C), 122.7 (C), 63.7 (q, *J* = 36.4 Hz, $CH_2$), 61.7 ($CH_2$), 35.1 ($CH_2$), 14.3 ($CH_3$).

**$^{19}$F NMR** (376 MHz, $CDCl_3$) $\delta_F$ −73.8 (t, *J* = 8.3 Hz, 3F).

**HRMS** ($ESI^+$) m/z calcd. for $C_{10}H_{11}O_3N_2F_3Na$ $(M + Na)^+$ 287.0614, found 287.0612.

**Preparative Scale Synthesis of ethyl 2-[3-[(1S)-1-imidazo[1,2-a]pyridin-6-ylethyl]triazolo[4,5-b]pyrazin-5-yl]acetate (S4)**

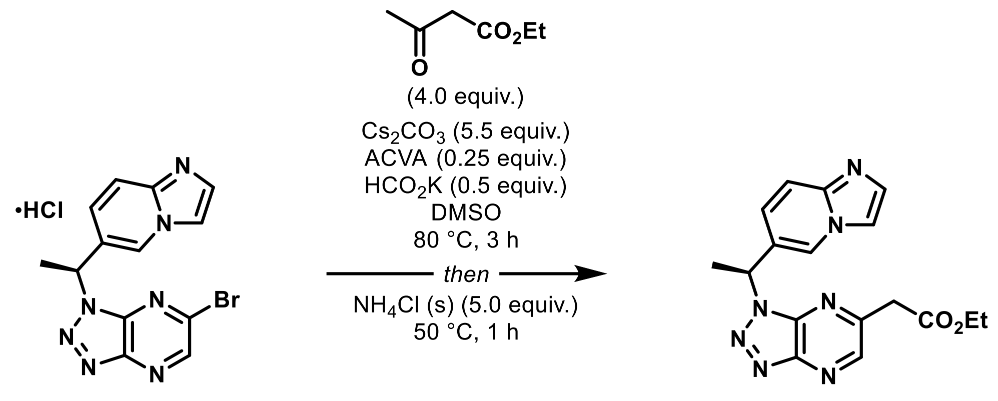


**59** (4.0 mmol)

To a 100 mL 2-piece easymax reactor vessel fitted with an overhead stirrer, $N_2$ inlet, solid addition port and a condenser was added **59**.HCl (1.52 g, 4.0 mmol, 1.0 equiv.), 4,4'-azobis(4-cyanovaleric acid) (0.280 g, 1.0 mmol, 0.25 equiv.), potassium formate (0.168 g, 2.0 mmol, 0.50 equiv) and cesium carbonate (7.17 g, 22 mmol, 5.5 equiv.). DMSO (450 mL, 10 L/mol wrt **59**) that had been degassed was added, giving a dark orange solution with well dispersed white solid. Ethyl acetoacetate (2.04 mL, 16 mmol, 4.0 equiv.) was added as a liquid via syringe via a septum over the solid addition port. The reaction was sampled for T=0 via uHPLC/MS then heated to an internal reaction temperature of 80 °C. A reaction sample at T=3 h indicated the reaction was complete (starting material consumed) by uHPLC/MS analysis and was cooled to 50 °C. Solid $NH_4Cl$ (1.07 g, 20 mmol, 5.0 equiv.) was added and the reaction was stirred for 1 hour at 50 °C then cooled to room temperature. The reaction mixture was transferred to a separating funnel and diluted with 120 mL of water, then extracted with 3 x 100 mL of 1:1 MTBE/EtAOc, the combined organics dried with $Na_2SO_4$, filtered and concentrated to give the crude product (12.2 g, 38% NMR assay purity, 41% yield). The crude product was purified via silica chromatography on a Teledyne CombiFlash system (80 g column) eluting with 20% EtOAc iso-hexane to elute impurities and side products then 10% MeOH/$CH_2Cl_2$ to collect the *titled compound* **S4** (450 mg, 1.28 mmol, 32% yield) as a dark orange oil.

**$^1$H NMR** (500 MHz, $CDCl_3$) $\delta_H$ 8.72 (s, 1H), 8.36 (dd, *J* = 1.8, 0.9 Hz, 1H), 7.62 (d, *J* = 1.2 Hz, 1H), 7.59 (br s, 1H), 7.58 (br d, *J* = 9.5 Hz, 1H), 7.35 (dd, *J* = 9.5, 1.8 Hz, 1H), 6.30 (q, *J* = 7.2 Hz, 1H), 4.19 (q, *J* = 7.2 Hz, 1H), 4.06 (s, 2H), 2.25 (d, *J* = 7.2 Hz, 3H), 1.24 (t, *J* = 7.2 Hz, 3H).

$^{13}C$ **NMR** (126 MHz, $CDCl_3$) $\delta_C$ 168.9 (C), 150.9 (C), 148.6 (C), 144.9 (C), 144.6 (C), 137.3 (C), 134.5 (CH), 124.8 (C), 124.5 (CH), 123.8 ($CH_3$), 118.4 (CH), 113.2 (CH), 61.9 ($CH_2$), 56.3(CH), 41.9 ($CH_2$), 20.4 ($CH_3$), 14.2 ($CH_3$)

**HRMS** ($ESI^+$) m/z calcd. for $C_{17}H_{18}N_7O_2$ $(M + H)^+$ 352.1517, found 352.1515.

## 8. References


53. G. R. Buettner, Spin trapping: ESR parameters of spin adducts. *Free Radic. Biol. Med.* **3**, 259-303 (1987).

54. S. Stoll and A. Schweiger, EasySpin, a comprehensive software package for spectral simulation and analysis in EPR. *J. Man. Reson.* **178**, 42-55 (2006).

55. F. Neese, Software update: The ORCA program system—Version 5.0. *Wiley Interdisciplinary Reviews: Computational Molecular Science* **12**, e1606 (2022).

56. Y. Zhao, D. G. Truhlar, The M06 suite of density functionals for main group thermochemistry, thermochemical kinetics, noncovalent interactions, excited states, and transition elements: two new functionals and systematic testing of four M06-class functionals and 12 other functionals. *Theor Chem Account* **120**, 215–241 (2008).

57. F. Weigend, Accurate Coulomb-fitting basis sets for H to Rn. *Phys. Chem. Chem. Phys.* **8**, 1057–1065 (2006).

58. A. V. Marenich, C. J. Cramer, D. G. Truhlar, Universal Solvation Model Based on Solute Electron Density and on a Continuum Model of the Solvent Defined by the Bulk Dielectric Constant and Atomic Surface Tensions. *J. Phys. Chem. B* **113**, 6378–6396 (2009).

59. ChemCraft 1.8, https://www.chemcraftprog.com

60. C. J. Cramer, *Essentials of Computational Chemistry: Theories and Models*. (Wiley, ed. 2, 2013).

61. Roth, H. G., Romero, N. A. & Nicewicz, D. A. Experimental and Calculated Electrochemical Potentials of Common Organic Molecules for Applications to Single-Electron Redox Chemistry. *Synlett* **27**, 714–723 (2016).

62. Neumeier, M. *et al.* Dichromatic Photocatalytic Substitutions of Aryl Halides with a Small Organic Dye. *Chem. Eur. J.* **24**, 105–108 (2018).

63. Isse, A. A. & Gennaro, A. Absolute Potential of the Standard Hydrogen Electrode and the Problem of Interconversion of Potentials in Different Solvents. *J. Phys. Chem. B* **114**, 7894–7899 (2010).

64. Y. Li, S. Chen, M. Wang, X. Jiang, Sodium Dithionite-Mediated Decarboxylative Sulfonylation: Facile Access to Tertiary Sulfones. *Angew. Chem. Int. Ed.* **59**, 8907–8911 (2020).

65. *Recommendations on the Transport of Dangerous Goods, Manual of Tests and Criteria*. (United Nations, ed. 7, 2019)

66. Q. Yang, M. Sheng, X. Li, C. Tucker, S. Vásquez Céspedes, N. J. Webb, G. T. Whiteker, J. Yu, Potential Explosion Hazards Associated with the Autocatalytic Thermal Decomposition of Dimethyl Sulfoxide and Its Mixtures. *Org. Process Res. Dev.* **24**, 916–939 (2020).

67. C. W. Coley, L. Rogers, W. H. Green, K. F. Jensen, SCScore: Synthetic Complexity Learned from a Reaction Corpus. *J. Chem. Inf. Model.* **58**, 252–261 (2018).

68. J. R. Proudfoot, A path based approach to assessing molecular complexity. *Bioorg. Med. Chem. Lett.* **27**, 2014–2017 (2017).

# 9. NMR Spectral Data

## $^{1}$H NMR of ethyl 2-(4-cyanophenyl)acetate (4)

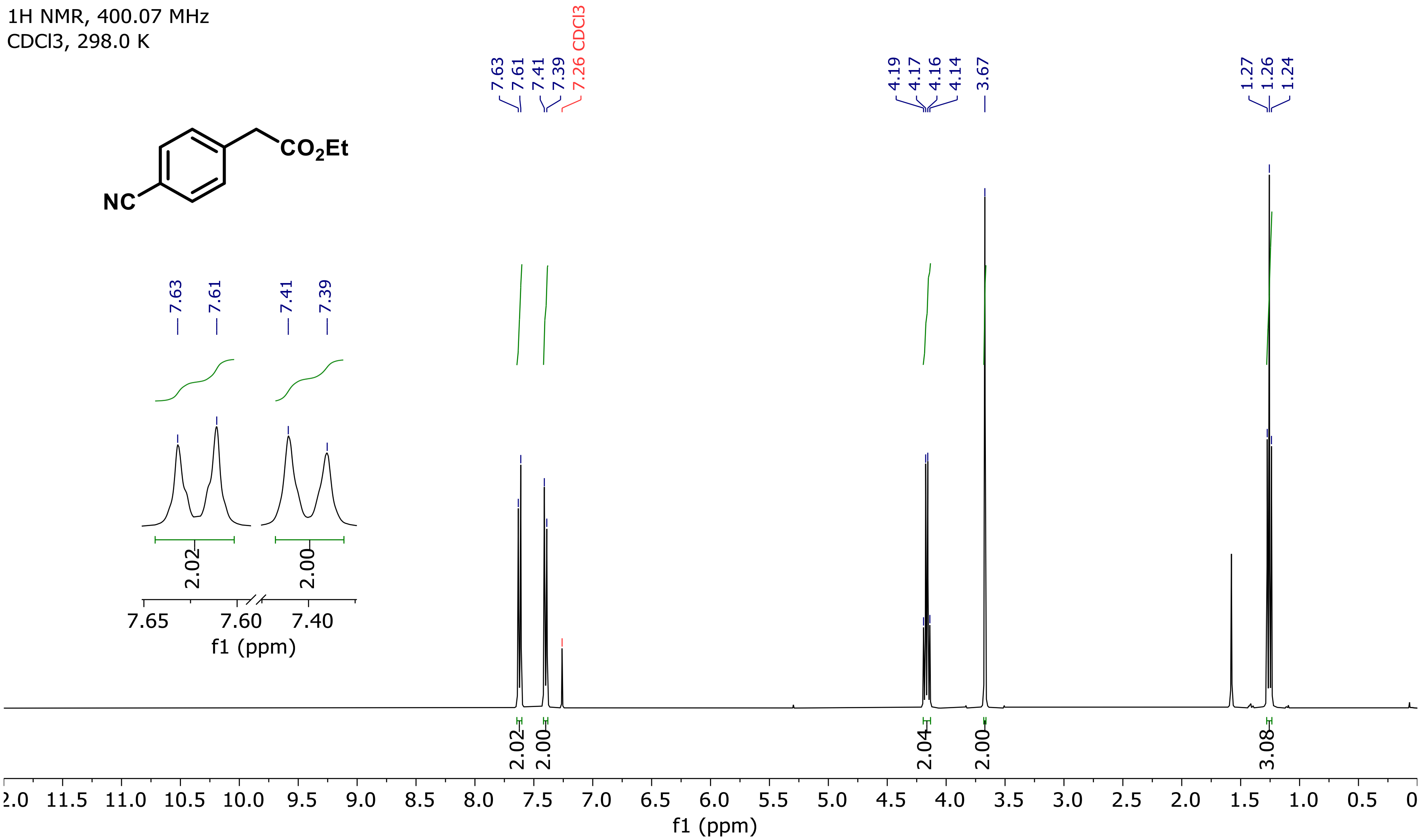

**$^{13}$C NMR of ethyl 2-(4-cyanophenyl)acetate (4)**

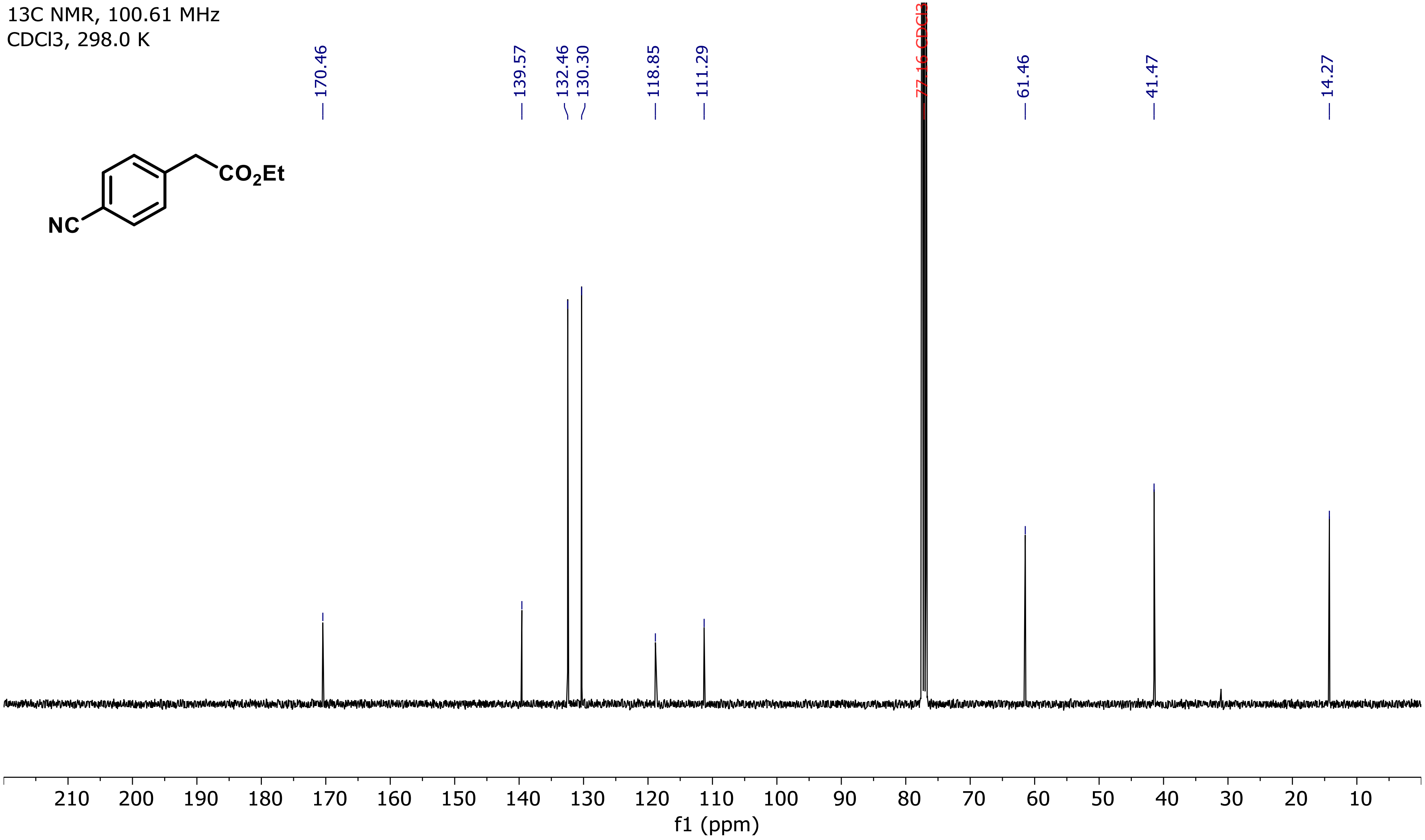

**$^{1}$H NMR of ethyl 2-(3-cyanophenyl)acetate (6)**

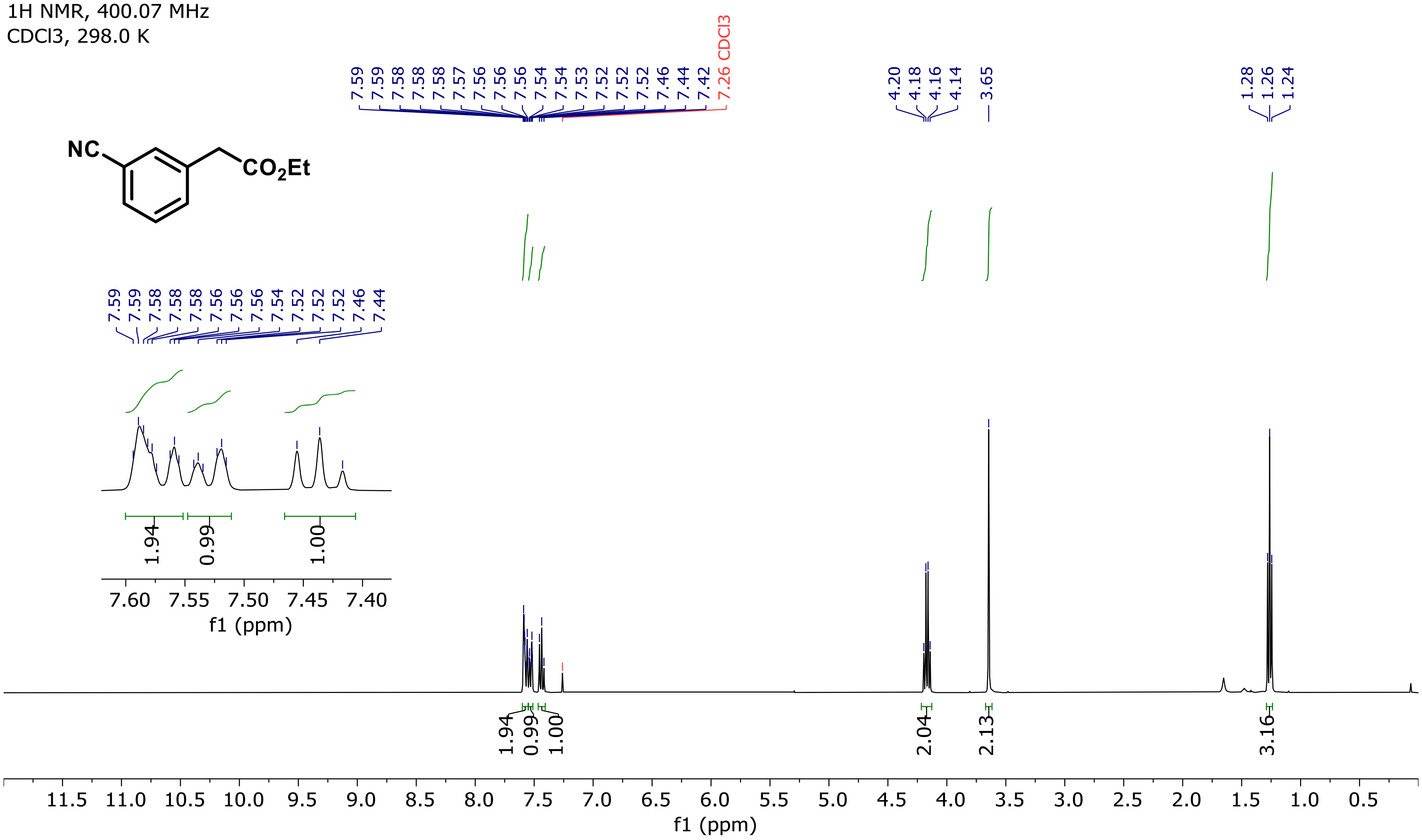

## $^{13}$C NMR of ethyl 2-(3-cyanophenyl)acetate (6)

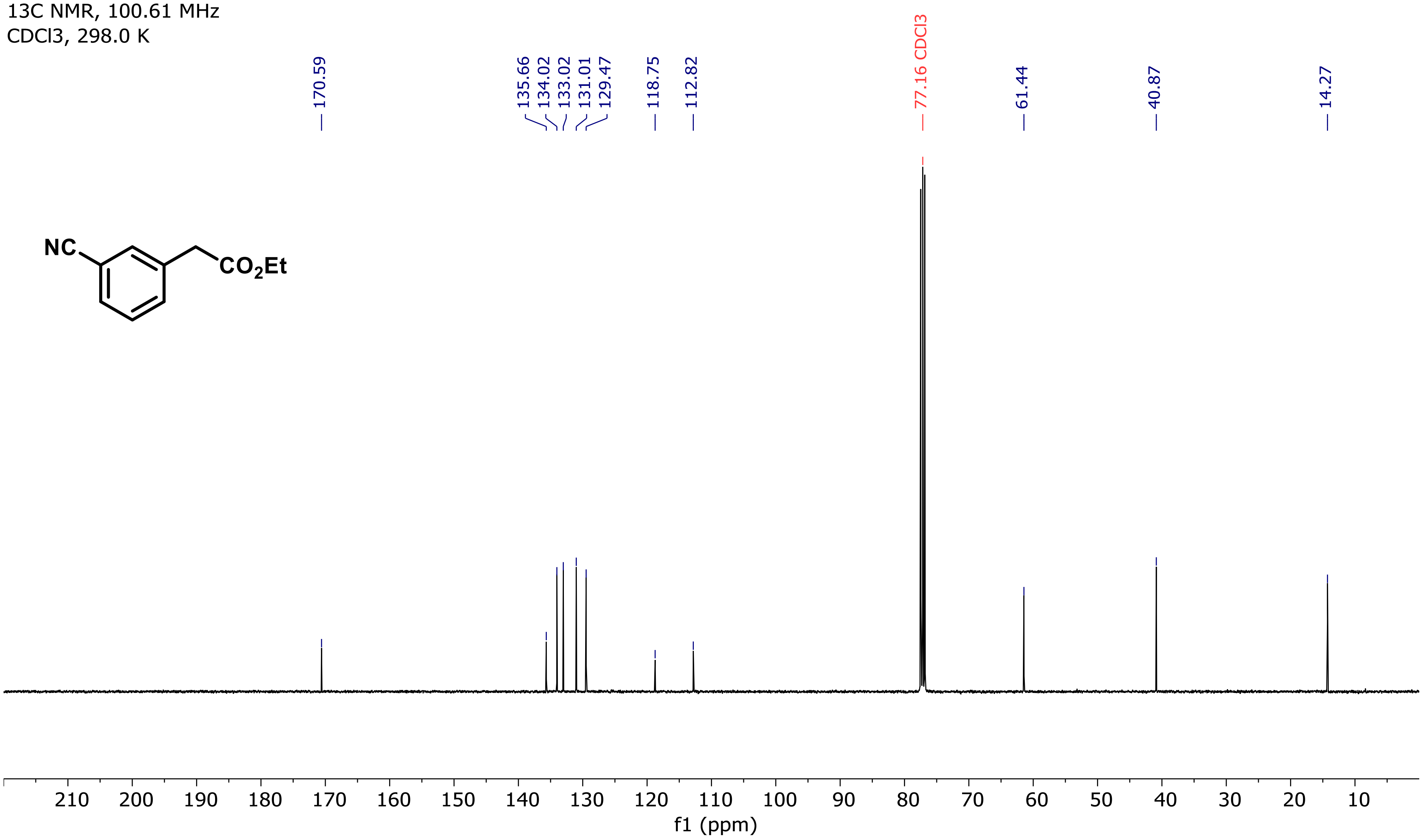

**$^{1}$H NMR of ethyl 2-(2-cyanophenyl)acetate (7)**

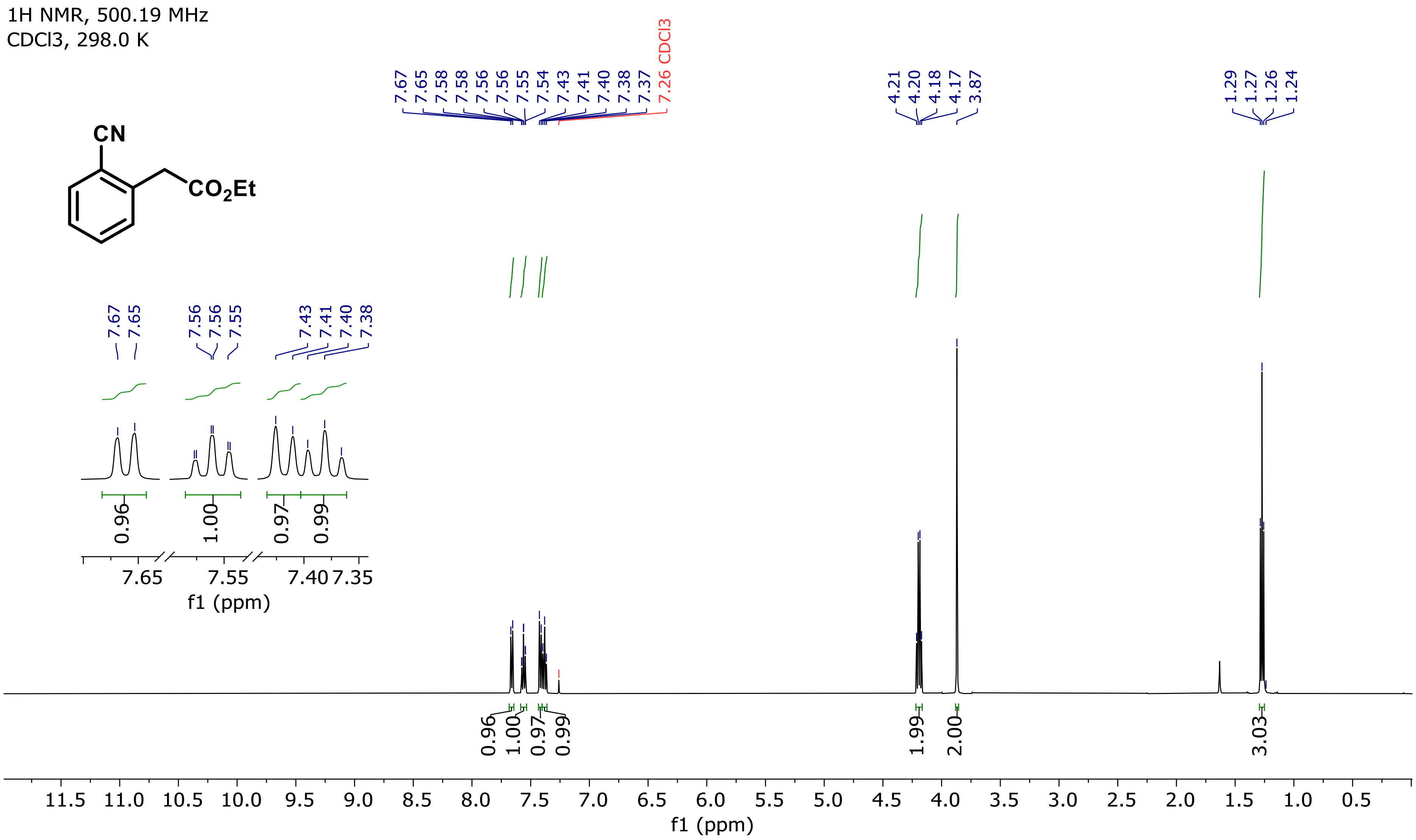

**$^{13}$C NMR of ethyl 2-(2-cyanophenyl)acetate (7)**

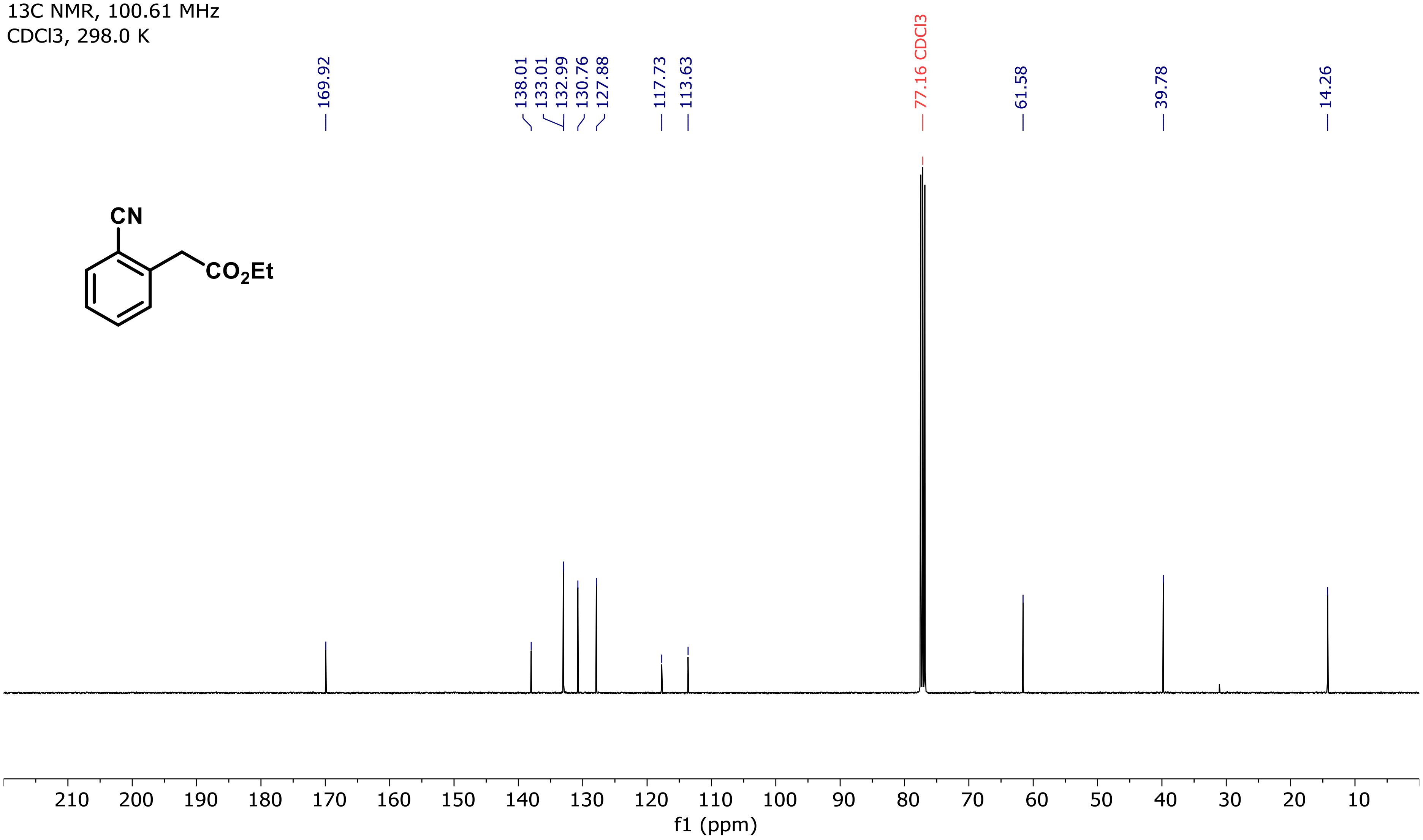

**$^{1}$H NMR of ethyl 2-(4-(methylsulfonyl)phenyl)acetate (8)**

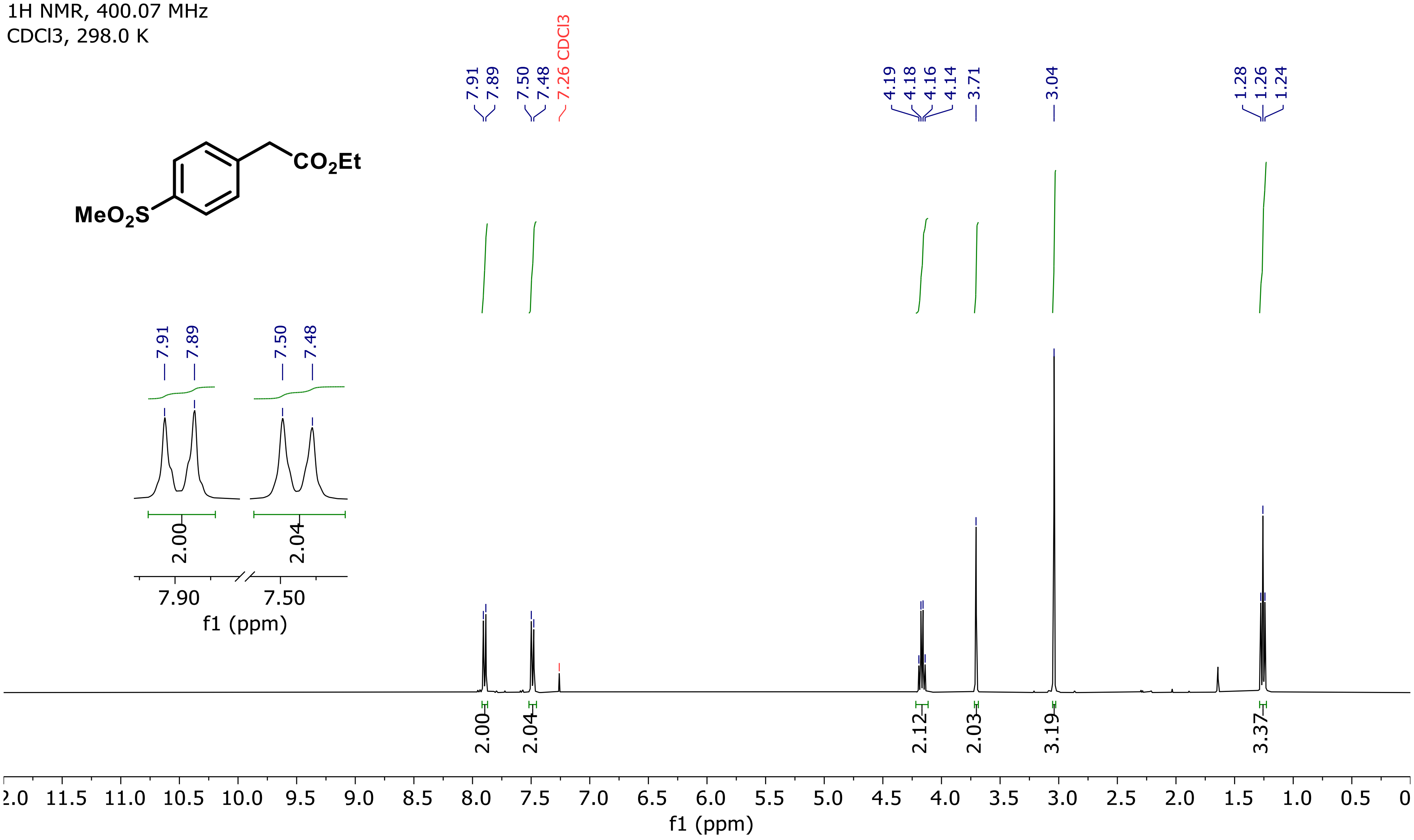

**$^{13}$C NMR of ethyl 2-(4-(methylsulfonyl)phenyl)acetate (8)**

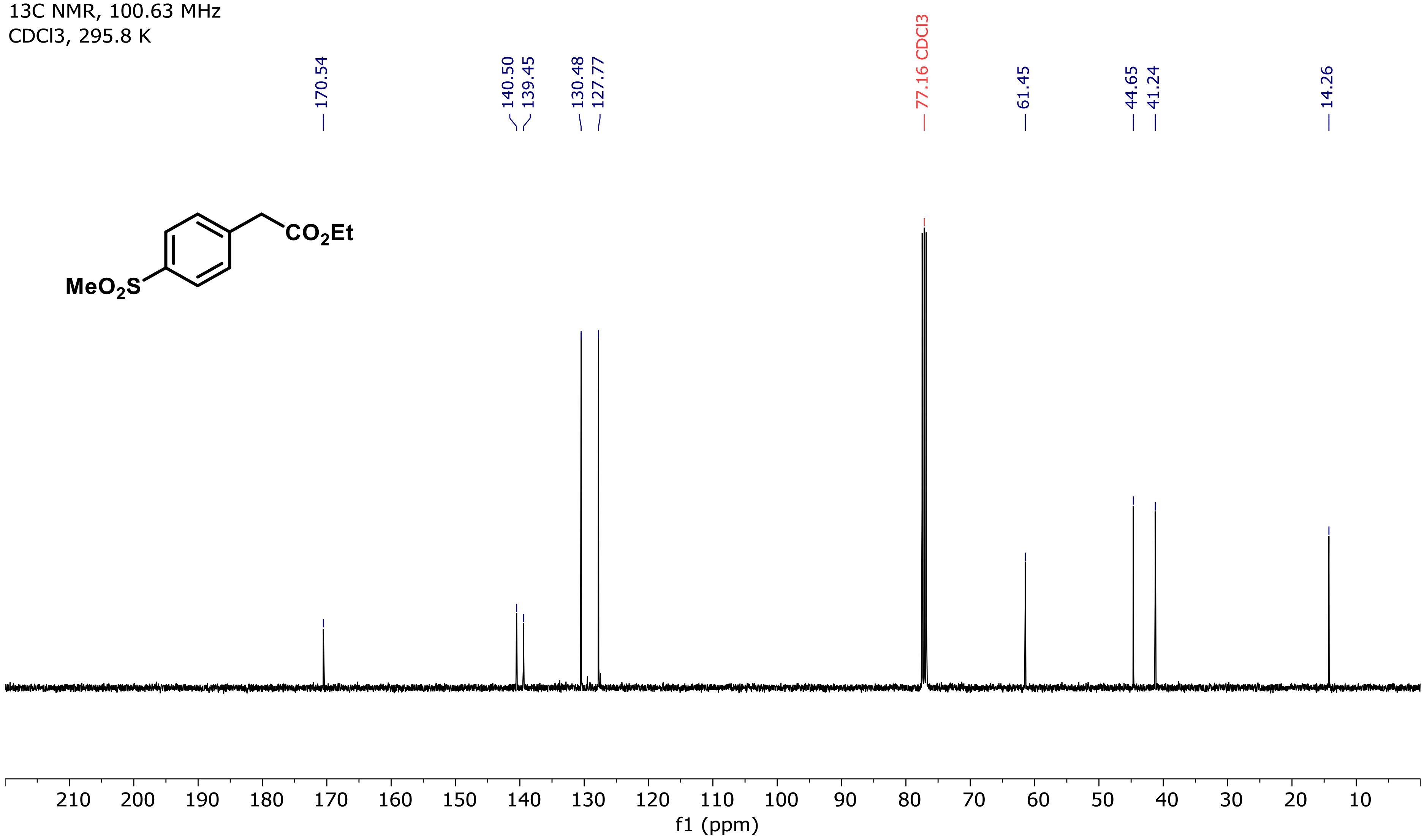

**$^{1}$H NMR of ethyl 2-(3-(methylsulfonyl)phenyl)acetate (9)**

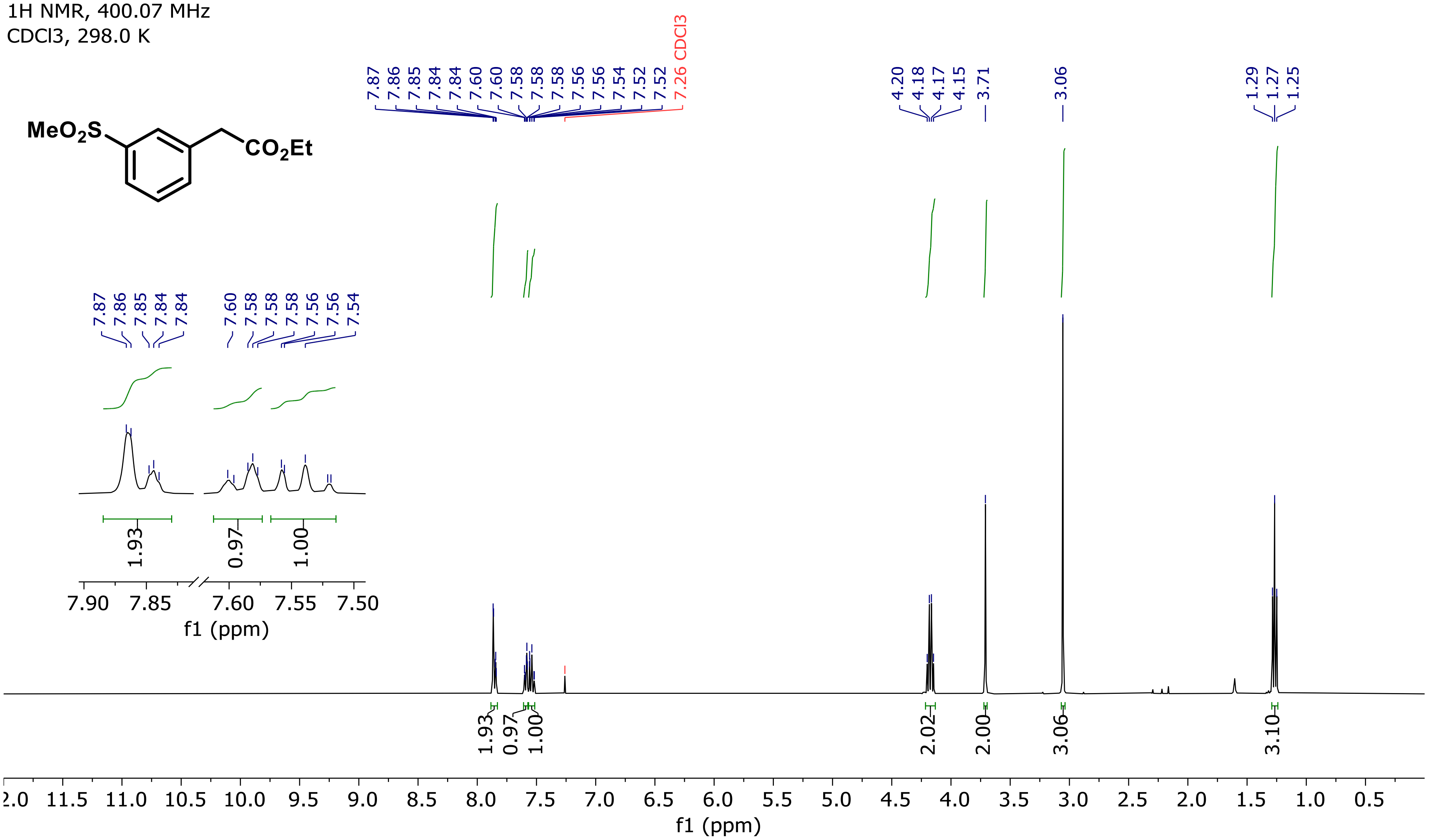

**$^{13}$C NMR of ethyl 2-(3-(methylsulfonyl)phenyl)acetate (9)**

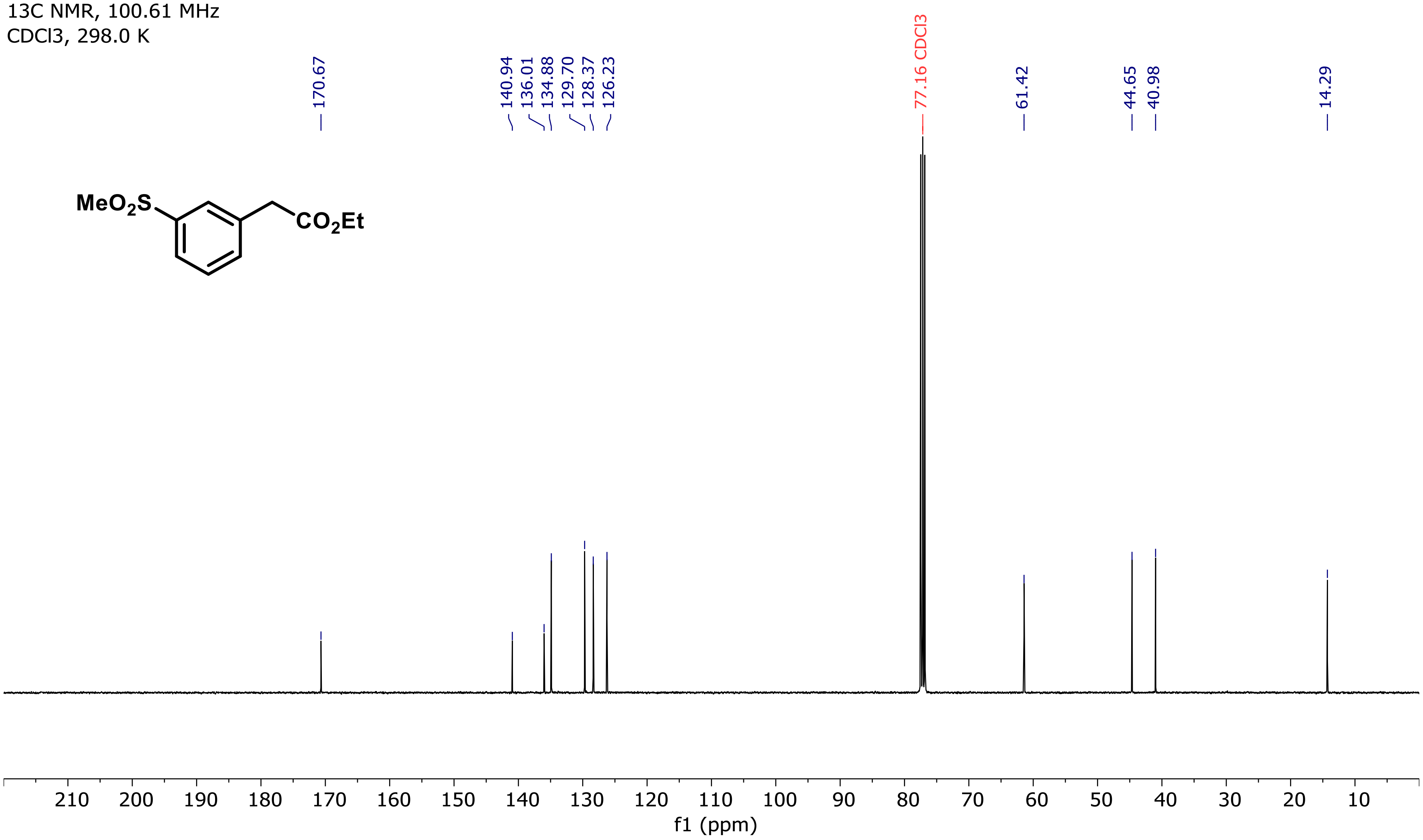

**$^{1}$H NMR used to determine yield of ethyl 2-(2-(methylsulfonyl)phenyl)acetate (10)**

1H NMR, 400.17 MHz
DMSO, 294.9 K

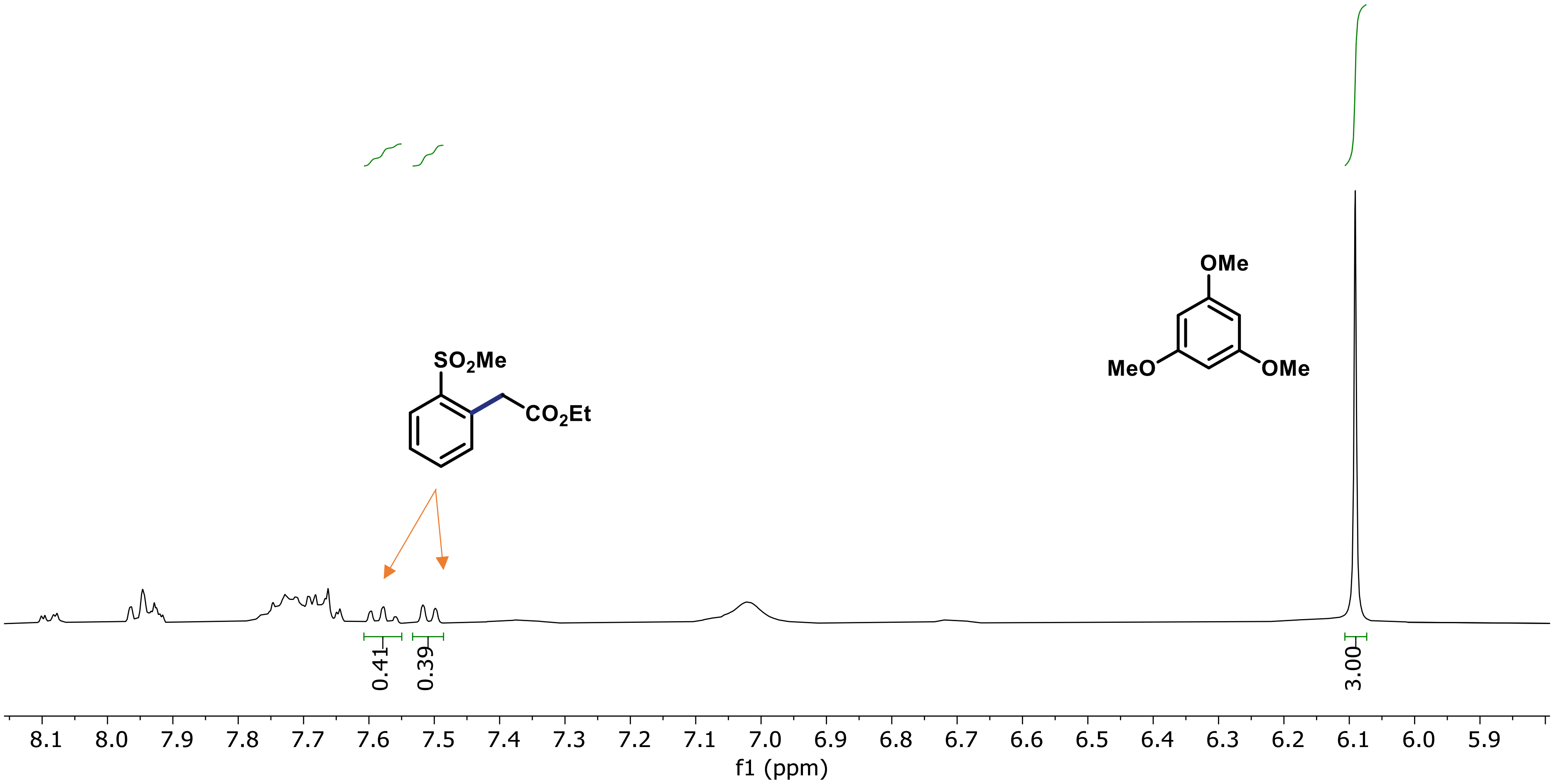

**$^{1}$H NMR of ethyl 2-(4-nitrophenyl)acetate (11)**

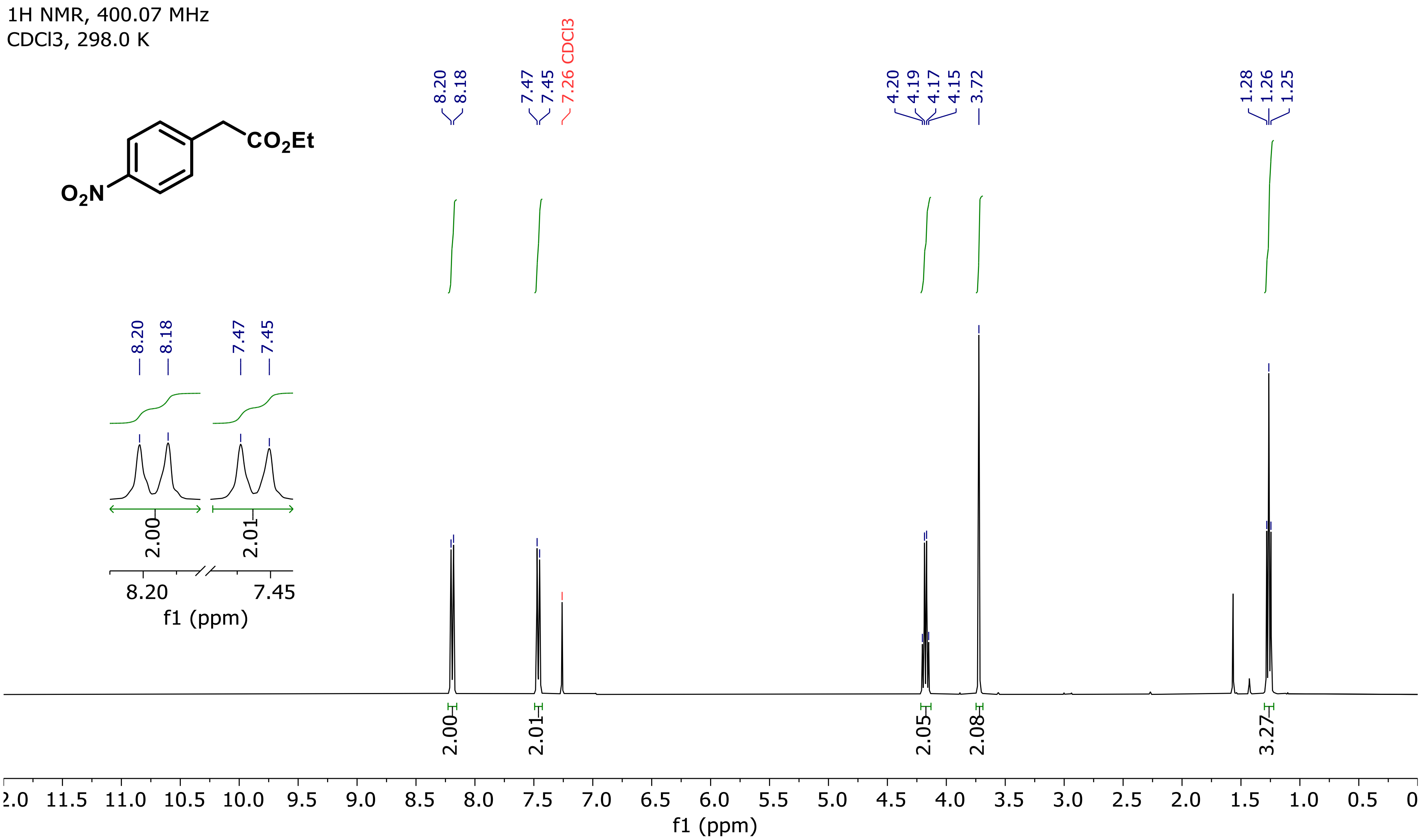

**$^{13}$C NMR of ethyl 2-(4-nitrophenyl)acetate (11)**

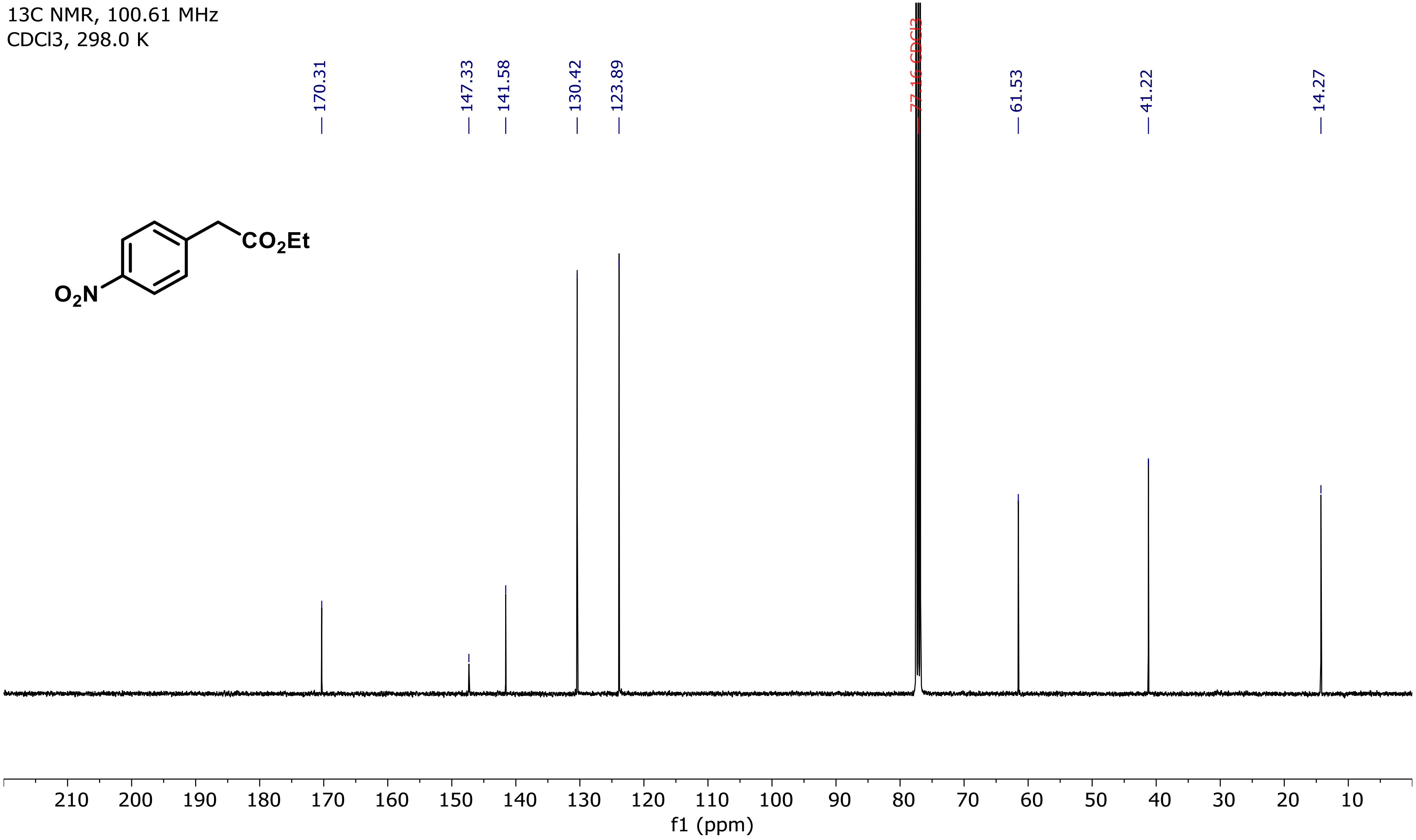

**$^{1}$H NMR of ethyl 2-(3-nitrophenyl)acetate (12)**

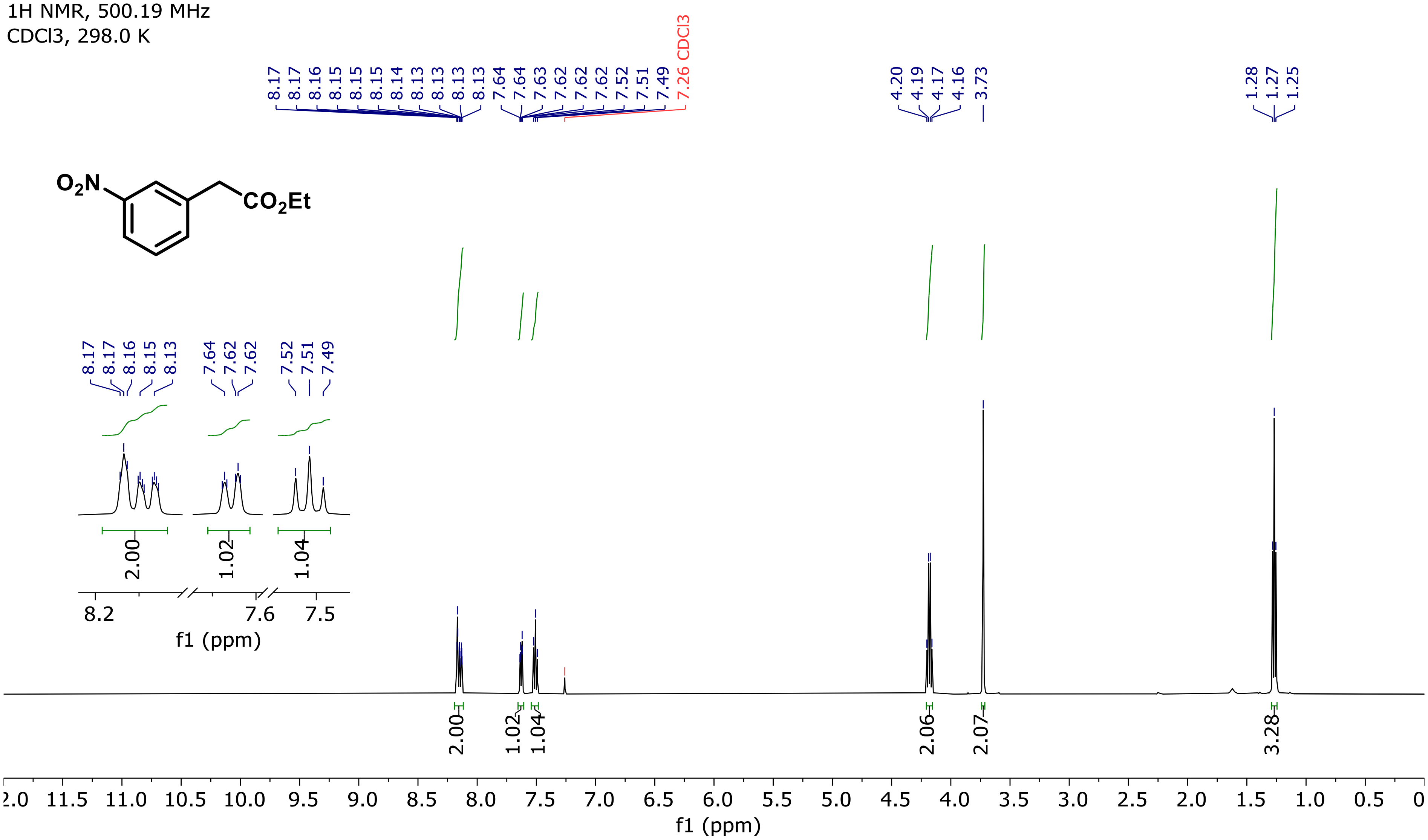

## $^{13}$C NMR of ethyl 2-(3-nitrophenyl)acetate (12)

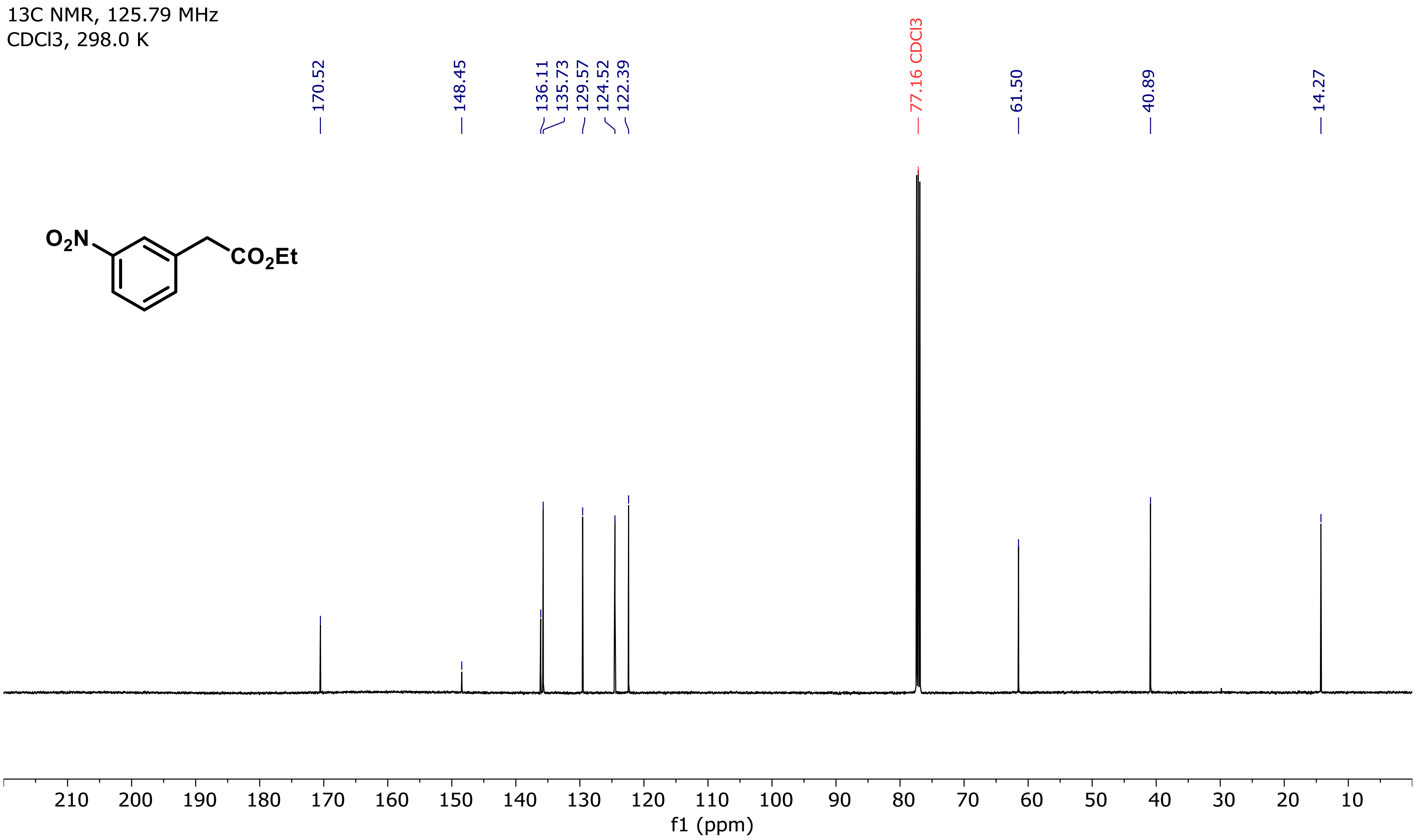

**$^1$H NMR of ethyl 2-(2-nitrophenyl)acetate (13)**

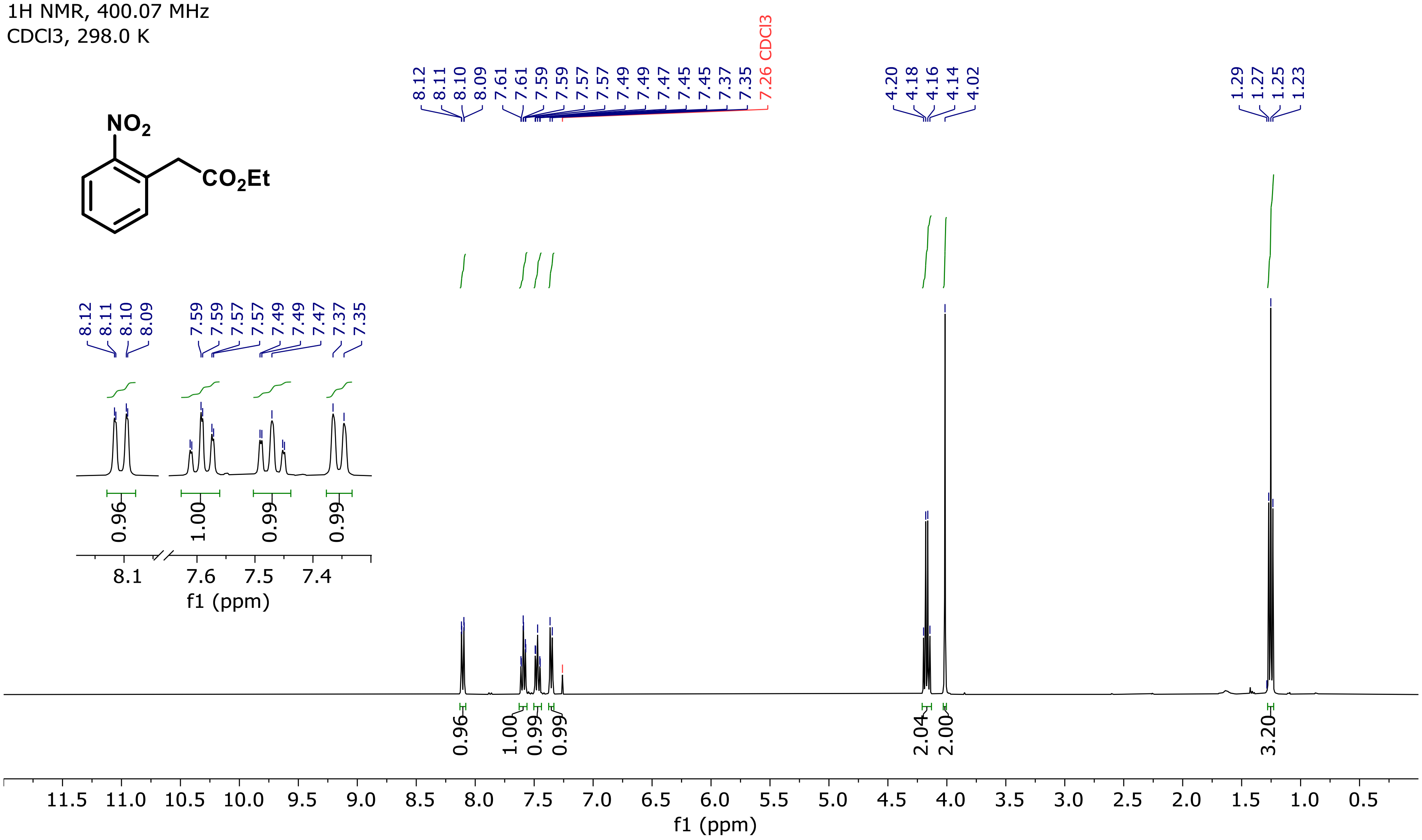

**$^{13}$C NMR of ethyl 2-(2-nitrophenyl)acetate (13)**

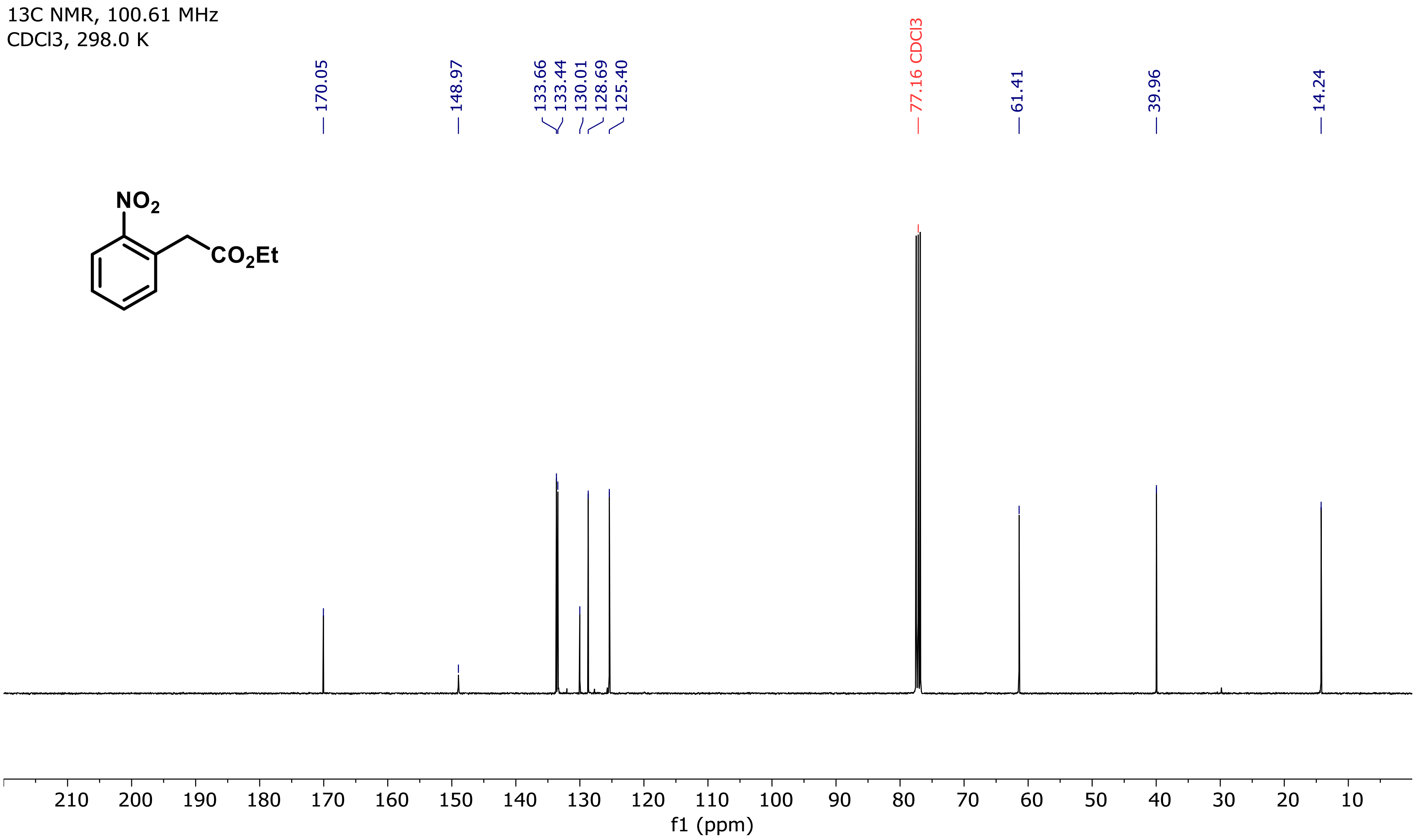

**$^{1}$H NMR of ethyl 2-(4-acetylphenyl)acetate (14)**

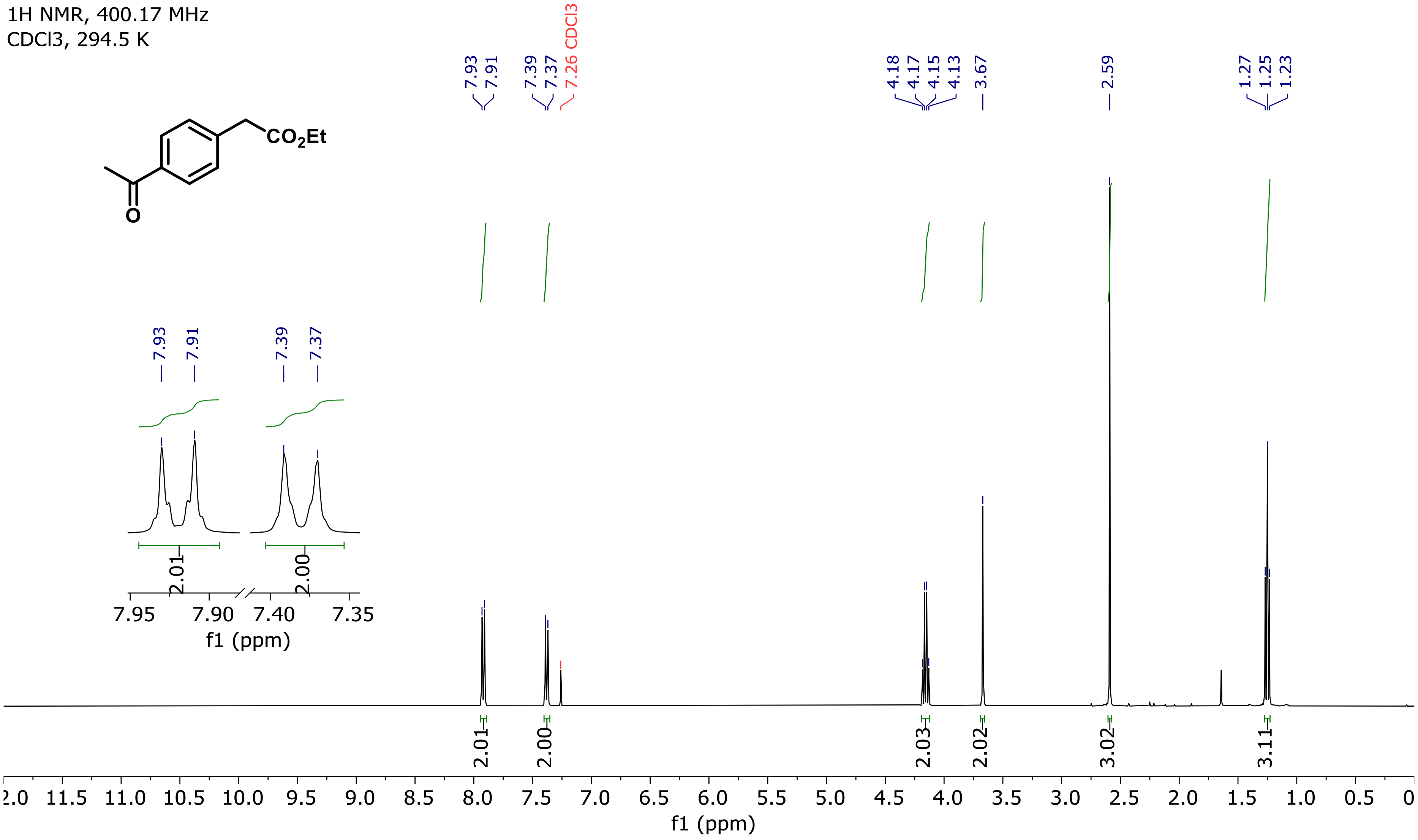

**$^{13}$C NMR of ethyl 2-(4-acetylphenyl)acetate (14)**

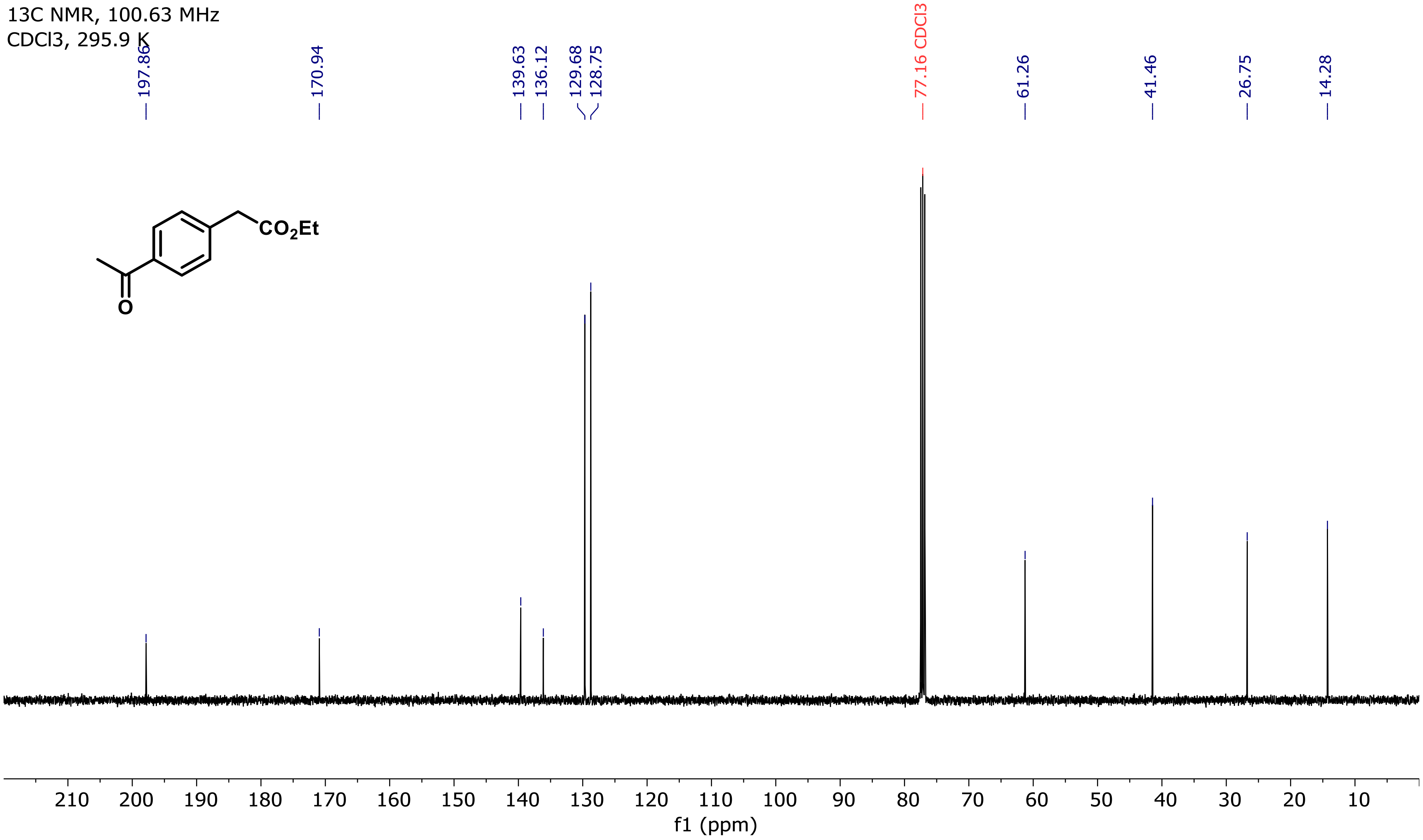

**$^{1}$H NMR of ethyl 2-(4-benzoylphenyl)acetate (15)**

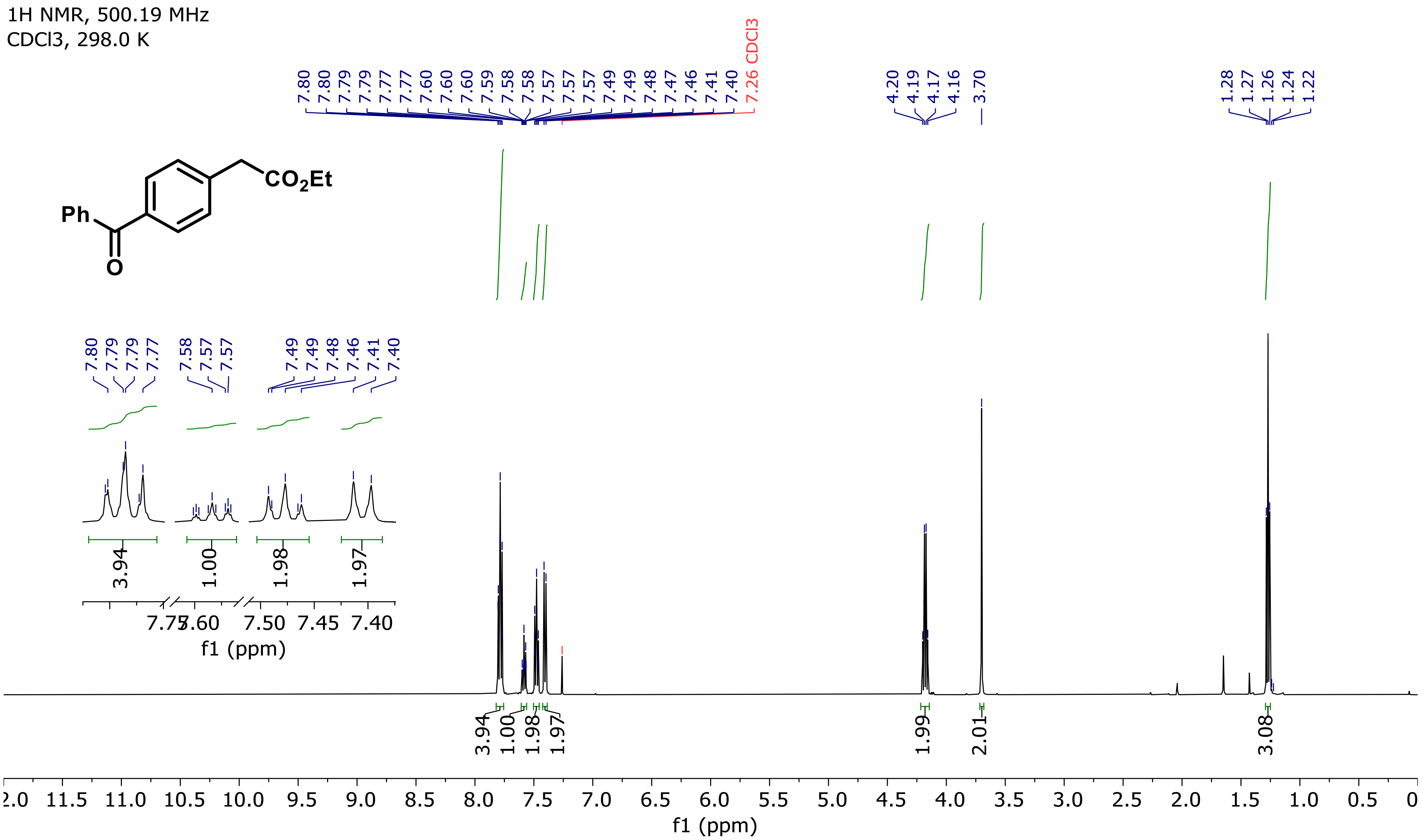

**$^{13}$C NMR of ethyl 2-(4-benzoylphenyl)acetate (15)**

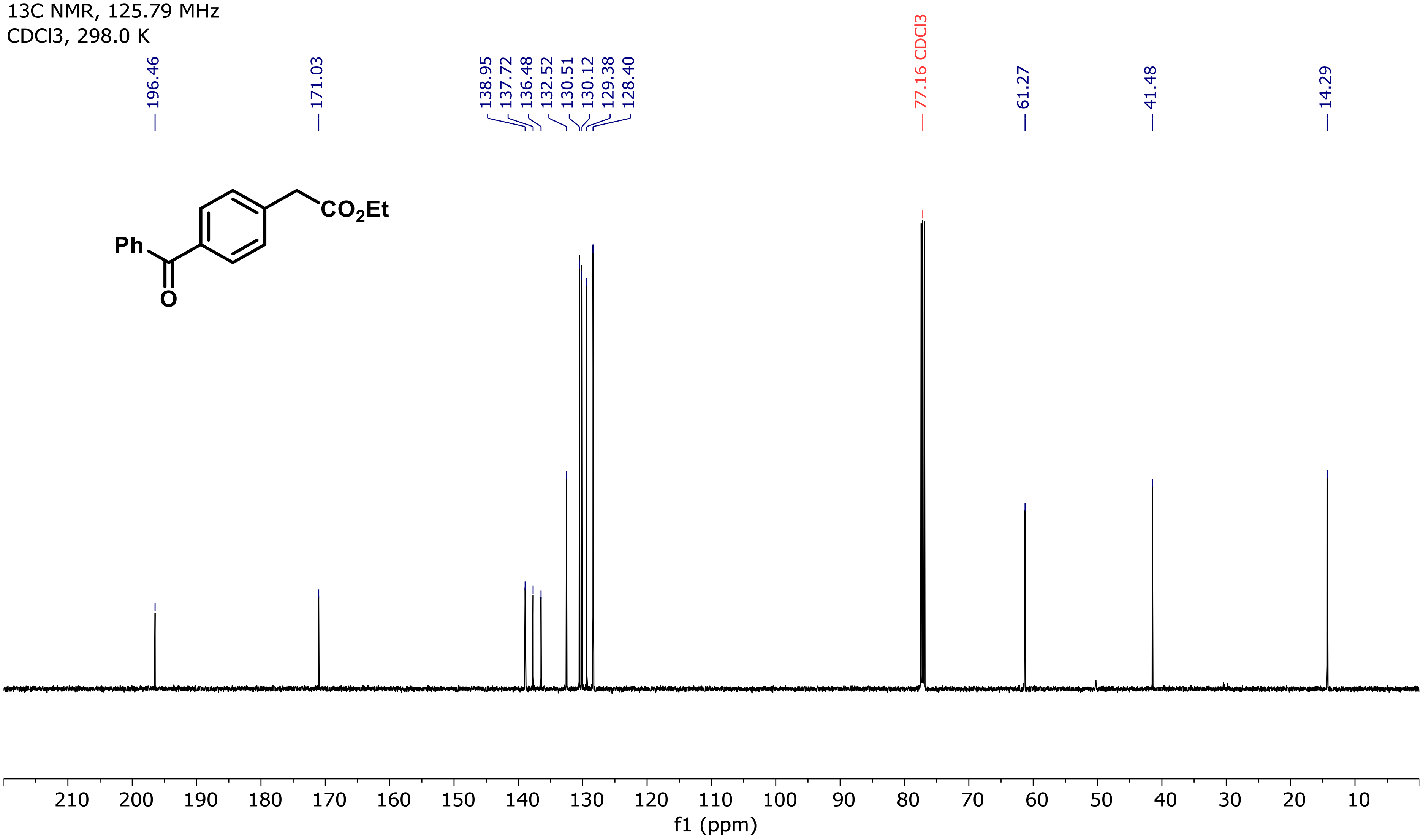

**$^{1}$H NMR of ethyl 2-(1-oxo-2,3-dihydro-1*H*-inden-5-yl)acetate (16)**

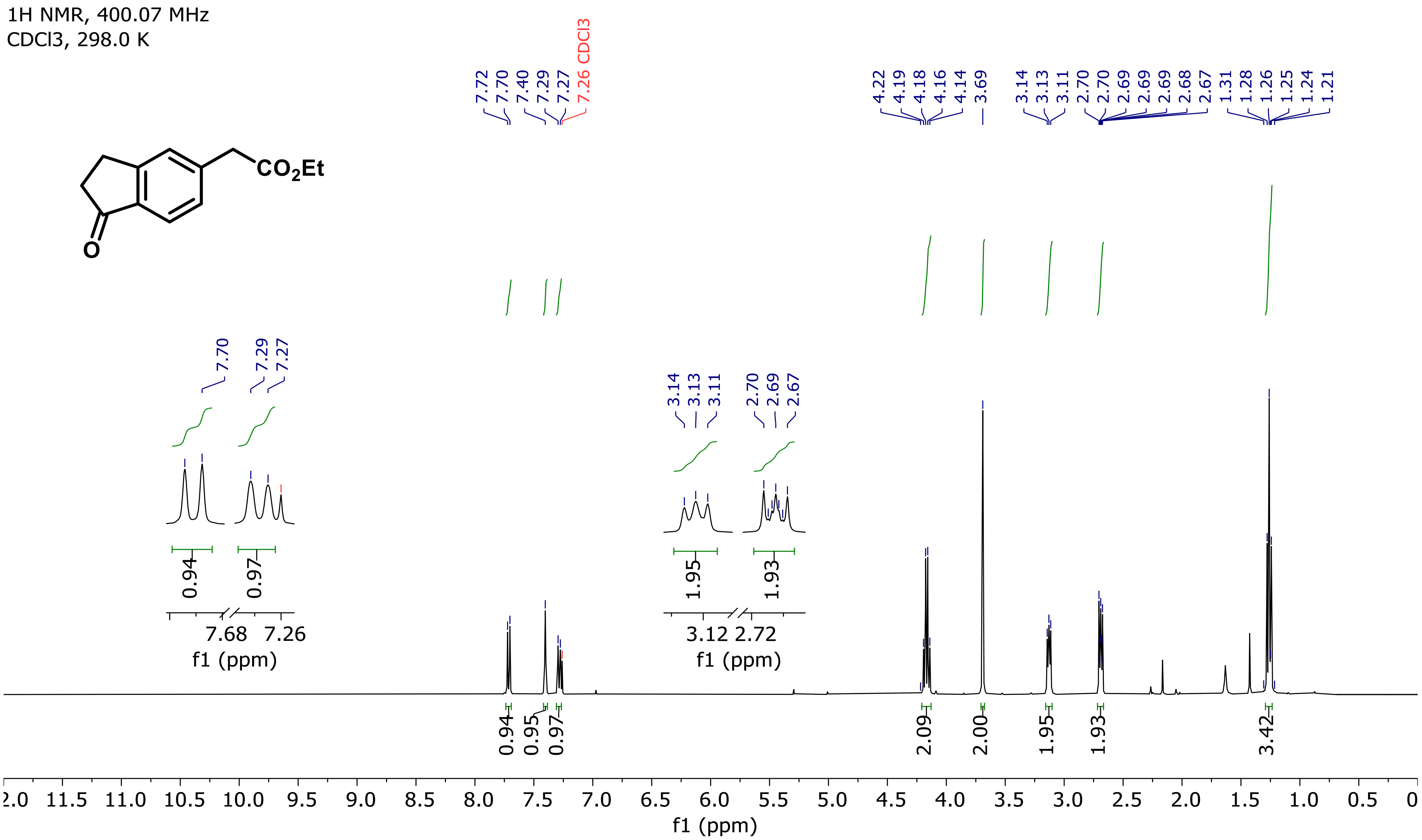

**$^{13}$C NMR of ethyl 2-(1-oxo-2,3-dihydro-1*H*-inden-5-yl)acetate (16)**

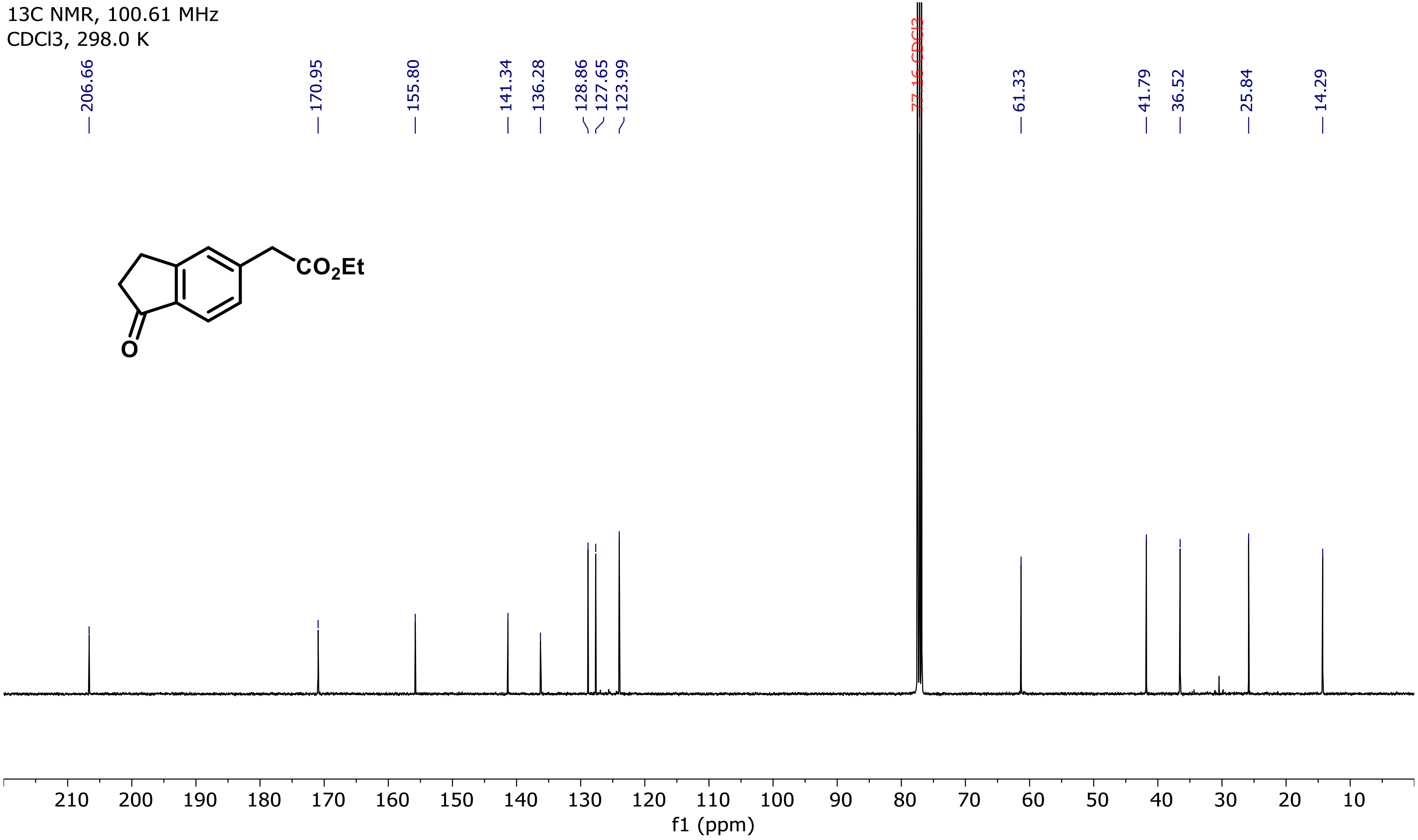

**$^{1}$H NMR of methyl 4-(2-ethoxy-2-oxoethyl)benzoate (17)**

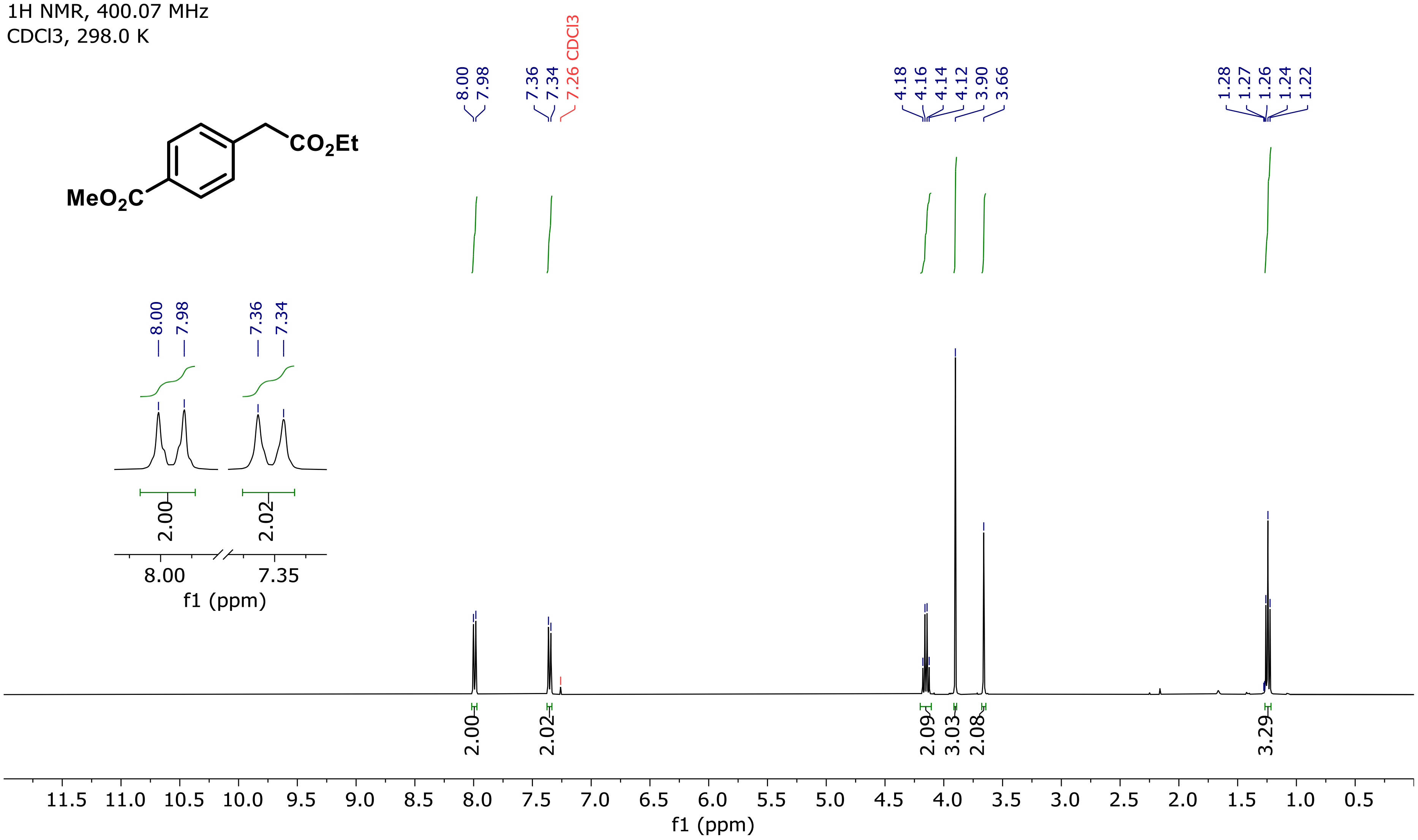

**$^{13}$C NMR of methyl 4-(2-ethoxy-2-oxoethyl)benzoate (17)**

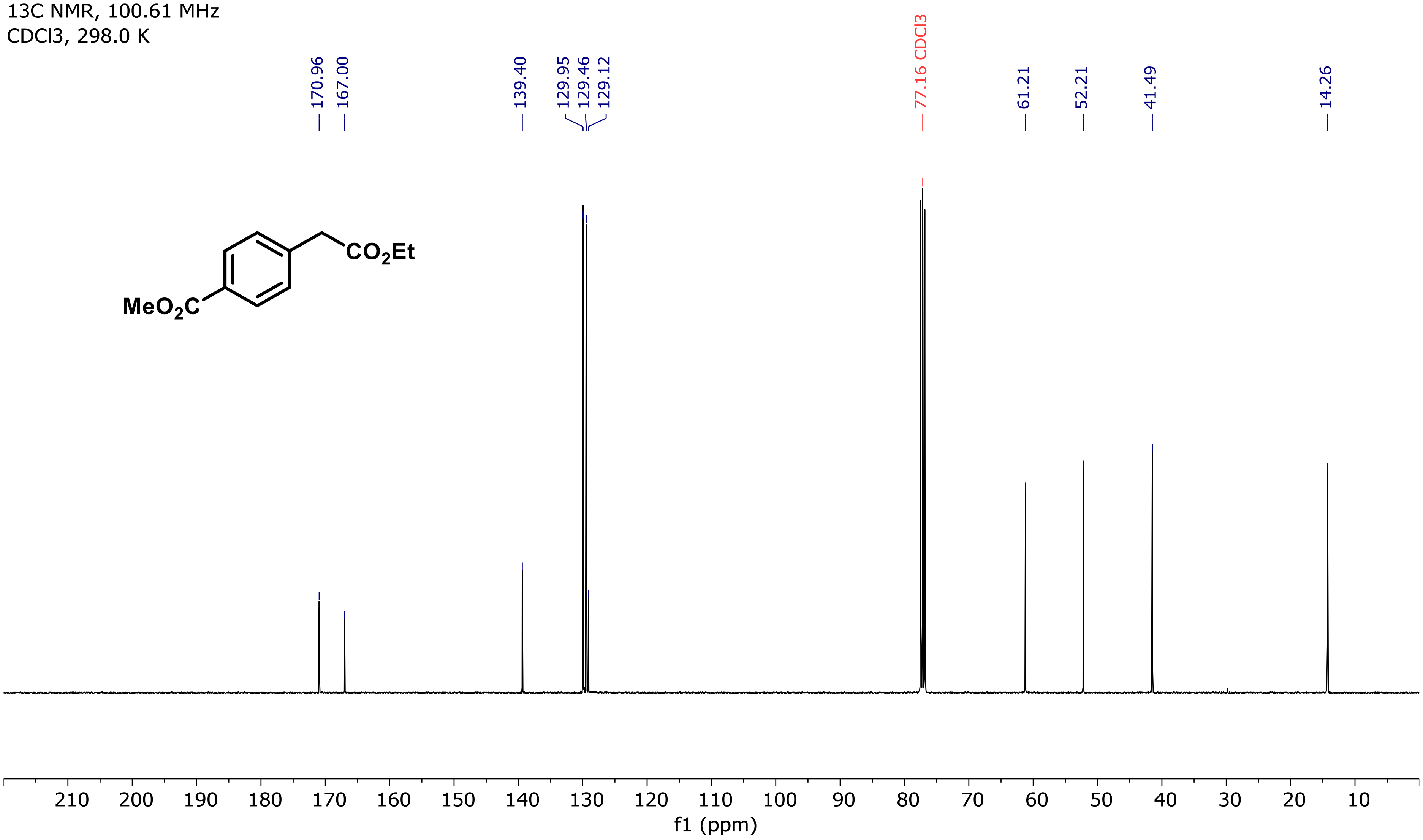

**$^{1}$H NMR of ethyl 4-(2-ethoxy-2-oxoethyl)benzoate (18)**

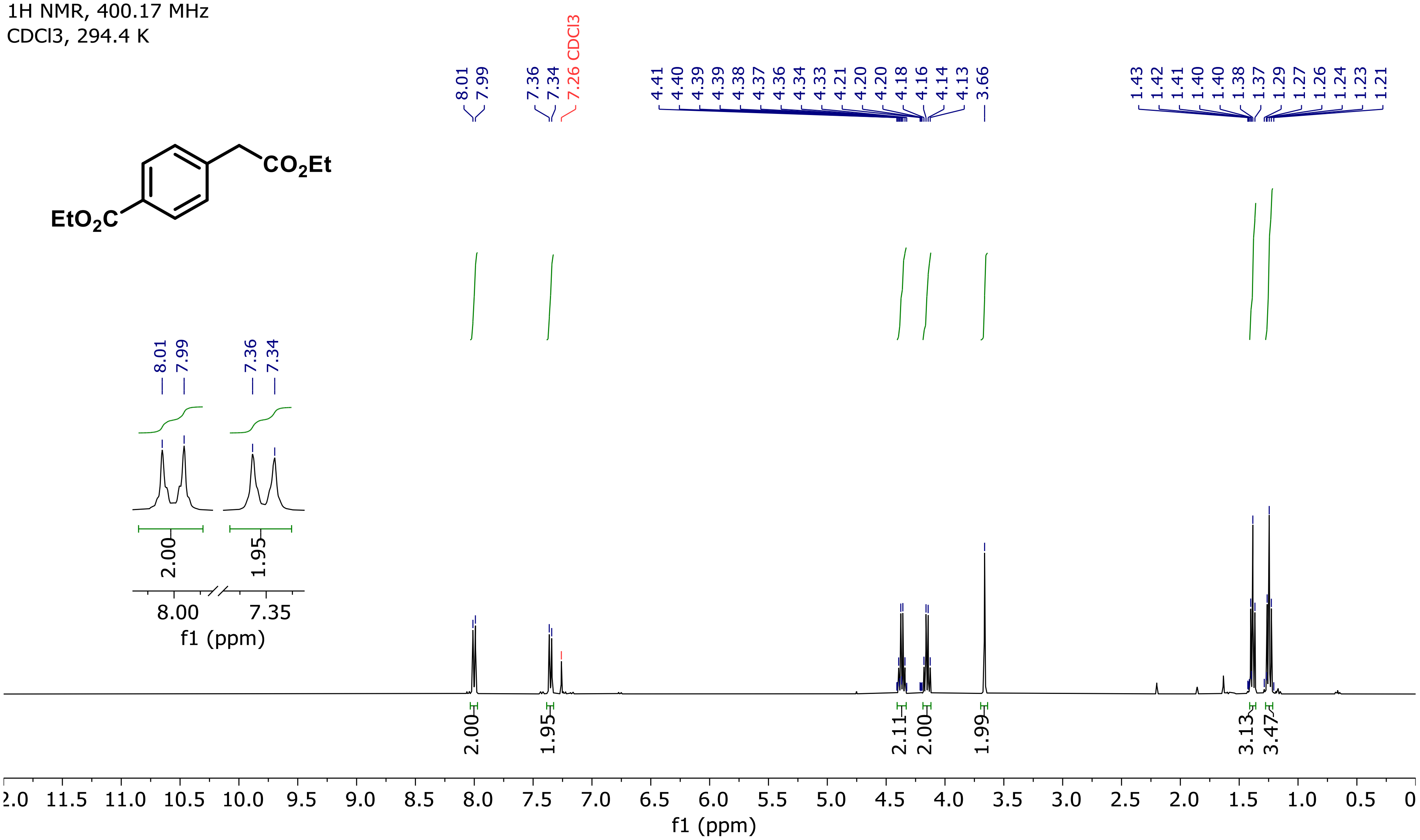

**$^{13}$C NMR of ethyl 4-(2-ethoxy-2-oxoethyl)benzoate (18)**

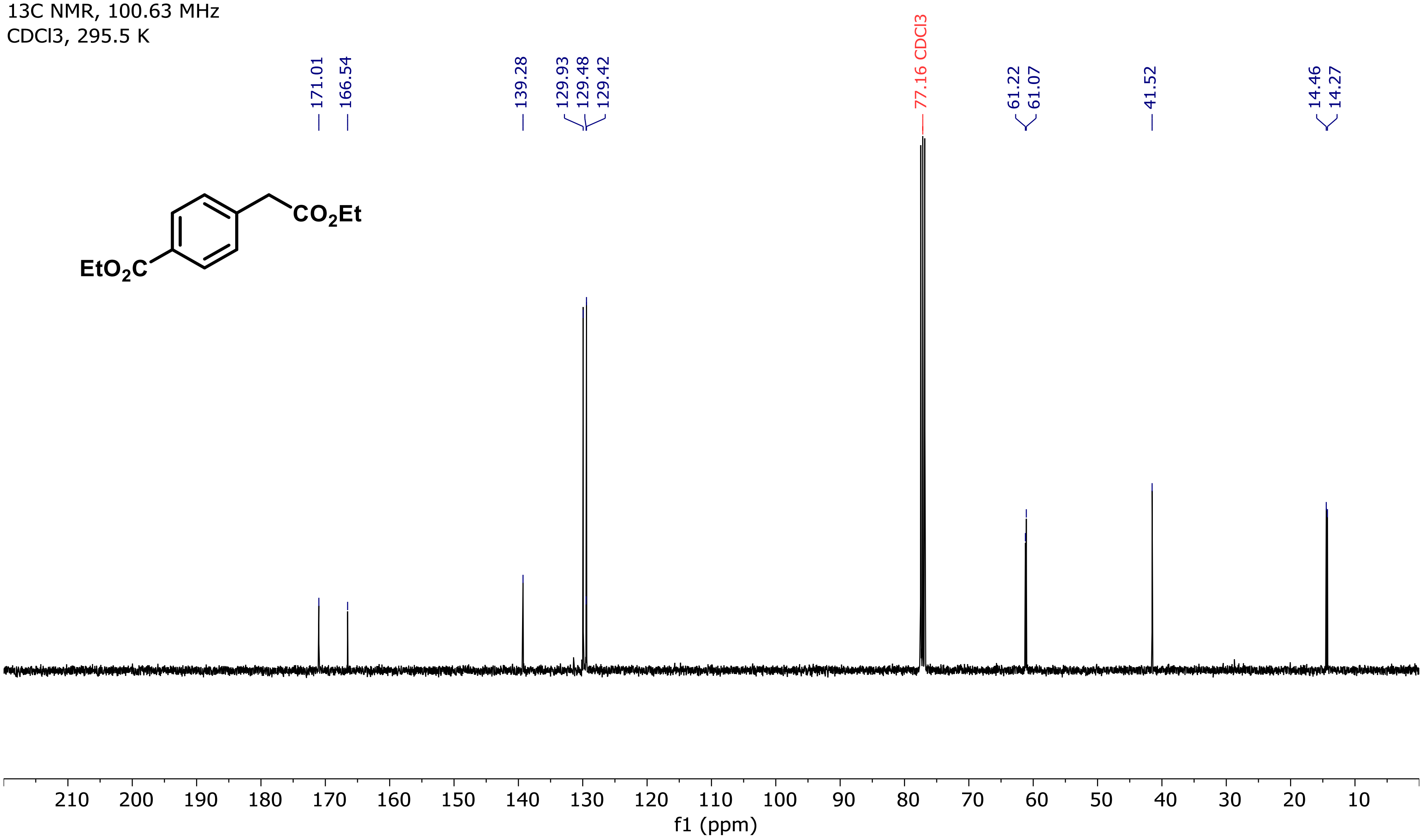

**$^{1}$H NMR of ethyl 2-(4-carbamoyl-2-nitrophenyl)acetate (19)**

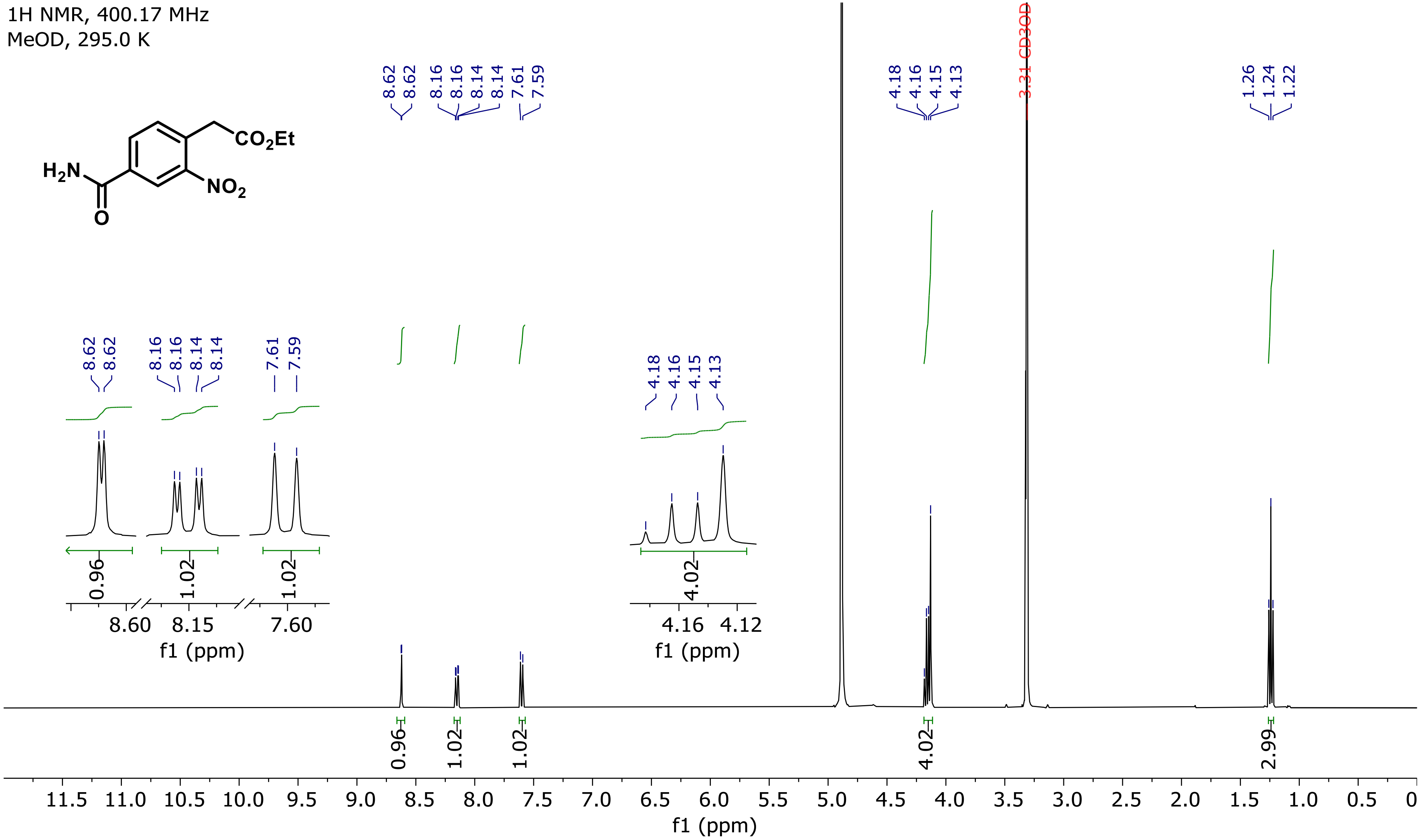

**$^{13}$C NMR of ethyl 2-(4-carbamoyl-2-nitrophenyl)acetate (19)**

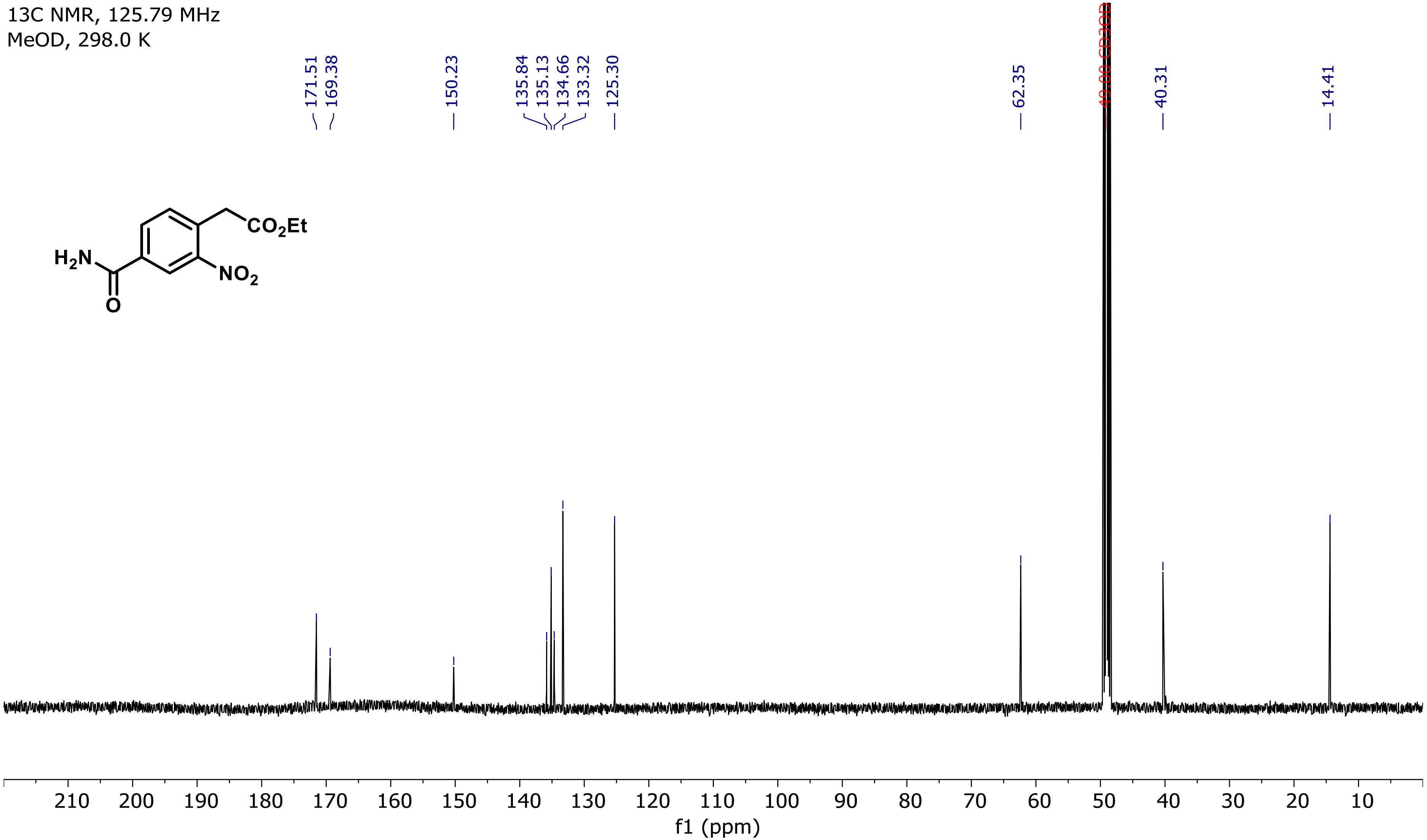

**$^{1}$H NMR ethyl 2-(4-acetyl-3-fluorophenyl)acetate (20)**

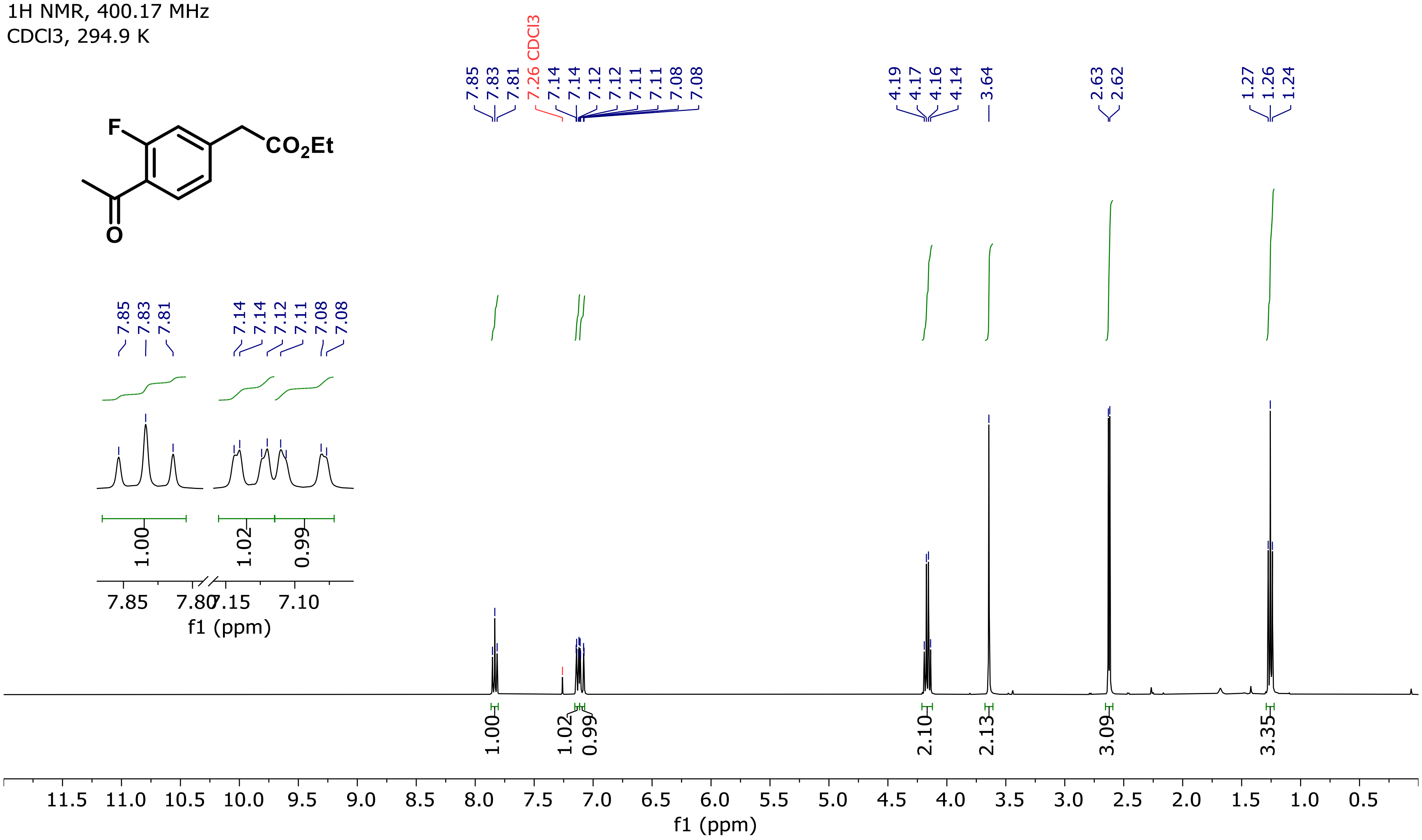

**$^{13}$C NMR ethyl 2-(4-acetyl-3-fluorophenyl)acetate (20)**

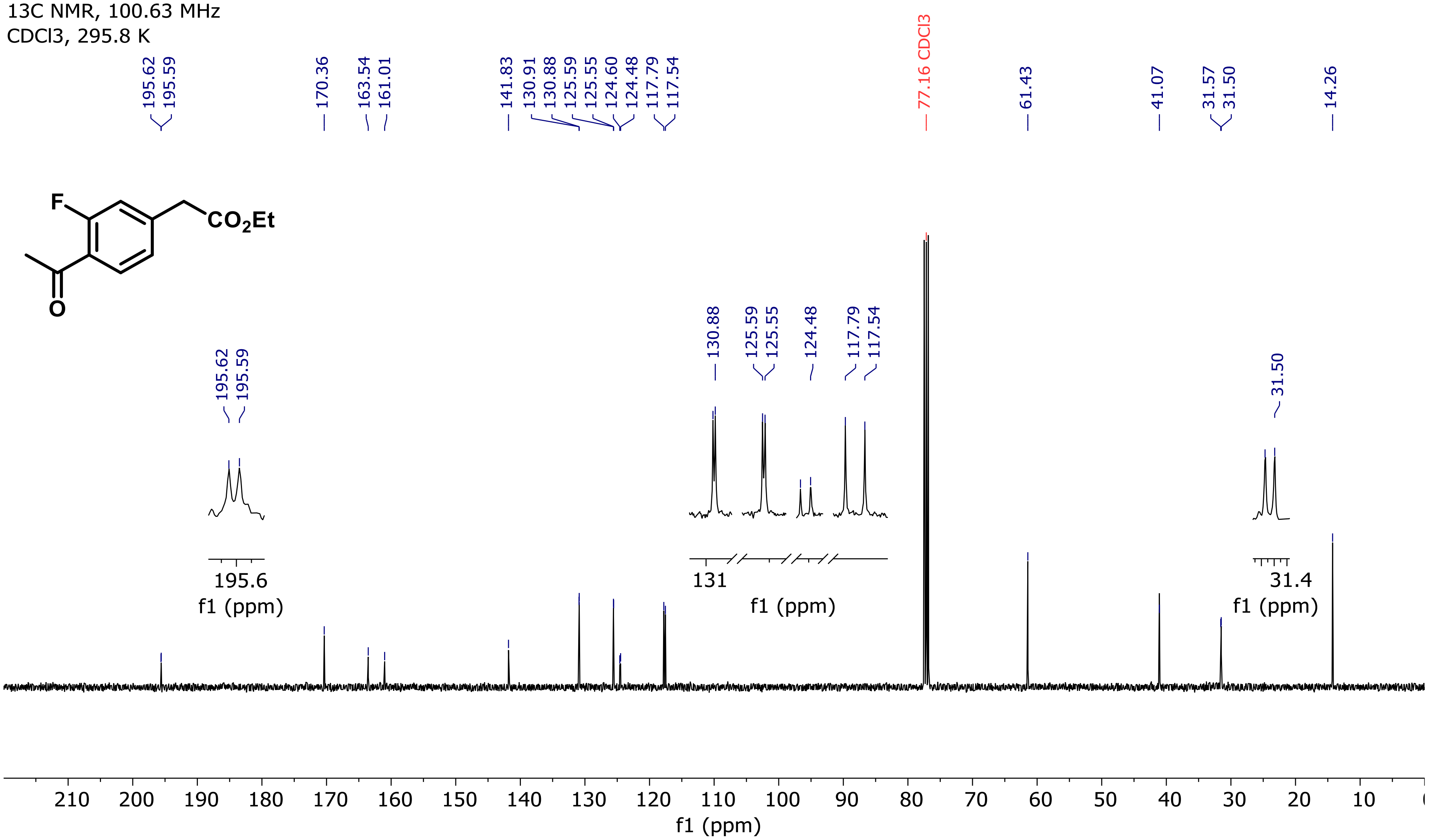

**$^{19}$F NMR ethyl 2-(4-acetyl-3-fluorophenyl)acetate (20)**

19F NMR, 376.50 MHz
CDCl3, 294.3 K

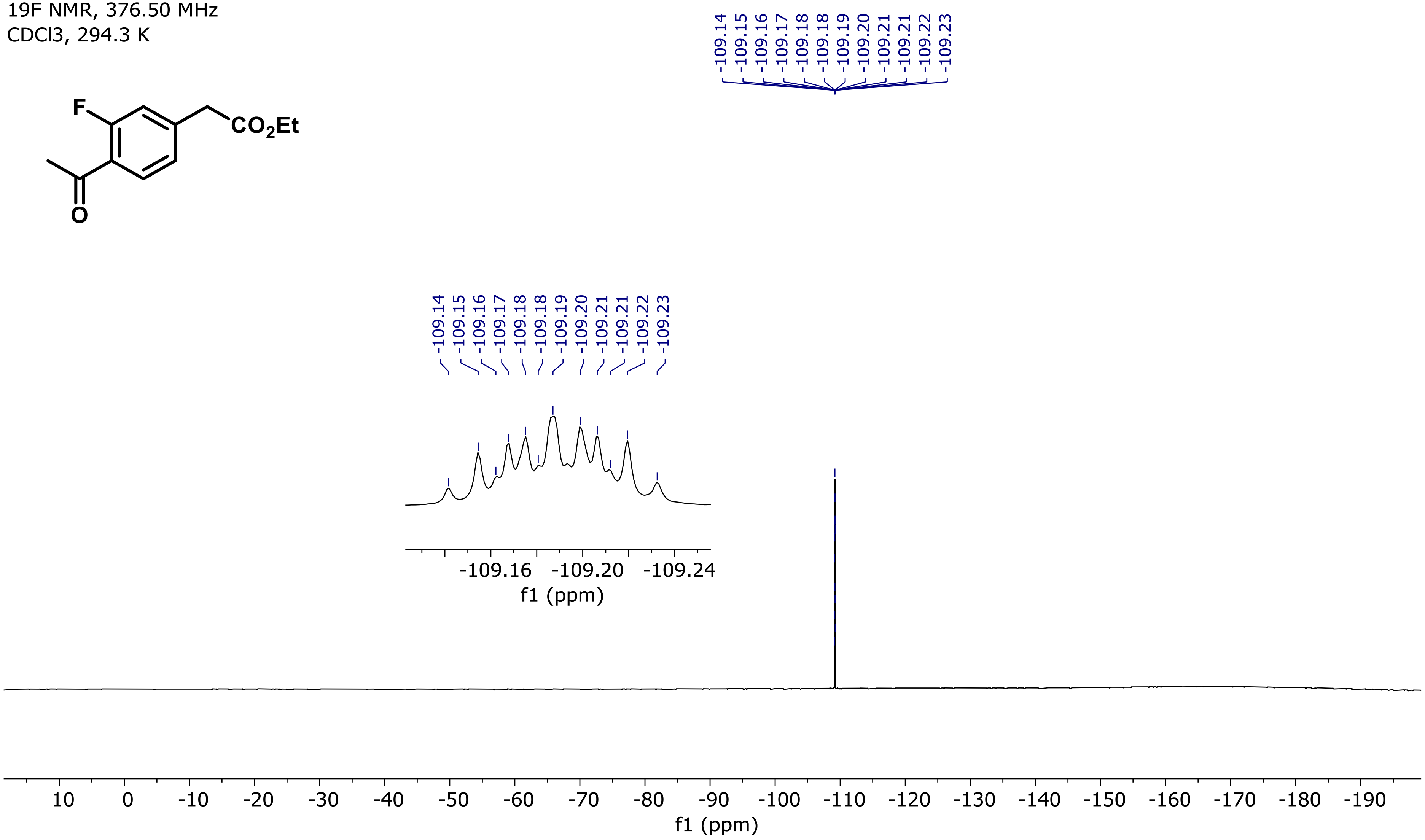

**$^{1}$H NMR ethyl 2-(3,5-bis(trifluoromethyl)phenyl)acetate (21)**

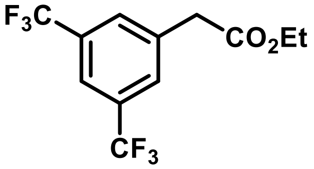

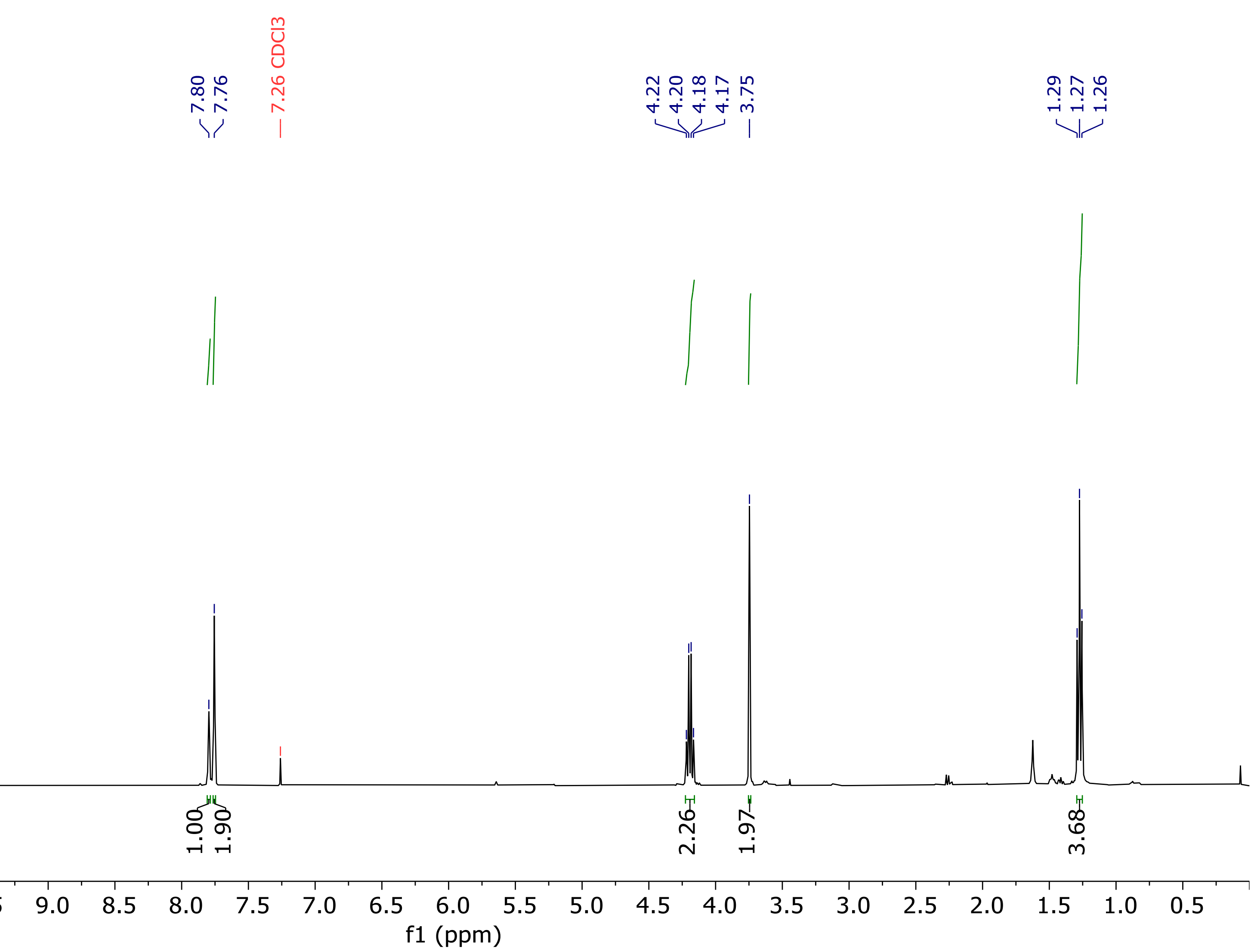

## $^{13}C$ NMR ethyl 2-(3,5-bis(trifluoromethyl)phenyl)acetate (21)

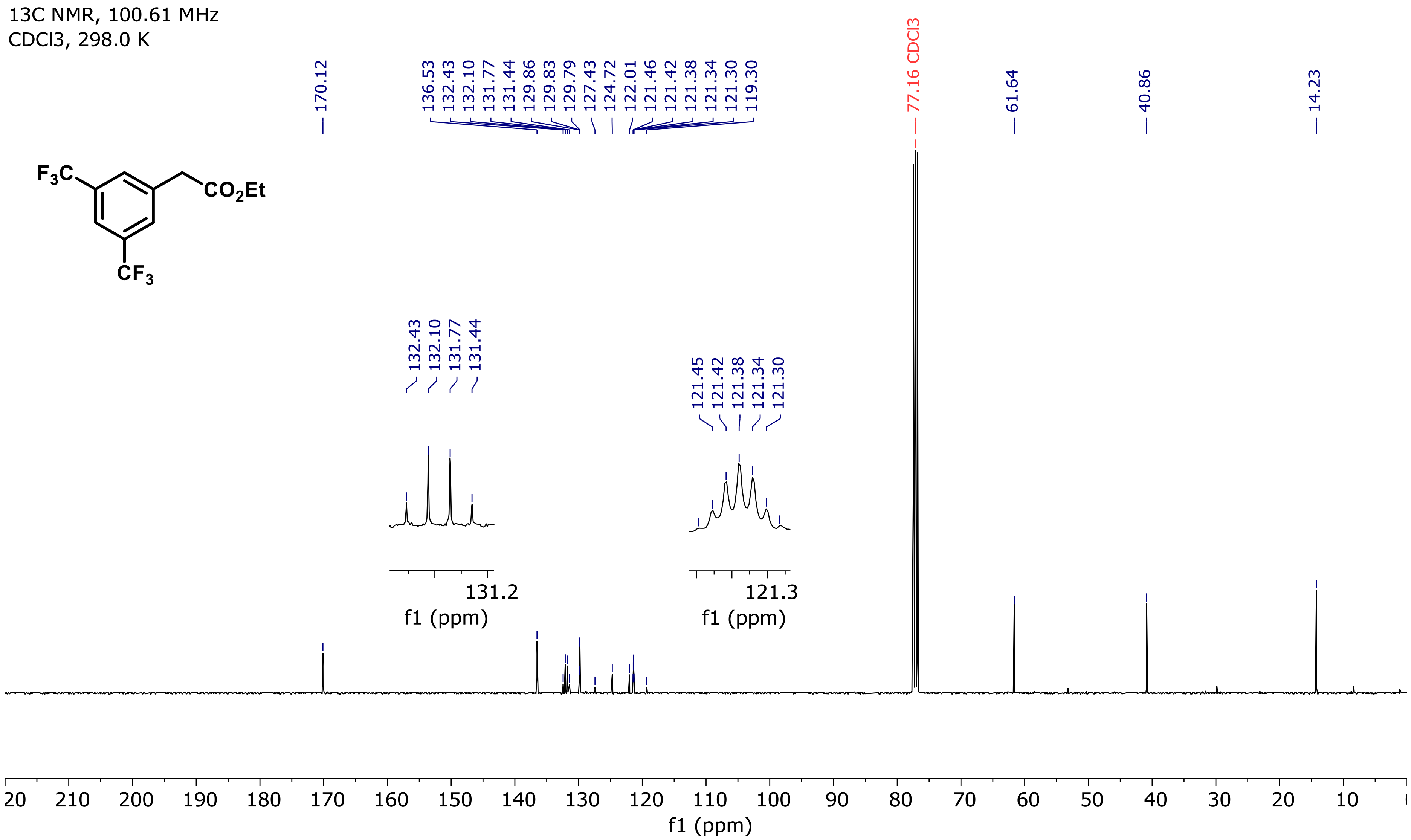

**$^{19}$F NMR ethyl 2-(3,5-bis(trifluoromethyl)phenyl)acetate (21)**

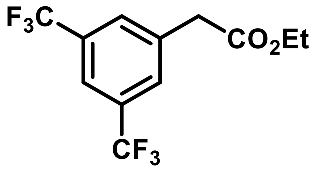


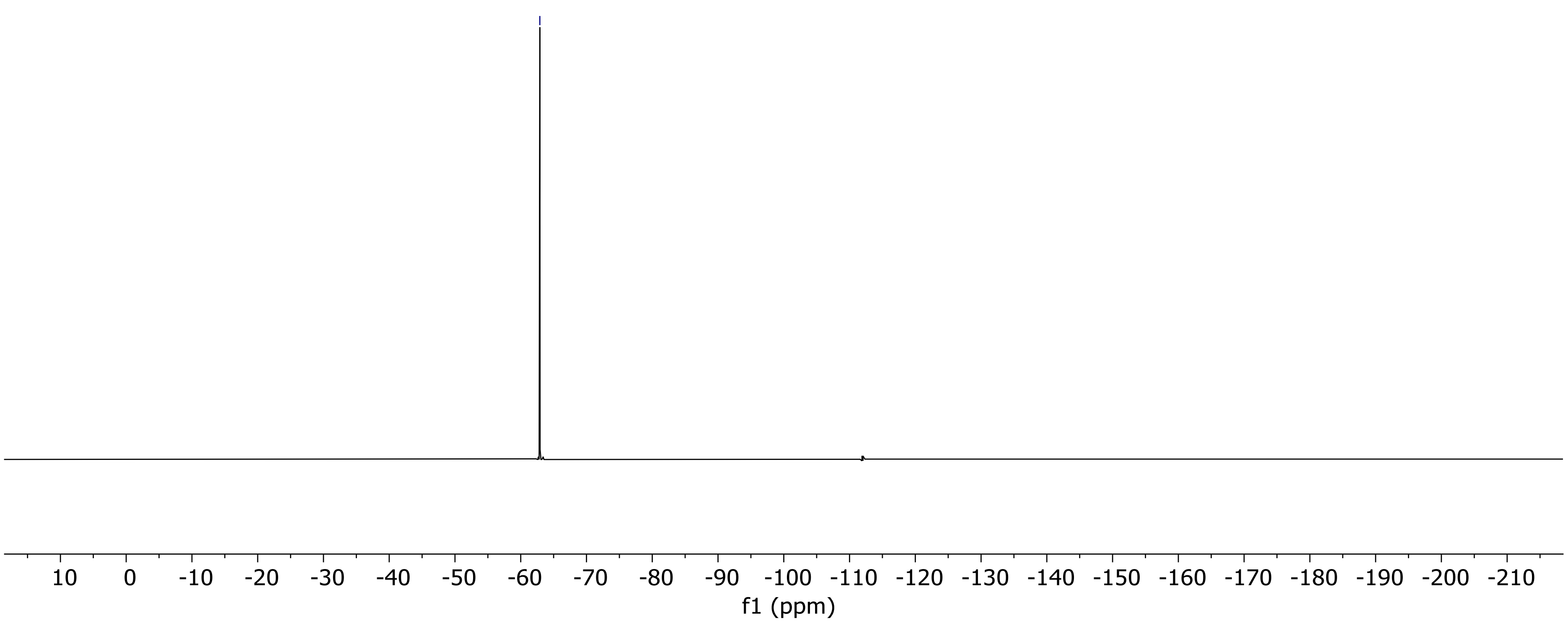

**$^{1}$H NMR of ethyl 2-(2-fluoro-[1,1'-biphenyl]-4-yl)acetate (22)**

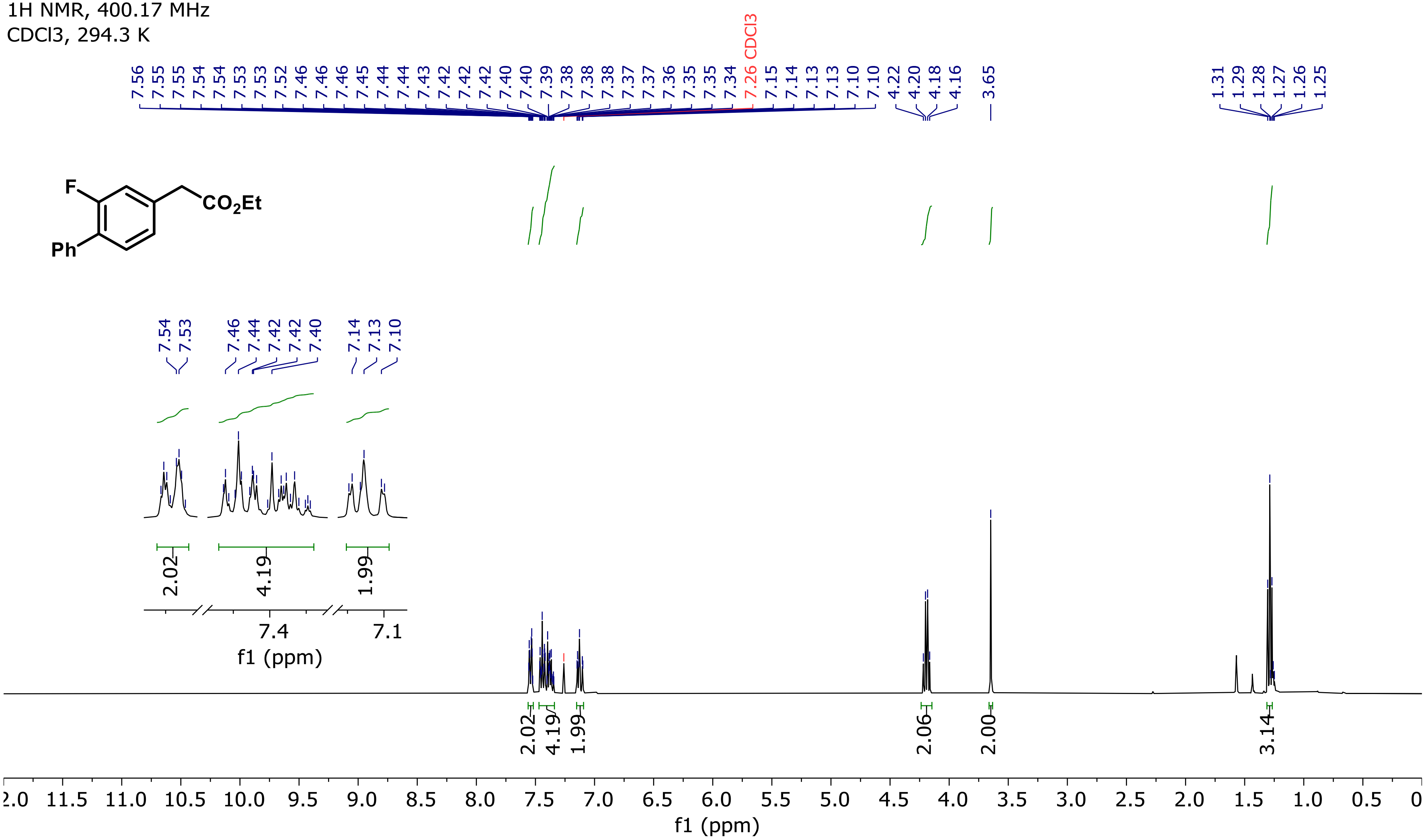

**$^{13}$C NMR of ethyl 2-(2-fluoro-[1,1'-biphenyl]-4-yl)acetate (22)**

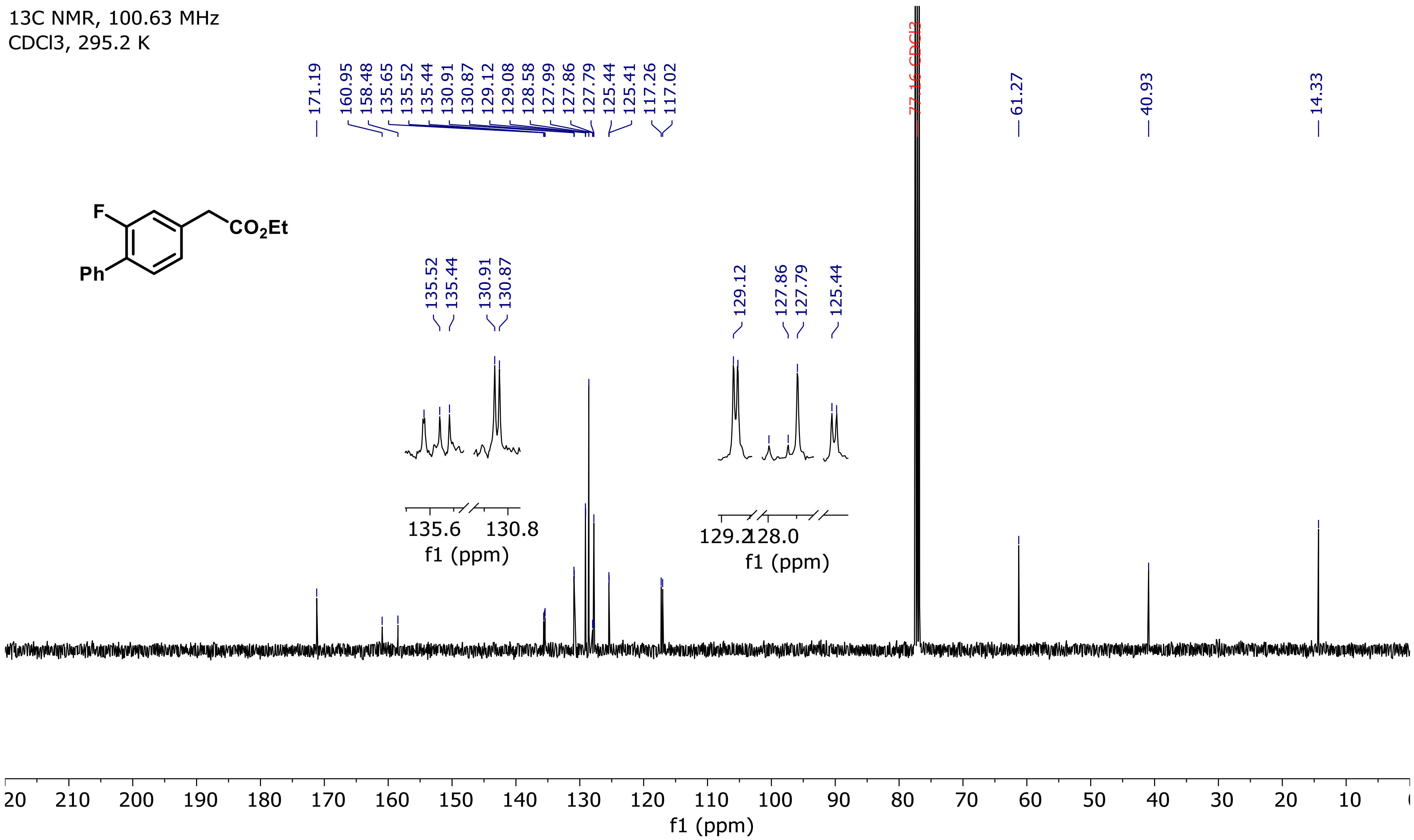

**$^{19}$F NMR of ethyl 2-(2-fluoro-[1,1'-biphenyl]-4-yl)acetate (22)**

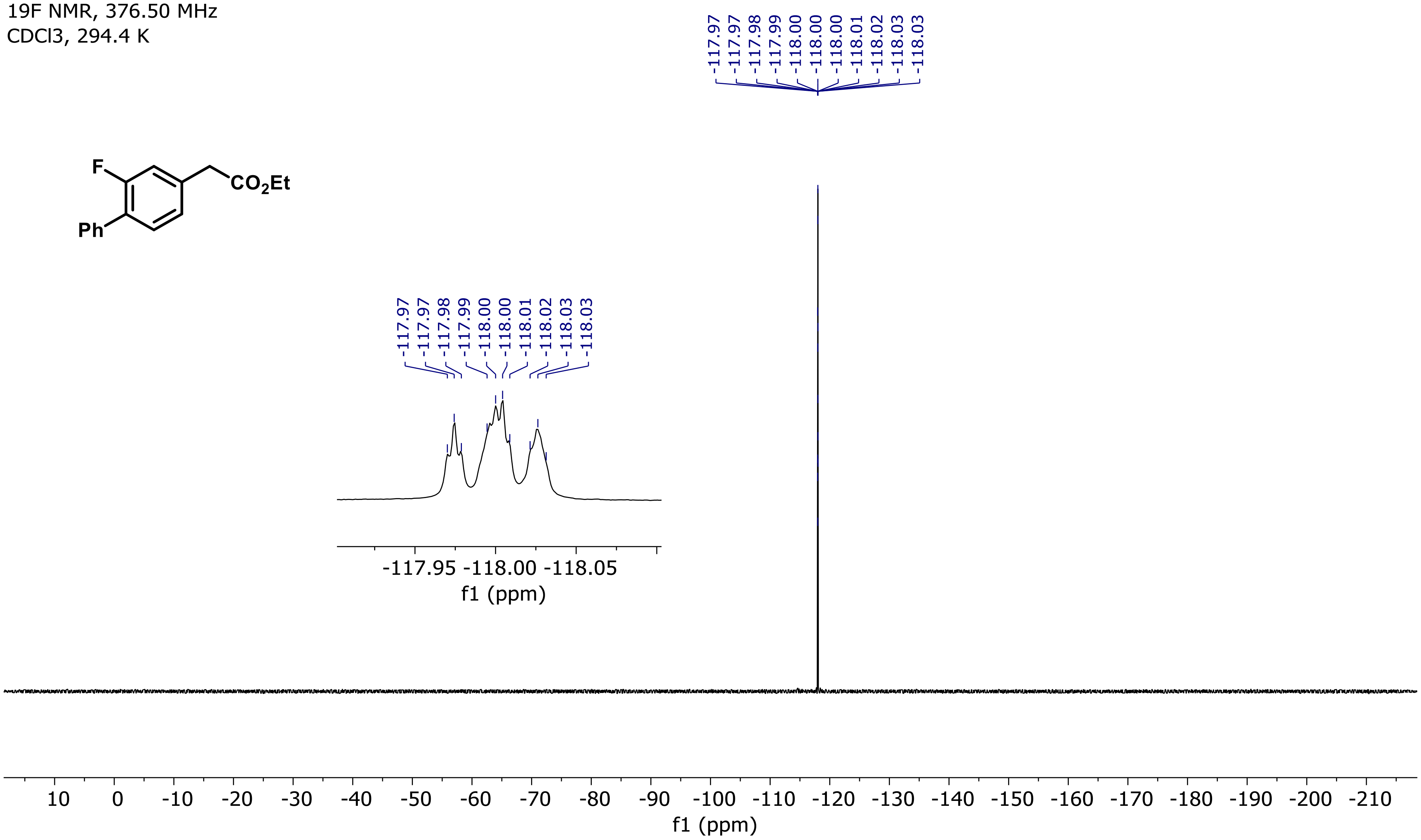

**$^{1}$H NMR used to determine yield of anisole (23)**

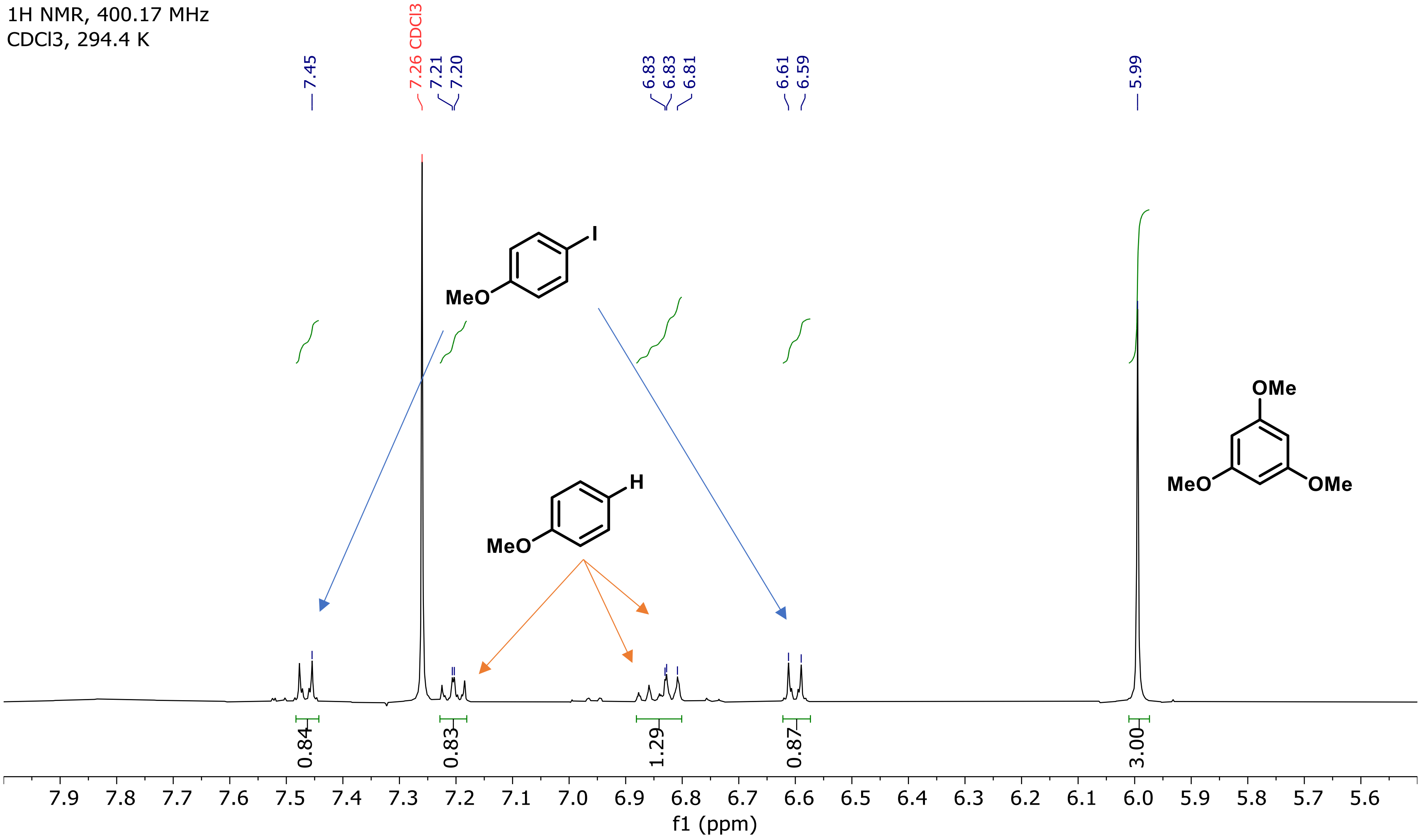

**1H NMR of ethyl 2-(6-(trifluoromethyl)pyridin-3-yl)acetate (24)**

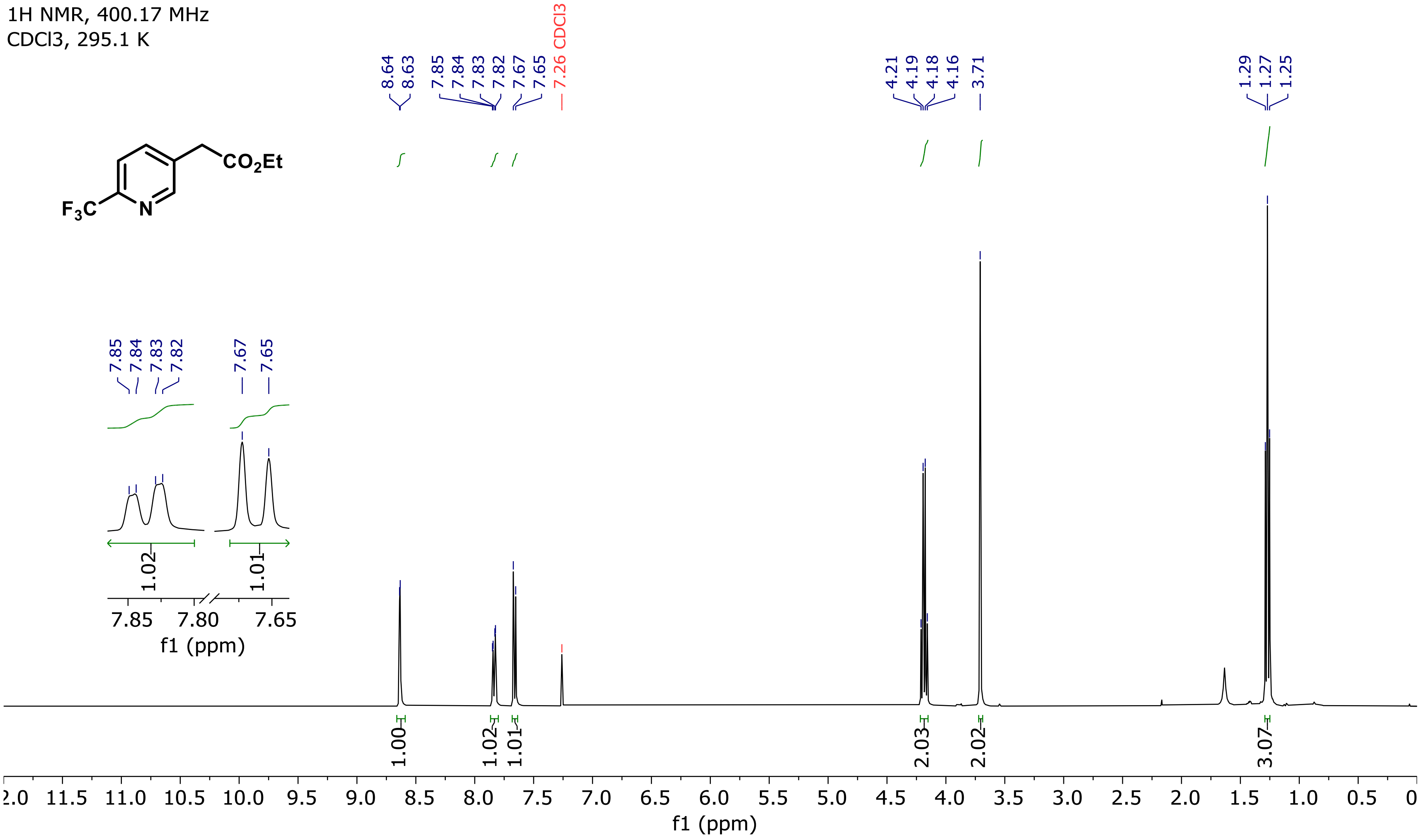

**$^{13}$C NMR of ethyl 2-(6-(trifluoromethyl)pyridin-3-yl)acetate (24)**

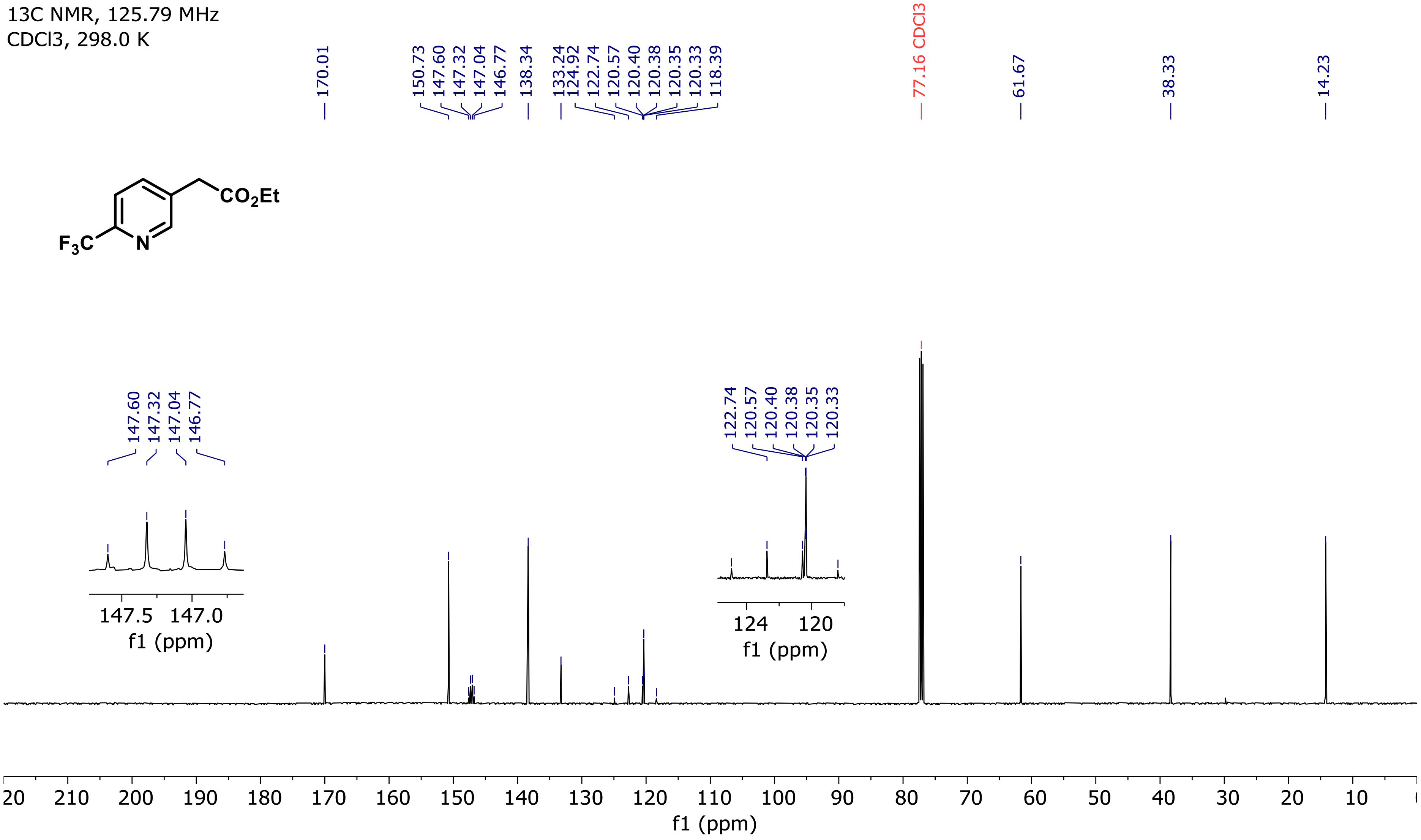

**$^{19}$F NMR of ethyl 2-(6-(trifluoromethyl)pyridin-3-yl)acetate (24)**

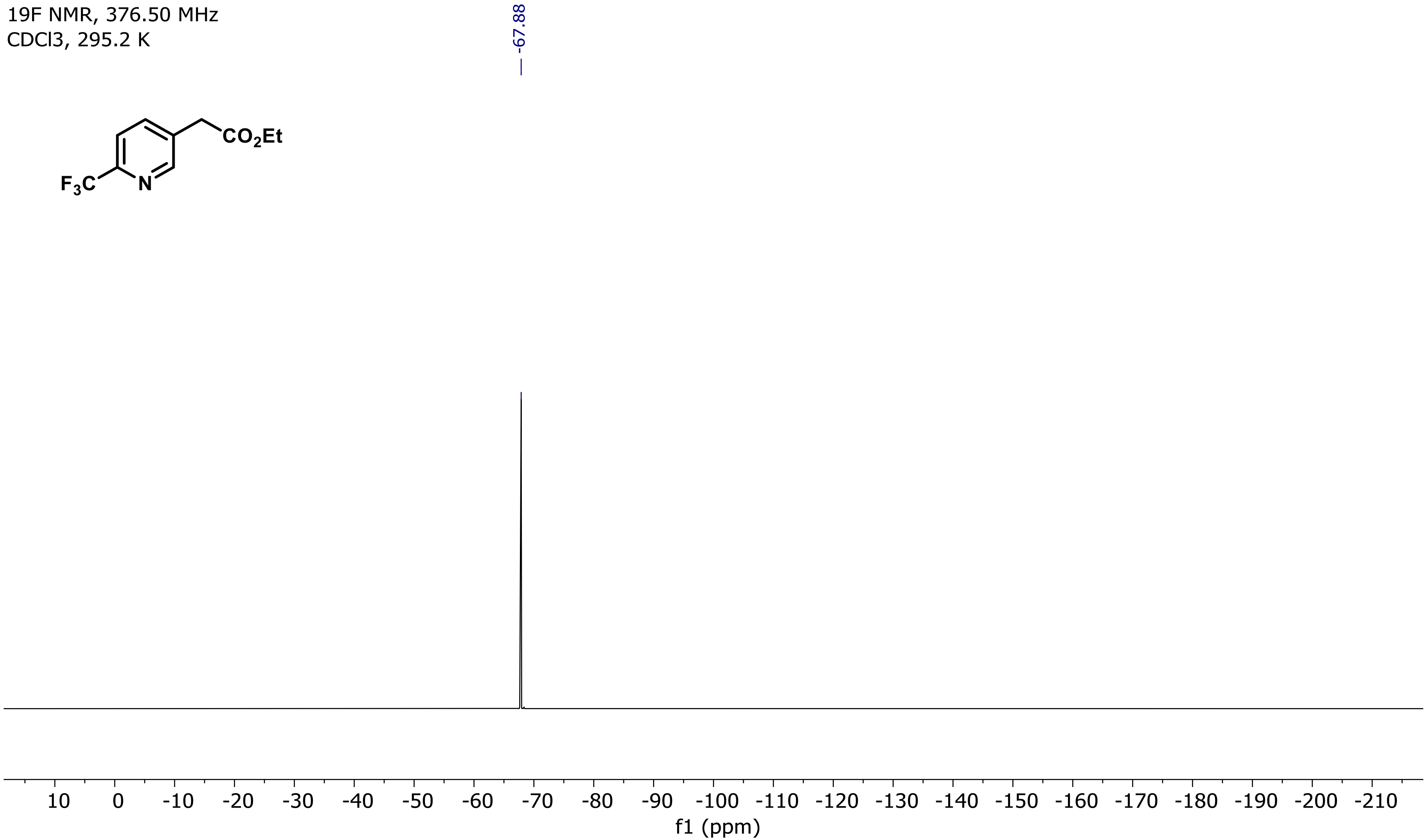

**$^{1}$H NMR of ethyl 2-(6-acetylpyridin-3-yl)acetate (25)**

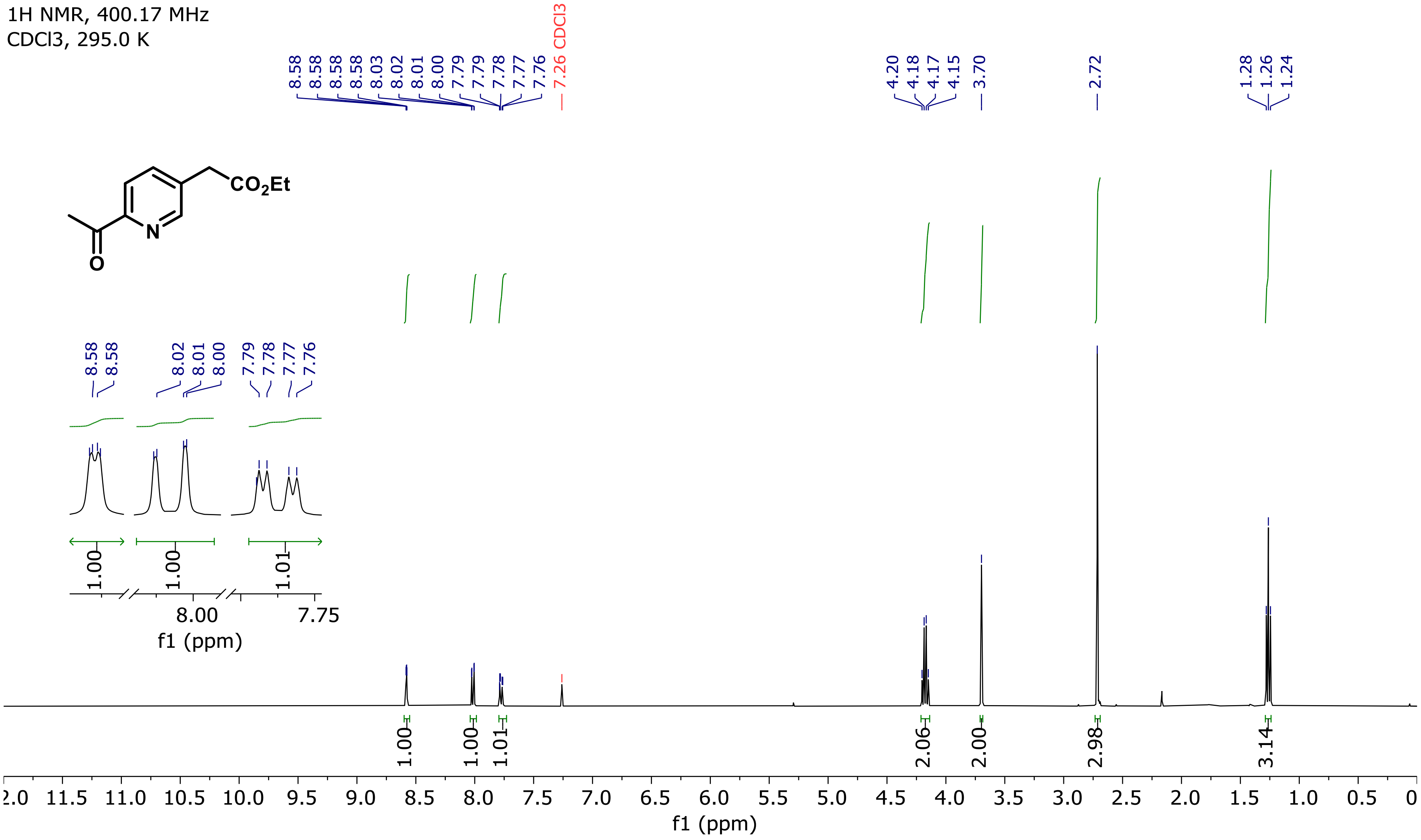

**$^{13}$C NMR of ethyl 2-(6-acetylpyridin-3-yl)acetate (25)**

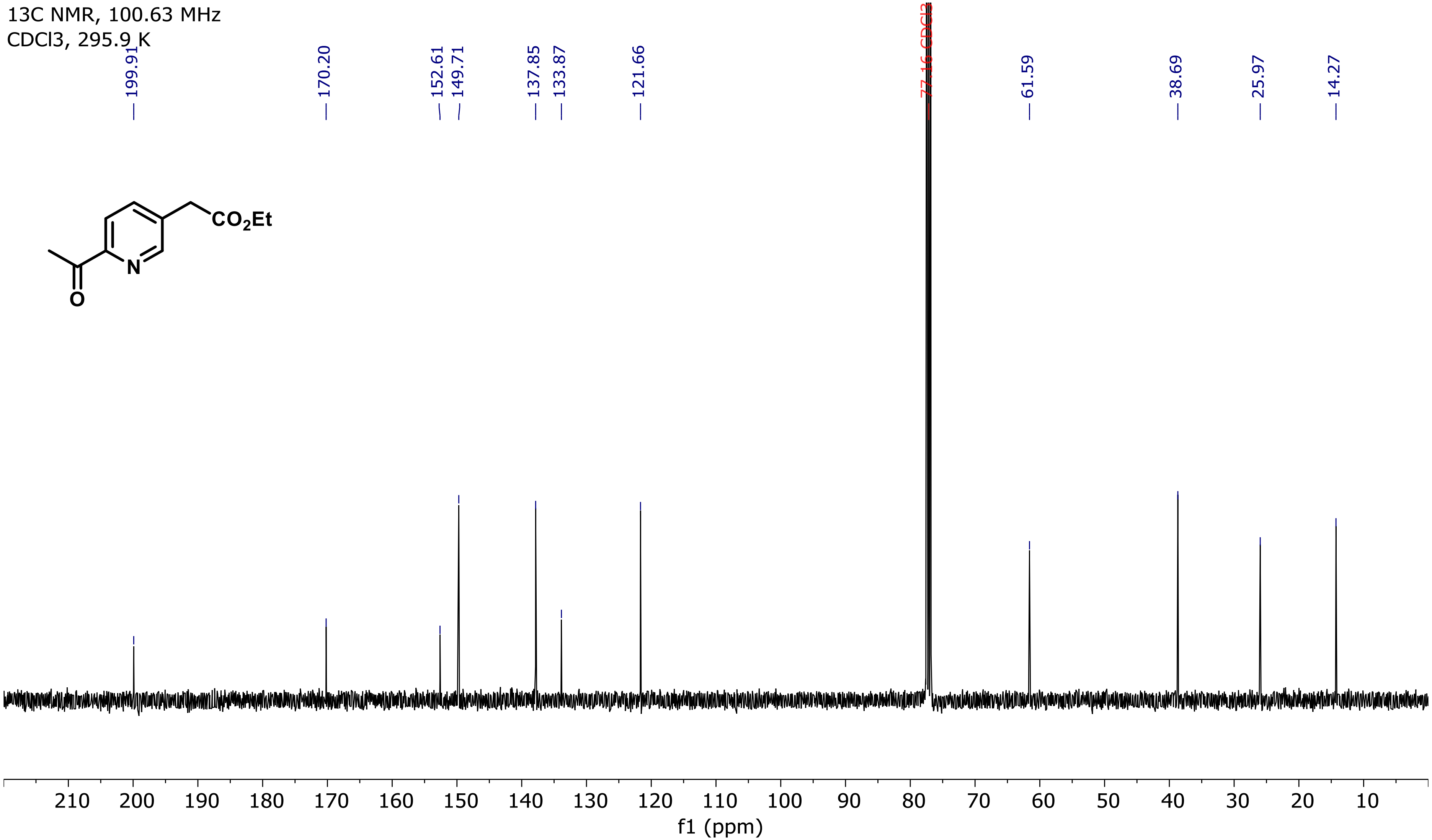

**$^{1}$H NMR of ethyl 2-(6-cyanopyridin-3-yl)acetate (26)**

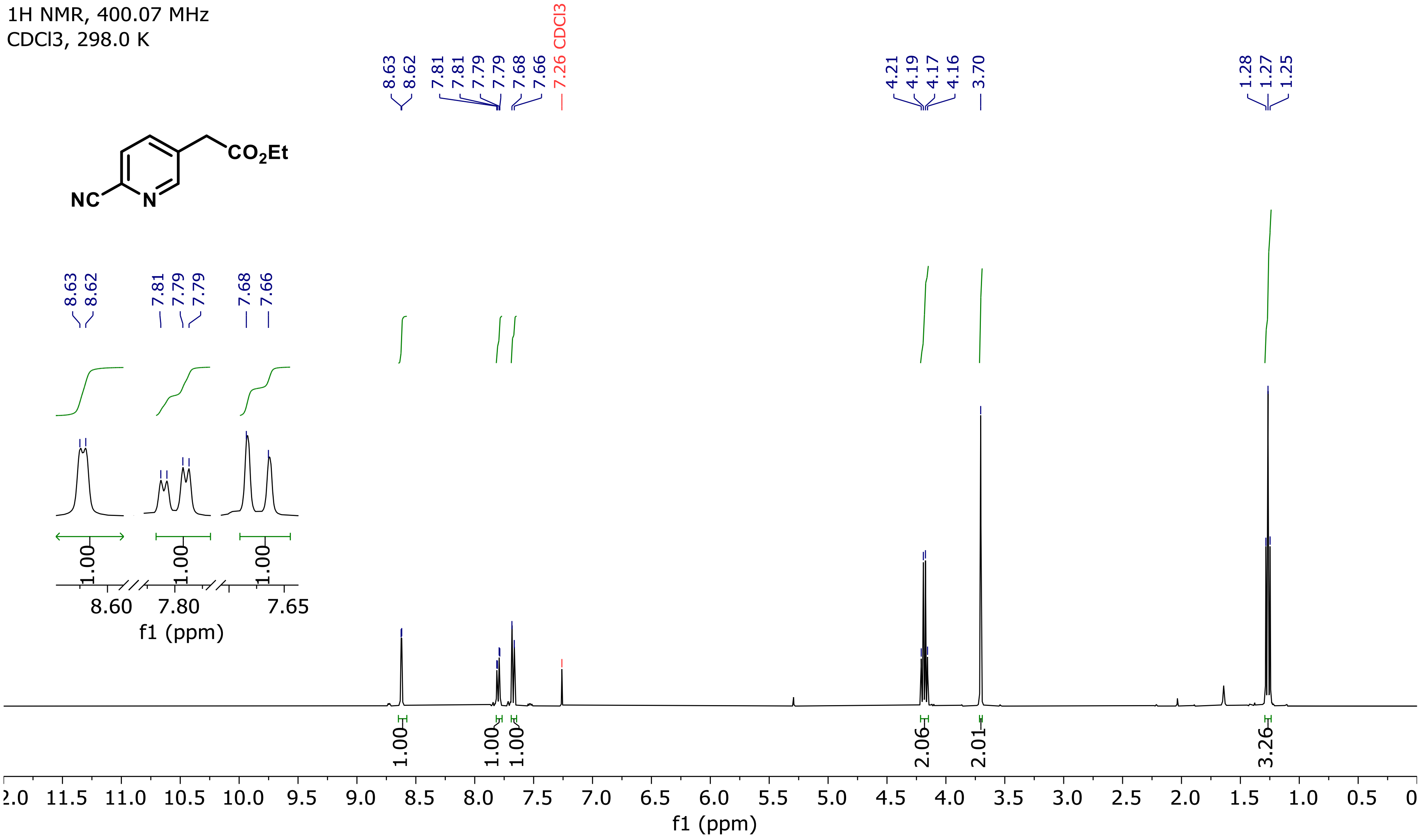

$^{13}$**C NMR of ethyl 2-(6-cyanopyridin-3-yl)acetate (26)**

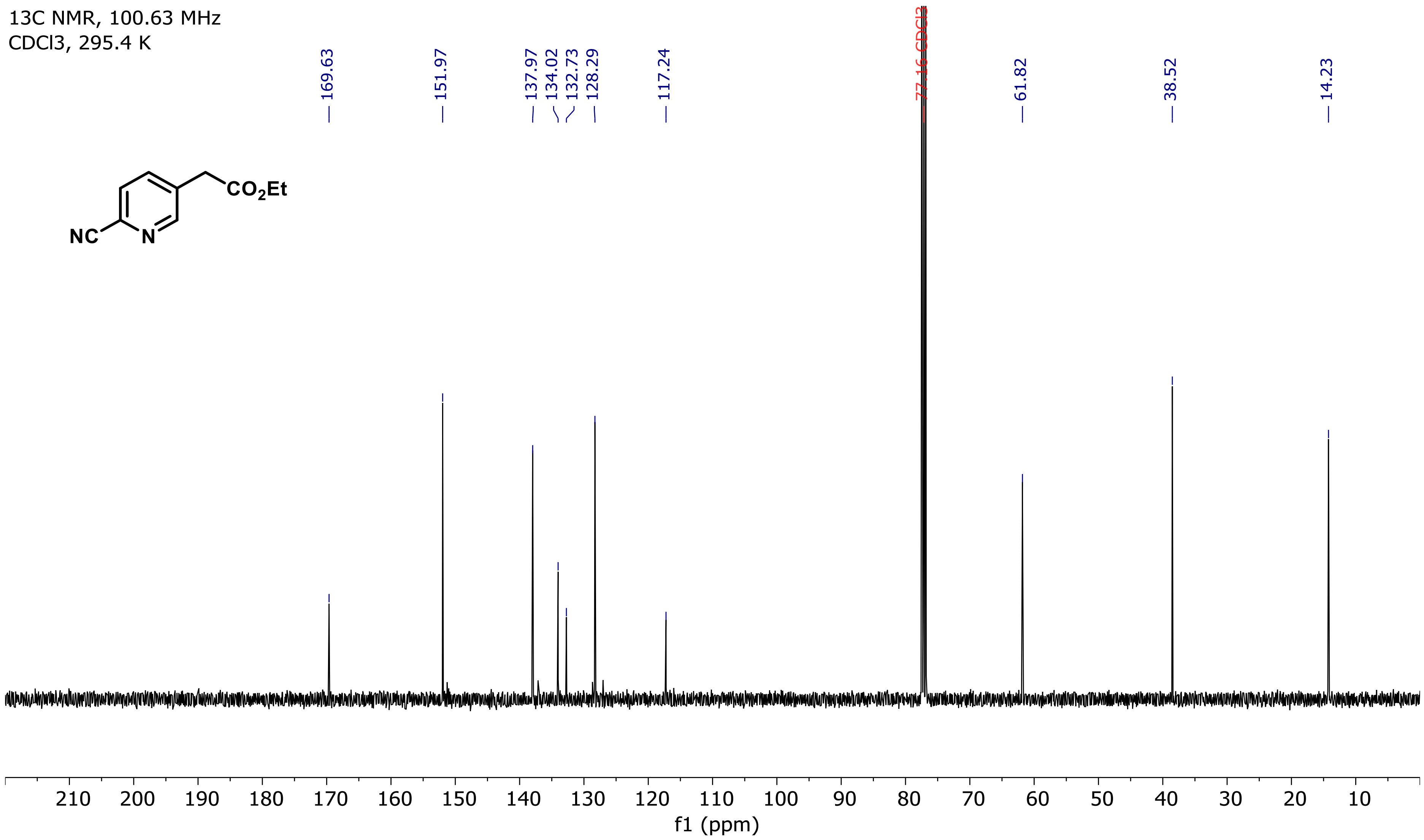

**$^{1}$H NMR of ethyl 2-(6-nitropyridin-3-yl)acetate (27)**

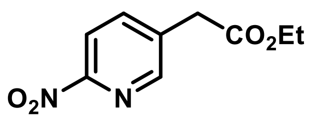


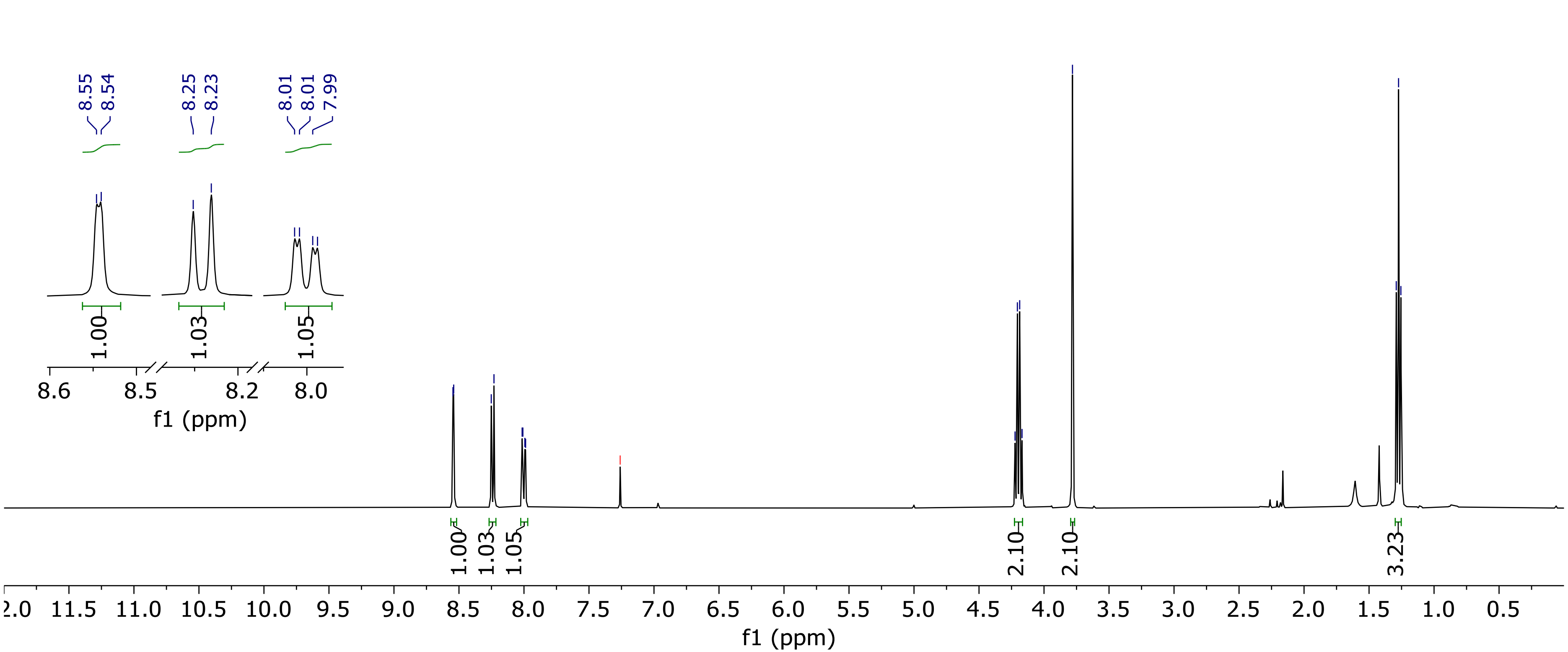

**$^{13}$C NMR of ethyl 2-(6-nitropyridin-3-yl)acetate (27)**

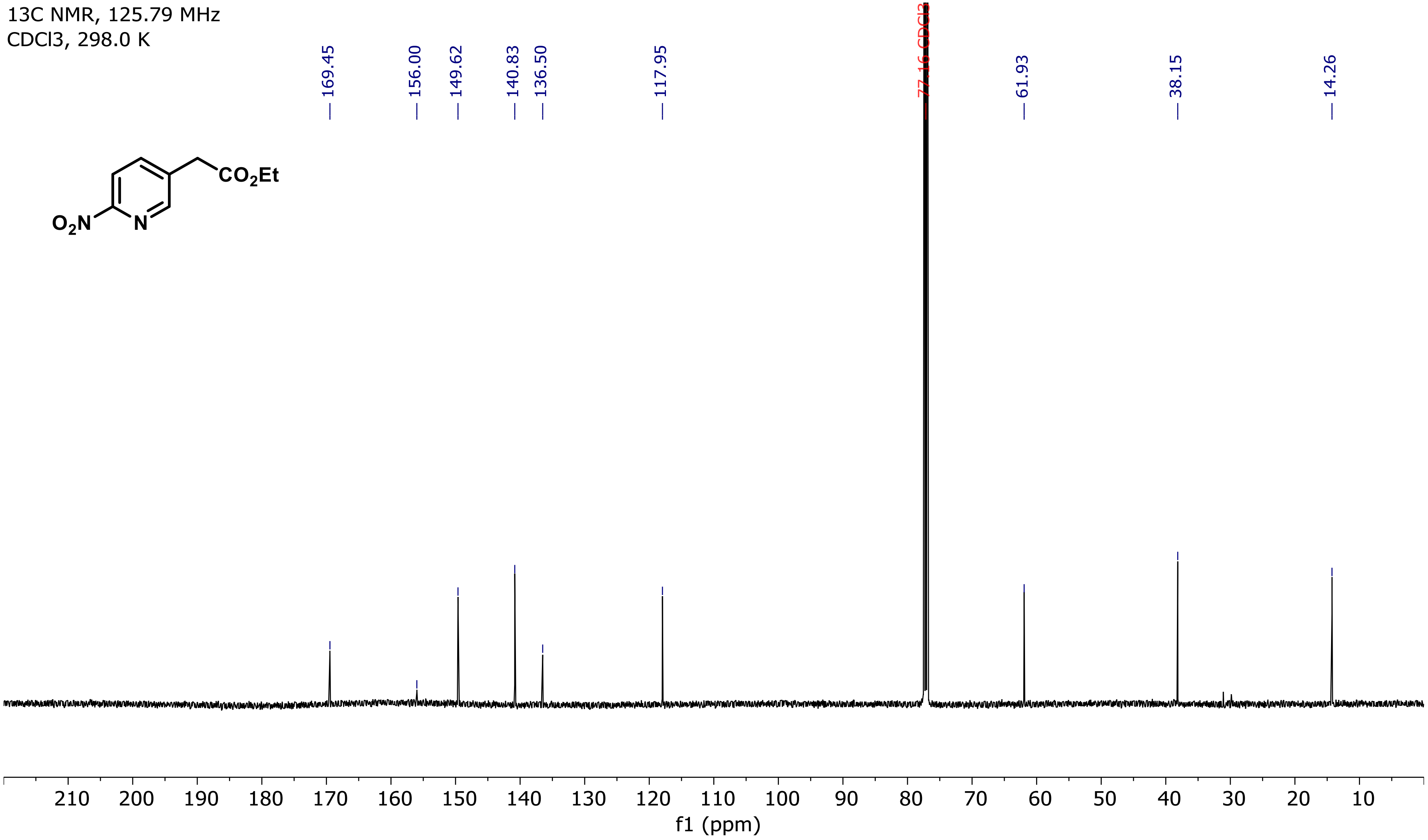

**$^{1}$H NMR of ethyl 2-(6'-methyl-3-(4-(methylsulfonyl)phenyl)-[2,3'-bipyridin]-5-yl)acetate (28)**

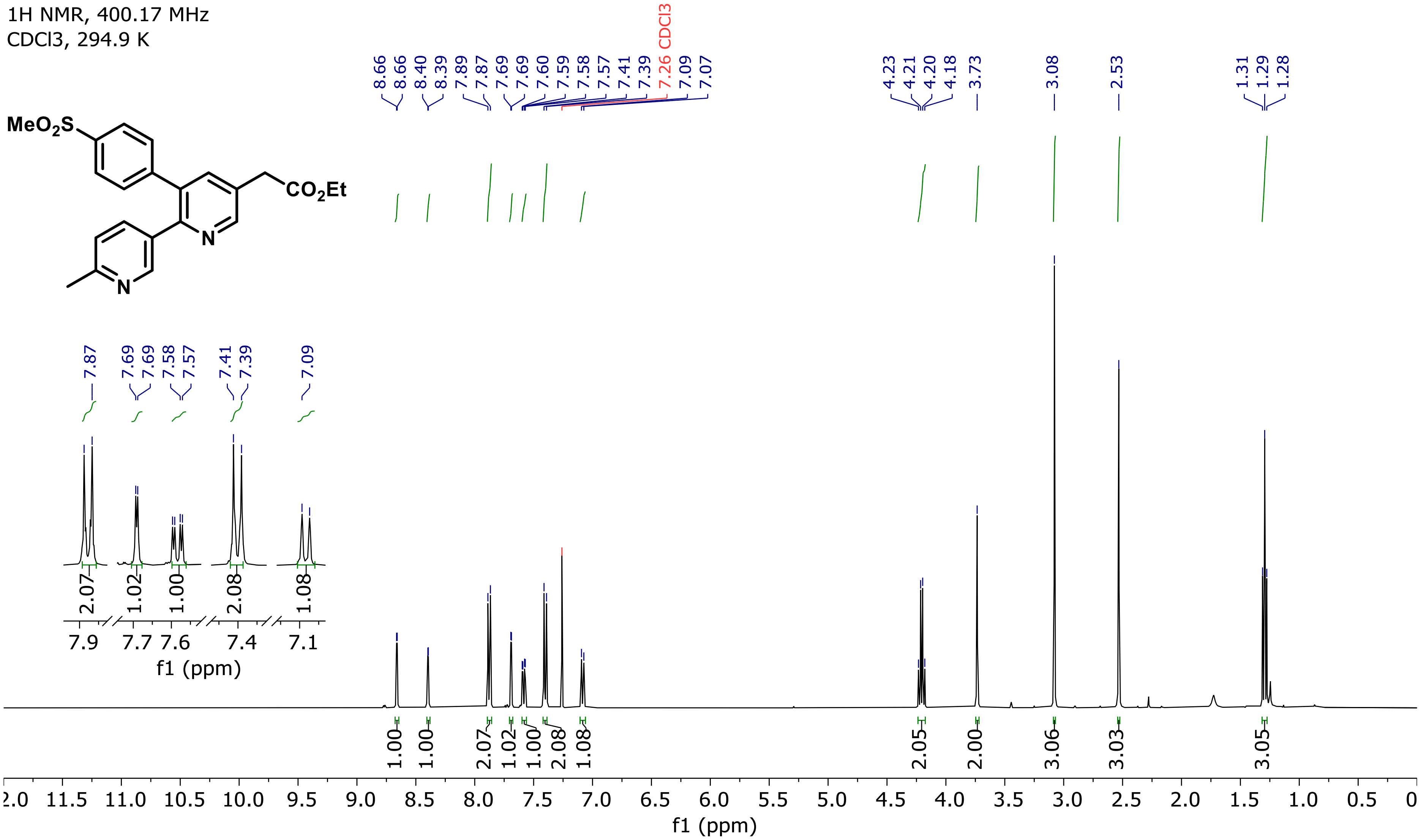

**$^{13}$C NMR of ethyl 2-(6'-methyl-3-(4-(methylsulfonyl)phenyl)-[2,3'-bipyridin]-5-yl)acetate (28)**

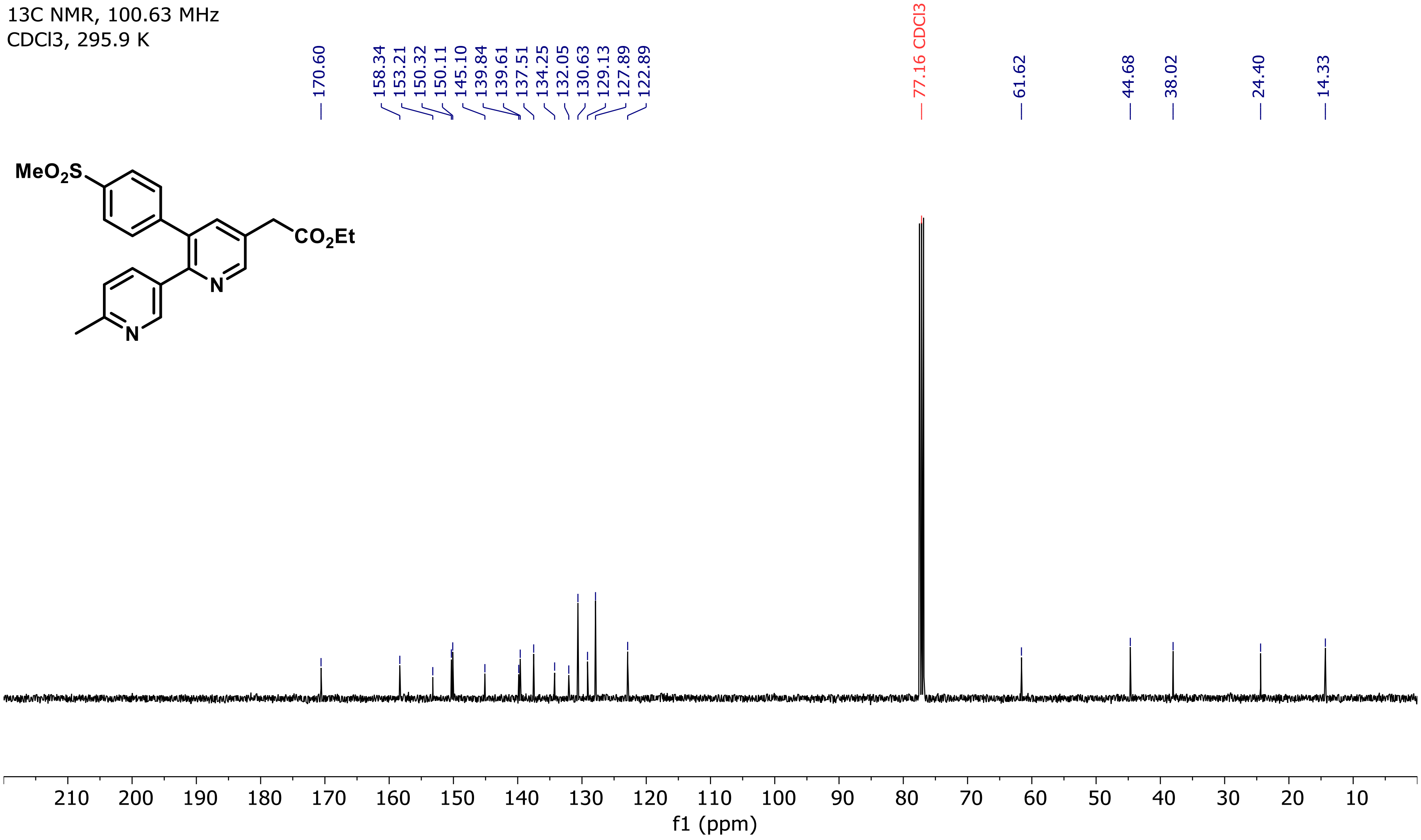

**$^{1}$H NMR of methyl 5-(2-ethoxy-2-oxoethyl)nicotinate (29)**

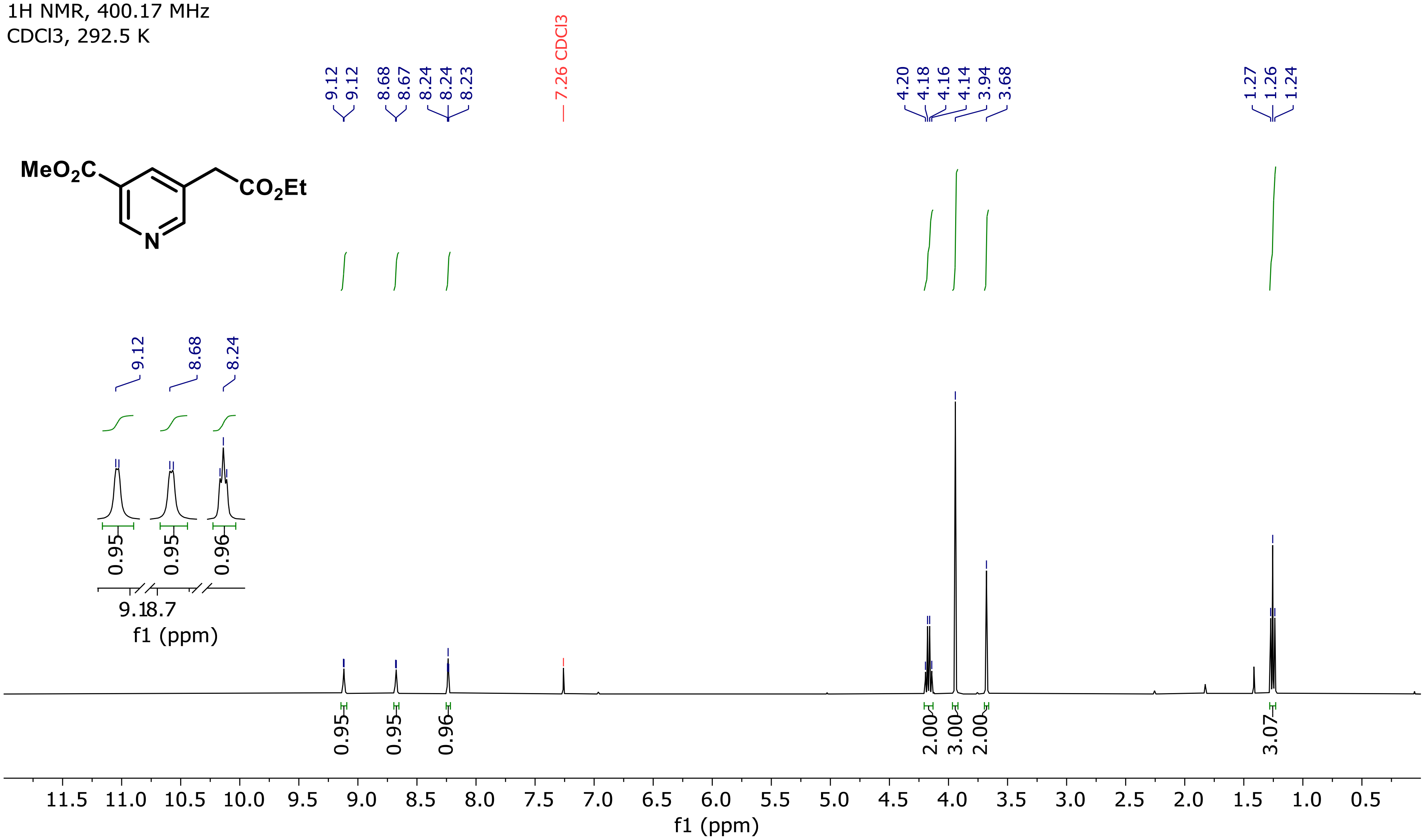

**$^{13}$C NMR of methyl 5-(2-ethoxy-2-oxoethyl)nicotinate (29)**

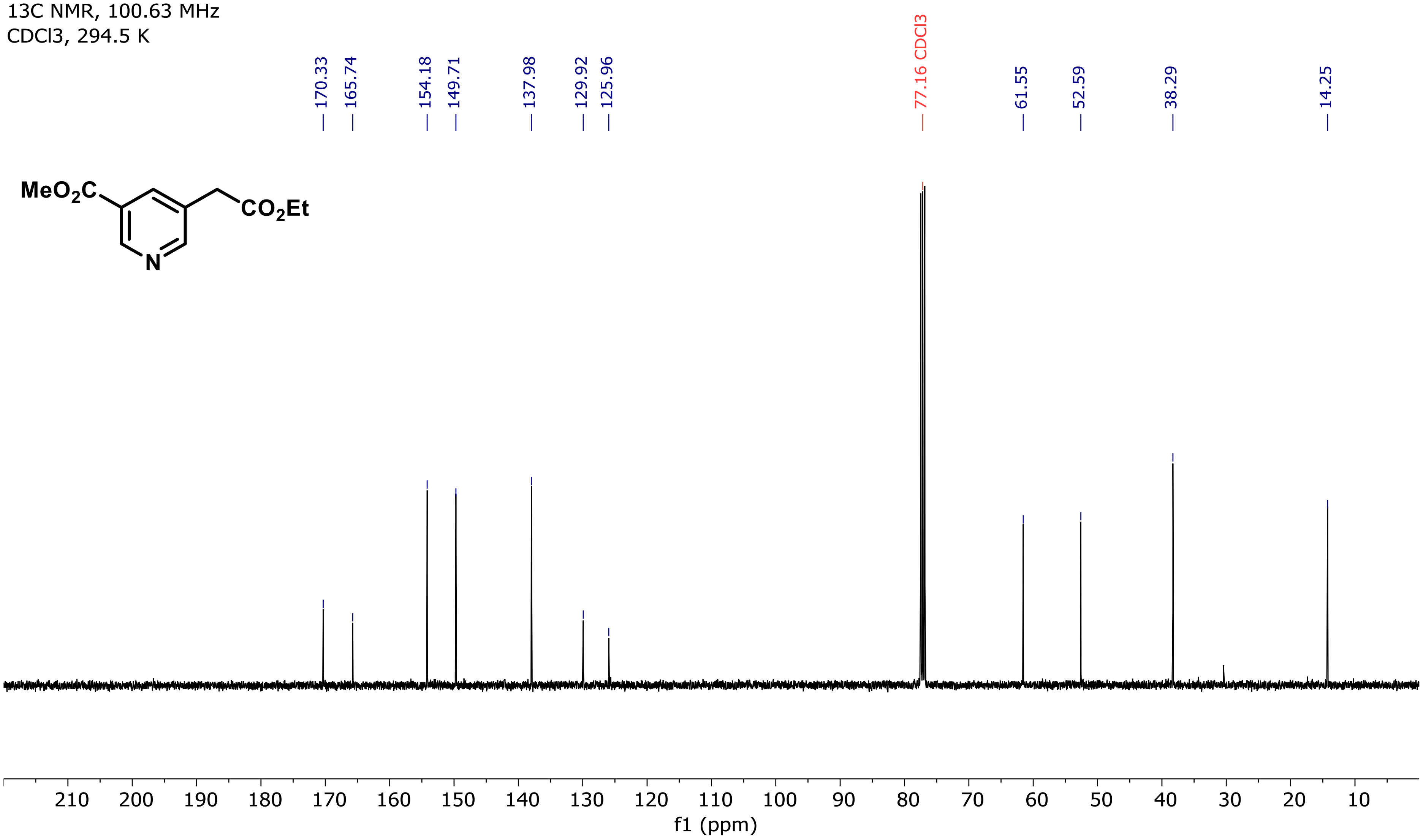

**$^1$H NMR of ethyl 2-(5-cyanopyridin-2-yl)acetate (30)**

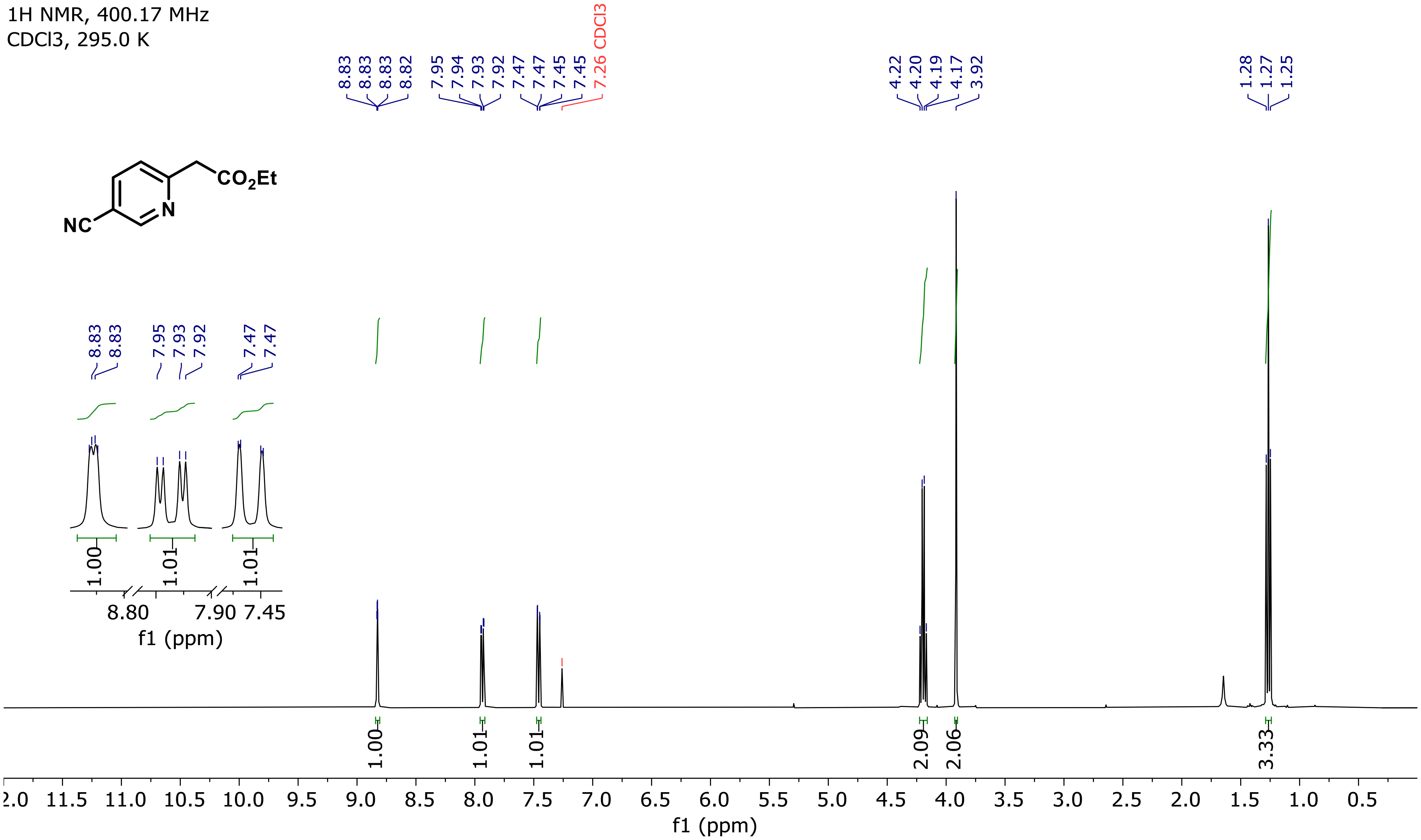

**$^{13}$C NMR of ethyl 2-(5-cyanopyridin-2-yl)acetate (30)**

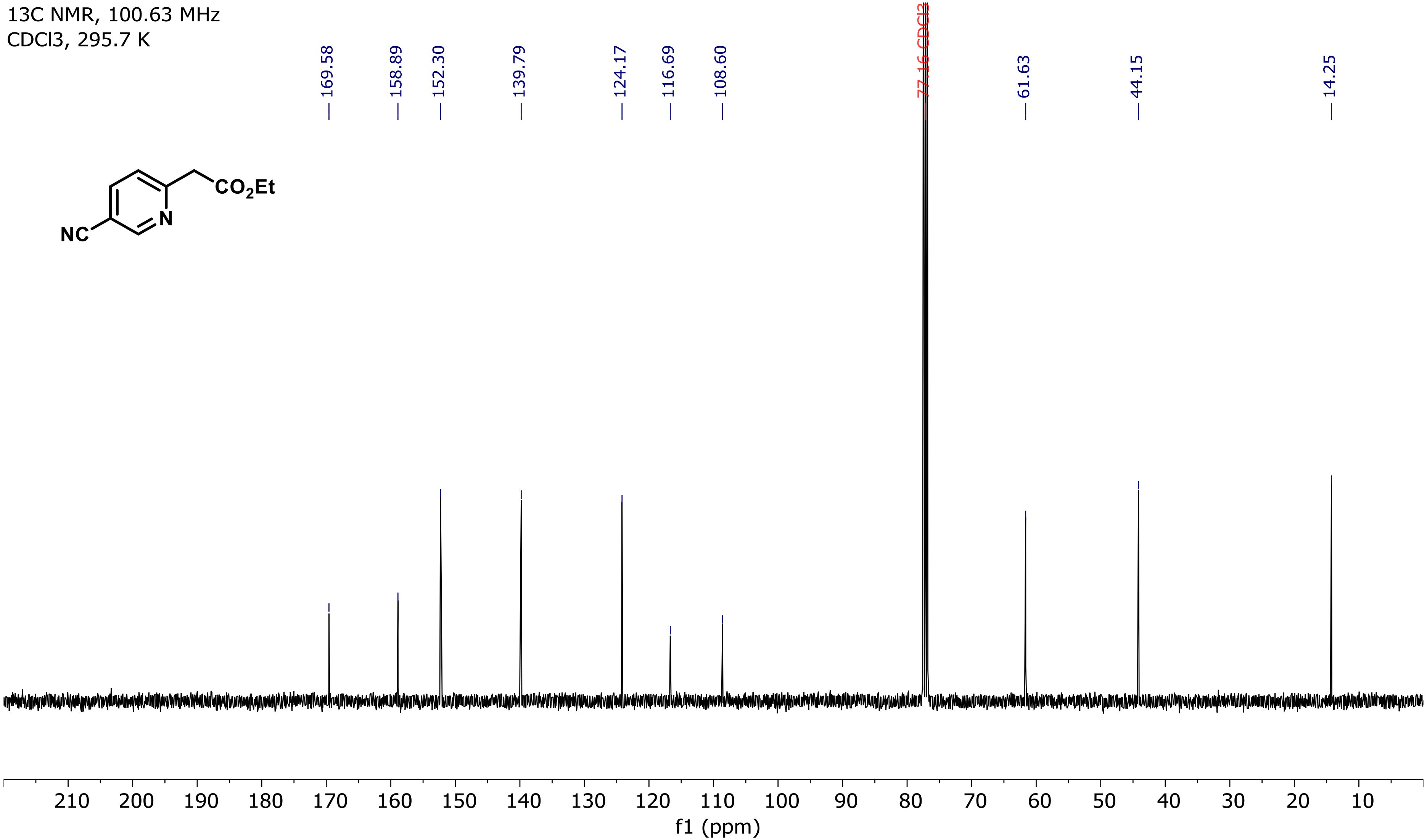

**$^{1}$H NMR of ethyl 2-(5-(morpholine-4-carbonyl)pyridin-2-yl)acetate (31)**

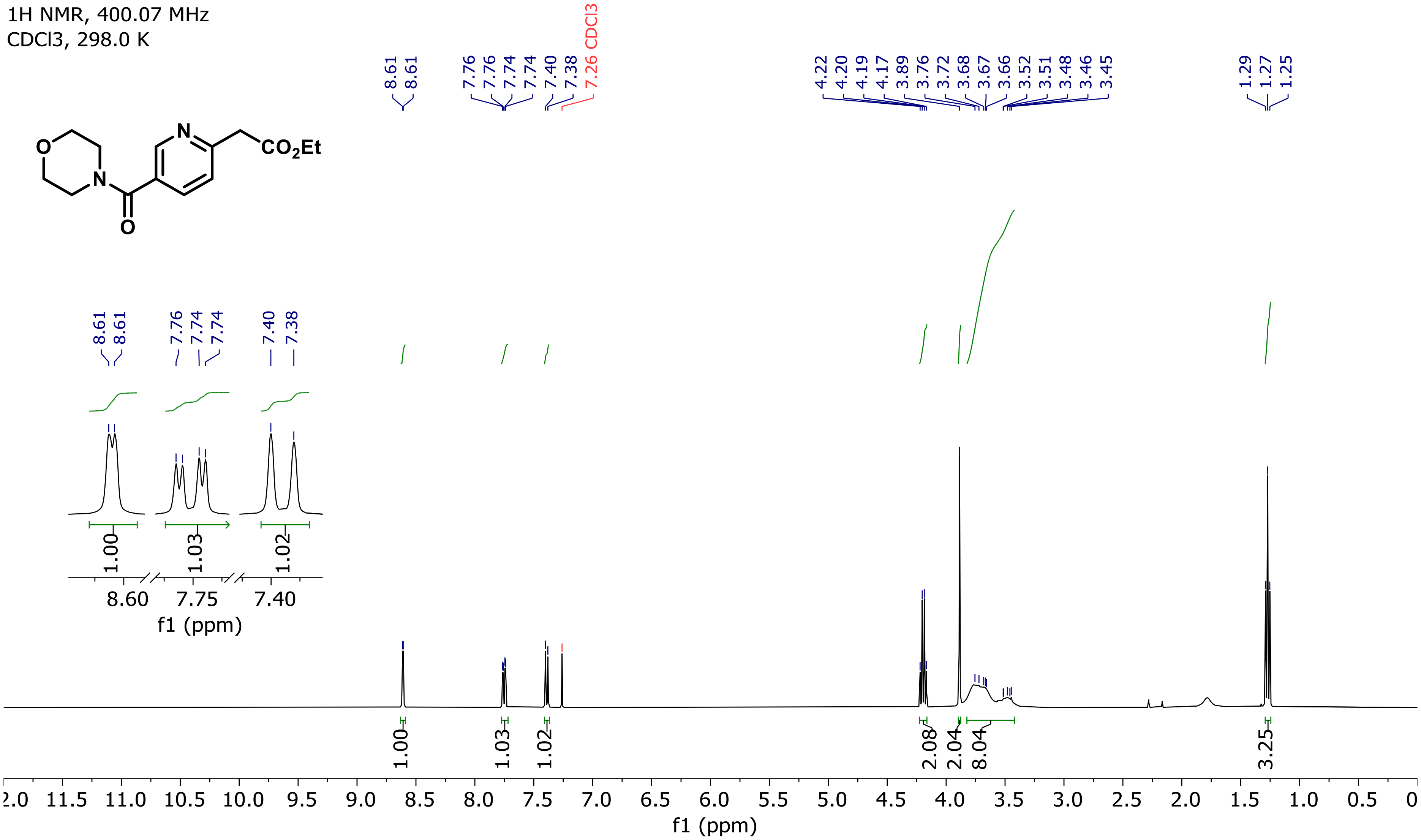

**$^{13}$C NMR of ethyl 2-(5-(morpholine-4-carbonyl)pyridin-2-yl)acetate (31)**

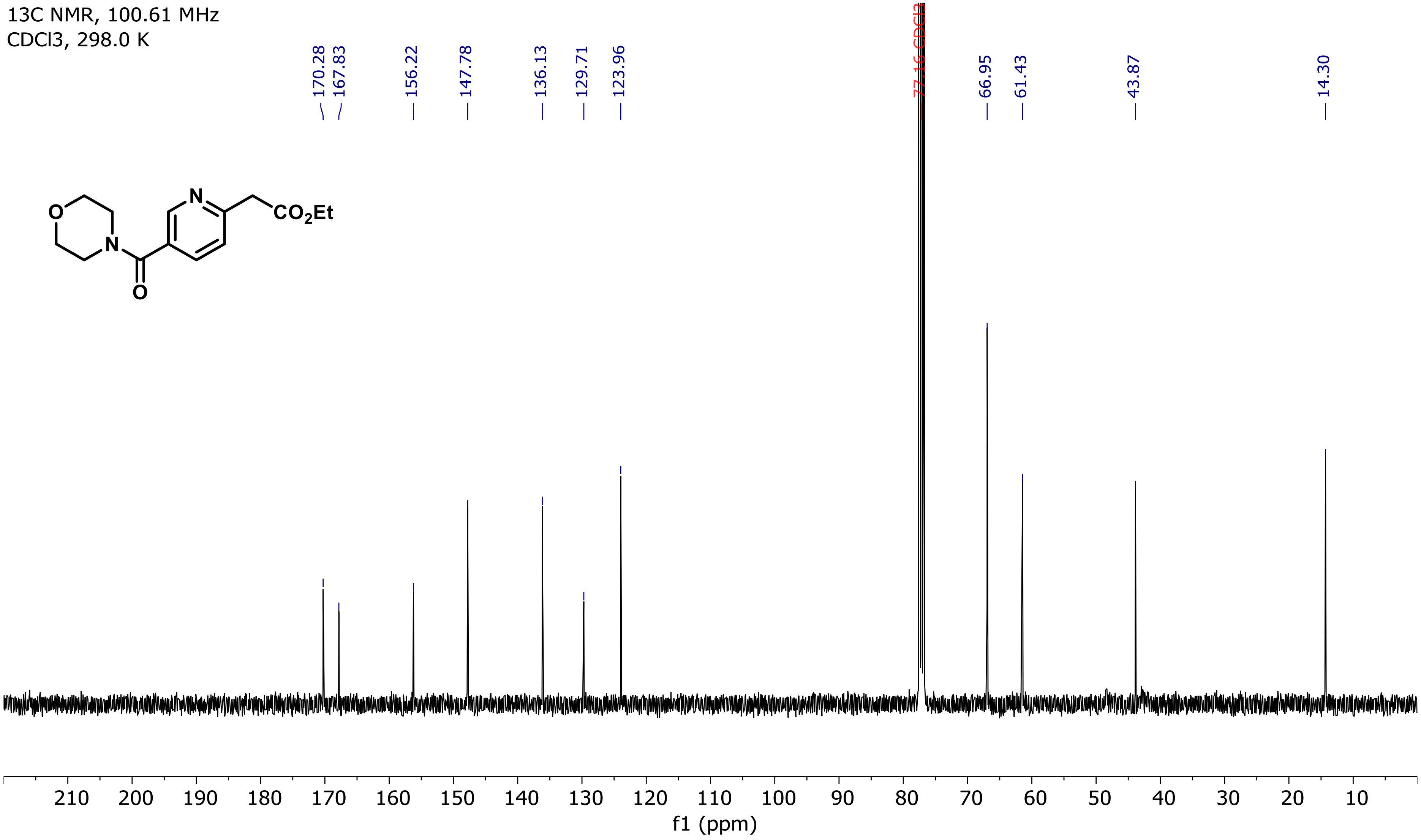

**$^{1}$H NMR of ethyl 2-(5-(trifluoromethyl)pyridin-2-yl)acetate (32)**

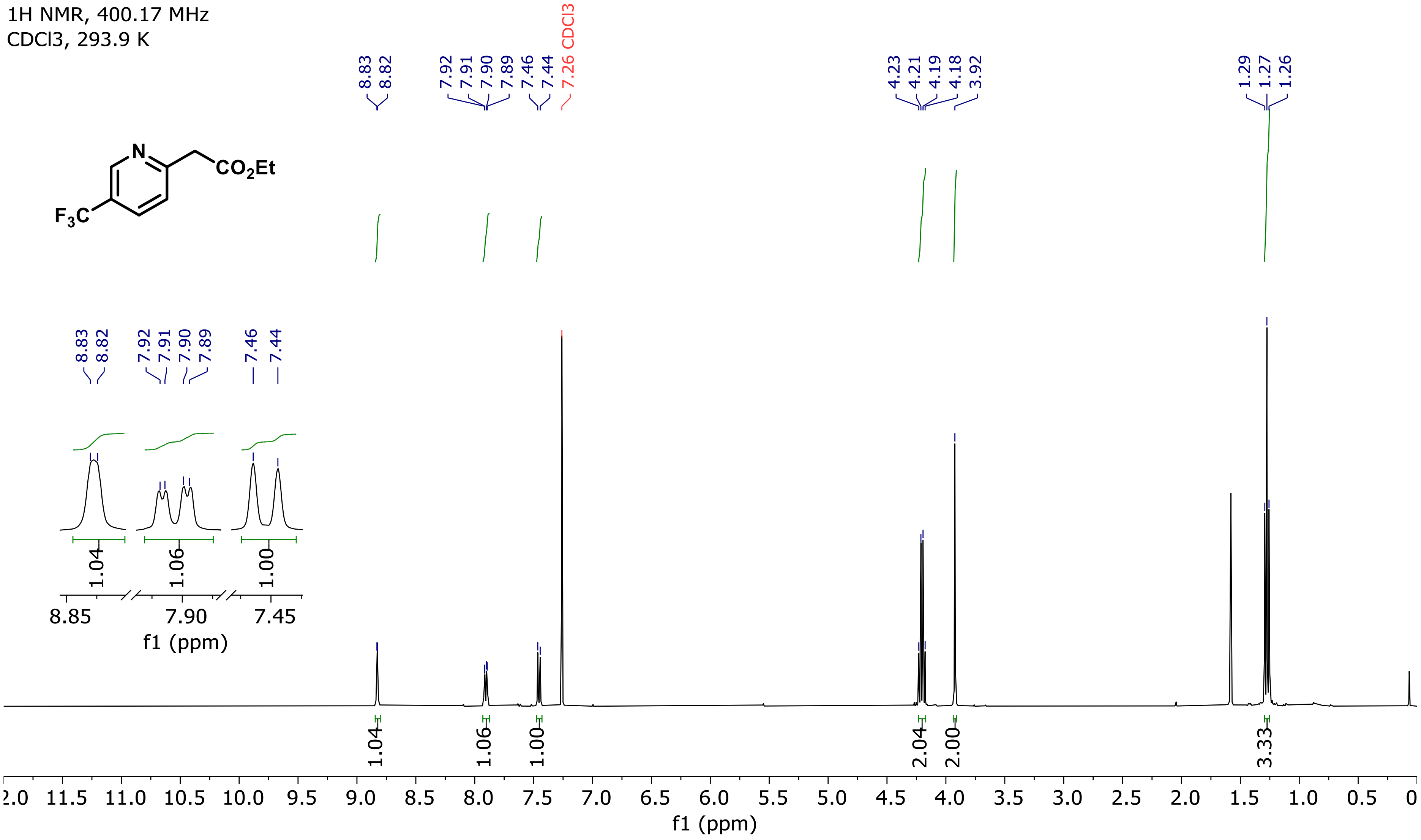

**$^{13}$C NMR of ethyl 2-(5-(trifluoromethyl)pyridin-2-yl)acetate (32)**

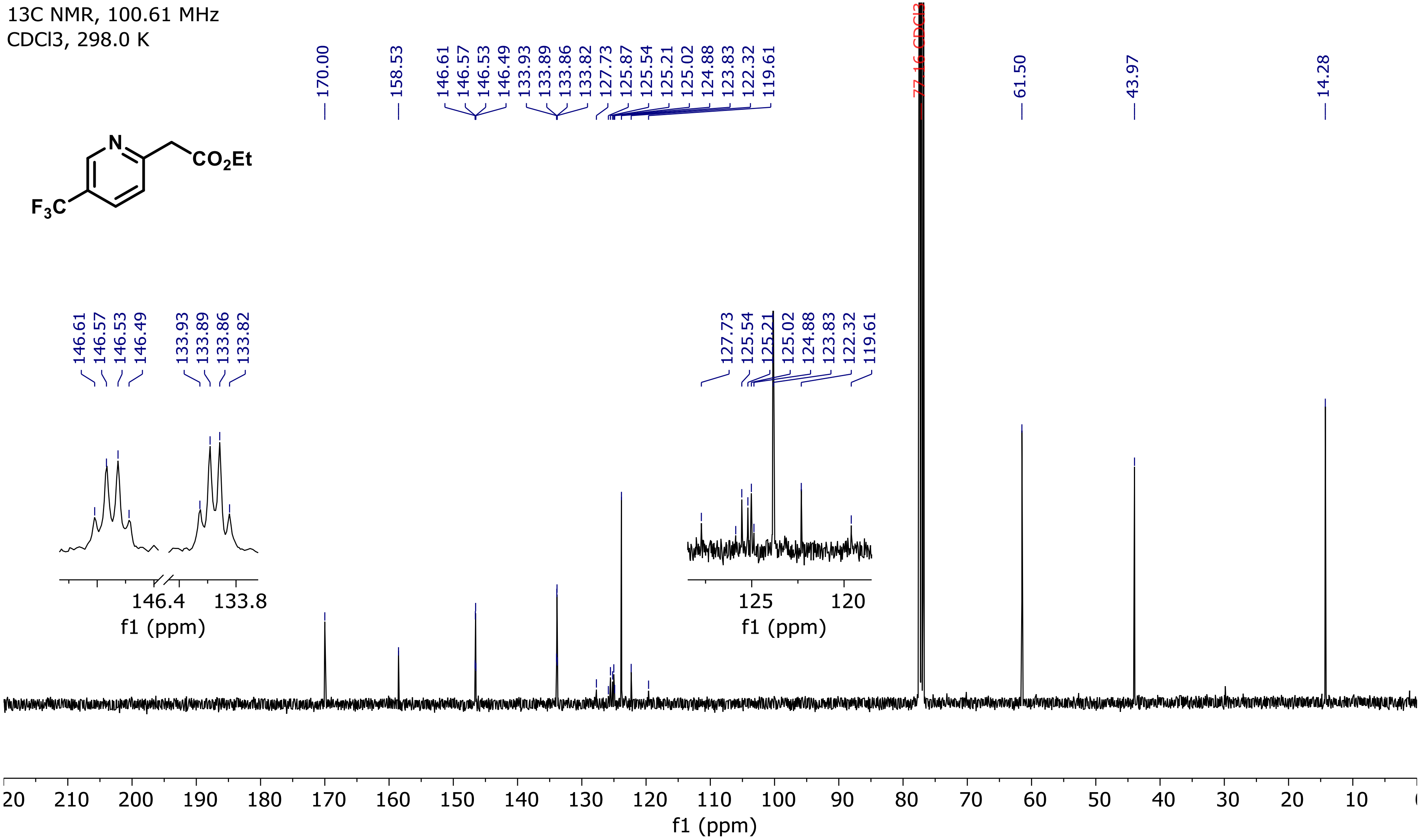

**$^{19}$F NMR of ethyl 2-(5-(trifluoromethyl)pyridin-2-yl)acetate (32)**

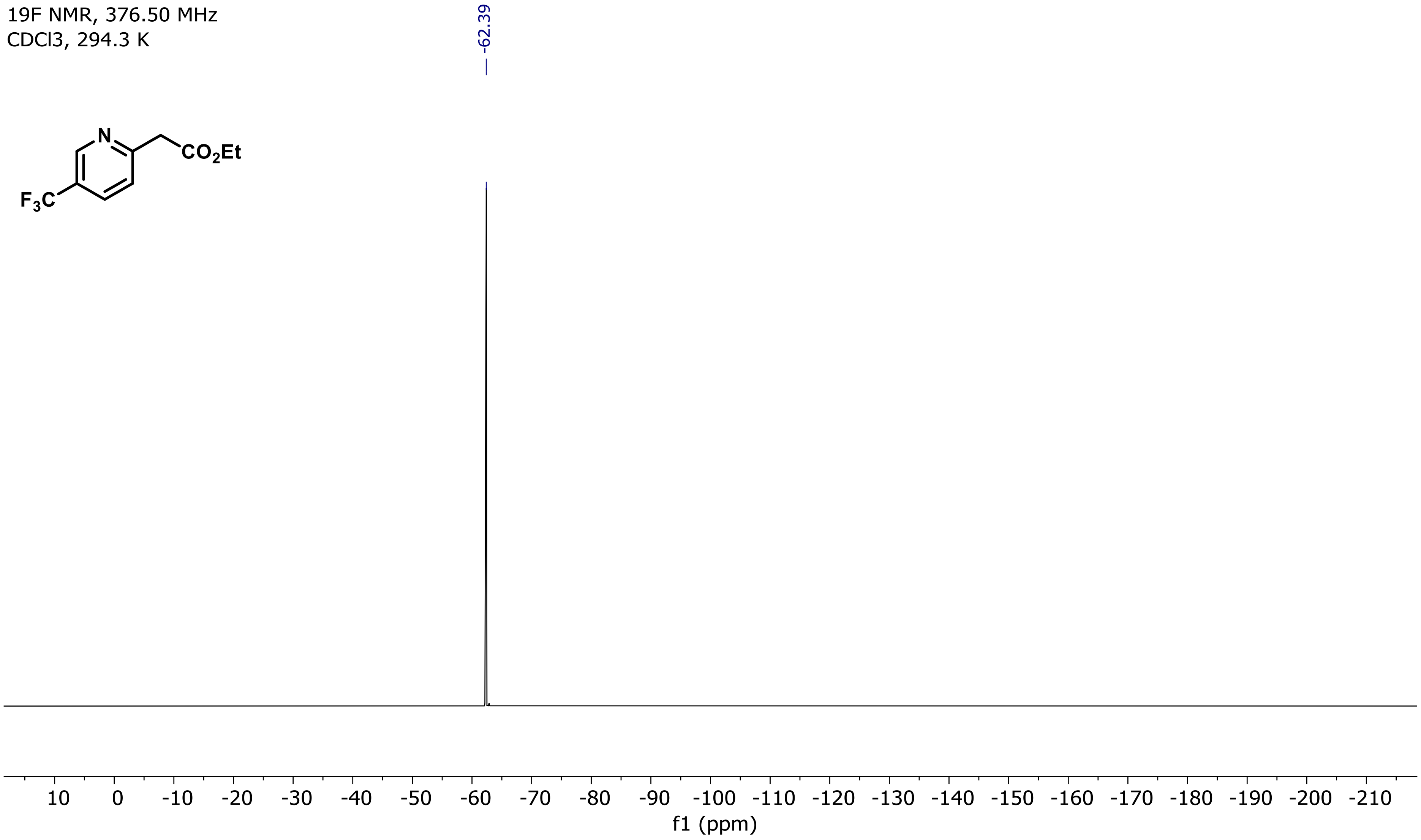

**$^{1}$H NMR of ethyl 2-(2-fluoropyridin-4-yl)acetate (33)**

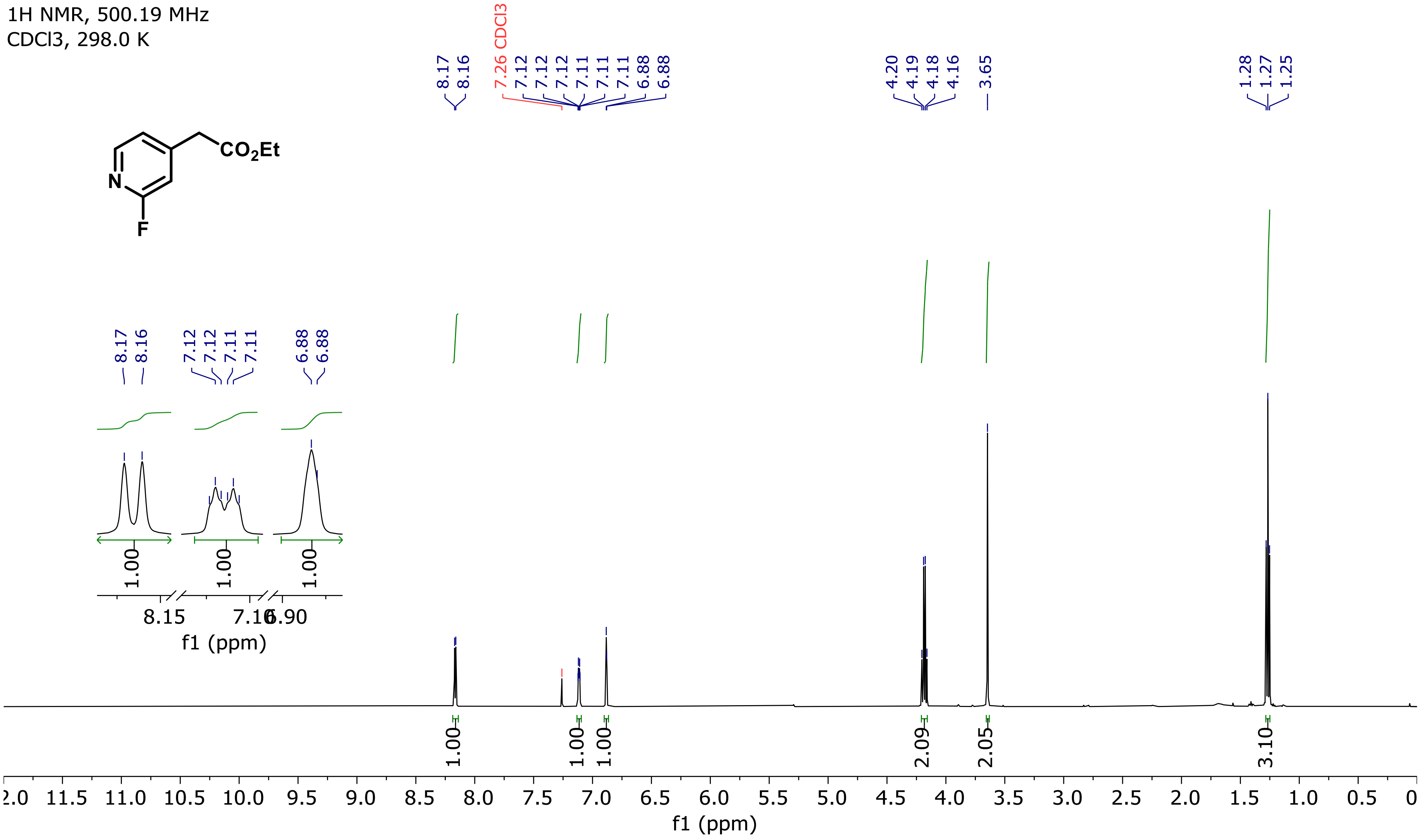

**$^{13}$C NMR of ethyl 2-(2-fluoropyridin-4-yl)acetate (33)**

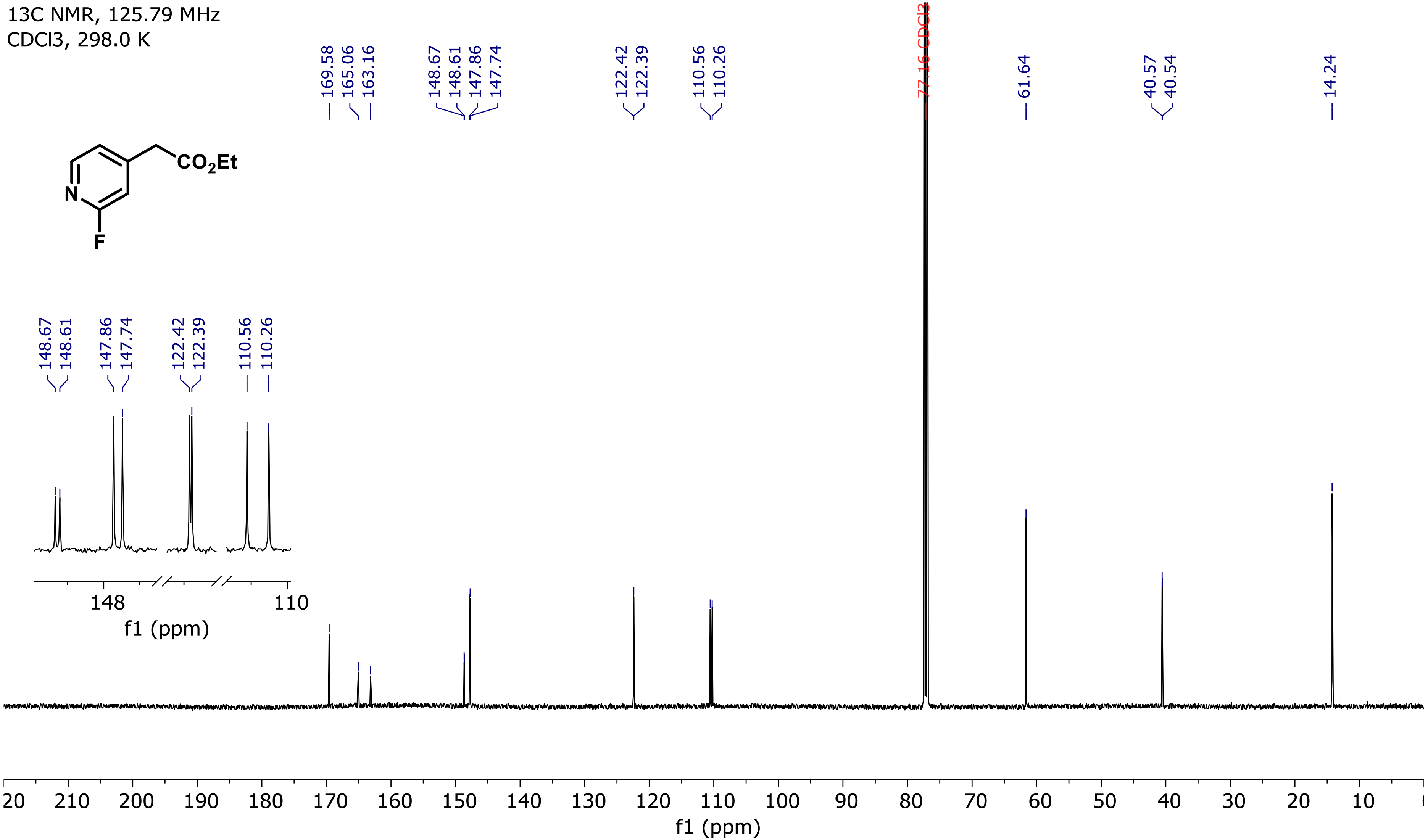

**$^{19}$F NMR of ethyl 2-(2-fluoropyridin-4-yl)acetate (33)**

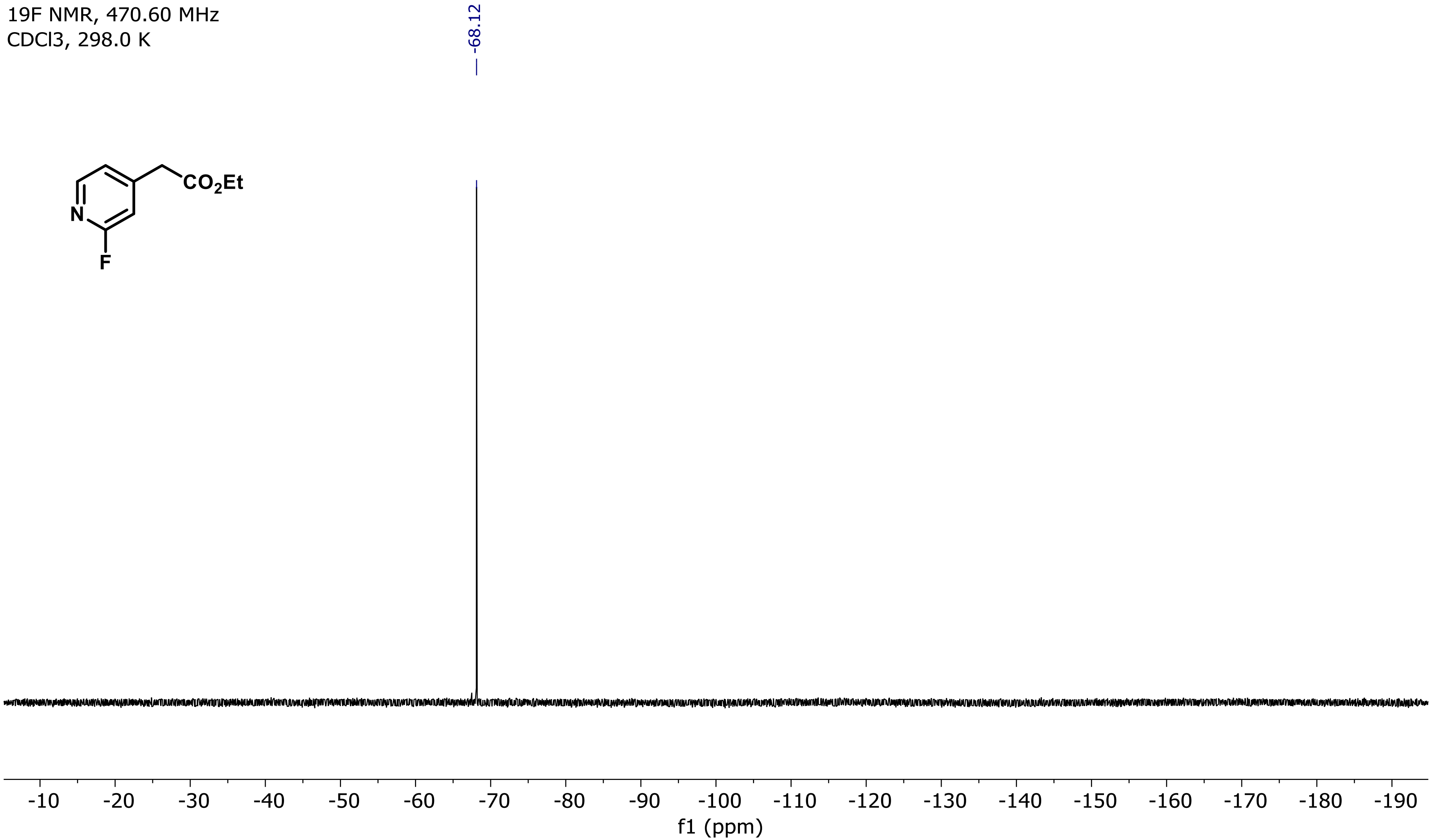

**$^{1}$H NMR of methyl 5-(2-ethoxy-2-oxoethyl)pyrazine-2-carboxylate (34)**

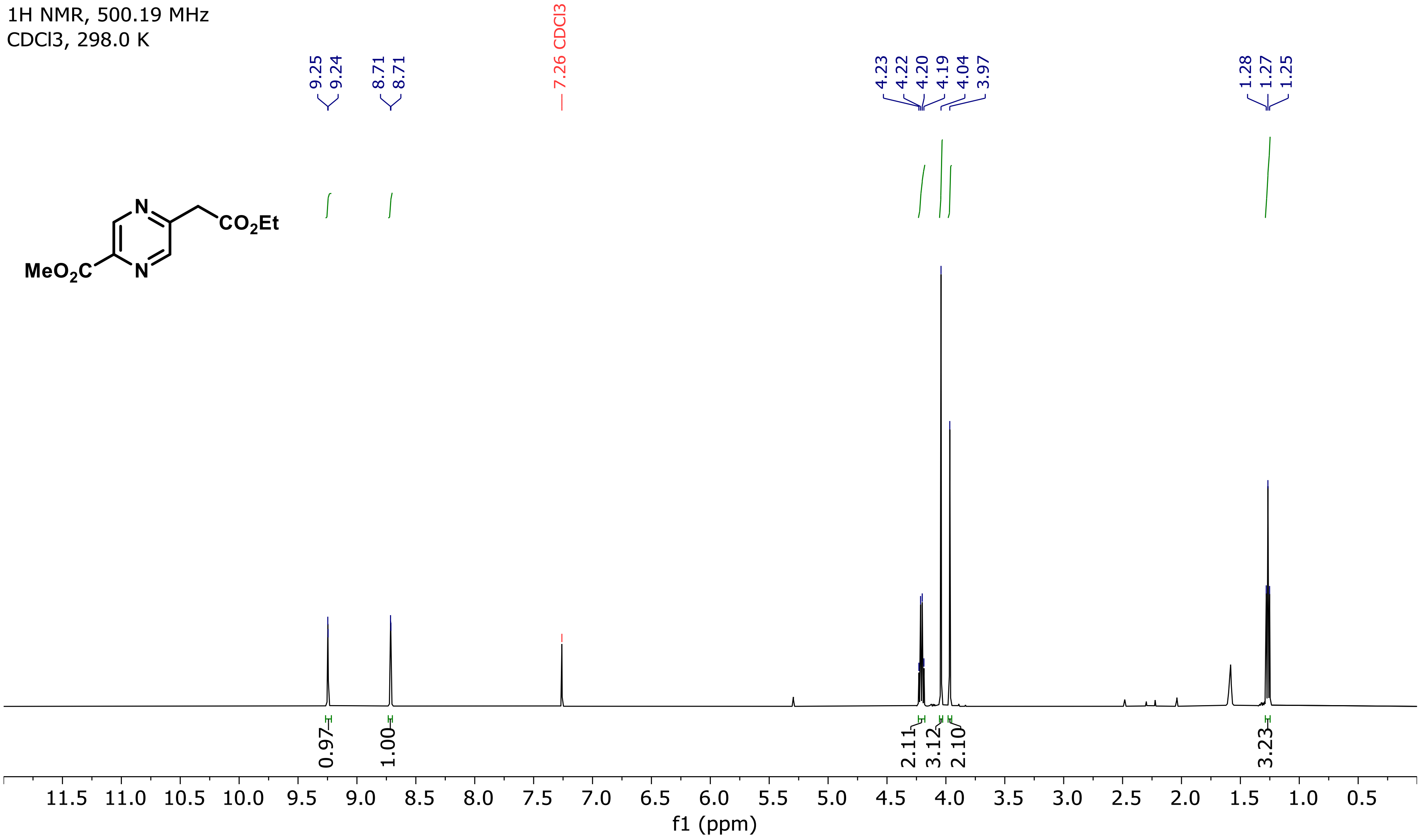

**$^{13}$C NMR of methyl 5-(2-ethoxy-2-oxoethyl)pyrazine-2-carboxylate (34)**

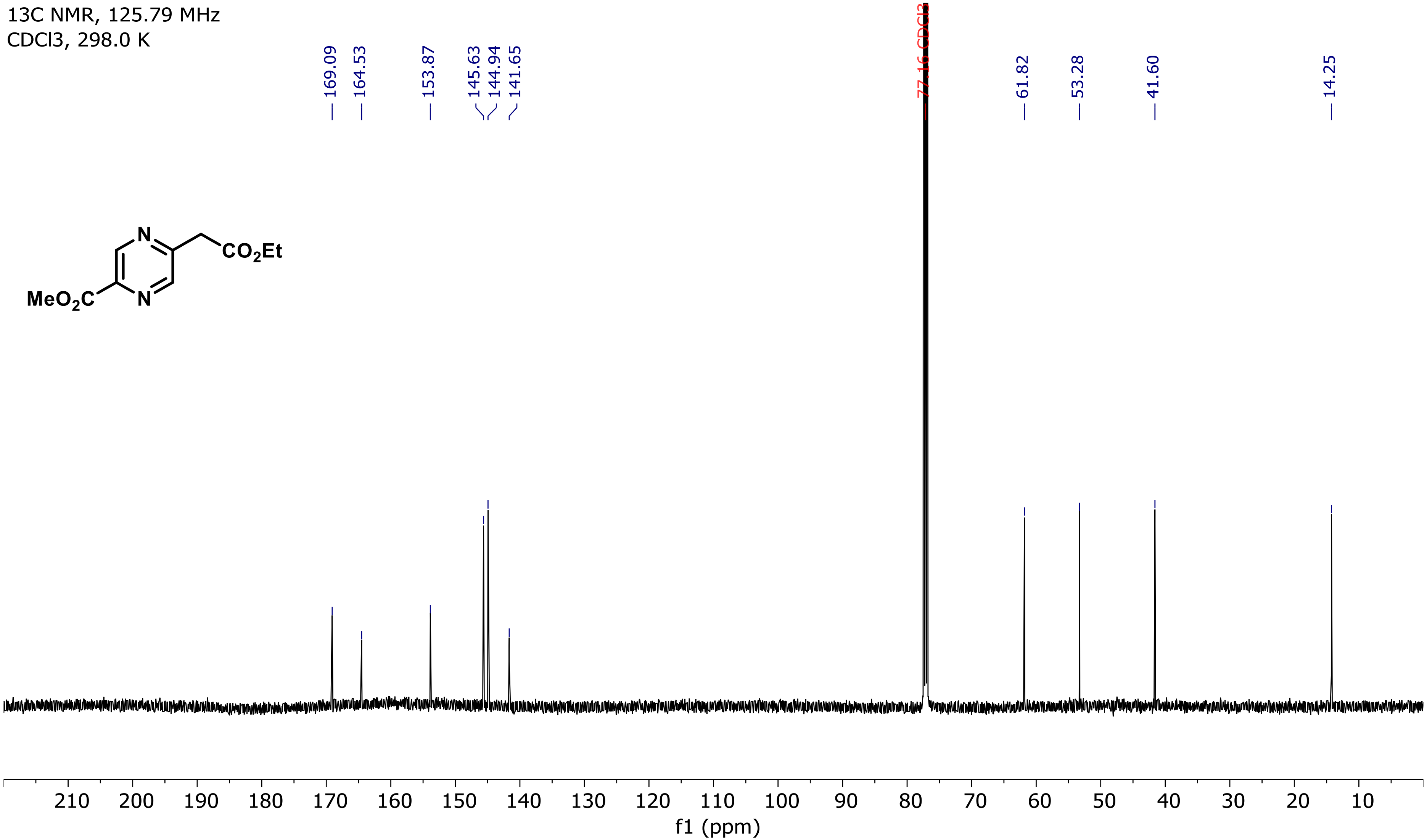

**$^{1}$H NMR of ethyl 2-(pyrimidin-5-yl)acetate (35)**

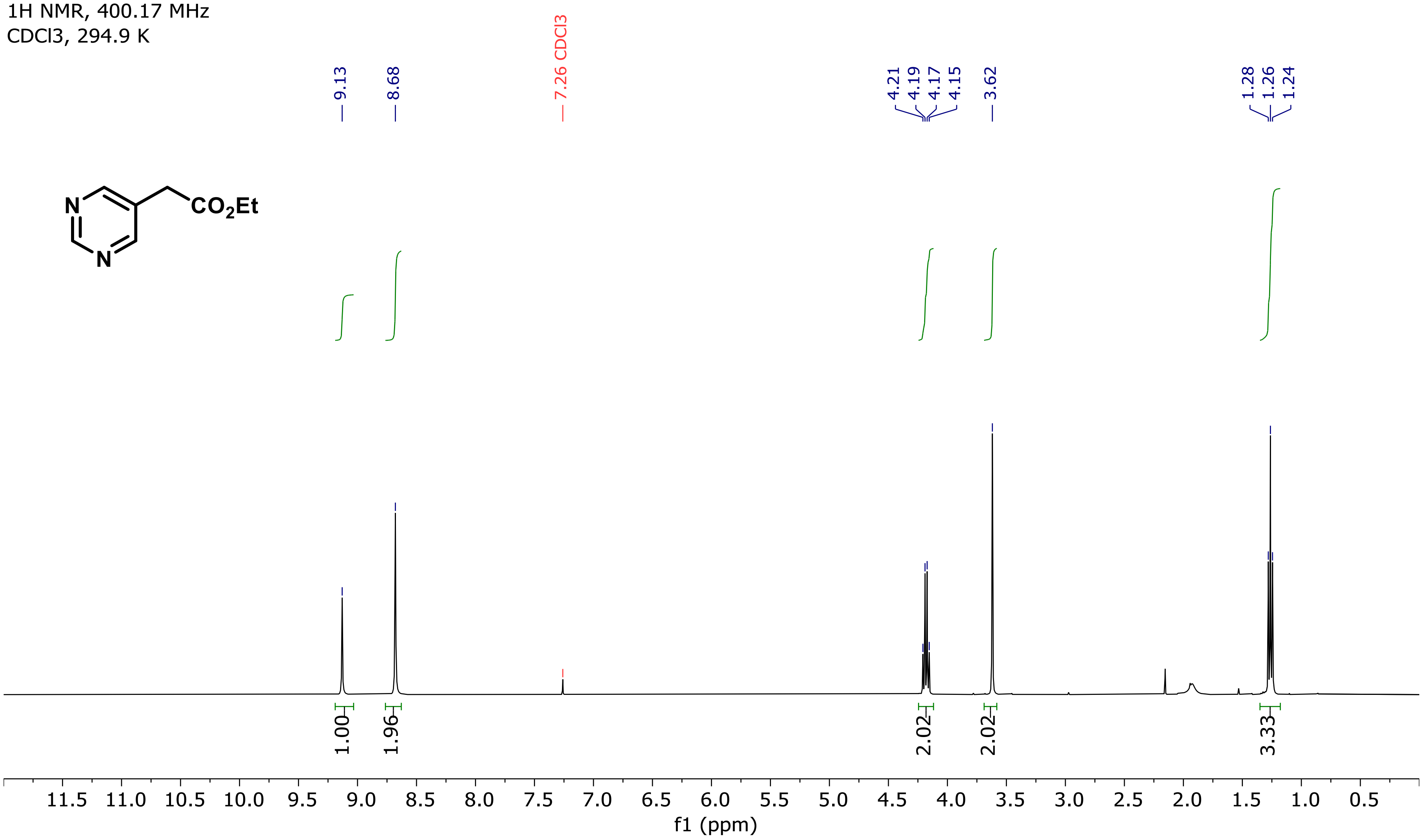

**$^{13}$C NMR of ethyl 2-(pyrimidin-5-yl)acetate (35)**

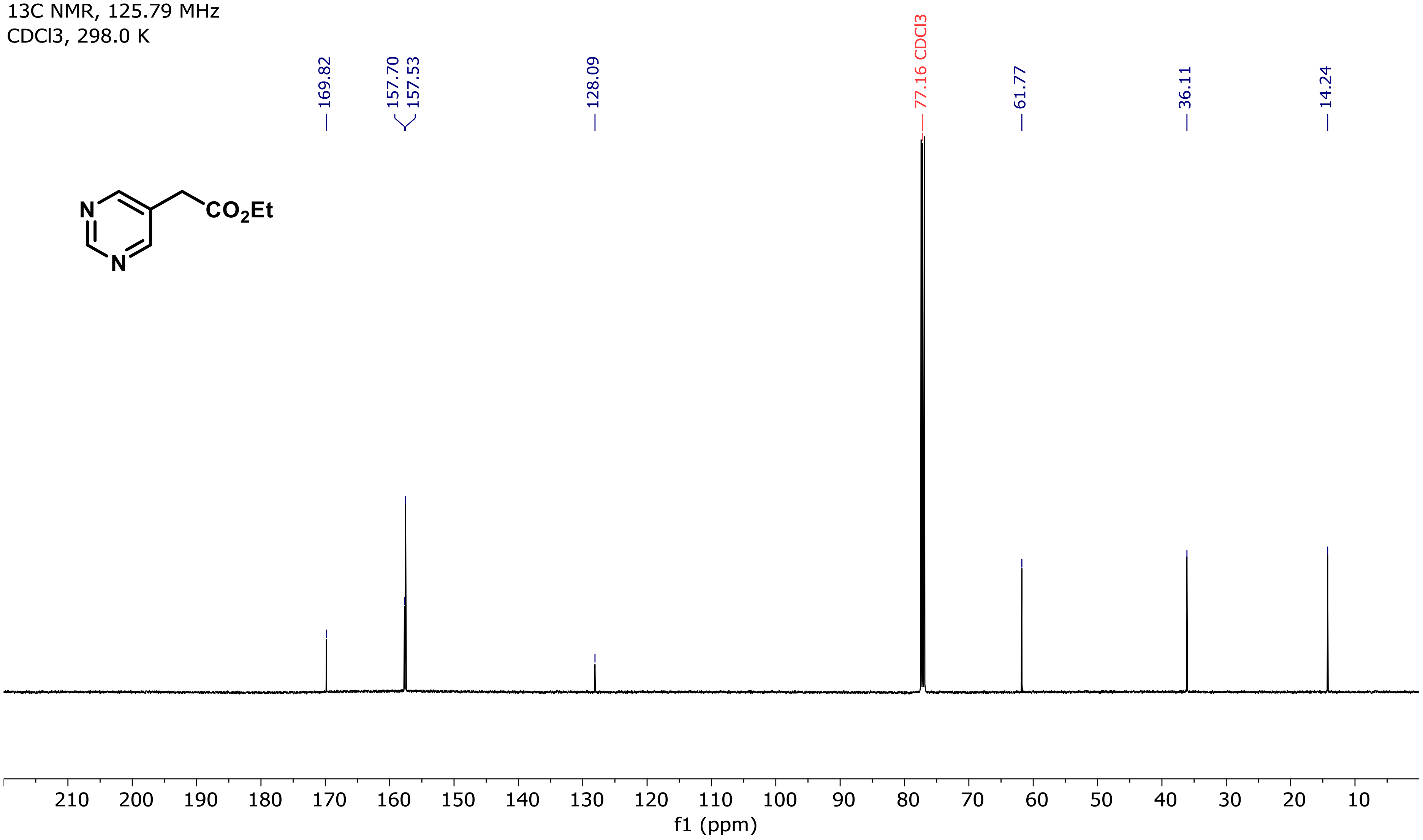

# $^{1}$H NMR of ethyl 2-(quinolin-4-yl)acetate (36)

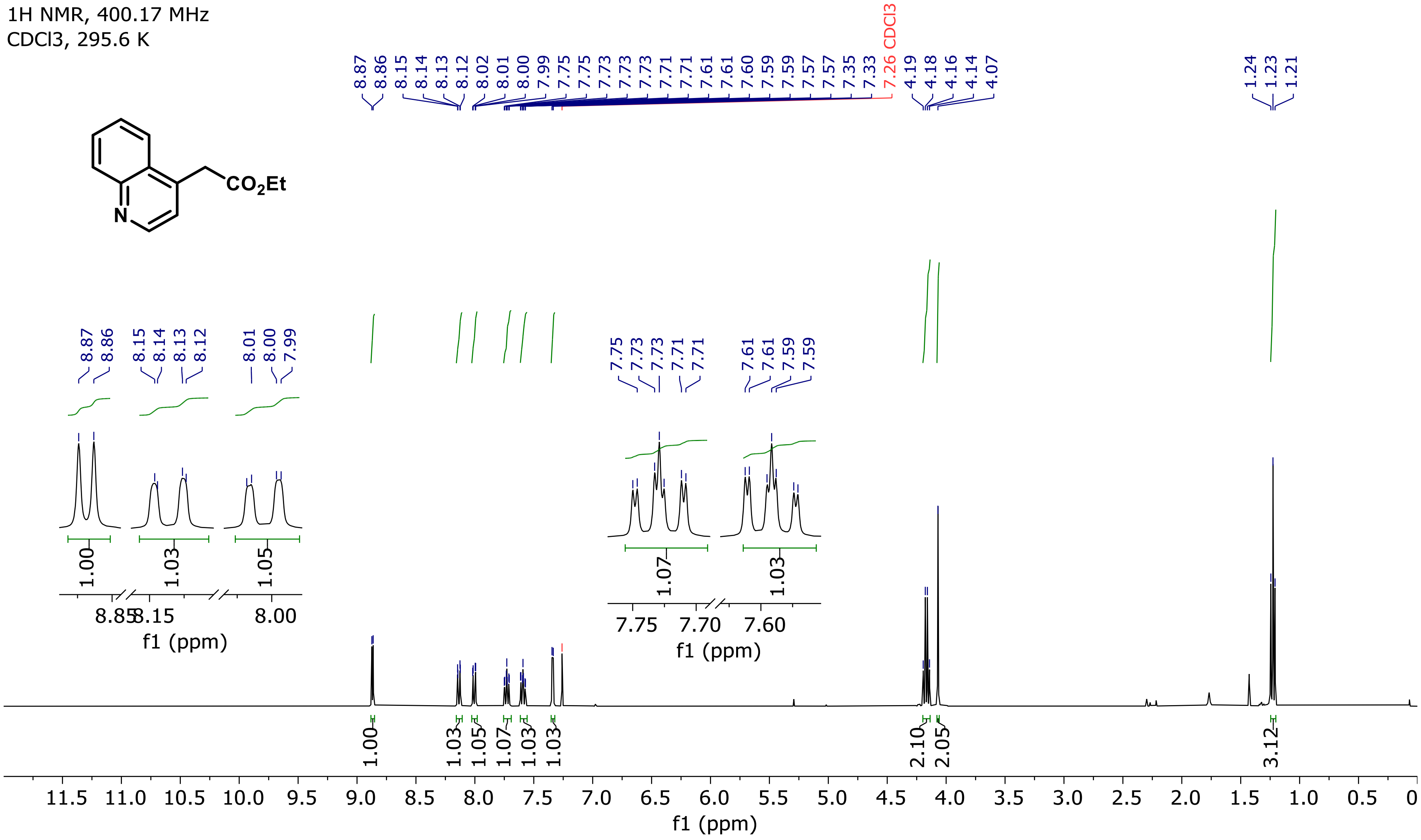

**$^{13}$C NMR of ethyl 2-(quinolin-4-yl)acetate (36)**

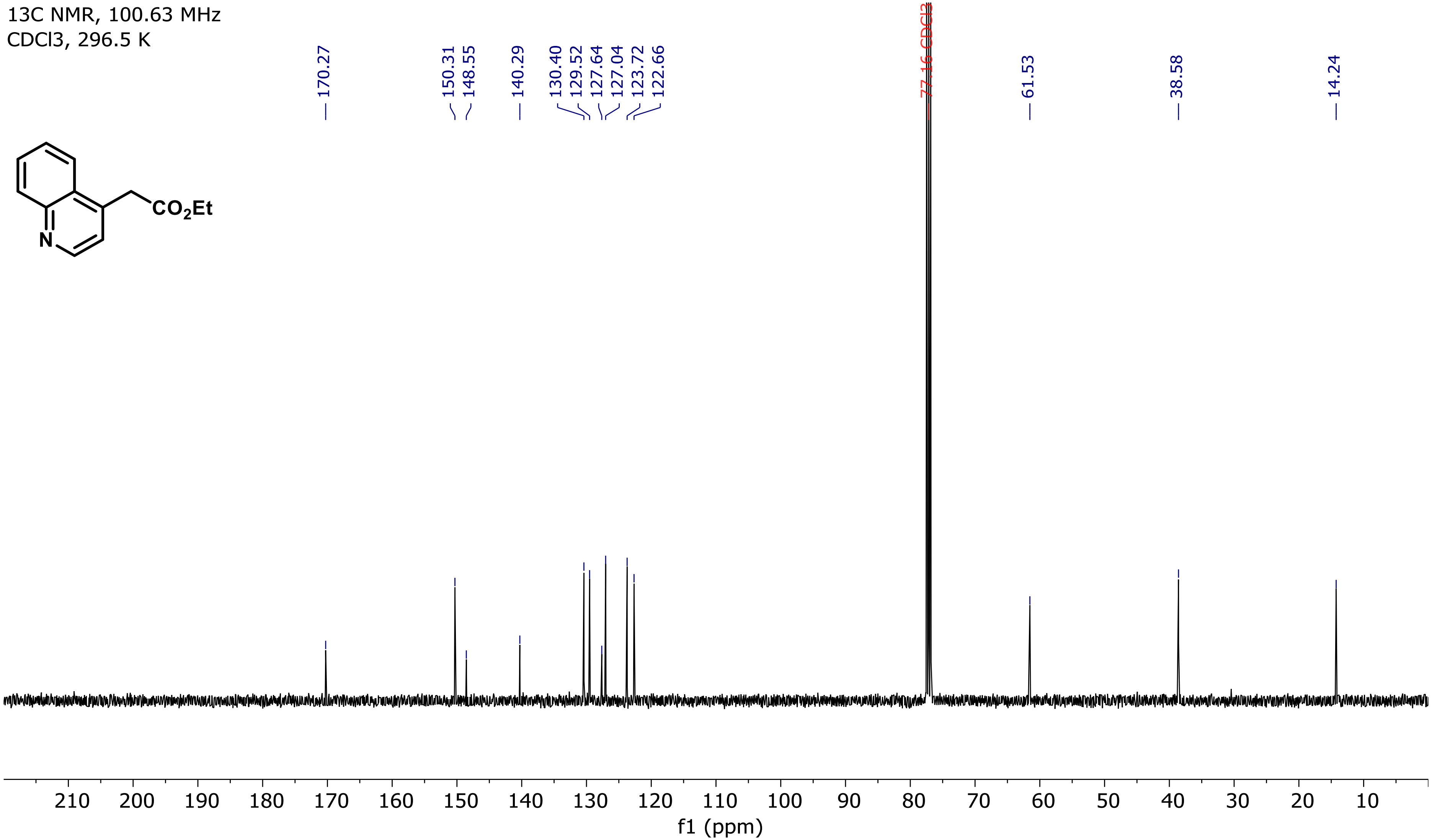

**$^1$H NMR of ethyl 2-(isoquinolin-4-yl)acetate (37)**

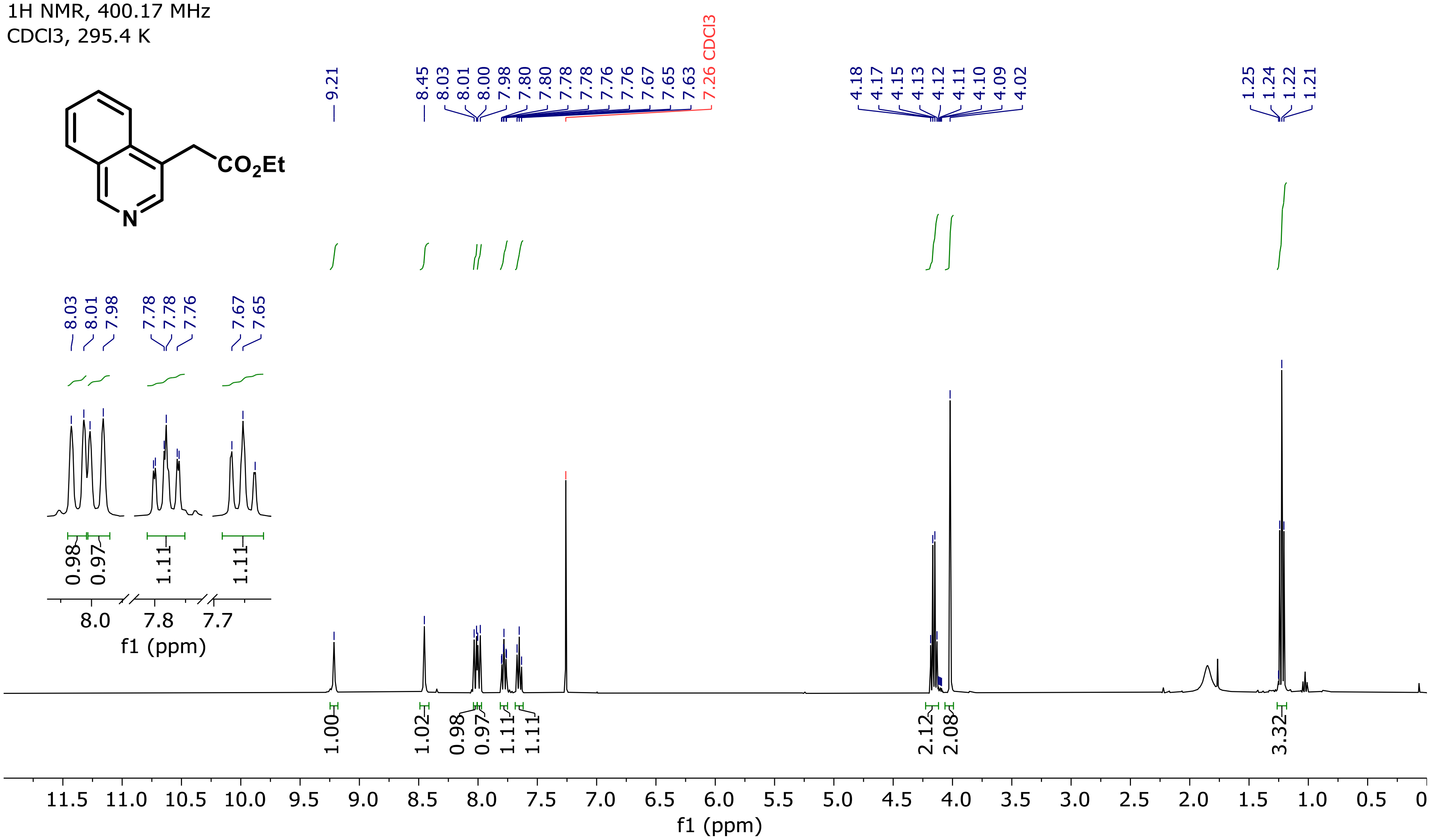

$^{13}$C NMR of ethyl 2-(isoquinolin-4-yl)acetate (37)

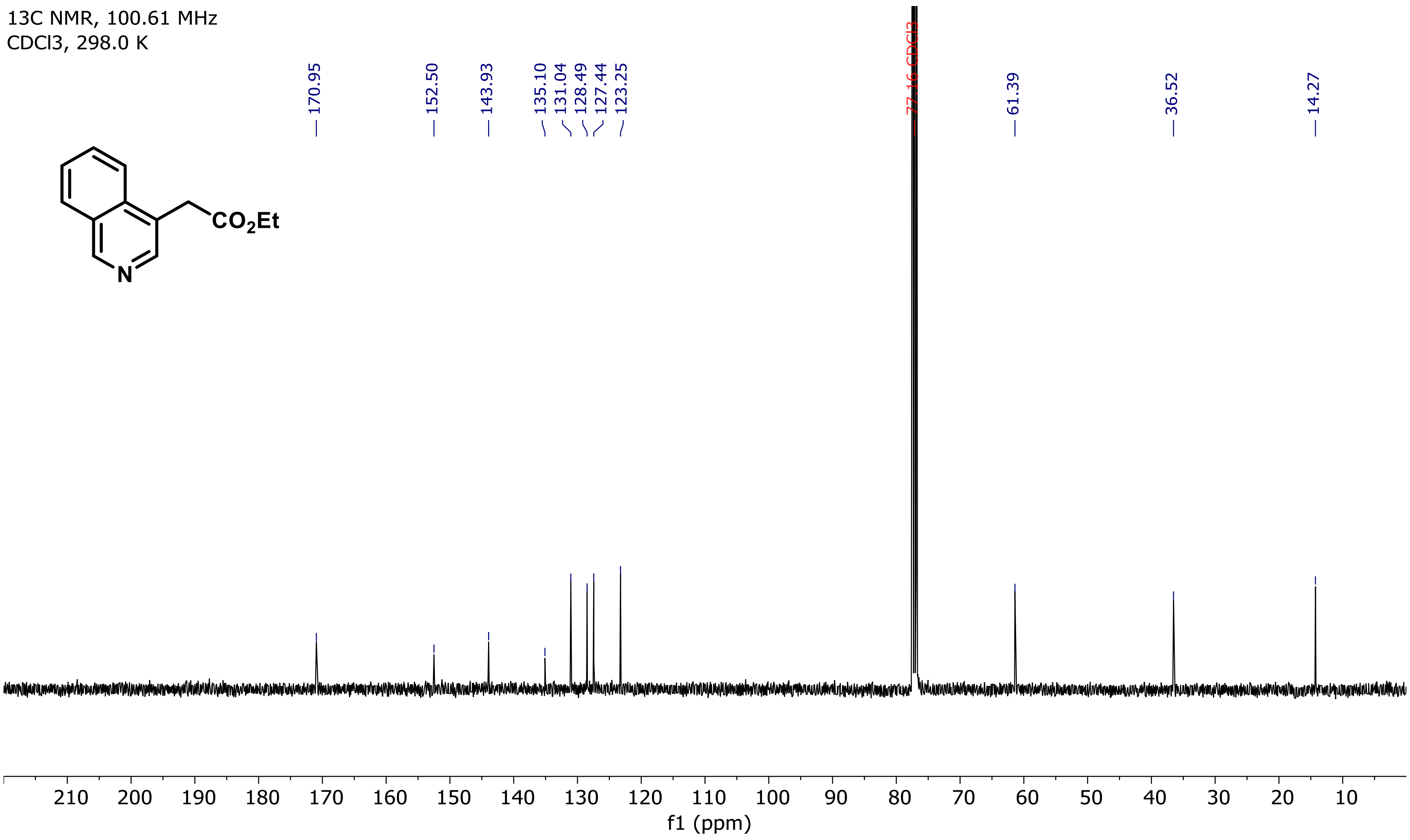

**$^{1}$H NMR of ethyl 2-(quinolin-3-yl)acetate (38)**

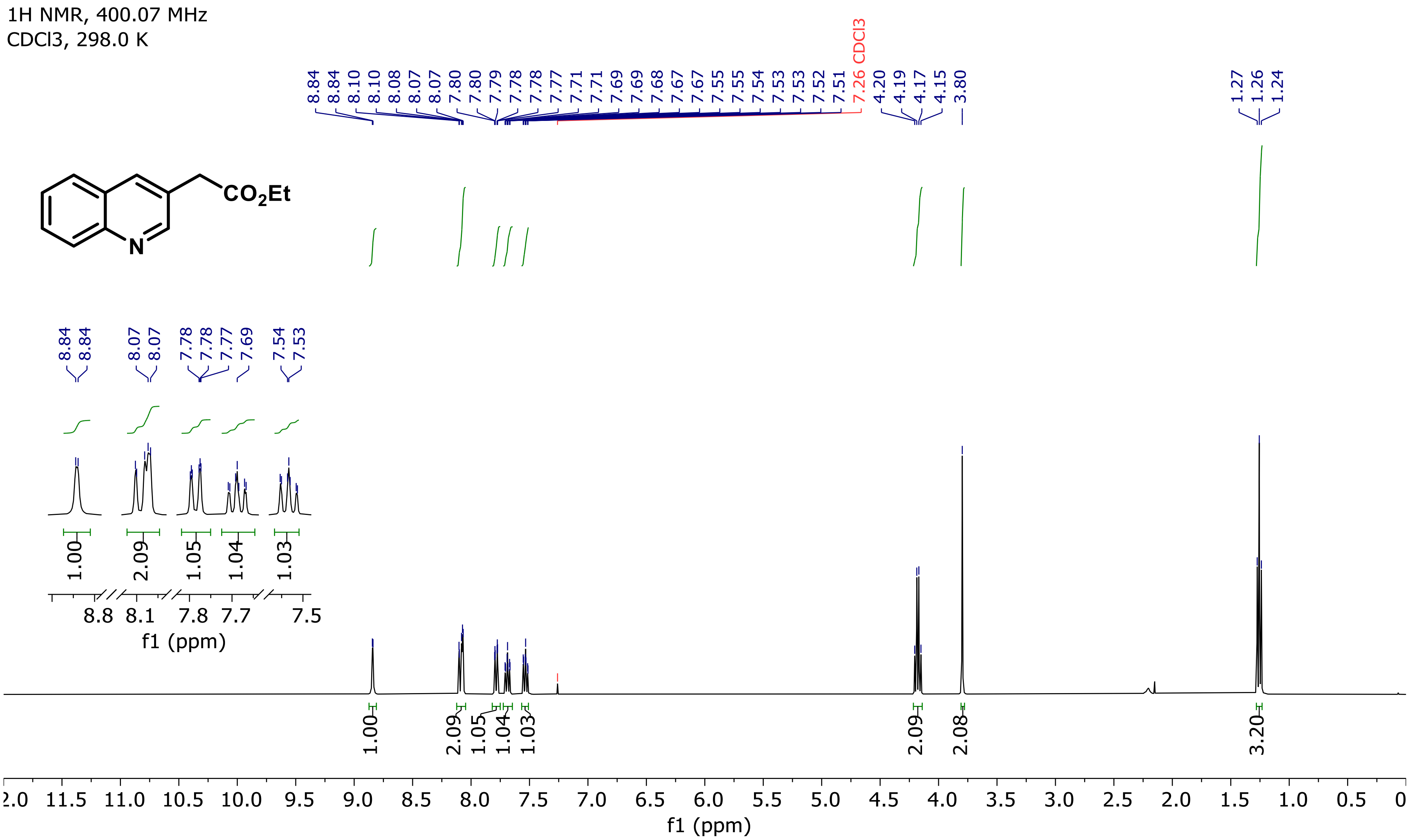

**$^{13}$C NMR of ethyl 2-(quinolin-3-yl)acetate (38)**

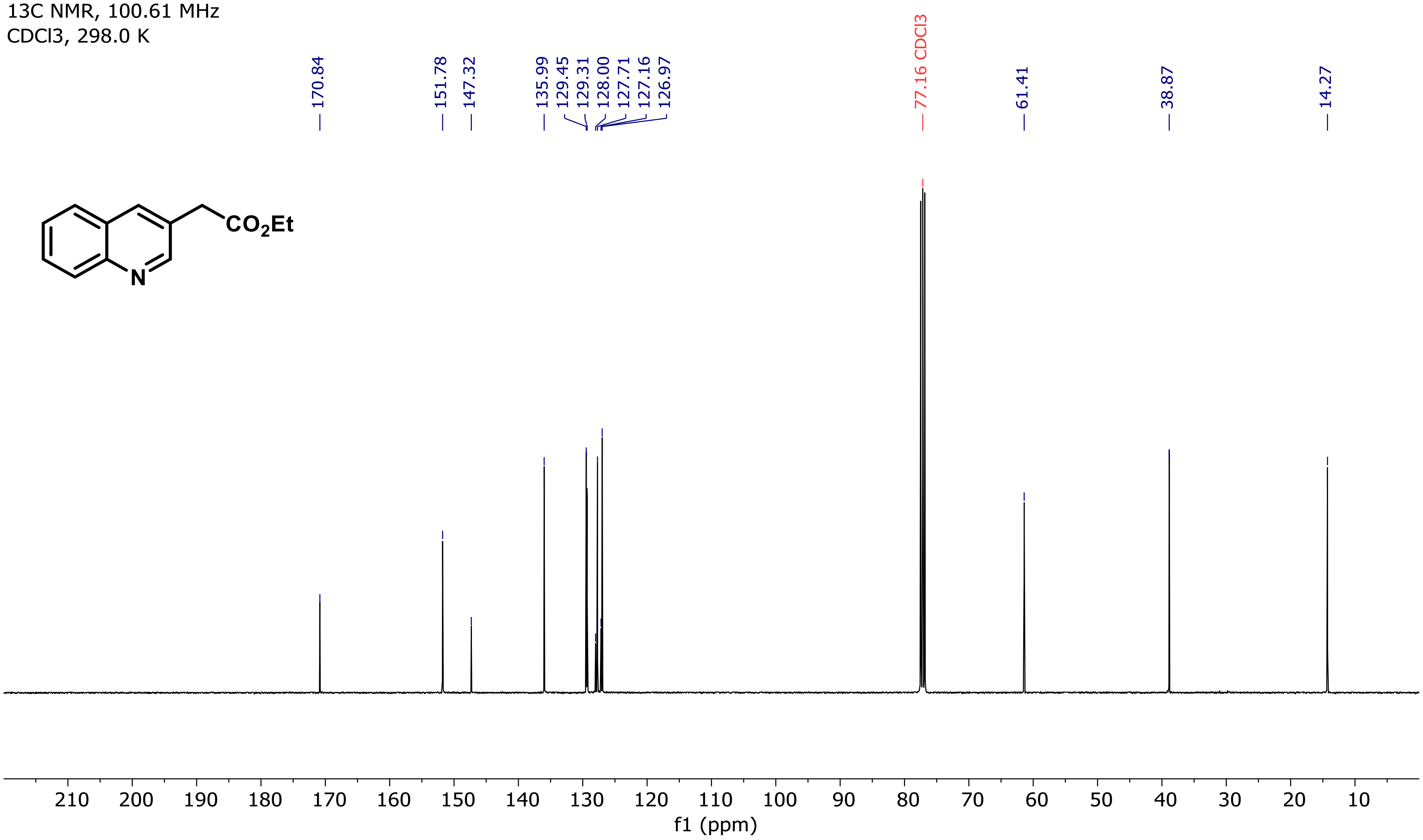

## $^{1}$H NMR of ethyl 2-(quinoxalin-2-yl)acetate (39)

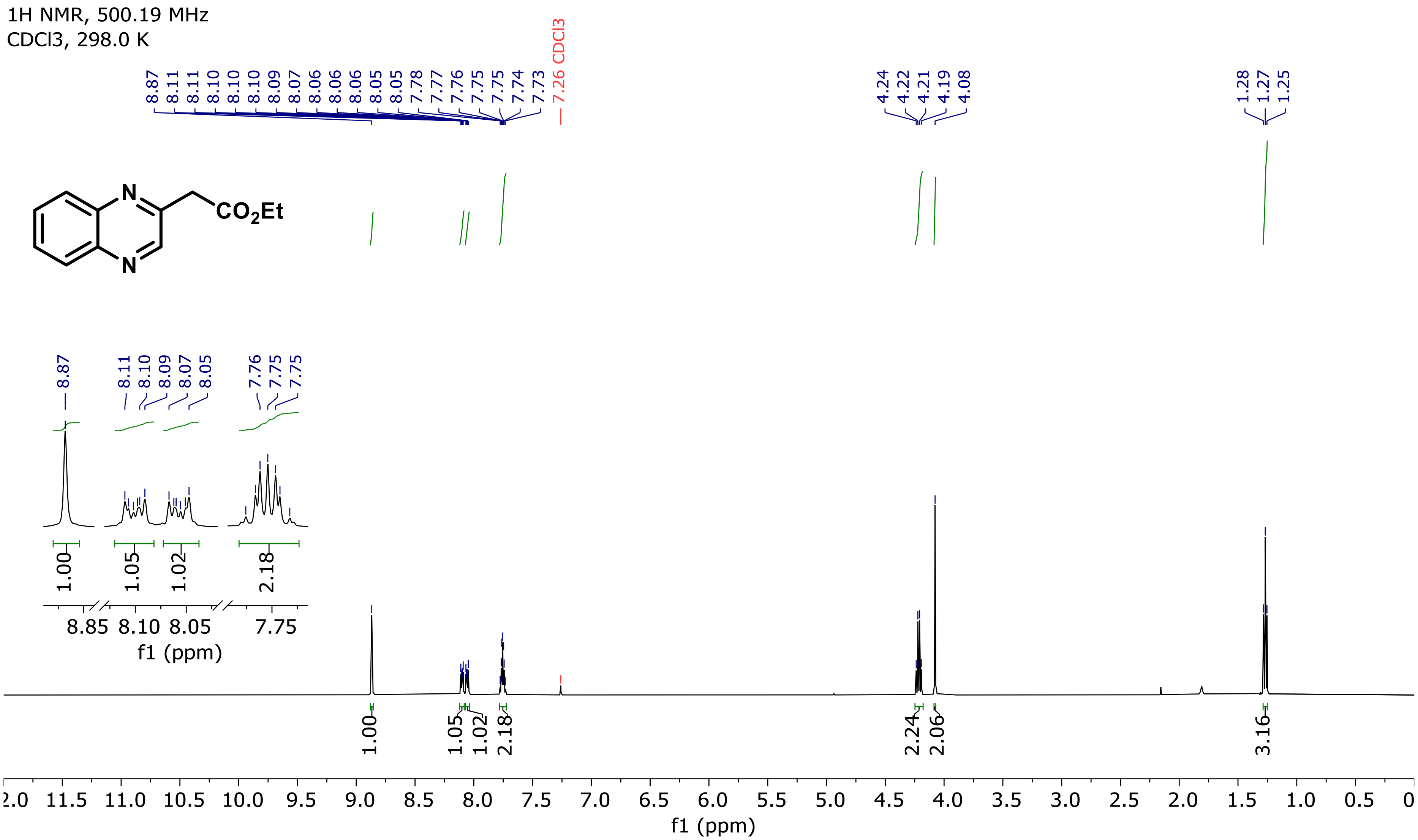

**$^{13}$C NMR of ethyl 2-(quinoxalin-2-yl)acetate (39)**

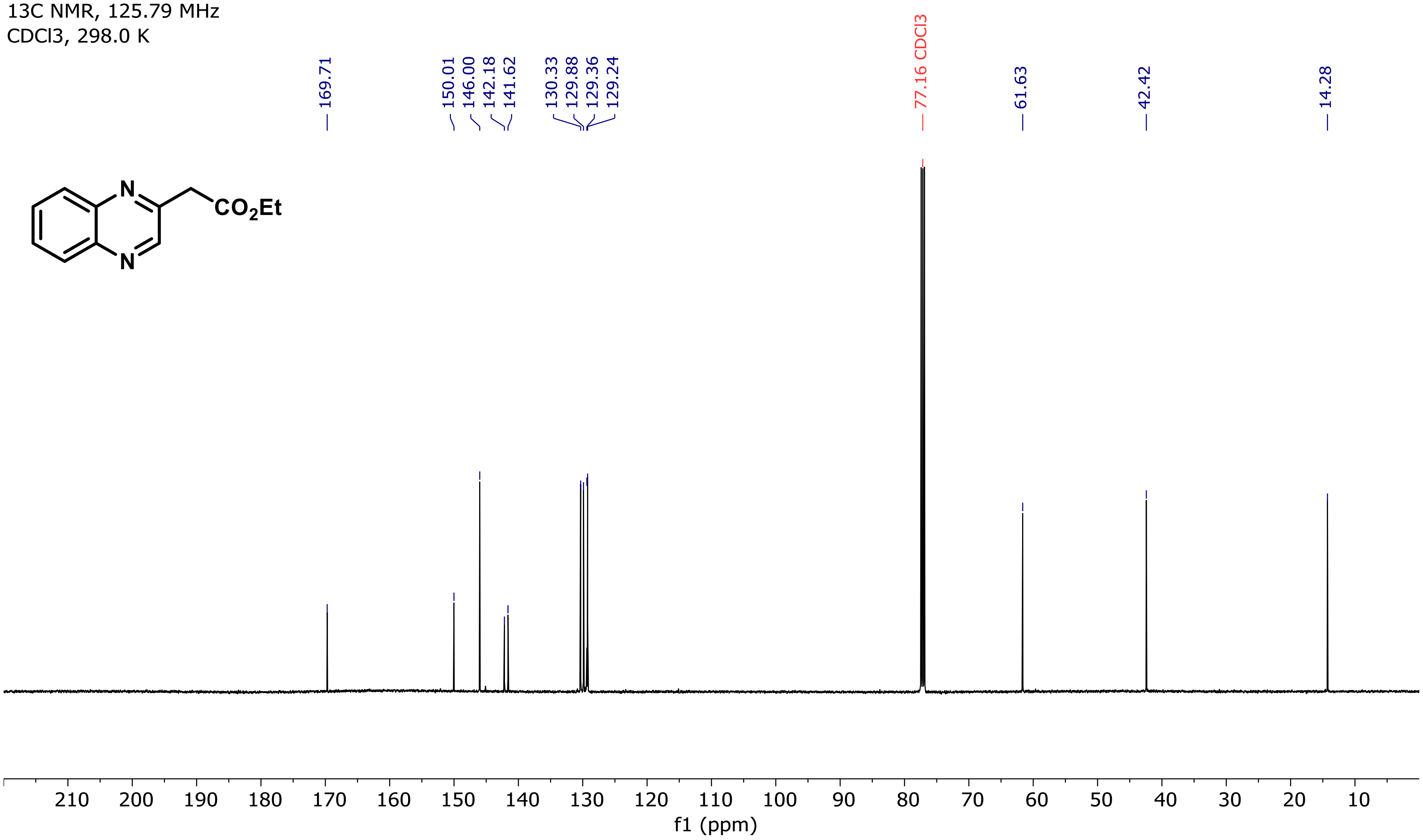

**$^1$H NMR of ethyl 2-(quinazolin-4-yl)acetate (40)**

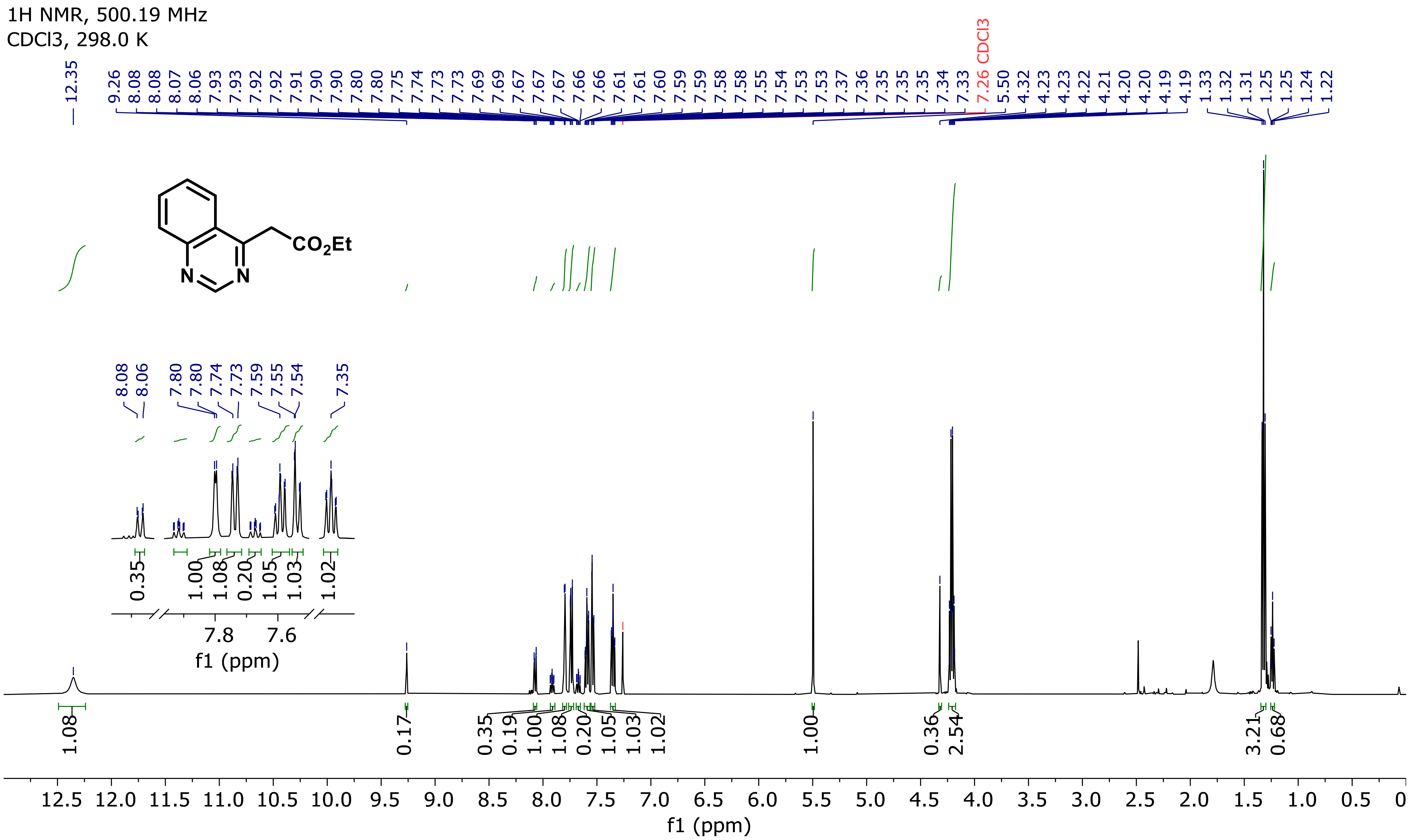

**$^{13}$C NMR of ethyl 2-(quinazolin-4-yl)acetate (40)**

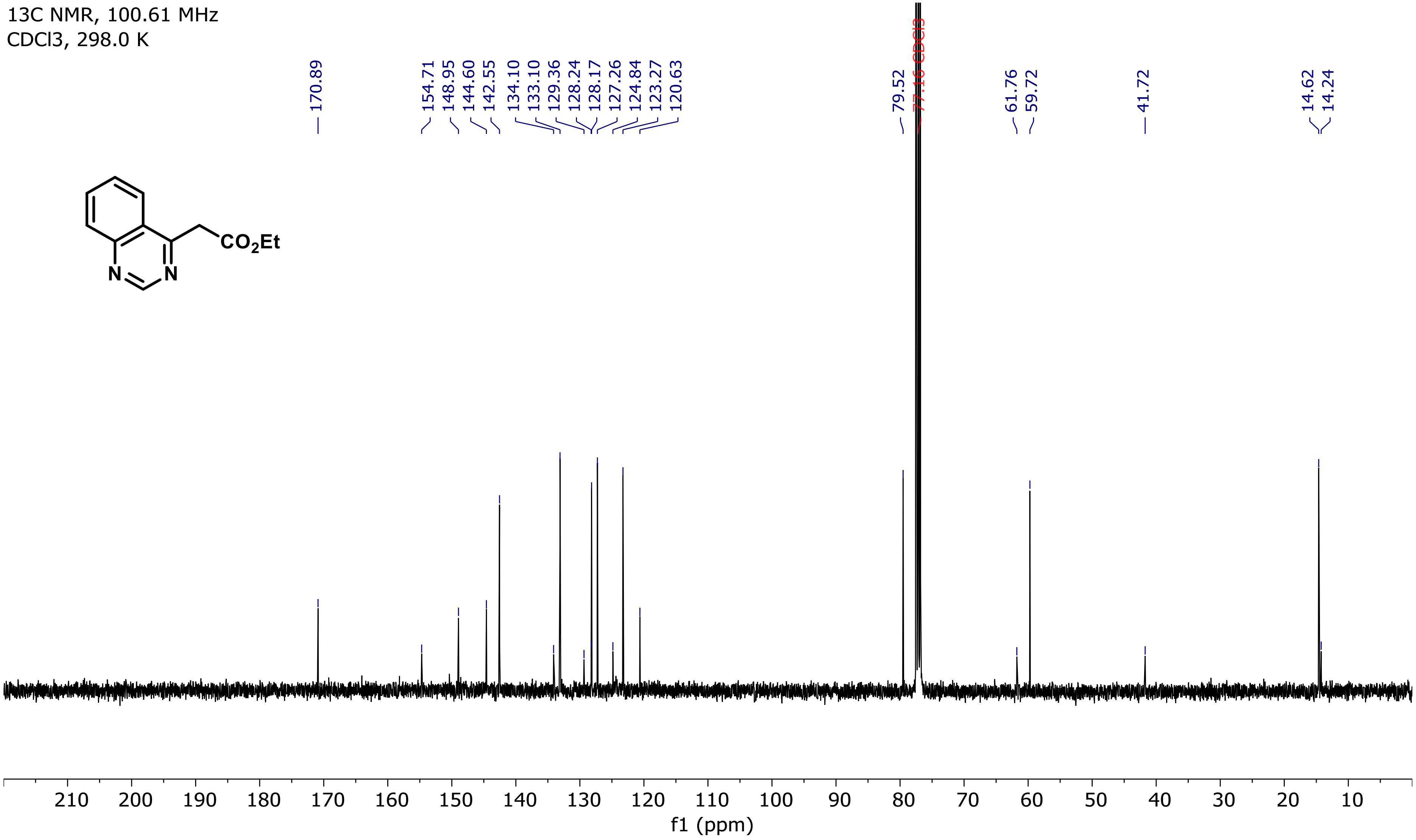

**$^{1}$H NMR of ethyl 2-(benzo[*d*]thiazol-2-yl)acetate (41)**

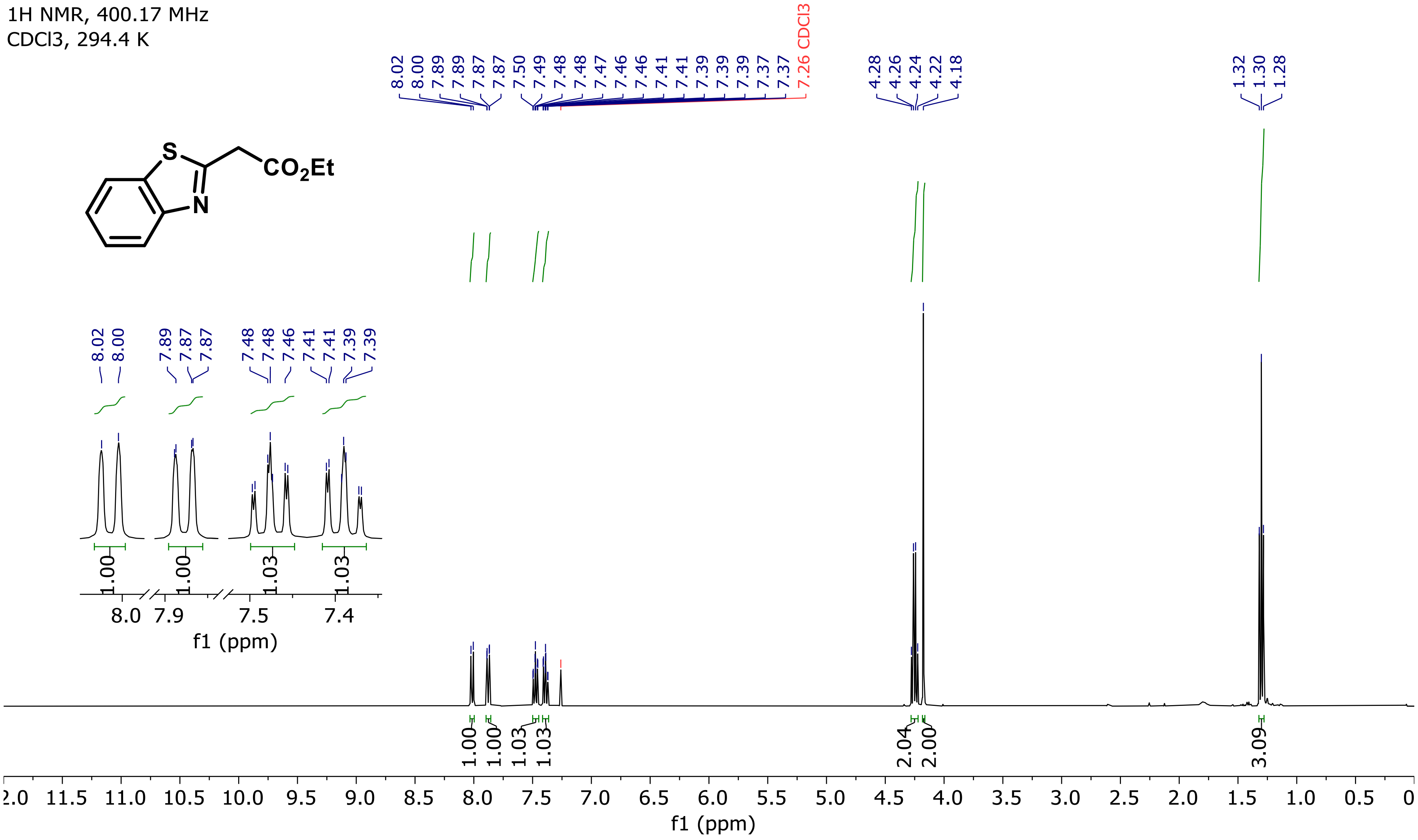

**$^{13}$C NMR of ethyl 2-(benzo[*d*]thiazol-2-yl)acetate (41)**

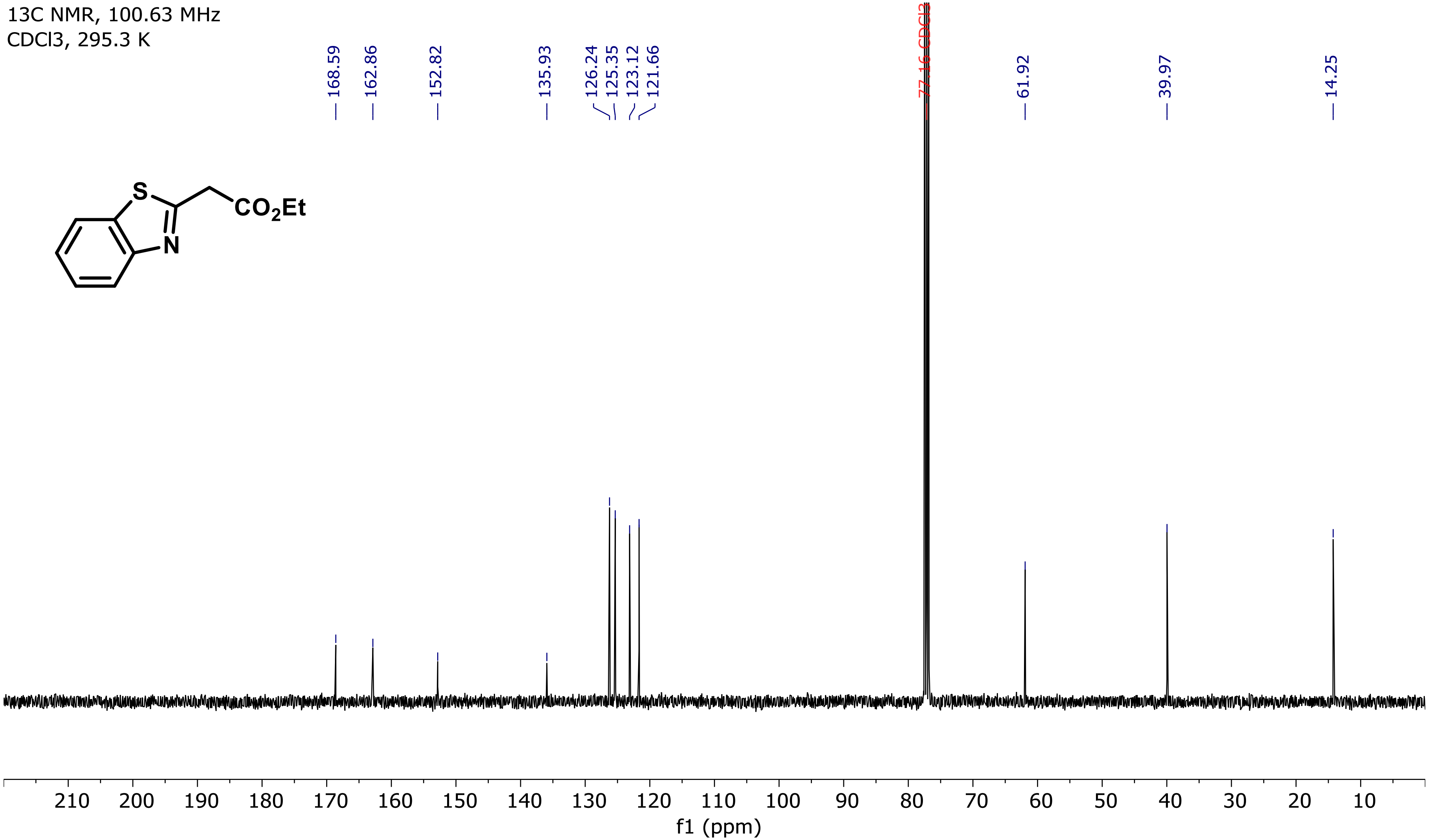

**$^{1}$H NMR of ethyl 2-(pyrazolo[1,5-a]pyrimidin-5-yl)acetate (42)**

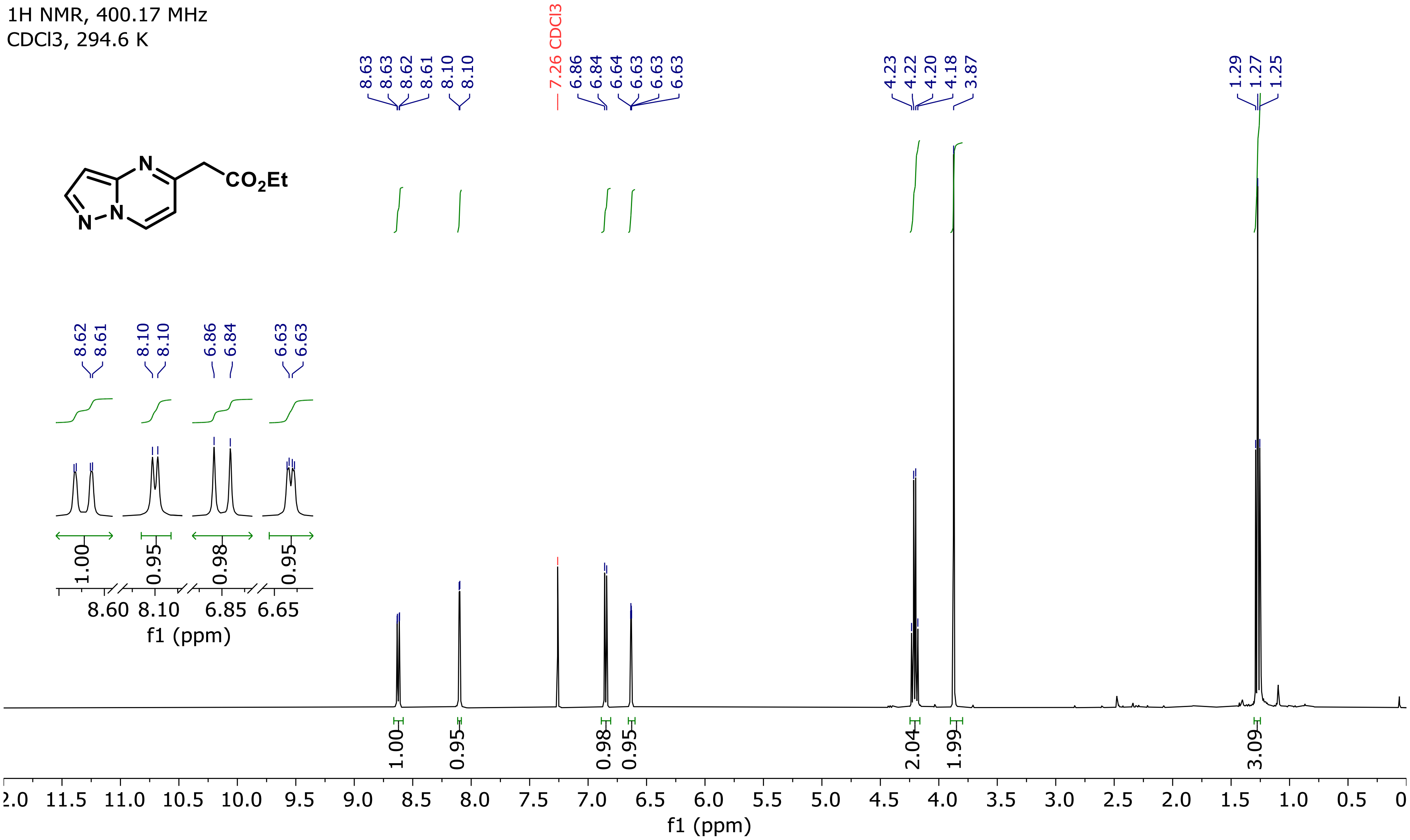

**$^{13}$C NMR of ethyl 2-(pyrazolo[1,5-a]pyrimidin-5-yl)acetate (42)**

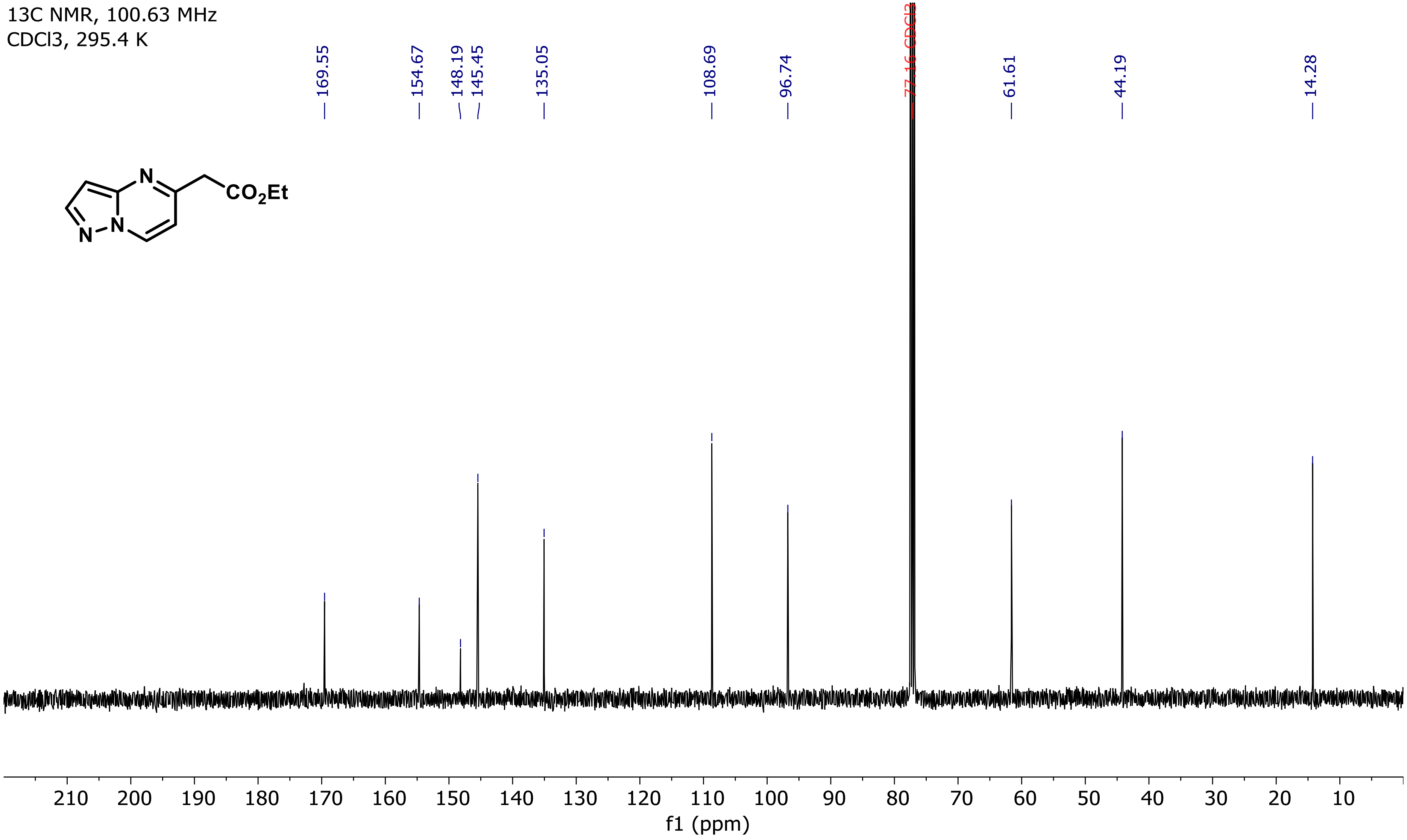

**$^{1}$H NMR of ethyl 2-(imidazo[1,2-*b*]pyridazin-6-yl)acetate (43)**

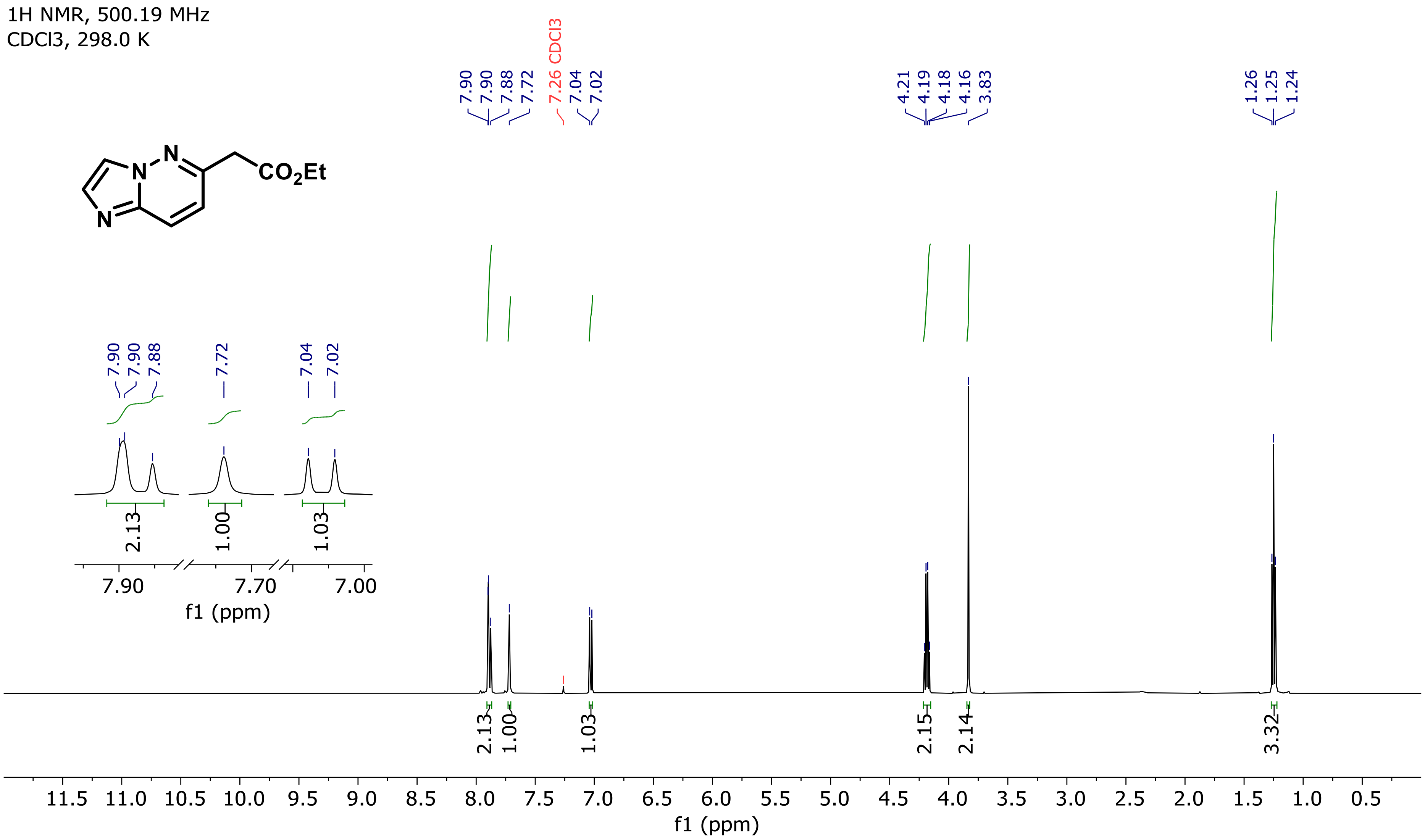

**$^{13}$C NMR of ethyl 2-(imidazo[1,2-*b*]pyridazin-6-yl)acetate (43)**

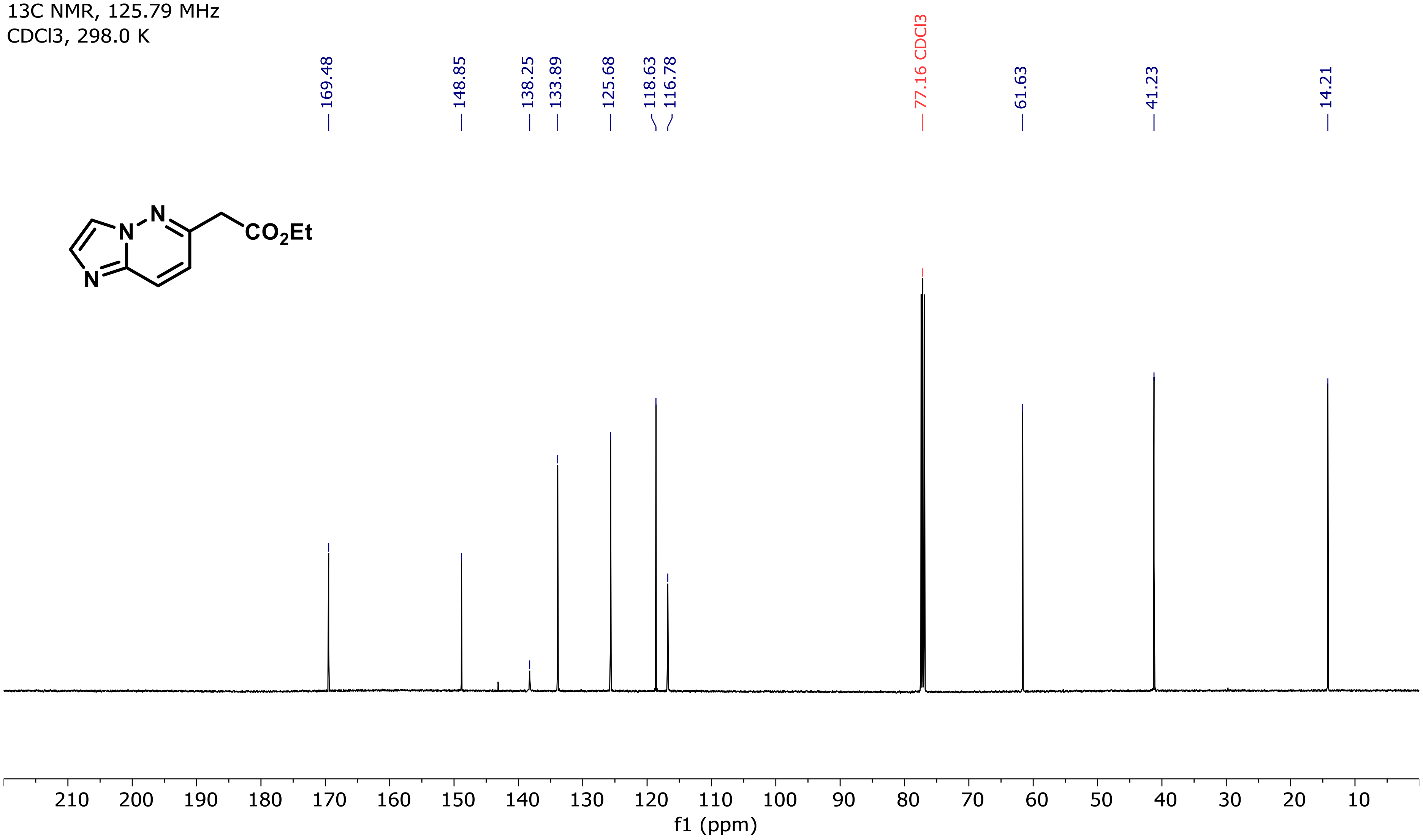

**$^{1}$H NMR of methyl 2-(4-cyanophenyl)acetate (69)**

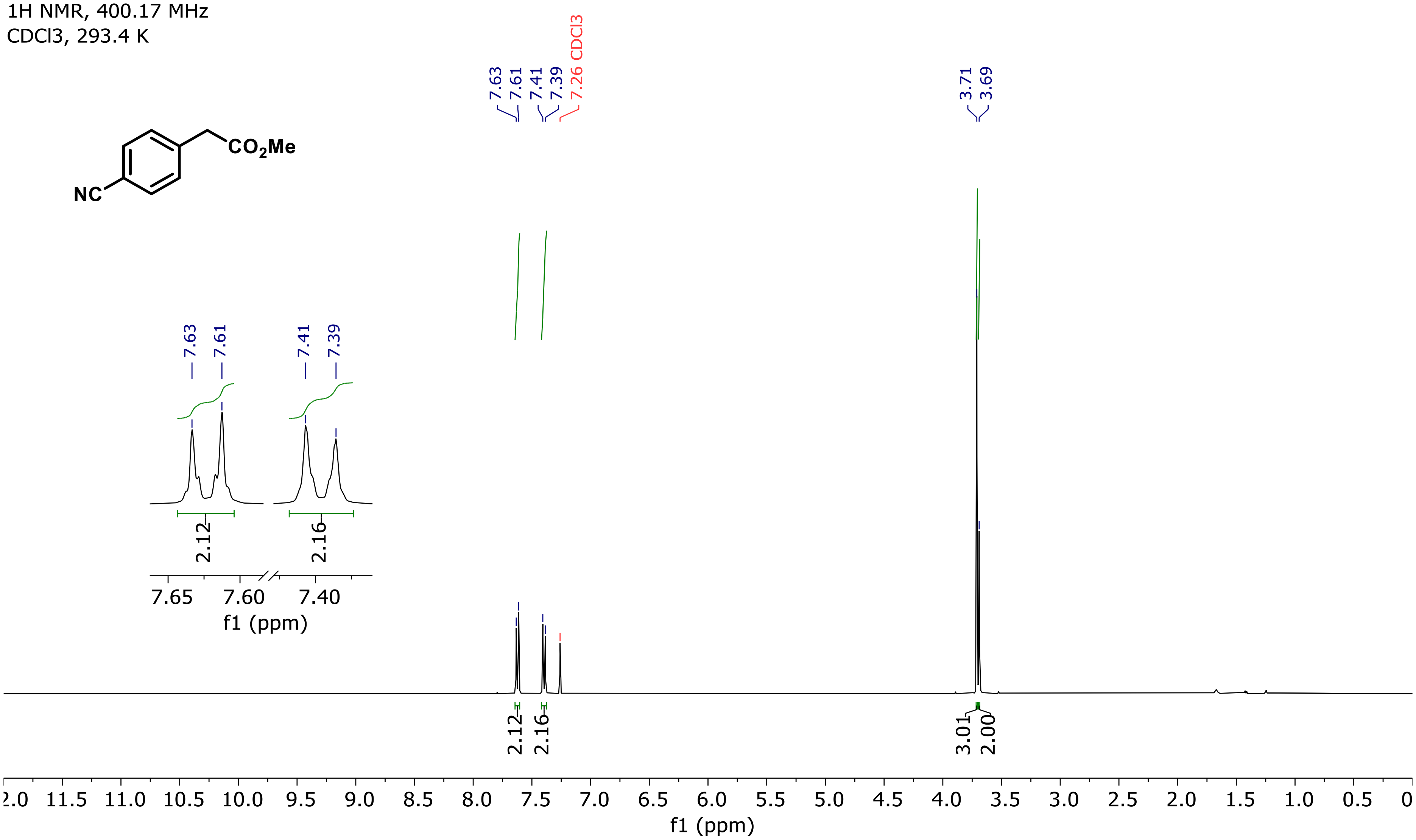

**$^{13}$C NMR of methyl 2-(4-cyanophenyl)acetate (69)**

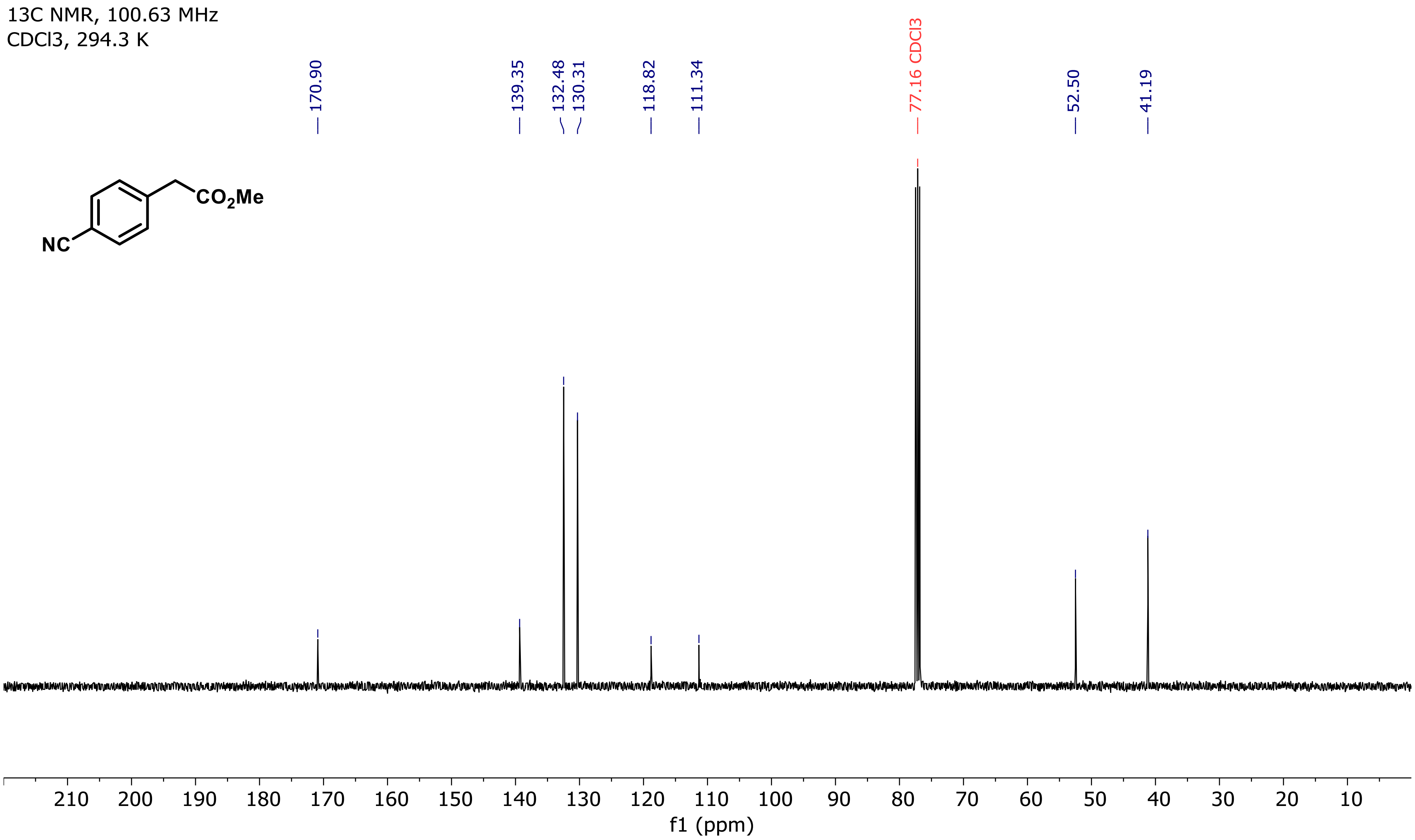

$^{1}$H NMR of *tert*-butyl 2-(4-cyanophenyl)acetate (70)

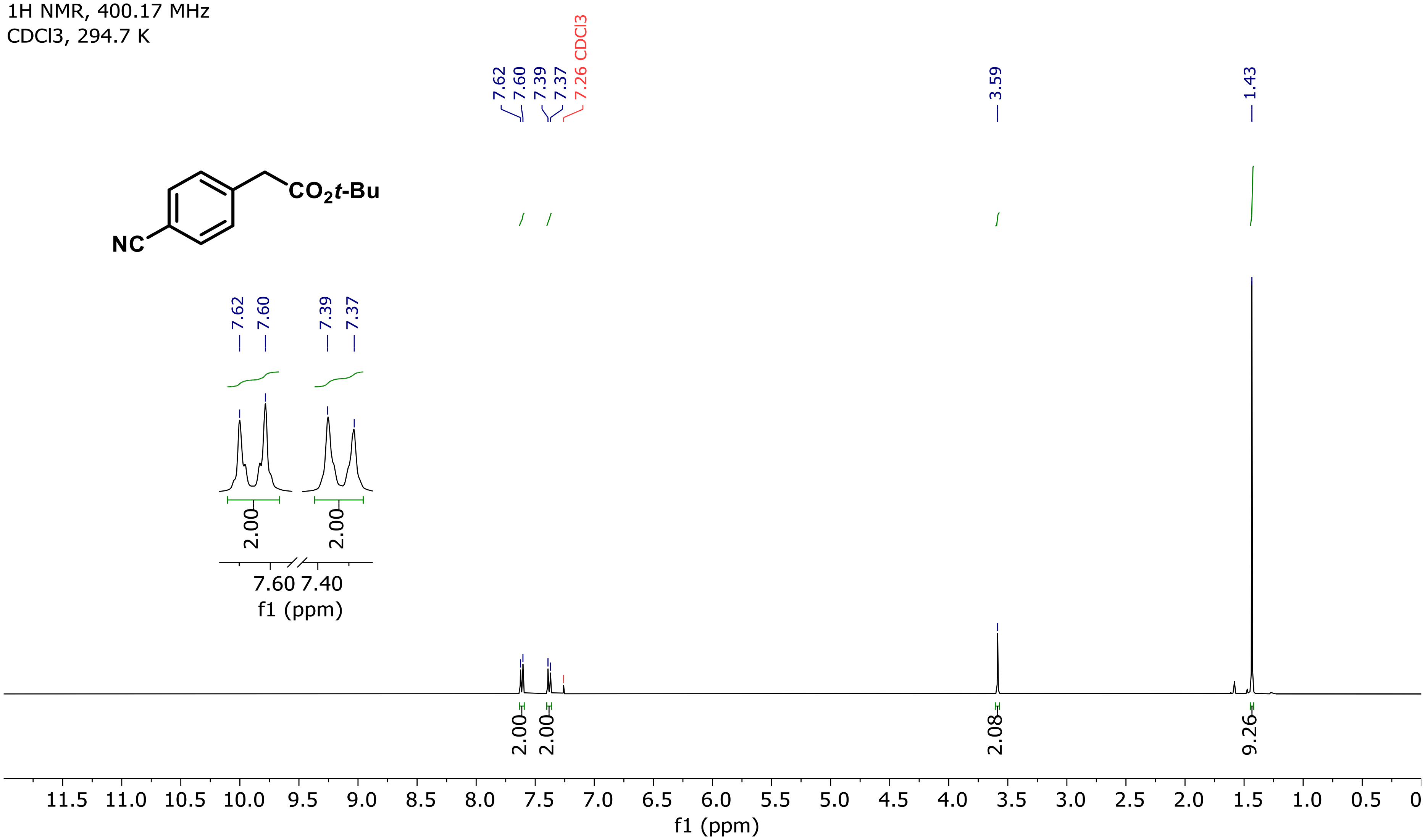

**$^{13}$C NMR of *tert*-butyl 2-(4-cyanophenyl)acetate (70)**

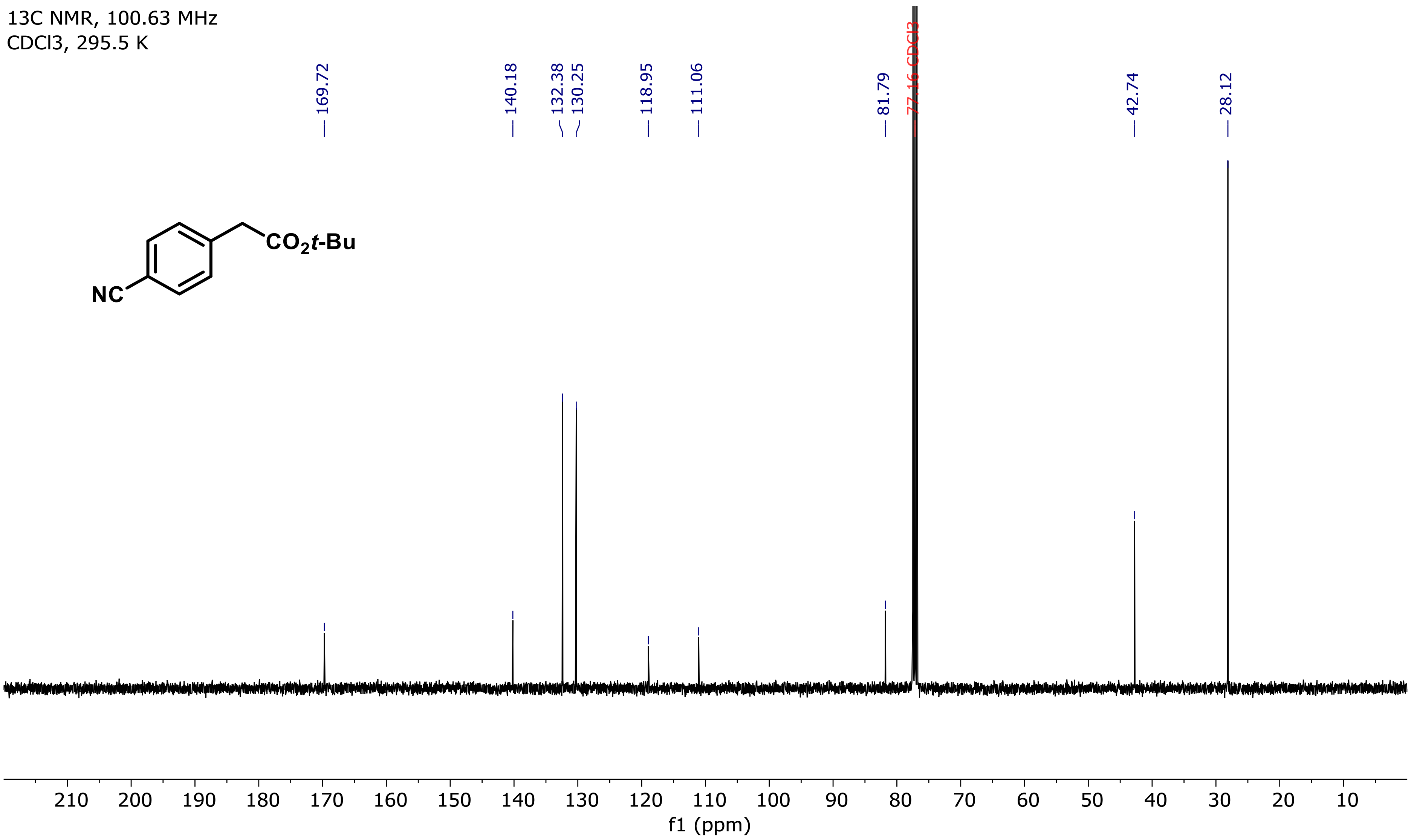

**$^{1}$H NMR of 4-(2-oxopropyl)benzonitrile (72)**

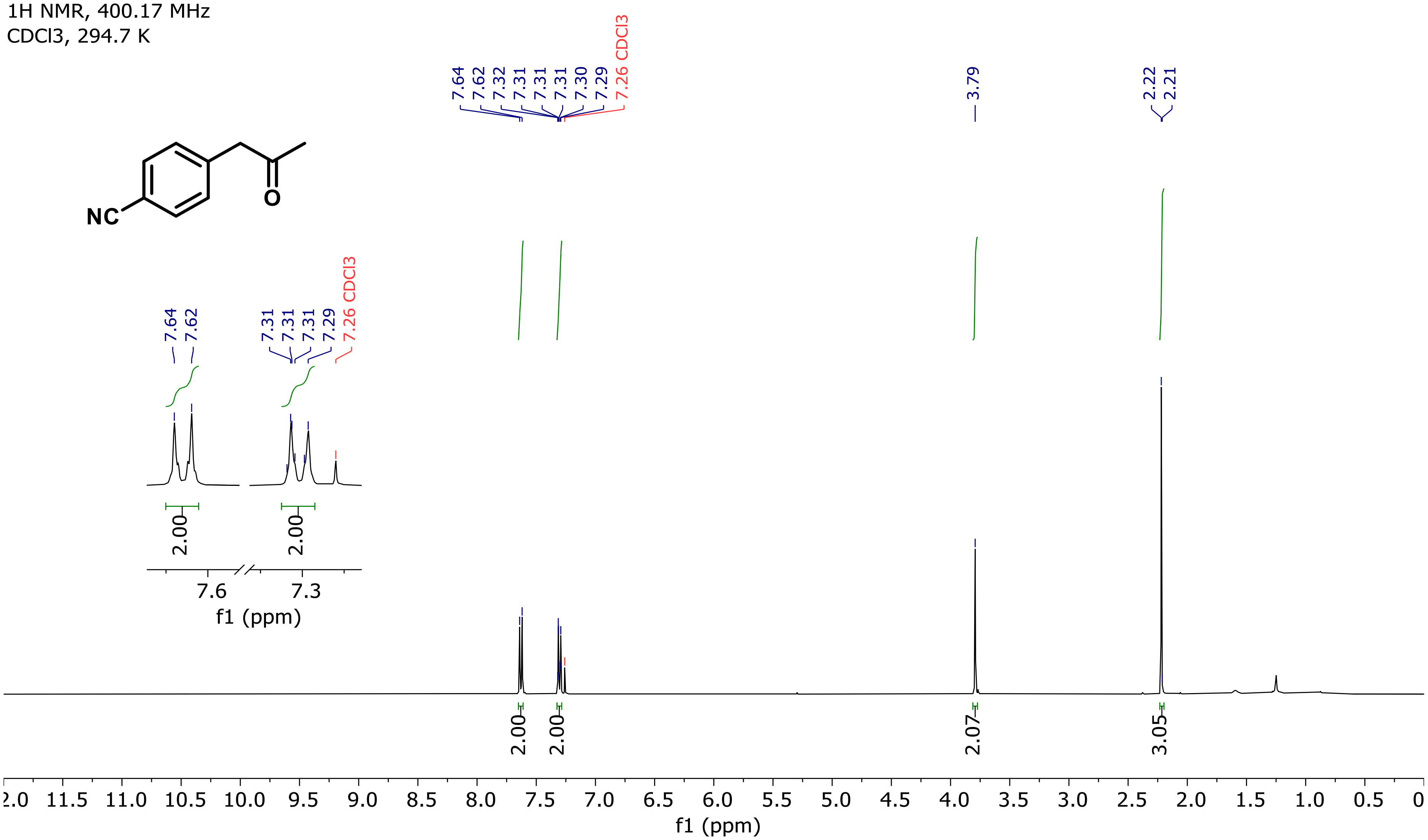

**$^{13}$C NMR of 4-(2-oxopropyl)benzonitrile (72)**

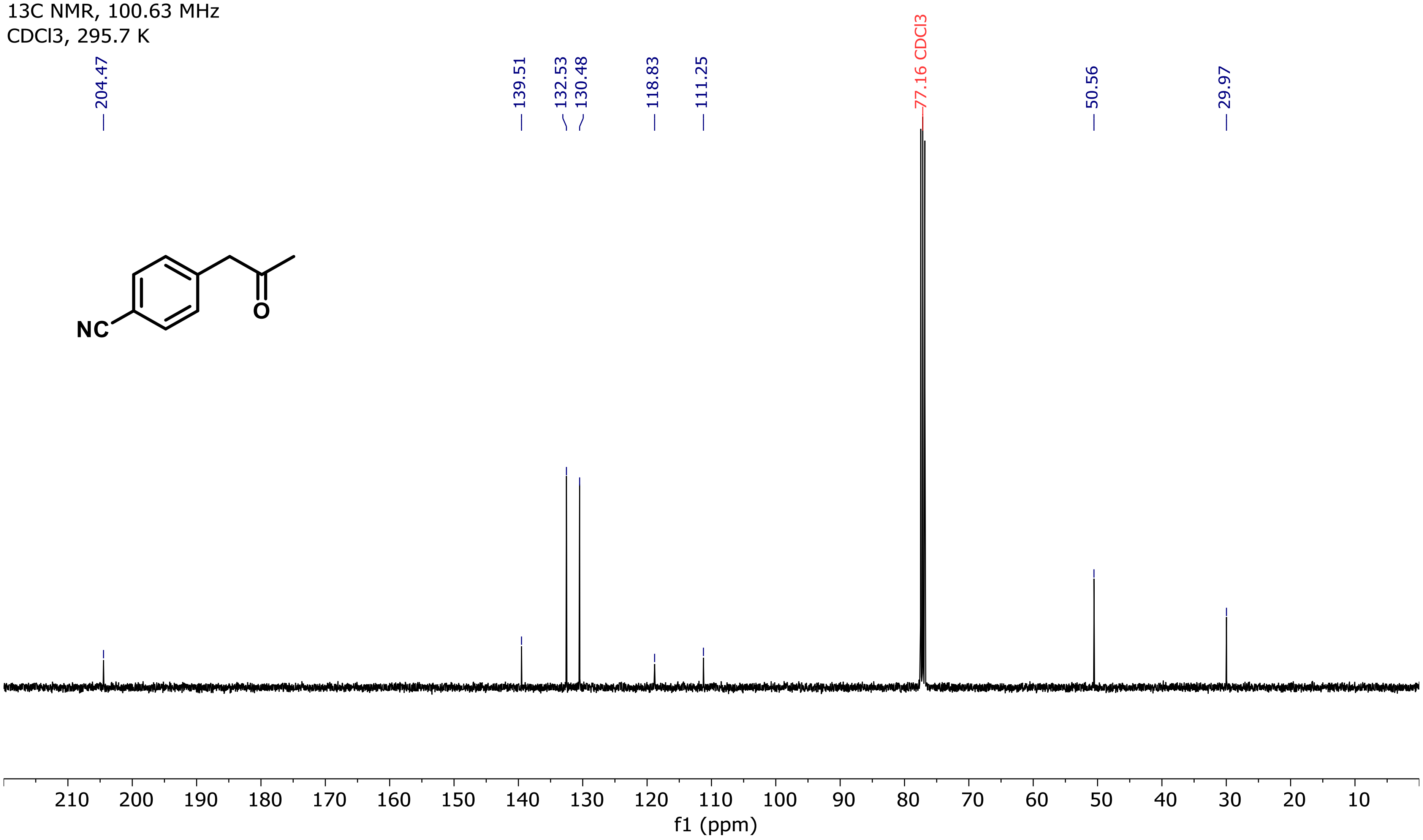

**$^{1}$H NMR of 2-(4-cyanophenyl)-*N*,*N*-diethyl-3-oxobutanamide (74)**

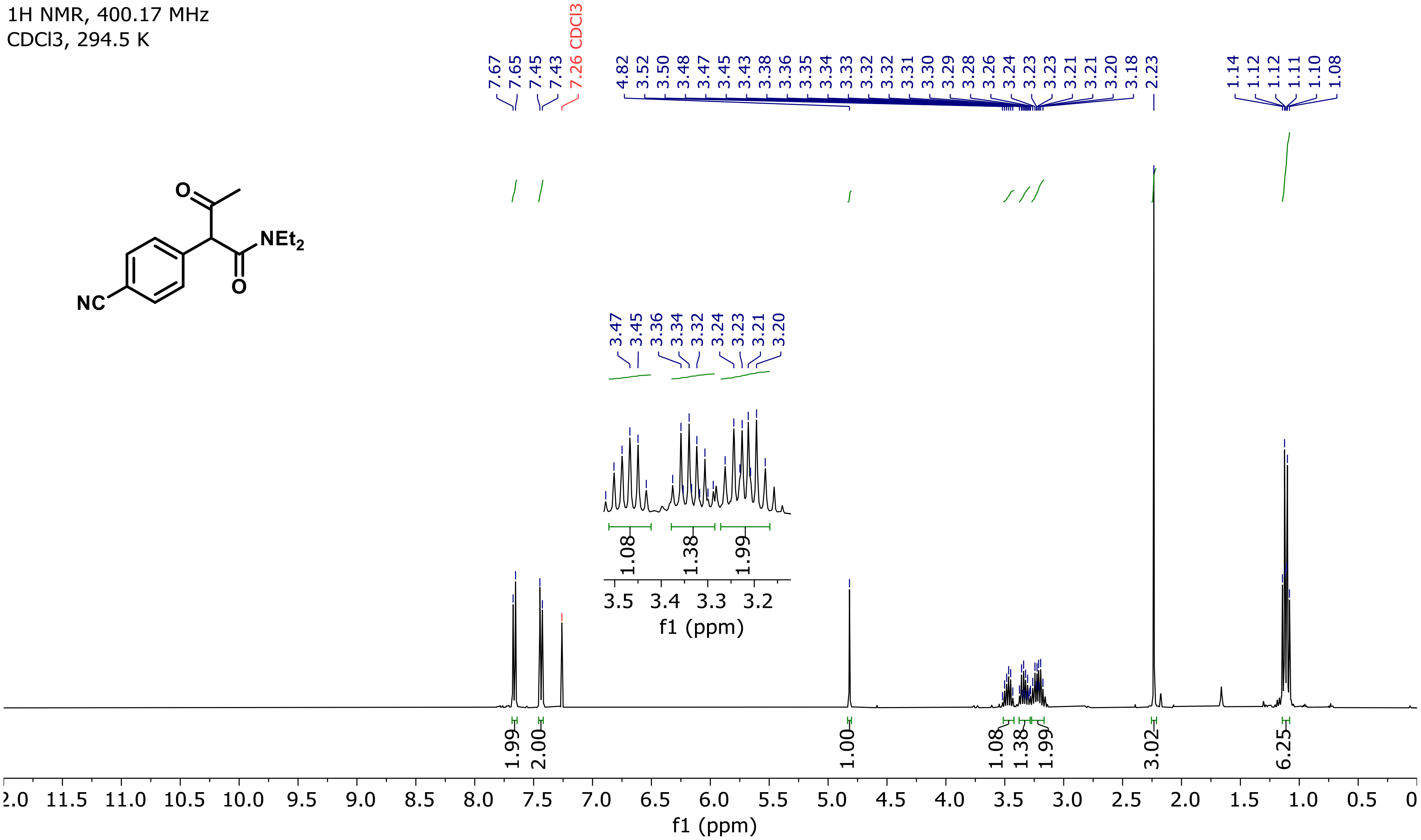

**$^{13}$C NMR of 2-(4-cyanophenyl)-*N*,*N*-diethyl-3-oxobutanamide (74)**

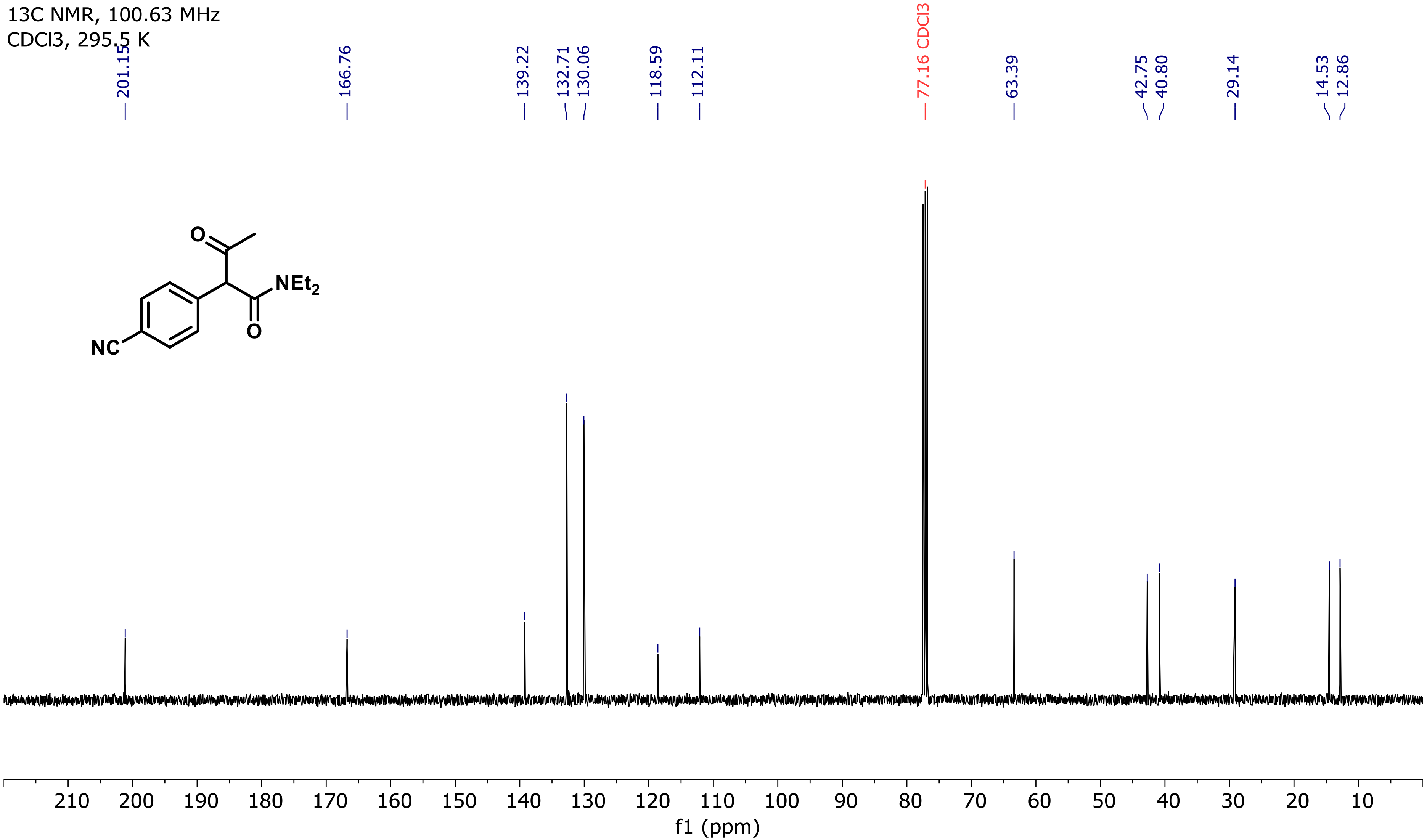

**$^{1}$H NMR of ethyl 2-(4-cyanophenyl)propanoate (76)**

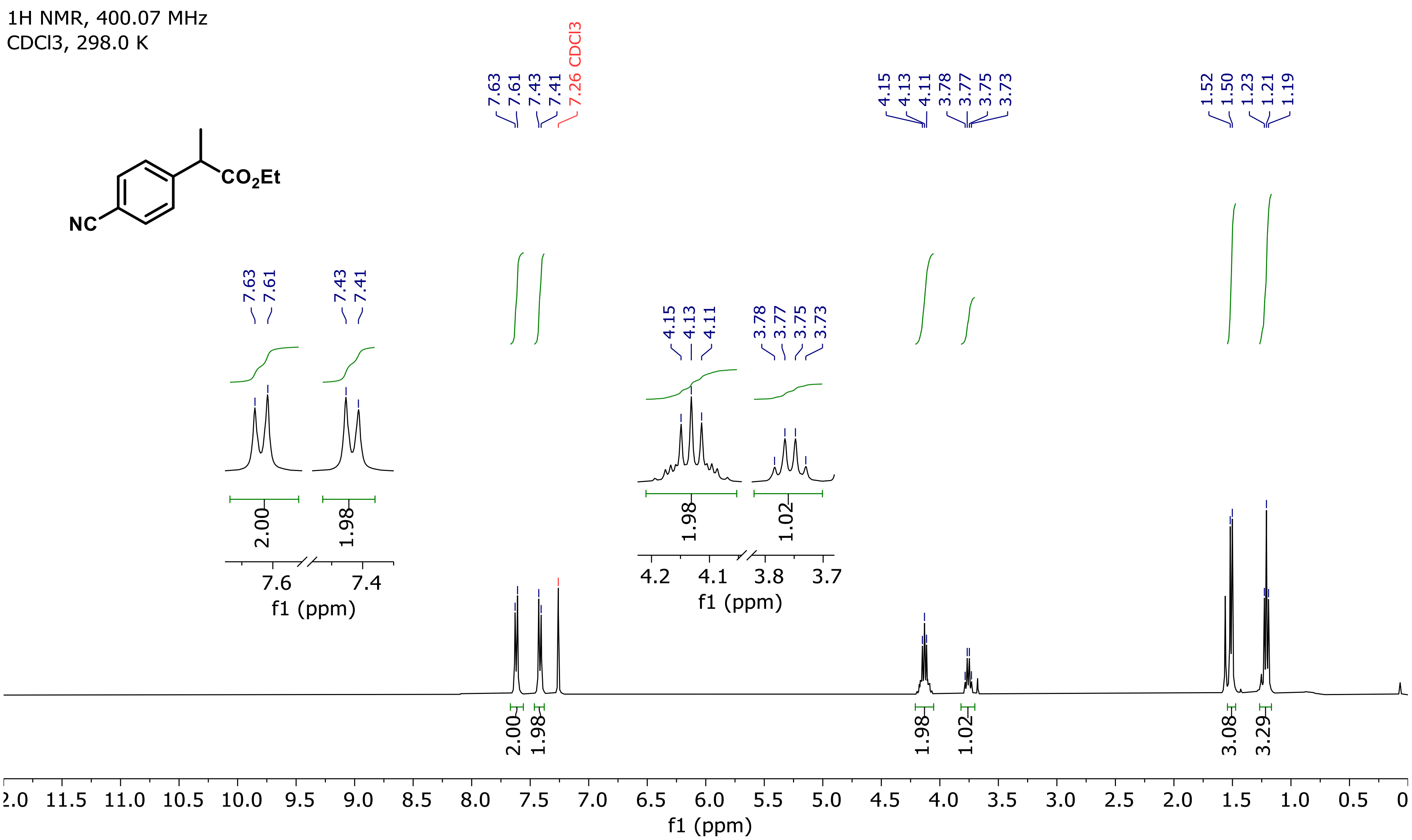

**$^{13}$C NMR of ethyl 2-(4-cyanophenyl)propanoate (76)**

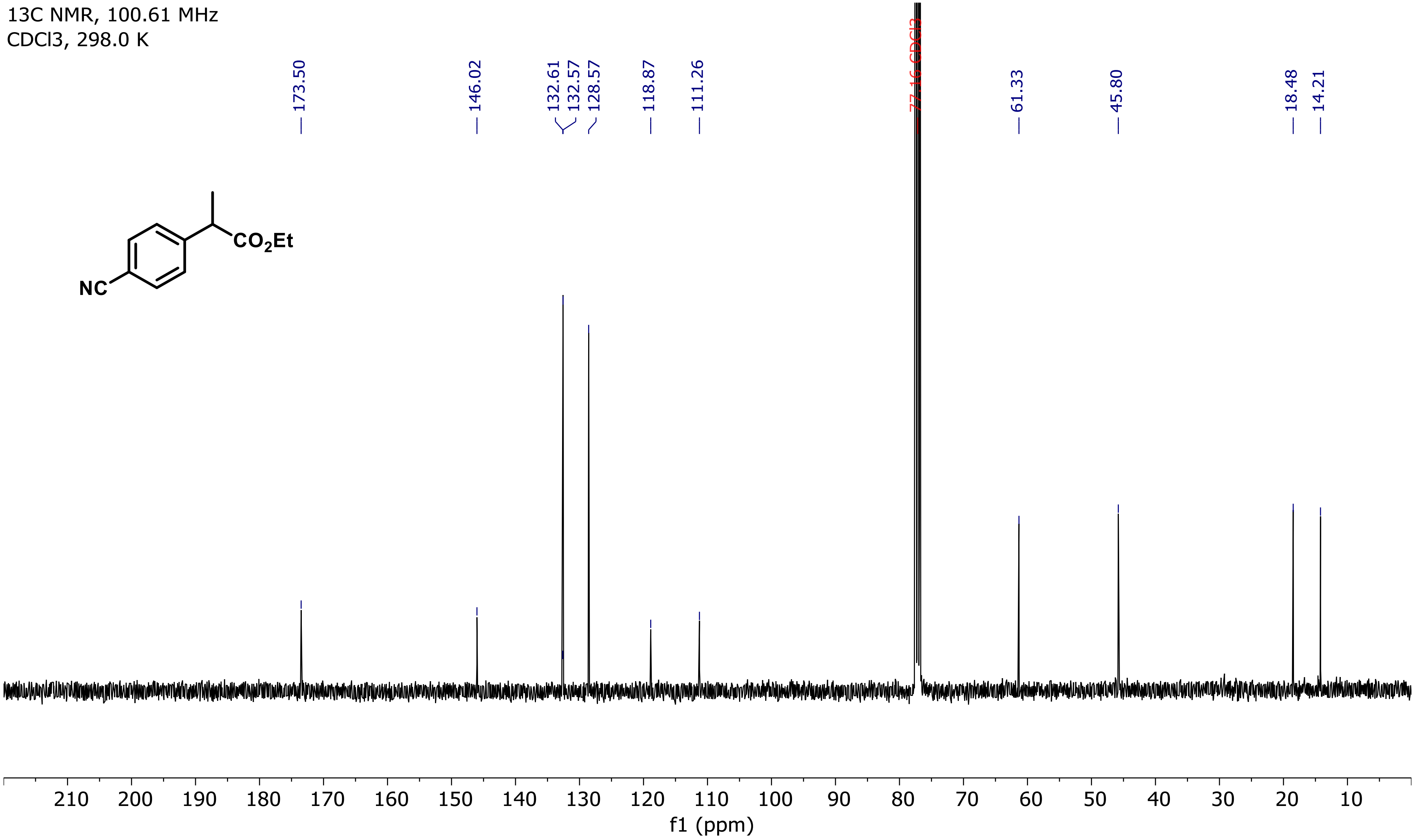

**$^{1}$H NMR used to determine yield of ethyl 2-(4-cyanophenyl)propanoate (76)**

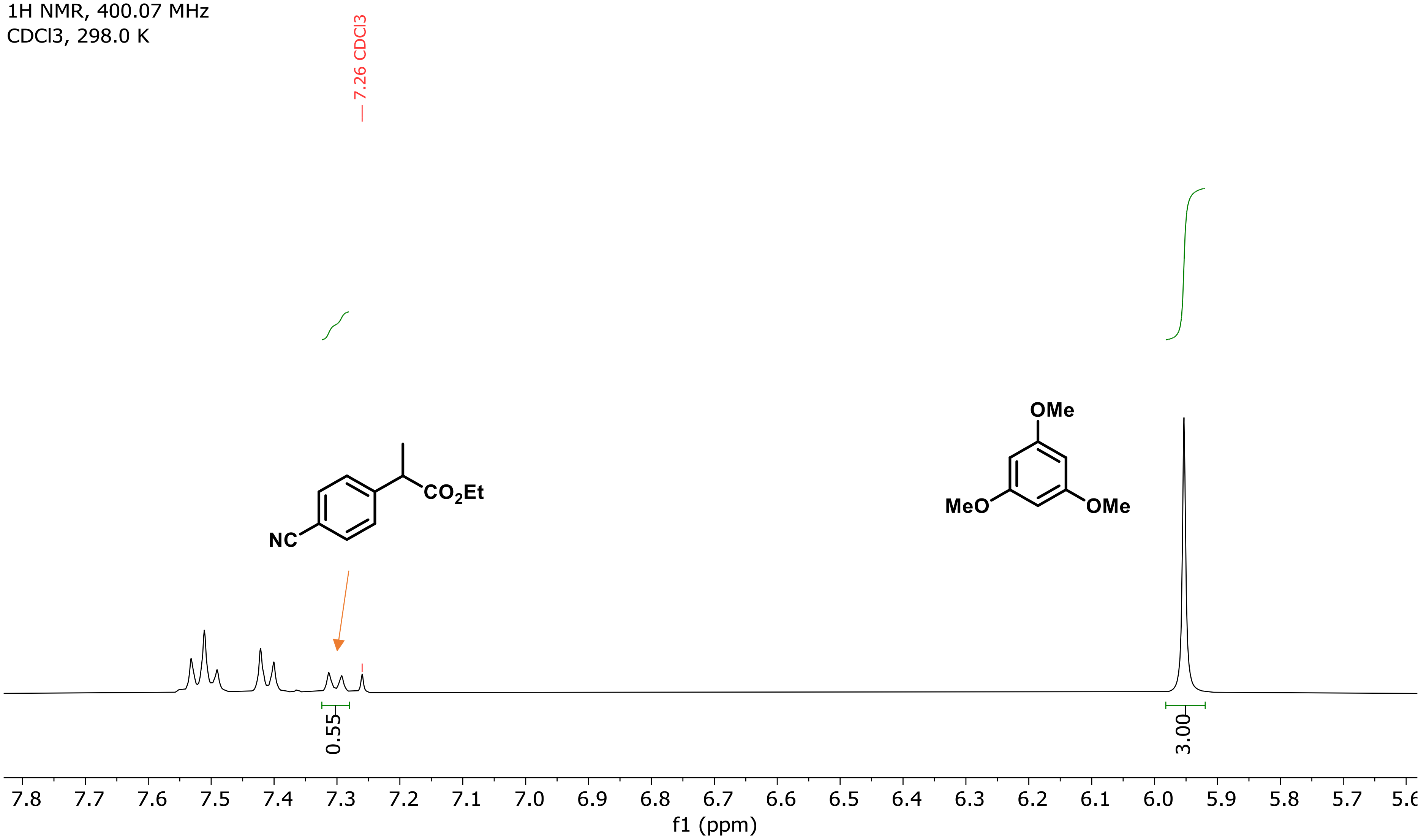

**$^{1}$H NMR of ethyl 2-(4-(thiophene-2-carbonyl)phenyl)propanoate (78)**

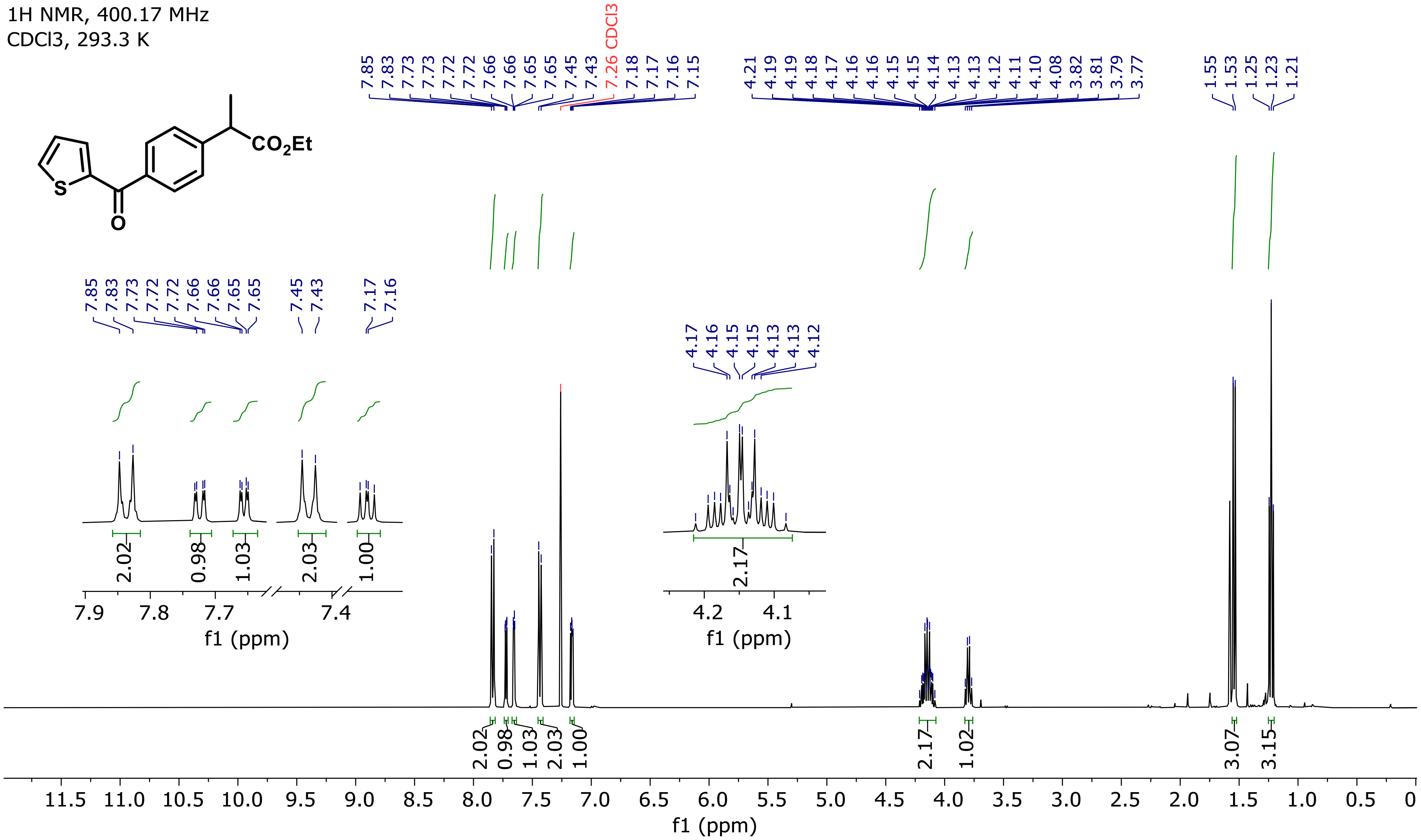

**$^{13}$C NMR of ethyl 2-(4-(thiophene-2-carbonyl)phenyl)propanoate (78)**

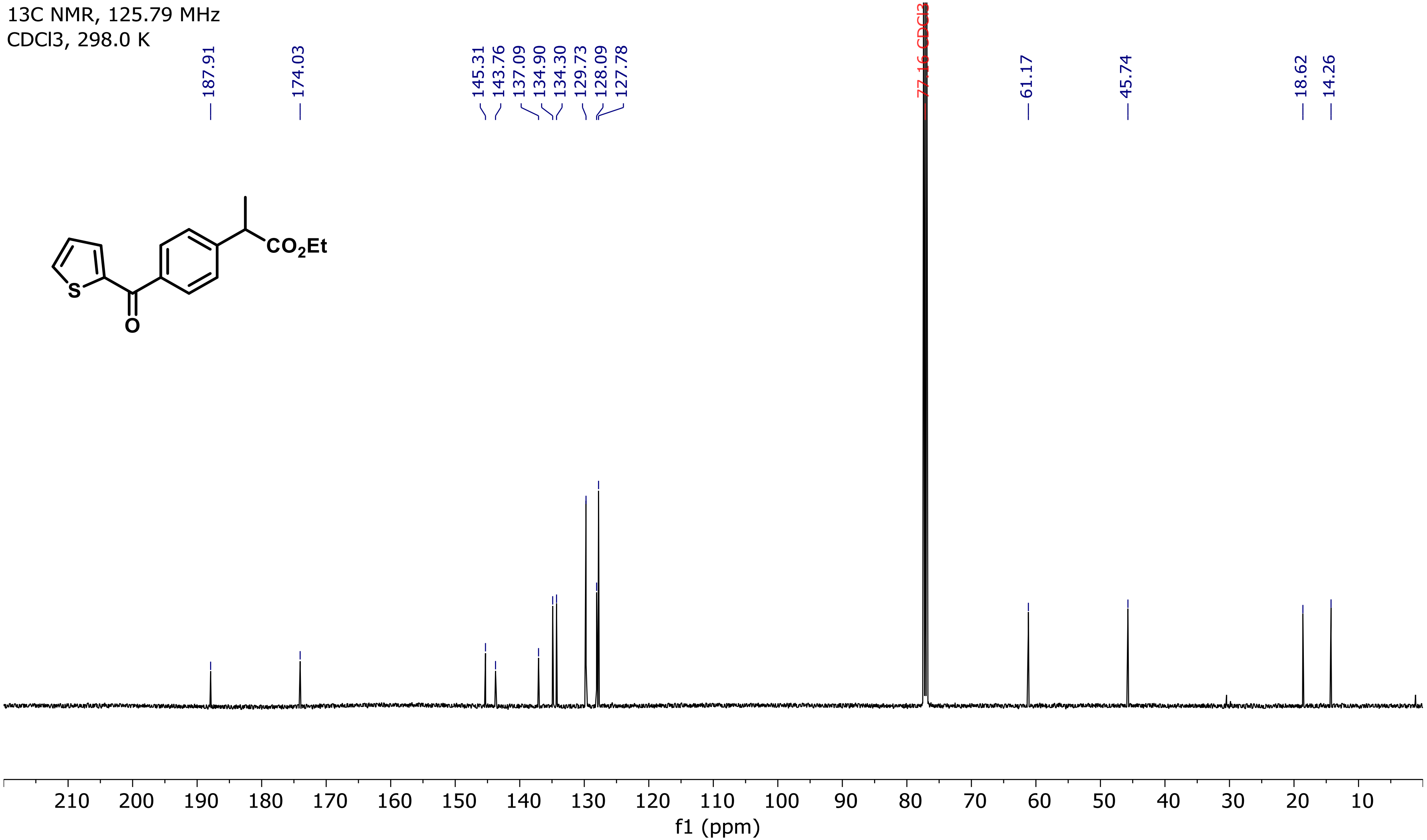

**$^{1}$H NMR of ethyl 1-(4-cyanophenyl)-2-oxocyclohexane-1-carboxylate (80)**

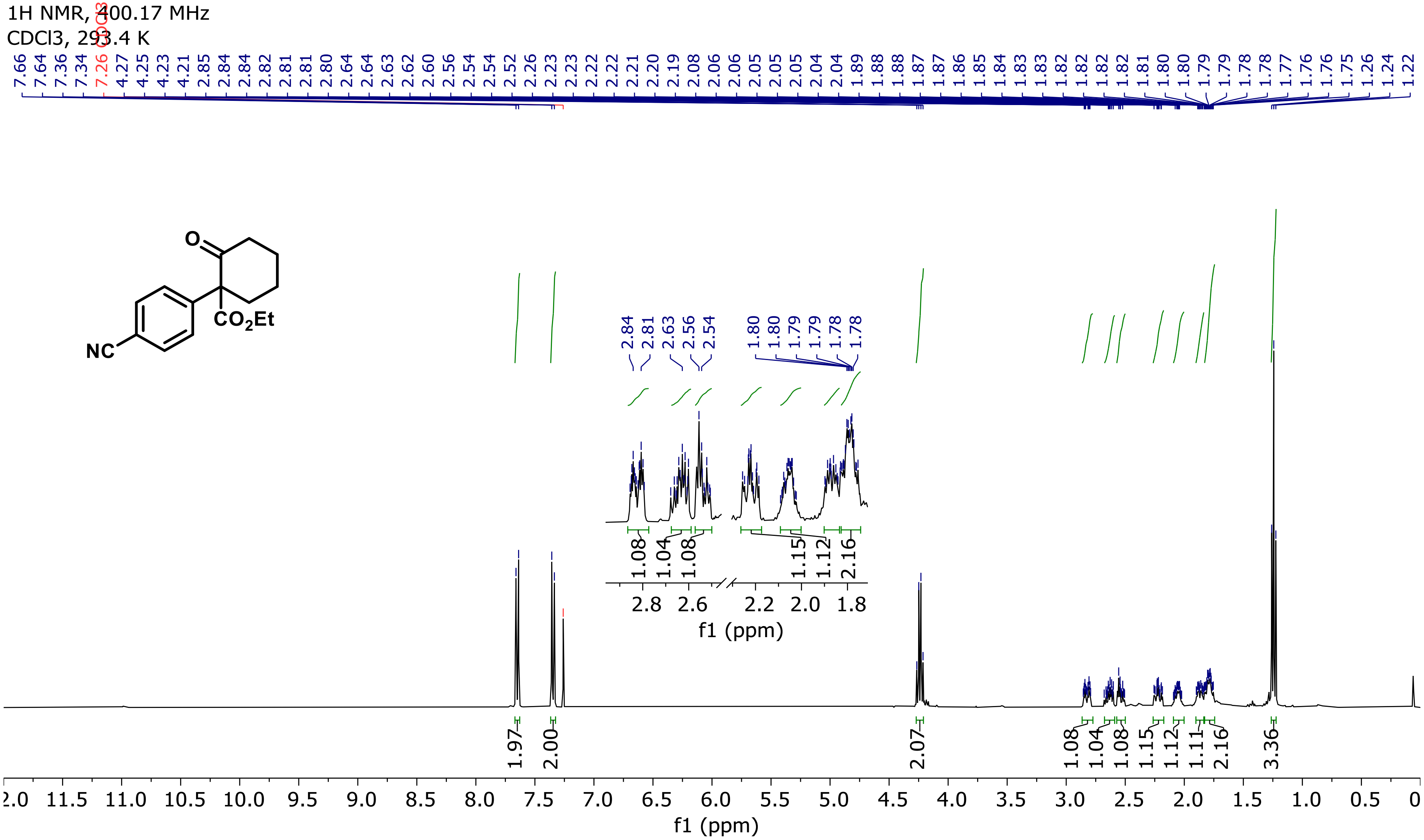

**$^{13}$C NMR of ethyl 1-(4-cyanophenyl)-2-oxocyclohexane-1-carboxylate (80)**

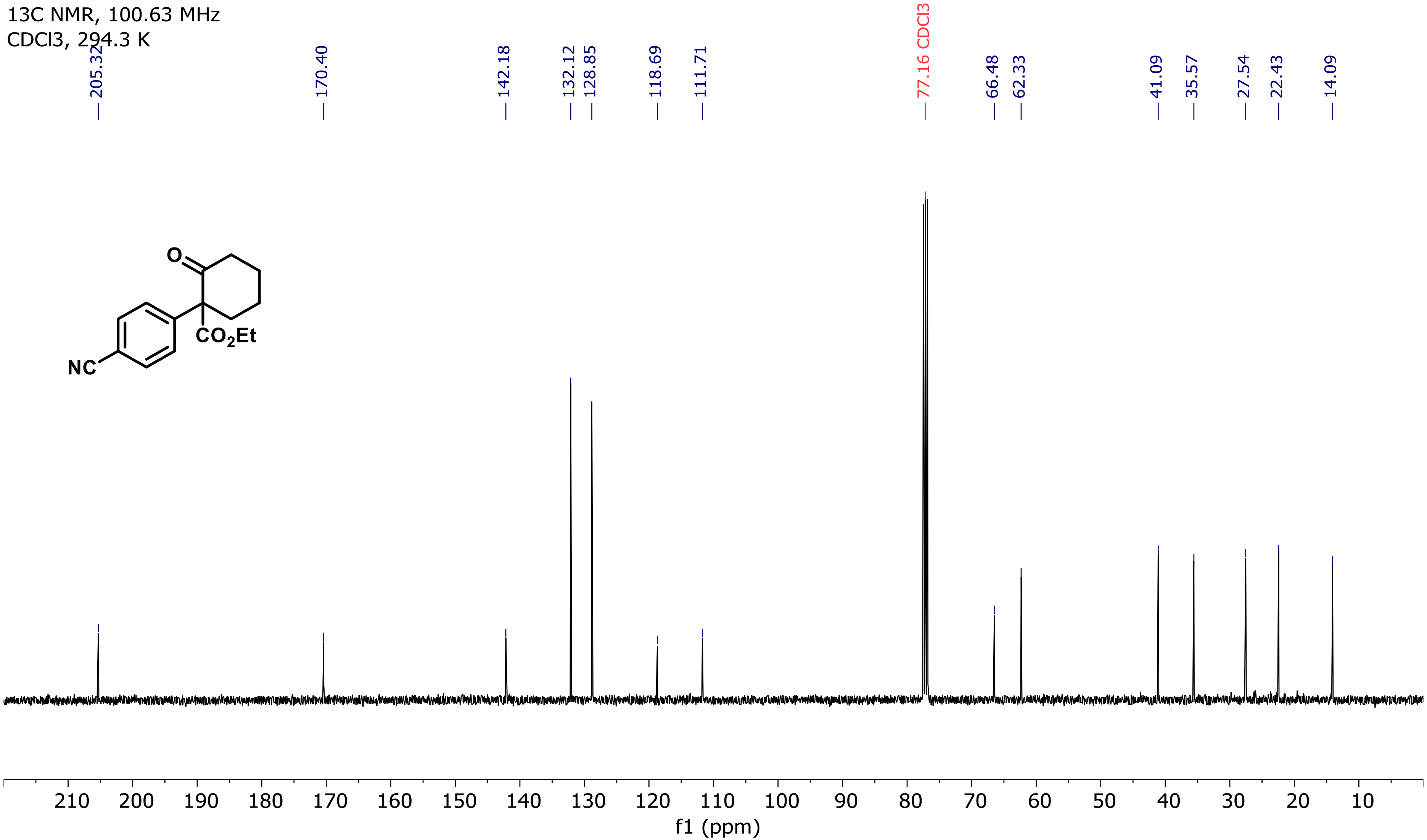

**$^{1}$H NMR of ethyl 2-cyano-2-(4-cyanophenyl)acetate (82)**

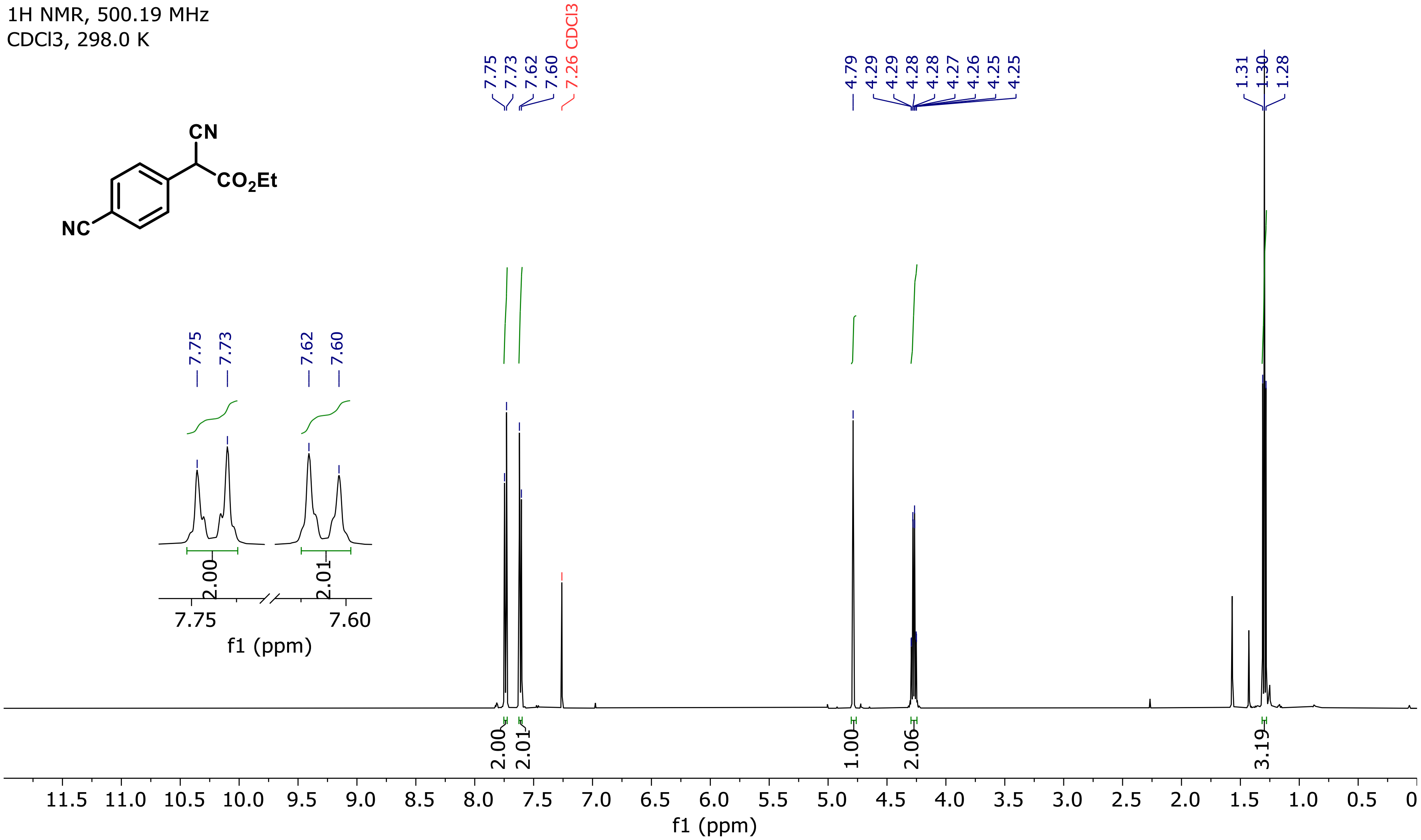

**$^{13}$C NMR of ethyl 2-cyano-2-(4-cyanophenyl)acetate (82)**

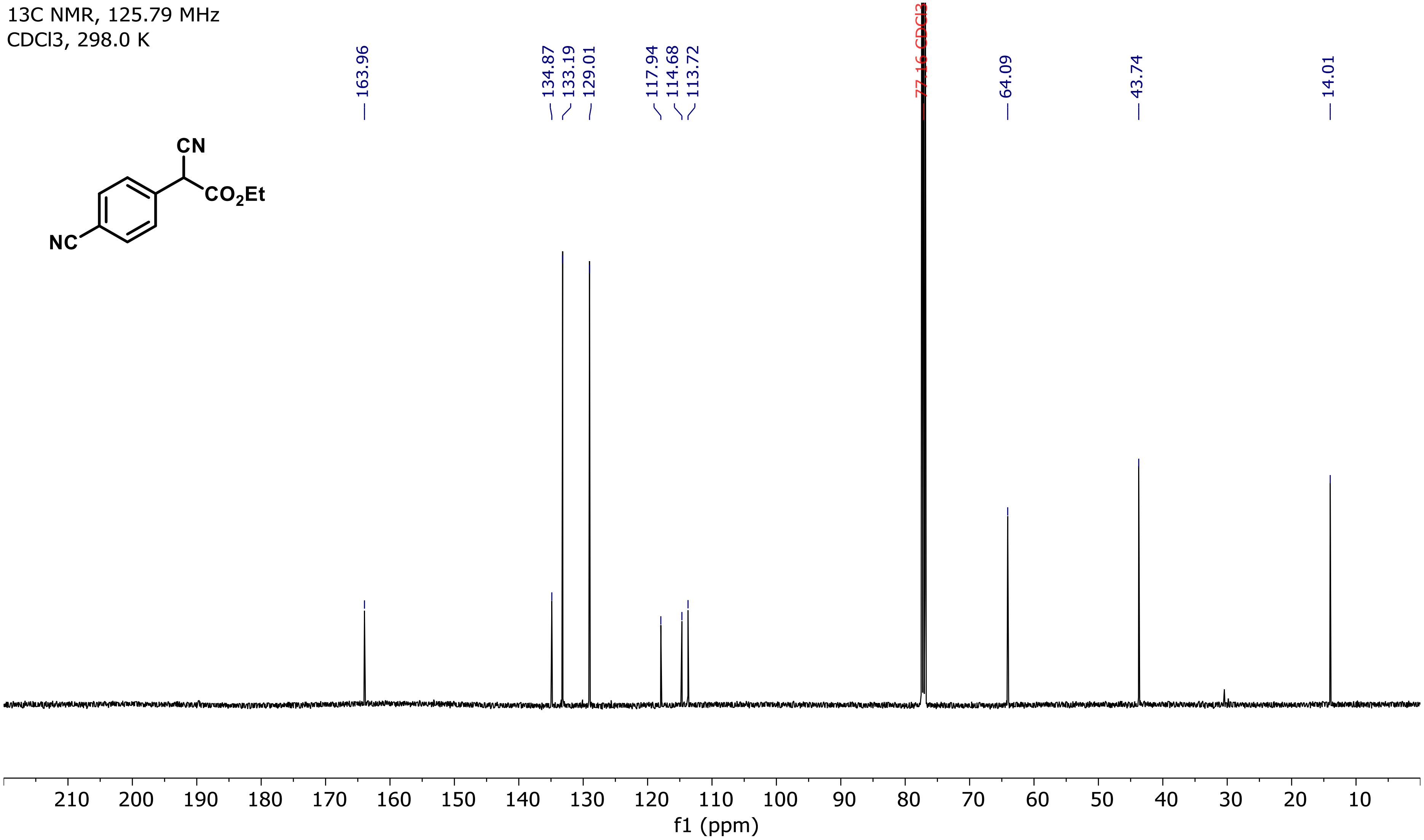

**$^{1}$H NMR of diethyl 2-(4-cyanophenyl)malonate (86)**

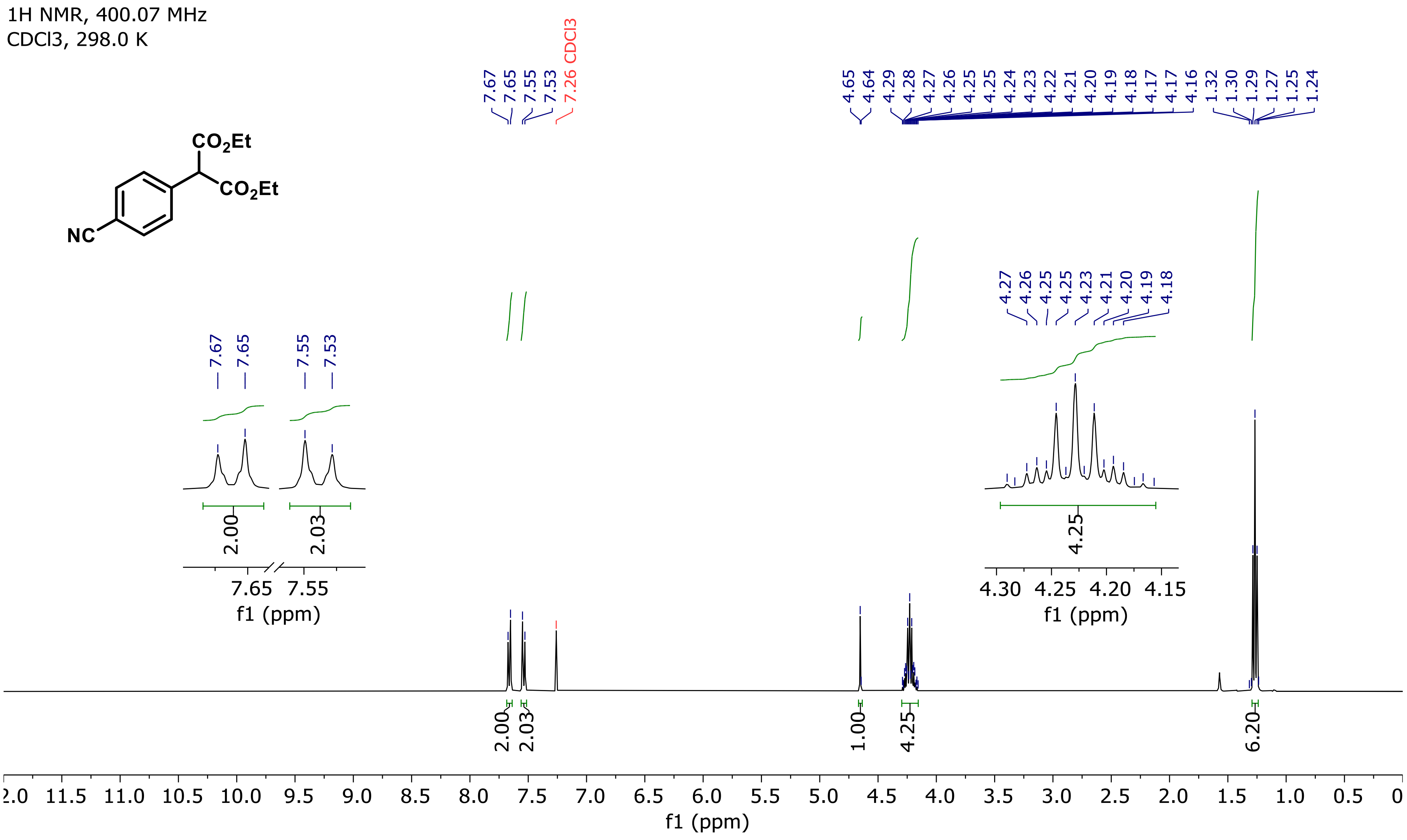

**$^{13}$C NMR of diethyl 2-(4-cyanophenyl)malonate (86)**

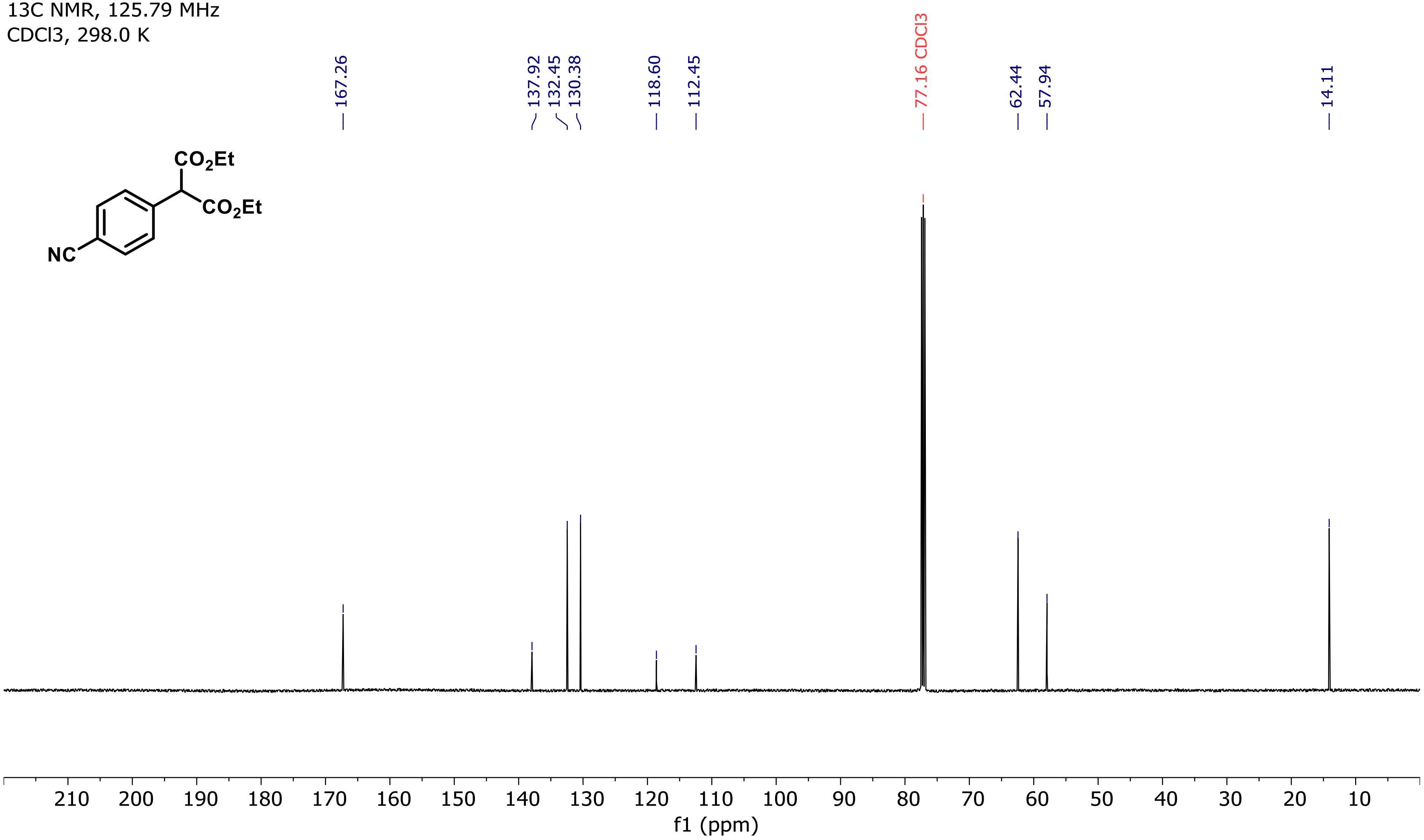

**$^{1}$H NMR of di-*tert*-butyl 2-(4-cyanophenyl)malonate (87)**

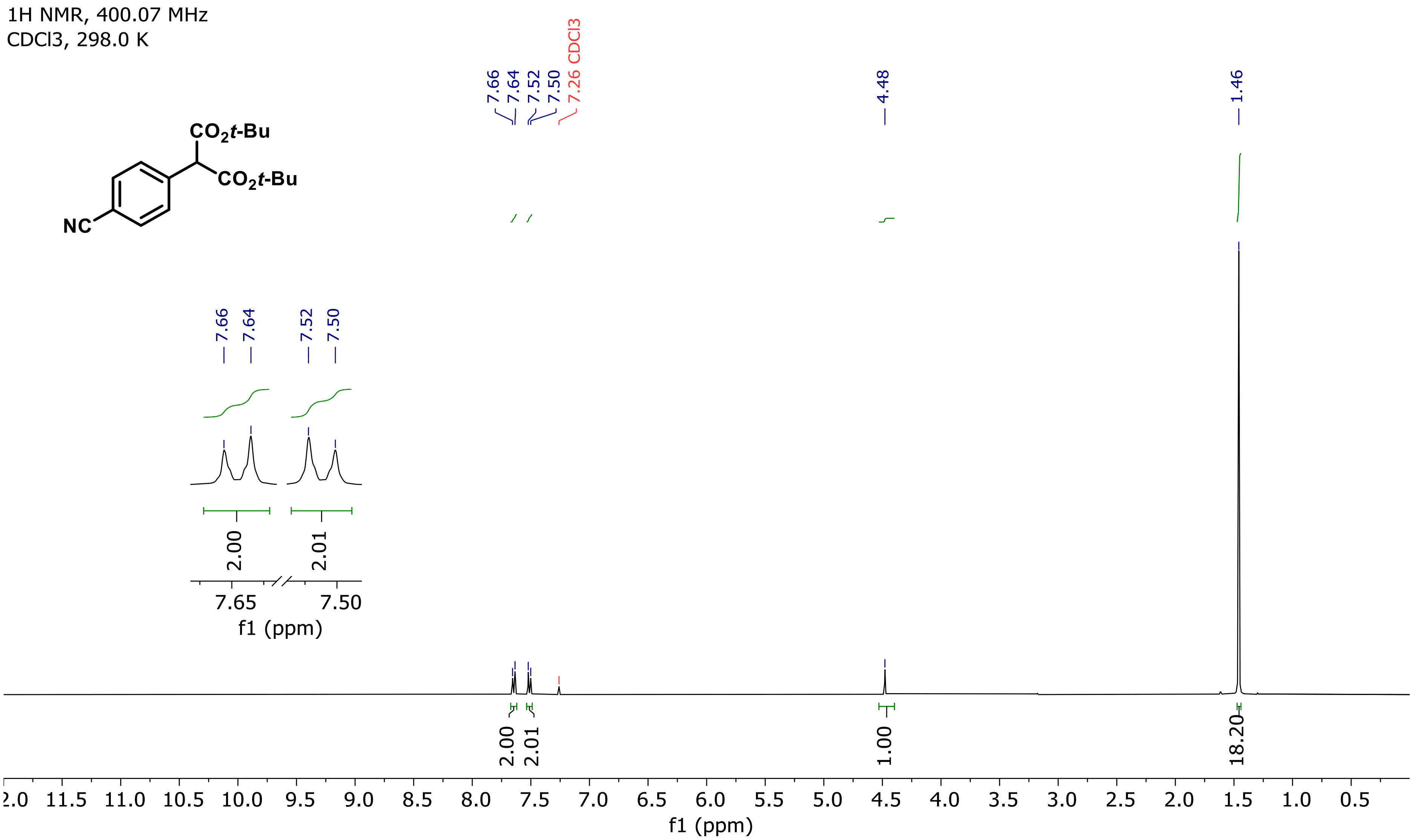

**$^{13}$C NMR of di-*tert*-butyl 2-(4-cyanophenyl)malonate (87)**

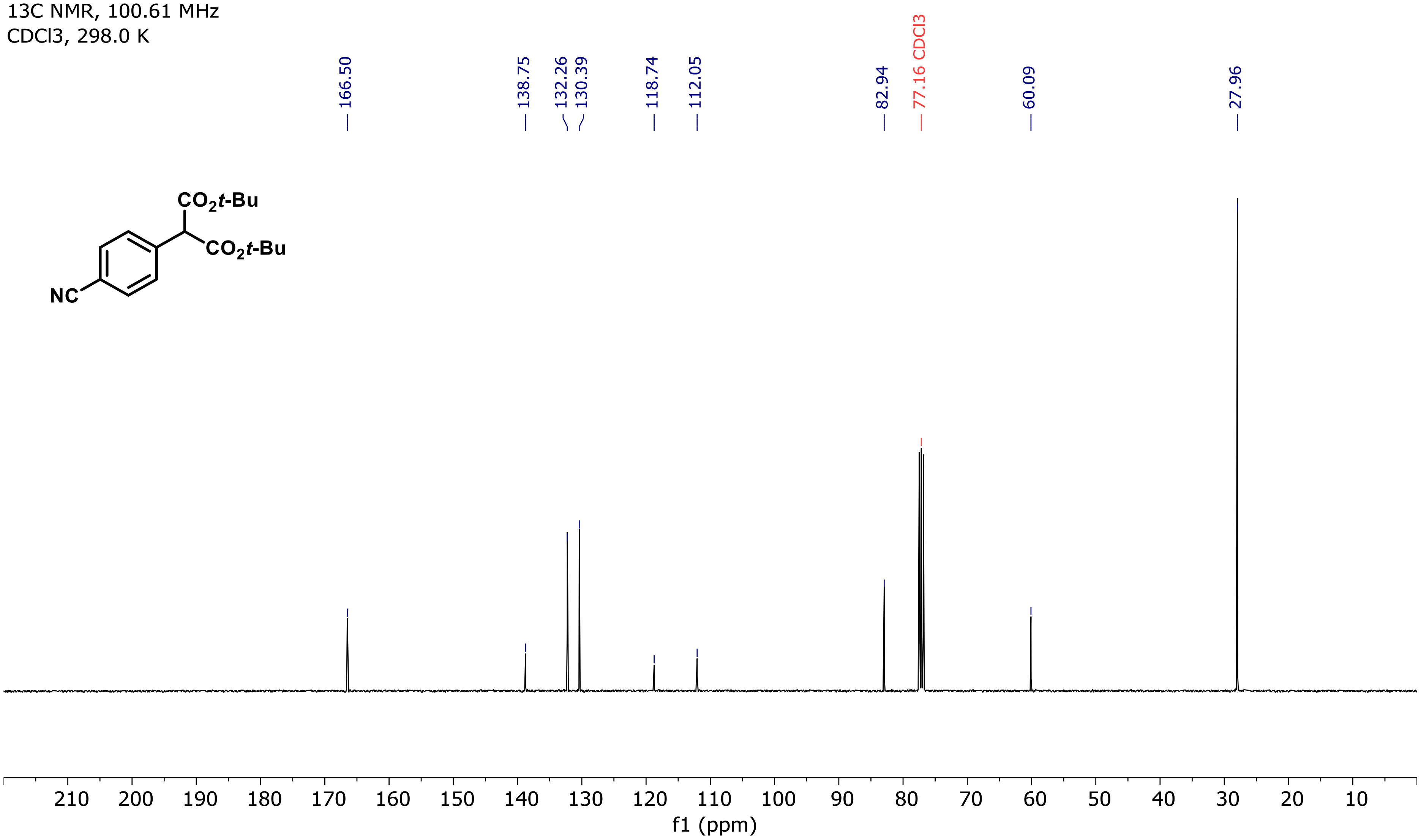

**$^{1}$H NMR of diethyl 2-(4-cyanophenyl)-2-methylmalonate (88)**

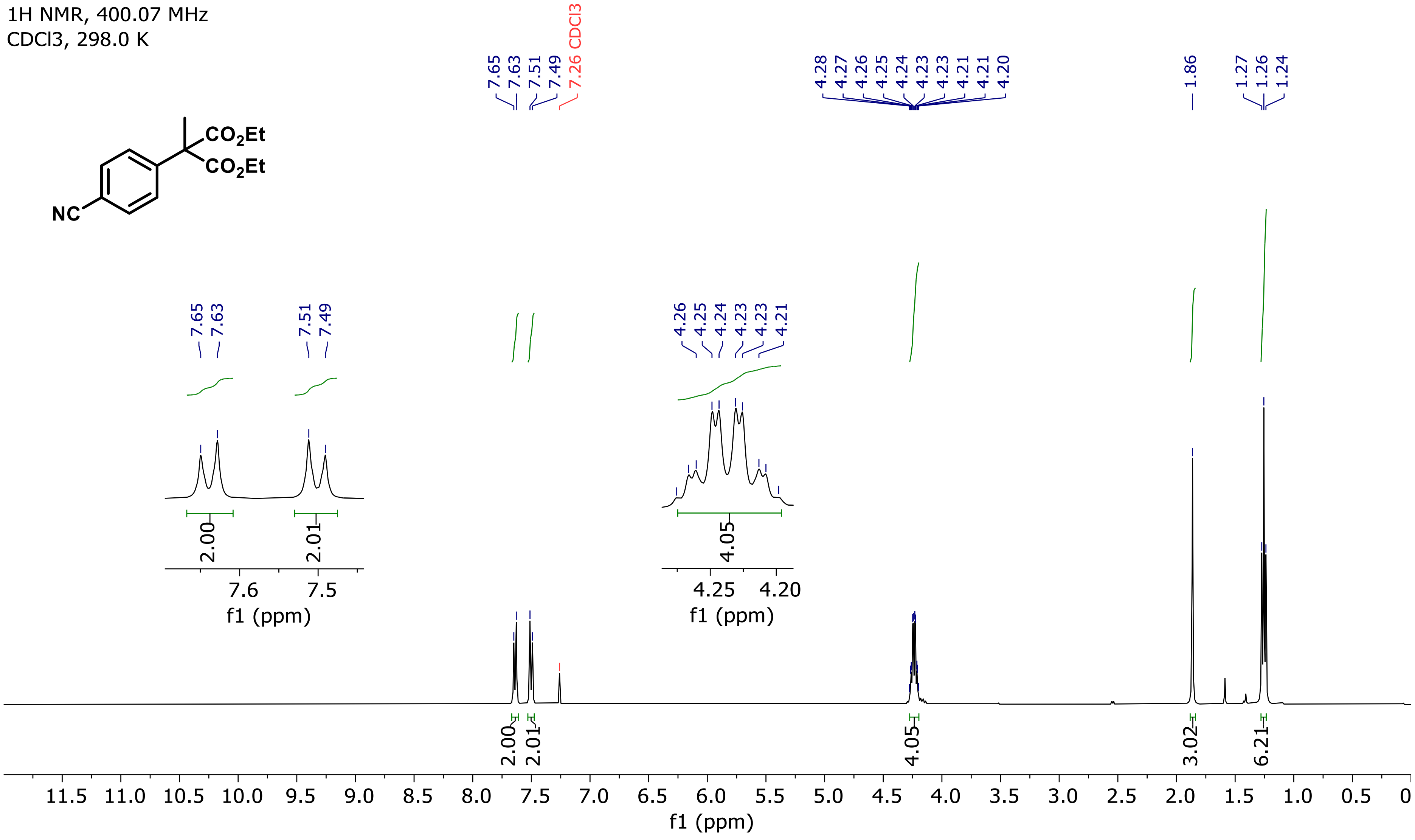

**$^{13}$C NMR of diethyl 2-(4-cyanophenyl)-2-methylmalonate (88)**

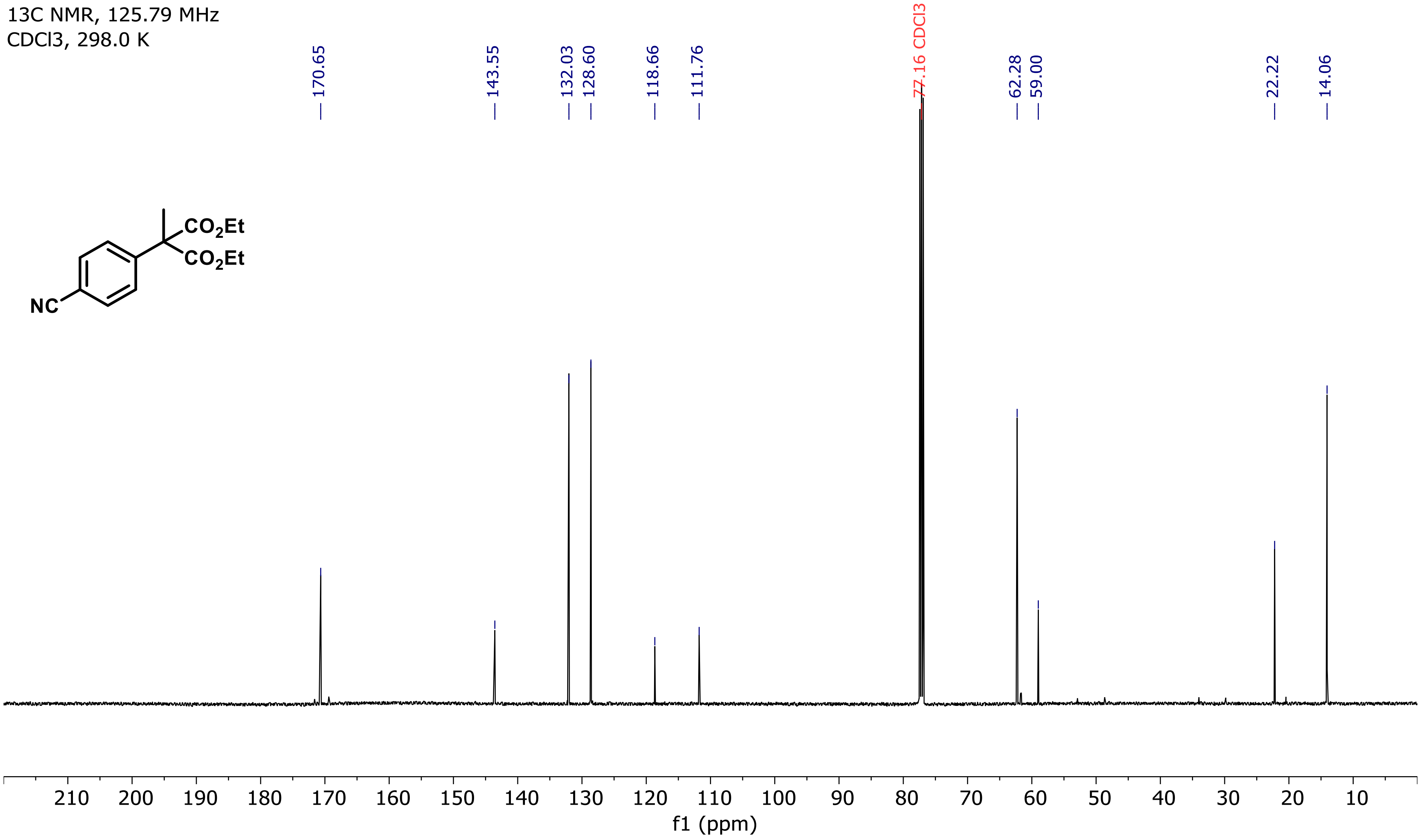

**$^{1}$H NMR of ethyl 2-(4-cyanophenyl)-2-((diphenylmethylene)amino)acetate (90)**

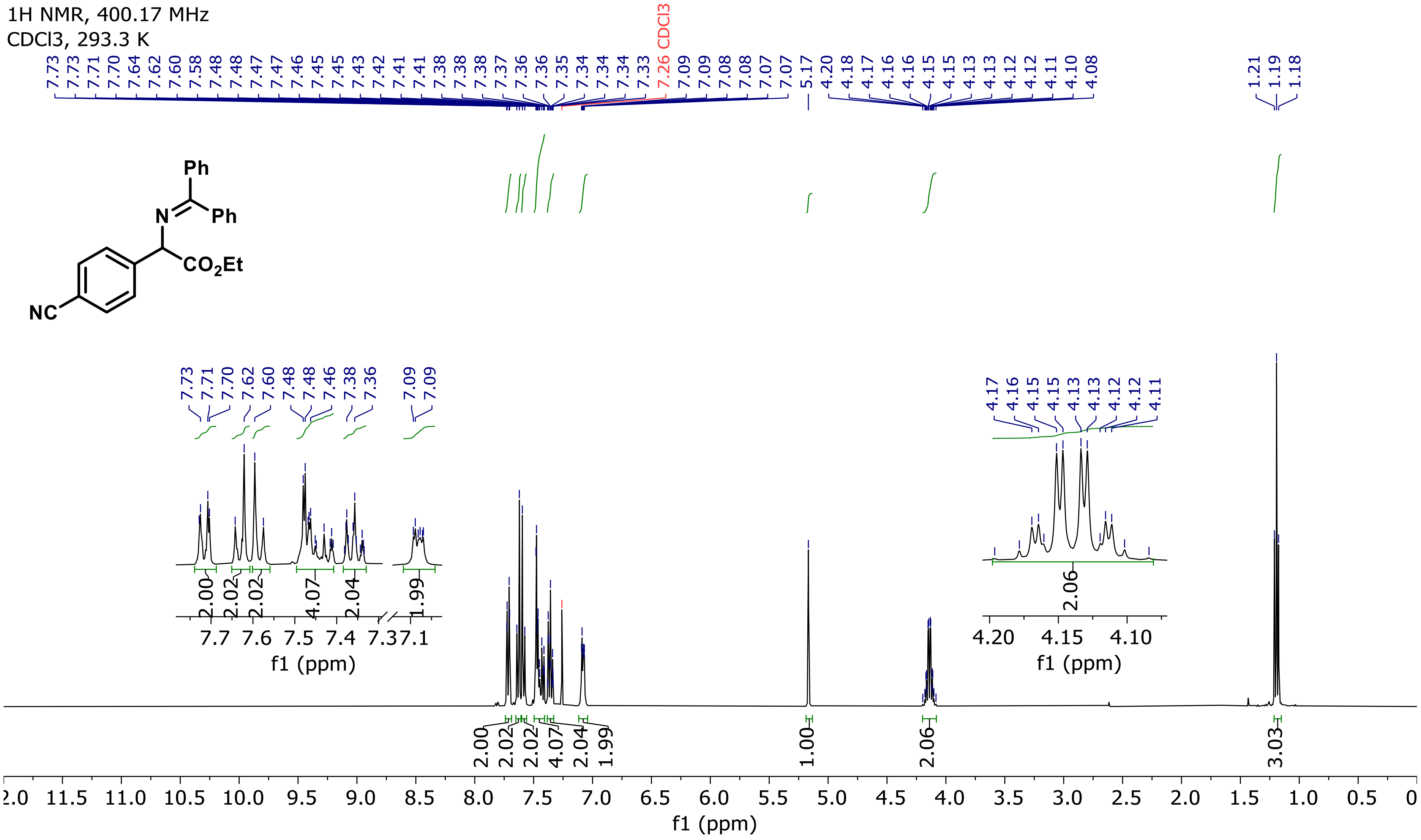

**$^{13}$C NMR of ethyl 2-(4-cyanophenyl)-2-((diphenylmethylene)amino)acetate (90)**

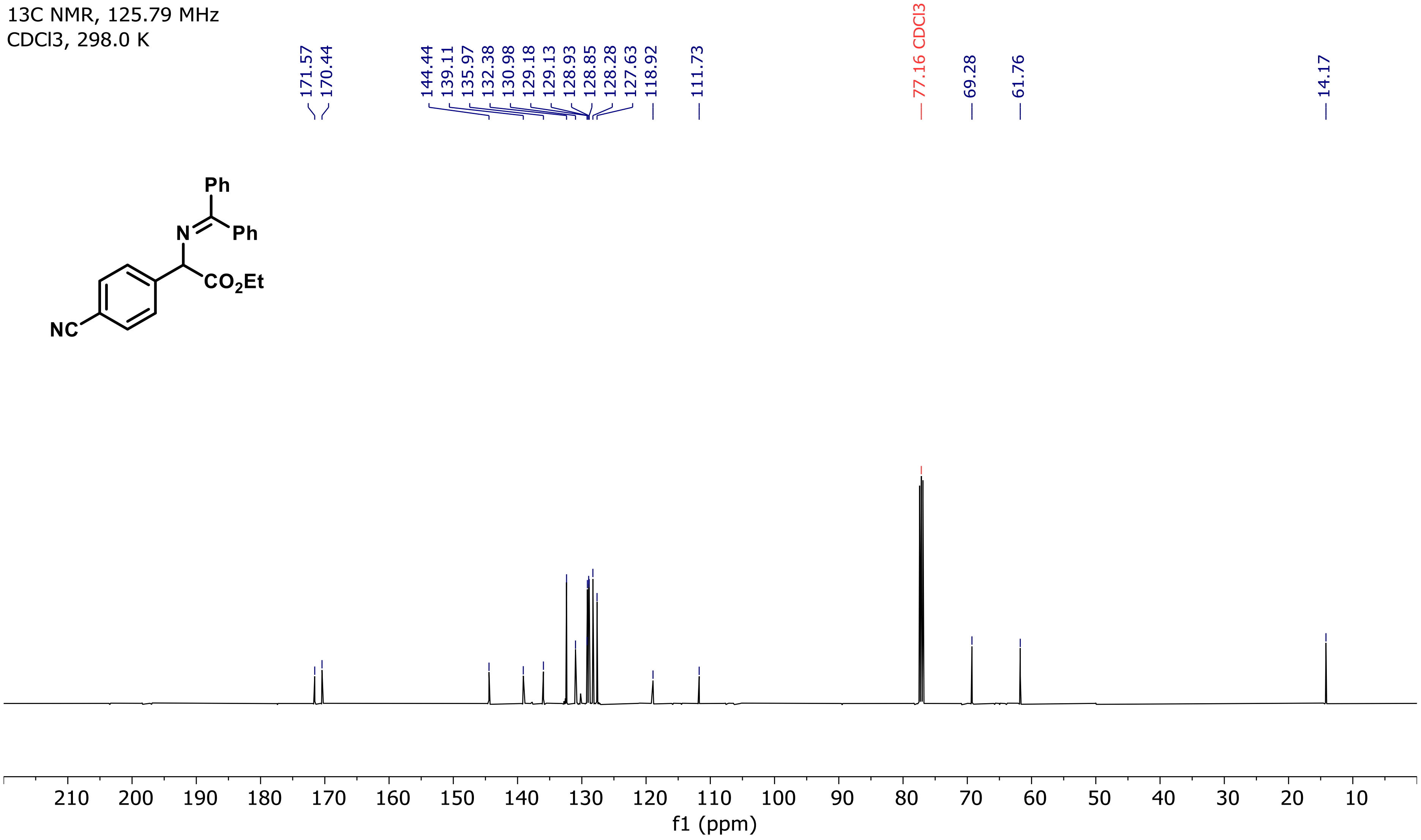

**$^{1}$H NMR of 4-(2-oxocyclohexyl)benzonitrile (92)**

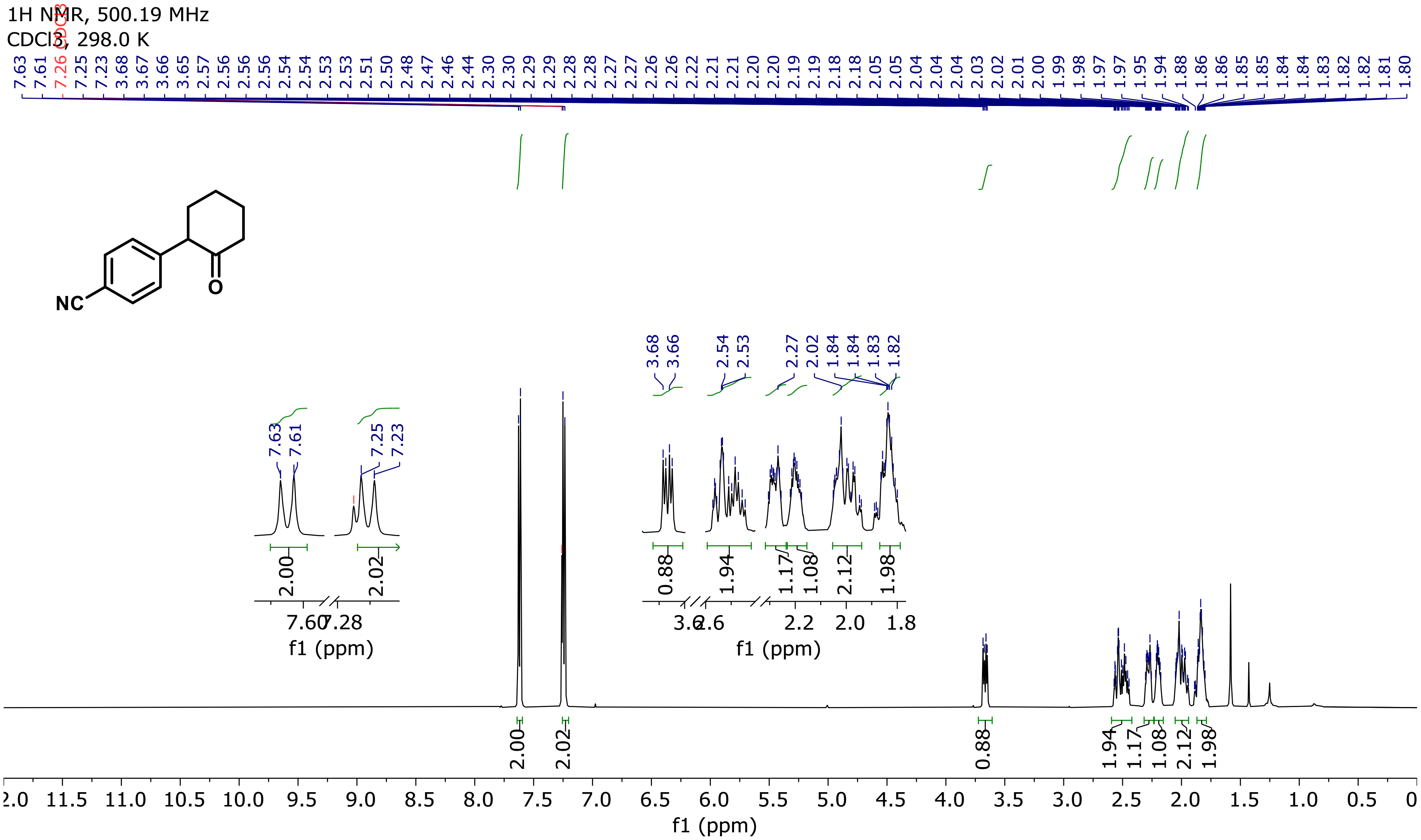

**$^{13}$C NMR of 4-(2-oxocyclohexyl)benzonitrile (92)**

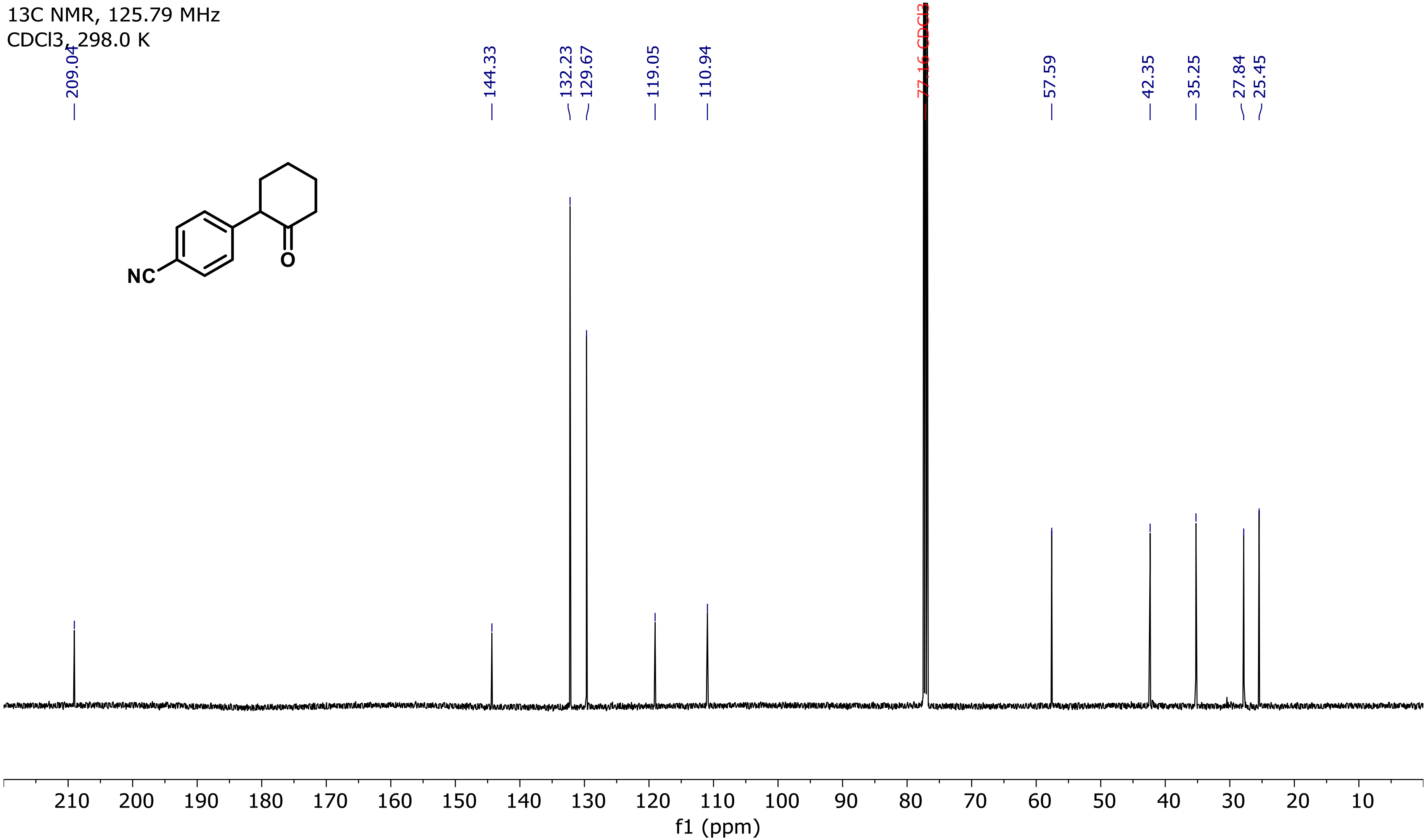

**$^{1}$H NMR of [1,1'-biphenyl]-4-yl(p-tolyl)sulfane (94)**

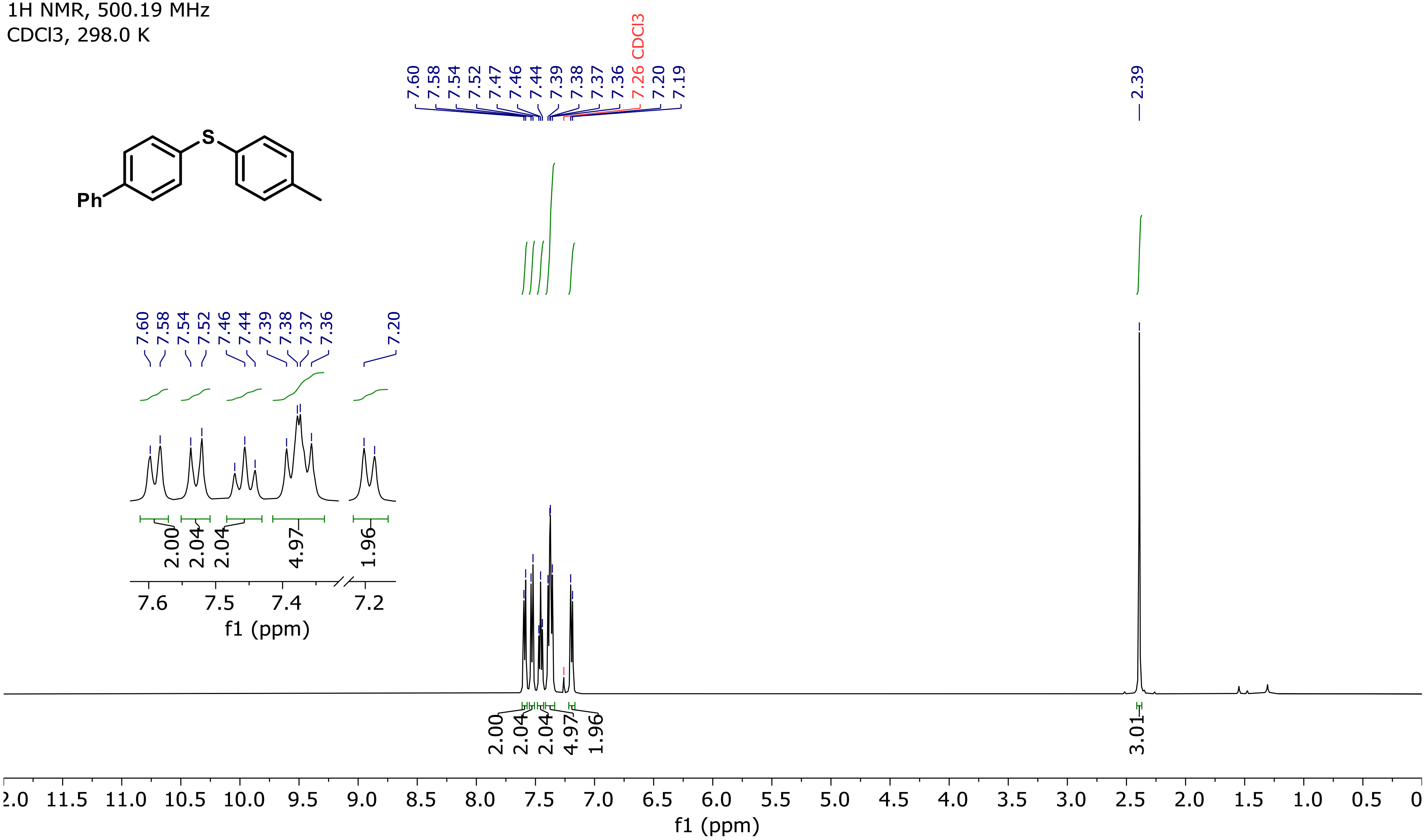

$^{13}$C NMR of [1,1'-biphenyl]-4-yl(p-tolyl)sulfane (94)

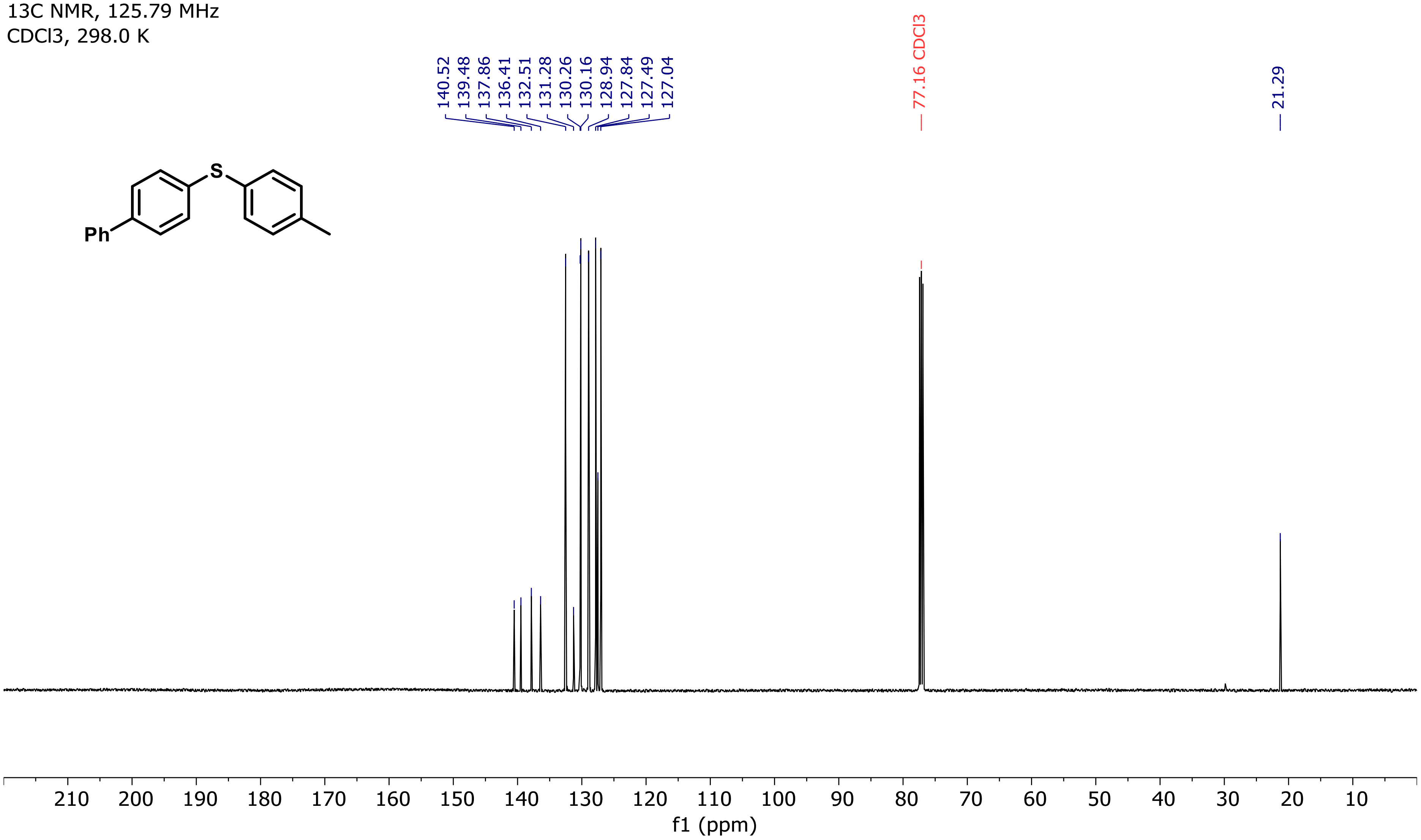

**$^{1}$H NMR of phenyl(4-(4,4,5,5-tetramethyl-1,3,2-dioxaborolan-2-yl)phenyl)methanone (97)**

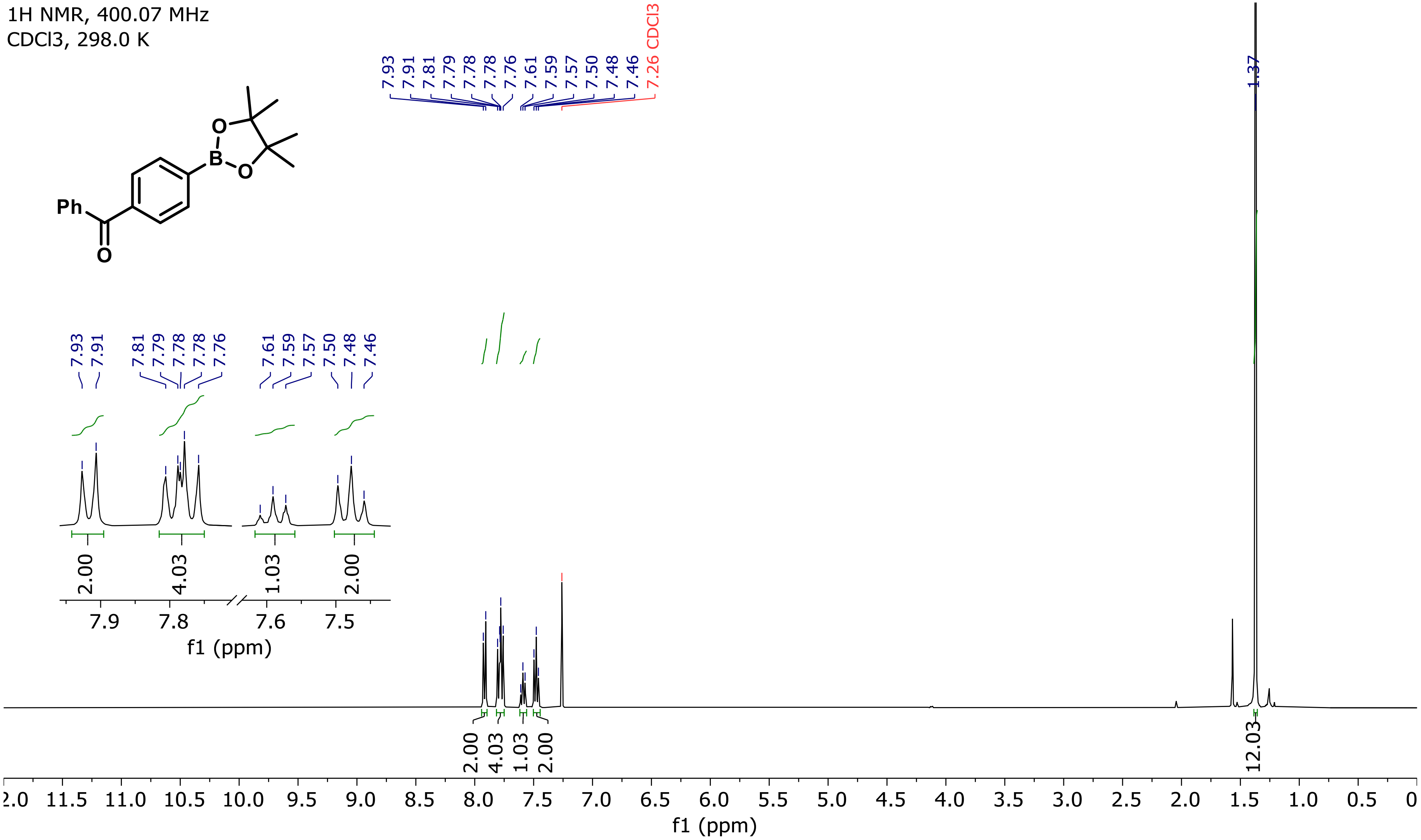

**$^{13}$C NMR of phenyl(4-(4,4,5,5-tetramethyl-1,3,2-dioxaborolan-2-yl)phenyl)methanone (97)**

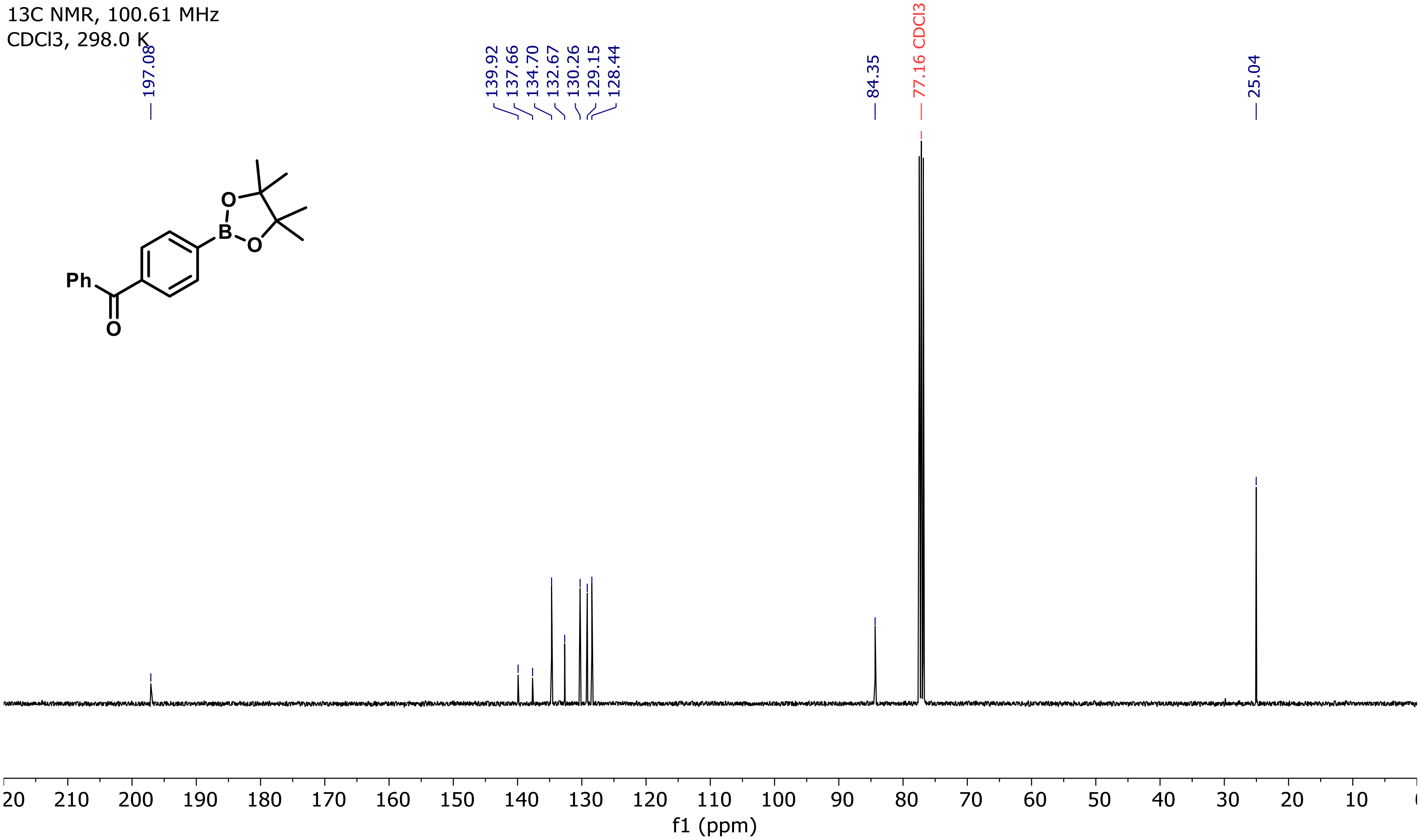

$^{1}$H NMR of diethyl (4-benzoylphenyl)phosphonate (98)

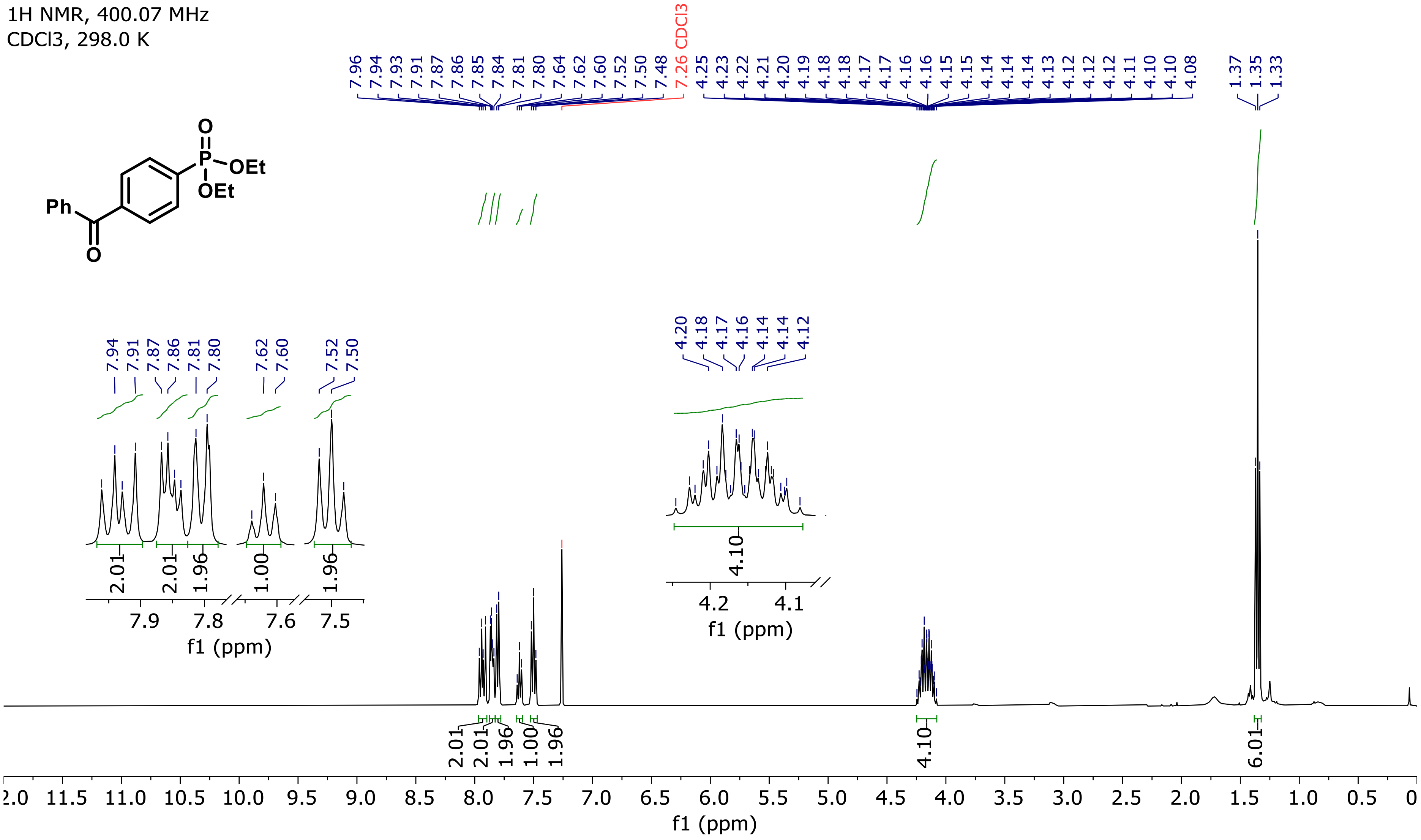

**$^{13}$C NMR of diethyl (4-benzoylphenyl)phosphonate (98)**

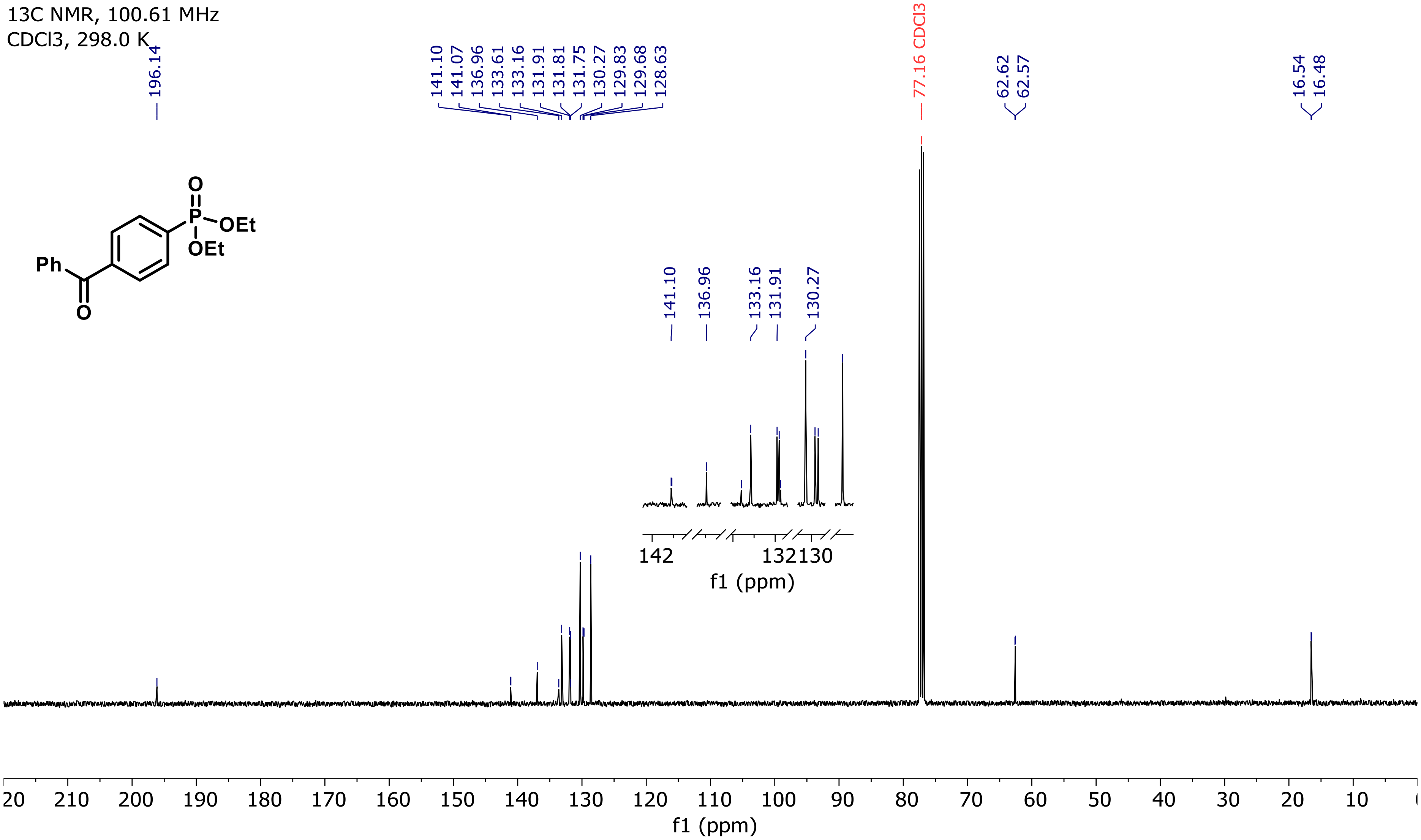

**$^{31}$P NMR of diethyl (4-benzoylphenyl)phosphonate (98)**

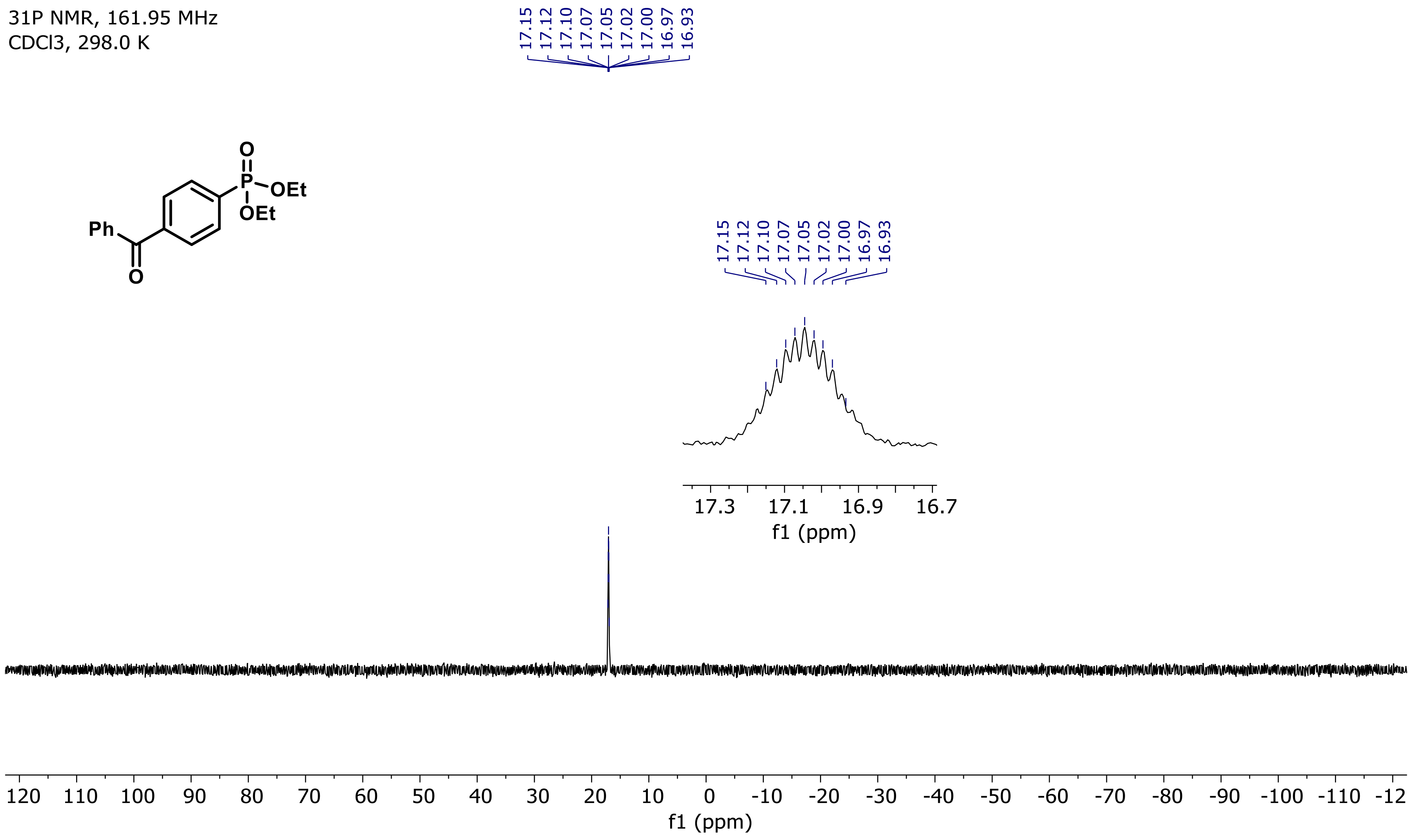

**$^{1}$H NMR of ethyl 2-(2-(2,2,2-trifluoroethoxy)pyrimidin-5-yl)acetate (S3)**

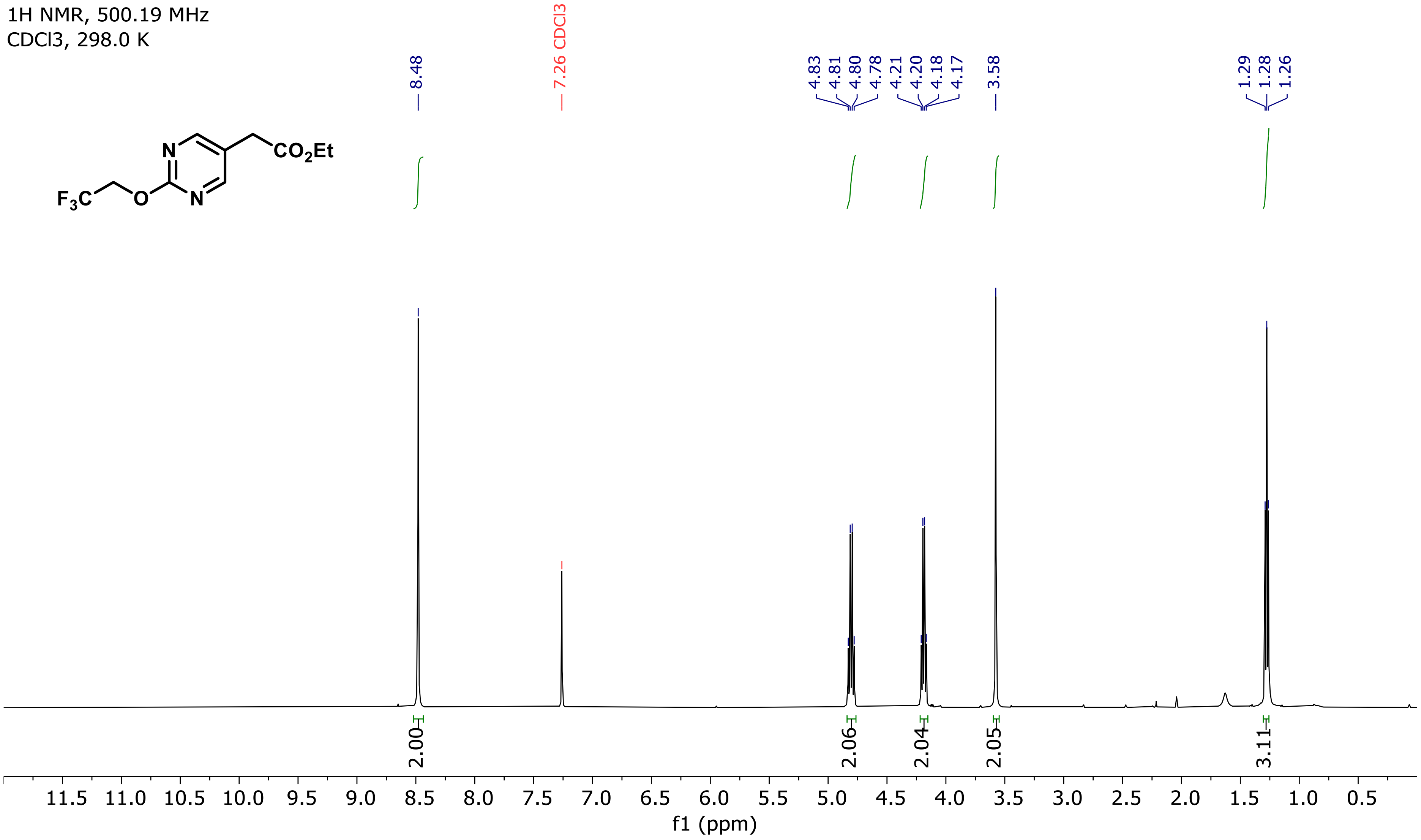

# $^{13}$C NMR of ethyl 2-(2-(2,2,2-trifluoroethoxy)pyrimidin-5-yl)acetate (S3)

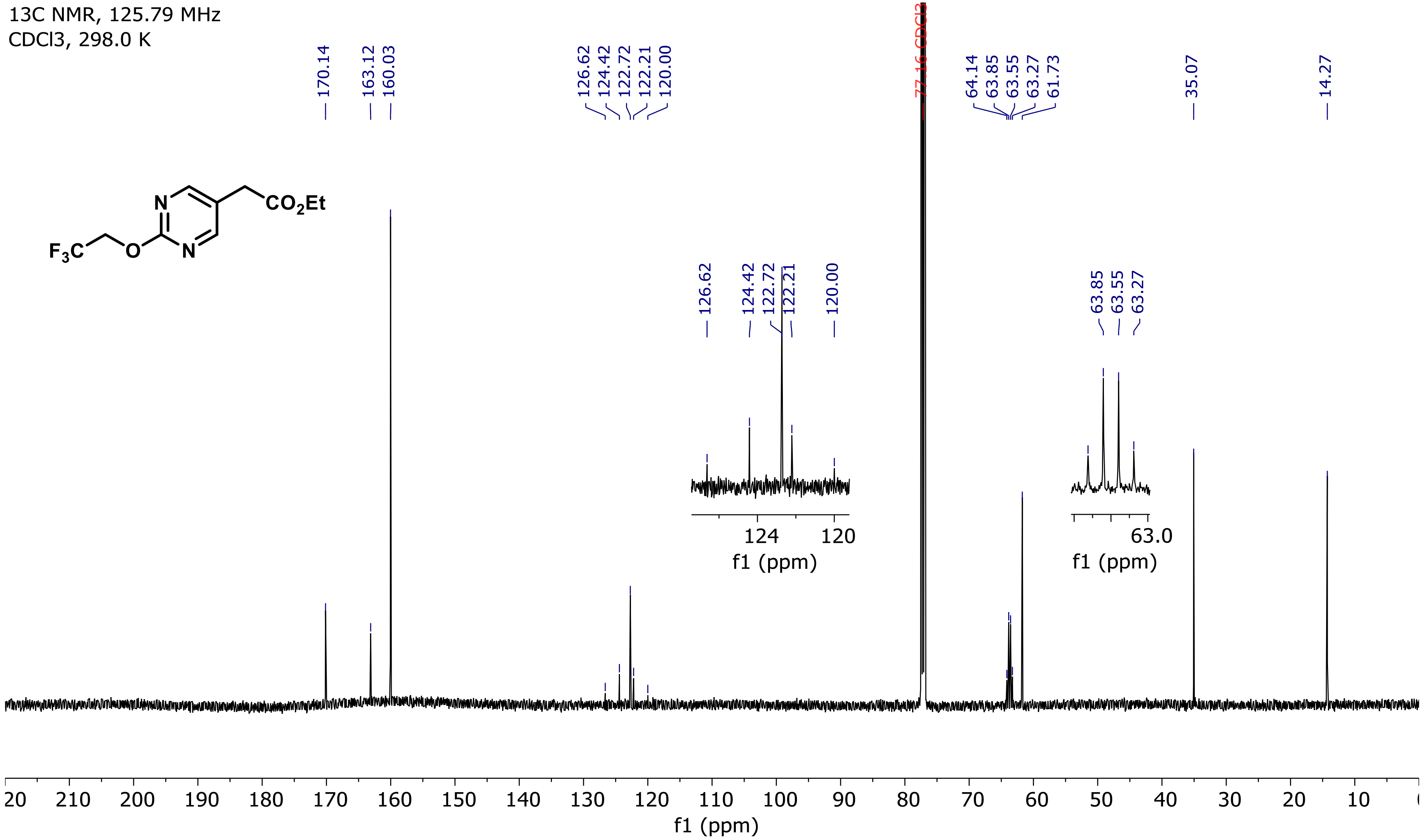

**$^{19}$F NMR of ethyl 2-(2-(2,2,2-trifluoroethoxy)pyrimidin-5-yl)acetate (S3)**

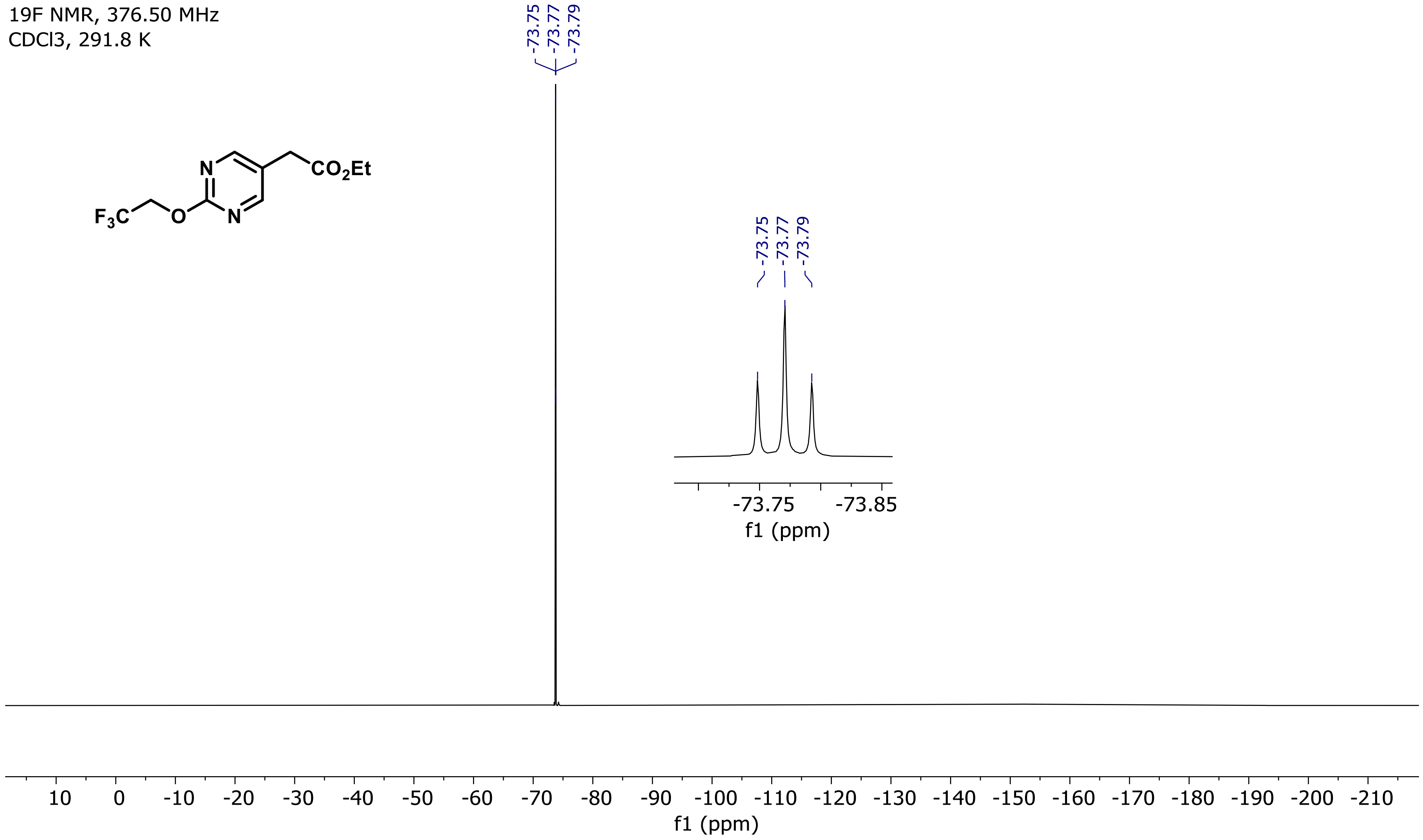

## $^{1}$H NMR of ethyl 2-[3-[(1S)-1-imidazo[1,2-a]pyridin-6-ylethyl]triazolo[4,5-b]pyrazin-5-yl]acetate (S4)

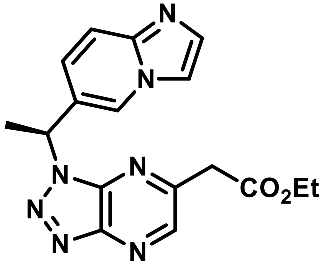

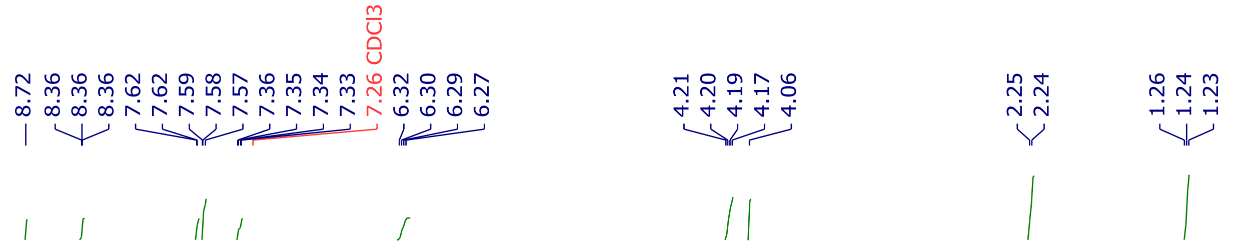

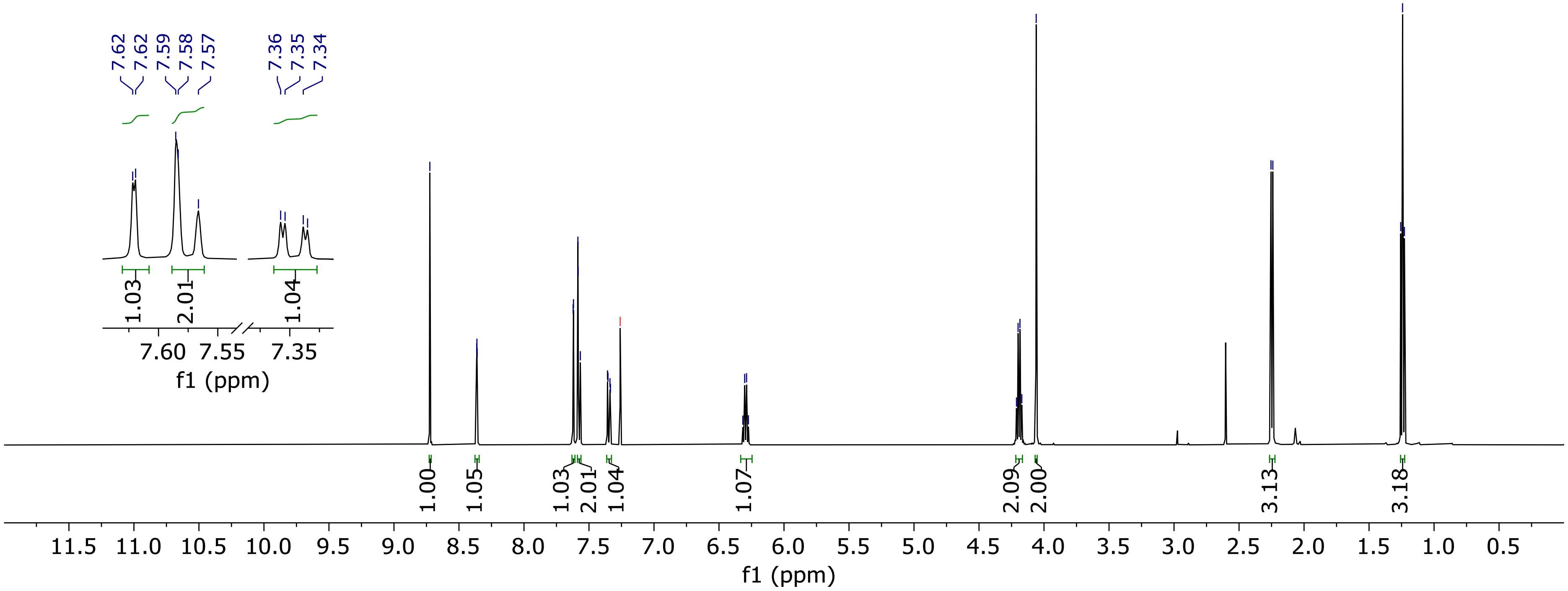

**$^{13}$C NMR of ethyl 2-[3-[(1S)-1-imidazo[1,2-a]pyridin-6-ylethyl]triazolo[4,5-b]pyrazin-5-yl]acetate (S4)**

13C NMR, 125.71 MHz
CDCl3, 300.0 K

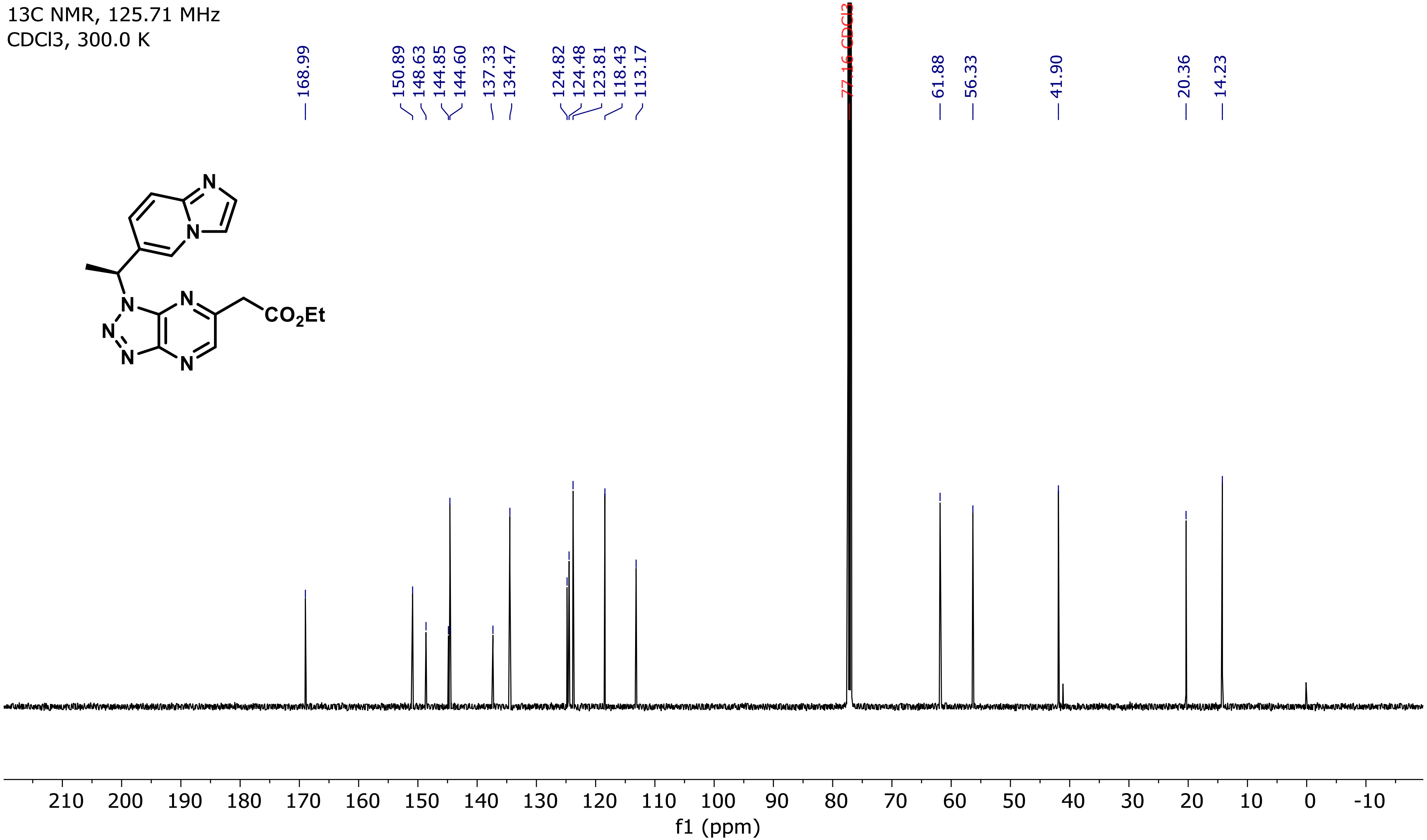

Supplement: Supplementary file 1 — Experimental details, Supplementary Figs. 1–12 and Tables 1–7. [file 44160_2025_919_MOESM1_ESM.pdf]
